# Supplementary material for: Self-care interventions for preconception, antenatal, intrapartum and postpartum care: a scoping review
Source: BMJ Open. 2023 May 9;13(5):e068713. doi: 10.1136/bmjopen-2022-068713 (PMC10173967; doi:10.1136/bmjopen-2022-068713)
Supplement: Supplementary data [file bmjopen-2022-068713supp002.pdf]

Supplementary Table 1. Evidence map for outcomes measured

Categories \ Outcome	Maternal mortality	Perinatal mortality	Neonatal outcomes	Labour outcomes	Anthropometric & cardiometabolic outcomes	Diabetes & other metabolic disorders	Hypertensive disorders of pregnancy	Postpartum infection & haemorrhage	Urogenital outcomes	Nutritional deficiencies	Gastrointestinal outcomes	Other maternal complications	Infectious diseases	Other minor physiological discomforts	Mental wellbeing & quality of life	Behavioural change	Breastfeeding	Other
Diet & nutrition	5	29	71	27	53	47	34	4	9	11	12	14	3	3	21	42	7	0
Physical activity	0	6	48	33	58	45	22	7	5	1	1	9	0	13	40	50	7	0
Lifestyle adjustments	0	9	16	1	0	1	4	0	1	0	1	5	12	9	11	49	1	0
Complementary & alternative therapies	0	3	15	26	1	1	2	2	14	0	6	3	0	10	23	1	8	0
Psycho-social strategies	0	3	16	23	1	6	5	1	4	0	1	4	0	9	75	8	7	2
Breast & nipple care	0	3	6	4	0	2	1	1	0	0	0	0	0	16	1	2	17	1
Sexual health & family planning	0	0	1	0	0	0	0	0	0	0	0	1	1	0	7	9	0	0
Self-monitoring	3	18	39	28	23	27	24	3	0	0	0	8	0	0	15	17	1	1
Self-management of medication	6	4	7	0	1	0	1	5	0	0	0	4	4	0	1	12	0	0
Self-testing or sampling	1	2	4	1	1	0	1	0	0	0	0	0	1	0	0	5	0	1
Other self-management skills	5	5	16	27	0	1	5	17	3	0	0	10	0	1	17	4	1	3
**All self-care categories**	**20<br>3%**	**67<br>12%**	**177<br>31%**	**125<br>22%**	**78<br>13%**	**79<br>14%**	**65<br>11%**	**33<br>6%**	**32<br>6%**	**11<br>2%**	**17<br>3%**	**43<br>7%**	**20<br>3%**	**46<br>8%**	**150<br>26%**	**147<br>25%**	**34<br>6%**	**8<br>1%**

Note: Number in each cell denotes the number of studies featuring the corresponding self-care category (row) and measuring the corresponding outcome (column). Percentages are based on total studies (N=580). For further details on the definition of each outcome category, see Supplementary File S5
