# Supplementary material for: Self-care interventions for preconception, antenatal, intrapartum and postpartum care: a scoping review
Source: BMJ Open. 2023 May 9;13(5):e068713. doi: 10.1136/bmjopen-2022-068713 (PMC10173967; doi:10.1136/bmjopen-2022-068713)
Supplement: Author's manuscript [file bmjopen-2022-068713.draft_revisions.pdf]

BMJ Open

BMJ Open is committed to open peer review. As part of this commitment we make the peer review history of every article we publish publicly available.

When an article is published we post the peer reviewers' comments and the authors' responses online. We also post the versions of the paper that were used during peer review. These are the versions that the peer review comments apply to.

The versions of the paper that follow are the versions that were submitted during the peer review process. They are not the versions of record or the final published versions. They should not be cited or distributed as the published version of this manuscript.

BMJ Open is an open access journal and the full, final, typeset and author-corrected version of record of the manuscript is available on our site with no access controls, subscription charges or pay-per-view fees (http://bmjopen.bmj.com).

If you have any questions on BMJ Open's open peer review process please email info.bmjopen@bmj.com

BMJ Open

Self-care interventions for preconception, antenatal, intrapartum and postpartum care: a scoping review

Journal:	*BMJ Open*
Manuscript ID	bmjopen-2022-068713
Article Type:	Original research
Date Submitted by the Author:	27-Sep-2022

Complete List of Authors:	Nguyen, Phi-Yen; Burnet Institute, International Development; Monash University, School of Public Health and Preventive Medicine<br>Caddy, Cassandra; Burnet Institute, International Development<br>Wilson, Alyce; Burnet Institute, International Development<br>Blackburn, Kara; Burnet Institute, International Development<br>Page, Matthew; Monash University, School of Public Health and Preventive Medicine<br>Gülmezoglu, Ahmet Metin ; Concept Foundation<br>Narasimhan, Manjulaa; World Health Organization, Department of Reproductive Health and Research<br>Bonet, Mercedes; World Health Organization, Department of Reproductive Health and Research<br>Tunçalp, Özge; World Health Organization, Department of Reproductive Health and Research<br>Vogel, Joshua P.; Burnet Institute, International Development

Keywords:	Antenatal < GENETICS, PUBLIC HEALTH, SEXUAL MEDICINE, REPRODUCTIVE MEDICINE, Maternal medicine < OBSTETRICS, Health policy < HEALTH SERVICES ADMINISTRATION & MANAGEMENT

SCHOLARONE™
Manuscripts

I, the Submitting Author has the right to grant and does grant on behalf of all authors of the Work (as defined in the below author licence), an exclusive licence and/or a non-exclusive licence for contributions from authors who are: i) UK Crown employees; ii) where BMJ has agreed a CC-BY licence shall apply, and/or iii) in accordance with the terms applicable for US Federal Government officers or employees acting as part of their official duties; on a worldwide, perpetual, irrevocable, royalty-free basis to BMJ Publishing Group Ltd ("BMJ") its licensees and where the relevant Journal is co-owned by BMJ to the co-owners of the Journal, to publish the Work in this journal and any other BMJ products and to exploit all rights, as set out in our licence.

The Submitting Author accepts and understands that any supply made under these terms is made by BMJ to the Submitting Author unless you are acting as an employee on behalf of your employer or a postgraduate student of an affiliated institution which is paying any applicable article publishing charge ("APC") for Open Access articles. Where the Submitting Author wishes to make the Work available on an Open Access basis (and intends to pay the relevant APC), the terms of reuse of such Open Access shall be governed by a Creative Commons licence – details of these licences and which Creative Commons licence will apply to this Work are set out in our licence referred to above.

Other than as permitted in any relevant BMJ Author's Self Archiving Policies, I confirm this Work has not been accepted for publication elsewhere, is not being considered for publication elsewhere and does not duplicate material already published. I confirm all authors consent to publication of this Work and authorise the granting of this licence.

For peer review only

Self-care interventions for preconception, antenatal, intrapartum and postpartum care: a scoping review

Phi-Yen Nguyen[1,2], Cassandra Caddy[1], Alyce N. Wilson[1], Kara Blackburn[1], Matthew J. Page[2], A. Metin Gülmezoglu[3], Manjulaa Narasimhan[4], Mercedes Bonet [4], Özge Tunçalp[4], Joshua P. Vogel[1 *]

1. Maternal, Child and Adolescent Health Program, Burnet Institute, Melbourne, Australia

2. Methods in Evidence Synthesis Unit, School of Public Health and Preventive Medicine, Monash University, Melbourne, Australia

3. Concept Foundation, Geneva, Switzerland

4. Department of Sexual and Reproductive Health and Research, including UNDP/UNFPA/UNICEF/WHO/World Bank Special Programme of Research, Development and Research Training in Human Reproduction (HRP), World Health Organization, Geneva, Switzerland

***Correspondence to:** A/P Joshua P. Vogel, Global Women's and Newborn's Health Group, Burnet Institute, 85 Commercial Rd, Melbourne, Victoria, Australia 3004.

Email address: joshua.vogel@burnet.edu

Total word count: 2,980 words (excluding tables and figures)

Keywords: self-care, pregnancy, preconception, antenatal, intrapartum, postpartum, postnatal, maternal outcomes, neonatal outcomes, infants, newborns

For peer review only - http://bmjopen.bmj.com/site/about/guidelines.xhtml

ABSTRACT (300 words)

Objective: To identify current and emerging self-care interventions with the aims of improving the quality or coverage of maternity health care, and/or improving women's health, well-being and care experiences.

Design: A systematic scoping review was conducted. Six databases were searched (Medline, Embase, Emcare, PsycINFO, Cochrane CENTRAL/CDSR, CINAHL Plus, from inception to 17 October, 2021) for eligible studies (evidence syntheses, interventional or observational studies). Screening and data collection were conducted independently by two reviewers.

Context: Preconception, antenatal, intrapartum and postpartum care

Participants: Women preparing to get pregnant, currently pregnant, giving birth, or in the postpartum period (up to 42 days after birth).

Intervention: Any tool, resource or strategy designed to promote or facilitate self-care

Results: Most included studies evaluated interventions in high-income countries (45%) and in the antenatal period (76%). From 545 eligible studies, 112 self-care interventions were identified and organised into 11 categories via an inductive approach. Self-care categories with the most evidence were diet and nutrition (26% of all studies), physical activity (24%), psycho-social strategies (18%) and other lifestyle adjustments such as sleeping positions, protection against mosquitoes or minimising air pollution exposure (17%). Limited evidence was identified for sexual health and postpartum family planning (2%), self-management of medication (3%), and self-testing/sampling (3%). Involvement of health workers and community health workers were described in 38% and 8% of studies, respectively, supporting self-care interventions through provision of therapeutics for home use, instruction information or via counselling. Use of digital platforms (e.g. mobile apps, websites) to provide self-care interventions were featured in 18% of studies.

Conclusions: Self-care interventions across the childbearing continuum are diverse in their applications and intended outcomes. Their implementation requires close engagement with health

For peer review only - http://bmjopen.bmj.com/site/about/guidelines.xhtml

workers and health systems. Future research is warranted into task-sharing aspects of clinical care that could be transferred to or supported at the home setting.

Strengths

- Broad searches of multiple self-care concepts and databases
- Systematic searching and screening processes
- Use of the new WHO self-care classification system to organise and categorise findings

Limitations

- Not including interventions that took place after 42 days postpartum, or those that related primarily to care of newborns and breastfeeding (which should require dedicated reviews)
- Search limited to published reports and reports written in English only

For peer review only

For peer review only - http://bmjopen.bmj.com/site/about/guidelines.xhtml

BACKGROUND

Self-care is defined by the World Health Organization (WHO) as "the ability of individuals, families and communities to promote health, prevent disease, maintain health, and to cope with illness and disability with or without the support of a health worker" [1]. Self-care may include, but is not limited to, health literacy, self-awareness of physical and mental health, physical activity, healthy eating, good hygiene, and rational use of medicines and diagnostics [2]. By extension, self-care interventions are those that can support or promote people's self-care [3]. Self-care interventions can act to complement or even broaden delivery of health services, ideally improving health outcomes and care experiences [4,5]. Humanitarian crises, emergencies and pandemics introduce additional challenges to the provision of routine healthcare services [6,7]. In this context, self-care interventions can help ensure health and well-being is maintained with reduced in-person contact [8].

In 2021, WHO published a guideline on self-care interventions for health and wellbeing, which included 13 recommendations on selected self-care interventions to improve preconception, antenatal, intrapartum and postpartum care [3]. By encouraging women to engage in these self-care interventions, the overall efficiency of healthcare delivery can be enhanced. In addition, there are interventions for pregnant and postpartum women that are not currently included in WHO's self-care guideline, but have the potential of transitioning into a self-care paradigm. Examples include prescription medicines that can be made available over-the-counter, self-collection of samples for testing, or home testing kits for certain diseases. Some systematic reviews have been conducted on selected self-care interventions in pregnancy [9–11]; however no previous review has explored the broad and heterogeneous body of research across preconception, antenatal, intrapartum and postpartum interventions, in order to identify those that may be effective or applicable from a self-care perspective. We therefore aimed to identify all current and emerging self-care interventions via a scoping review that have been evaluated in the context of preconception, antenatal, intrapartum and postpartum care.

For peer review only - http://bmjopen.bmj.com/site/about/guidelines.xhtml

METHODS

The scoping review was conducted in accordance with the methodological guide for scoping reviews by Peters and colleagues [12], and reported in accordance with the PRISMA guideline for Scoping Reviews [13]. The protocol was registered online at https://doi.org/10.17605/OSF.IO/97X25 (Supplementary File S1).

Electronic searches

The search strategy was developed in consultation with an information specialist (Supplementary File S2) and combined self-care concepts with terms related to preconception, antenatal, intrapartum and postpartum care, based on the framework proposed by Narasimhan and colleagues [4]. The terms and concepts used were informed by previous reviews of pregnancy care and self-care for other health conditions [14–16] and by inputs from team members with clinical and public health background. The following databases were systematically searched to identify published studies from inception to October 17th, 2021: Medline, Embase, Emcare and PsycINFO (via Ovid), CENTRAL/Cochrane Database of Systematic Reviews (via Cochrane Library) and CINAHL Plus (via EBSCOhost). Reference lists of included studies were manually screened to identify other relevant studies. Grey literature was not searched after considering the volume and variable quality of such evidence [17].

Study selection

Two reviewers (PN and CC) first independently screened all titles and abstracts to exclude irrelevant studies, and then independently reviewed potentially eligible full texts based on pre-determined eligibility criteria. All stages of screening were conducted using Covidence software [18]. Any disagreement was resolved by discussion or adjudication by a third reviewer (JPV).

For peer review only - http://bmjopen.bmj.com/site/about/guidelines.xhtml

The eligibility criteria for inclusion were: (A) Participants: women preparing to get pregnant, currently pregnant, giving birth, or in the postpartum period (up to 42 days after birth), of any age. Studies involving partners or family members in maternal care were also eligible. Interventions for abortion were not eligible. We also excluded interventions that were only directed at newborns and postnatal breastfeeding interventions for improving neonatal outcomes, though antenatal programmes related to preparation for the postnatal period were eligible. (B) Interventions: For this review, a self-care intervention was defined as any tool, resource or strategy designed to promote or facilitate self-care by a woman intending to get pregnant, or a currently pregnant, labouring or in the postpartum period, for the purpose of improving the quality or coverage of maternity health care, and/or improving her health, well-being and care experiences. To be eligible, interventions must have been aimed at reducing risks of pregnancy, childbirth or postpartum complications and/or enabling a positive pregnancy, childbirth or postpartum experience. Each intervention was evaluated independently by two reviewers for inclusion based on a set of pre-specified principles (Supplementary File S3). (C) Comparator: placebo, no treatment or standard care; single-arm interventional studies were also eligible. (D) Outcomes: outcomes related to maternal physical, psychological or emotional health, behavioural change affecting a mother's own health, or diagnostic accuracy of tests for women. Studies reporting economic outcomes only were not included, and neither were feasibility studies evaluating only retention rates and acceptability. (E) Types of studies: randomized trials, quasi-experimental studies and cohort studies, scoping reviews, systematic reviews, meta-analyses and overviews of systematic reviews. Systematic reviews and overviews must have stated some form of search strategy and presented all included studies in a systematic manner to be eligible for inclusion. Ongoing studies (defined as trials with protocols published from 2019 onwards which were not completed) were eligible; no restriction on date of publication was applied otherwise. Case series, case studies, case reports, dissertations and theses, reports published as abstracts only, correspondence letters, editorials, and qualitative studies were excluded. Only studies with full texts written in English were eligible.

For peer review only - http://bmjopen.bmj.com/site/about/guidelines.xhtml

Data collection and analysis

Two reviewers independently extracted data on characteristics of included studies, including study population, intervention, and outcomes used, using a standardised extraction form we developed to suit the review (Supplementary File S4). Study designs were classified as evidence syntheses, interventional studies, observational studies or diagnostic accuracy studies. Countries where studies took place were classified based on the World Bank 2021 country income levels [19]. For population characteristics, we recorded whether the studies included women under the age of 18, and whether those with high-risk pregnancies were included. For each study, the self-care activities that the intervention aimed to promote or facilitate, and the period of pregnancy during which the intervention took place (preconception, antenatal, intrapartum or postpartum period) were recorded. A set of self-care categories were created to organise all identified interventions, using an inductive approach. For interventional and observational studies, we recorded the modes of delivery/places of access to self-care based on WHO's framework [20]. We also recorded whether any external support person was required to promote or facilitate self-care. All interventions were matched with WHO's classification of self-care interventions [20]. Any disagreement was resolved via consensus or adjudication by a third reviewer (JPV). In line with scoping review methodology [12], quality assessment of included studies was not performed. Descriptive statistics (frequency and percentage) were used to report study, participant and intervention characteristics. Studies that feature more than three self-care interventions were excluded from final analysis. All analyses and graphs were generated from R v4.1.2.

RESULTS

A total of 12,215 records from six databases were retrieved, together with an additional 182 records from manual reference screening (Fig. 1). In total, 10,712 reports were excluded at the title/abstract

For peer review only - http://bmjopen.bmj.com/site/about/guidelines.xhtml

screening stage and another 743 reports at full text screening (Supplementary File S5). The final sample consisted of 580 unique studies (616 reports) (Supplementary File S6), 18 (3%) of which were ongoing studies.

Fig. 1. PRISMA flowchart of record retrieval and selection

Characteristics of included studies

Among 580 studies, two-thirds were interventional studies (n=365, 63%), a majority of which reported results of randomised trials (n=306, 53%). Evidence syntheses (n=165) accounted for 28% of all studies, a majority of which were meta-analyses (n=109, 19%). Observational studies were less common (n=32, 6%) and were mainly retrospective or prospective cohort studies (n=25, 4%). Study designs used were largely similar across all self-care categories, with some exceptions (Fig. 2A). For example, in self-management of medication, the proportion of non-randomised studies (n=7, 41%) was similar to that of randomised trials (n=6, 35%). In self-testing and self-collection of samples, many studies (n=10, 56%) were diagnostic accuracy studies.

Most studies took place in high-income countries (n=260, 45%), followed by middle-income (n=146, 25%) and low-income countries (n=11, 2%). Countries with the most studies were the United States of America (USA) (n=86, 15%), Iran (n=46, 8%), United Kingdom (UK) (n=33, 6%) and Australia (n=30, 5%). Self-care interventions most commonly took place in the antenatal (n=439, 76%) and postpartum periods (n=119, 21%). Few studies related to the preconception (n=21, 4%) or intrapartum periods (n=62, 11%) (Fig. 2B).

Fig. 2. Characteristics of included studies

Thirty-eight studies (7%) included women under the age of 18. A third of included studies (n=170, 29%) included women with high-risk pregnancies. High-risk pregnancies were variably defined – the most common definitions included pregnant women with gestational or existing diabetes (n=54, 9%), obesity or overweight (n=35, 6%) and ex-/current smokers (n=31, 5%).

For peer review only - http://bmjopen.bmj.com/site/about/guidelines.xhtml

Self-care interventions and their characteristics

Among included studies, 35 studies (6%) described a complex intervention involving more than three different self-care activities (Supplementary File S7). The remaining 545 studies (94%), which featured up to 3 self-care activities, constituted the final sample for identification of self-care interventions. From these 545 studies, a total of 112 unique self-care interventions were identified and organised into 11 broad categories (Fig. 3; Supplementary File S8). The most common self-care categories identified included diet and nutrition (n=143/545, 26%), physical activity (n=132, 24%), psycho-social strategies (n=97, 18%) and lifestyle adjustments (n=90, 17%). Few evidence was identified for sexual health and family planning (2%), self-management of medication (3%), and self-testing/sampling (3%). All identified interventions demonstrate various degrees of interaction with the health system [4], from little to no direct contact (self-awareness/regulation), some interaction (self-management of health conditions) and close linkage with the health system for follow-up (self-testing & diagnosis) (Fig. 3).

Fig. 3. All self-care interventions identified from included studies and their interactions with the health system

The identified interventions correspond to a range of categories from WHO's classification of self-care interventions (Supplementary File S9). The most common WHO categories were interventions for improving capacity and promoting autonomy in self-care (n=325, 56%), self-care prevention including risk avoidance and support for physical and mental health and well-being (n=296, 51%) and self-care for short-term health conditions (n=189, 33%). Some WHO categories did not feature amongst included studies, such as on-demand information services for health information, diagnostic devices in community locations, online symptom checkers for diagnosis, and interventions enabling individuals to identify location of health facilities/structures. Notably, we identified no studies that targeted self-care outside the context of a specific health issue, such as campaigns to promote awareness about self-care or programmes to improve health and digital literacy.

For peer review only - http://bmjopen.bmj.com/site/about/guidelines.xhtml

Among 545 studies under analysis, self-care was accessible through several avenues as described in the WHO classification framework [20]. These avenues include health facilities (n=238/545, 44%), digital platforms (n=97, 18%), community and non-facility venues (n=74, 14%), partner or peer support (n=39, 7%) or over-the-counter (OTC) products e.g. nutritional supplements, topical products, or support devices (n=69, 13%). In 80 studies (15%), there was insufficient detail to determine that the aforementioned modes of access were used, hence the intervention was assumed to take place at home. No studies took place at the workplace, in schools, or in emergency or humanitarian settings.

Across the self-care categories, provision of information and demonstration of self-care were most commonly done in health facilities, especially for self-testing (94% of studies in this category) and self-monitoring (58%). Provision of similar services outside health facilities, such as via community health workers or at community-based venues, was mainly for the support of self-management of medication (41%) and physical activity (28%). Home-based self-care (i.e. direct access to self-care resources and information at home) was less frequent, mainly in the categories of breast or nipple care (23%) and lifestyle adjustments (20%). Digital platforms were popular in some categories, notably self-monitoring (51%). Use of OTC products was common in complementary/alternative therapies (39%) and breast and nipple care (27%). Lastly, peer or partner support was relatively less common across categories (<20%), and it should be noted that partner support was relatively less frequent than support from peers (e.g. other women, members of support groups) (Fig. 4A).

A large number of studies described the involvement of health workers in promoting or facilitating self-care (n=208/545, 38%), especially for sexual health & family planning (67% of studies in this category), psychosocial strategies (45%) or physical activity (44%) (Fig. 4B). Few studies mentioned involvement of trained community health workers (n=41, 8%), non-trained community members (n=25, 5%) or family members (n=3, 1%).

Fig. 4. Characteristics of self-care interventions

For peer review only - http://bmjopen.bmj.com/site/about/guidelines.xhtml

Types of outcomes in self-care studies

Included studies used a diverse range of outcomes, including both maternal and neonatal outcomes (Table 1). The most commonly measured ones were neonatal outcomes (birth weight, Apgar scores, preterm birth, admission to neonatal intensive care unit and other neonatal complications) (n=177, 31%); maternal mental wellbeing and quality of life (n=150, 26%), and labour outcomes (duration of labour stages, labour pain, mode of delivery or labour induction methods) (n=125, 22%). A total of 147 studies (25%) measured behavioural outcomes, mainly related to reductions in risky behaviours such as smoking or alcohol consumption. Maternal mortality (n=20, 3%) and perinatal mortality (n=67, 12%) were less commonly evaluated.

For peer review only

For peer review only - http://bmjopen.bmj.com/site/about/guidelines.xhtml

Table 1. Evidence map for outcomes measured

Categories / Outcome	Maternal mortality	Perinatal mortality	Neonatal outcomes	Labour outcomes	Anthropometric & cardiometabolic outcomes	Diabetes & other metabolic disorders	Hypertensive disorders of pregnancy	Postpartum infection & haemorrhage	Urogenital outcomes	Nutritional deficiencies	Gastrointestinal outcomes	Other maternal complications	Infectious diseases	Other minor physiological discomforts	Mental wellbeing & quality of life	Behavioural change	Breastfeeding	Other
Diet & nutrition	5	29	71	27	53	47	34	4	9	11	12	14	3	3	21	42	7	0
Physical activity	0	6	48	33	58	45	22	7	5	1	1	9	0	13	40	50	7	0
Lifestyle adjustments	0	9	16	1	0	1	4	0	1	0	1	5	12	9	11	49	1	0
Complementary & alternative therapies	0	3	15	26	1	1	2	2	14	0	6	3	0	10	23	1	8	0
Psycho-social strategies	0	3	16	23	1	6	5	1	4	0	1	4	0	9	75	8	7	2
Breast & nipple care	0	3	6	4	0	2	1	1	0	0	0	0	0	16	1	2	17	1
Sexual health & family planning	0	0	1	0	0	0	0	0	0	0	0	1	1	0	7	9	0	0
Self-monitoring	3	18	39	28	23	27	24	3	0	0	0	8	0	0	15	17	1	1
Self-management of medication	6	4	7	0	1	0	1	5	0	0	0	4	4	0	1	12	0	0
Self-testing or sampling	1	2	4	1	1	0	1	0	0	0	0	0	1	0	0	5	0	1
Other self-management skills	5	5	16	27	0	1	5	17	3	0	0	10	0	1	17	4	1	3
**All self-care categories**	**20 3%**	**67 12%**	**177 31%**	**125 22%**	**78 13%**	**79 14%**	**65 11%**	**33 6%**	**32 6%**	**11 2%**	**17 3%**	**43 7%**	**20 3%**	**46 8%**	**150 26%**	**147 25%**	**34 6%**	**8 1%**

Note: Number in each cell denotes the number of studies featuring the corresponding self-care category (row) and measuring the corresponding outcome (column). Percentages are based on total studies (N=580). For further details on the definition of each outcome category, see Supplementary File S4.

For peer review only - http://bmjopen.bmj.com/site/about/guidelines.xhtml

DISCUSSION

Main findings

In this scoping review, 545 studies were identified pertaining to 112 self-care interventions across preconception, antenatal, intrapartum and postpartum periods. These interventions encompassed multiple modalities, settings and levels of provider involvement. The majority of self-care interventions required close engagement with health facilities, in which healthcare workers or community health workers would teach self-care skills or provide therapeutics for home use. As such, self-care should be viewed as an extension of the health system and not as a separate entity from formal healthcare services. Primary healthcare services are a critical starting point for successful self-care approaches [21–23]. Our findings also reflect a broader trend towards task-sharing in order to increase maternity care coverage [7,24]. While the majority of self-care interventions were implemented in the antenatal period, many of these studies aimed to equip pregnant individuals with self-care skill via workshops or counselling, which can be used in the intrapartum (e.g. relaxation methods during labour) or postpartum periods (e.g. pelvic floor exercises, breast and nipple care). Few interventions featured digital technologies; those identified were mostly used for communication between the pregnant individual and a remote health worker, or as a platform for the pregnant individual to record her medical information and receive automatic, individualised health advice. While digital technologies seem to currently play a limited role, they have great potential to catalyse and promote maternity-related self-care [25].

Most studies concentrated on self-care interventions related to lifestyle modification - such as diet, nutrition/supplement intake, physical activity and sleep habits - as well as strategies to optimise mental and social well-being. This likely reflects a more well-established evidence base for lifestyle modification interventions, generally associated with a lower risk of harm. Conversely, there were relatively few studies that explored self-monitoring, self-testing or self-management of medications

For peer review only - http://bmjopen.bmj.com/site/about/guidelines.xhtml

or pregnancy complications. While studies of these self-care approaches can be complex or technically challenging, they could provide solutions to improving care in remote and low-resource settings [6]. While advanced distribution and self-administration of misoprostol for postpartum haemorrhage prevention has been well-explored [26–28], there was limited or no evidence on similar approaches to other therapeutics, such as use of anthelminthics, malaria prophylaxis, antimalarials, antiretroviral therapy, or vitamin and micronutrient supplementation. Although many studies reported on the prevalence and determinants of self-medication during pregnancy [29–31], none investigated interventions aimed at promoting safe self-medication practices, or how to seek information when self-medicating. There were also few self-care interventions aimed at communicable disease detection and prevention during or after pregnancy. Future research is warranted to investigate how these aspects of clinical care during and after pregnancy could be approached from a self-care perspective.

We identified studies describing self-testing options for urinary tract infections and HIV, but few or no studies on malaria, syphilis or chlamydia self-testing, for which rapid test technologies are available but not yet scaled up or tested in pregnancy [32–34]. Similarly, self-collection of vaginal samples for reproductive tract infections is an emerging alternative to clinic-based sampling [35]. While some studies have investigated diagnostic accuracy of self-sampling for reproductive tract infections in pregnancy, evidence on their benefits, harms, and effective linkages with health systems is currently lacking [36]. Lastly, we identified self-care studies on insecticide-treated nets for malaria, mostly from the sub-Saharan Africa region. However there was little evidence for other low-cost strategies amenable to a self-care approach (indoor residual spraying and insect repellents), or from other malaria-endemic regions such as Asia and Latin America [37].

Strengths and limitations

This is the first comprehensive scoping review on self-care for preconception, antenatal, intrapartum and postpartum care. Strengths of this review include broad searches of multiple self-care concepts

For peer review only - http://bmjopen.bmj.com/site/about/guidelines.xhtml

and databases, and a systematic searching and screening process, following methods that were pre-specified in a registered protocol. We analysed studies based on multiple characteristics, as well as applying the new WHO self-care classification system to organise, categorise and facilitate interpretation of findings.

This scoping review was intended to present an overview of the research landscape, and not to critically appraise or meta-analyse available evidence. The quantity of evidence for a given self-care intervention may not correlate with evidence quality, or its effectiveness. Some limitations must nonetheless be acknowledged. We opted to focus on maternity-related self-care, thus excluding interventions that took place after 42 days postpartum, or those that related primarily to care of newborns and breastfeeding. These exclusions do not discount their importance, but rather acknowledge that these topics require a separate, dedicated review. Our search of published reports means that self-care interventions that have only been evaluated in unpublished studies or grey literature would not have been identified. With 45 reports excluded as not written in English (3.3% of all full-text reports), language bias is possible.

For peer review only

CONCLUSION

Self-care interventions during pre-conception, antepartum, intrapartum and postpartum periods are diverse in scope and implementation characteristics. Many of these self-care interventions require formal support and engagement with the health system. Future research is warranted on self-care interventions that explore task-sharing from clinical care to home or community settings, or effective self-management of complications where access to care is unavailable.

List of abbreviations

WHO: World Health Organization

OTC: over-the-counter

For peer review only - http://bmjopen.bmj.com/site/about/guidelines.xhtml

DECLARATIONS

Ethics approval and consent to participate: Not applicable

Consent for publication: Not applicable

Availability of data and materials: Data supporting the findings and conclusion of this study are included in this published article and its supplementary files. The underlying dataset and analytic code are available at osf.io/26f7y.

Competing interests: None declared.

Funding: This project was funded by UNDP/UNFPA/UNICEF/WHO/World Bank Special Programme of Research, Development and Research Training in Human Reproduction (HRP), Department of Sexual and Reproductive Health and Research, World Health Organization (# PO 202724071). While members of our authorship team (MN, MB, OT) are employed by HRP/WHO, the funder organization had no role in the design and conduct of the study; collection, management, analysis, and interpretation of the data; preparation, review, or approval of the manuscript; and decision to submit the manuscript for publication.

Acknowledgement: We thank Steve McDonald (Cochrane Australia) for his guidance in formulating the search strategy.

Patient and Public Involvement Statement: Members of our authorship team provided perspectives of health policy makers (MN, MB, OT), as well as clinicians and midwives (AW, KB, JPV) during the conceptualisation and conduct of this review. As this was a scoping review, it was not appropriate to involve patients or the public in the design, or conduct, or reporting, or dissemination plans of our research.

For peer review only - http://bmjopen.bmj.com/site/about/guidelines.xhtml

Author contributions

The corresponding author attests that all listed authors meet authorship criteria and that no others meeting the criteria have been omitted.

P-YN: Data Curation, Formal Analysis, Investigation, Methodology, Writing – Original Draft Preparation

CC: Investigation, Writing – Review & Editing

MJP: Methodology, Writing – Review & Editing

AW, KB, AMG, MN, MB, OT: Validation, Writing – Review & Editing

JPV: Conceptualisation, Funding Acquisition, Supervision, Validation, Writing – Review & Editing

Supplementary Files

Supplementary File S1: Protocol as registered on Open Science Framework

Supplementary File S2: Search strategy for all databases

Supplementary File S3: Defining self-care interventions, a conceptual framework and examples for making judgements on specific interventions

Supplementary File S4: Data collection form

Supplementary File S5: List of excluded full texts

Supplementary File S6: Reference list of included studies

Supplementary File S7: Complex interventions and their characteristics

Supplementary File S8: List of all self-care interventions identified from included studies

Supplementary File S9: Categorising self-care interventions based on WHO's classification framework

Supplementary File S10: PRISMA-ScR checklist

For peer review only - http://bmjopen.bmj.com/site/about/guidelines.xhtml

For peer review only

For peer review only - http://bmjopen.bmj.com/site/about/guidelines.xhtml

References

1 World Health Organization. Self-care interventions for health. Fact sheet. 2021.https://www.who.int/news-room/fact-sheets/detail/self-care-health-interventions (accessed 29 May 2021).

2 International Self-Care Foundation. The Seven Pillars of Self-Care. https://isfglobal.org/practise-self-care/the-seven-pillars-of-self-care/ (accessed 13 Jan 2022).

3 World Health Organization. Guideline on Self-Care Interventions for Health and Well-Being. Geneva: 2021. https://www.who.int/publications/i/item/9789240030909

4 Narasimhan M, Allotey P, Hardon A. Self care interventions to advance health and wellbeing: A conceptual framework to inform normative guidance. *BMJ* 2019;**365**. doi:10.1136/bmj.l688

5 Narasimhan M, Logie CH, Gauntley A, *et al.* Self-care interventions for sexual and reproductive health and rights for advancing universal health coverage. *Sex Reprod Heal matters* 2020;**28**. doi:10.1080/26410397.2020.1778610

6 Pavlidis P, Eddy K, Phung L, *et al.* Clinical guidelines for caring for women with COVID-19 during pregnancy, childbirth and the immediate postpartum period. Women and Birth. 2020. doi:10.1016/j.wombi.2020.10.015

7 Gülmezoglu AM, Ammerdorffer A, Narasimhan M, *et al.* Self-care and remote care during pregnancy: A new paradigm? *Heal Res Policy Syst* 2020;**18**:1–3. doi:10.1186/S12961-020-00627-4

8 World Health Organization. Maintaining essential health services: operational guidance for the COVID-19 context, interim guidance. 2020.https://www.who.int/publications/i/item/WHO-2019-nCoV-essential_health_services-2020.2 (accessed 21 Jan 2022).

9 Rasouli M, Pourheidari M, Gardesh Z. Effect of Self-care Before and During Pregnancy to Prevention and Control Preeclampsia in High-risk Women. *Int J Prev Med* 2019;**10**. doi:10.4103/IJPVM.IJPVM_300_17

10 Dalrymple KV, Flynn AC, Poston L, *et al.* Preventing postpartum weight retention: Lifestyle interventions in pregnancy, postpartum or both. A systematic review of the literature. *Proc Nutr Soc* 2018;**77**:77. doi:10.1017/S002966511800191X

11 Kolivand M, Rahimi MA, Shariati M, *et al.* The Effect of Self-care Educational/Training Interventions on the Outcomes of Gestational Diabetes: A Review Article. *Iran J Public Health* 2018;**47**:1805.https://www.ncbi.nlm.nih.gov/pmc/articles/PMC6379603/ (accessed 27 Apr 2022).

12 Peters MDJ, Godfrey CM, Khalil H, *et al.* Guidance for conducting systematic scoping reviews. *Int J Evid Based Healthc* 2015;**13**:141–6. doi:10.1097/XEB.0000000000000050

13 Tricco AC, Lillie E, Zarin W, *et al.* PRISMA extension for scoping reviews (PRISMA-ScR): Checklist and explanation. Ann. Intern. Med. 2018;**169**:467–73. doi:10.7326/M18-0850

14 Abalos E, Chamillard M, Diaz V, *et al.* Antenatal care for healthy pregnant women: a mapping of interventions from existing guidelines to inform the development of new WHO guidance on antenatal care. *BJOG* 2016;**123**:519–28. doi:10.1111/1471-0528.13820

15 Donald M, Kahlon BK, Beanlands H, *et al.* Self-management interventions for adults with chronic kidney disease: a scoping review. *BMJ Open* 2018;**8**:e019814.

For peer review only - http://bmjopen.bmj.com/site/about/guidelines.xhtml

doi:10.1136/BMJOPEN-2017-019814

16 Foster G, Taylor SJC, Eldridge SE, *et al.* Self-management education programmes by lay leaders for people with chronic conditions. *Cochrane Database Syst Rev* 2007.

17 Benzies KM, Premji S, Hayden KA, *et al.* State-of-the-Evidence Reviews: Advantages and Challenges of Including Grey Literature. *Worldviews Evidence-Based Nurs* 2006;**3**:55–61. doi:10.1111/J.1741-6787.2006.00051.X

18 Veritas Health Innovation. Covidence Systematic Review Software, Melbourne Australia. 2020.www.covidence.org (accessed 22 Jul 2021).

19 World Bank. The World by Income and Region. 2021.https://datatopics.worldbank.org/world-development-indicators/the-world-by-income-and-region.html (accessed 20 Jan 2022).

20 World Health Organization. Classification of self-care interventions for health: a shared language to describe the uses of self-care interventions. Geneva: 2021. https://www.who.int/publications/i/item/9789240039469 (accessed 6 Apr 2022).

21 Bell J, Dziekan G, Pollack C, *et al.* Self-Care in the Twenty First Century: A Vital Role for the Pharmacist. *Adv Ther* 2016;**33**:1691–703. doi:10.1007/S12325-016-0395-5

22 Davies NJ. Improving self-management for patients with long-term conditions. *Nurs Stand* 2010;**24**. doi:10.7748/NS2010.02.24.25.49.C7562

23 Lorig KR, Holman HR. Self-Management Education: History, Definition, Outcomes, and Mechanisms. *Ann Behav Med* 2003;**26**:1–7.https://academic.oup.com/abm/article/26/1/1/4630312 (accessed 13 Jan 2022).

24 Dawson AJ, Buchan J, Duffield C, *et al.* Task shifting and sharing in maternal and reproductive health in low-income countries: a narrative synthesis of current evidence. *Health Policy Plan* 2014;**29**:396–408. doi:10.1093/HEAPOL/CZT026

25 DeNicola N, Grossman D, Marko K, *et al.* Telehealth Interventions to Improve Obstetric and Gynecologic Health Outcomes: A Systematic Review. *Obstet Gynecol* 2020;**135**:371–82. doi:10.1097/AOG.0000000000003646

26 Oladapo OT, Blum J, Abalos E, *et al.* Advance misoprostol distribution to pregnant women for preventing and treating postpartum haemorrhage. *Cochrane Database Syst Rev* 2020;**2020**. doi:10.1002/14651858.CD009336.PUB3

27 Onongé S, Campbell OMR, Kaharuza F, *et al.* Effectiveness and safety of misoprostol distributed to antenatal women to prevent postpartum haemorrhage after child-births: A stepped-wedge cluster-randomized trial. *BMC Pregnancy Childbirth* 2015;**15**. doi:10.1186/S12884-015-0750-6

28 Weeks AD, Ditai J, Ononge S, *et al.* The MamaMiso study of self-administered misoprostol to prevent bleeding after childbirth in rural Uganda: A community-based, placebo-controlled randomised trial. *BMC Pregnancy Childbirth* 2015;**15**:1–10. doi:10.1186/S12884-015-0650-9

29 Atmadani RN, Nkoka O, Yunita SL, *et al.* Self-medication and knowledge among pregnant women attending primary healthcare services in Malang, Indonesia: A cross-sectional study. *BMC Pregnancy Childbirth* 2020;**20**:1–11. doi:10.1186/S12884-020-2736-2

30 Niriayo YL, Mohammed K, Asgedom SW, *et al.* Self-medication practice and contributing factors among pregnant women. *PLoS One* 2021;**16**:e0251725. doi:10.1371/JOURNAL.PONE.0251725

For peer review only - http://bmjopen.bmj.com/site/about/guidelines.xhtml

31 Mohseni M, Azami-Aghdash S, Sheyklo SG, *et al.* Prevalence and reasons of self-medication in pregnant women: A systematic review and meta-analysis. Int. J. Community Based Nurs. Midwifery. 2018;**6**.

32 Berthod D, Rochat J, Voumard R, *et al.* Self-diagnosis of malaria by travellers: A cohort study on the use of malaria rapid diagnostic tests provided by a Swiss travel clinic. *Malar J* 2017;**16**:1–8. doi:10.1186/S12936-017-2079-2

33 Wang C, Cheng W, Li C, *et al.* Syphilis Self-testing: A Nationwide Pragmatic Study Among Men Who Have Sex With Men in China. *Clin Infect Dis* 2020;**70**:2178–86. doi:10.1093/CID/CIZ603

34 Sacks-Davis R, Gold J, Aitken CK, *et al.* Home-based chlamydia testing of young people attending a music festival - Who will pee and post? *BMC Public Health* 2010;**10**:1–9. doi:10.1186/1471-2458-10-376

35 Fajardo-Bernal L, Aponte-Gonzalez J, Vigil P, *et al.* Home-based versus clinic-based specimen collection in the management of Chlamydia trachomatis and Neisseria gonorrhoeae infections. *Cochrane Database Syst Rev* 2015;**2015**. doi:10.1002/14651858.CD011317.PUB2

36 Siegfried N, Narasimhan M, Logie CH, *et al.* Prioritising gender, equity, and human rights in a GRADE-based framework to inform future research on self care for sexual and reproductive health and rights. *BMJ Glob Heal* 2020;**5**:2128. doi:10.1136/BMJGH-2019-002128

37 Menéndez C, D'Alessandro U, ter Kuile FO. Reducing the burden of malaria in pregnancy by preventive strategies. *Lancet Infect Dis* 2007;**7**:126–35. doi:10.1016/S1473-3099(07)70024-5

For peer review only

For peer review only - http://bmjopen.bmj.com/site/about/guidelines.xhtml

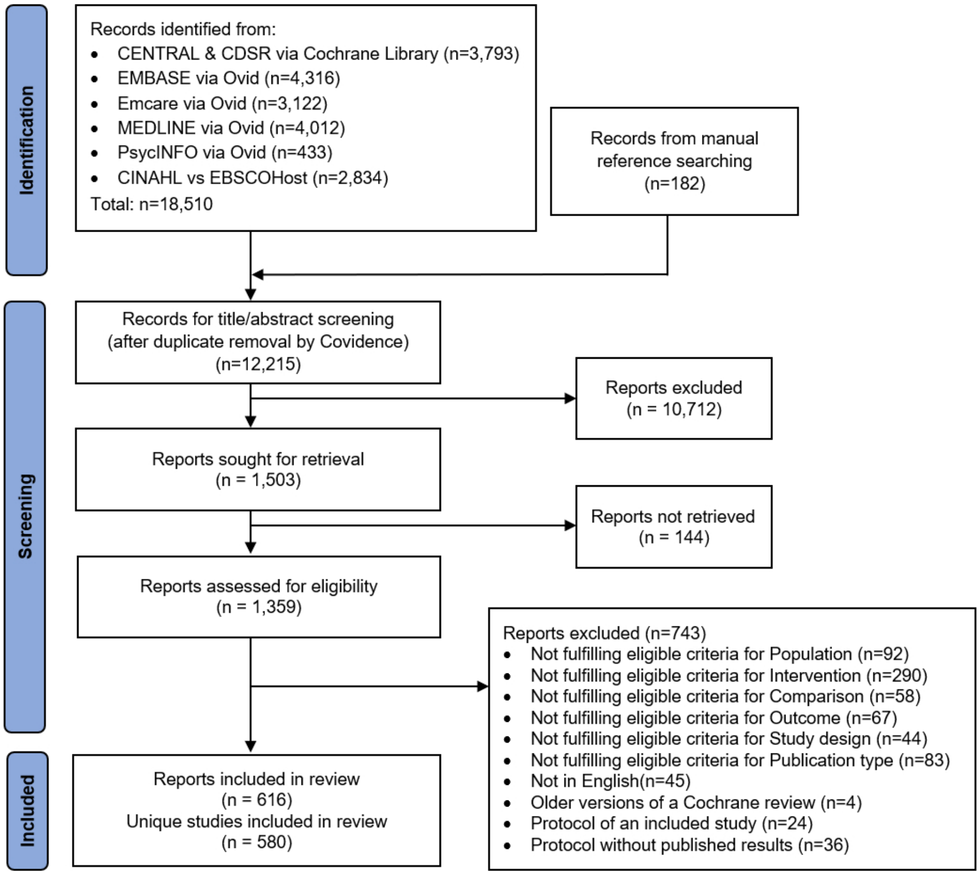

Fig. 1. PRISMA flowchart of record retrieval and selection

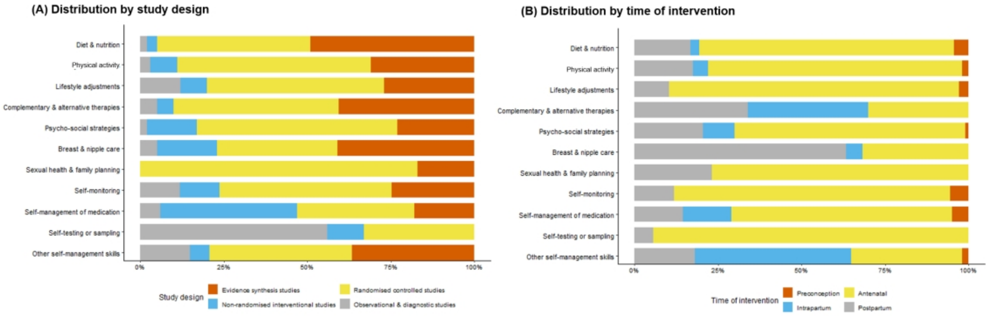

Fig. 2. Characteristics of included studies

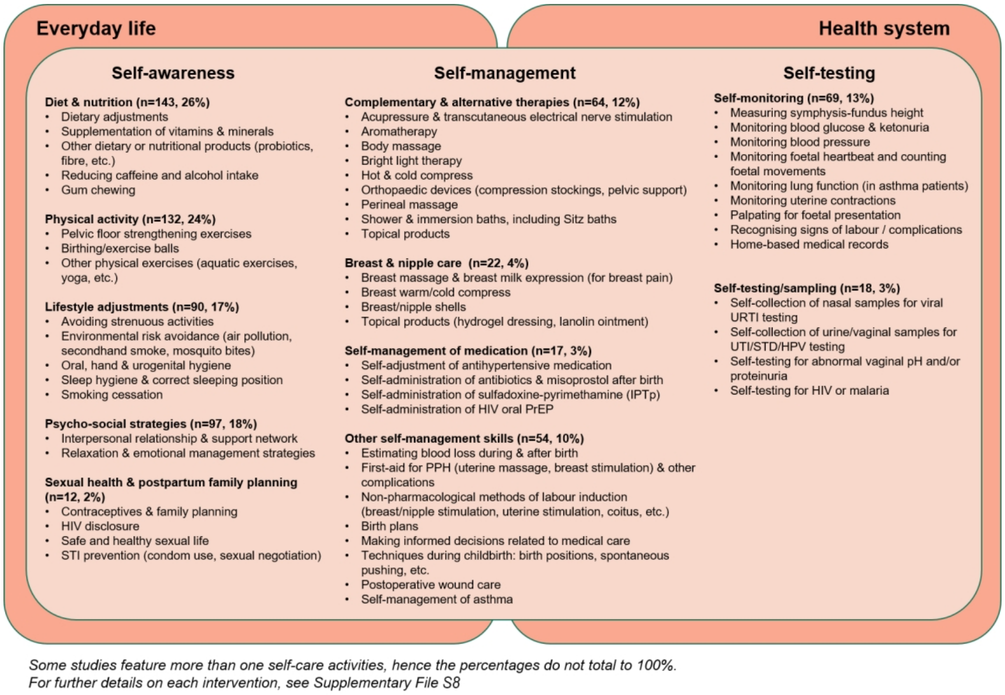

Fig. 3. All self-care interventions identified from included studies and their interactions with the health system

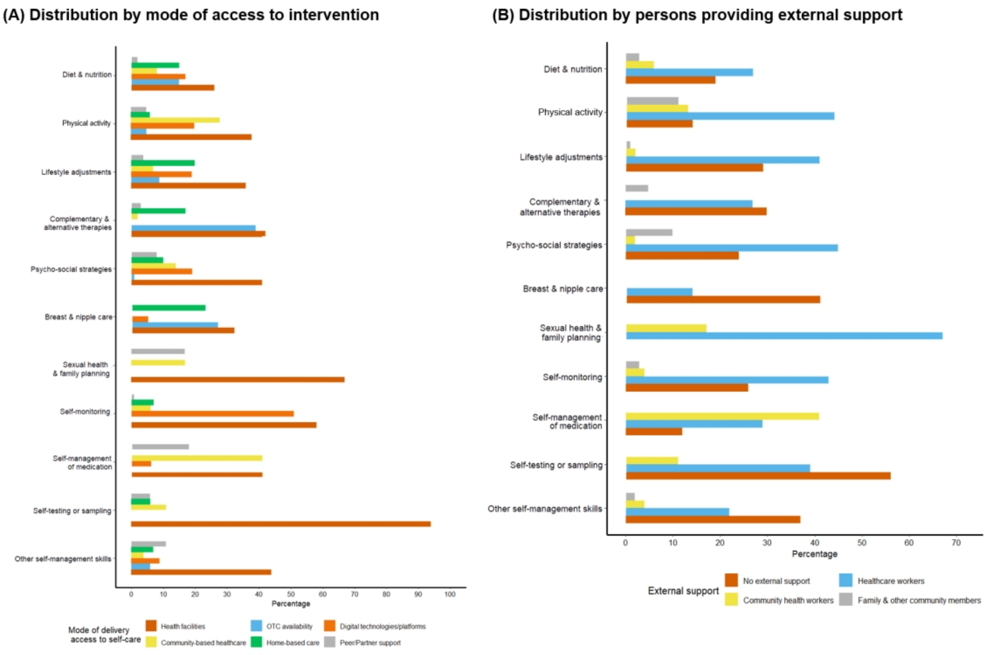

Fig. 4. Characteristics of self-care interventions

Title page

Supplementary File S1: Protocol as registered on Open Science Framework 2

Supplementary File S2: Search strategy for all databases 6

Supplementary File S3: Defining self-care interventions, a conceptual framework and examples for making judgements on specific interventions 28

Supplementary File S4: Data collection form.. 32

Supplementary File S5: List of excluded full texts 37

Supplementary File S6: Reference list of included studies 62

Supplementary File S7: Complex interventions and their characteristics...................................... 94

Supplementary File S8: List of all self-care interventions identified from included studies 96

Supplementary File S9: Categorising self-care interventions based on WHO's classification framework 101

For peer review only

Supplementary File S1 | Protocol as registered on Open Science Framework

Title: Self-care interventions for antenatal, intrapartum and postnatal care: protocol for a scoping review

1. INTRODUCTION

Self-care is defined by the World Health Organization (WHO) as “the ability of individuals, families and communities to promote health, prevent disease, maintain health, and to cope with illness and disability with or without the support of a healthcare provider” (WHO, 2021). Self-care interventions are tools that support self-care, which include “evidence-based, high-quality drugs, devices, diagnostics and/or digital interventions that can be provided fully or partially outside formal health services and be used with or without a health worker” (WHO, 2021). Self-care interventions offer a solution to reduce health disparities and inequity in health access, especially in low-resource settings. By co-opting the pregnant woman and her family, self-care interventions can enhance the overall efficiency of healthcare delivery, while also improving service uptake and adherence to treatment.

There is a large and heterogeneous body of research on maternal and perinatal health interventions that are useful (or promising) from a self-care perspective. In this context, a scoping review can help identify established or emerging self-care interventions that could be considered for developing recommendations.

2. OBJECTIVES

- To identify self-care interventions in antenatal, intrapartum and postnatal care with sufficient evidence that may warrant a WHO recommendation
- To identify emerging self-care interventions that are promising research areas and should be prioritised
- To describe and classify these interventions by self-care concepts, behaviours, mode of delivery and other characteristics

3. METHODS

This protocol was adapted from the methodological guide for scoping reviews (Peters et al., 2015). The review will be reported using the PRISMA for Scoping Reviews extension (Tricco et al., 2018).

3.1. Electronic searches

We will systematically search Medline, Embase, Emcare and PsycINFO (via Ovid), CENTRAL and CDSR (via Cochrane Library) and CINAHL Plus (via EBSCOhost) to identify published studies from inception to current. We have developed a search strategy which combines various self-care concepts with terms related to pregnancy care, based on the framework proposed by Narasimhan et al. (2019). The terms and concepts used were informed by previous reviews of pregnancy care and self-care for other health conditions (e.g. chronic diseases). References of included articles will be manually screened to identify studies.

Table 1. Categories of self-care interventions

Self-management	Self-testing	Self-awareness

For peer review only - http://bmjopen.bmj.com/site/about/guidelines.xhtml

Self-medication	Self-sampling	Self-help
Self-treatment	Self-screening	Self-education
Self-examination	Self-diagnosis	Self-regulation
Self-injection	Self-collection	Self-efficacy
Self-use	Self-monitoring	Self-determination

2. Study selection

Two reviewers will independently screen titles and abstracts to remove irrelevant studies and then review full texts based on eligible criteria below. All stages of screening will be conducted in Covidence (Veritas Health Innovation, Australia). Any disagreement is resolved by discussion or adjudication to a third reviewer.

The eligibility criteria for inclusion are:

3.1 Participants: Women preparing to get pregnant, pregnant women, women giving birth, or women in the postnatal period (up to 42 days after birth), of any age. Studies examining interventions only directed at newborns will be excluded.

3.2 Interventions

For the purpose of this review, a self-care intervention is defined as any tool, resource or strategy designed to promote or facilitate self-care practices by the pregnant woman for the purpose of improving the quality or coverage of maternity health care, and/or improving the health, well-being and experiences of the woman and her baby.

A self-care practice include those that can be taken by a pregnant woman of her own accord towards the goals of (a) information-seeking and improving health literacy, (b) promoting mental health, self-efficacy and self-awareness, (c) increasing physical activity, (d) maintaining a healthy diet, (e) reducing risk and exposure avoidance, (d) good hygiene, and (7) safe and appropriate use of medical products and services.

This definition framework is summarised in Figure 1.

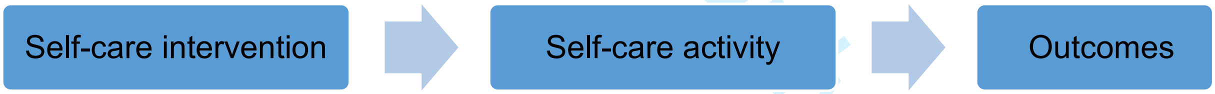

Annex 2 further elaborates on this conceptual framework and outlines the principles that guide the reviewers in judging whether an intervention is considered a self-care intervention.

3.3 Comparator: No intervention, placebo or standard/clinical care; single-arm interventional studies are eligible.

3.4 Outcomes: These interventions must be aimed at reducing risks of any pregnancy, childbirth or postnatal complications and/or enabling a positive pregnancy, childbirth or postnatal experience. The outcomes can be relating to physical, psychological or emotional health, behavioural change or product performance. Interventions measuring social, economic and financial outcomes are excluded from the scope of this review. Feasibility studies evaluating only retention rates and acceptability are excluded.

3.5 Types of studies:

We will include RCTs, quasi-experimental studies and cohort studies, as well as systematic reviews and meta-analyses for mapping. Reviews must state some forms of search strategy

For peer review only

and present all included studies in a systematic manner to be eligible. No date restriction will be applied. Case series, case studies, case reports, dissertations and theses, reports published as abstracts only, correspondence letters and editorials, qualitative studies will be excluded. Only studies with full texts written in English are eligible.

Quality assessment

In line with scoping review methodology (Peters et al., 2015), quality assessment will not be performed.

Data extraction and analysis

We will collect the following data on study characteristics (study design, country of origin, publication year), population characteristics (generic vs condition-specific, age, individual vs group, cultural/socioeconomic disadvantaged groups), intervention characteristics (provider, location, mode of delivery, period of pregnancy, routine vs condition-specific care, basic description), type of outcomes (physical, psychological or emotional). Interventions will be organised into themes, which are adapted from an earlier WHO's guideline on antenatal care (WHO, 2016) and will be further modified based on an iterative process of analysing retrieved interventions. The current proposed themes are as follows: (1) lifestyle adjustments, including nutritional interventions and physical activities; (2) interventions for emotional and spiritual wellbeing; (3) self-monitoring and assessment to aid decision-making; (4) preventive measures against pregnancy complications; and (5) interventions to alleviate common physiological symptoms.

Two reviewers will perform data extraction independently using a predefined template. Any disagreement is resolved via discussion or adjudication to a third reviewer. Given the rapid and scoping nature of this review, authors will not be contacted for missing data.

Results will be summarised as an evidence map accompanied by a narrative summary. Based on a 2D-intervention-outcome framework (Saran et al, 2018), the evidence map will visually display the type and quantity of evidence for each intervention, classified by outcome type, type of evidence, findings of evidence and sample size. The narrative summary will discuss the scope and strength of the evidence base. We will focus on identifying self-care interventions with high-quality evidence to be taken to scale, interventions that require further evidence synthesis with systematic reviews, and interventions that lack primary evidence.

References

Narasimhan, M., Allotey, P., & Hardon, A. (2019). Self care interventions to advance health and wellbeing: A conceptual framework to inform normative guidance. *BMJ (Online)*, *365*. https://doi.org/10.1136/bmj.l688

Peters, M. D. J., Godfrey, C. M., Khalil, H., McInerney, P., Parker, D., & Soares, C. B. (2015). Guidance for conducting systematic scoping reviews. *International Journal of Evidence-Based Healthcare*, *13*(3), 141–146. https://doi.org/10.1097/XEB.0000000000000050

Saran A, White H. Evidence and gap maps: a comparison of different approaches. Campbell Systematic Reviews. 2018 Jan;14(1):1-38.

Tricco, A. C., Lillie, E., Zarin, W., O'Brien, K. K., Colquhoun, H., Levac, D., Moher, D., Peters, M. D. J., Horsley, T., Weeks, L., Hempel, S., Akl, E. A., Chang, C., McGowan, J., Stewart, L., Hartling, L., Aldcroft, A., Wilson, M. G., Garritty, C., … Straus, S. E. (2018). PRISMA extension for scoping reviews (PRISMA-ScR): Checklist and explanation. In *Annals of Internal Medicine* (Vol. 169, Issue 7, pp. 467–473). American College of Physicians. https://doi.org/10.7326/M18-0850

For peer review only

WHO. (2016). *Recommendations on antenatal care for a positive pregnancy experience*. World Health Organization. https://www.who.int/publications-detail-redirect/9789241549912

WHO. (2019). *What do we mean by self-care?* World Health Organization. http://www.who.int/reproductivehealth/self-care-interventions/definitions/en/

WHO. (2021). *Guideline on Self-Care Interventions for Health and Well-Being*. World Health Organization. https://www.who.int/publications-detail-redirect/9789240030909

For peer review only

Supplementary File S2 | Search strategy for all databases

Cochrane Library <October 17, 2021>

#1	(self next care):ti,ab,kw
#2	(selfcare):ti,ab,kw
#3	MeSH descriptor: [Self Care] explode all trees
#4	((home or "home-based" or homebased) NEAR/3 care):ti,ab,kw
#5	(self near/1 manag*):ti,ab,kw
#6	(selfmanag*):ti,ab,kw
#7	MeSH descriptor: [Self-Management] explode all trees
#8	((home or "home-based" or homebased) near/3 manag*):ti,ab,kw
#9	(self NEXT medicat*):ti,ab,kw
#10	(selfmedicat*):ti,ab,kw
#11	MeSH descriptor: [Self Medication] explode all trees
#12	((home or "home-based" or homebased) near/3 (medicat* or medicine)):ti,ab,kw
#13	(self NEXT treat*):ti,ab,kw
#14	(selftreat*):ti,ab,kw
#15	((home or "home-based" or homebased) near/3 treat*):ti,ab,kw
#16	(self NEXT examin*):ti,ab,kw
#17	(selfexamin*):ti,ab,kw
#18	MeSH descriptor: [Self-Examination] explode all trees
#19	((home or "home-based" or homebased) near/3 exam*):ti,ab,kw
#20	(self NEXT inject*):ti,ab,kw
#21	(selfinject*):ti,ab,kw
#22	((home or "home-based" or homebased) near/3 inject*):ti,ab,kw
#23	(self NEXT administ*):ti,ab,kw
#24	(selfadminist*):ti,ab,kw
#25	MeSH descriptor: [Self Administration] explode all trees
#26	((home or "home-based" or homebased) near/3 (administ* not administrator*)):ti,ab,kw
#27	(self NEXT (use or usage)):ti,ab,kw
#28	(selfuse or selfusage):ti,ab,kw
#29	(self NEXT test*):ti,ab,kw
#30	(selftest*):ti,ab,kw
#31	MeSH descriptor: [Self-Testing] explode all trees
#32	((home or "home-based" or homebased) near/3 (test* or kits or kit)):ti,ab,kw
#33	(self NEXT sampl*):ti,ab,kw
#34	(selfsampl*):ti,ab,kw
#35	((home or "home-based" or homebased) near/3 sampl*):ti,ab,kw
#36	(self NEXT screen*):ti,ab,kw
#37	(selfscreen*):ti,ab,kw
#38	((home or "home-based" or homebased) near/3 screen*):ti,ab,kw
#39	(self NEXT diagnos*):ti,ab,kw
#40	(selfdiagnos*):ti,ab,kw
#41	((home or "home-based" or homebased) near/3 diagnos*):ti,ab,kw
#42	(self NEXT collect*):ti,ab,kw
#43	(selfcollect*):ti,ab,kw
#44	((home or "home-based" or homebased) near/3 collect*):ti,ab,kw
#45	(self NEXT monitor*):ti,ab,kw
#46	(selfmonitor*):ti,ab,kw
#47	((home or "home-based" or homebased) near/3 monitor*):ti,ab,kw
#48	MeSH descriptor: [Blood Glucose Self-Monitoring] explode all trees
#49	(SMBG):ti,ab,kw

#50	(self NEXT measur*):ti,ab,kw
#51	(selfmeasur*):ti,ab,kw
#52	((home or "home-based" or homebased) near/3 measur*):ti,ab,kw
#53	(self NEXT assess*):ti,ab,kw
#54	(selfassess*):ti,ab,kw
#55	MeSH descriptor: [Self-Assessment] explode all trees
#56	((home or "home-based" or homebased) near/3 assess*):ti,ab,kw
#57	(self NEXT evaluat*):ti,ab,kw
#58	(selfevaluat*):ti,ab,kw
#59	MeSH descriptor: [Diagnostic Self Evaluation] explode all trees
#60	((home or "home-based" or homebased) near/3 evaluat*):ti,ab,kw
#61	(self NEXT aware*):ti,ab,kw
#62	(selfaware*):ti,ab,kw
#63	(self NEXT help*):ti,ab,kw
#64	(selfhelp*):ti,ab,kw
#65	MeSH descriptor: [Self-Help Devices] explode all trees
#66	(self NEXT educat*):ti,ab,kw
#67	(selfeducat*):ti,ab,kw
#68	(self NEXT regulat*):ti,ab,kw
#69	(selfregulat*):ti,ab,kw
#70	MeSH descriptor: [Self-Control] explode all trees
#71	((home or "home-based" or homebased) near/3 control):ti,ab,kw
#72	(self NEXT efficac*):ti,ab,kw
#73	(selfeffica*):ti,ab,kw
#74	MeSH descriptor: [Self Efficacy] explode all trees
#75	(self NEXT determin*):ti,ab,kw
#76	(selfdetermin*):ti,ab,kw
#77	MeSH descriptor: [Personal Autonomy] explode all trees
#78	MeSH descriptor: [Self Disclosure] explode all trees
#79	(self NEXT advoca*):ti,ab,kw
#80	(selfadvoca*):ti,ab,kw
#81	(self NEXT reliance):ti,ab,kw
#82	(selfreliance):ti,ab,kw
#83	(self-adjust* NEAR/3 (medication* or drug* or dosage* or doses or dosing)):ti,ab,kw
#84	(person NEXT cent* NEAR/3 care):ti,ab,kw
#85	(patient NEXT orient* NEAR/3 care):ti,ab,kw
#86	(patient NEXT cent* NEAR/3 care):ti,ab,kw
#87	MeSH descriptor: [Patient-Centered Care] explode all trees
#88	(family cent* NEAR/3 care):ti,ab,kw
#89	("non-pharmacological" or "non-pharmaceutical" or "non-clinical" or "non-medical"):ti,ab,kw
#90	((prevention or preventive) near/3 (infectious or infection*)):ti,kw
#91	(yeast or candida):ti,kw and (prevent* or reduc* or test* or diagnos*):ti,kw
#92	(postpartum NEAR/1 (anemia or anaemia)):ti,kw
#93	(hpv or papillo*):ti,kw and (prevent* or reduc*):ti,kw
#94	(cmv or cytomegalo*):ti,kw and (prevent* or reduc*):ti,kw
#95	(torch and prevent*):ti,kw
#96	(zika and (prevent* or reduc* or practices or strategies)):ti,kw
#97	(malaria:ti) and (prevent* or reduc* or practices or strategies):ti,kw
#98	((mosquito* or "vector-borne") and (prevent* or reduc* or practices or strategies)):ti,kw
#99	(sti or stis or std or stds or "sexually-transmitted"):ti,kw and (prevention or reduc* or practices or strategies):ti,kw
#100	#1 OR #2 OR #3 OR #4 OR #5 OR #6 OR #7 OR #8 OR #9 OR #10 OR #11 OR #12 OR #13 OR #14 OR #15 OR #16 OR #17 OR #18 OR #19 OR #20 OR #21 OR #22 OR #23 OR #24 OR #25 OR #26 OR #27

	OR #28 OR #29 OR #30 OR #31 OR #32 OR #33 OR #34 OR #35 OR #36 OR #37 OR #38 OR #39 OR #40 OR #41 OR #42 OR #43 OR #44 OR #45 OR #46 OR #47 OR #48 OR #49 OR #50 OR #51 OR #52 OR #53 OR #54 OR #55 OR #56 OR #57 OR #58 OR #59 OR #60 OR #61 OR #62 OR #63 OR #64 OR #65 OR #66 OR #67 OR #68 OR #69 OR #70 OR #71 OR #72 OR #73 OR #74 OR #75 OR #76 OR #77 OR #78 OR #79 OR #80 OR #81 OR #82 OR #83 OR #84 OR #85 OR #86 OR #87 OR #88 OR #89 OR #90 OR #91 OR #92 OR #93 OR #94 OR #95 OR #96 OR #97 OR #98 OR #99
#101	(antenatal or "ante-natal"):TI,KW
#102	(prenatal or "pre-natal"):TI,KW
#103	(perinatal or "peri-natal"):TI,KW
#104	(intrapartum or "intra-partum"):TI,KW
#105	(postpartum or "post-partum"):TI,KW
#106	(postnatal or "post-natal"):TI,KW
#107	(preconception or pre-conception):TI,KW
#108	("pregnancy care" or (pregnancy NEXT outcom*) or (pregnancy NEXT complication*)):TI,KW
#109	("maternal care" or (maternal NEXT outcom*) or (maternal NEXT complication*)):TI,KW
#110	("obstetric care" or (obstetric NEXT outcom*) or (obstetric NEXT complication*)):TI,KW
#111	(birth or childbirth):ti,kw
#112	(labor or labour):ti,kw
#113	(Nulligrav* or nullipar* or primigrav* or primipar* or multigrav* or multipar*):ti,kw
#114	pregnan*:ti,kw
#115	MeSH descriptor: [Pregnancy] explode all trees
#116	MeSH descriptor: [Pregnancy Outcome] explode all trees
#117	MeSH descriptor: [Pregnancy Complications] explode all trees
#118	MeSH descriptor: [Preconception Care] explode all trees
#119	MeSH descriptor: [Prenatal Care] explode all trees
#120	MeSH descriptor: [Perinatal Care] explode all trees
#121	MeSH descriptor: [Postnatal Care] explode all trees
#122	MeSH descriptor: [Prenatal Diagnosis] explode all trees
#123	MeSH descriptor: [Pregnancy Tests] explode all trees
#124	MeSH descriptor: [Prenatal Exposure Delayed Effects] explode all trees
#125	MeSH descriptor: [Prenatal Education] explode all trees
#126	MeSH descriptor: [Maternal Health Services] explode all trees
#127	MeSH descriptor: [Delivery, Obstetric] explode all trees
#128	#101 OR #102 OR #103 OR #104 OR #105 OR #106 OR #107 OR #108 OR #109 OR #110 OR #111 OR #112 OR #113 OR #114 OR #115 OR #116 OR #117 OR #118 OR #119 OR #120 OR #121 OR #122 OR #123 OR #124 OR #125 OR #126 R #127
#129	#100 AND #128
#130	(clinicaltrials.gov):so
#131	(www.who.int):so
#132	#129 not (#130 OR #131)

For peer review only

CINHAL Plus via EBSCOHost <1937 to October 17, 2021>

S1	TI "self care" OR SU "self care" OR TI selfcare OR SU selfcare
S2	TI (home or "home-based" or homebased) N3 care) OR SU (home or "home-based" or homebased) N3 care)
S3	(MH "Self Care+")
S4	TI "self manag*" OR SU "self manag*" OR TI selfmanag* OR SU selfmanag*
S5	TI (home or "home-based" or homebased) N3 manag*) OR SU (home or "home-based" or homebased) N3 manag*)
S6	(MH "Self-Management")
S7	TI "self medicat*" OR SU "self medicat*" OR TI selfmedicat* OR SU selfmedicat*
S8	TI (home or "home-based" or homebased) N3 (medicat* or medicine)) OR SU (home or "home-based" or homebased) N3 (medicat* or medicine))
S9	(MH "Self Medication")
S10	TI "self treat*" OR SU "self treat*" OR TI selftreat* OR SU selftreat*
S11	TI (home or "home-based" or homebased) N3 treat*) OR SU (home or "home-based" or homebased) N3 treat*)
S12	TI "self examin*" OR SU "self examin*" OR TI selfexamin* OR SU selfexamin*
S13	TI (home or "home-based" or homebased) N3 exam*) OR SU (home or "home-based" or homebased) N3 exam*)
S14	(MH "Vulvar Self-Examination")
S15	TI "self inject*" OR SU "self inject*" OR TI "selfinject*" OR SU "selfinject*"
S16	TI (home or "home-based" or homebased) N3 inject*) OR SU (home or "home-based" or homebased) N3 inject*)
S17	TI "self administ*" OR SU "self administ*" OR TI selfadminist* OR SU selfadminist*"
S18	TI (home or "home-based" or homebased) N3 (administ* not administrator*)) OR SU (home or "home-based" or homebased) N3 (administ* not administrator*))
S19	(MH "Self Administration+")
S20	TI ("self use" or "self usage") OR SU ("self use" or "self usage")
S21	TI (selfuse or selfusage) OR SU (selfuse or selfuage)
S22	TI "self test*" OR SU "self test*" OR TI selftest* OR SU selftest*
S23	TI (home or "home-based" or homebased) N3 (test* or kits or kit)) OR SU (home or "home-based" or homebased) N3 (test* or kits or kit))
S24	(MH "Self-Testing")
S25	TI "self sampl*" OR SU "self sampl*" OR TI selfsampl* OR SU selfsampl*
S26	TI (home or "home-based" or homebased) N3 sampl*) OR SU (home or "home-based" or homebased) N3 sampl*)
S27	TI "self screen*" OR SU "self screen*" OR TI selfscreen* OR SU selfscreen*
S28	TI (home or "home-based" or homebased) N3 screen*) OR SU (home or "home-based" or homebased) N3 screen*)
S29	TI "self diagnos*" OR SU "self diagnos*" OR TI selfdiagnos* OR SU selfdiagnos*
S30	TI (home or "home-based" or homebased) N3 diagnos*) OR SU (home or "home-based" or homebased) N3 diagnos*)
S31	(MH "Self-Diagnosis+")
S32	TI "self collect*" OR SU "self collect*" OR TI selfcollect* OR SU selfcollect*
S33	TI (home or "home-based" or homebased) N3 collect*) OR SU (home or "home-based" or homebased) N3 collect*)
S34	TI "self monitor*" OR SU "self monitor*" OR TI selfmonitor* OR SU selfmonitor*
S35	TI (home or "home-based" or homebased) N3 monitor*) OR SU (home or "home-based" or homebased) N3 monitor*)
S36	(MH "Blood Glucose Self-Monitoring")
S37	TI "self assess*" OR SU "self assess*" OR TI selfassess* OR SU selfassess*
S38	TI (home or "home-based" or homebased) N3 assess*) OR SU (home or "home-based" or homebased) N3 assess*)
S39	(MH "Self Assessment")
S40	TI "self evaluat*" OR SU "self evaluat*" OR TI "selfevaluat*" OR SU "selfevaluat*"

S41	TI (home or "home-based" or homebased) N3 evaluat*) OR SU (home or "home-based" or homebased) N3 evaluat*)
S42	TI "self aware*" OR SU "self aware*"
S43	TI selfaware* OR SU selfaware*
S44	(MH "Self-Awareness")
S45	TI "self help*" OR SU "self help*"
S46	TI selfhelp* OR SU selfhelp*
S47	TI "self educat*" OR SU "self educat*"
S48	TI selfeducat* OR SU selfeducat*
S49	TI "self regulat*" OR SU "self regulat*"
S50	TI selfregulat* OR SU selfregulat*
S51	(MH "Self Regulation+")
S52	TI "self-control" OR SU "self-control" OR TI selfcontrol OR SU selfcontrol
S53	TI (home or "home-based" or homebased) N3 control) OR SU (home or "home-based" or homebased) N3 control)
S54	TI "self efficac*" OR SU "self efficac*"
S55	TI selfeffica* OR SU selfeffica*
S56	TI "self determin*" OR SU "self determin*"
S57	TI selfdetermin* OR SU selfdetermin*
S58	(MH "Patient Autonomy")
S59	TI ("patient-cent*" N2 care) OR SU ("patient-cent*" N2 care)
S60	TI ("person-cent*" N2 care) OR SU ("person-cent*" N2 care)
S61	(MH "Breast Self-Examination")
S62	TI "patient-orient*" N2 care OR SU "patient-orient*" N2 care
S63	TI SMBG OR SU SMBG
S64	(MH "Self Disclosure")
S65	TI "selfrelian*" OR SU "selfrelian*"
S66	TI "self relian*" OR SU "self relian*"
S67	TI "self advoca*" OR SU "self advoca*"
S68	TI "selfadvoca*" OR SU "selfadvoca*"
S69	(TI (self-adjust* N3 (medication* or drug* or dosage* or doses or dosing))) OR AB ((self-adjust* N3 (medication* or drug* or dosage* or doses or dosing)))
S70	TI "self measur*" OR SU "self measur*" OR TI "selfmeasur*" OR SU "selfmeasur*"
S71	TI (home or "home-based" or homebased) N3 measur*) OR SU (home or "home-based" or homebased) N3 measur*)
S72	TI (("non-pharmacological" or "non-pharmaceutical" or "non-clinical" or "non-medical")) OR SU (("non-pharmacological" or "non-pharmaceutical" or "non-clinical" or "non-medical"))
S73	TI (((prevention or preventive) N3 (infectious or infection*)))
S74	TI ((yeast or candida) and (prevent* or reduc* or test* or diagnos*)) OR SU ((yeast or candida) and (prevent* or reduc* or test* or diagnos*))
S75	TI (postpartum N1 (anemia or anaemia)) OR SU (postpartum N1 (anemia or anaemia))
S76	TI (hpv or papillo*) and (prevent* or reduc*)) OR SU (hpv or papillo*). and (prevent* or reduc*))
S77	TI ((cmv or cytomegalo*) AND (prevent* or reduc*)) OR SU ((cmv or cytomegalo*) AND (prevent* or reduc*))
S78	TI (torch and prevent*) OR SU (torch and prevent*)
S79	TI (zika and (prevent* or reduc* or practices or strategies)) OR SU (zika and (prevent* or reduc* or practices or strategies))
S80	TI (malaria) and (TI (prevent* or reduc* or practices or strategies) OR SU (prevent* or reduc* or practices or strategies))
S81	TI ((mosquito* or "vector-borne") and (prevent* or reduc* or practices or strategies)) OR SU ((mosquito* or "vector-borne") and (prevent* or reduc* or practices or strategies))
S82	TI ((sti or stis or std or stds or "sexually-transmitted") and (prevention or reduc* or practices or strategies)) OR SU ((sti or stis or std or stds or "sexually-transmitted") and (prevention or reduc* or practices or strategies))
S83	S1 OR S2 OR S3 OR S4 OR S5 OR S6 OR S7 OR S8 OR S9 OR S10 OR S11 OR S12 OR S13 OR S14 OR S15 OR S16 OR S17 OR S18 OR S19 OR S20 OR S21 OR S22 OR S23 OR S24 OR S25 OR S26 OR

	S27 OR S28 OR S29 OR S30 OR S31 OR S32 OR S33 OR S34 OR S35 OR S36 OR S37 OR S38 OR S39 OR S40 OR S41 OR S42 OR S43 OR S44 OR S45 OR S46 OR S47 OR S48 OR S49 OR S50 OR S51 OR S52 OR S53 OR S54 OR S55 OR S56 OR S57 OR S58 OR S59 OR S60 OR S61 OR S62 OR S63 OR S64 OR S65 OR S66 OR S67 OR S68 OR S69 OR S70 OR S71 OR S72 OR S73 OR S74 OR S75 OR S76 OR S77 OR S78 OR S79 OR S80 OR S81 OR S82
S84	TI (antenatal or "ante-natal") OR SU (antenatal or "ante-natal")
S85	TI (prenatal or "pre-natal") OR SU (prenatal or "pre-natal")
S86	TI (perinatal or "peri-natal") OR SU (perinatal or "peri-natal")
S87	TI (intrapartum or "intra-partum") OR SU (intrapartum or "intra-partum")
S88	TI (postnatal or "post-natal") OR SU (postnatal or "post-natal")
S89	TI (postpartum or "post-partum") OR SU (postpartum or "post-partum")
S90	TI (preconception or "pre-conception") OR SU (preconception or "pre-conception")
S91	TI (pregnancy N1 (care or outcome* or complication*)) OR SU (pregnancy N1 (care or outcome* or complication*))
S92	TI (maternal N1 (care or outcome* or complication*)) OR SU (maternal N1 (care or outcome* or complication*))
S93	TI (obstetric N1 (care or outcome* or complication*)) OR SU (obstetric N1 (care or outcome* or complication*))
S94	TI (birth or childbirth) OR SU (birth or childbirth)
S95	TI (labor or labour) OR SU (labor or labour)
S96	TI (pregnan*) OR SU (pregnan*)
S97	(MH "Pregnancy Outcomes")
S98	(MH "Pregnancy Complications+")
S99	TI (Nulligrav* or nullipar* or primigrav* or primipar* or multigrav* or multipar*) OR SU (Nulligrav* or nullipar* or primigrav* or primipar* or multigrav* or multipar*)
S100	(MH "Postnatal Period+") OR (MH "Periconceptual Period")
S101	(MH "Pregnancy+") OR (MH "Pregnancy, Multiple") OR (MH "Pregnancy Trimesters+")
S102	(MH "Pregnancy Tests+")
S103	(MH "Prenatal Diagnosis+")
S104	(MH "Prenatal Exposure Delayed Effects") OR (MH "Prenatal Nutritional Physiology")
S105	(MH "Perinatal Risk (Saba CCC)+")
S106	(MH "Postpartum Care (Saba CCC)") OR (MH "Postnatal Care+")
S107	(MH "Prenatal Care") OR (MH "Prenatal Care (Iowa NIC)")
S108	(MH "Intrapartum Care+")
S109	(MH "Childbirth education")
S110	(MH "Maternal Nutritional Physiology+") OR (MH "Maternal Exposure")
S111	(MH "Maternal Health Services+")
S112	S84 OR S85 OR S86 OR S87 OR S88 OR S89 OR S90 OR S91 OR S92 OR S93 OR S94 OR S95 OR S96 OR S97 OR S98 OR S99 OR S100 OR S101 OR S102 OR S103 OR S104 OR S105 OR S106 OR S107 OR S108 OR S109 OR S110 OR S111
S113	(MH "Systematic Review") OR (MH "Meta Analysis")
S114	MH (random assignment) OR MH (cluster sample) OR MH (crossover design) OR MH (placebos)
S115	PT (Systematic review) or PT (clinical trial) OR PT (nursing interventions) OR PT (randomized controlled trial)
S116	(MH "Study Design+") OR (MH "Clinical Research+") OR (MH "Comparative Studies+") OR (MH "Epidemiological Research") OR (MH "Evaluation Research+") OR (MH "Multicenter Studies") OR (MH "Field Studies") OR (MH "Pilot Studies") OR (MH "Secondary Analysis")
S117	TI ((metaanalysis or "meta-analysis")) OR SU ((metaanalysis or "meta-analysis"))
S118	TI (((literature or systematic or quantitative or integrative) N2 (overview or review))) OR SU (((literature or systematic or quantitative or integrative) N2 (overview or review)))
S119	TI ((clinical or randomised or randomized or experimental or control or controlled or blind*) N10 (trial or study)) OR SU ((clinical or randomised or randomized or experimental or control or controlled or blind*) N10 (trial or study))
S120	TI (trial) AND (AB (randomised or randomized or (random* N3 allocat*) or placebo or control or controlled or blinded or blinding) OR SU (randomised or randomized or (random* N3 allocat*) or placebo or control or controlled or blinded or blinding))

For peer review only

S121	AB ((experiment* or intervention* or study or control or controlled or allocat*) N3 group*)
S122	TI ((pretest or pre-test or posttest or post-test or pre-post)) OR SU ((pretest or pre-test or posttest or post-test or pre-post))
S123	TI ((quasi-experiment* or quasiexperiment*)) OR SU ((quasi-experiment* or quasiexperiment*))
S124	TI (((prospective or retrospective or observational or longitudinal or observational or population or interventional) n4 (analysis or stud* or cohort))) OR SU (((prospective or retrospective or observational or longitudinal or observational or population or interventional) n4 (analysis or stud* or cohort)))
S125	TI (((cohort or evaluation or comparative or pilot) n3 (study or analysis))) OR SU (((cohort or evaluation or comparative or pilot) n3 (study or analysis)))
S126	TI cross-sectional OR SU cross-sectional
S127	TI mixed-methods OR SU mixed-methods
S128	S113 OR S114 OR S115 OR S116 OR S117 OR S118 OR S119 OR S120 OR S121 OR S122 OR S123 OR S124 OR S125 OR S126 OR S127
S129	(MH "Case Studies") OR (MH "Case Control Studies+")
S130	(MH "Qualitative Studies+") OR (MH "Time and Motion Studies+") OR (MH "Genome wide association study+")
S131	PT masters thesis or PT doctoral dissertation OR PT editorial OR PT commentary OR PT case study
S132	TI ("case stud*") or AB ("case stud*" N5 (method* or design))
S133	TI ("case series") or AB ("case series" N5 (method* or design))
S134	TI ("case control*") or AB ("case control*" N5 (method* or design))
S135	TI (editorial or comment or letter or conference or proceeding* or thesis or dissertation)
S136	(((MH "Animals+") OR (MH "Animal Studies") OR (TI "animal model*")) NOT (MH "human"))
S137	AB ((rat or rats or mice or mouse or animal) N3 (model or study or method* or design)) or TI (mice or mouse or rat or rats or "animal study")
S138	S129 OR S130 OR S131 OR S132 OR S133 OR S134 OR S135 OR S136 OR S137
S139	S83 and S112 and S128 NOT S138

For peer review only

Ovid MEDLINE(R) and Epub Ahead of Print, In-Process, In-Data-Review & Other Non-Indexed Citations, Daily and Versions(R) <1946 to October 17, 2021>

1	self-care.ti,kf.
2	selfcare.ti,kf.
3	exp self care/
4	((home or "home-based" or homebased) adj3 care).ti,kf.
5	self-manag*.ti,kf.
6	selfmanag*.ti,kf.
7	exp Self-Management/
8	((home or "home-based" or homebased) adj3 manag*).ti,kf.
9	self-medicat*.ti,kf.
10	selfmedicat*.ti,kf.
11	((home or "home-based" or homebased) adj3 (medicat* or medicine)).ti,kf.
12	exp self medication/
13	self-treat*.ti,kf.
14	selftreat*.ti,kf.
15	((home or "home-based" or homebased) adj3 treat*).ti,kf.
16	self-examin*.ti,kf.
17	selfexamin*.ti,kf.
18	exp self examination/
19	((home or "home-based" or homebased) adj3 exam*).ti,kf.
20	self-inject*.ti,kf.
21	selfinject*.ti,kf.
22	exp self injection/
23	((home or "home-based" or homebased) adj3 inject*).ti,kf.
24	self-administ*.ti,kf.
25	selfadminist*.ti,kf.
26	exp Self Administration/
27	((home or "home-based" or homebased) adj3 (administ* not administrator*)).ti,kf.
28	("self-use" or "self-usage").ti,kf.
29	(selfuse or selfusage).ti,kf.
30	self-test*.ti,kf.
31	selftest*.ti,kf.
32	exp Self-Testing/
33	((home or "home-based" or homebased) adj3 (test* or kits or kit)).ti,kf.
34	self-sampl*.ti,kf.
35	selfsampl*.ti,kf.
36	((home or "home-based" or homebased) adj3 sampl*).ti,kf.
37	self-screen*.ti,kf.
38	selfscreen*.ti,kf.
39	((home or "home-based" or homebased) adj3 screen*).ti,kf.
40	self-diagnos*.ti,kf.
41	selfdiagnos*.ti,kf.
42	((home or "home-based" or homebased) adj3 diagnos*).ti,kf.
43	self-collect*.ti,kf.
44	selfcollect*.ti,kf.
45	((home or "home-based" or homebased) adj3 collect*).ti,kf.
46	self-monitor*.ti,kf.
47	selfmonitor*.ti,kf.
48	((home or "home-based" or homebased) adj3 monitor*).ti,kf.
49	exp Blood Glucose Self-Monitoring/
50	SMBG.ti,kf.
51	self-measur*.ti,kf.

52	selfmeasu*.ti,kf.
53	((home or "home-based" or homebased) adj3 measur*).ti,kf.
54	self-control*.ti,kf.
55	selfcontrol.ti,kf.
56	exp self-control/
57	((home or "home-based" or homebased) adj3 control).ti,kf.
58	self-assess*.ti,kf.
59	selfassess.ti,kf.
60	exp Self-assessment/
61	((home or "home-based" or homebased) adj3 assess*).ti,kf.
62	self-evaluat*.ti,kf.
63	selfevaluat*.ti,kf.
64	exp self evaluation/
65	exp Diagnostic Self Evaluation/
66	((home or "home-based" or homebased) adj3 evaluat*).ti,kf.
67	Self-aware*.ti,kf.
68	selfaware*.ti,kf.
69	self-help*.ti,kf.
70	selfhelp*.ti,kf.
71	exp self help/
72	self-educat*.ti,kf.
73	selfeducat*.ti,kf.
74	self-regulat*.ti,kf.
75	selfregulat*.ti,kf.
76	exp self concept/
77	exp self disclosure/
78	self-effica*.ti,kf.
79	selfeffica*.ti,kf.
80	exp Self-efficacy/
81	self-determin*.ti,kf.
82	selfdetermin*.ti,kf.
83	self-reliance.ti,kf.
84	selfreliance.ti,kf.
85	self-advoc*.ti,kf.
86	selfadvoc*.ti,kf.
87	(self-adjust* adj3 (medication* or drug* or dosage* or doses or dosing)).ti,ab,kf.
88	(patient-cent* adj1 care).ti,kf.
89	(person-cent* adj1 care).ti,kf.
90	(patient-oriented adj1 care).ti,kf.
91	exp Patient-Centered Care/
92	(family-cent* adj1 care).ti,kf.
93	("non-pharmacological" or "non-pharmaceutical" or "non-clinical" or "non-medical").ti,kf.
94	((prevention or preventive) adj3 (infectious or infection*)).ti,kf.
94	(yeast or candida).ti,kf. and (prevent* or reduc* or test* or diagnos*).ti,kf.
95	(postpartum adj1 (anemia or anaemia)).ti,kf.
96	(hpv or papillo*).ti,kf. and (prevent* or reduc*).ti,kf.
97	(cmv or cytomegalo*).ti,kf. and (prevent* or reduc*).ti,kf.
98	torch.ti,kf. and prevent*.ti,ab,kf.
99	(zika and (prevent* or reduc* or practices or strategies)).ti,kf.
100	(malaria.ti.) and (prevent* or reduc* or practices or strategies).ti,kf.
101	((mosquito* or "vector-borne") and (prevent* or reduc* or practices or strategies)).ti,kf.
102	(sti or stis or std or stds or "sexually-transmitted").ti,kf. and (prevention or reduc* or practices or strategies).ti,kf.

103	or/1-102
104	antenatal.ti,kf.
105	(prenatal or pre-natal).ti,kf.
106	(perinatal or peri-natal).ti,kf.
107	(intrapartum or intra-partum).ti,kf.
108	(postpartum or post-partum).ti,kf.
109	(postnatal or post-natal).ti,kf.
110	(preconception or pre-conception).ti,kf.
111	("pregnancy care" or "pregnancy outcom*" or "pregnancy complication*").ti,kf.
112	("maternal care" or "maternal outcom*" or "maternal complication*").ti,kf.
113	("obstetric care" or "obstetric outcom*" or "obstetric complication*").ti,kf.
114	birth.ti,kf.
115	childbirth.ti,kf.
116	labour.ti,kf.
117	labor.ti,kf.
118	(Nulligrav* or nullipar* or primigrav* or primipar* or multigrav* or multipar*).ti,kf.
119	pregnan*.ti,kf.
120	exp pregnancy/
121	exp pregnancy outcome/
122	exp Pregnancy Complications/
123	exp Preconception care/
124	exp perinatal care/
125	exp prenatal care/
126	exp postnatal care/
127	exp pregnancy tests/
128	exp prenatal diagnosis/
129	exp Prenatal Exposure Delayed Effects/
130	exp prenatal education/
131	exp maternal health services/
132	exp Delivery, Obstetric/
133	or/104-132
134	exp Systematic Review/ or exp meta-analysis/
135	((literature or systematic or quantitative or integrative) adj2 (overview or review)).ti,ab,kf,sh.
136	(metaanalysis or meta-analysis).ti,ab,kf,sh.
137	exp Clinical trial/ or Clinical Study/ or exp Comparative Study/ or exp Evaluation Study/ or exp Multicenter Study/ or exp Observational Study/ or exp Evaluation Studies as Topic/
138	exp Epidemiologic Research Design/ not (Genome-Wide Association Study/ or exp Reproducibility of Results/)
139	exp Epidemiologic Study Characteristics/
140	((clinical or randomised or randomized or experimental or controlled or control or blind*) adj10 (trial or study)).ti,kf.
141	trial.ti. and (randomised or randomized or (random* adj3 allocat*) or placebo or control or controlled or blinded or blinding).ab,kf.
142	((experiment* or intervention* or study or control or controlled or allocat*) adj3 group*).ab.
143	(pretest or "pre-test" or posttest or "post-test" or "pre-post" or "quasi-experiment*" or quasiexperiment* or "cross-sectional" or "mixed-method*").ti,kf.
144	((prospective or retrospective or observational or longitudinal or observational or population or interventional) adj4 (analysis or stud* or cohort)).ti,kf.
145	((cohort or evaluation or comparative or pilot) adj3 (study or analysis)).ti,kf.
146	or/134-145
147	case study.ti. or ("case study" adj5 (method* or design)).ab.
148	case report.ti. or ("case report" adj5 (method* or design)).ab.
149	case series.ti. or ("case series" adj5 (method* or design)).ab.
150	case control.ti. or ("case control" adj5 (method* or design)).ab.

151	(editorial or "conference abstract" or "conference review" or "conference paper" or "poster abstract*" or erratum or commentary or (letter adj3 editor)).ti.
152	(Clinical Conference or Editorial or Letter or Meeting Abstracts or Newspaper Article).pt.
153	exp Case-Control Studies/ or exp Case Reports/ or exp case Studies/ or exp Clinical conference/
154	(animals/ or exp animal experiment/) not (humans/ and animals/)
155	((rat or rats or mice or mouse or animal) adj3 (model or study or method* or design)).ab. or (mice or mouse or rat or rats or "animal study").ti.
156	or/147-155
157	(103 and 133 and 146) not 156

For peer review only

Embase Classic+Embase <1947 to October 17, 2021>

1	self-care.ti,kw.
2	selfcare.ti,kw.
3	exp self care/
4	((home or "home-based" or homebased) adj3 care).ti,kw.
5	self-manag*.ti,kw.
6	selfmanag*.ti,kw.
7	((home or "home-based" or homebased) adj3 manag*).ti,kw.
8	self-medicat*.ti,kw.
9	selfmedicat*.ti,kw.
10	exp self medication/
11	((home or "home-based" or homebased) adj3 (medicat* or medicine)).ti,kw.
12	self-treat*.ti,kw.
13	selftreat*.ti,kw.
14	((home or "home-based" or homebased) adj3 treat*).ti,kw.
15	self-examin*.ti,kw.
16	selfexamin*.ti,kw.
17	exp self examination/
18	((home or "home-based" or homebased) adj3 exam*).ti,kw.
19	self-inject*.ti,kw.
20	selfinject*.ti,kw.
21	exp self injection/
22	((home or "home-based" or homebased) adj3 inject*).ti,kw.
23	self-administ*.ti,kw.
24	selfadminist*.ti,kw.
25	exp drug self administration/
26	((home or "home-based" or homebased) adj3 (administ* not administrator*)).ti,kw.
27	("self-use" or "self usage").ti,kw.
28	(selfuse or selfusage).ti,kw.
29	self-test*.ti,kw.
30	selftest*.ti,kw.
31	((home or "home-based" or homebased) adj3 (test* or kits or kit)).ti,kw.
32	self-sampl*.ti,kw.
33	selfsampl*.ti,kw.
34	((home or "home-based" or homebased) adj3 sampl*).ti,kw.
35	self-screen*.ti,kw.
36	selfscreen*.ti,kw.
37	((home or "home-based" or homebased) adj3 screen*).ti,kw.
38	self-diagnos*.ti,kw.
39	selfdiagnos*.ti,kw.
40	((home or "home-based" or homebased) adj3 diagnos*).ti,kw.
41	self-collect*.ti,kw.
42	selfcollect*.ti,kw.
43	((home or "home-based" or homebased) adj3 collect*).ti,kw.
44	self-monitor*.ti,kw.
45	selfmonitor*.ti,kw.
46	exp self monitoring/
47	((home or "home-based" or homebased) adj3 monitor*).ti,kw.
48	*self monitoring of blood glucose/
49	*self monitoring of blood pressure/
50	SMBG.ti,kw.
51	self-measur*.ti,kw.
52	selfmeasu*.ti,kw.

53	((home or "home-based" or homebased) adj3 measur*).ti,kw.
54	self-control*.ti,kw.
55	selfcontrol.ti,kw.
56	exp self-control/
57	((home or "home-based" or homebased) adj3 control).ti,kw.
58	self-assess*.ti,kw.
59	selfassess.ti,kw.
60	((home or "home-based" or homebased) adj3 assess*).ti,kw.
61	self-evaluat*.ti,kw.
62	selfevaluat*.ti,kw.
63	exp self evaluation/
64	((home or "home-based" or homebased) adj3 evaluat*).ti,kw.
65	Self-aware*.ti,kw.
66	selfaware*.ti,kw.
67	self-help*.ti,kw.
68	selfhelp*.ti,kw.
69	exp self help/
70	self-educat*.ti,kw.
71	selfeducat*.ti,kw.
72	self-regulat*.ti,kw.
73	selfregulat*.ti,kw.
74	exp self esteem/
75	exp self disclosure/
76	self-effica*.ti,kw.
77	selfeffica*.ti,kw.
78	self-determin*.ti,kw.
79	selfdetermin*.ti,kw.
80	self-reliance.ti,kw.
81	selfreliance.ti,kw.
82	self-advoc*.ti,kw.
83	selfadvoc*.ti,kw.
84	(self-adjust* adj3 (medication* or drug* or dosage* or doses or dosing)).tw.
85	(patient-cent* adj1 care).ti,kw.
86	(person-cent* adj1 care).ti,kw.
87	(patient-oriented adj1 care).ti,kw.
88	(family-cent* adj1 care).ti,kw.
89	("non-pharmacological" or "non-pharmaceutical" or "non-clinical" or "non-medical").ti,kw.
90	((prevention or preventive) adj3 (infectious or infection*)).ti,kw.
91	(yeast or candida).ti,kw. and (prevent* or reduc* or test* or diagnos*).ti,kw.
92	(postpartum adj1 (anemia or anaemia)).ti,kw.
93	(hpv or papillo*).ti,kw. and (prevent* or reduc*).ti,kw.
94	(cmv or cytomegalo*).ti,kw. and (prevent* or reduc*).ti,kw.
95	torch.ti,kw. and prevent*.ti,ab,kw.
96	(zika and (prevent* or reduc* or practices or strategies)).ti,kw.
97	(malaria.ti.) and (prevent* or reduc* or practices or strategies).ti,kw.
98	((mosquito* or "vector-borne") and (prevent* or reduc* or practices or strategies)).ti,kw.
99	(sti or stis or std or stds or "sexually-transmitted").ti,kw. and (prevention or reduc* or practices or strategies).ti,kw.
100	or/1-99
101	(preconception or pre-conception).ti,kw.
102	antenatal.ti,kw.
103	(prenatal or pre-natal).ti,kw.
104	(perinatal or peri-natal).ti,kw.

105	(intrapartum or intra-partum).ti,kw.
106	(postpartum or post-partum).ti,kw.
107	(postnatal or post-natal).ti,kw.
108	("pregnancy care" or "pregnancy outcom*" or "pregnancy complication*").ti,kw.
109	("maternal care" or "maternal outcom*" or "maternal complication*").ti,kw.
110	("obstetric care" or "obstetric outcom*" or "obstetric complication*").ti,kw.
111	birth.ti,kw.
112	childbirth.ti,kw.
113	labour.ti,kw.
114	labor.ti,kw.
115	(Nulligrav* or nullipar* or primigrav* or primipar* or multigrav* or multipar*).ti,kw.
116	pregnan*.ti,kw.
117	exp pregnancy/
118	exp pregnancy complication/
119	exp pregnancy outcome/
120	exp obstetric procedure/
121	exp maternal health services/
122	exp prenatal exposure/
123	exp childbirth education/
124	or/101-123
125	exp "systematic review"/ or exp meta analysis/ or "systematic review (topic)"/ or "meta analysis (topic)"/
126	(review or meta-analysis).pt.
127	(metaanalysis or meta-analysis).ti,ab,kw,sh.
128	((literature or systematic or quantitative or integrative) adj2 (overview or review)).ti,ab,kw,sh.
129	exp clinical study/ or exp comparative study/ or exp experimental study/ or exp feasibility study/ or exp field study/ or exp observational study/ or exp quasi experimental study/ or exp pilot study/ or exp experimental study/ or exp comparative study/
130	clinical trial (topic)/ or parallel design/ or crossover procedure/ or triple blind procedure/ or double blind procedure/ or single blind procedure/
131	exp methodology/ and (randomization/ or placebo/)
132	control group/ or nonequivalent control group/
133	intention to treat analysis/ or secondary analysis/
134	pretest posttest control group design/ or experimental design/ or quantitative study/ or participatory research/
135	cohort analysis/ or correlational study/ or cross-sectional study/
136	((clinical or randomised or randomized or experimental or controlled or control or blind*) adj10 (trial or study)).ti,kw.
137	trial.ti. and (randomised or randomized or (random* adj3 allocat*) or placebo or control or controlled or blinded or blinding).ab,kw.
138	((experiment* or intervention* or study or control or controlled or allocat*) adj3 group*).ab.
139	((prospective or retrospective or observational or longitudinal or observational or population or interventional) adj4 (analysis or stud* or cohort)).ti,kw.
140	((cohort or evaluation or comparative or pilot) adj3 (study or analysis)).ti,kw.
141	(pretest or "pre-test" or posttest or "post-test" or "pre-post" or "quasi-experiment*" or quasiexperiment* or "cross-sectional" or "mixed-method*").ti,kw.
142	or/125-141
143	exp case control study/ or exp case report/ or exp case study/
144	case study.ti. or ("case study" adj5 (method* or design)).ab.
145	case report.ti. or ("case report" adj5 (method* or design)).ab.
146	case series.ti. or ("case series" adj5 (method* or design)).ab.
147	case control.ti. or ("case control" adj5 (method* or design)).ab.
148	(editorial or conference or erratum or letter or note).pt.
149	(editorial or "conference abstract" or "conference review" or "conference paper" or "poster abstract*" or erratum or commentary or (letter adj3 editor)).ti.
150	(animals/ or exp animal experiment/) not (humans/ and animals/)

For peer review only

151	((rat or rats or mice or mouse or animal) adj3 (model or study or method* or design)).ab. or (mice or mouse or rat or rats or "animal study").ti.
152	or/143-151
153	(100 and 124 and 142) not 152

For peer review only

Ovid Emcare <1995 to 2021 Week 41>

1	self-care.ti,kw.
2	selfcare.ti,kw.
3	exp self care/
4	((home or "home-based" or homebased) adj3 care).ti,kw.
5	self-manag*.ti,kw.
6	selfmanag*.ti,kw.
7	((home or "home-based" or homebased) adj3 manag*).ti,kw.
8	self-medicat*.ti,kw.
9	selfmedicat*.ti,kw.
10	exp self medication/
11	((home or "home-based" or homebased) adj3 (medicat* or medicine)).ti,kw.
12	self-treat*.ti,kw.
13	selftreat*.ti,kw.
14	((home or "home-based" or homebased) adj3 treat*).ti,kw.
15	self-examin*.ti,kw.
16	selfexamin*.ti,kw.
17	exp self examination/
18	((home or "home-based" or homebased) adj3 exam*).ti,kw.
19	self-inject*.ti,kw.
20	selfinject*.ti,kw.
21	exp self injection/
22	((home or "home-based" or homebased) adj3 inject*).ti,kw.
23	self-administ*.ti,kw.
24	selfadminist*.ti,kw.
25	exp drug self administration/
26	((home or "home-based" or homebased) adj3 (administ* not administrator*)).ti,kw.
27	("self-use" or "self usage").ti,kw.
28	(selfuse or selfusage).ti,kw.
29	self-test*.ti,kw.
30	selftest*.ti,kw.
31	((home or "home-based" or homebased) adj3 (test* or kits or kit)).ti,kw.
32	self-sampl*.ti,kw.
33	selfsampl*.ti,kw.
34	((home or "home-based" or homebased) adj3 sampl*).ti,kw.
35	self-screen*.ti,kw.
36	selfscreen*.ti,kw.
37	((home or "home-based" or homebased) adj3 screen*).ti,kw.
38	self-diagnos*.ti,kw.
39	selfdiagnos*.ti,kw.
40	((home or "home-based" or homebased) adj3 diagnos*).ti,kw.
41	self-collect*.ti,kw.
42	selfcollect*.ti,kw.
43	((home or "home-based" or homebased) adj3 collect*).ti,kw.
44	self-monitor*.ti,kw.
45	selfmonitor*.ti,kw.
46	exp self monitoring/
47	((home or "home-based" or homebased) adj3 monitor*).ti,kw.
48	*self monitoring of blood glucose/
49	*self monitoring of blood pressure/
50	SMBG.ti,kw.
51	self-measur*.ti,kw.
52	selfmeasu*.ti,kw.

53	((home or "home-based" or homebased) adj3 measur*).ti,kw.
54	self-control*.ti,kw.
55	selfcontrol.ti,kw.
56	exp self-control/
57	((home or "home-based" or homebased) adj3 control).ti,kw.
58	self-assess*.ti,kw.
59	selfassess.ti,kw.
60	((home or "home-based" or homebased) adj3 assess*).ti,kw.
61	self-evaluat*.ti,kw.
62	selfevaluat*.ti,kw.
63	exp self evaluation/
64	((home or "home-based" or homebased) adj3 evaluat*).ti,kw.
65	Self-aware*.ti,kw.
66	selfaware*.ti,kw.
67	self-help*.ti,kw.
68	selfhelp*.ti,kw.
69	exp self help/
70	self-educat*.ti,kw.
71	selfeducat*.ti,kw.
72	self-regulat*.ti,kw.
73	selfregulat*.ti,kw.
74	exp self esteem/
75	exp self disclosure/
76	self-effica*.ti,kw.
77	selfeffica*.ti,kw.
78	self-determin*.ti,kw.
79	selfdetermin*.ti,kw.
80	self-reliance.ti,kw.
81	selfreliance.ti,kw.
82	self-advoc*.ti,kw.
83	selfadvoc*.ti,kw.
84	(self-adjust* adj3 (medication* or drug* or dosage* or doses or dosing)).tw.
85	(patient-cent* adj1 care).ti,kw.
86	(person-cent* adj1 care).ti,kw.
87	(patient-oriented adj1 care).ti,kw.
88	(family-cent* adj1 care).ti,kw.
89	("non-pharmacological" or "non-pharmaceutical" or "non-clinical" or "non-medical").ti,kw.
90	((prevention or preventive) adj3 (infectious or infection*)).ti,kw.
91	(yeast or candida).ti,kw. and (prevent* or reduc* or test* or diagnos*).ti,kw.
92	(postpartum adj1 (anemia or anaemia)).ti,kw.
93	(hpv or papillo*).ti,kw. and (prevent* or reduc*).ti,kw.
94	(cmv or cytomegalo*).ti,kw. and (prevent* or reduc*).ti,kw.
95	torch.ti,kw. and prevent*.ti,ab,kw.
96	(zika and (prevent* or reduc* or practices or strategies)).ti,kw.
97	(malaria.ti.) and (prevent* or reduc* or practices or strategies).ti,kw.
98	((mosquito* or "vector-borne") and (prevent* or reduc* or practices or strategies)).ti,kw.
99	(sti or stis or std or stds or "sexually-transmitted").ti,kw. and (prevention or reduc* or practices or strategies).ti,kw.
100	or/1-99
101	(preconception or pre-conception).ti,kw.
102	antenatal.ti,kw.
103	(prenatal or pre-natal).ti,kw.
104	(perinatal or peri-natal).ti,kw.

105	(intrapartum or intra-partum).ti,kw.
106	(postpartum or post-partum).ti,kw.
107	(postnatal or post-natal).ti,kw.
108	("pregnancy care" or "pregnancy outcom*" or "pregnancy complication*").ti,kw.
109	("maternal care" or "maternal outcom*" or "maternal complication*").ti,kw.
110	("obstetric care" or "obstetric outcom*" or "obstetric complication*").ti,kw.
111	birth.ti,kw.
112	childbirth.ti,kw.
113	labour.ti,kw.
114	labor.ti,kw.
115	(Nulligrav* or nullipar* or primigrav* or primipar* or multigrav* or multipar*).ti,kw.
116	pregnan*.ti,kw.
117	exp pregnancy/
118	exp pregnancy complication/
119	exp pregnancy outcome/
120	exp obstetric procedure/
121	exp maternal health services/
122	exp prenatal exposure/
123	exp childbirth education/
124	or/101-123
125	exp "systematic review"/ or exp meta analysis/ or "systematic review (topic)"/ or "meta analysis (topic)"/
126	(review or meta-analysis).pt.
127	(metaanalysis or meta-analysis).ti,ab,kw,sh.
128	((literature or systematic or quantitative or integrative) adj2 (overview or review)).ti,ab,kw,sh.
129	exp clinical study/ or exp comparative study/ or exp experimental study/ or exp feasibility study/ or exp field study/ or exp observational study/ or exp quasi experimental study/ or exp pilot study/ or exp experimental study/ or exp comparative study/
130	clinical trial (topic)/ or parallel design/ or crossover procedure/ or triple blind procedure/ or double blind procedure/ or single blind procedure/
131	exp methodology/ and (randomization/ or placebo/)
132	control group/ or nonequivalent control group/
133	intention to treat analysis/ or secondary analysis/
134	pretest posttest control group design/ or experimental design/ or quantitative study/ or participatory research/
135	cohort analysis/ or correlational study/ or cross-sectional study/
136	((clinical or randomised or randomized or experimental or controlled or control or blind*) adj10 (trial or study)).ti,kw.
137	trial.ti. and (randomised or randomized or (random* adj3 allocat*) or placebo or control or controlled or blinded or blinding).ab,kw.
138	((experiment* or intervention* or study or control or controlled or allocat*) adj3 group*).ab.
139	((prospective or retrospective or observational or longitudinal or observational or population or interventional) adj4 (analysis or stud* or cohort)).ti,kw.
140	((cohort or evaluation or comparative or pilot) adj3 (study or analysis)).ti,kw.
141	(pretest or "pre-test" or posttest or "post-test" or "pre-post" or "quasi-experiment*" or quasiexperiment* or "cross-sectional" or "mixed-method*").ti,kw.
142	or/125-141
143	exp case control study/ or exp case report/ or exp case study/
144	case study.ti. or ("case study" adj5 (method* or design)).ab.
145	case report.ti. or ("case report" adj5 (method* or design)).ab.
146	case series.ti. or ("case series" adj5 (method* or design)).ab.
147	case control.ti. or ("case control" adj5 (method* or design)).ab.
148	(editorial or conference or erratum or letter or note).pt.
149	(editorial or "conference abstract" or "conference review" or "conference paper" or "poster abstract*" or erratum or commentary or (letter adj3 editor)).ti.
150	(animals/ or exp animal experiment/) not (humans/ and animals/)

151	((rat or rats or mice or mouse or animal) adj3 (model or study or method* or design)).ab. or (mice or mouse or rat or rats or "animal study").ti.
152	or/143-151
153	(100 and 124 and 142) not 152

For peer review only

APA PsycInfo <1806 to October Week 2 2021>

1	self-care.ti,id.
2	selfcare.ti,id.
3	exp self care/
4	((home or "home-based" or homebased) adj3 care).ti,id.
5	self-manag*.ti,id.
6	selfmanag*.ti,id.
7	exp Self-Management/
8	((home or "home-based" or homebased) adj3 manag*).ti,id.
9	self-medicat*.ti,id.
10	selfmedicat*.ti,id.
11	exp self-medication/
12	((home or "home-based" or homebased) adj3 (medicat* or medicine)).ti,id.
13	self-treat*.ti,id.
14	selftreat*.ti,id.
15	((home or "home-based" or homebased) adj3 treat*).ti,id.
16	self-examin*.ti,id.
17	selfexamin*.ti,id.
18	exp self examination/
19	exp "Self-Examination (Medical)"/
20	((home or "home-based" or homebased) adj3 exam*).ti,id.
21	self-inject*.ti,id.
22	selfinject*.ti,id.
23	((home or "home-based" or homebased) adj3 inject*).ti,id.
24	self-administ*.ti,id.
25	selfadminist*.ti,id.
26	exp drug self administration/
27	((home or "home-based" or homebased) adj3 (administ* not administrator*)).ti,id.
28	("self-use" or "self-usage").ti,id.
29	(selfuse or selfusage).ti,id.
30	self-test*.ti,id.
31	selftest*.ti,id.
32	((home or "home-based" or homebased) adj3 (test* or kits or kit)).ti,id.
33	self-sampl*.ti,id.
34	selfsampl*.ti,id.
35	((home or "home-based" or homebased) adj3 sampl*).ti,id.
36	self-screen*.ti,id.
37	selfscreen*.ti,id.
38	((home or "home-based" or homebased) adj3 screen*).ti,id.
39	self-diagnos*.ti,id.
40	selfdiagnos*.ti,id.
41	((home or "home-based" or homebased) adj3 diagnos*).ti,id.
42	self-collect*.ti,id.
43	selfcollect*.ti,id.
44	((home or "home-based" or homebased) adj3 collect*).ti,id.
45	self-monitor*.ti,id.
46	selfmonitor*.ti,id.
47	exp self-monitoring/
48	((home or "home-based" or homebased) adj3 monitor*).ti,id.
49	SMBG.ti,id.
50	self-measur*.ti,id.
51	selfmeasu*.ti,id.

52	((home or "home-based" or homebased) adj3 measur*).ti,id.
53	self-control*.ti,id.
54	selfcontrol.ti,id.
55	exp self-control/
56	((home or "home-based" or homebased) adj3 control).ti,id.
57	self-assess*.ti,id.
58	selfassess.ti,id.
59	((home or "home-based" or homebased) adj3 assess*).ti,id.
60	self-evaluat*.ti,id.
61	selfevaluat*.ti,id.
62	exp self-evaluation/
63	((home or "home-based" or homebased) adj3 evaluat*).ti,id.
64	Self-aware*.ti,id.
65	selfaware*.ti,id.
66	self-perception/
67	self-help*.ti,id.
68	selfhelp*.ti,id.
69	exp self help/
70	self-educat*.ti,id.
71	selfeducat*.ti,id.
72	exp self-knowledge/
73	self-regulat*.ti,id.
74	selfregulat*.ti,id.
75	exp self-regulation/
76	exp emotional control/
77	exp emotional regulation/
78	exp emotional adjustment/
79	exp self esteem/
80	exp self disclosure/
81	self-effica*.ti,id.
82	selfeffica*.ti,id.
83	exp self-efficacy/
84	self-determin*.ti,id.
85	selfdetermin*.ti,id.
86	self-reliance.ti,id.
87	selfreliance.ti,id.
88	self-advoc*.ti,id.
89	selfadvoc*.ti,id.
90	exp self-advocacy/
91	(self-adjust* adj3 (medication* or drug* or dosage* or doses or dosing)).ti,id.
92	(patient-cent* adj1 care).ti,id.
93	(person-cent* adj1 care).ti,id.
94	(patient-oriented adj1 care).ti,id.
95	exp Patient Centered Care/
96	(family-cent* adj1 care).ti,id.
97	("non-pharmacological" or "non-pharmaceutical" or "non-clinical" or "non-medical").ti,id.
98	((prevention or preventive) adj3 (infectious or infection*)).ti,id.
99	(yeast or candida).ti,id. and (prevent* or reduc* or test* or diagnos*).ti,id.
100	(postpartum adj1 (anemia or anaemia)).ti,id.
101	(hpv or papillo*).ti,id. and (prevent* or reduc*).ti,id.
102	(cmv or cytomegalo*).ti,id. and (prevent* or reduc*).ti,id.
103	torch.ti,id. and prevent*.ti,ab,id.
104	(zika and (prevent* or reduc* or practices or strategies)).ti,id.

105	(malaria.ti.) and (prevent* or reduc* or practices or strategies).ti,id.
106	((mosquito* or "vector-borne") and (prevent* or reduc* or practices or strategies)).ti,id.
107	(sti or stis or std or stds or "sexually-transmitted").ti,id. and (prevention or reduc* or practices or strategies).ti,id.
108	or/1-107
109	antenatal.ti,id.
110	(prenatal or pre-natal).ti,id.
111	(perinatal or peri-natal).ti,id.
112	(intrapartum or intra-partum).ti,id.
113	(postpartum or post-partum).ti,id.
114	(postnatal or post-natal).ti,id.
115	(preconception or pre-conception).ti,id.
116	("pregnancy care" or "pregnancy outcom*" or "pregnancy complication*").ti,id.
117	("maternal care" or "maternal outcom*" or "maternal complication*").ti,id.
118	("obstetric care" or "obstetric outcom*" or "obstetric complication*").ti,id.
119	pregnan*.ti,id.
120	(childbirth or birth).ti,id.
121	(labor or labour).ti,id.
122	(Nulligrav* or nullipar* or primigrav* or primipar* or multigrav* or multipar*).ti,id.
123	exp pregnancy/
124	exp prenatal care/ or exp prenatal diagnosis/ or exp prenatal exposure/
125	exp perinatal period/ or exp antepartum period/ or exp intrapartum period exp postnatal period/ or exp perinatal period/
126	or/109-125
127	exp Systematic Review/ or exp meta-analysis/
128	((literature or systematic or quantitative or integrative) adj2 (overview or review)).ti,ab,id,sh.
129	(metaanalysis or meta-analysis).ti,ab,id,sh.
130	exp experimental design/ not exp single-case experimental design/
131	exp cohort analysis/ or exp mixed methods research/ or exp quantitative methods/ or exp quasi-experimental methods/
132	((clinical or randomised or randomized or experimental or controlled or control or blind*) adj10 (trial or study)).ti,id.
133	trial.ti. and (randomised or randomized or (random* adj3 allocat*) or placebo or control or controlled or blinded or blinding).ab,id.
134	((experiment* or intervention* or study or control or controlled or allocat*) adj3 group*).ab.
135	(pretest or "pre-test" or posttest or "post-test" or "pre-post" or "quasi-experiment*" or quasiexperiment* or "cross-sectional" or "mixed-method*").ti,id.
136	((prospective or retrospective or observational or longitudinal or observational or population or interventional) adj4 (analysis or stud* or cohort)).ti,id.
137	((cohort or evaluation or comparative or pilot) adj3 (study or analysis)).ti,id.
138	or/127-137
139	(editorial or "newspaper article" or conference* or commentary).ti.
140	case study.ti. or ("case study" adj5 (method* or design)).ab.
141	case report.ti. or ("case report" adj5 (method* or design)).ab.
142	case series.ti. or ("case series" adj5 (method* or design)).ab.
143	case control.ti. or ("case control" adj5 (method* or design)).ab.
144	exp Case Report/ or exp Case-control studies/ or exp cross-sectional studies/
145	(animals/ or exp animal experiment/) not (humans/ and animals/)
146	((rat or rats or mice or mouse or animal) adj3 (model or study or method* or design)).ab. or (mice or mouse or rat or rats or "animal study").ti.
147	or/139-146
148	(108 and 126 and 138) not 147

For peer review only - http://bmjopen.bmj.com/site/about/guidelines.xhtml

Supplementary File S3. Defining self-care interventions, a conceptual framework and examples for making judgements on specific interventions

Definitions of self-care in WHO Guideline on Self-care Interventions for Health and Wellbeing (2021)

Self-care is the ability of individuals, families and communities to promote health, prevent disease, maintain health, and cope with illness and disability with or without the support of a health worker.

Self-care recognizes individuals as active agents in managing their own health care, in areas including health promotion; disease prevention and control; self-medication; providing care to dependent persons, and rehabilitation, including palliative care.

Self-care interventions are evidence-based, quality tools that support self-care. They include medicines, counselling, diagnostics and/or digital technologies which can be accessed fully or partially outside of formal health services. Depending on the intervention, they can be used with or without the direct supervision of health workers.

Working definition of self-care in this review

For the purpose of this review, a *self-care intervention* is defined as any tool, resource or strategy designed to promote or facilitate self-care by women preparing to get pregnant, currently pregnant, labouring/birthing or in the postpartum period (up to 42 days after birth), for the purpose of improving the quality or coverage of maternity health care, and/or improving the health, well-being and experiences of the pregnant woman and her newborn.

A *self-care activity* includes those that can be taken by a preconceiving, pregnant, labouring/birthing or postpartum woman of her own accord towards the goals of (a) information-seeking and improving health literacy, (b) promoting mental health, self-efficacy and self-awareness, (c) increasing physical activity, (d) maintaining a healthy diet, (e) reducing risk and exposure avoidance, (d) good hygiene, and (7) safe and appropriate use of products, services, diagnostics and medicines.

This definition framework is summarised as:

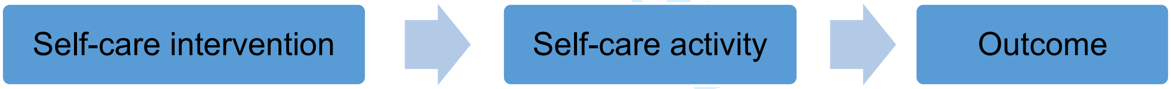

General principles

The following text considers several components of interventions that may have bearing on whether an intervention could be considered a self-care intervention or not.

1. Place of access

The self-care intervention might take place partially or fully outside formal health settings (e.g. outpatient services, clinics and hospitals). These places of access include, but are not limited to pharmacies, home, school and/or digital platforms.

2. Intersection with health systems

The intervention can be entirely initiated by the woman, or may involve some element of health worker (HW) support or engagement. In all instances, the woman must have the autonomy of her own self-care independent of a HW's presence.

(a) If the intervention relates to an informed action that can only be undertaken by the preconceiving, pregnant, labouring/birthing or postpartum woman (e.g. physical activity, daily dietary intake, etc.), it is considered self-care. Such an intervention may utilise

For peer review only

measures delivered by HW to facilitate or support the individual's self-care activity, for example by providing information, skills or tools.

> Example: Smoking cessation
>
> HW-led interventions aimed at promoting self-help for smoking cessation were eligible. These include HWs providing self-help materials (brochures, videos, online sources) or conducting educational sessions to give self-help instructions so that the woman can initiate smoking cessation at home.
>
> Interventions requiring intensive and regular contacts with physicians, therapists or nurses and without a fixed number of sessions were not considered self-care interventions. Interventions solely focusing on motivation enhancing e.g. motivational interviewing are not considered self-care interventions as these are not designed to develop skills and self-efficacy.

> Example: Acupuncture, transcutaneous electrical nerve stimulation (TENS), acupressure and massage
>
> Acupuncture is eligible if women are provided with and trained to operate a suitable device. TENS is eligible as TENS machines are easy to operate without prior training. Acupressure and massage, which do not require specific devices, are also eligible if administered by the woman or her partner.

> Example: Pain relief during labour
>
> Non-pharmacological therapies (e.g. aromatherapy, body massage, showering or water baths) are considered self-care if taught to the pregnant women or their caregivers during birth preparation workshops, or instructed to carry out at home, prior to admission. Studies where these interventions were administered by birth attendants or clinic staff are not eligible.

(b) If the intervention relates to care usually provided within the health system (e.g. testing, prescribing or administering medications), only those interventions where the woman self-manages some or all aspects of such care were considered as self-care. In studies, these interventions were usually compared against standard clinical care i.e. care delivered by physicians, nurses or community health workers.

> Example: Misoprostol for postpartum haemorrhage (PPH) prevention
>
> The administration of misoprostol by HWs (whether in facility or community settings) for PPH prevention is received by the pregnant woman - she does not self-administer. Therefore, it is not a self-care intervention, even if misoprostol administration takes place in the context of birth at home or in the community.
>
> Conversely, advanced distribution of misoprostol during antenatal visits to be used after childbirth, often accompanied by brief advice on how and when to use misoprostol safely, is considered a self-care intervention. This is because the intervention aims to enable the mother or her partners to self-administer misoprostol at home, when a skilled birth attendant, CHW or lay health worker is not available.

(c) Studies that investigated pharmacokinetics, efficacy and safety of pharmacological agents were excluded because their findings did not allow distinguishing between self-medication and being prescribed the medication by health providers. On the other hand, interventions aimed at promoting self-management or improving adherence to multi-dose therapies at home were eligible.

For peer review only

Example: Prophylactic agents for malaria and HIV prevention

Studies involving self-administration of IPTp and daily oral HIV PrEP at home (as opposed to supervised administration at antenatal clinics) are eligible. However, trials investigating pharmacokinetics, efficacy and safety of individual agents (sulfadoxine-pyrimethamine for malaria, tenofovir/emtricitabine for HIV) in pregnant and postpartum cohorts did not sufficiently show that these agents can be effectively implemented in a self-care approach, and were thus excluded.

3. Interventions beyond the pregnancy period

(a) Preconception studies

In our review, preconception interventions were eligible only if they were designed to improve pregnancy outcomes and reduce risks of maternal or perinatal complications. Interventions to improve the chance of successful conception (e.g. in couples undertaking *in vitro* fertilisation therapy) were excluded.

Interventions to manage existing medical conditions were excluded unless they were framed in the context of preparation for pregnancy and evaluated pregnancy-related outcomes.

Example: Type 1 diabetes mellitus (T1DM)

Uncontrolled glucose levels during pregnancy may increase risks of miscarriage or birth defects. Therefore, interventions aimed at achieving glycaemic control in women with T1DM who wish to get pregnant were eligible. However, general studies of self-care interventions among women with T1DM (not otherwise specified) were excluded.

(b) Postpartum studies

The postpartum period is defined as the first 42 days after birth. Interventions initiated outside this time frame were excluded.

Interventions designed to facilitate newborn care (i.e. infant nutrition, postnatal infant monitoring, kangaroo care, etc.) and breastfeeding (i.e. measures to increase lactation, breastfeeding techniques, etc.) were excluded from our review as their broad scope warrants a separate focused review.

4. Role of digital health

Digital interventions were possibly considered self-care interventions as they can provide platforms for the woman to seek information, communicate with health providers and access external support, ultimately creating a supporting environment for self-care. However, use of digital communication alone does not determine whether an intervention is self-care intervention or not, but rather what practice is facilitated by its use.

(a) Client-to-client technology

- Digital platforms to connect the pregnant or postpartum women to support groups (e.g. trained volunteers, other mothers) were considered self-care interventions. The underlying assumption is that when the women use these platforms, she is actively seeking information and support from sources outside the health system.

(b) Client-to-provider technology

- The use of digital trackers to enable the woman to take appropriate actions for self-management of her health conditions (e.g. taking her own physical measurements or self-adjusting medications based on these measurements) is a self-care intervention.

For peer review only

- If the digital trackers feed information directly to the clinician for decision-making, without involving the patient in the process, it is not a self-care intervention.

> Example: Blood glucose monitoring
>
> Interventions where women check their own blood glucose levels using a glucometer were self-care interventions.
>
> Interventions using automatic monitoring systems (i.e. sensors that automatically measure glucose levels in interstitial fluid and transmit readings to mobile devices) were evaluated on a case-by-case basis. If the woman was taught to adjust her lifestyle, calibrate her medications or notify her clinician of hypoglycaemic episodes, the intervention is a self-care intervention. If the data was masked to the participants during the study duration, the study was excluded.
>
> Interventions where blood glucose levels were tested by a health worker in inpatient or outpatient settings were not self-care interventions.

(c) Provider-to-client technology

- The use of mHealth/eHealth to provide self-help information, instructions or reminders to women to encourage adoption of a healthy lifestyle (or otherwise change behaviour) is a self-care intervention.
- The use of telehealth for virtual consults to replace face-to-face antenatal or postnatal visits is not a self-care intervention, because virtual delivery only replaces the mode of communication between the patient and the clinician, but still requires a health worker to provide effective care.

5. Mixed intervention

If an intervention consists of a mix of self-care and non-self-care interventions, the programme was evaluated on a case-by-case basis. The factors considered to judge eligibility are:

- Whether self-care components constitute a majority of the intervention
- Whether the intervention as a whole places an emphasis on self-care or explicitly promotes self-care
- Whether the intervention represents task-sharing certain aspects of clinical care to the women or their family members

For peer review only

Supplementary File S4. Data collection form

	Field	Format	Option/Example
**General characteristics**	Study ID	Free text	e.g. McManus_2018
	Reviewer's initial	Free text	
	Title	Free text	
	Trial register number (if any)	Free text	
**Linking**	ID of other reports from the same studies	Free text	
	Ongoing study	Y/N	Yes if the protocol was published as a journal article from 2019 onwards and trial register records did not indicate study completion
**Methods**	Study design	Multiple choice	O Meta-analysis and network meta-analysis<br>O Systematic review without meta-analysis<br>O Scoping review<br>O Overview of reviews<br>O Randomised controlled trial<br>O Non-randomised interventional study (including pretest-posttest, quasi-randomised trial, etc.)<br>O Retrospective & prospective observational cohort study<br>O Cross-sectional study<br>O Diagnostic test accuracy study
	Country	Free text	N/A for reviews and meta-analyses
**Participants**	<18 years of age	Y/N	Yes if the study inclusion criteria specified a minimum age <18 or adolescent girls

For peer review only

	Field	Format	Option/Example
	High-risk pregnancies	Y/N	Yes if the study inclusion criteria included: age >35 years old, presence of risk behaviours (substance use, smoking), obesity or overweight, pre-existing or pregnancy-induced chronic conditions (diabetes mellitus, hypertension, thyroid disease, or other co-morbidities), history of pregnancy complications or pregnancy loss, HIV-positive status, and multiple pregnancies
**Interventions**	Self-care activities	List any that is applicable	Refer to Supplementary File S5 for the full list of self-care activities
	Modes of access to and delivery of self-care interventions	List any that is applicable	▪ Health facilities e.g. self-diagnostic tests provided and taken in facilities, and information on and demonstration of self-care interventions provided in health facilities<br>▪ Community and non-facility-based healthcare e.g. products and services delivered to home by community health workers; activities taking place at community centres; traditional, complementary or alternative medicines<br>▪ Peer and/or partner counselling and support<br>▪ Over-the-counter availability of drugs and therapeutics, medical devices and diagnostics through pharmacies and drug stores<br>▪ Digital technologies and platforms e.g. telemedicine, individuals tracking their personal health data, eHealth including health information and peer support, ordering tests or drugs online<br>▪ Healthcare during emergency and humanitarian responses e.g. in shelters and refugee camps and settlements<br>▪ Home-based care i.e. direct access to health products, tools and information at home<br>▪ School: education, support and counselling taking place at school<br>▪ Workplace: health, safety and well-being interventions provided within the workplace, such as first aid andinterventions to support mental health and avoid burnout
	Time of intervention	List any that is applicable	▪ Preconception<br>▪ Antenatal<br>▪ Intrapartum<br>▪ Postpartum: Up to 42 days postpartum

For peer review only

	Field	Format	Option/Example
			▪ N/A for reviews and meta-analyses
	Any external support in the uptake or implementation of self-care	List any that is applicable	▪ None<br>▪ Family: partner & immediate family<br>▪ Community members: non-familial, non-medically trained community members, including peer support groups, friends, social leaders<br>▪ CHWs: informally-trained health workers such as traditional birth attendants or doulas<br>▪ HCWs: doctors, nurses, midwives, allied health practitioners, therapists and research students involved in delivering care.<br>HCWs that were only involved in facilitating study conduct (i.e. recruiting, explaining the study procedures, training participants on using the self-care tools etc.), without delivering actual medical care, were not considered as having supported delivery of the intervention.<br>▪ N/A for reviews and meta-analyses
**Outcomes**	Maternal mortality	Y/N	Maternal death
	Perinatal mortality	Y/N	Miscarriage, stillbirth, neonatal death
	Neonatal outcomes	Y/N	Birth weight, Apgar score, preterm birth, birth injuries, gestational age at delivery & other neonatal complications/outcomes, etc.
	Labour outcomes	Y/N	Duration of labour, labour pain, onset of labour (spontaneous, induced or pre-labour C-section); mode of delivery (spontaneous vaginal, instrumental – forceps/vacuum, C-section planned vs unplanned), etc.
	Anthropometric & cardiometabolic outcomes	Y/N	Maternal weight & BMI, lipid profile, inflammatory biomarkers, etc.
	Diabetes & other metabolic disorders	Y/N	GDM/T2DM diagnosis, various glycaemic biomarkers & serum indicators, etc.
	Hypertensive disorders of pregnancy	Y/N	Preeclampsia, eclampsia, gestational/postpartum hypertension, HELLP syndrome, blood pressure measurements etc.
	Postpartum infection & haemorrhage	Y/N	Postpartum haemorrhage, amount of blood loss, puerperal sepsis, endometritis, surgical site infection, etc.
	Urogenital outcomes	Y/N	Urinary incontinence, urinary tract infections, pelvic floor disorders, postpartum perineal issues etc.

For peer review only

	Field	Format	Option/Example
	Nutritional deficiencies	Y/N	Any nutritional deficiencies or serum levels of micronutrients, iron-deficiency anaemia, etc.
	Gastrointestinal outcomes	Y/N	Nausea & vomiting, constipation, etc.
	Other maternal complications	Y/N	Renal dysfunction, liver dysfunction & any other maternal complications not elsewhere classified
	Infectious diseases	Y/N	Malaria, HIV, STI, etc.
	Other minor physiological discomforts	Y/N	Breast & nipple issues, lumbopelvic pain & other minor ailments not elsewhere classified
	Mental wellbeing & quality of life	Y/N	Depression, anxiety, sexual functioning, childbirth fear/readiness
	Behavioural change	Y/N	Smoking cessation, alcohol use reduction, contraceptive use, supplement intake, physical activity & any other health-promoting or risk-avoiding behaviours
	Breastfeeding*	Y/N	Breastfeeding patterns (exclusive/full/partial), duration, initiation or continuation
	Other	Y/N	Diagnostic performance of tests, accordance with gold standards, intimate partner violence
**Self-care category (based on WHO classification [1])**	Individual Agency	List any that is applicable	▪ Interventions to promote awareness about self-care<br>▪ Interventions to promote autonomy and/or confidence to engage in self-care<br>▪ Interventions for improving self-care capacities and capabilities<br>▪ Interventions for improving health and digital literacy<br>▪ Interventions to promote sustained adoption of self-care practices and behaviours
	Health information-seeking	List any that is applicable	▪ Acquiring health education for informed health decision-making<br>▪ Improving health literacy to support health decision-making<br>▪ Individuals seeking health information via on-demand information services<br>▪ Individuals health decision-making support via online or mobile tools
	Social and community support	List any that is applicable	▪ Peer mentorship and counselling<br>▪ Peer-to-peer action to support access to and the uptake and use of self-care interventions<br>▪ Individuals sharing health data with peers

For peer review only

	Field	Format	Option/Example
	Personal health tracking	List any that is applicable	▪ Home-based record for health and diagnostic data<br>▪ Self-monitoring of health<br>▪ Active data capture/documentation by self-care user<br>▪ Passive data capture/documentation by device<br>▪ Use of diagnostic device at community location for<br>▪ self-measuring health indicator<br>▪ Tracking rational and responsible self-use of medicines and therapeutics
	Self-diagnosis of health conditions	List any that is applicable	▪ Self-testing, including to inform prevention<br>▪ Self-examining for health conditions<br>▪ Self-collection of samples to send for external testing<br>▪ Using online symptom checkers and health information
	Self-management of health	List any that is applicable	▪ Self-care for long-term health conditions<br>▪ Self-care for short-term health conditions<br>▪ Self-medication/treatment without medical prescription including for side effects<br>▪ Self-regulation of health conditions<br>▪ Self-care prevention including risk avoidance and support for physical and mental health and well-being<br>▪ Health management performed by caregivers<br>▪ Individuals procurement of safe over-the-counter medicines and products for self-care
	Individuals linkage to health system	List any that is applicable	▪ Individuals-initiated communication with health system<br>▪ Individuals sharing health data with health workers<br>▪ Individuals identifying location of health facilities/structures<br>▪ Individuals receiving feedback from health workers
	Individuals financial transactions for health	-	Not eligible under this review

**Although breastfeeding interventions were not eligible for inclusion, breastfeeding outcomes were recorded as they were often evaluated in interventions targeting other health issues, such as smoking cessation, diabetes management, breast & nipple care, etc.*

[1] World Health Organization. Classification of self-care interventions for health: a shared language to describe the uses of self-care interventions. Geneva, 2021 https://www.who.int/publications/i/item/9789240039469 (accessed April 6, 2022).

For peer review only

Supplementary File S5. List of excluded full texts

Author/Year	Title	Reasons for exclusion
Abbaspoor_2020	The effect of education through mobile phone short message service on promoting self-care in pre-diabetic pregnant women: A randomized controlled trial	Comparison - no appropriate control
Akanbi_2005	Effect of self-medication with antimalarial drugs on malaria infection in pregnant women in south-western Nigeria	Comparison - no appropriate control
Alemi_1996	Electronic self-help and support groups	Comparison - no appropriate control
Barbato_2014	Web-Based Treatment for Women With Stress Urinary Incontinence	Comparison - no appropriate control
Bradizza_2017	Smoking Cessation for Pregnant Smokers: Development and Pilot Test of an Emotion Regulation Treatment Supplement to Standard Smoking Cessation for Negative Affect Smokers	Comparison - no appropriate control
Burgess_2021	Feasibility of Using Blood Pressure Self-Monitoring and the Epic MyChart Blood Pressure Flowsheet to Monitor Blood Pressure after Preeclampsia	Comparison - no appropriate control
Bussiere-Cote_2019	Twice-Daily vs 4-Times-Daily Glucose Testing in Women With Gestational Diabetes Mellitus: a Pilot Study	Comparison - no appropriate control
Cao_2012	Comprehensive intensive therapy for Chinese gestational diabetes benefits both newborns and mothers	Comparison - no appropriate control
Chi_2016	Randomized comparison of group versus individual educational interventions for pregnant women to reduce their secondhand smoke exposure	Comparison - no appropriate control
Chung_2009	Validation and compliance of a home monitoring device in pregnancy: Microlife WatchBP home	Comparison - no appropriate control
Clapp_1989	The effects of maternal exercise on early pregnancy outcome	Comparison - no appropriate control
Cuneo_2017	Heart sounds at home: feasibility of an ambulatory fetal heart rhythm surveillance program for anti-SSA-positive pregnancies	Comparison - no appropriate control
Darvall_2020	A Pedometer-Guided Physical Activity Intervention for Obese Pregnant Women (the Fit MUM Study): randomized Feasibility Study	Comparison - no appropriate control
Dettinger_2019	PrEP Implementation for Mothers in Antenatal Care (PrIMA): Study protocol of a cluster randomised trial	Comparison - no appropriate control
Dyson_1998	Monitoring women at risk for preterm labor	Comparison - no appropriate control
Ganapathy_2016	Remote monitoring of blood pressure to reduce the risk of preeclampsia related complications with an innovative use of mobile technology	Comparison - no appropriate control
Gao_2012	Effects of an interpersonal-psychotherapy-oriented childbirth education programme for Chinese first-time childbearing women at 3-month follow up: randomised controlled trial	Comparison - no appropriate control
Gao_2021	An psychoeducation programme based on self-efficacy theory to improve experience of natural birth for primigravid women: Study protocol for a randomised controlled trial	Comparison - no appropriate control
Ghasemi_2021	Comparing the effect of individual counseling with counseling on social application on self-care and quality of life of women with gestational diabetes	Comparison - no appropriate control
Heuvel_2019	SAFE@HOME - Feasibility study of a telemonitoring platform combining blood pressure and preeclampsia symptoms in pregnancy care	Comparison - no appropriate control
Heuvel_2019	Validation of the iHealth Track and Omron HEM-9210T automated blood pressure devices for use in pregnancy	Comparison - no appropriate control
Hoppe_2016	An evaluation of diabetes targeted apps for Android smartphone in relation to behaviour change techniques	Comparison - no appropriate control
Humphries_2021	Decision Analysis in SHared decision making for Thromboprophylaxis during Pregnancy (DASH-TOP): a sequential explanatory mixed methods pilot study protocol	Comparison - no appropriate control
Iwama_2016	Maternal clinic and home blood pressure measurements during pregnancy and infant birth weight: The BOSHI study	Comparison - no appropriate control
Jiao_2019	Web-based versus home-based postnatal psychoeducational interventions for first-time mothers: A randomised controlled trial	Comparison - no appropriate control
Johnson_2018	Impacting diabetes self-management in women with gestational diabetes mellitus using short messaging reminders	Comparison - no appropriate control
Kerner_2004	Maternal self-administered fetal heart rate monitoring and transmission from home in high-risk pregnancies	Comparison - no appropriate control
Kominiarek_2019	A feasibility study of activity tracking devices in pregnancy	Comparison - no appropriate control
Lan_2017	Home blood pressure measurement in women with pregnancy-related hypertensive disorders	Comparison - no appropriate control
Lihme_2017	Self-monitoring of blood pressure during pregnancy: An observational study in the obstetrical waiting room	Comparison - no appropriate control
Limruangrong_2011	Effectiveness of a self-regulation program on diet control, exercise, and two-hour postprandial blood glucose levels in Thais with gestational diabetes mellitus	Comparison - no appropriate control
Malm_2014	Women's experiences of two different self-assessment methods for monitoring fetal movements in full-term pregnancy - a crossover trial	Comparison - no appropriate control
Manfredi_2011	Dissemination strategies to improve implementation of the PHS smoking cessation guideline in MCH public health clinics: experimental evaluation results and contextual factors	Comparison - no appropriate control

Author/Year	Title	Reasons for exclusion
Mantzana_2014	Applicability of self-obtained urine and vaginal samples for HPV-16, -18, -31 and -45 cervical cancer screening in pregnancy: A pilot cross-sectional study	Comparison - no appropriate control
Mendez-Figueroa_2017	Gestational Diabetes Mellitus and Frequency of Blood Glucose Monitoring: A Randomized Controlled Trial	Comparison - no appropriate control
Mikami_2017	Provisional criteria for the diagnosis of hypertension in pregnancy using home blood pressure measurements	Comparison - no appropriate control
Navaee_2020	Effect of pre-cesarean foot reflexology massage on anxiety of primiparous women	Comparison - no appropriate control
Pauley_2018	Gestational Weight Gain Intervention Impacts Determinants of Healthy Eating and Exercise in Overweight/Obese Pregnant Women	Comparison - no appropriate control
Pyra_2018	Tenofovir and tenofovir-diphosphate concentrations during pregnancy among HIV-uninfected women using oral preexposure prophylaxis	Comparison - no appropriate control
Raynes-Greenow_2009	Do audio-guided decision aids improve outcomes? A randomized controlled trial of an audio-guided decision aid compared with a booklet decision aid for Australian women considering labour analgesia	Comparison - no appropriate control
Roske_2008	Postpartum smoking cessation and relapse prevention intervention: A structural equation modeling application to behavioral and non-behavioral outcomes of a randomized controlled trial	Comparison - no appropriate control
Saffari_2020	Effect of a health-education program using motivational interviewing on oral health behavior and self-efficacy in pregnant women: a randomized controlled trial	Comparison - no appropriate control
Salomon_2004	Fetal home monitoring for the prenatal management of gastroschisis	Comparison - no appropriate control
Shieh_2017	Feasibility and Potential Benefits of a Self-Monitoring Enhanced Lifestyle Intervention to Prevent Excessive Gestational Weight Gain in Women Who Are Overweight or Obese	Comparison - no appropriate control
Smith_2014	Advance distribution of misoprostol for prevention of postpartum hemorrhage (PPH) at home births in two districts of Liberia	Comparison - no appropriate control
Svensson_2009	Randomised-controlled trial of two antenatal education programmes	Comparison - no appropriate control
Takeuchi_2016	Randomised controlled trial using smartphone website vs leaflet to support antenatal perineal massage practice for pregnant women	Comparison - no appropriate control
Tremonti_2017	Reliability of home blood pressure monitoring devices in pregnancy	Comparison - no appropriate control
Tucker_2018	How do home and clinic blood pressure readings compare in pregnancy? A systematic review and individual patient data meta-analysis	Comparison - no appropriate control
Twedt_2015	Sleep Duration and Blood Glucose Control in Women With Gestational Diabetes Mellitus	Comparison - no appropriate control
Ussher_2017	The London Exercise And Pregnant smokers (LEAP) trial: a randomised controlled trial of physical activity for smoking cessation in pregnancy with an economic evaluation	Comparison - no appropriate control
Vakilian_2018	Controlled breathing with or without lavender aromatherapy for labor pain at the first stage: a randomized clinical trial	Comparison - no appropriate control
Valbo_1991	Smoking cessation in pregnancy Mode of intervention and effect	Comparison - no appropriate control
Valiani_2014	Comparison of childbirth training workshop effects on knowledge, attitude, and delivery method between mothers and couples groups referring to Isfahan health centers in Iran	Comparison - no appropriate control
Vallely_2016	Feasibility and acceptability of clean birth kits containing misoprostol for self-administration to prevent postpartum hemorrhage in rural Papua New Guinea	Comparison - no appropriate control
Waugh_2003	Patient initiated home blood pressure recordings are accurate in hypertensive pregnant women	Comparison - no appropriate control
Wu_2021	Application of diversified and quantitative management model of exercise intervention in patients with gestational diabetes mellitus	Comparison - no appropriate control
Young_2000	The effectiveness of weekly iron supplementation in pregnant women of rural northern Malawi	Comparison - no appropriate control
Chen_2020	Non-pharmacological interventions for postpartum depression: A protocol for systematic review and network meta-analysis	Design - not a systematic review
Chow_2021	Appraisal of systematic reviews on interventions for postpartum depression: systematic review	Design - not a systematic review
Cottrell_2010	An updated review of of evidence to discourage douching	Design - not a systematic review
Davies_2018	Global and national guidance for the use of pre-exposure prophylaxis during peri-conception, pregnancy and breastfeeding	Design - not a systematic review
Dennis_2014	Psychosocial interventions for the treatment of perinatal depression	Design - not a systematic review
Devoe_2000	Home uterine activity monitoring: A critical review	Design - not a systematic review
Dominguez-Solis_2021	Non-pharmacological interventions to reduce anxiety in pregnancy, labour and postpartum: A systematic review	Design - not a systematic review
Edmonds_2014	Shared decision-making and decision support: Their role in obstetrics and gynecology	Design - not a systematic review
Evans_2018	Systematic review and meta-analysis of non-pharmacological interventions to reduce the symptoms of mild to moderate anxiety in pregnant women	Design - not a systematic review

Author/Year	Title	Reasons for exclusion
Feig_2018	Continuous glucose monitoring in pregnant women with Type 1 diabetes: benefits for mothers, using pumps or pens, and their babies	Design - not a systematic review
Flash_2017	Pre-exposure prophylaxis for HIV prevention in women: Current perspectives	Design - not a systematic review
Grange_2020	Smoking and smoking cessation in pregnancy Synthesis of a systematic review	Design - not a systematic review
Greaves_2019	Tailored Intervention for Smoking Reduction and Cessation for Young and Socially Disadvantaged Women During Pregnancy	Design - not a systematic review
Horgan_2019	Pre-exposure prophylaxis for HIV prevention during pregnancy and lactation: forget not the women and children	Design - not a systematic review
Hoyme_2010	Prevention of Preterm Birth Is Possible by Vaginal pH Screening, Early Diagnosis of Bacterial Vaginosis or Abnormal Vaginal Flora and Treatment	Design - not a systematic review
Joubert_2012	Personal continuous glucose monitoring (CGM) in diabetes management: Review of the literature and implementation for practical use	Design - not a systematic review
Jovanovic_2008	Using meal-based self-monitoring of blood glucose as a tool to improve outcomes in pregnancy complicated by diabetes	Design - not a systematic review
Koukoulithras_2021	The Effectiveness of Non-Pharmaceutical Interventions Upon Pregnancy-Related Low Back Pain: A Systematic Review and Meta-Analysis	Design - not a systematic review
Lambermon_2020	Maternal self-care in the early postpartum period: An integrative review	Design - not a systematic review
Melvin_2000	Recommended cessation counselling for pregnant women who smoke: A review of the evidence	Design - not a systematic review
Menendez_2007	Reducing the burden of malaria in pregnancy by preventive strategies	Design - not a systematic review
Montag_2012	A review of evidence-based approaches for reduction of alcohol consumption in Native women who are pregnant or of reproductive age	Design - not a systematic review
Moradi_2020	COVID-19 and self-care strategies for women with gestational diabetes mellitus	Design - not a systematic review
Nosova_2020	Leveraging technology for the treatment of type 1 diabetes in pregnancy: A review of past, current, and future therapeutic tools	Design - not a systematic review
Rodbard_2017	Continuous glucose monitoring: A review of recent studies demonstrating improved glycemic outcomes	Design - not a systematic review
Sachs_1991	Home monitoring of uterine activity Does it prevent prematurity?	Design - not a systematic review
Siebern_2012	Non-Pharmacological Treatment of Insomnia	Design - not a systematic review
Stalter_2021	Safety review of tenofovir disoproxil fumarate/emtricitabine pre-exposure prophylaxis for pregnant women at risk of HIV infection	Design - not a systematic review
Zinsser_2020	Changing behaviour in pregnant women: A scoping review	Design - not a systematic review
Cairns_2020	Implementing self-management: A mixed methods study of women's experiences of a postpartum hypertension intervention (SNAP-HT)	Design - qualitative study
Chiarelli_2003	Acceptability of a urinary continence promotion programme to women in postpartum	Design - qualitative study
Gosha_1986	A self-help group for new mothers: an evaluation	Design - qualitative study
Gueguen_2021	Hypnosis for labour and childbirth: A meta-integration of qualitative and quantitative studies	Design - qualitative study
Jidong_2021	Psychological interventions for maternal depression among women of African and Caribbean origin: a systematic review	Design - qualitative study
Labrecque_2001	Women's views on the practice of prenatal perineal massage	Design - qualitative study
Lee_2008	A pilot intervention for pregnant women in Sichuan, China on passive smoking	Design - qualitative study
McCaffrey_2020	The role and outcomes of music listening for women in childbirth: An integrative review	Design - qualitative study
Moulaei_2021	The Development and Usability Assessment of an mHealth Application to Encourage Self-Care in Pregnant Women against COVID-19	Design - qualitative study
Price_1991	Comparison of three antismoking interventions among pregnant women in an urban setting: A randomized trial	Design - qualitative study
Radestad_2021	Midwives' experiences of using the Mindfetalness method when talking with pregnant women about fetal movements	Design - qualitative study
Shorey_2019	A systematic mixed-studies review on mindfulness-based childbirth education programs and maternal outcomes	Design - qualitative study
Stonbraker_2020	Establishing content for a digital educational support group for new adolescent mothers in the Dominican Republic: A user-centered design approach	Design - qualitative study
Wallace_2009	A pregnancy wellness guide to enhance care through self-assessment, personal reflection, and self-referral	Design - qualitative study
Yoshida_2001	Postnatal depression in Japanese mothers and the reconsideration of 'Satogaeri bunben'	Design - qualitative study
Bivia-Roig_2020	Efficacy of an internet-based intervention to promote a healthy lifestyle on the reproductive parameters of overweight and obese women: study protocol for a randomised controlled trial	Intervention - conception/fertility
Boedt_2021	Systematic development of a mobile preconception lifestyle programme for couples undergoing IVF: the PreLiFe-programme	Intervention - conception/fertility

Author/Year	Title	Reasons for exclusion
Galletly_1996	Improved pregnancy rates for obese, infertile women following a group treatment program: An open pilot study	Intervention - conception/fertility
Kim_2020	Effectiveness of non-pharmacological interventions for overweight or obese infertile women: A systematic review and meta-analysis	Intervention - conception/fertility
Soltani_2015	Maternal obesity management using mobile technology: A feasibility study to evaluate a text messaging based complex intervention during pregnancy	Intervention - conception/fertility
Sridhar_2020	Non-pharmacological anxiety reduction with immersive virtual reality for first-trimester dilation and curettage: a pilot study	Intervention - conception/fertility
Taghavi_2021	Pharmacological and non?pharmacological strategies for obese women with subfertility	Intervention - conception/fertility
Ahn_2004	The Effects of a Home-Visiting Discharge Education on Maternal Self-esteem, Maternal Attachment, PostpartumDepression and Family Function in theMothers of NICU Infants	Intervention - newborn care
Da?li_2021	The effect of oxytocin massage and music on breast milk production and anxiety level of the mothers of premature infants who are in the neonatal intensive care unit: A self-controlled trial	Intervention - newborn care
Deave_2019	A study to evaluate the effectiveness of Best Beginnings' Baby Buddy phone app in England: a protocol paper	Intervention - newborn care
Dol_2021	Effectiveness of a Postpartum Text Message Program (Essential Coaching for Every Mother) on Maternal Psychosocial Outcomes: Protocol for a Randomized Controlled Trial	Intervention - newborn care
Jamalivand_2017	Comparing the effect of electronic software and training booklet on maternal self-confidence and awareness about newborn care: A randomized controlled clinical trial	Intervention - newborn care
Missler_2020	Effectiveness of a psycho-educational intervention for expecting parents to prevent postpartum parenting stress, depression and anxiety: a randomized controlled trial	Intervention - newborn care
Mohammadi_2018	The effect of stress inoculation training on breastfeeding self-efficacy and perceived stress of mothers with low birth weight infants: A clinical trial	Intervention - newborn care
Ngai_2009	The effects of a childbirth psychoeducation program on learned resourcefulness, maternal role competence and perinatal depression: a quasi-experiment	Intervention - newborn care
Ondersma_2018	Computer-delivered indirect screening and brief intervention for drug use in the perinatal period: A randomized trial	Intervention - newborn care
Shorey_2015	A randomized controlled trial of the effectiveness of a postnatal psychoeducation programme on self-efficacy, social support and postnatal depression among primiparas	Intervention - newborn care
Shorey_2017	Effectiveness of the 'Home-but not Alone' mobile health application educational programme on parental outcomes: a randomized controlled trial, study protocol	Intervention - newborn care
Allard_2015	Gestational Diabetes Mellitus Identification Based on Self-Monitoring of Blood Glucose	Intervention - no intervention identified
Barbosa_2016	Educational technologies to encourage (self) care in postpartum women	Intervention - no intervention identified
Barrera_2015	Online prevention of postpartum depression for Spanish- and English-speaking pregnant women: a pilot randomized controlled trial	Intervention - no intervention identified
Bessinger_2002	Substrate utilization and hormonal responses to moderate intensity exercise during pregnancy and after delivery	Intervention - no intervention identified
Black-Olien_1993	The effects of prenatal programs on postpartum emotional well-being	Intervention - no intervention identified
Carlson_2018	Antepartum Care of Women Who Are Obese During Pregnancy: Systematic Review of the Current Evidence	Intervention - no intervention identified
Chung_2020	Medical help-seeking strategies for perinatal women with obstetric and mental health problems and changes in medical decision making based on online health information: Path analysis	Intervention - no intervention identified
Clapp_2008	Long-term outcome after exercising throughout pregnancy: fitness and cardiovascular risk	Intervention - no intervention identified
Cosson_2017	Poor reliability and poor adherence to self-monitoring of blood glucose are common in women with gestational diabetes mellitus and may be associated with poor pregnancy outcomes	Intervention - no intervention identified
Dennis_2007	Psychosocial and psychological interventions for treating antenatal depression	Intervention - no intervention identified
Dennis-Tiwary_2017	Salutary effects of an attention bias modification mobile application on biobehavioral measures of stress and anxiety during pregnancy	Intervention - no intervention identified
Dol_2020	Impact of mobile health interventions during the perinatal period on maternal psychosocial outcomes: A systematic review	Intervention - no intervention identified
Ekelof_2021	Depressive symptoms postpartum is associated with physical activity level the year prior to giving birth - A retrospective observational study	Intervention - no intervention identified
Feng_2021	Psychological or educational eHealth interventions on depression, anxiety or stress following preterm birth: a systematic review	Intervention - no intervention identified
Gbagbo_2020	Implications of self-medication in pregnancy for Safe Motherhood and Sustainable Development Goal-3 in selected Ghanaian communities	Intervention - no intervention identified
Gribel_2020	Influence of non-pharmacological obstetric interventions on adverse outcomes of childbirth under regional analgesia	Intervention - no intervention identified
Hall_2011	The use of complementary and alternative medicine by pregnant women: a literature review	Intervention - no intervention identified
Hewage_2020	Barriers to Gestational Diabetes Management and Preferred Interventions for Women With Gestational Diabetes in Singapore: Mixed Methods Study	Intervention - no intervention identified

Author/Year	Title	Reasons for exclusion
Hong_2021	Perspectives on antenatal education associated with pregnancy outcomes: Systematic review and meta-analysis	Intervention - no intervention identified
Hoyme_2020	Reduced incidence of early preterm birth in the State of Thuringia following an intravaginal pH-self-monitoring screening program	Intervention - no intervention identified
Igwesi-Chidobe_2021	Community-based non-pharmacological interventions for improving pain, disability and quality of life in pregnant women with musculoskeletal conditions: Protocol for a systematic review with meta-analyses	Intervention - no intervention identified
Iyawa_2021	Mobile apps for self-management in pregnancy: a systematic review	Intervention - no intervention identified
Jung_2021	Psychosocial support interventions for women with gestational diabetes mellitus: a systematic review	Intervention - no intervention identified
Kim_2017	Effectiveness of Psychosocial and Educational Prenatal and Postnatal Care Interventions for Married Immigrant Women in Korea: Systematic Review and Meta-analysis	Intervention - no intervention identified
Lau_2016	Efficacy of Internet-Based Self-Monitoring Interventions on Maternal and Neonatal Outcomes in Perinatal Diabetic Women: A Systematic Review and Meta-Analysis	Intervention - no intervention identified
Leis_2009	A systematic review of home-based interventions to prevent and treat postpartum depression	Intervention - no intervention identified
Leventhal_1989	Active coping reduces reports of pain from childbirth	Intervention - no intervention identified
Lin_2018	Effectiveness of self-help psychological interventions for treating and preventing postpartum depression: a meta-analysis	Intervention - no intervention identified
Mackiewicz-Seghete_2020	Protocol for a mechanistic study of mindfulness based cognitive therapy during pregnancy	Intervention - no intervention identified
Manandhar_2004	Effect of a participatory intervention with women's groups on birth outcomes in Nepal: cluster-randomised controlled trial	Intervention - no intervention identified
Martin_2017	Adherence-Specific Social Support Enhances Adherence to Calcium Supplementation Regimens among Pregnant Women	Intervention - no intervention identified
Masoumi_2018	Effect of a sex education program on females' sexual satisfaction during pregnancy: a randomized clinical trial	Intervention - no intervention identified
Mekonnen_2021	Adherence to iron with folic acid supplementation among pregnant women attending antenatal care in public health centers in simada district, northwest ethiopia: using health belief model perspective	Intervention - no intervention identified
Mertens_2019	Effect of Lifestyle Coaching Including Telemonitoring and Telecoaching on Gestational Weight Gain and Postnatal Weight Loss: A Systematic Review	Intervention - no intervention identified
Mohamadirizi_2018	The effect of religious-spiritual support on childbirth self-efficacy	Intervention - no intervention identified
Nadal_2021	Educational films for improving screening and self-management of gestational diabetes in India and Uganda (GUIDES): study protocol for a cluster-randomised controlled trial	Intervention - no intervention identified
Naughton_2015	Use and effectiveness of lapse prevention strategies among pregnant smokers	Intervention - no intervention identified
Navidian_2017	The effect of home-based supportive-educational counseling on primigravidas' postpartum stress	Intervention - no intervention identified
Nazik_2013	The Prevention And Reduction Of Postpartum Complications: Orem's Model	Intervention - no intervention identified
Newham_2012	State-trait anxiety inventory (STAI) scores during pregnancy following intervention with complementary therapies	Intervention - no intervention identified
Notley_2019	Development of a complex intervention for the maintenance of postpartum smoking abstinence: process for defining evidence-based intervention	Intervention - no intervention identified
O'Connell_2021	Interventions for fear of childbirth including tocophobia	Intervention - no intervention identified
Ogrodniczuk_2003	Preventing Postnatal Depression: a Review of Research Findings	Intervention - no intervention identified
Olander_2015	Person-centred care in interventions to limit weight gain in pregnant women with obesity - a systematic review	Intervention - no intervention identified
Olayiwola_2013	Living Smart, Living Fit: a patient-centered program to improve perinatal outcomes in a community health center population	Intervention - no intervention identified
Ouedraogo_2013	Maternal anemia in pregnancy: Assessing the effect of routine preventive measures in a malaria-endemic area	Intervention - no intervention identified
Ratnayake_2018	Validity of over the counter finger stick glucose measurement devices in comparison with laboratory venous plasma glucose measurements on pregnant women with diabetes	Intervention - no intervention identified
Sayakhot_2016	Use of a web-based educational intervention to improve knowledge of healthy diet and lifestyle in women with Gestational Diabetes Mellitus compared to standard clinic-based education	Intervention - no intervention identified
Silverstein_2011	Problem-solving education to prevent depression among low-income mothers of preterm infants: a randomized controlled pilot trial	Intervention - no intervention identified
Sinclair_2017	A systematic literature review of computer-based behavioural change interventions to inform the design of an online VBAC intervention for the OptiBIRTH European randomised trial (project HEALTH -- F3 -- 2012-305208)	Intervention - no intervention identified
Song_2015	A systematic review of psychosocial interventions for women with postpartum stress	Intervention - no intervention identified
Sun_2020	A mushroom diet reduced the risk of pregnancy-induced hyper tension and macrosomia: A randomized clinical trial	Intervention - no intervention identified

Author/Year	Title	Reasons for exclusion
Westerhoff_2019	About:blank? Online Interventions for Postpartum Depression	Intervention - no intervention identified
Windsor_2016	A Process Evaluation of the WV Smoking Cessation and Reduction in Pregnancy Treatment (SCRIPT) Dissemination Initiative: Assessing the Fidelity and Impact of Delivery for State-Wide, Home-Based Healthy Start Services	Intervention - no intervention identified
Wulp_2016	Correlates of partner support to abstain from prenatal alcohol use: a cross-sectional survey among Dutch partners of pregnant women	Intervention - no intervention identified
Wylie_2018	Evaluation of patient- versus provider-collected vaginal swabs for microbiome analysis during pregnancy	Intervention - no intervention identified
Abbas_2020	Testing a home-based model of care using misoprostol for prevention and treatment of postpartum hemorrhage: Results from a randomized placebo-controlled trial conducted in Badakhshan province, Afghanistan	Intervention - not self-care
Abdollahi_2020	Effect of Psychotherapy on Reduction of Fear of Childbirth and Pregnancy Stress: A Randomized Controlled Trial	Intervention - not self-care
Abuogi_2020	HIV status disclosure patterns and male partner reactions among pregnant women with HIV on lifelong ART in Western Kenya	Intervention - not self-care
Afandi_2019	The value of Continuous Glucose Monitoring and Self-Monitoring of Blood Glucose in patients with Gestational Diabetes Mellitus during Ramadan fasting	Intervention - not self-care
Agarwal_2012	Evaluation of isosorbide mononitrate for cervical ripening prior to induction of labor for postdated pregnancy in an outpatient setting	Intervention - not self-care
Ahmad_2021	The Knowledge of Danger Signs of Obstetric Complications among Women in Rural India: Evaluating an Integrated Microfinance and Health Literacy Program	Intervention - not self-care
Akbarzadeh_2015	Comparison of the Effects of Maternal Supportive Care and Acupressure (at BL32 Acupoint) on Labor Length and Infant's Apgar Score	Intervention - not self-care
Akpan_2018	Antenatal Deworming and Materno-Perinatal Outcomes in Calabar, Nigeria	Intervention - not self-care
Alfirevic_2020	Home versus inpatient induction of labour for improving birth outcomes	Intervention - not self-care
Alimoradi_2019	Comparing the effect of auricular acupressure and body acupressure on pain and duration of the first stage of labor: study protocol for a randomized controlled trial	Intervention - not self-care
Amanak_2019	The impact of prenatal education based on the Roy adaptation model on gestational hypertension, adaptation to pregnancy and pregnancy outcomes	Intervention - not self-care
Anderson_2021	Effects of a Home-Based Intervention on HIV Prevention Health Behaviors in Pregnant/Postpartum Kenyan Women: Estimating Moderating Effects of Depressive Symptoms	Intervention - not self-care
Aquino_2020	Exploring the Use of Telemonitoring for Patients at High Risk for Hypertensive Disorders of Pregnancy in the Antepartum and Postpartum Periods: scoping Review	Intervention - not self-care
Ayerle_2018	Effects of the birthing room environment on vaginal births and client-centred outcomes for women at term planning a vaginal birth: BE-UP, a multicentre randomised controlled trial	Intervention - not self-care
Bain_2014	Interventions for managing asthma in pregnancy	Intervention - not self-care
Baljon_2020	Effectiveness of breathing exercises, foot reflexology and back massage (BRM) on labour pain, anxiety, duration, satisfaction, stress hormones and newborn outcomes among primigravidae during the first stage of labour in Saudi Arabia: a study protocol for	Intervention - not self-care
Bar-Zeev_2019	Improving smoking cessation care in pregnancy at Aboriginal Medical Services: 'ICAN QUIT in Pregnancy' step-wedge cluster randomised study	Intervention - not self-care
Batool_2018	Comparison of the efficacy of IV iron versus oral iron therapy in Postpartum Anemia	Intervention - not self-care
Beckmann_1996	Accuracy of maternal perception of preterm uterine activity	Intervention - not self-care
Beiranvand_2014	The effects of religion and spirituality on postoperative pain, hemodynamic functioning and anxiety after cesarean section	Intervention - not self-care
Bhandal_2006	Intravenous versus oral iron therapy for postpartum anaemia	Intervention - not self-care
Birdsall_2002	Benefits of monitoring blood glucose by collection of blood on filter paper strips	Intervention - not self-care
Bishop_2016	Evaluating acupuncture and standard care for pregnant women with back pain (ease back): a pilot randomised trial	Intervention - not self-care
Bolinder_2016	Novel glucose-sensing technology and hypoglycaemia in type 1 diabetes: a multicentre, non-masked, randomised controlled trial	Intervention - not self-care
Bollapragada_2006	IMOP: Randomised placebo controlled trial of outpatient cervical ripening with isosorbide mononitrate (IMN) prior to induction of labour - Clinical trial with analyses of efficacy, cost effectiveness and acceptability	Intervention - not self-care
Bollapragada_2009	Randomized placebo-controlled trial of outpatient (at Home) cervical ripening with isosorbide mononitrate (IMN) prior to induction of labor-clinical trial with analyses of efficacy and acceptability: the IMOP study	Intervention - not self-care
Brown_1999	A randomized comparison of home uterine activity monitoring in the outpatient management of women treated for preterm labor	Intervention - not self-care
Bryce_2009	CATCH: development of a home-based midwifery intervention to support young pregnant smokers to quit	Intervention - not self-care
Bullarbo_2007	Outpatient vaginal administration of the nitric oxide donor isosorbide mononitrate for cervical ripening and labor induction postterm: a randomized controlled study	Intervention - not self-care
Bullock_2009	Baby BEEP: a randomized controlled trial of nurses' individualized social support for poor rural pregnant smokers	Intervention - not self-care
ButlerTobah_2019	Randomized comparison of a reduced-visit prenatal care model enhanced with remote monitoring	Intervention - not self-care
Carter_2020	Pilot Randomized Controlled Trial of Diabetes Group Prenatal Care	Intervention - not self-care
Catling-Paull_2011	Non-clinical interventions that increase the uptake and success of vaginal birth after caesarean section: a systematic review	Intervention - not self-care

Author/Year	Title	Reasons for exclusion
Chen_2003	Continuous glucose monitoring for the evaluation and improved control of gestational diabetes mellitus	Intervention - not self-care
Choko_2017	Investigating interventions to increase uptake of HIV testing and linkage into care or prevention for male partners of pregnant women in antenatal clinics in Blantyre, Malawi: Study protocol for a cluster randomised trial	Intervention - not self-care
Choko_2019	HIV self-testing alone or with additional interventions, including financial incentives, and linkage to care or prevention among male partners of antenatal care clinic attendees in Malawi: An adaptive multi-arm, multi-stage cluster randomised trial	Intervention - not self-care
Choko_2021	Partner-delivered HIV self-test kits with and without financial incentives in antenatal care and index patients with HIV in Malawi: a three-arm, cluster-randomised controlled trial	Intervention - not self-care
CHUMS-Group_1995	A multicenter randomized controlled trial of home uterine monitoring: active versus sham device The Collaborative Home Uterine Monitoring Study (CHUMS) Group	Intervention - not self-care
Chung_2012	Randomized non-invasive sham-controlled pilot trial of electroacupuncture for postpartum depression	Intervention - not self-care
Clarke_2020	OptiBIRTH: a cluster randomised trial of a complex intervention to increase vaginal birth after caesarean section	Intervention - not self-care
Colton_1995	A metaanalysis of home uterine activity monitoring	Intervention - not self-care
Corey_2019	Bedside Music Therapy for Women during Antepartum and Postpartum Hospitalization	Intervention - not self-care
Corna_2019	Supporting maternal mental health of Rohingya refugee women during the perinatal period to promote child health and wellbeing: A field study in Cox's Bazar	Intervention - not self-care
Corwin_1996	Multicenter randomized clinical trial of home uterine activity monitoring: Pregnancy outcomes for all women randomized	Intervention - not self-care
Cottrell_2006	Vaginal douching practices of women in eight Florida panhandle counties	Intervention - not self-care
Cummins_2016	Telephone Intervention for Pregnant Smokers: A Randomized Controlled Trial	Intervention - not self-care
Cypryk_2006	Evaluation of metabolic control in women with gestational diabetes mellitus by the continuous glucose monitoring system: a pilot study	Intervention - not self-care
Davey_2020	Emerging evidence from a systematic review of safety of pre-exposure prophylaxis for pregnant and postpartum women: where are we now and where are we heading?	Intervention - not self-care
Dellicour_2016	Effectiveness of the delivery of interventions to prevent malaria in pregnancy in Kenya	Intervention - not self-care
Desai_2018	Prevention versus treatment for malaria in pregnant women	Intervention - not self-care
deSousa_2019	Building trust to childbirth: Assessment of a nursing intervention programme	Intervention - not self-care
Dolatian_2010	The effect of reflexology on pain intensity of labor	Intervention - not self-care
Dowswell_2009	Transcutaneous electrical nerve stimulation (TENS) for pain management in labour	Intervention - not self-care
Doyle_2015	Home based educational intervention to improve perinatal outcomes for a disadvantaged community: a randomised control trial	Intervention - not self-care
El-Khouly_2017	Comparison of intravenous ferrous sucrose and oral ferrous sulphate in treatment of postpartum iron deficiency anemia	Intervention - not self-care
Evans_2021	A systematic review of supportive interventions to promote women's comfort and well-being during induction of labour	Intervention - not self-care
Facco_2019	Can We Use Home Sleep Testing for the Evaluation of Sleep Apnea in Obese Pregnant Women?	Intervention - not self-care
Fantinelli_2019	Assessment of psychological dimensions in telemedicine care for gestational diabetes mellitus: A systematic review of qualitative and quantitative studies	Intervention - not self-care
Fealy_2019	The Support for New Mums Project: A protocol for a pilot randomized controlled trial designed to test a postnatal psychoeducation smartphone application	Intervention - not self-care
Fenwick_2013	Study protocol for reducing childbirth fear: a midwife-led psycho-education intervention	Intervention - not self-care
Ferrara_2016	The Comparative Effectiveness of Diabetes Prevention Strategies to Reduce Postpartum Weight Retention in Women With Gestational Diabetes Mellitus: The Gestational Diabetes' Effects on Moms (GEM) Cluster Randomized Controlled Trial	Intervention - not self-care
Field_2013	Peer support and interpersonal psychotherapy groups experienced decreased prenatal depression, anxiety and cortisol	Intervention - not self-care
Firouzan_2020	The effect of midwifery led counseling based on Gamble's approach on childbirth fear and self-efficacy in nulligravida women	Intervention - not self-care
Ford_2001	Effects of a prenatal care intervention on the self-concept and self-efficacy of adolescent mothers	Intervention - not self-care
Ford_2002	Effects of a prenatal care intervention for adolescent mothers on birth weight, repeat pregnancy, and educational outcomes at one year postpartum	Intervention - not self-care
Foster_2016	Evaluating Acupuncture and Standard carE for pregnant women with Back pain (EASE Back): A feasibility study and pilot randomised trial	Intervention - not self-care
Fottrel_2013	The effect of increased coverage of participatory women's groups on neonatal mortality in Bangladesh: A cluster randomized trial	Intervention - not self-care
Fraser_1997	Randomized controlled trial of a prenatal vaginal birth after cesarean section education and support program Childbirth Alternatives Post-Cesarean Study Group	Intervention - not self-care
Garg_2015	A comparative study to evaluate the efficacy and safety of single dose intravenous iron carboxymaltose vs multidose iron sucrose in postpartum cases of severe iron deficiency anemia	Intervention - not self-care
Given_2013	Comparing patient-generated blood glucose diary records with meter memory in diabetes: a systematic review	Intervention - not self-care
Given_2015	Tele-Mum: A Feasibility Study for a Randomized Controlled Trial Exploring the Potential for Telemedicine in the Diabetes Care of Those with Gestational Diabetes	Intervention - not self-care
Grady_2004	Pregnancy outcomes of adolescents enrolled in a CenteringPregnancy program	Intervention - not self-care

Author/Year	Title	Reasons for exclusion
Green_1992	Telephone fetal heart rate monitoring in South Australia	Intervention - not self-care
Greve_2020	A pragmatic real-life study of flash glucose monitoring versus self-monitoring of blood glucose	Intervention - not self-care
Griffiths_2021	Interventions for preventing nausea and vomiting in women undergoing regional anaesthesia for caesarean section	Intervention - not self-care
Guittier_2009	Moxibustion for breech version: a randomized controlled trial	Intervention - not self-care
Guo_2019	Evaluating the effects of mobile health intervention on weight management, glycemic control and pregnancy outcomes in patients with gestational diabetes mellitus	Intervention - not self-care
Gutman_2020	A cluster randomized trial of delivery of intermittent preventive treatment of malaria in pregnancy at the community level in Burkina Faso	Intervention - not self-care
Gyorkos_2006	Lack of risk of adverse birth outcomes after deworming in pregnant women	Intervention - not self-care
Habib_2008	Outpatient cervical ripening with nitric oxide donor isosorbide mononitrate prior to induction of labor	Intervention - not self-care
Hajesmaeel-Gohari_2021	Virtual reality applications to assist pregnant women: a scoping review	Intervention - not self-care
Hanjani_2015	The Effect of Foot Reflexology on Anxiety, Pain, and Outcomes of the Labor in Primigravida Women	Intervention - not self-care
Haq_2005	Control of postpartum and post abortal haemorrhage with uterine packing	Intervention - not self-care
Heazell_2017	Can promoting awareness of fetal movements and focusing interventions reduce fetal mortality? A stepped-wedge cluster randomised trial (AFFIRM)	Intervention - not self-care
Hedderson_2018	A Tailored Letter Based on Electronic Health Record Data Improves Gestational Weight Gain Among Women With Gestational Diabetes Mellitus: The Gestational Diabetes' Effects on Moms (GEM) Cluster-Randomized Controlled Trial	Intervention - not self-care
Heuvel_2018	eHealth as the Next-Generation Perinatal Care: An Overview of the Literature	Intervention - not self-care
Hickling_1990	A non-pharmacological treatment of vascular headache during pregnancy	Intervention - not self-care
Holm_2019	Automated blood pressure self-measurement station compared to office blood pressure measurement for first trimester screening of pre-eclampsia	Intervention - not self-care
Holt_2017	Improving help-seeking for postnatal depression and anxiety: a cluster randomised controlled trial of motivational interviewing	Intervention - not self-care
Homko_2007	Use of an internet-based telemedicine system to manage underserved women with gestational diabetes mellitus	Intervention - not self-care
Homko_2012	Impact of a telemedicine system with automated reminders on outcomes in women with gestational diabetes mellitus	Intervention - not self-care
Horey_2004	Information for pregnant women about caesarean birth	Intervention - not self-care
Hughes_2018	Prediabetes in pregnancy, can early intervention improve outcomes? A feasibility study for a parallel randomised clinical trial	Intervention - not self-care
Ickovics_2011	Effects of group prenatal care on psychosocial risk in pregnancy: Results from a randomised controlled trial	Intervention - not self-care
Imhoff-Kunsch_2012	Antihelminthics in pregnancy and maternal, newborn and child health	Intervention - not self-care
Ivan_2015	Effect of Deworming on Disease Progression Markers in HIV-1-Infected Pregnant Women on Antiretroviral Therapy: A Longitudinal Observational Study From Rwanda	Intervention - not self-care
Izadirad_2018	Improving prenatal care in pregnant women in Iranshahr, Iran: Applying Health Belief Model	Intervention - not self-care
Jankowski_1987	Self-administered medications for obstetric patients	Intervention - not self-care
Jijimole_2018	Comparison of the effect of control, presence of a skilled birth attendant and reflexotherapy on labour outcomes in terms of pain, duration of labour and birth satisfaction among primigravid women-a pilot study	Intervention - not self-care
Johnsen_2018	Using eHealth to Increase Autonomy Supportive Care: a Multicenter Intervention Study in Antenatal Care	Intervention - not self-care
Johnson_2020	Computerized intervention for reducing intimate partner victimization for perinatal women seeking mental health treatment: A multisite randomized clinical trial protocol	Intervention - not self-care
Karaçam_2012	The Use of Perineal Massage in the Second Stage of Labor and Follow-Up of Postpartum Perineal Outcomes	Intervention - not self-care
Kellie_2020	Mechanical and surgical interventions for treating primary postpartum haemorrhage	Intervention - not self-care
Kelman_2018	A proof?of?concept pilot randomized comparative trial of brief Internet?based compassionate mind training and cognitive?behavioral therapy for perinatal and intending to become pregnant women	Intervention - not self-care
Kempe_1998	Home uterine activity monitoring in the prevention of very low birth weight reprinted from Public Health Reports, September/October 1997, Volume 112, published by the US Public Health Service	Intervention - not self-care
Kim_2019	Effects of a Web-Based Self-Management Program on the Behavior and Blood Glucose Levels of Women with Gestational Diabetes Mellitus	Intervention - not self-care
Krakowiak_2016	Home-Based HIV Testing Among Pregnant Couples Increases Partner Testing and Identification of Serodiscordant Partnerships	Intervention - not self-care
Kuo_2016	Auricular acupressure relieves anxiety and fatigue, and reduces cortisol levels in post-caesarean section women: a single-blind, randomised controlled study	Intervention - not self-care
Lane_2019	Real-Time Continuous Glucose Monitoring in Gestational Diabetes: A Randomized Controlled Trial	Intervention - not self-care
Larocque_2006	Should deworming be included in antenatal packages in hookworm-endemic areas of developing countries?	Intervention - not self-care
Larsen_2012	The Effectiveness of Childbirth Classes for Increasing Self-Efficacy in Women and Support Persons	Intervention - not self-care
Lathrop_2014	A pilot study of prenatal care visits blended group and individual for women with low income	Intervention - not self-care
Li_2020	Internet-based intervention for postpartum depression in China ("Mommy go"): Protocol for a randomized controlled trial	Intervention - not self-care

Author/Year	Title	Reasons for exclusion
Loayza_2011	Biofeedback for pain management during labour	Intervention - not self-care
Lumley_2006	Aiming to increase birth weight: a randomised trial of pre-pregnancy information, advice and counselling in inner-urban Melbourne	Intervention - not self-care
Luoba_2005	Earth-eating and reinfection with intestinal helminths among pregnant and lactating women in western Kenya	Intervention - not self-care
Ma_2011	Effects of Sanyinjiao (SP6) with electroacupuncture on labour pain in women during labour	Intervention - not self-care
Majewska_2021	Flash glucose monitoring in gestational diabetes mellitus: study protocol for a randomised controlled trial	Intervention - not self-care
Maloni_2010	Antepartum bed rest for pregnancy complications: Efficacy and safety for preventing preterm birth	Intervention - not self-care
Mark_2019	Male Partner Linkage to Clinic-Based Services for Sexually Transmitted Infections and Human Immunodeficiency Virus Services Following Couple Home-Based Education and Testing	Intervention - not self-care
Martin_2015	Providing Information and Support to Postnatal Women Who Have Experienced a Cesarean Section: A Pilot Study	Intervention - not self-care
Marwa_2019	The effects of HIV self-testing kits in increasing uptake of male partner testing among pregnant women attending antenatal clinics in Kenya: A randomized controlled trial	Intervention - not self-care
Mburu_2018	A model for predicting utilization of mHealth interventions in low-resource settings: case of maternal and newborn care in Kenya	Intervention - not self-care
McKellar_2017	Capture my mood: a feasibility study to develop a visual scale for women to self-monitor their mental wellbeing following birth	Intervention - not self-care
Meher_2005	Bed rest with or without hospitalisation for hypertension during pregnancy	Intervention - not self-care
Mendelson_2013	Impact of a preventive intervention for perinatal depression on mood regulation, social support, and coping	Intervention - not self-care
Mhajna_2020	Wireless, remote solution for home fetal and maternal heart rate monitoring	Intervention - not self-care
Milani_2015	The effects of postpartum home care on constipation and hemorrhoids at sixty days postpartum	Intervention - not self-care
Miller_2021	Reproducing fear: the effect of birth stories on nulligravid women's birth preferences	Intervention - not self-care
Mofid_2017	A Double-Blind Randomized Controlled Trial of Maternal Postpartum Deworming to Improve Infant Weight Gain in the Peruvian Amazon	Intervention - not self-care
Mohebbi_2019	Self-management intervention program based on the health belief model (Hbm) among women with gestational diabetes mellitus: A quazi-experimental study	Intervention - not self-care
Momenyan_2021	Immersive virtual reality analgesia in un-medicated laboring women (during stage 1 and 2): a randomized controlled trial	Intervention - not self-care
Monincx_2001	Maternal health, antenatal and at 8 weeks after delivery, in home versus in-hospital fetal monitoring in high-risk pregnancies	Intervention - not self-care
MooreSimas_2018	A Systematic Review of Integrated Care Interventions Addressing Perinatal Depression Care in Ambulatory Obstetric Care Settings	Intervention - not self-care
Morrison_2005	Women's health groups to improve perinatal care in rural Nepal	Intervention - not self-care
Mourad-Youssif_2010	Can the Non-pneumatic Anti-Shock Garment (NASG) reduce adverse maternal outcomes from postpartum hemorrhage? Evidence from Egypt and Nigeria	Intervention - not self-care
Msyamboza_2009	Community-based distribution of sulfadoxine-pyrimethamine for intermittent preventive treatment of malaria during pregnancy improved coverage but reduced antenatal attendance in southern Malawi	Intervention - not self-care
Mundell_2011	The impact of structured support groups for pregnant South African women recently diagnosed HIV positive	Intervention - not self-care
Nasso_2018	The Gestational Diabetes Group Program	Intervention - not self-care
Navaee_2015	Effect of role play education on primiparous women's fear of natural delivery and their decision on the mode of delivery	Intervention - not self-care
Ndibazza_2010	Effects of Deworming during Pregnancy on Maternal and Perinatal Outcomes in Entebbe, Uganda: A Randomized Controlled Trial	Intervention - not self-care
Ndibazza_2015	Effects of Deworming during Pregnancy on Maternal and Perinatal Outcomes in Entebbe, Uganda: A Randomized Controlled Trial	Intervention - not self-care
Nezamnia_2020	Effectiveness of cognitive-behavioral therapy on sexual function and sexual self-efficacy in pregnant women: An RCT	Intervention - not self-care
Norman_2018	Awareness of fetal movements and care package to reduce fetal mortality (AFFIRM): a stepped wedge, cluster-randomised trial	Intervention - not self-care
Ojengbede_2011	Assessing the role of the non-pneumatic anti-shock garment in reducing mortality from postpartum hemorrhage in Nigeria	Intervention - not self-care
Ondersma_2007	Computer-based brief intervention a randomized trial with postpartum women	Intervention - not self-care
Ondersma_2015	Computer-Delivered Screening and Brief Intervention forAlcohol Use in Pregnancy: A Pilot Randomized Trial	Intervention - not self-care
Orobaton_2016	Scaling-up the use of sulfadoxine-pyrimethamine for the preventive treatment of malaria in pregnancy: results and lessons on scalability, costs and programme impact from three local government areas in Sokoto State, Nigeria	Intervention - not self-care
Osoti_2015	Home-based HIV testing for men preferred over clinic-based testing by pregnant women and their male partners, a nested cross-sectional study	Intervention - not self-care
Osterman_2012	Effects of a motivational interviewing intervention to decrease prenatal alcohol use	Intervention - not self-care
Palmer_2017	A retrospective cohort study of hospital versus home care for pregnant women with preterm prelabor rupture of membranes	Intervention - not self-care
Pantoja_2015	Oxytocin for preventing postpartum haemorrhage (PPH) in non-facility birth settings	Intervention - not self-care

Author/Year	Title	Reasons for exclusion
Penn_2018	Non?pneumatic anti?shock garment (NASG) as a first aid for preventing or reversing hypovolemic shock secondary to obstetric hemorrhage	Intervention - not self-care
Perichart-Perera_2009	A medical nutrition therapy program improves perinatal outcomes in Mexican pregnant women with gestational diabetes and type 2 diabetes mellitus	Intervention - not self-care
Pikee_2021	New Innovation: Use of Flash Glucose Monitoring for Evaluating Glycaemic Variability, Patient Satisfaction and Clinical Utility in Pregnant Women with Diabetes	Intervention - not self-care
Raams_2018	Task shifting in active management of the third stage of labor: A systematic review	Intervention - not self-care
Redulla_2018	Bed rest with and without hospitalization in multiple pregnancy for improving perinatal outcomes	Intervention - not self-care
Reid_2002	A two-centred pragmatic randomised controlled trial of two interventions of postnatal support	Intervention - not self-care
Runjati_2020	The effect of postpartum coping skill classes (PCSC) on stress level, cortisol levels, maternal self-efficacy, and baby's growth and development In Semarang, Central Java	Intervention - not self-care
Ryo_2012	Fetal movement counting at home with a fetal movement acceleration measurement recorder: A preliminary report	Intervention - not self-care
Salam_2021	Effect of mass deworming with antihelminthics for soil?transmitted helminths during pregnancy	Intervention - not self-care
Sandeford_2021	Protocol for probiotic therapy vs placebo for preterm prelabour rupture of membranes to prolong pregnancy duration (Pro-PPROM) trial	Intervention - not self-care
Sathe_2016	Procedures and Uterine-Sparing Surgeries for Managing Postpartum Hemorrhage: A Systematic Review	Intervention - not self-care
Schachman_2004	Baby boot camp: facilitating maternal role adaptation among military wives	Intervention - not self-care
Scott_2018	Accuracy, User Acceptability, and Safety Evaluation for the FreeStyle Libre Flash Glucose Monitoring System When Used by Pregnant Women with Diabetes	Intervention - not self-care
Sekhavatpour_2020	The effect of spiritual self-care training on the quality of life of mothers of preterm infants: A randomized controlled trial	Intervention - not self-care
Senoun_2014	Planned home versus hospital care for preterm prelabour rupture of the membranes (PPROM) prior to 37 weeks' gestation	Intervention - not self-care
Shah_1993	Evaluation of the home-based maternal record: a WHO collaborative study	Intervention - not self-care
Shirvani_2021	Importance of childbirth preparation classes in improving childbirth self-efficacy and reducing worry in primigravidas	Intervention - not self-care
Shirzad_2020	Effect of "motivational interviewing" and "information, motivation, and behavioral skills" counseling interventions on choosing the mode of delivery in pregnant women: a study protocol for a randomized controlled trial	Intervention - not self-care
Snell_2006	An exploratory study in the UK of the effectiveness of three different pain management regimens for post-caesarean section women	Intervention - not self-care
Solhi_2019	Effect of Health Literacy Education on Self-Care in Pregnant Women: A Randomized Controlled Clinical Trial	Intervention - not self-care
Sosa_2001	Bed rest in singleton pregnancies for preventing preterm birth	Intervention - not self-care
Stamp_1995	Evaluation of antenatal and postnatal support to overcome postnatal depression: a randomized, controlled trial	Intervention - not self-care
Stock_2014	Home cervical ripening with dinoprostone gel in nulliparous women with singleton pregnancies	Intervention - not self-care
Stotts_2002	One-to-One A motivational intervention for resistant pregnant smokers	Intervention - not self-care
Stotts_2004	Impact of a motivational intervention on mechanisms of change in low-income pregnant smokers	Intervention - not self-care
Sultan_2019	Oral vs intravenous iron therapy for postpartum anemia: a systematic review and meta-analysis	Intervention - not self-care
Svahn-Ekdahl_2021	Maintenance of physical activity level, functioning and health after non-pharmacological treatment of pelvic girdle pain with either transcutaneous electrical nerve stimulation or acupuncture: A randomised controlled trial	Intervention - not self-care
Tandon_2014	Six-month outcomes from a randomized controlled trial to prevent perinatal depression in low-income home visiting clients	Intervention - not self-care
Tappin_2005	Randomised controlled trial of home based motivational interviewing by midwives to help pregnant smokers quit or cut down	Intervention - not self-care
Thayer_2017	Effects of deworming on child and maternal health: a literature review and meta-analysis	Intervention - not self-care
Thomas_2004	Effectiveness of a comprehensive psychoeducational intervention with pregnant and parenting adolescents: A pilot study	Intervention - not self-care
Thome_2013	Evaluation of a family nursing intervention for distressed pregnant women and their partners: A single group before and after study	Intervention - not self-care
Thomson_2016	Gestational Weight Gain: results from the Delta Healthy Sprouts Comparative Impact Trial	Intervention - not self-care
Toohill_2014	A randomized controlled trial of a psycho-education intervention by midwives in reducing childbirth fear in pregnant women	Intervention - not self-care
Torlesse_2001	Albendazole therapy and reduced decline in haemoglobin concentration during pregnancy (Sierra Leone)	Intervention - not self-care
Trevillion_2020	An exploratory parallel-group randomised controlled trial of antenatal Guided Self-Help (plus usual care) versus usual care alone for pregnant women with depression: DAWN trial	Intervention - not self-care
Triebwasser_2020	Successful implementation of text-based blood pressure monitoring for postpartum hypertension	Intervention - not self-care
Tripathy_2016	Effect of participatory women's groups facilitated by Accredited Social Health Activists on birth outcomes in rural eastern India: a cluster-randomised controlled trial	Intervention - not self-care
Troy_2003	The effectiveness of a self-care intervention for the management of postpartum fatigue	Intervention - not self-care
Turan_2018	Development and Piloting of a Home-Based Couples Intervention During Pregnancy and Postpartum in Southwestern Kenya	Intervention - not self-care

For peer review only

Author/Year	Title	Reasons for exclusion
Turawa_2020	Interventions for preventing postpartum constipation	Intervention - not self-care
Urquhart_2017	Home uterine monitoring for detecting preterm labour	Intervention - not self-care
van-der-Ploeg_1996	Transcutaneous nerve stimulation (TENS) during the first stage of labour: a randomized clinical trial	Intervention - not self-care
Varnfield_2021	MTHer, an mHealth System to Support Women with Gestational Diabetes Mellitus: Feasibility and Acceptability Study	Intervention - not self-care
Verma_2011	Intravenous iron therapy versus oral iron in postpartum patients in rural area	Intervention - not self-care
Voormolen_2018	Continuous glucose monitoring during diabetic pregnancy (GlucoMOMS): a multicentre randomized controlled trial	Intervention - not self-care
Walia_2020	Effect of sharing health messages on antenatal care behavior among women involved in microfinance-based self-help groups in Bihar India	Intervention - not self-care
Walia_2021	Routine deworming during antenatal care decreases risk of neonatal mortality and low birthweight: A retrospective cohort of survey data	Intervention - not self-care
Wapner_1995	A randomized multicenter trial assessing a home uterine activity monitoring device used in the absence of daily nursing contact	Intervention - not self-care
Watson_1990	Management of preterm labor patients at home: does daily uterine activity monitoring and nursing support make a difference?	Intervention - not self-care
Webb_2012	The effect of anthelmintic treatment during pregnancy on HIV plasma viral load: results from a randomized, double-blind, placebo-controlled trial in Uganda	Intervention - not self-care
Wei_2016	Effect of a CGMS and SMBG on Maternal and Neonatal Outcomes in Gestational Diabetes Mellitus: a Randomized Controlled Trial	Intervention - not self-care
Willcox_2011	Is parasite clearance clinically important after malaria treatment in a high transmission area? A 3-month follow-up of home-based management with herbal medicine or ACT	Intervention - not self-care
Williams_2012	Associations between preconception counseling and maternal behaviors before and during pregnancy	Intervention - not self-care
Witt_2016	Therapeutic Breast Massage in Lactation for the Management of Engorgement, Plugged Ducts, and Mastitis	Intervention - not self-care
Witteveen_2020	Effectiveness of a guided ACT-based self-help resilience training for depressive symptoms during pregnancy: Study protocol of a randomized controlled trial embedded in a prospective cohort	Intervention - not self-care
Wyck_2007	Intravenous Ferric Carboxymaltose Compared With Oral Iron in the Treatment of Postpartum Anemia: A Randomized Controlled Trial	Intervention - not self-care
Xiu_2020	Psychological intervention for patients with high-risk pregnancy can improve their self-management ability and pregnancy outcome	Intervention - not self-care
Yang_2018	Medical nutrition treatment of women with gestational diabetes mellitus by a telemedicine system based on smartphones	Intervention - not self-care
Yu_2019	Application and Utility of Continuous Glucose Monitoring in Pregnancy: A Systematic Review	Intervention - not self-care
Zaharieva_2020	Continuous Glucose Monitoring Versus Self-Monitoring of Blood Glucose to Assess Glycemia in Gestational Diabetes	Intervention - not self-care
Zaharieva_2021	Continuous Glucose Monitoring Versus Self-Monitoring of Blood Glucose to Assess Glycemia in Gestational Diabetes	Intervention - not self-care
Zandinava_2017	Effect of educational package on Self-Care behavior, quality of life, and blood glucose levels in pregnantwomen with gestational diabetes: A randomized controlled trial	Intervention - not self-care
Zhang_2021	The effect of individualized exercise prescriptions combined with dietary management on blood glucose in the second-and-third trimester of gestational diabetes mellitus	Intervention - not self-care
Abobaker_2020	Study the effect of non-pharmaceutical approach on traumatic nipple for lactating mothers	No full text available
Abraham-Justice_2010	The effects of a postpartum education program on self-care and healthcare-seeking behaviors in mothers	No full text available
Afshar_2012	Effect of a sex educational package on sexual function in pregnant women: a multicenter randomized controlled trial	No full text available
Agot_2018	Secondary distribution of HIV self-tests as a way to promote HIV testing among male partners of young women: subgroup analysis from a randomized trial	No full text available
Alipour_2017	The effectiveness of relaxation techniques on depression, anxiety and stress in pregnant women: based on self-efficacy theory	No full text available
Arpin_2007	Evaluation of pain relief as well as nurses' and mothers' satisfaction following the implementation of an automedication program in obstetrics	No full text available
Artal_1996	Home uterine activity monitoring and its role in reducing the incidence of prematurity	No full text available
Askari_2016	Effect of back massage with sesames oil on pain and length of delivery in primiparous women	No full text available
Asklund_2019	The use of an app with a pfmt programme among pregnant and postnatal women for preventive use and treatment of urinary incontinence	No full text available
Attanayake_2016	Cervical ripening with self administered iso sorbide mononitrate vaginally, in uncomplicated singleton pregnancies at 39 weeks gestation: a double blind randomised controlled trial	No full text available
BabaDizavandi_2012	The effect of music therapy on labour pain in nulliparous women in mashad, Iran in 2010	No full text available
Bahri_2015	Educational intervention to improve oral health beliefs and behaviors during pregnancy: a randomized-controlled trial	No full text available
Bais_2018	A randomized, double-blind controlled clinical trial of light therapy for pregnant women with major depressive disorder	No full text available
Barfoot_2015	A pilot randomised trial of 4 physiotherapy interventions for pregnancy related pelvic girdle pain	No full text available
Beeharry_2017	A multimedia approach to diabetes structured education: the effects of "Keeping Healthy After Gestational Diabetes (GDM)", a multimedia based education program on patient knowledge, self-efficacy and acceptability	No full text available

Author/Year	Title	Reasons for exclusion
Berry_2015	A Postpartum Community-Based Weight Management Intervention Designed for Low-Income Women: Feasibility and Initial Efficacy Testing	No full text available
Bertz_2010	Dietary restriction or dietary restriction and exercise, but not exercise intervention alone, reduces weight and fat mass in overweight and obese women postpartum	No full text available
Bjorn_2013	Relaxation strategies during pregnancy Health benefits	No full text available
Blondel_1992	Home uterine activity monitoring in France: a randomized, controlled trial	No full text available
Buhling_2004	Introductory experience with the continuous glucose monitoring system (CGMS; Medtronic Minimed) in detecting hyperglycemia by comparing the self-monitoring of blood glucose (SMBG) in non-pregnant women and in pregnant women with impaired glucose tolerance	No full text available
Bung_1993	[Regular exercise therapy in disorders of carbohydrate metabolism in pregnancy--results of a prospective, randomized longitudinal study]	No full text available
Burke_2019	Feasibility and acceptability of home use of a smartphone-based urine testing application among women in prenatal care	No full text available
Cairns_2017	A novel self-management intervention for adjustment of postnatal antihypertensive treatment	No full text available
Cairns_2017	Self-management of postnatal antihypertensive treatment: a pilot randomised controlled trial	No full text available
Carissoli_2017	Enhancing psychological wellbeing of women approaching the childbirth: A controlled study with a mobile application	No full text available
Cohn_2014	Mind in Labor: Effects of Mind/Body Training on Childbirth Appraisals and Pain Medication Use During Labor	No full text available
Das_2015	Effect of a behavioral intervention with cereal fiber or resistant starch on gestational weight gain: a randomized clinical trial	No full text available
DavimRMB_2008	Non pharmacological strategies evaluation in parturients' pain relief	No full text available
DavimRMB_2008	Showering as a non pharmacological strategy to relief the parturients pain	No full text available
Dyson_1988	The role of home uterine monitoring in the prevention of preterm birth in high risk patients	No full text available
Dyson_1990	Preterm birth prevention - the role of education - palpation vs home uterine monitoring in non-multiple gestations	No full text available
Dyson_1991	Prevention of preterm birth in high-risk patients: the role of education and provider contact versus home uterine monitoring	No full text available
El-Refaey_2020	The effect of tailored psycho-educational program on pregnant women's anxiety and knowledge about self-care management regarding minor discomforts	No full text available
Epp_2019	A Review of the Effects of Physical Therapy on Self-Esteem in Postpartum Women With Lumbopelvic Dysfunction	No full text available
Ershoff_1990	Pregnancy and medical cost outcomes of a self-help prenatal smoking cessation program in an HMO	No full text available
Ershoff_1995	Relapse prevention among women who stop smoking early in pregnancy: A randomized clinical trial of a self-help intervention	No full text available
Eva_2021	Virtual reality in obstetrics as a non pharmacological method	No full text available
Evans_2020	Non-pharmacological interventions to reduce the symptoms of mild to moderate anxiety in pregnant women A systematic review and narrative synthesis of women's views on the acceptability of and satisfaction with interventions	No full text available
Evans_2020	Developing a complex intervention to support pregnant women with mild to moderate anxiety: application of the Medical Research Council framework	No full text available
Evies_2007	Effects of education for self-care on the metabolic control of pregnant diabetic patients	No full text available
Fakhri_2017	Effect of education based on health belief model with relaxation on anxiety of nulliparouse women	No full text available
Faraji_2016	The impact of supplementation with fish oil on lipid profile of pregnant mothers: a randomized controlled trial	No full text available
Farajnejad_2020	Aromatherapy in postpartum depression: a clinical trial based on precede model	No full text available
Fawcett_1993	Effects of information on adaptation to cesarean birth	No full text available
Feingold_1993	Contribution of home uterine activity monitoring to the reduction of preterm delivery rate	No full text available
Frye_2015	On the trail of misoprostol in the community: a secondary analysis of self-administered misoprostol for the prevention of postpartum hemorrhage in Uganda	No full text available
Geelhoed-Duijvestijn_2016	Real-time continuous glucose monitoring during pregnancy in women with type 1 diabetes: is RTCGM related to better glycaemic control and key obstetric outcomes?	No full text available
Ghasemi_2018	The effect of group counseling with cognitive-behavioral approach on self-efficacy of pregnant women's choice of vaginal delivery	No full text available
Gookin_1994	Randomized comparison of home uterine activity monitoring and routine care in patients discharged after treatment for preterm labor	No full text available
Greene_1999	A postpartum self-medication program: Effect on narcotic use	No full text available
Grimes_1992	Randomized controlled trials of home uterine activity monitoring: a review and critique	No full text available
Guerra_2005	Educational for self care of pregnant diabetic patients	No full text available
Hannover_2004	Interventions to prevent health risks due to tobacco smoke in pregnant women, women postpartum and their infants	No full text available
Hanson_1984	Self-monitoring of blood glucose by diabetic women during the third trimester of pregnancy	No full text available
Hartmann_1996	A randomized controlled trial of smoking cessation intervention in pregnancy in an academic clinic	No full text available
Henrique_2018	Pain, anxiety, and stress during childbirth-management without medication	No full text available

Author/Year	Title	Reasons for exclusion
Hill_1990	Home uterine activity monitoring is associated with a reduction in preterm birth	No full text available
Hjalmarson_1991	Stopping smoking in pregnancy: effect of a self-help manual in controlled trial	No full text available
Hodnett_1999	Home?based social support for socially disadvantaged mothers	No full text available
Holden_2016	Yoga for pregnancy related back pain	No full text available
Hollingsworth_1987	Postprandial walking exercise in pregnant insulin-dependent (type I) diabetic women: reduction of plasma lipid levels but absence of a significant effect on glycemic control	No full text available
Horio_1998	Fetal monitor for non-stress-test screening at home	No full text available
Howell_2011	Mothers avoiding depression through empowerment intervention trial (made it)	No full text available
Hoyme_1998	Reduction of prematurity by pH-screening	No full text available
Hsieh_2017	Efficacy of Warm Showers on Postpartum Fatigue Among Vaginal-Birth Taiwanese Women: A Quasi-Experimental Design	No full text available
Huddle_1987	Diabetes in pregnancy: The use of home blood glucose monitoring and intensive monitoring to ensure favourable perinatal outcome	No full text available
Iams_1987	A prospective random trial of home uterine activity monitoring in pregnancies at increased risk of preterm labor	No full text available
Iams_1988	A prospective random trial of home uterine activity monitoring in pregnancies at increased risk of preterm labor Part II	No full text available
Iams_1992	Randomized controlled trials of home uterine activity monitoring: a review and critique	No full text available
Iams_1995	Current status of home uterine activity monitoring	No full text available
Igarashi_2010	Effects of aromatherapy for self-care during pregnancy	No full text available
Jack_2019	Effect of Addition of an Intimate Partner Violence Intervention to a Nurse Home Visitation Program on Maternal Quality of Life: a Randomized Clinical Trial	No full text available
Jaqin_2019	Differences in self-efficacy before and after antenatal education	No full text available
Jeon_2018	Effects of an educational program on knowledge and Efficacy of self-care during pregnancy among marriage immigrant women in South Korea	No full text available
Johnson_2000	Preventing smoking relapse in postpartum women	No full text available
Johnston_2006	The Community Perinatal Care Study: home visiting and nursing support for pregnant women	No full text available
Kaplan_2011	The impact of a behavioral intervention on weight gain during pregnancy	No full text available
Kavanagh_2020	Does an online wellbeing program (Baby Steps) prvent perinatal distress in first-time fathers and mothers?	No full text available
Kaveh_2012	Impact of Education on Nutrition and Exercise on the Level of Knowledge and Metabolic Control Indicators (FBS & PPBS) of Gestational Diabetes Mellitus (GDM) Patients	No full text available
Knuppel_1990	Preventing preterm birth in twin gestation: home uterine activity monitoring and perinatal nursing support	No full text available
Kyung_2003	Effects of postpartum care program for primiparous women and care-givers on the knowledge and behavior of postpartum care and postpartum recovery in primiparous women	No full text available
Lara_2010	Retention rates and potential predictors in a longitudinal randomized control trial to prevent postpartum depression	No full text available
Larsen_2001	A prospective study of self-efficacy expectancies and labour pain	No full text available
Lee_2018	Implementation and evaluation of gestational diabetes management using mobile health care service-a pilot study	No full text available
Lehugeur_2017	Non-Pharmacological Management Of Relief In Deliveries Assisted By An Obstetric Nurse	No full text available
Lillington_1995	Evaluation of a smoking cessation program for pregnant minority women	No full text available
Lipatov_2019	Possibility of non-pharmacological and pharmacological therapies for placental insufficiency	No full text available
Liu_2010	Effects of music therapy on labour pain and anxiety in Taiwanese first-time mothers	No full text available
Lombard_2011	A randomized controlled trial investigating self-weighing and the prevention of excess weight gain in early pregnancy	No full text available
Lowe_1998	Evaluation of antenatal smoking cessation programs for pregnant women	No full text available
Lumluk_2011	Effect of antenatal education for better self-correct diagnosis of true labor: a randomized control study	No full text available
Luo_2015	Influence of Orem self-care model on birth outcomes of gestational diabetes mellitus patients	No full text available
Luoto_2012	Physical activity and dietary counseling and supervised group exercise for first-time pregnant women - a feasibility study of a controlled trial	No full text available
Maloni_1998	Prescription of activity restriction to treat high-risk pregnancies	No full text available
Marquez-Sterling_2000	Physical and psychological changes with vigorous exercise in sedentary primigravidae	No full text available
Millan-Ferro_2017	Participation of latinos with type 2 diabetes in a culturally and linguistically oriented virtual diabetes self-care and education program	No full text available
Miller_2004	Improving the identification and treatment of postpartum depression in a managed care organization	No full text available
Mirzakhani_2015	The effect of exercise with special ball during pregnancy on duration of active phase of first stage of birth in nulliparous women	No full text available
Mohammadi_2021	The effect of pregnancy training classes based on bandura self-efficacy theory on postpartum depression and anxiety and type of delivery	No full text available
Morrison_2003	Current status of home uterine activity monitoring	No full text available
Mortazavi_2021	Effectiveness of solution-focused counseling therapy on pregnancy anxiety and fear of childbirth: A randomized clinical trial	No full text available
Mou_1991	Multicenter randomized clinical trial of home uterine activity monitoring for detection of preterm labor	No full text available

Author/Year	Title	Reasons for exclusion
Mullen_1999	Maternal smoking during pregnancy and evidence-based intervention to promote cessation	No full text available
Naef_1998	Home blood pressure monitoring for pregnant patients with hypertension	No full text available
Nagey_1993	Randomized comparison of home uterine activity monitoring and routine care in patients discharged after treatment for preterm labor	No full text available
Nehbandani_2018	Comparison the effects of aromatherapy with rose extract and lavender on the pain of the active phase of labor in primipara women	No full text available
Newman_1988	Randomized trial of home uterine activity monitoring	No full text available
Norheim_2001	Acupressure treatment of morning sickness in pregnancy A randomised, double-blind, placebo-controlled study	No full text available
O'Connor_1992	Effectiveness of a pregnancy smoking cessation program	No full text available
Pasinlioglu_2004	Health education for pregnant women: The role of background characteristics	No full text available
Pawalia_2018	Behavioral Intervention during Pregnancy for Preventing Abdominal Obesity and Pregnancy Complications in Indian Women: Protocol for a Randomised Controlled Trial	No full text available
Petersen_1992	Smoking reduction during pregnancy by a program of self-help and clinical support	No full text available
Pont_2019	Prenatal yoga program on primigravida 3rd trimester reducing complaints in the first stage and self-efficacy in the labor	No full text available
Pormosayebi_2018	The effect of training based on health belief model (HBM) in preventing exposure to polluted air in pregnant women	No full text available
Posadas_2014	Intake of vitamin C, probiotics, flavonoids and nutritional status in pregnant women with urinary tract infection	No full text available
Pratt_2016	Effect of a brief educational intervention concerning the human papillomavirus on the knowledge and attitudes of antenatal patients in New Providence, The Bahamas	No full text available
Ramezanpoor_2019	The effectiveness of an educational intervention based on social cognitive theory on fruit and vegetable intake in pregnant women	No full text available
Ratner_2000	Twelve-month follow-up of a smoking relapse prevention intervention for postpartum women	No full text available
Rees_1995	Effect of relaxation with guided imagery on anxiety, depression, and self-esteem in primiparas	No full text available
Reichmann_2009	Home uterine activity monitoring: an evidence review of its utility in multiple gestations	No full text available
Reyhani_2014	Investigating the effects of spiritual self-care training on psychological stress of mothers with preterm infants admitted in neonatal intensive care unit	No full text available
Rezaeean_2017	Application of Orem's theory for promotion of self-care behaviors of pregnant women at risk for preterm delivery: A clinical trial	No full text available
Sanyal_2019	Management of gestational diabetes in a resource-limited setting in India	No full text available
Seto_2019	The Accuracy of Self-Screening of Group B Streptococcus in Pregnant Women-A Randomized Crossover Study	No full text available
Shobeiri_2016	The effect of orem's self care model on control of preeclampsia in pregnant women: A randomized clinical trial	No full text available
Smith_1990	Reliability of compact electronic blood pressure monitors for hypertensive pregnant women	No full text available
Smith_1992	Patient's acceptance of monitoring fetal movement: A randomized comparison of charting techniques	No full text available
Smith_1993	Educational efforts in preventing preterm delivery among inner city adolescents	No full text available
Smith_1994	Reduction of preterm births in a low-income population utilizing home uterine activity monitoring	No full text available
Sunderji_1991	Earlier detection of preterm labor: multicenter prospective randomized clinical trial of home uterine activity monitoring	No full text available
Swaroopa_2017	Effect of antenatal education in improving maternal confidence and reducing anxiety about labor in primigravida women attending saveetha medical college and hospital,chennai,tamil nadu, india	No full text available
Taheri_2021	The effect of health belief-based education on physical activates of nulliparous women: a randomized control trial	No full text available
Tian_2013	Effects of birth ball exercises on labor pain and childbirth satisfaction	No full text available
Unger_2015	Mobile wach: developing and evaluating a humancomputer hybrid mobile messaging system for women's and children's health in Kenya	No full text available
Valbo_1994	Smoking cessation in pregnancy: The effect of self-help manuals	No full text available
Vasaiya_2020	Comparison between Effect of Foot Exercise and Warm Water Foot Soak on Foot Edema among Antenatal Women	No full text available
Wahyuni_2020	The effect of early mobilization towards self-care levels in post-sectio caesarea mother with self-care theory approach	No full text available
Wardani_2018	The effectiveness of acupressure at LI 4 and SP 6 point on uterine contraction in the first stage of labor on primiparous women	No full text available
Wilson_2020	Making BUMP work: intervention development in a large randomised controlled trial of blood pressure self-monitoring in pregnancy	No full text available
Wing_2010	Comparison of urinary cytokines after ingestion of cranberry juice cocktail in pregnant subjects: a pilot study	No full text available
Wirz-Justice_2011	A randomized, double-blind, placebo-controlled study of light therapy for antepartum depression	No full text available
Yildirim_2004	The effect of breathing and skin stimulation techniques on labour pain perception of Turkish women	No full text available
Zardosht_2021	Effect of chamomile oil on cesarean section pain in primiparous women: a randomized clinical trial	No full text available
Zeng_2008	A research on relationship between fetal growth restriction and vitamin nutritional status in late pregnancy	No full text available

Author/Year	Title	Reasons for exclusion
Ainscough_2016	Impact of an mHealth supported healthy lifestyle intervention on behavioural stage of change in overweight and obese pregnancy	Not a peer-reviewed journal article
Al-Hashmi_2017	The Effectiveness of a Health Education Intervention on Self-Efficacy for Adherence to Healthy Behaviors among Women with Gestational Diabetes	Not a peer-reviewed journal article
Andalib_2016	Evaluation of the effect of corrected nutrition on reduction of stress in gestational diabetes	Not a peer-reviewed journal article
Apay_2015	The Effect of the Care Given Using Orem's Self-Care Model on the Postpartum Self- Evaluation	Not a peer-reviewed journal article
Barrett_2017	Home Monitoring of Fasting and Postprandial Triglycerides in Late Pregnancy: A Pilot Study	Not a peer-reviewed journal article
Brixval_2016	Antenatal education in small classes may increase childbirth self-efficacy: Results from a Danish randomised trial	Not a peer-reviewed journal article
Buckingham-Schutt_2019	The Behavioral Wellness in Pregnancy Study: a theory-based multi-component intervention to promote appropriate weight gain and healthy lifestyle behaviors in previously sedentary pregnant women	Not a peer-reviewed journal article
Butler_2019	Assessing and managing risk online: a clinician's perspective on internet-delivered intervention, 'MUMentum', for perinatal distress, anxiety, and depression	Not a peer-reviewed journal article
Chappell_2021	Self-monitoring of blood pressure in women with pregnancy hypertension: the BUMP2 multicentre randomised controlled trial	Not a peer-reviewed journal article
Chaudhry_2020	Development and assessment of smartphone based intervention (TechMotherCare) for maternal depression in Pakistan	Not a peer-reviewed journal article
Cheung_2011	A pilot structured behavioural intervention trial to increase physical activity among women with recent gestational diabetes	Not a peer-reviewed journal article
Chiarelli_2002	A physiotherapist-led programme of postpartum pelvic floor exercises reduced urinary incontinence at 3 months	Not a peer-reviewed journal article
Dahlen_2011	Moxibustion for cephalic version: a feasibility study	Not a peer-reviewed journal article
Dal_2021	The effect of pregnancy yoga on breastfeeding self-efficacy and prenatal attachment: A semi-experimental study	Not a peer-reviewed journal article
Dias_2011	Effectiveness of perineal exercises in controlling urinary incontinence and improving pelvic floor muscle function during pregnancy	Not a peer-reviewed journal article
Draffin_2013	Gestational diabetes: things you need to know (but maybe don't): development of an educational DVD for women recently diagnosed with gestational diabetes	Not a peer-reviewed journal article
Durmaz_2016	The Self-Care Training is Increasing Body Image Perception Self- Esteem and Self-Care Abilities in Pregnant Adolescents: A Pretest- Posttest Quasi-Experimental Study	Not a peer-reviewed journal article
Ericksen_2020	Treatment for antenatal anxiety and depression with Beating the Blues before Birth BBB© positively impacts infant postnatal development at 9 months, a pilot RCT	Not a peer-reviewed journal article
ErtekinPinar_2016	Effects of coping with stress training on pregnant women's depression, stress and coping with stress levels	Not a peer-reviewed journal article
Fancourt_2017	Associations between singing to babies and symptoms of postnatal depression, wellbeing, self-esteem and mother-infant bond	Not a peer-reviewed journal article
Farrell_2013	The use of logs and forms for the tracking of RT-CGM devices in the conceptt trial	Not a peer-reviewed journal article
Fox_2021	Short term postpartum blood pressure management and long-term blood pressure control: a randomised controlled trial	Not a peer-reviewed journal article
Freeman_1986	Randomised trial of self hypnosis for analgesia in labour	Not a peer-reviewed journal article
Goldberg_1986	Gestational diabetes: impact of home glucose monitoring on neonatal birth weight	Not a peer-reviewed journal article
Gunther_2019	The effect of lifestyle counselling on dietary behavior of pregnant women - Secondary results of the randomised controlled GeliS trial	Not a peer-reviewed journal article
Hadar_2021	Mobile Self-Operated Home Ultrasound System for Remote Fetal Assessment During Pregnancy	Not a peer-reviewed journal article
Harrison_2014	How effective is self-weighing in the setting of a lifestyle intervention to reduce gestational weight gain and postpartum weight retention?	Not a peer-reviewed journal article
Herring_2017	Intervening during and after pregnancy to prevent weight retention among African American women	Not a peer-reviewed journal article
Hill_2012	Nutrient intake of pregnant women with type 1 diabetes in the DAPIT Trial; relationships with anthropometry and glycaemic control	Not a peer-reviewed journal article
Hoffmann_2020	Does an antenatal lifestyle intervention in routine care improve maternal and infant health outcomes in the first year postpartum-12 months follow-up of the cluster-randomised GeliS trial	Not a peer-reviewed journal article
Hoirisch-Clapauch_2016	A protocol combining daily walking and a lowglycemic index diet increases the rate of take-home babies in women with consecutive first-trimester miscarriages	Not a peer-reviewed journal article
Houshmandpour_2019	The effect of midwife-oriented group counseling, based on orem's model on self-care and empowerment, in primiparous women: A clinical trial	Not a peer-reviewed journal article
Howharn_2008	Effects of childbirth preparation classes on self-efficacy in coping with labor pain in Thai primiparas	Not a peer-reviewed journal article
Hoyme_2002	Results and potential consequences of the thuringia prematurity preventional campaign 2000	Not a peer-reviewed journal article

Author/Year	Title	Reasons for exclusion
Hunt_2018	Evaluation of a home-use oral glucose tolerance test kit for screening for gestational diabetes	Not a peer-reviewed journal article
Hutchesson_2019	Be Healthe for your Heart Study Protocol: preventing cardiovascular disease among women with a history of pre-eclampsia	Not a peer-reviewed journal article
Ip_2005	The effect of a theory-based intervention on promoting self-efficacy for childbirth among pregnant women in Hong Kong	Not a peer-reviewed journal article
Keytash_2015	Impact of preconception care on overweight/obese women: long-term effects of a behavioural change intervention	Not a peer-reviewed journal article
Kirop_2021	Feasibility and Acceptability of a Telephone Support Intervention During Early Postnatal Period Among Teenage Mothers in Western Kenya: A Pilot Randomised Controlled Trial	Not a peer-reviewed journal article
Kisa_2019	Repeat HIV testing of individuals with discrepant HIV self-test results in Central Uganda	Not a peer-reviewed journal article
Lee_2016	Did self-sampling improve the adherence to group B streptococci screening in pregnant women?	Not a peer-reviewed journal article
Lineberger_1987	Pregnant adolescents attending prenatal parent education classes: self-concept, anxiety and depression levels	Not a peer-reviewed journal article
Loukopoulou_2012	Urinary nicotine and cotinine as a method for assessing the effectiveness of an intervention to aid smoking cessation during pregnancy	Not a peer-reviewed journal article
Makvandi_2018	Effect of normal physiologic childbirth program in mother-friendly hospitals on duration of labor	Not a peer-reviewed journal article
Mayer_1990	A randomized evaluation of smoking cessation interventions for pregnant women at a WIC clinic	Not a peer-reviewed journal article
McGready_2001	A double-blind randomized therapeutic trial of insect repellents for the prevention of malaria in pregnancy	Not a peer-reviewed journal article
McManus_2017	The snap-HT trial: self-management of antihypertensive medication postpartum - Can women do it better?	Not a peer-reviewed journal article
McManus_2018	109 Using blood pressure selfmonitoring in pregnancy: A systematic review and individual patient data metaanalysis	Not a peer-reviewed journal article
Miquelutti_2012	Evaluation of the efficacy of an antenatal birth preparation program	Not a peer-reviewed journal article
Mirghafourvand_2019	Effectiveness of self-care training on pregnancy consequences in gestational diabetes: A randomized controlled clinical trial	Not a peer-reviewed journal article
Mollart_2019	Are women interested in Acupressure to increase spontaneous onset of labour?	Not a peer-reviewed journal article
Montazeri_2020	The effect of writing therapy on anxiety in pregnant women: a randomized controlled trial	Not a peer-reviewed journal article
Murphy_2016	Asthma self-management education and inhaled corticosteroid use during pregnancy and postpartum from 2004 to 2014	Not a peer-reviewed journal article
Neoh_2017	The conceptt-diet study: an analysis of diet and glycaemia in women with type 1 diabetes before and during pregnancy	Not a peer-reviewed journal article
Nguyen_2017	Feasibility and impacts of integrating nutrition interventions into an existing maternal, neonatal, and child health platform in Bangladesh	Not a peer-reviewed journal article
Nicklas_2016	Beta-testing a mobile health program designed to increase postpartum weight loss in women at elevated risk for cardiometabolic disease	Not a peer-reviewed journal article
Offiah_2020	A feasibility study on the prevention of post-partum stress urinary incontinence by midwife-delivered antenatal pelvic floor muscle training: a cluster randomised controlled trial	Not a peer-reviewed journal article
Parry_2017	Early vs Late wake therapy improves mood in antepartum vs Postpartum depression by differentially altering melatonin and sleep timing	Not a peer-reviewed journal article
Pintye_2019	Self-tests for at-home partner testing are acceptable & utilized among pregnant women	Not a peer-reviewed journal article
Porreco_2020	Fetal Movement Counting and Perinatal Mortality: A Systematic Review and Meta-analysis	Not a peer-reviewed journal article
Roche_2018	Analysis of comparable home/clinic blood pressure readings in the LEANBH (Learning to Evaluate and manage ANtenatal Blood pressure at Home) population	Not a peer-reviewed journal article
Rowe_2019	Online and mobile psychoeducation initiatives to prevent postnatal mental health problems: research and evaluation	Not a peer-reviewed journal article
Rubertsson_2020	A randomized controlled study during pregnacy comparing internetbased cognitive behavioral therapy and counseling by standard care for fear of birth	Not a peer-reviewed journal article
Ruggiero_1997	A stage-matched smoking cessation program for pregnant smokers	Not a peer-reviewed journal article
Sanavi_2014	Effective education to decrease elective caesarean section	Not a peer-reviewed journal article
Secher_2014	Real-time continuous glucose monitoring as a tool to prevent severe hypoglycaemia in selected pregnant women with Type 1 diabetes - an observational study	Not a peer-reviewed journal article
Slade_2019	Preventing post-traumatic stress disorder: the Stress and Wellbeing after Childbirth Study(STRAWB2)	Not a peer-reviewed journal article
Smith_2014	Investigation of a lifestyle change strategy for high-risk women with a history of gestational diabetes	Not a peer-reviewed journal article

Author/Year	Title	Reasons for exclusion
Smith_2014	The Blossom Project Online: Use of a behaviorally-based website to promote physical activity and prevent excessive gestational weight gain in previously sedentary pregnant women	Not a peer-reviewed journal article
Suhaimi_2016	A low-gi diet improves glucose self-monitoring in women with gestational diabetes mellitus	Not a peer-reviewed journal article
Tappin_2000	The effect of home-based motivational interviewing on the smoking behaviour of pregnant women: a pilot randomized controlled efficacy study	Not a peer-reviewed journal article
Tucker_2019	Self-monitoring of blood pressure during pregnancy: the BUMP trials	Not a peer-reviewed journal article
Varrassi_1989	Effects of physical activity on maternal plasma beta-endorphin levels and perception of labor pain	Not a peer-reviewed journal article
Wapner_1996	The CHUMS (Collaborative Home Uterine Monitoring Study) clinical trial - What does it really say?	Not a peer-reviewed journal article
Weeks_2013	Self-administration of misoprostol to prevent bleeding after homebirths in uganda: a pilot placebo-controlled, randomised trial	Not a peer-reviewed journal article
Wei_2013	Earlier management with continuous monitoring blood glucose decrease gestational weight gain in gestational diabetes mellitus: a randomised clinical trial	Not a peer-reviewed journal article
Wong_2015	A randomized controlled trial of an educational intervention to improve influenza vaccine uptake among pregnant women	Not a peer-reviewed journal article
Wroblewski_2004	Implementing a comprehensive postpartum depression support program	Not a peer-reviewed journal article
Wu_2018	The application of online pelvic floor training during the rehabilitation of postpartum pelvic floor	Not a peer-reviewed journal article
XiangHu_2020	Effects of exercise therapy for pregnancy-related low back pain and pelvic pain: A protocol for systematic review and meta-analysis	Not a peer-reviewed journal article
Yang_2018	Effectiveness of aerobic gymnastic exercise on stress, fatigue, and sleep quality during postpartum: a pilot randomized controlled trial	Not a peer-reviewed journal article
Yazdkhasti_2018	The effect of localized heat and cold therapy on pain intensity, duration of phases of labor, and birth outcomes among primiparous females: a randomized, controlled trial	Not a peer-reviewed journal article
Zlotnick_2001	Postpartum Depression in Women Receiving Public Assistance: Pilot Study of an Interpersonal-Therapy-Oriented Group Intervention	Not a peer-reviewed journal article
Ahn_2011	Effects of breast massage on breast pain, breast-milk sodium, and newborn suckling in early postpartum mothers	Not in English
Bakhteh_2018	Effect of cognitive behavioral therapy on self-efficacy in women with gestational diabetes: A randomized clinical trial	Not in English
Chae_2021	[Maternal Health Effects of Internet-Based Education Interventions during the Postpartum Period: A Systematic Review]	Not in English
deSouzaMelo_2020	Maternal and perinatal parameters after non-pharmacological interventions: a randomised, controlled clinical trial	Not in English
Gayeski_2010	Non-pharmacological approach to pain relief during labor as hard-light care technology: a systematic review	Not in English
Ghaderpanah_2017	The effect of 5A model on behavior change of physical activity in overweight pregnant women	Not in English
Ghazaie_2016	The effectiveness of cognitive-behavioral therapy on fear of childbirth, fear of pain, self-efficacy of childbirth and tendency to caesarean in nulliparous women	Not in English
Hanjani_2013	The effect of foot reflexology on pain intensity and duration of labor on primiparous	Not in English
Hung_2017	Non-pharmacological interventions for pregnancy-related sleep disturbances	Not in English
Imano_2013	Evaluation of program to improve mental and physical health	Not in English
Jeon_2018	Effects of a postnatal care program on self-efficacy, self-management, and glycemic control in women with gestational diabetes mellitus	Not in English
Khadivzadeh_2015	Effects of self-care education on perceived stress in women with gestational diabetes under insulin treatment	Not in English
Kharazi_2018	Effect of an educational intervention based on self-efficacy theory and health literacy strategies on pregnancy outcomes: A randomized clinical trial	Not in English
Khodaparast_2020	Examining the effect of educational intervention based on self-regulation model on perceived stress in women with gestational diabetes: A clinical trial study	Not in English
Khojasteh_2018	Comparison of in-person counseling and telephone support on delivery self-efficacy in primiparous women	Not in English
Kim_2014	Effects of an integrated self-management program on self-management, glycemic control, and maternal identity in women with gestational diabetes mellitus	Not in English
Kim_2021	Effects of nursing intervention programs for women with gestational diabetes: a systematic review of randomized controlled trials	Not in English
KimSue_2011	Effects of a Coaching-based Childbirth Program on Anxiety and Childbirth Self-efficacy among Primigravida Women	Not in English
Ko_2014	Effects of a coaching program on comprehensive lifestyle modification for women with gestational diabetes mellitus	Not in English
Lawani_2003	Effect of antenatal gymnastics on childbirth: a study on 50 sedentary women in the Republic of Benin during the second and third quarters of pregnancy	Not in English
Lee_2017	Development and Validation of a Postpartum Care Mobile Application for First-time Mothers	Not in English
Lee_2018	Effects of family centered postpartum care for postpartum depression among previously infertile couples	Not in English

For peer review only

Author/Year	Title	Reasons for exclusion
Liang_2019	Effectiveness of a Labor-Admission Education Program on Anxiety, Uncertainty, Locus of Labor Control, and Labor Outcomes in Pregnant Women	Not in English
Madady_2017	Effect of hot shower and intravenous injection of hyoscine on childbirth experience of nulliparous women: a randomized clinical trial	Not in English
Mafetoni_2014	NON-PHARMACOLOGICAL METHODS FOR PAIN RELIEF DURING LABOR: INTEGRATIVE REVIEW	Not in English
Makhouli_2018	Implementation of targeted education to promote normal vaginal delivery: a randomized controlled trial	Not in English
Manabe_2006	Implementation and evaluation of health training guidance for the purpose of improving self-care behavior among primiparae	Not in English
Mascarenhas_2019	Scientific evidence on non-pharmacological methods for relief of labor pain	Not in English
Mazloomy-Mahmoodabad_2020	The Effect of Nutrition Education on Gestational Weight Gain based on the Pender's Health Promotion Model: a randomized clinical trial study	Not in English
Mohamadkhani-Shahri_2017	Effect of massage aromatherapy with Lavender oil on pain intensity of active phase of labor in nulliparous women	Not in English
Mojahed_2018	The effect of motivational interviewing on self-efficacy to quit hookah smoking in pregnant women	Not in English
Moller_2003	Current aspects of the Thuringia prematurity preventional campaign 2000	Not in English
Niazi_2018	Comparison of the effects of purslane cream and Lanolin for treatment of breast fissure on lactation self-efficacy: A randomized clinical trial	Not in English
Ozgoli_2016	Comparison of peppermint and clove essential oil aroma on pain intensity and anxiety at first stage of labor	Not in English
Pour_2012	Comparison of effect of transcutaneous electrical nerve stimulation and acupressure in decreasing labor pain in primiparous women	Not in English
Rezaeian_2017	The relationship of prenatal Self-Care behaviors with stress, anxiety and depression in women at risk of preterm delivery	Not in English
Rostampour_2020	The effect of couples' supportive counseling on self-care behavior in women with insulin-treated gestational diabetes: A randomized clinical trial	Not in English
Safat_2018	Efficacy of cognitive behavioral therapy-based self-management intervention on labor outcome in nulliparous women	Not in English
Saling_2000	New method for the self assessment of the pH-value of the vaginal secretions by an indicator coated panty liner - First preliminary communication	Not in English
Saling_2001	A simple, efficient and inexpensive program for preventing prematurity	Not in English
Shim_2012	[Effects of a yoga-focused prenatal program on stress, anxiety, self confidence and labor pain in pregnant women with in vitro fertilization treatment]	Not in English
Shimada_2005	A randomized controlled trial on evaluating effectiveness of perineal massage during pregnancy in primiparous women	Not in English
Siegmund-Schultze_2005	pH self assessment to reduce the risk of preterm birth - A pilot study becomes a model project	Not in English
Wan_2016	Clinical observation of analgesia in delivery treated with acupoint pressure therapy	Not in English
Wang_2015	Effects of a Mobile Web-based Pregnancy Health Care Educational Program for Mothers at an Advanced Maternal Age	Not in English
Khunpradit_2011	Non-clinical interventions for reducing unnecessary caesarean section	Older versions of a Cochrane review
Lauzon_1998	Antenatal education for self?diagnosis of the onset of active labour at term	Older versions of a Cochrane review
Moy_2014	Techniques of monitoring blood glucose during pregnancy for women with pre-existing diabetes	Older versions of a Cochrane review
Moy_2017	Techniques of monitoring blood glucose during pregnancy for women with pre-existing diabetes	Older versions of a Cochrane review
Pintye_2018	Preexposure Prophylaxis for Human Immunodeficiency Virus (HIV) Prevention Among HIV-uninfected Pregnant Women: Estimated Coverage Using Risk-based Versus Regional Prevalence Approaches	Outcome - feasibility/acceptability only
Smith_2016	Programmes for advance distribution of misoprostol to prevent post-partum haemorrhage: A rapid literature review of factors affecting implementation	Outcome - feasibility/acceptability only
Steinberg_2021	Optimizing Engagement in an mHealth Intervention for Diabetes Support During Pregnancy: the Role of Baseline Patient Health and Behavioral Characteristics	Outcome - feasibility/acceptability only
Aba_2017	Antenatal Education on Pregnant Adolescents in Turkey: Prenatal Adaptation, Postpartum Adaptation, and Newborn Perceptions	Outcome - no health or behavioural outcomes
Aveyard_2006	A randomized controlled trial of smoking cessation for pregnant women to test the effect of a transtheoretical model-based intervention on movement in stage and interaction with baseline stage	Outcome - no health or behavioural outcomes
Bastani_2010	Impact of preconception health education on health locus of control and self-efficacy in women	Outcome - no health or behavioural outcomes
Carolan-Olah_2016	Educational and intervention programmes for gestational diabetes mellitus (GDM) management: An integrative review	Outcome - no health or behavioural outcomes
Chang_2004	Effect of Taegyo-focused prenatal education on maternal-fetal attachment and self-efficacy related to childbirth	Outcome - no health or behavioural outcomes
Chen_2021	The accuracy and influencing factors for preference of self-sampling in group B streptococcus screening: a cross-sectional study	Outcome - no health or behavioural outcomes
Chillrud_2021	The effect of clean cooking interventions on mother and child personal exposure to air pollution: results from the Ghana Randomized Air Pollution and Health Study (GRAPHS)	Outcome - no health or behavioural outcomes

Author/Year	Title	Reasons for exclusion
Clarke_2005	Alternative site self blood glucose testing is preferred by women with gestational diabetes	Outcome - no health or behavioural outcomes
Conde_2019	Effect of Electronic Video Education on Patient's Self-Assessed Knowledge About Obesity and Pelvic Floor Disorders: A Randomized Controlled Trial	Outcome - no health or behavioural outcomes
Damodaran_2015	Effect of Progressive Muscle Relaxation Technique in Terms oi Anxiety and Physiological Parameters of Antenatal Mothers with Pregnancy-Induced Hypertension	Outcome - no health or behavioural outcomes
Dean_2012	A pilot study using interactive SMS support groups to prevent mother-to-child HIV transmission in South Africa	Outcome - no health or behavioural outcomes
Dickerson_2010	Pregnancy and village outreach tibet: a descriptive report of a community- and home-based maternal-newborn outreach program in rural Tibet	Outcome - no health or behavioural outcomes
Elsinga_2008	The effect of preconception counselling on lifestyle and other behaviour before and during pregnancy	Outcome - no health or behavioural outcomes
Evans_2014	Initial outcomes from a 4-week follow-up study of the Text4baby program in the military women's population: randomized controlled trial	Outcome - no health or behavioural outcomes
Grym_2019	Feasibility of smart wristbands for continuous monitoring during pregnancy and one month after birth 11 Medical and Health Sciences 1117 Public Health and Health Services	Outcome - no health or behavioural outcomes
Haakstad_2018	How does a lifestyle intervention during pregnancy influence perceived barriers to leisure-time physical activity? The Norwegian fit for delivery study, a randomized controlled trial	Outcome - no health or behavioural outcomes
Harris_2015	A Pilot Study of Home-Based Smoking Cessation Programs for Rural, Appalachian, Pregnant Smokers	Outcome - no health or behavioural outcomes
Harrison_2020	A consumer co-created infographic improves short-term knowledge about physical activity and self-efficacy to exercise in women with gestational diabetes mellitus: a randomised trial	Outcome - no health or behavioural outcomes
Hasani_2021	The effect of counseling based on health promotion awareness on mental health and self-esteem in women with ectopic pregnancy: a randomized controlled clinical trial	Outcome - no health or behavioural outcomes
Hausenblas_2008	Development and evaluation of a multimedia CD-ROM for exercise during pregnancy and postpartum	Outcome - no health or behavioural outcomes
Hazra_2020	Effects of health behaviour change intervention through women's self-help groups on maternal and newborn health practices and related inequalities in rural india: A quasi-experimental study	Outcome - no health or behavioural outcomes
Howarth_2019	Low-cost, self-paced, educational programmes increase birth satisfaction in first-time mothers	Outcome - no health or behavioural outcomes
Jack_2015	Reducing Preconception Risks Among African American Women with Conversational Agent Technology	Outcome - no health or behavioural outcomes
Ke_2021	Perioperative mobile application for mothers undergoing Cesarean delivery: a prospective cohort study on patient engagement	Outcome - no health or behavioural outcomes
Korukcu_2017	The effect of the mindfulness-based transition to motherhood program in pregnant women with preterm premature rupture of membranes	Outcome - no health or behavioural outcomes
Koury_2019	A 3Dimensional Anatomical Education Model in Postpartum Perineal Laceration Care: A PrePost Intervention Study	Outcome - no health or behavioural outcomes
Kuonza_2010	Non-adherence to the single dose nevirapine regimen for the prevention of mother-to-child transmission of HIV in Bindura town, Zimbabwe: a cross-sectional analytic study	Outcome - no health or behavioural outcomes
Lawrence_2003	Design and evaluation of interventions promoting periconceptional multivitamin use	Outcome - no health or behavioural outcomes
Ledford_2016	Mobile application as a prenatal education and engagement tool: A randomized controlled pilot	Outcome - no health or behavioural outcomes
Lepper_2018	Effectiveness of a Prison-Based Healthy Pregnancy Curriculum Delivered to Pregnant Inmates	Outcome - no health or behavioural outcomes
Liu_2019	Effects of exercise on pregnant women's quality of life: A systematic review	Outcome - no health or behavioural outcomes
Logsdon_2020	Partnering with new mothers to develop a smart phone app to prevent maternal mortality after hospital discharge: A pilot study	Outcome - no health or behavioural outcomes
Mahomed_2000	Home-based mother's record: operational feasibility, understanding and usage in a rural community in Zimbabwe	Outcome - no health or behavioural outcomes
Medley_2018	Interventions during pregnancy to prevent preterm birth: an overview of Cochrane systematic reviews	Outcome - no health or behavioural outcomes
Menard_2012	Self-collected vaginal swabs for the quantitative real-time polymerase chain reaction assay of Atopobium vaginae and Gardnerella vaginalis and the diagnosis of bacterial vaginosis	Outcome - no health or behavioural outcomes
Mendelson_2018	A mindfulness intervention to reduce maternal distress in neonatal intensive care: a mixed methods pilot study	Outcome - no health or behavioural outcomes
Mollart_2016	A feasibility randomised controlled trial of acupressure to assist spontaneous labour for primigravid women experiencing a post-date pregnancy	Outcome - no health or behavioural outcomes
Mozumdar_2018	Increasing knowledge of home based maternal and newborn care using self-help groups: Evidence from rural Uttar Pradesh, India	Outcome - no health or behavioural outcomes
Muhammad_2019	Pelvic Floor Muscle Exercise Education and Factors Associated with Implementation among Antenatal Women in Hospital Universiti Sains Malaysia	Outcome - no health or behavioural outcomes
Murphy_2005	Asthma self-management skills and the use of asthma education during pregnancy	Outcome - no health or behavioural outcomes

Author/Year	Title	Reasons for exclusion
Nadeau_2021	Beta Testing of a Gestational Diabetes Risk Reduction Intervention for American Indian and Alaska Native TeensPre-eclampsia Educational Tool Impact on Knowledge, Anxiety, and Satisfaction in Pregnant Women: A Randomized Trial	Outcome - no health or behavioural outcomes
Ockhuijsen_2015	Exploring a self-help coping intervention for pregnant women with a miscarriage history	Outcome - no health or behavioural outcomes
Osterman_2011	Feasibility of Using Motivational Interviewing to Decrease Alcohol Consumption During Pregnancy	Outcome - no health or behavioural outcomes
Parsa_2019	Improving the knowledge of pregnant women using a pre-eclampsia app: A controlled before and after study	Outcome - no health or behavioural outcomes
Paterno_2020	Feasibility of a pilot, randomized controlled trial using a personalized health monitoring device with pregnant women for behavioral sleep research	Outcome - no health or behavioural outcomes
PetrovFieril_2016	Hemodynamic responses to single sessions of aerobic exercise and resistance exercise in pregnancy	Outcome - no health or behavioural outcomes
Prado_2016	Effects of maternal and child lipid-based nutrient supplements on infant development: a randomized trial in Malawi	Outcome - no health or behavioural outcomes
Reilly_2021	Attitudes and engagement of pregnant and postnatal women with a web-based emotional health tool (mummatters): Cross-sectional study	Outcome - no health or behavioural outcomes
Rigaud_2017	Assessing a Tool for Self-Monitoring Hydration Using Urine Color in Pregnant and Breastfeeding Women: A Cross-Sectional, Online Survey	Outcome - no health or behavioural outcomes
Robins_2019	Treatment Fidelity in Mind–Body Interventions	Outcome - no health or behavioural outcomes
Ross-McGill_2000	Antenatal home blood pressure monitoring: a pilot randomised controlled trial	Outcome - no health or behavioural outcomes
Sapiano_2012	Attitudes towards preconception care in Maltese women with type 1 diabetes mellitus	Outcome - no health or behavioural outcomes
Sercekus_2010	Effects of antenatal education on maternal prenatal and postpartum adaptation	Outcome - no health or behavioural outcomes
Shahnazi_2016	Effect of educational intervention on perceived susceptibility self-efficacy and DMFT of pregnantwomen	Outcome - no health or behavioural outcomes
Sharps_2013	Engaging and retaining abused women in perinatal home visitation programs	Outcome - no health or behavioural outcomes
Snyder_2019	Mental and physical support (MAPS) for moms: preliminary findings from a prenatal health support program	Outcome - no health or behavioural outcomes
Szymanski_2018	Uterine Artery Doppler Velocimetry During Individually Prescribed Exercise in Pregnancy	Outcome - no health or behavioural outcomes
Thackeray_2017	Effectiveness of message framing on women's intention to perform cytomegalovirus prevention behaviors: A cross-sectional study	Outcome - no health or behavioural outcomes
Troy_1995	Development of a self-care guide for postpartum fatigue	Outcome - no health or behavioural outcomes
Truong_2019	Community pharmacist counseling in early pregnancy—Results from the SafeStart feasibility study	Outcome - no health or behavioural outcomes
Ventura_2012	Cortisol and anxiety response to a relaxing intervention on pregnant women awaiting amniocentesis	Outcome - no health or behavioural outcomes
Weis_2012	Mentors offering maternal support: a support intervention for military mothers	Outcome - no health or behavioural outcomes
Weis_2017	Mentors Offering Maternal Support Reduces Prenatal, Pregnancy-Specific Anxiety in a Sample of Military Women	Outcome - no health or behavioural outcomes
Aveyard-2005	The influence of in-pregnancy smoking cessation programmes on partner quitting and women's social support mobilization: A randomized controlled trial	Outcome - socioeconomic/financial
Ammerman_2011	An Open Trial of In-Home CBT for Depressed Mothers in Home Visitation	Population - beyond 42 days postpartum
Armstrong_2004	The effectiveness of a pram-walking exercise programme in reducing depressive symptomatology for postnatal women	Population - beyond 42 days postpartum
Ayers_2015	Brief Online Self-help Exercises for Postnatal Women to Improve Mood: A Pilot Study	Population - beyond 42 days postpartum
Ayers_2018	Evaluation of expressive writing for postpartum health: a randomised controlled trial	Population - beyond 42 days postpartum
Bennion_2020	Impact of an Internet-Based Lifestyle Intervention on Behavioral and Psychosocial Factors During Postpartum Weight Loss	Population - beyond 42 days postpartum
Berry_2013	Rationale, design, and methodology for the optimizing outcomes in women with gestational diabetes mellitus and their infants study	Population - beyond 42 days postpartum
Bick_2019	Protocol for a two-arm feasibility RCT to support postnatal maternal weight management and positive lifestyle behaviour in women from an ethnically diverse inner city population: The SWAN feasibility trial	Population - beyond 42 days postpartum
Brandon_2012	Self-help booklets for preventing postpartum smoking relapse: a randomized trial	Population - beyond 42 days postpartum
Branquinho_2020	A blended cognitive–behavioral intervention for the treatment of postpartum depression: study protocol for a randomized controlled trial	Population - beyond 42 days postpartum
Carson_2013	Evaluation of home testing to improve follow up after gestational diabetes (Fingerstick Assessments of sugar two-months postpartum or FAST)	Population - beyond 42 days postpartum

For peer review only - http://bmjopen.bmj.com/site/about/guidelines.xhtml

Author/Year	Title	Reasons for exclusion
Chen_2000	Effects of support group intervention in postnatally distressed women A controlled study in Taiwan	Population - beyond 42 days postpartum
Cramp_2006	Moms in motion: a group-mediated cognitive-behavioral physical activity intervention	Population - beyond 42 days postpartum
Cramp_2009	Sustaining self-regulatory efficacy and psychological outcome expectations for postnatal exercise: effects of a group-mediated cognitive behavioural intervention	Population - beyond 42 days postpartum
Crawley_2018	Feasibility and acceptability of expressive writing with postpartum women: A randomised controlled trial	Population - beyond 42 days postpartum
Daley_2008	Feasibility of an exercise intervention for women with postnatal depression: a pilot randomised controlled trial	Population - beyond 42 days postpartum
Daley_2012	The effectiveness of exercise as a treatment for postnatal depression: study protocol	Population - beyond 42 days postpartum
Daley_2015	A pragmatic randomized controlled trial to evaluate the effectiveness of a facilitated exercise intervention as a treatment for postnatal depression: the PAM-PeRS trial	Population - beyond 42 days postpartum
Daley_2020	Feasibility and acceptability of a brief routine weight management intervention for postnatal women embedded within the national child immunisation programme in primary care: randomised controlled cluster feasibility trial	Population - beyond 42 days postpartum
Daley_2021	Practice nurse-supported weight self-management delivered within the national child immunisation programme for postnatal women: a feasibility cluster RCT	Population - beyond 42 days postpartum
Danaher_2012	Web-based intervention for postpartum depression: Formative research and design of the mommoodbooster program	Population - beyond 42 days postpartum
Danaher_2013	MomMoodBooster web-based intervention for postpartum depression: feasibility trial results	Population - beyond 42 days postpartum
Dennis_2003	The effect of peer support on postpartum depression: a pilot randomized controlled trial	Population - beyond 42 days postpartum
deRosset_2013	Mama Sana Usted Sana: Lessons learned from a postpartum weight loss intervention for Hispanic women with infants six months or less	Population - beyond 42 days postpartum
Diebold_2021	Testing the effects of a prenatal depression preventive intervention on parenting and young children's self-regulation and functioning (EPIC): protocol for a longitudinal observational study	Population - beyond 42 days postpartum
Dunning_2013	Wide Awake Parenting: Study protocol for a randomised controlled trial of a parenting program for the management of post-partum fatigue	Population - beyond 42 days postpartum
Fjeldsoe_2010	MobileMums: A randomized controlled trial of an SMS-based physical activity intervention	Population - beyond 42 days postpartum
Fleming_1992	The effects of a social support group on depression, maternal attitudes and behavior in new mothers	Population - beyond 42 days postpartum
Fonseco_2019	Be a Mom, a WebBased Intervention to Prevent Postpartum Depression: The Enhancement of SelfRegulatory Skills and Its Association With Postpartum Depressive Symptoms	Population - beyond 42 days postpartum
Gammer_2020	A randomized controlled trial of an online, compassion-based intervention for maternal psychological well-being in the first year postpartum	Population - beyond 42 days postpartum
Giallo_2015	The efficacy of an intervention for the management of postpartum fatigue	Population - beyond 42 days postpartum
Golmakani_2015	The effect of pelvic floor muscle exercises program on sexual self-efficacy in primiparous women after delivery	Population - beyond 42 days postpartum
Gutke_2010	Specific muscle stabilizing as home exercises for persistent pelvic girdle pain after pregnancy: a randomized, controlled clinical trial	Population - beyond 42 days postpartum
Herring_2014	Using Technology to Promote Postpartum Weight Loss in Urban, Low-Income Mothers: A Pilot Randomized Controlled Trial	Population - beyond 42 days postpartum
Herring_2020	Feasibility of using a peer coach to deliver a behavioral intervention for promoting postpartum weight loss in Black and Latina mothers	Population - beyond 42 days postpartum
Kim_2021	Self-management mobile virtual reality program for women with gestational diabetes	Population - beyond 42 days postpartum
Koniak-Griffin_2003	Nurse visitation for adolescent mothers: two-year infant health and maternal outcomes	Population - beyond 42 days postpartum
Krummel_2010	Lessons Learned from the Mothers' Overweight Management Study in 4 West Virginia WIC Offices	Population - beyond 42 days postpartum
LeCheminant_2014	Effect of resistance training on body composition, self-efficacy, depression, and activity in postpartum women	Population - beyond 42 days postpartum
Mailey_2019	Is a general or specific exercise recommendation more effective for promoting physical activity among postpartum mothers?	Population - beyond 42 days postpartum
Mitchell_2018	Feasibility and acceptability of a brief online self-compassion intervention for mothers of infants	Population - beyond 42 days postpartum
Monteiro_2021	Be a Mom, a WebBased Intervention to Promote Positive Mental Health Among Postpartum Women With Low Risk for Postpartum Depression: Exploring Psychological Mechanisms of Change	Population - beyond 42 days postpartum
Morrell_2009	Clinical effectiveness of health visitor training in psychologically informed approaches for depression in postnatal women: pragmatic cluster randomised trial in primary care	Population - beyond 42 days postpartum
Muijsers_2020	Blood pressure after PREeclampsia/HELLP by SELF monitoring (BP-PRESELF): Rationale and design of a multicenter randomized controlled trial	Population - beyond 42 days postpartum
Murphy_2017	Pilot trial of a parenting and self-care intervention for HIV-positive mothers: the IMAGE program	Population - beyond 42 days postpartum

Author/Year	Title	Reasons for exclusion
Peacock_2015	A randomised controlled trial to delay or prevent type 2 diabetes after gestational diabetes: walking for exercise and nutrition to prevent diabetes for you	Population - beyond 42 days postpartum
Perez-Blasco_2013	Effects of a mindfulness-based intervention on psychological distress, well-being, and maternal self-efficacy in breast-feeding mothers: results of a pilot study	Population - beyond 42 days postpartum
Phelan_2017	Effect of an Internet-Based Program on Weight Loss for Low-Income Postpartum Women: a Randomized Clinical Trial	Population - beyond 42 days postpartum
Power_2019	Engagement and Weight Loss in a Web and Mobile Program for Low-Income Postpartum Women: fit Moms/Mamás Activas	Population - beyond 42 days postpartum
Rich-Edwards_2019	Randomized Trial to Reduce Cardiovascular Risk in Women with Recent Preeclampsia	Population - beyond 42 days postpartum
Rollo_2020	The feasibility and preliminary efficacy of an ehealth lifestyle program in women with recent gestational diabetes mellitus: a pilot study	Population - beyond 42 days postpartum
Sampson_2020	Reducing depression among mothers in a residential treatment setting: building evidence for the PST4PPD intervention	Population - beyond 42 days postpartum
Swanson_2009	Resolution of depression and grief during the first year after miscarriage: a randomized controlled clinical trial of couples-focused interventions	Population - beyond 42 days postpartum
Teychenne_2021	Mums on the Move: A pilot randomised controlled trial of a home-based physical activity intervention for mothers at risk of postnatal depression	Population - beyond 42 days postpartum
Timlin_2017	A preliminary randomised control trial of the effects of Dru yoga on psychological well-being in Northern Irish first time mothers	Population - beyond 42 days postpartum
Tripette_2014	Home-Based Active Video Games to Promote Weight Loss during the Postpartum Period	Population - beyond 42 days postpartum
Walker_2012	Ethnic-Specific Weight-Loss Interventions for Low-Income Postpartum Women: Findings and Lessons	Population - beyond 42 days postpartum
Watson_2005	The feasibility and effectiveness of pram walking groups for postpartum women in western Sydney	Population - beyond 42 days postpartum
Wilkinson_2015	Trial for Reducing Weight Retention in New Mums: a randomised controlled trial evaluating a low intensity, postpartum weight management programme	Population - beyond 42 days postpartum
Abbasi_2017	Comparing the effect of e-learning and educational booklet on the childbirth self-efficacy: a randomized controlled clinical trial	Population - not targeting pregnant women
Aleman_2017	Brief counseling on secondhand smoke exposure in pregnant women in Argentina and uruguay	Population - not targeting pregnant women
Althabe_2016	A Multifaceted Strategy to Implement Brief Smoking Cessation Counseling During Antenatal Care in Argentina and Uruguay: A Cluster Randomized Trial	Population - not targeting pregnant women
Baradaran-Akbarzadeh_2018	The effect of educational package on sexual function in cold temperament women of reproductive age	Population - not targeting pregnant women
Briggs_2016	A systematic review of interventions to improve knowledge and self-management skills concerning contraception, pregnancy and breastfeeding in people with rheumatoid arthritis	Population - not targeting pregnant women
Chomat_2019	Women's circles as a culturally safe psychosocial intervention in Guatemalan indigenous communities: a community-led pilot randomised trial	Population - not targeting pregnant women
DeNicola_2020	Telehealth Interventions to Improve Obstetric and Gynecologic Health Outcomes: A Systematic Review	Population - not targeting pregnant women
Fallin-Bennett_2018	Pilot Tobacco Treatment Intervention for Women in Residential Treatment for Substance Use Disorder	Population - not targeting pregnant women
Fisher_2018	Addressing multiple modifiable risks through structured community-based Learning Clubs to improve maternal and infant health and infant development in rural Vietnam: protocol for a parallel group cluster randomised controlled trial	Population - not targeting pregnant women
Ghetti_2019	Longitudinal Study of music Therapy's Effectiveness for Premature infants and their caregivers (LongSTEP): protocol for an international randomised trial	Population - not targeting pregnant women
Harvey_2008	Hygiene interventions for prevention of cytomegalovirus infection among childbearing women: Systematic review	Population - not targeting pregnant women
Hillemeier_2008	Improving women's preconceptional health: findings from a randomized trial of the Strong Healthy Women intervention in the Central Pennsylvania women's health study	Population - not targeting pregnant women
Johnson_2018	Protocol for the ROSE sustainment (ROSES) study, a sequential multiple assignment randomized trial to determine the minimum necessary intervention to maintain a postpartum depression prevention program in prenatal clinics serving low-income women	Population - not targeting pregnant women
Karimiankakolaki_2019	Designing an educational intervention on second-hand smoke in smoker men on the exposure of pregnant wives: a protocol for a randomized controlled trial	Population - not targeting pregnant women
Karyotaki_2021	The effects of psychological treatment of perinatal depression: an overview	Population - not targeting pregnant women
Mackintosh_2020	Interventions to increase patient and family involvement in escalation of care for acute life-threatening illness in community health and hospital settings	Population - not targeting pregnant women
Makaruk_2019	The Effectiveness of Regular Exercise Programs in the Prevention of Gestational Diabetes Mellitus-A Systematic Review	Population - not targeting pregnant women
Matkin_2019	Telephone counselling for smoking cessation	Population - not targeting pregnant women
McRobbie_2016	Tackling obesity in areas of high social deprivation: clinical effectiveness and cost-effectiveness of a task-based weight management group programme - A randomised controlled trial and economic evaluation	Population - not targeting pregnant women

Author/Year	Title	Reasons for exclusion
Mutale_2021	Addition of HIV self-test kits to partner notification services to increase HIV testing of male partners of pregnant women in Zambia: two parallel randomised trials	Population - not targeting pregnant women
Nayak_2019	Randomized Trial of an Innovative Electronic Screening and Brief Intervention for Reducing Drinking among Women of Childbearing Age	Population - not targeting pregnant women
Nguyen_2018	Engagement of Husbands in a Maternal Nutrition Program Substantially Contributed to Greater Intake of Micronutrient Supplements and Dietary Diversity during Pregnancy: Results of a Cluster-Randomized Program Evaluation in Bangladesh	Population - not targeting pregnant women
O'Dea_2015	Can the Onset of Type 2 Diabetes Be Delayed by a Group-Based Lifestyle Intervention in Women with Prediabetes following Gestational Diabetes Mellitus (GDM)? Findings from a Randomized Control Mixed Methods Trial	Population - not targeting pregnant women
Okeibunor_2011	Preventing malaria in pregnancy through community-directed interventions: Evidence from Akwa Ibom State, Nigeria	Population - not targeting pregnant women
Ovretveit_2021	Innovations in self care and close care made during COVID 19 pandemic: a narrative review	Population - not targeting pregnant women
Pettifor_2020	HIV self-testing among young women in rural South Africa: A randomized controlled trial comparing clinic-based HIV testing to the choice of either clinic testing or HIV self-testing with secondary distribution to peers and partners	Population - not targeting pregnant women
Pinchoff_2019	Evidence-Based Process for Prioritizing Positive Behaviors for Promotion: Zika Prevention in Latin America and the Caribbean and Applicability to Future Health Emergency Responses	Population - not targeting pregnant women
Pintye_2019	Acceptability and outcomes of distributing HIV selftests for male partner testing in Kenyan maternal and child health and family planning clinics	Population - not targeting pregnant women
Shen_2021	Clinical Evaluation of a Self-Testing Kit for Vaginal Infection Diagnosis	Population - not targeting pregnant women
Taylor_2019	Be Healthe for Your Heart: protocol for a Pilot Randomized Controlled Trial Evaluating a Web-Based Behavioral Intervention to Improve the Cardiovascular Health of Women With a History of Preeclampsia	Population - not targeting pregnant women
Tieu_2017	Preconception care for diabetic women for improving maternal and infant health	Population - not targeting pregnant women
van-der-Zwan_2019	The Effect of Heart Rate Variability Biofeedback Training on Mental Health of Pregnant and Non-Pregnant Women: a Randomized Controlled Trial	Population - not targeting pregnant women
Vézina-Im_2016	Implementation intentions intervention to promote fruit and vegetable intake in childbearing age women at risk for gestational diabetes mellitus: 6-month follow-up of a randomized controlled trial	Population - not targeting pregnant women
Youngkin_2010	Promoting self-care and secondary prevention in women's health: A study to test the accuracy of a home self-test system for bacterial vaginosis	Population - not targeting pregnant women
Adolfsson_2014	A web-based support for pregnant women and new mothers with type 1 diabetes mellitus in Sweden (MODIAB-Web): Study protocol for a randomized controlled trial	Protocol - results already included
Bais_2016	Bright light therapy in pregnant women with major depressive disorder: study protocol for a randomized, double-blind, controlled clinical trial	Protocol - results already included
Band_2019	Intervention planning and modification of the BUMP intervention: A digital intervention for the early detection of raised blood pressure in pregnancy	Protocol - results already included
Clifton_2016	Design of lifestyle intervention trials to prevent excessive gestational weight gain in women with overweight or obesity	Protocol - results already included
Cooper_2015	Pilot study to evaluate a tailored text message intervention for pregnant smokers (MiQuit): Study protocol for a randomised controlled trial	Protocol - results already included
Dodd_2011	Limiting weight gain in overweight and obese women during pregnancy to improve health outcomes: the LIMIT randomised controlled trial	Protocol - results already included
Dol_2019	Impact of mobile health interventions during the perinatal period for mothers in low- and middle-income countries: a systematic review protocol	Protocol - results already included
Evans_2019	Design of a novel digital intervention to promote healthy weight management among postpartum African American women	Protocol - results already included
Garmendia_2018	Effectiveness on maternal and offspring metabolic control of a home-based dietary counseling intervention and DHA supplementation in obese/overweight pregnant women (MIGHT study): A randomized controlled trial-Study protocol	Protocol - results already included
Griffiths_2016	Are digital interventions for smoking cessation in pregnancy effective? A systematic review protocol	Protocol - results already included
Grzeskowiak_2014	A randomized controlled trial to assess the clinical and cost effectiveness of a nurse-led Antenatal Asthma Management Service in South Australia (AAMS study)	Protocol - results already included
Jones_2012	Pain management for women in labour: an overview of systematic reviews	Protocol - results already included
Kinser_2020	Protocol for Pilot Study on Self-Management of Depressive Symptoms in Pregnancy	Protocol - results already included
Lim_2019	Smart Phone APP to Restore Optimal Weight (SPAROW): protocol for a randomised controlled trial for women with recent gestational diabetes	Protocol - results already included
Loukopoulou_2011	Design and study protocol of the maternal smoking cessation during pregnancy study, (M-SCOPE)	Protocol - results already included
Lucas_2019	Promoting self-management of breast and nipple pain in breastfeeding women: Protocol of a pilot randomized controlled trial	Protocol - results already included
Miquelutti_2015	Developing strategies to be added to the protocol for antenatal care: an exercise and birth preparation program	Protocol - results already included
Navas_2018	Effectiveness and safety of moderate-intensity aerobic water exercise during pregnancy for reducing use of epidural analgesia during labor: protocol for a randomized clinical trial	Protocol - results already included

Author/Year	Title	Reasons for exclusion
Sapkota_2019	Counselling-based psychosocial intervention to improve the mental health of abused pregnant women: a protocol for randomised controlled feasibility trial in a tertiary hospital in eastern Nepal	Protocol - results already included
Ternstrom_2017	A randomized controlled study comparing internet-based cognitive behavioral therapy and counselling by standard care for fear of birth - A study protocol	Protocol - results already included
Trevillion_2015	Depression: an exploratory parallel-group randomised controlled trial of antenatal guided self help for women (dawn trial)	Protocol - results already included
Whitemore_2019	Effectiveness and cost-effectiveness of a tailored text-message programme (MiQuit) for smoking cessation in pregnancy: Study protocol for a randomised controlled trial (RCT) and meta-analysis	Protocol - results already included
Willcox_2015	Testing the feasibility of a mobile technology intervention promoting healthy gestational weight gain in pregnant women (txt4two) - study protocol for a randomised controlled trial	Protocol - results already included
Zairina_2015	Study protocol for a randomised controlled trial evaluating the efficacy of a telehealth program - management of asthma with supportive telehealth of respiratory function in pregnancy (MASTERY©)	Protocol - results already included
Aslani_2014	Impact of Computer-Based Pregnancy-Induced Hypertension and Diabetes Decision Aids on Empowering Pregnant Women	Protocol - results not published
Baird_2016	Southampton PRegnancy Intervention for the Next Generation (SPRING): protocol for a randomised controlled trial	Protocol - results not published
Bei_2019	A scalable cognitive behavioural program to promote healthy sleep during pregnancy and postpartum periods: Protocol of a randomised controlled trial (the SEED project)	Protocol - results not published
Browne_2015	Uterine Tonus Assessment by Midwives versus Patient self-assessment in the active management of the third stage of labor (UTAMP): Study protocol for a randomized controlled trial	Protocol - results not published
Caramlau_2011	Mums 4 Mums: structured telephone peer-support for women experiencing postnatal depression Pilot and exploratory RCT of its clinical and cost effectiveness	Protocol - results not published
Carolan-Olah_2015	Development and initial testing of a GDM information website for multi-ethnic women with GDM	Protocol - results not published
Chang_2015	Electronic personal maternity records: Both web and smartphone services	Protocol - results not published
Dallosso_2018	Baby Steps - A structured group education programme with accompanying mobile web application designed to promote physical activity in women with a history of gestational diabetes: Study protocol for a randomised controlled trial	Protocol - results not published
Davis_2018	Optimizing Gestational Weight Gain With the Eating4Two Smartphone App: Protocol for a Randomized Controlled Trial	Protocol - results not published
Edwards_2019	Can an educational web-intervention, co-created by service users alongside self-efficacy theory, affect nulliparous women's experiences of early labour? A study protocol for a randomised control trial (the L-TEL Trial)	Protocol - results not published
Hawkins_2019	Feasibility of a Sleep Self-Management Intervention in Pregnancy Using a Personalized Health Monitoring Device: Protocol for a Pilot Randomized Controlled Trial	Protocol - results not published
Koushede_2013	Group-based antenatal birth and parent preparation for improving birth outcomes and parenting resources: study protocol for a randomised trial	Protocol - results not published
LeBlanc_2016	Prepare, a randomized trial to promote and evaluate weight loss among overweight and obese women planning pregnancy: Study design and rationale	Protocol - results not published
Lenz_2018	Mindfulness-based Stress Reduction in Pregnancy: an App-Based Programme to Improve the Health of Mothers and Children (MINDFUL/PMI Study)	Protocol - results not published
Martin_2018	A pilot randomized controlled trial of a remotely-delivered behavioral health coaching program to limit weight gain in pregnancy and reduce postpar-tum weight retention	Protocol - results not published
Monteiro_2019	Non-pharmacological interventions for treating sexual dysfunction in postpartum women: A systematic review protocol	Protocol - results not published
Nagle_2013	Primary prevention of gestational diabetes for women who are overweight and obese: a randomised controlled trial	Protocol - results not published
Nwolise_2017	Exploring the acceptability and feasibility of a preconception and diabetes information app for women with pregestational diabetes: A mixed-methods study protocol	Protocol - results not published
Pallitto_2016	Testing a counselling intervention in antenatal care for women experiencing partner violence: a study protocol for a randomized controlled trial in Johannesburg, South Africa	Protocol - results not published
Pascual-Morena_2019	Exercise vs metformin for gestational diabetes mellitus: Protocol for a network meta-analysis	Protocol - results not published
Patten_2019	The Healthy Pregnancies Project: Study protocol and baseline characteristics for a cluster-randomized controlled trial of a community intervention to reduce tobacco use among Alaska Native pregnant women	Protocol - results not published
Pelaez-Crisologo_2009	Different techniques of blood glucose monitoring in women with gestational diabetes for improving maternal and infant health	Protocol - results not published
Prabhu_2020	The effect of a prenatal psychosocial education program on postnatal depression, stress, and parenting self-efficacy in women in South India - A study protocol for a randomised controlled trial	Protocol - results not published
Qian_2021	Effectiveness of non-pharmacological interventions for reducing postpartum fatigue: a systematic review protocol	Protocol - results not published
Reynolds_2016	MAHILA: a protocol for evaluating a nurse-delivered mHealth intervention for women with HIV and psychosocial risk factors in India	Protocol - results not published
Roberts_2004	Protocol for a randomised controlled trial of a decision aid for the management of pain in labour and childbirth	Protocol - results not published
Sacristan-Martin_2019	A mindfulness and compassion-based program applied to pregnant women and their partners to decrease depression symptoms during pregnancy and postpartum: study protocol for a randomized controlled trial	Protocol - results not published

For peer review only

Author/Year	Title	Reasons for exclusion
Seo_2021	The Development of the Postpartum Depression Self-Management Mobile Application "happy Mother"	Protocol - results not published
Singh_2017	Internet-delivered blood glucose awareness training (BGAT) for women with type 1 diabetes (T1D) contemplating pregnancy: results from the Bump2be trial	Protocol - results not published
Tielsch_2015	Designs of two randomized, community-based trials to assess the impact of alternative cookstove installation on respiratory illness among young children and reproductive outcomes in rural Nepal	Protocol - results not published
Tomfohr-Madsen_2016	Mindfulness-based cognitive therapy for psychological distress in pregnancy: study protocol for a randomized controlled trial	Protocol - results not published
Tomlinson_2011	An effectiveness study of an integrated, community-based package for maternal, newborn, child and HIV care in South Africa: study protocol for a randomized controlled trial	Protocol - results not published
Veringa_2016	I've Changed My Mind', Mindfulness-Based Childbirth and Parenting (MBCP) for pregnant women with a high level of fear of childbirth and their partners: study protocol of the quasi-experimental controlled trial	Protocol - results not published
Volrathongchai_2013	Non-pharmacological interventions for women with postpartum fatigue	Protocol - results not published
Wilcox_2018	A randomized controlled trial to prevent excessive gestational weight gain and promote postpartum weight loss in overweight and obese women: Health In Pregnancy and Postpartum (HIPP)	Protocol - results not published
Yeung_2019	Birth ball for pregnant women in labour research protocol: A multi-centre randomised controlled trial	Protocol - results not published

For peer review only

Supplementary File S6. Reference list of included studies

1. Aaronson NK, Ershoff DH, Danaher BG: Smoking cessation in pregnancy: a self-help approach. *Addict Behav* 1985, 10(1):103-108.
2. Abdel-Aleem H, Singata M, Abdel-Aleem M, Mshweshwe N, Williams X, Hofmeyr GJ: Uterine massage to reduce postpartum hemorrhage after vaginal delivery. *Int J Gynaecol Obstet* 2010, 111(1):32-36.
3. Abedi P, Jahanfar S, Namvar F, Lee J: Breastfeeding or nipple stimulation for reducing postpartum haemorrhage in the third stage of labour. In., vol. 2016; 2016.
4. Adams J, Frawley J, Steel A, Broom A, Sibbritt D: Use of pharmacological and non-pharmacological labour pain management techniques and their relationship to maternal and infant birth outcomes: examination of a nationally representative sample of 1835 pregnant women. *Midwifery* 2015, 31(4):458-463.
5. Adler SP, Finney JW, Manganello AM, Best AM: Prevention of child-to-mother transmission of cytomegalovirus among pregnant women. *J Pediatr* 2004, 145(4):485-491.
6. Afravi S, Abbaspoor Z, Montazeri S, Cheraghian B: The effect of Hugo point pressure on postpartum pain in multiparous women. *Family Medicine & Primary Care Review* 2019, 21(1):7-11.
7. Ahmadpour P, Mosavi S, Mohammad-Alizadeh-Charandabi S, Jahanfar S, Mirghafourvand M: Evaluation of the birth plan implementation: a parallel convergent mixed study. *Reprod Health* 2020, 17(1):138.
8. Ajibaye O, Balogun EO, Olukosi YA, Orok BA, Oyebola KM, Iwalokun BA, Aina OO, Shittu O, Adeneye AK, Ojewunmi OO *et al*: Impact of training of mothers, drug shop attendants and voluntary health workers on effective diagnosis and treatment of malaria in Lagos, Nigeria. *Trop Parasitol* 2019, 9(1):36-44.
9. Ajuzieogu OV, Amucheazi A, Ezike HA, Achi J, Abam DS: The efficacy of chewing gum on postoperative ileus following cesarean section in Enugu, South East Nigeria: A randomized controlled clinical trial. *Niger J Clin Pract* 2014, 17(6):739-742.
10. Akselsson A, Lindgren H, Georgsson S, Pettersson K, Steineck G, Skokic V, Radestad I: Mindfetalness to increase women's awareness of fetal movements and pregnancy outcomes: a cluster-randomised controlled trial including 39 865 women. *BJOG* 2020, 127(7):829-837.
11. Al Hashmi I, Nandy K, Seshan V: Non-medical strategies to improve pregnancy outcomes of women with gestational diabetes mellitus: a literature review. In., vol. 19; 2019.
12. Al Khamis S, Asimakopoulou K, Newton T, Daly B: The effect of dental health education on pregnant women's adherence with toothbrushing and flossing - A randomized control trial. *Community Dent Oral Epidemiol* 2017, 45(5):469-477.
13. Al-Hashmi I, Hodge F, Nandy K, Thomas E, Brecht ML: The Effect of a Self-Efficacy-Enhancing Intervention on Perceived Self-Efficacy and Actual Adherence to Healthy Behaviours Among Women with Gestational Diabetes Mellitus. *Sultan Qaboos Univ Med J* 2018, 18(4):e513-e519.
14. Alekseev NP, Vladimir, II, Nadezhda TE: Pathological postpartum breast engorgement: prediction, prevention, and resolution. *Breastfeed Med* 2015, 10(4):203-208.
15. Alexander DA, Northcross A, Karrison T, Morhasson-Bello O, Wilson N, Atalabi OM, Dutta A, Adu D, Ibigbami T, Olamijulo J *et al*: Pregnancy outcomes and ethanol cook stove intervention: A randomized-controlled trial in Ibadan, Nigeria. *Environ Int* 2018, 111:152-163.
16. Alfadhli E, Osman E, Basri T: Use of a real time continuous glucose monitoring system as an educational tool for patients with gestational diabetes. *Diabetol Metab Syndr* 2016, 8(1):48.
17. Alizadeh S, Riazi H, Majd HA, Ozgoli G: The effect of sexual health education on sexual activity, sexual quality of life, and sexual violence in pregnancy: a prospective randomized controlled trial. *BMC Pregnancy Childbirth* 2021, 21(1):334.
18. Alnuaimi K, Abuidhail J, Abuzaid H: The effects of an educational programme about preeclampsia on women's awareness: a randomised control trial. *Int Nurs Rev* 2020, 67(4):501-511.

For peer review only

19. Altazan AD, Redman LM, Burton JH, Beyl RA, Cain LE, Sutton EF, Martin CK: Mood and quality of life changes in pregnancy and postpartum and the effect of a behavioral intervention targeting excess gestational weight gain in women with overweight and obesity: a parallel-arm randomized controlled pilot trial. *BMC Pregnancy Childbirth* 2019, 19(1):50.
20. Ambardekar S, Shochet T, Bracken H, Coyaji K, Winikoff B: Calibrated delivery drape versus indirect gravimetric technique for the measurement of blood loss after delivery: a randomized trial. *BMC Pregnancy Childbirth* 2014, 14(1):276.
21. Anderson L, Kynoch K, Kildea S, Lee N: Effectiveness of breast massage for the treatment of women with breastfeeding problems: a systematic review. *JBI Database System Rev Implement Rep* 2019, 17(8):1668-1694.
22. Anderson NH, Gordon A, Li M, Cronin RS, Thompson JMD, Raynes-Greenow CH, Heazell AEP, Stacey T, Culling VM, Wilson J *et al*: Association of Supine Going-to-Sleep Position in Late Pregnancy With Reduced Birth Weight: A Secondary Analysis of an Individual Participant Data Meta-analysis. *JAMA Netw Open* 2019, 2(10):e1912614.
23. Antoszewski B, Sobczak M, Kasielska-Trojan A: Self-assessment of striae gravidarum prophylaxis. *Postepy Dermatol Alergol* 2015, 32(6):459-464.
24. Aprilina HD, Krislinggardini K, Isnaini N, Suratmi S: The Effect of Cabbage Leaves Compress on Breast Engorgement in Postpartum Mother. *Open Access Macedonian Journal of Medical Sciences* 2021, 9(T4):124-128.
25. Araban M, Baharzadeh K, Karimy M: Nutrition modification aimed at enhancing dietary iron and folic acid intake: an application of health belief model in practice. *Eur J Public Health* 2017, 27(2):287-292.
26. Araban M, Tavafian SS, Zarandi SM, Hidarnia AR, Burri A, Montazeri A: A behavioral strategy to minimize air pollution exposure in pregnant women: a randomized controlled trial. *Environ Health Prev Med* 2017, 22(1):26.
27. Ardilouze A, Bouchard P, Hivert MF, Simard C, Allard C, Garant MP, Menard J, Ouellet A, Houde G, Pesant MH *et al*: Self-Monitoring of Blood Glucose: A Complementary Method Beyond the Oral Glucose Tolerance Test to Identify Hyperglycemia During Pregnancy. *Can J Diabetes* 2019, 43(8):627-635.
28. Arimori N: Randomized controlled trial of decision aids for women considering prenatal testing: The effect of the Ottawa Personal Decision Guide on decisional conflict. *Japan Journal of Nursing Science* 2006, 3(2):119-130.
29. Arthur C, Di Corleto E, Ballard E, Kothari A: A randomized controlled trial of daily weighing in pregnancy to control gestational weight gain. *BMC Pregnancy Childbirth* 2020, 20(1):223.
30. Asci O, Rathfisch G: Effect of lifestyle interventions of pregnant women on their dietary habits, lifestyle behaviors, and weight gain: a randomized controlled trial. *J Health Popul Nutr* 2016, 35:7.
31. Ashorn P, Alho L, Ashorn U, Cheung YB, Dewey KG, Harjunmaa U, Lartey A, Nkhoma M, Phiri N, Phuka J *et al*: The impact of lipid-based nutrient supplement provision to pregnant women on newborn size in rural Malawi: a randomized controlled trial. *Am J Clin Nutr* 2015, 101(2):387-397.
32. Ashworth DC, Maule SP, Stewart F, Nathan HL, Shennan AH, Chappell LC: Setting and techniques for monitoring blood pressure during pregnancy. In., vol. 2020; 2020.
33. Avery MD, Leon AS, Kopher RA: Effects of a partially home-based exercise program for women with gestational diabetes. *Obstet Gynecol* 1997, 89(1):10-15.
34. Avsar TS, McLeod H, Jackson L: Health outcomes of smoking during pregnancy and the postpartum period: an umbrella review. *BMC Pregnancy Childbirth* 2021, 21(1):254.
35. Bagharpoosh M, Sangestani G, Goodarzi M: Effect of progressive muscle relaxation technique on pain relief during labor. *Acta Medica Iranica* 2006, 44(3).
36. Bais B, Kamperman AM, Bijma HH, Hoogendijk WJ, Souman JL, Knijff E, Lambregtse-van den Berg MP: Effects of bright light therapy for depression during pregnancy: a randomised, double-blind controlled trial. *BMJ Open* 2020, 10(10):e038030.

For peer review only

37. Balami AD, Said SM, Zulkefli NAM, Norsa'adah B, Audu B: Improving malaria preventive practices and pregnancy outcomes through a health education intervention: A randomized controlled trial. *Malar J* 2021, 20(1):55.
38. Bao H, Yu P, Song X, Zhou Y, Zhu Y, Xu X: The influence of home-based exercise on gestational diabetes: a meta-analysis of randomized controlled trials. *J Matern Fetal Neonatal Med* 2020, 33(13):2300-2305.
39. Barakat R, Vargas M, Brik M, Fernandez I, Gil J, Coteron J, Santacruz B: Does Exercise During Pregnancy Affect Placental Weight?: A Randomized Clinical Trial. *Eval Health Prof* 2018, 41(3):400-414.
40. Barber V, Calvert A, Vandrevala T, Star C, Khalil A, Griffiths P, Heath PT, Jones CE: Prevention of Acquisition of Cytomegalovirus Infection in Pregnancy through Hygiene-based Behavioral Interventions: A Systematic Review and Gap Analysis. In.; 2020.
41. Barroso CS, Yockey A, Degon E, Gautam Poudel P, Brown SD, Hedderson MM, Moreno-Hunt C, Ehrlich SF: Efficacious lifestyle interventions for appropriate gestational weight gain in women with overweight or obesity set in the health care system: a scoping review. In.; 2021.
42. Bartu A, Sharp J, Ludlow J, Doherty DA: Postnatal home visiting for illicit drug-using mothers and their infants: a randomised controlled trial. *Aust N Z J Obstet Gynaecol* 2006, 46(5):419-426.
43. Baruth M, Schlaff RA, Deere S, Walker JL, Dressler BL, Wagner SF, Boggs A, Simon HA: The Feasibility and Efficacy of a Behavioral Intervention to Promote Appropriate Gestational Weight Gain. *Matern Child Health J* 2019, 23(12):1604-1612.
44. Bastani F, Hidarnia A, Kazemnejad A, Vafaei M, Kashanian M: A randomized controlled trial of the effects of applied relaxation training on reducing anxiety and perceived stress in pregnant women. *J Midwifery Womens Health* 2005, 50(4):e36-40.
45. Bastani FF, Sharami SH, Aski SK, Milani F, Khakifirooz B, Shakiba M, Attari SM, Omidkhah T: The Effect of Simultaneous Application of Transcutaneous Electrical Nerve Stimulation (TENS) on Specific Lumbar and Acupuncture Points on Labour Pain Relief: A Randomised Clinical Trial. *Journal of Clinical and Diagnostic Research* 2020.
46. Bastiaenen CH, de Bie RA, Vlaeyen JW, Goossens ME, Leffers P, Wolters PM, Bastiaanssen JM, Brandt PA, Essed GG: Long-term effectiveness and costs of a brief self-management intervention in women with pregnancy-related low back pain after delivery. *BMC Pregnancy Childbirth* 2008, 8:19.
47. Batra P, Mangione CM, Cheng E, Steers WN, Nguyen TA, Bell D, Kuo AA, Gregory KD: A Cluster Randomized Controlled Trial of the MyFamilyPlan Online Preconception Health Education Tool. *Am J Health Promot* 2018, 32(4):897-905.
48. Baumel A, Tinkelman A, Mathur N, Kane JM: Digital Peer-Support Platform (7Cups) as an Adjunct Treatment for Women With Postpartum Depression: Feasibility, Acceptability, and Preliminary Efficacy Study. *JMIR Mhealth Uhealth* 2018, 6(2):e38.
49. Beckmann MM, Stock OM: Antenatal perineal massage for reducing perineal trauma. *Cochrane Database Syst Rev* 2013(4):CD005123.
50. Beetham KS, Giles C, Noetel M, Clifton V, Jones JC, Naughton G: The effects of vigorous intensity exercise in the third trimester of pregnancy: A systematic review and meta-analysis. In., vol. 19; 2019.
51. Beleza ACS, Ferreira C, Meirelles M, Santos C, Nakanno AM: The effect of cryotherapy in perineal pain after vaginal childbirth with episiotomy. *Physiotherapy (united kingdom)* 2011, 97.
52. Bellussi F, Po G, Livi A, Saccone G, De Vivo V, Oliver EA, Berghella V: Fetal Movement Counting and Perinatal Mortality: A Systematic Review and Meta-analysis. *Obstet Gynecol* 2020, 135(2):453-462.
53. Berghella V, Di Mascio D: Evidence-based labor management: before labor (Part 1). In., vol. 2; 2020.
54. Bergman E, Axelsson O, Petzold M, Sonesson C, Kieler H: Self-administered symphysis-fundus measurements analyzed with a novel statistical method for detection of intrauterine growth restriction: a clinical evaluation. *Acta Obstet Gynecol Scand* 2011, 90(8):890-896.

For peer review only

55. Bergstrom S: External cephalic version and daily post-versional maternal self-assessment of fetal presentation. A prospective study. *Gynecol Obstet Invest* 1992, 33(1):15-18.

56. Berta M, Lindgren H, Christensson K, Mekonnen S, Adefris M: Effect of maternal birth positions on duration of second stage of labor: systematic review and meta-analysis. *BMC Pregnancy Childbirth* 2019, 19(1):466.

57. Bhutta ZA, Rizvi A, Raza F, Hotwani S, Zaidi S, Moazzam Hossain S, Soofi S, Bhutta S: A comparative evaluation of multiple micronutrient and iron-folic acid supplementation during pregnancy in Pakistan: impact on pregnancy outcomes. *Food Nutr Bull* 2009, 30(4 Suppl):S496-505.

58. Biana CB, Cecagno D, Porto AR, Cecagno S, Marques VA, Soares MC: Non-pharmacological therapies applied in pregnancy and labor: an integrative review. *Rev Esc Enferm USP* 2021, 55:e03681.

59. Bilgin CN, Ak B, Ayhan F, Kocyigit F, Yorgun S, Topcuoglu MA: Effect of childbirth education on the perceptions of childbirth and breastfeeding self-efficacy and the obstetric outcomes of nulliparous women. *Health Care Women Int* 2020, 41(2):188-204.

60. Bitzer EM, Schneider A, Wenzlaff P, Hoyme UB, Siegmund-Schultze E: Self-testing of vaginal pH to prevent preterm delivery: a controlled trial. *Dtsch Arztebl Int* 2011, 108(6):81-86.

61. Bornstein J, Ohel G, Sorokin Y, Reape KZ, Shnaider O, Kessary-Shoham H, Ophir E: Effectiveness of a novel home-based testing device for the detection of rupture of membranes. *Am J Perinatol* 2009, 26(1):45-50.

62. Boryri T, Navidian A, Zehi F: Assessing the effect of self-care education on anxiety and depression among pregnant women with a history of spontaneous abortion. In., vol. 9; 2020.

63. Bowen L, Pealing L, Tucker K, McManus RJ, Chappell LC: Adherence with blood pressure self-monitoring in women with pregnancy hypertension, and comparisons to clinic readings: A secondary analysis of OPTIMUM-BP. *Pregnancy Hypertens* 2021, 25:68-74.

64. Brennan M, Young G, Devane D: Topical preparations for preventing stretch marks in pregnancy. *Cochrane Database Syst Rev* 2012, 11:CD000066.

65. Bright KS, Mughal MK, Wajid A, Lane-Smith M, Murray L, Roy N, Van Zanten SV, McNeil DA, Stuart S, Kingston D: Internet-based interpersonal psychotherapy for stress, anxiety, and depression in prenatal women: study protocol for a pilot randomized controlled trial. *Trials* 2019, 20(1):814.

66. Brik M, Fernandez-Buhigas I, Martin-Arias A, Vargas-Terrones M, Barakat R, Santacruz B: Does exercise during pregnancy impact on maternal weight gain and fetal cardiac function? A randomized controlled trial. *Ultrasound Obstet Gynecol* 2019, 53(5):583-589.

67. Brown J, Alwan NA, West J, Brown S, McKinlay CJD, Farrar D, Crowther CA: Lifestyle interventions for the treatment of women with gestational diabetes. In., vol. 2017; 2017.

68. Brustman LE, Langer O, Anyaegbunam A, Belle C, Merkatz IR: Education does not improve patient perception of preterm uterine contractility. *International Journal of Gynecology & Obstetrics* 1991, 34(3):290-290.

69. Buppasiri P, Lumbiganon P, Thinkhamrop J, Ngamjarus C, Laopaiboon M, Medley N: Calcium supplementation (other than for preventing or treating hypertension) for improving pregnancy and infant outcomes. In., vol. 2015; 2015.

70. Byrne J, Hauck Y, Fisher C, Bayes S, Schutze R: Effectiveness of a Mindfulness-Based Childbirth Education pilot study on maternal self-efficacy and fear of childbirth. *J Midwifery Womens Health* 2014, 59(2):192-197.

71. Cahill AG, Haire-Joshu D, Cade WT, Stein RI, Woolfolk CL, Moley K, Mathur A, Schechtman K, Klein S: Weight Control Program and Gestational Weight Gain in Disadvantaged Women with Overweight or Obesity: A Randomized Clinical Trial. *Obesity (Silver Spring)* 2018, 26(3):485-491.

72. Callaghan S: The role of fetal movement counting and 'kick charts' to reduce stillbirths in pregnancies ≥28 weeks' gestation. In., vol. 26; 2018.

73. Callaway LK, McIntyre HD, Barrett HL, Foxcroft K, Tremellen A, Lingwood BE, Tobin JM, Wilkinson S, Kothari A, Morrison M *et al*: Probiotics for the Prevention of Gestational Diabetes Mellitus in Overweight and Obese Women: Findings From the SPRING Double-Blind Randomized Controlled Trial. *Diabetes Care* 2019, 42(3):364-371.

For peer review only

74. Can HO, Saruhan A: Evaluation of the effects of ice massage applied to large intestine 4 (hegu) on postpartum pain during the active phase of labor. *Iran J Nurs Midwifery Res* 2015, 20(1):129-138.

75. Cankaya S, Simsek B: Effects of Antenatal Education on Fear of Birth, Depression, Anxiety, Childbirth Self-Efficacy, and Mode of Delivery in Primiparous Pregnant Women: A Prospective Randomized Controlled Study. *Clin Nurs Res* 2021, 30(6):818-829.

76. Carducci B, Keats EC, Bhutta ZA: Zinc supplementation for improving pregnancy and infant outcome. In., vol. 2021; 2021.

77. Carlson LM, Harris S, Hardisty EE, Hocutt G, Vargo D, Campbell E, Davis E, Gilmore K, Vora NL: Use of a novel computerized decision aid for aneuploidy screening: a randomized controlled trial. *Genet Med* 2019, 21(4):923-929.

78. Carrascosa MDC, Navas A, Artigues C, Ortas S, Portells E, Soler A, Bennasar-Veny M, Leiva A, Aquanatal T: Effect of aerobic water exercise during pregnancy on epidural use and pain: A multi-centre, randomised, controlled trial. *Midwifery* 2021, 103:103105.

79. Chamberlain C, O'Mara-Eves A, Porter J, Coleman T, Perlen SM, Thomas J, McKenzie JE: Psychosocial interventions for supporting women to stop smoking in pregnancy. In., vol. 2017; 2017.

80. Chang G, McNamara TK, Orav EJ, Koby D, Lavigne A, Ludman B, Vincitorio NA, Wilkins-Haug L: Brief intervention for prenatal alcohol use: a randomized trial. *Obstet Gynecol* 2005, 105(5 Pt 1):991-998.

81. Chankhunaphas W, Charoenkwan K: Effect of elastic abdominal binder on pain and functional recovery after caesarean delivery: a randomised controlled trial. *J Obstet Gynaecol* 2020, 40(4):473-478.

82. Chao AS, Chao A, Wang TH, Chang YC, Peng HH, Chang SD, Chao A, Chang CJ, Lai CH, Wong AMK: Pain relief by applying transcutaneous electrical nerve stimulation (TENS) on acupuncture points during the first stage of labor: a randomized double-blind placebo-controlled trial. *Pain* 2007, 127(3):214-220.

83. Charkamyani F, Hosseinkhani A, Neisani Samani L, Khedmat L: Reducing the Adverse Maternal and Fetal Outcomes in IVF Women by Exercise Interventions During Pregnancy. *Res Q Exerc Sport* 2019, 90(4):589-599.

84. Chatzakis C, Goulis DG, Mareti E, Eleftheriades M, Zavlanos A, Dinas K, Sotiriadis A: Prevention of gestational diabetes mellitus in overweight or obese pregnant women: A network meta-analysis. In., vol. 158; 2019.

85. Chen I, Opiyo N, Tavender E, Mortazhejri S, Rader T, Petkovic J, Yogasingam S, Taljaard M, Agarwal S, Laopaiboon M *et al*: Non-clinical interventions for reducing unnecessary caesarean section. *Cochrane Database Syst Rev* 2018, 9:CD005528.

86. Chen M, Chang Q, Duan T, He J, Zhang L, Liu X: Uterine massage to reduce blood loss after vaginal delivery: a randomized controlled trial. *Obstet Gynecol* 2013, 122(2 Pt 1):290-295.

87. Chen Y, Yue R, Zhang B, Li Z, Shui J, Huang X: Effects of probiotics on blood glucose, biomarkers of inflammation and oxidative stress in pregnant women with gestational diabetes mellitus: A meta-analysis of randomized controlled trials. *Med Clin (Barc)* 2020, 154(6):199-206.

88. Chi YC, Wu CL, Chen CY, Lyu SY, Lo FE, Morisky DE: Randomized trial of a secondhand smoke exposure reduction intervention among hospital-based pregnant women. *Addict Behav* 2015, 41:117-123.

89. Chirakalwasan N, Amnakkittikul S, Wanitcharoenkul E, Charoensri S, Saetung S, Chanprasertyothin S, Chailurkit LO, Panburana P, Bumrungphuet S, Thakkinstian A *et al*: Continuous Positive Airway Pressure Therapy in Gestational Diabetes With Obstructive Sleep Apnea: A Randomized Controlled Trial. *J Clin Sleep Med* 2018, 14(3):327-336.

90. Choi HS, Lee KE, Shin Y: Effects of an emotional regulation program on maladaptive cognitive emotion regulation, stress, anxiety, and postpartum depression among South Korean mothers of premature infants in the neonatal intensive care unit. *Child Health Nurs Res* 2021, 27(1):24-33.

91. Choi J, Lee JH, Vittinghoff E, Fukuoka Y: mHealth Physical Activity Intervention: A Randomized Pilot Study in Physically Inactive Pregnant Women. *Matern Child Health J* 2016, 20(5):1091-1101.

For peer review only

92. Christian P, Klemm R, Shamim AA, Ali H, Rashid M, Shaikh S, Wu L, Mehra S, Labrique A, Katz J *et al*: Effects of vitamin A and beta-carotene supplementation on birth size and length of gestation in rural Bangladesh: a cluster-randomized trial. *Am J Clin Nutr* 2013, 97(1):188-194.
93. Chughtai A, Navaee M, Hadi M, Yaghoubinia F: Comparing the Effect of Aromatherapy With Essential Oils of Rosa damascena and Lavender Alone and in Combination on Severity of Pain in the First Phase of Labor in Primiparous Women. 2018.
94. Clasen T, Checkley W, Peel JL, Balakrishnan K, McCracken JP, Rosa G, Thompson LM, Barr DB, Clark ML, Johnson MA *et al*: Design and Rationale of the HAPIN Study: A Multicountry Randomized Controlled Trial to Assess the Effect of Liquefied Petroleum Gas Stove and Continuous Fuel Distribution. *Environ Health Perspect* 2020, 128(4):47008.
95. Cluett ER, Burns E, Cuthbert A: Immersion in water during labour and birth. In., vol. 2018; 2018.
96. Coentro VS, Perrella SL, Lai CT, Rea A, Murray K, Geddes DT: Impact of Nipple Shield Use on Milk Transfer and Maternal Nipple Pain. *Breastfeed Med* 2021, 16(3):222-229.
97. Coleman J, Okere M, Seffah J, Kember A, O'Brien LM, Borazjani A, Butler M, Wells J, MacRitchie S, Isaac A *et al*: The Ghana PrenaBelt trial: a double-blind, sham-controlled, randomised clinical trial to evaluate the effect of maternal positional therapy during third-trimester sleep on birth weight. *BMJ Open* 2019, 9(4):e022981.
98. Coleman T, Clark M, Welch C, Whitemore R, Leonardi-Bee J, Cooper S, Hewitt C, Jones M, Sutton S, Watson J *et al*: Effectiveness of offering tailored text message, self-help smoking cessation support to pregnant women who want information on stopping smoking: MiQuit3 randomised controlled trial and meta-analysis. *Addiction* 2022, 117(4):1079-1094.
99. Crawford TJ, Crowther CA, Alsweiler J, Brown J: Antenatal dietary supplementation with myo-inositol in women during pregnancy for preventing gestational diabetes. In., vol. 2015; 2015.
100. Crepinsek MA, Taylor EA, Michener K, Stewart F: Interventions for preventing mastitis after childbirth. In., vol. 2020; 2020.
101. Crockett K, Zlotnick C, Davis M, Payne N, Washington R: A depression preventive intervention for rural low-income African-American pregnant women at risk for postpartum depression. *Arch Womens Ment Health* 2008, 11(5-6):319-325.
102. Cronin RS, Li M, Thompson JMD, Gordon A, Raynes-Greenow CH, Heazell AEP, Stacey T, Culling VM, Bowring V, Anderson NH *et al*: An Individual Participant Data Meta-analysis of Maternal Going-to-Sleep Position, Interactions with Fetal Vulnerability, and the Risk of Late Stillbirth. *EClinicalMedicine* 2019, 10:49-57.
103. Czech I, Fuchs P, Fuchs A, Lorek M, Tobolska-Lorek D, Drosdzol-Cop A, Sikora J: Pharmacological and Non-Pharmacological Methods of Labour Pain Relief-Establishment of Effectiveness and Comparison. *Int J Environ Res Public Health* 2018, 15(12).
104. D'Almeida A, Carter JP, Anatol A, Prost C: Effects of a combination of evening primrose oil (gamma linolenic acid) and fish oil (eicosapentaenoic + docahexaenoic acid) versus magnesium, and versus placebo in preventing pre-eclampsia. *Women Health* 1992, 19(2-3):117-131.
105. da Silva SG, Hallal PC, Domingues MR, Bertoldi AD, Silveira MFD, Bassani D, da Silva ICM, da Silva BGC, Coll CVN, Evenson K: A randomized controlled trial of exercise during pregnancy on maternal and neonatal outcomes: results from the PAMELA study. *Int J Behav Nutr Phys Act* 2017, 14(1):175.
106. Dabiri F, Shahi A: The Effect of LI4 Acupressure on Labor Pain Intensity and Duration of Labor: A Randomized Controlled Trial. *Oman Med J* 2014, 29(6):425-429.
107. Dahlen HG, Homer CS, Cooke M, Upton AM, Nunn R, Brodrick B: Perineal outcomes and maternal comfort related to the application of perineal warm packs in the second stage of labor: a randomized controlled trial. *Birth* 2007, 34(4):282-290.
108. Dai L, Shen Q, Redding SR, Ouyang YQ: Simulation-based childbirth education for Chinese primiparas: A pilot randomized controlled trial. *Patient Educ Couns* 2021, 104(9):2266-2274.

For peer review only

109. Daley AJ, Macarthur C, Winter H: The role of exercise in treating postpartum depression: a review of the literature. *J Midwifery Womens Health* 2007, 52(1):56-62.

110. Dashtinejad E, Abedi P, Afshari P: Comparison of the effect of breast pump stimulation and oxytocin administration on the length of the third stage of labor, postpartum hemorrhage, and anemia: a randomized controlled trial. *BMC Pregnancy Childbirth* 2018, 18(1):293.

111. Davidson SJ, Barrett HL, Price SA, Callaway LK, Dekker Nitert M: Probiotics for preventing gestational diabetes. In., vol. 2021; 2021.

112. de Vries H, Bakker M, Mullen PD, van Breukelen G: The effects of smoking cessation counseling by midwives on Dutch pregnant women and their partners. *Patient Educ Couns* 2006, 63(1-2):177-187.

113. Dehcheshmeh FS, Rafiei H: Complementary and alternative therapies to relieve labor pain: A comparative study between music therapy and Hoku point ice massage. *Complement Ther Clin Pract* 2015, 21(4):229-232.

114. Deliktas A, Kukulu K: A meta-analysis of the effect on maternal health of upright positions during the second stage of labour, without routine epidural analgesia. In., vol. 74; 2018.

115. Demirel G, Guler H: The Effect of Uterine and Nipple Stimulation on Induction With Oxytocin and the Labor Process. *Worldviews Evid Based Nurs* 2015, 12(5):273-280.

116. Dennis CL, Dowswell T: Interventions (other than pharmacological, psychosocial or psychological) for treating antenatal depression. In., vol. 2013; 2013.

117. Dennis CL, Jackson K, Watson J: Interventions for treating painful nipples among breastfeeding women. In., vol. 2014; 2014.

118. Dennis CL, Schottle N, Hodnett E, McQueen K: An all-purpose nipple ointment versus lanolin in treating painful damaged nipples in breastfeeding women: a randomized controlled trial. *Breastfeed Med* 2012, 7(6):473-479.

119. Dennis CLE: Treatment of postpartum depression, Part 2: A critical review of nonbiological interventions. In., vol. 65; 2004.

120. Dennis CLE: Preventing postpartum depression part I: A review of biological interventions. In., vol. 49; 2004.

121. Desai V, Kozyrskyj AL, Lau S, Sanni O, Dennett L, Walter J, Ospina MB: Effectiveness of Probiotic, Prebiotic, and Synbiotic Supplementation to Improve Perinatal Mental Health in Mothers: A Systematic Review and Meta-Analysis. In., vol. 12; 2021.

122. Dettinger JC, Kinuthia J, Pintye J, Abuna F, Begnel E, Mugwanya K, Sila J, Lagat H, Baeten JM, John-Stewart G: Perinatal outcomes following maternal pre-exposure prophylaxis (PrEP) use during pregnancy: results from a large PrEP implementation program in Kenya. *J Int AIDS Soc* 2019, 22(9):e25378.

123. Deussen AR, Ashwood P, Martis R, Stewart F, Grzeskowiak LE: Relief of pain due to uterine cramping/involution after birth. In., vol. 2020; 2020.

124. Diaz V, Abalos E, Carroli G: Methods for blood loss estimation after vaginal birth. In., vol. 2018; 2018.

125. DiClemente RJ, Wingood GM, Rose E, Sales JM, Crosby RA: Evaluation of an HIV/STD sexual risk-reduction intervention for pregnant African American adolescents attending a prenatal clinic in an urban public hospital: preliminary evidence of efficacy. *J Pediatr Adolesc Gynecol* 2010, 23(1):32-38.

126. DiMarco MA, Menke EM, McNamara T: Evaluating a support group for perinatal loss. *MCN Am J Matern Child Nurs* 2001, 26(3):135-140.

127. Dineva M, Fishpool H, Rayman MP, Mendis J, Bath SC: Systematic review and meta-analysis of the effects of iodine supplementation on thyroid function and child neurodevelopment in mildly-to-moderately iodine-deficient pregnant women. In., vol. 112; 2020.

128. Dodd JM, Turnbull D, McPhee AJ, Deussen AR, Grivell RM, Yelland LN, Crowther CA, Wittert G, Owens JA, Robinson JS *et al*: Antenatal lifestyle advice for women who are overweight or obese: LIMIT randomised trial. *BMJ* 2014, 348:g1285.

129. Dodd V, Chalmers C: Comparing the use of hydrogel dressings to lanolin ointment with lactating mothers. *J Obstet Gynecol Neonatal Nurs* 2003, 32(4):486-494.

For peer review only

130. Doering JJ, Dogan S: A Postpartum Sleep and Fatigue Intervention Feasibility Pilot Study. *Behav Sleep Med* 2018, 16(2):185-201.

131. Dokmak F, Michalek IM, Boulvain M, Desseauve D: Squatting position in the second stage of labor: A systematic review and meta-analysis. In., vol. 254; 2020.

132. Dougall G, Franssen M, Tucker KL, Yu LM, Hinton L, Rivero-Arias O, Abel L, Allen J, Band RJ, Chisholm A *et al*: Blood pressure monitoring in high-risk pregnancy to improve the detection and monitoring of hypertension (the BUMP 1 and 2 trials): protocol for two linked randomised controlled trials. *BMJ Open* 2020, 10(1):e034593.

133. Downe S, Finlayson K, Melvin C, Spiby H, Ali S, Diggle P, Gyte G, Hinder S, Miller V, Slade P *et al*: Self-hypnosis for intrapartum pain management in pregnant nulliparous women: a randomised controlled trial of clinical effectiveness. *BJOG* 2015, 122(9):1226-1234.

134. Downs DS, Dinallo JM, Birch LL, Paul IM, Ulbrecht JS: Randomized Face-to-Face vs. Home Exercise Interventions in Pregnant Women with Gestational Diabetes. *Psychol Sport Exerc* 2017, 30:73-81.

135. Downs DS, Savage JS, Rivera DE, Pauley AM, Leonard KS, Hohman EE, Guo P, McNitt KM, Stetter C, Kunselman A: Adaptive, behavioral intervention impact on weight gain, physical activity, energy intake, and motivational determinants: results of a feasibility trial in pregnant women with overweight/obesity. *J Behav Med* 2021, 44(5):605-621.

136. Draffin CR, Alderdice FA, McCance DR, Maresh M, Harper R, Patterson CC, Bernatavicius G, Brennan SF, Gough A, McSorley O *et al*: Impact of an educational DVD on anxiety and glycaemic control in women diagnosed with gestational diabetes mellitus (GDM): A randomised controlled trial. *Diabetes Res Clin Pract* 2017, 126:164-171.

137. Dritsa M, Dupuis G, Lowensteyn I, Da Costa D: Effects of home-based exercise on fatigue in postpartum depressed women: who is more likely to benefit and why? *J Psychosom Res* 2009, 67(2):159-163.

138. Dugas M, Shorten A, Dube E, Wassef M, Bujold E, Chaillet N: Decision aid tools to support women's decision making in pregnancy and birth: a systematic review and meta-analysis. *Soc Sci Med* 2012, 74(12):1968-1978.

139. Duncan LG, Cohn MA, Chao MT, Cook JG, Riccobono J, Bardacke N: Benefits of preparing for childbirth with mindfulness training: a randomized controlled trial with active comparison. *BMC Pregnancy Childbirth* 2017, 17(1):140.

140. Dutra LRDV, Araújo AMPdH, Micussi MTABC: Non-pharmacological therapies for postpartum analgesia: a systematic review. *Brazilian Journal Of Pain* 2019, 2(1).

141. East CE, Dolan WJ, Forster DA: Antenatal breast milk expression by women with diabetes for improving infant outcomes. In., vol. 2014; 2014.

142. East CE, Dorward ED, Whale RE, Liu J: Local cooling for relieving pain from perineal trauma sustained during childbirth. *Cochrane Database Syst Rev* 2020, 10(10):CD006304.

143. Ebrahimian A, Rahmani Bilandi R: Comparisons of the Effects of Watching Virtual Reality Videos and Chewing Gum on the Length of Delivery Stages and Maternal Childbirth Satisfaction: A Randomized Controlled Trial. *Iran J Med Sci* 2021, 46(1):15-22.

144. Edwards MJ, Geiser T, Chafin C, Weatherby NL, Smith CM: S.M.A.R.T. mothers are resisting tobacco: prenatal smoking cessation in WIC mothers. *J Allied Health* 2009, 38(3):170-176.

145. Ehrlich SF, Hedderson MM, Brown SD, Sternfeld B, Chasan-Taber L, Feng J, Adams J, Ching J, Crites Y, Quesenberry CP *et al*: Moderate intensity sports and exercise is associated with glycaemic control in women with gestational diabetes. *Diabetes Metab* 2017, 43(5):416-423.

146. Ejike BU, Ohaeri CC, Amaechi EC: Home management practices and its impact on malaria prevalence amongst pregnant women in South-Eastern Nigeria. *Asian Pacific Journal of Tropical Disease* 2017, 7(2):68-70.

147. Ekhtiari YS, Majlessi F, Foroushani AR, Shakibazadeh E: Effect of a self-care educational program based on the health belief model on reducing low birth weight among pregnant Iranian women. *International Journal of Preventive Medicine* 2014, 5(1).

For peer review only

148. El-Rafie MM, Khafagy GM, Gamal MG: Effect of aerobic exercise during pregnancy on antenatal depression. *Int J Womens Health* 2016, 8:53-57.
149. Epidural, Position Trial Collaborative G: Upright versus lying down position in second stage of labour in nulliparous women with low dose epidural: BUMPES randomised controlled trial. *BMJ* 2017, 359:j4471.
150. Erchick DJ, Agrawal NK, Khatry SK, Katz J, LeClerq SC, Reynolds MA, Mullany LC: Adherence to and acceptability of three alcohol-free, antiseptic oral rinses: A community-based pilot randomized controlled trial among pregnant women in rural Nepal. *Community Dent Oral Epidemiol* 2020, 48(6):501-512.
151. Ershoff DH, Mullen PD, Quinn VP: A randomized trial of a serialized self-help smoking cessation program for pregnant women in an HMO. *Am J Public Health* 1989, 79(2):182-187.
152. Ershoff DH, Quinn VP, Boyd NR, Stern J, Gregory M, Wirtschafter D: The Kaiser Permanente prenatal smoking-cessation trial11The full text of this article is available via AJPM Online at http://www.elsevier.com/locate/ajpmonline. *American Journal of Preventive Medicine* 1999, 17(3):161-168.
153. Espersen T, Klebe JG: Self-monitoring of blood glucose in pregnant diabetics. A comparative study of the blood glucose level and course of pregnancy in pregnant diabetics on an out-patient regime before and after the introduction of methods for home analysis of blood glucose. *Acta Obstet Gynecol Scand* 1985, 64(1):11-14.
154. Facchinetti F, Pedrielli G, Benoni G, Joppi M, Verlato G, Dante G, Balduzzi S, Cuzzolin L: Herbal supplements in pregnancy: unexpected results from a multicentre study. *Hum Reprod* 2012, 27(11):3161-3167.
155. Fathi-Ashtiani A, Ahmadi A, Ghobari-Bonab B, Azizi MP, Saheb-Alzamani SM: Randomized Trial of Psychological Interventions to Preventing Postpartum Depression among Iranian First-time Mothers. *Int J Prev Med* 2015, 6:109.
156. Feder L, Niolon PH, Campbell J, Whitaker DJ, Brown J, Rostad W, Bacon S: An Intimate Partner Violence Prevention Intervention in a Nurse Home Visitation Program: A Randomized Clinical Trial. *J Womens Health (Larchmt)* 2018, 27(12):1482-1490.
157. Feng G, Simpson JA, Chaluluka E, Molyneux ME, Rogerson SJ: Decreasing burden of malaria in pregnancy in Malawian women and its relationship to use of intermittent preventive therapy or bed nets. *PLoS One* 2010, 5(8):e12012.
158. Fernandez L, Cardenas N, Arroyo R, Manzano S, Jimenez E, Martin V, Rodriguez JM: Prevention of Infectious Mastitis by Oral Administration of Lactobacillus salivarius PS2 During Late Pregnancy. *Clin Infect Dis* 2016, 62(5):568-573.
159. Field T, Diego M, Delgado J, Medina L: Yoga and social support reduce prenatal depression, anxiety and cortisol. *J Bodyw Mov Ther* 2013, 17(4):397-403.
160. Flannery C, Fredrix M, Olander EK, McAuliffe FM, Byrne M, Kearney PM: Effectiveness of physical activity interventions for overweight and obesity during pregnancy: A systematic review of the content of behaviour change interventions. In., vol. 16; 2019.
161. Fogarty S, McInerney C, Hay P: Pregnancy-related Pelvic Girdle Pain and Pregnancy Massage: Findings from a Subgroup Analysis of an Observational Study. *Int J Ther Massage Bodywork* 2020, 13(2):1-8.
162. Fokam EB, Ngimuh L, Anchang-Kimbi JK, Wanji S: Assessment of the usage and effectiveness of intermittent preventive treatment and insecticide-treated nets on the indicators of malaria among pregnant women attending antenatal care in the Buea Health District, Cameroon. *Malar J* 2016, 15(1):172.
163. Foudil-Bey I, Murphy MSQ, Dunn S, Keely EJ, El-Chaar D: Evaluating antenatal breastmilk expression outcomes: a scoping review. *Int Breastfeed J* 2021, 16(1):25.
164. Franciscatto LG, Silva CM, Barcellos RB, Angeli S, Silva MS, Almeida SE, Rossetti ML: Comparison of urine and self-collected vaginal samples for detecting human papillomavirus DNA in pregnant women. *Int J Gynaecol Obstet* 2014, 125(1):69-72.
165. Fritel X, de Tayrac R, Bader G, Savary D, Gueye A, Deffieux X, Fernandez H, Richet C, Guilhot J, Fauconnier A: Preventing Urinary Incontinence With Supervised Prenatal Pelvic Floor Exercises: A Randomized Controlled Trial. *Obstet Gynecol* 2015, 126(2):370-377.

For peer review only

166. Fujioka N, Kobayashi T, Turale S: Short-term behavioral changes in pregnant women after a quit-smoking program via e-learning: a descriptive study from Japan. *Nurs Health Sci* 2012, 14(3):304-311.

167. Gallo RBS, Santana LS, Marcolin AC, Duarte G, Quintana SM: Sequential application of non-pharmacological interventions reduces the severity of labour pain, delays use of pharmacological analgesia, and improves some obstetric outcomes: a randomised trial. *J Physiother* 2018, 64(1):33-40.

168. Gamble C, Ekwaru JP, ter Kuile FO: Insecticide-treated nets for preventing malaria in pregnancy. *Cochrane Database Syst Rev* 2006(2):CD003755.

169. Garmendia ML, Casanello P, Flores M, Kusanovic JP, Uauy R: The effects of a combined intervention (docosahexaenoic acid supplementation and home-based dietary counseling) on metabolic control in obese and overweight pregnant women: the MIGHT study. *Am J Obstet Gynecol* 2021, 224(5):526 e521-526 e525.

170. Garnaes KK, Morkved S, Salvesen KA, Salvesen O, Moholdt T: Exercise training during pregnancy reduces circulating insulin levels in overweight/obese women postpartum: secondary analysis of a randomised controlled trial (the ETIP trial). *BMC Pregnancy Childbirth* 2018, 18(1):18.

171. Garnweidner-Holme L, Henriksen L, Torheim LE, Lukasse M: Effect of the Pregnant+ Smartphone App on the Dietary Behavior of Women With Gestational Diabetes Mellitus: Secondary Analysis of a Randomized Controlled Trial. *JMIR Mhealth Uhealth* 2020, 8(11):e18614.

172. Gau ML, Chang CY, Tian SH, Lin KC: Effects of birth ball exercise on pain and self-efficacy during childbirth: a randomised controlled trial in Taiwan. *Midwifery* 2011, 27(6):e293-300.

173. Gedde-Dahl M, Fors EA: Impact of self-administered relaxation and guided imagery techniques during final trimester and birth. *Complement Ther Clin Pract* 2012, 18(1):60-65.

174. Geisinger ML, Geurs NC, Bain JL, Kaur M, Vassilopoulos PJ, Cliver SP, Hauth JC, Reddy MS: Oral health education and therapy reduces gingivitis during pregnancy. *J Clin Periodontol* 2014, 41(2):141-148.

175. Germeroth LJ, Benno MT, Kolko Conlon RP, Emery RL, Cheng Y, Grace J, Salk RH, Levine MD: Trial design and methodology for a non-restricted sequential multiple assignment randomized trial to evaluate combinations of perinatal interventions to optimize women's health. *Contemp Clin Trials* 2019, 79:111-121.

176. Ghaffari M, Rakhshanderou S, Safari-Moradabadi A, Torabi S: Oral and dental health care during pregnancy: Evaluating a theory-driven intervention. *Oral Dis* 2018, 24(8):1606-1614.

177. Ghahremani L, Alipoor M, Amoee S, Keshavarzi S: Health Promoting Behaviors and Self-efficacy of Physical Activity During Pregnancy: An Interventional Study. *International Journal of Women's Health and Reproduction Sciences* 2017, 5(3):181-185.

178. Ghiasvand F, Riazi H, Hajian S, Kazemi E, Firoozi A: The effect of a self-care program based on the teach back method on the postpartum quality of life. *Electron Physician* 2017, 9(4):4180-4189.

179. Ghouri F, Hollywood A, Ryan K: A systematic review of non-antibiotic measures for the prevention of urinary tract infections in pregnancy. *BMC Pregnancy Childbirth* 2018, 18(1):99.

180. Gielen AC, Windsor R, Faden RR, O'Campo P, Repke J, Davis M: Evaluation of a smoking cessation intervention for pregnant women in an urban prenatal clinic. *Health Educ Res* 1997, 12(2):247-254.

181. Gilad R, Hochner H, Savitsky B, Porat S, Hochner-Celnikier D: Castor oil for induction of labor in post-date pregnancies: A randomized controlled trial. *Women Birth* 2018, 31(1):e26-e31.

182. Gilbert L, Gross J, Lanzi S, Quansah DY, Puder J, Horsch A: How diet, physical activity and psychosocial well-being interact in women with gestational diabetes mellitus: an integrative review. *BMC Pregnancy Childbirth* 2019, 19(1):60.

183. Gilinsky A, Swanson V, Power K: Interventions delivered during antenatal care to reduce alcohol consumption during pregnancy: A systematic review. In., vol. 19; 2011.

184. Gilinsky AS, Dale H, Robinson C, Hughes AR, McInnes R, Lavallee D: Efficacy of physical activity interventions in post-natal populations: systematic review, meta-analysis and content coding of behaviour change techniques. *Health Psychol Rev* 2015, 9(2):244-263.

For peer review only

185. Gille C, Boer B, Marschal M, Urschitz MS, Heinecke V, Hund V, Speidel S, Tarnow I, Mylonas I, Franz A *et al*: Effect of probiotics on vaginal health in pregnancy. EFFPRO, a randomized controlled trial. *Am J Obstet Gynecol* 2016, 215(5):608 e601-608 e607.
186. Godfrey KM, Barton SJ, El-Heis S, Kenealy T, Nield H, Baker PN, Chong YS, Cutfield W, Chan SY, Ni PSG: Myo-Inositol, Probiotics, and Micronutrient Supplementation From Preconception for Glycemia in Pregnancy: NiPPeR International Multicenter Double-Blind Randomized Controlled Trial. *Diabetes Care* 2021, 44(5):1091-1099.
187. Goetz M, Schiele C, Muller M, Matthies LM, Deutsch TM, Spano C, Graf J, Zipfel S, Bauer A, Brucker SY *et al*: Effects of a Brief Electronic Mindfulness-Based Intervention on Relieving Prenatal Depression and Anxiety in Hospitalized High-Risk Pregnant Women: Exploratory Pilot Study. *J Med Internet Res* 2020, 22(8):e17593.
188. Goksin I, Ayaz-Alkaya S: The effect of progressive muscle relaxation on the postpartum depression risk and general comfort levels in primiparas. *Stress Health* 2020, 36(3):322-329.
189. Gomes F, King SE, Dallmann D, Golan J, da Silva ACF, Hurley KM, Bergeron G, Bourassa MW, Mehta S: Interventions to increase adherence to micronutrient supplementation during pregnancy: a systematic review. *Ann N Y Acad Sci* 2021, 1493(1):41-58.
190. Goodman JH, Guarino A, Chenausky K, Klein L, Prager J, Petersen R, Forget A, Freeman M: CALM Pregnancy: results of a pilot study of mindfulness-based cognitive therapy for perinatal anxiety. *Arch Womens Ment Health* 2014, 17(5):373-387.
191. Grant SM, Wolever TM, O'Connor DL, Nisenbaum R, Josse RG: Effect of a low glycaemic index diet on blood glucose in women with gestational hyperglycaemia. *Diabetes Res Clin Pract* 2011, 91(1):15-22.
192. Green SM, Donegan E, McCabe RE, Streiner DL, Agako A, Frey BN: Cognitive behavioral therapy for perinatal anxiety: A randomized controlled trial. *Aust N Z J Psychiatry* 2020, 54(4):423-432.
193. Grenvik JM, Rosenthal E, Saccone G, Della Corte L, Quist-Nelson J, Gerkin RD, Gimovsky AC, Kwan M, Mercier R, Berghella V: Peanut ball for decreasing length of labor: A systematic review and meta-analysis of randomized controlled trials. *Eur J Obstet Gynecol Reprod Biol* 2019, 242:159-165.
194. Griffith RJ, Alsweiler J, Moore AE, Brown S, Middleton P, Shepherd E, Crowther CA: Interventions to prevent women from developing gestational diabetes mellitus: an overview of Cochrane Reviews. In., vol. 2020; 2020.
195. Griffiths SE, Parsons J, Naughton F, Fulton EA, Tombor I, Brown KE: Are digital interventions for smoking cessation in pregnancy effective? A systematic review and meta-analysis. In., vol. 12; 2018.
196. Grzeskowiak LE, Smith B, Roy A, Dekker GA, Clifton VL: An observational study of the impact of an antenatal asthma management service on asthma control during pregnancy. *Eur J Obstet Gynecol Reprod Biol* 2016, 197:48-53.
197. Guelfi KJ, Ong MJ, Crisp NA, Fournier PA, Wallman KE, Grove JR, Doherty DA, Newnham JP: Regular Exercise to Prevent the Recurrence of Gestational Diabetes Mellitus: A Randomized Controlled Trial. In*: 2016*.
198. Gunther J, Hoffmann J, Kunath J, Spies M, Meyer D, Stecher L, Rosenfeld E, Kick L, Rauh K, Hauner H: Effects of a Lifestyle Intervention in Routine Care on Prenatal Dietary Behavior-Findings from the Cluster-Randomized GeliS Trial. *J Clin Med* 2019, 8(7).
199. Guo W, Zhang B, Wang X: Lifestyle interventions for gestational diabetes mellitus to control blood glucose: a meta-analysis of randomized studies. *International Journal of Diabetes in Developing Countries* 2017, 38(1):26-35.
200. Gupta JK, Sood A, Hofmeyr GJ, Vogel JP: Position in the second stage of labour for women without epidural anaesthesia. In., vol. 2017; 2017.
201. Gustafsson MK, Romundstad PR, Stafne SN, Helvik AS, Stunes AK, Morkved S, Salvesen KA, Thorsby PM, Mosti MP, Syversen U: The effect of an exercise program in pregnancy on vitamin D status among healthy, pregnant Norwegian women: a randomized controlled trial. *BMC Pregnancy Childbirth* 2019, 19(1):76.
202. Gutke A, Betten C, Degerskär K, Pousette S, Fagevik Olsén M: Treatments for pregnancy-related lumbopelvic pain: A systematic review of physiotherapy modalities. In., vol. 94; 2015.

For peer review only

203. Hagan R, Evans SF, Pope S: Preventing postnatal depression in mothers of very preterm infants: a randomised controlled trial. *BJOG* 2004, 111(7):641-647.

204. Haghighat M, Mirghafourvand M, Mohammad-Alizadeh-Charandabi S, Malakouti J, Erfani M: The Effect of Spiritual Counseling on Stress and Anxiety in Pregnancy: A Randomized Controlled Clinical Trial. *Iranian Red Crescent Medical Journal* 2018, In Press(In Press).

205. Haire-Joshu D, Cahill AG, Stein RI, Cade WT, Woolfolk CL, Moley K, Mathur A, Schwarz CD, Schechtman KB, Klein S: Randomized Controlled Trial of Home-Based Lifestyle Therapy on Postpartum Weight in Underserved Women with Overweight or Obesity. *Obesity (Silver Spring)* 2019, 27(4):535-541.

206. Hajek P, West R, Lee A, Foulds J, Owen L, Eiser JR, Main N: Randomized controlled trial of a midwife-delivered brief smoking cessation intervention in pregnancy. *Addiction* 2001, 96(3):485-494.

207. Halse RE, Wallman KE, Dimmock JA, Newnham JP, Guelfi KJ: Home-Based Exercise Improves Fitness and Exercise Attitude and Intention in Women with GDM. *Med Sci Sports Exerc* 2015, 47(8):1698-1704.

208. Halvorsen S, Haakstad LA, Edvardsen E, Bo K: Effect of aerobic dance on cardiorespiratory fitness in pregnant women: a randomised controlled trial. *Physiotherapy* 2013, 99(1):42-48.

209. Hannover W, Thyrian JR, Roske K, Grempler J, Rumpf HJ, John U, Hapke U: Smoking cessation and relapse prevention for postpartum women: results from a randomized controlled trial at 6, 12, 18 and 24 months. *Addict Behav* 2009, 34(1):1-8.

210. Harding KB, Peña-Rosas JP, Webster AC, Yap CMY, Payne BA, Ota E, De-Regil LM: Iodine supplementation for women during the preconception, pregnancy and postpartum period. In., vol. 2017; 2017.

211. Hardy I, Lloyd A, Morisset AS, Camirand Lemyre F, Baillargeon JP, Fraser WD: Healthy for My Baby Research Protocol- a Randomized Controlled Trial Assessing a Preconception Intervention to Improve the Lifestyle of Overweight Women and Their Partners. *Front Public Health* 2021, 9:670304.

212. Harrison CL, Lombard CB, Teede HJ: Limiting postpartum weight retention through early antenatal intervention: the HeLP-her randomised controlled trial. *Int J Behav Nutr Phys Act* 2014, 11(1):134.

213. Haruna M, Matsuzaki M, Ota E, Shiraishi M, Hanada N, Mori R: Guided imagery for treating hypertension in pregnancy. *Cochrane Database Syst Rev* 2019, 4(4):CD011337.

214. Haugland KS, Rasmussen S, Daltveit AK: Group intervention for women with pelvic girdle pain in pregnancy. A randomized controlled trial. *Acta Obstet Gynecol Scand* 2006, 85(11):1320-1326.

215. Haver J, Ansari N, Zainullah P, Kim YM, Tappis H: Misoprostol for Prevention of Postpartum Hemorrhage at Home Birth in Afghanistan: Program Expansion Experience. *J Midwifery Womens Health* 2016, 61(2):196-202.

216. Hawkins JS, Casey BM, Lo JY, Moss K, McIntire DD, Leveno KJ: Weekly compared with daily blood glucose monitoring in women with diet-treated gestational diabetes. *Obstet Gynecol* 2009, 113(6):1307-1312.

217. Heffron R, Mugo N, Hong T, Celum C, Marzinke MA, Ngure K, Asiimwe S, Katabira E, Bukusi EA, Odoyo J *et al*: Pregnancy outcomes and infant growth among babies with in-utero exposure to tenofovir-based preexposure prophylaxis for HIV prevention. *AIDS* 2018, 32(12):1707-1713.

218. Heller HM, Hoogendoorn AW, Honig A, Broekman BFP, van Straten A: The Effectiveness of a Guided Internet-Based Tool for the Treatment of Depression and Anxiety in Pregnancy (MamaKits Online): Randomized Controlled Trial. *J Med Internet Res* 2020, 22(3):e15172.

219. Henrique AJ, Gabrielloni MC, Rodney P, Barbieri M: Non-pharmacological interventions during childbirth for pain relief, anxiety, and neuroendocrine stress parameters: A randomized controlled trial. *Int J Nurs Pract* 2018, 24(3):e12642.

220. Herbec A, Brown J, Tombor I, Michie S, West R: Pilot randomized controlled trial of an internet-based smoking cessation intervention for pregnant smokers ('MumsQuit'). *Drug Alcohol Depend* 2014, 140:130-136.

221. Herring SJ, Cruice JF, Bennett GG, Rose MZ, Davey A, Foster GD: Preventing excessive gestational weight gain among African American women: A randomized clinical trial. *Obesity (Silver Spring)* 2016, 24(1):30-36.

For peer review only

222. Heslehurst N, Hayes L, Jones D, Newham J, Olajide J, McLeman L, McParlin C, de Brun C, Azevedo L: The effectiveness of smoking cessation, alcohol reduction, diet and physical activity interventions in changing behaviours during pregnancy: A systematic review of systematic reviews. *PLoS One* 2020, 15(5):e0232774.
223. Hill B, Skouteris H, Fuller-Tyszkiewicz M: Interventions designed to limit gestational weight gain: a systematic review of theory and meta-analysis of intervention components. *Obes Rev* 2013, 14(6):435-450.
224. Hirshberg A, Downes K, Srinivas S: Comparing standard office-based follow-up with text-based remote monitoring in the management of postpartum hypertension: a randomised clinical trial. *BMJ Qual Saf* 2018, 27(11):871-877.
225. Ho M, Chang YY, Chang WC, Lin HC, Wang MH, Lin WC, Chiu TH: Oral Lactobacillus rhamnosus GR-1 and Lactobacillus reuteri RC-14 to reduce Group B Streptococcus colonization in pregnant women: A randomized controlled trial. *Taiwan J Obstet Gynecol* 2016, 55(4):515-518.
226. Hofmeyr GJ, Abdel-Aleem H, Abdel-Aleem MA: Uterine massage for preventing postpartum haemorrhage. *Cochrane Database Syst Rev* 2013(7):CD006431.
227. Hofmeyr GJ, Lawrie TA, Atallah ÁN, Torloni MR: Calcium supplementation during pregnancy for preventing hypertensive disorders and related problems. In., vol. 2018; 2018.
228. Hofmeyr GJ, Manyame S, Medley N, Williams MJ: Calcium supplementation commencing before or early in pregnancy, for preventing hypertensive disorders of pregnancy. *Cochrane Database Syst Rev* 2019, 9:CD011192.
229. Holden SC, Manor B, Zhou J, Zera C, Davis RB, Yeh GY: Prenatal Yoga for Back Pain, Balance, and Maternal Wellness: A Randomized, Controlled Pilot Study. *Glob Adv Health Med* 2019, 8:2164956119870984.
230. Hollenbach D, Broker R, Herlehy S, Stuber K: Non-pharmacological interventions for sleep quality and insomnia during pregnancy: A systematic review. *J Can Chiropr Assoc* 2013, 57(3):260-270.
231. Homko CJ, Sivan E, Reece EA: The impact of self-monitoring of blood glucose on self-efficacy and pregnancy outcomes in women with diet-controlled gestational diabetes. *The Diabetes educator* 2002, 28(3).
232. Howarth AM, Swain NR: Skills-based childbirth preparation increases childbirth self-efficacy for first time mothers. *Midwifery* 2019, 70:100-105.
233. Howell EA, Balbierz A, Wang J, Parides M, Zlotnick C, Leventhal H: Reducing postpartum depressive symptoms among black and Latina mothers: a randomized controlled trial. *Obstet Gynecol* 2012, 119(5):942-949.
234. Howell EA, Bodnar-Deren S, Balbierz A, Loudon H, Mora PA, Zlotnick C, Wang J, Leventhal H: An intervention to reduce postpartum depressive symptoms: a randomized controlled trial. *Arch Womens Ment Health* 2014, 17(1):57-63.
235. Hsiao WH, Paterno MT, Iradukunda F, Hawkins M: The Preliminary Efficacy of a Sleep Self-management Intervention Using a Personalized Health Monitoring Device during Pregnancy. *Behav Sleep Med* 2021, 19(6):705-716.
236. Hu Y, Lu H, Huang J, Zang Y: Efficacy and safety of non-pharmacological interventions for labour pain management: A systematic review and Bayesian network meta-analysis. In., vol. 30; 2021.
237. Huang CM, Wu HL, Huang SH, Chien LY, Guo JL: Transtheoretical model-based passive smoking prevention programme among pregnant women and mothers of young children. *Eur J Public Health* 2013, 23(5):777-782.
238. Huang TT, Yeh CY, Tsai YC: A diet and physical activity intervention for preventing weight retention among Taiwanese childbearing women: a randomised controlled trial. *Midwifery* 2011, 27(2):257-264.
239. Hughes BL, Gans KM, Raker C, Hipolito ER, Rouse DJ: A brief prenatal intervention of behavioral change to reduce the risk of maternal cytomegalovirus: A randomized controlled trial. In*: 2017*.
240. Huhn EA, Linder T, Eppel D, Weisshaupt K, Klapp C, Schellong K, Henrich W, Yerlikaya-Schatten G, Rosicky I, Husslein P *et al*: Effectiveness of real-time continuous glucose monitoring to improve glycaemic control and pregnancy outcome in patients with gestational diabetes mellitus: a study protocol for a randomised controlled trial. *BMJ Open* 2020, 10(11):e040498.

For peer review only

241. Hui AL, Ludwig S, Gardiner P, Sevenhuysen G, Murray R, Morris M, Shen GX: Community-based Exercise and Dietary Intervention During Pregnancy:A Pilot Study. *Canadian Journal of Diabetes* 2006, 30(2):1-7.
242. Hussein N, Kai J, Qureshi N: The effects of preconception interventions on improving reproductive health and pregnancy outcomes in primary care: A systematic review. In., vol. 22; 2016.
243. Huvinen E, Koivusalo SB, Meinila J, Valkama A, Tiitinen A, Rono K, Stach-Lempinen B, Eriksson JG: Effects of a Lifestyle Intervention During Pregnancy and First Postpartum Year: Findings From the RADIEL Study. *J Clin Endocrinol Metab* 2018, 103(4):1669-1677.
244. Ibrahim HA-F, Said HII, Elgzar WTI: Effect of upright and ambulant positions versus lying down during the active first stage of labor on birth outcomes among nulliparous women: randomized controlled clinical trial. *Frontiers of Nursing* 2020, 7(3):239-248.
245. Ip WY, Tang CS, Goggins WB: An educational intervention to improve women's ability to cope with childbirth. *J Clin Nurs* 2009, 18(15):2125-2135.
246. Isbir GG, Inci F, Onal H, Yildiz PD: The effects of antenatal education on fear of childbirth, maternal self-efficacy and post-traumatic stress disorder (PTSD) symptoms following childbirth: an experimental study. *Appl Nurs Res* 2016, 32:227-232.
247. Islam S, Mohanty SK: Understanding the association between gradient of cooking fuels and low birth weight in India. *SSM Popul Health* 2021, 13:100732.
248. Jackson KT, Dennis CL: Lanolin for the treatment of nipple pain in breastfeeding women: a randomized controlled trial. *Matern Child Nutr* 2017, 13(3).
249. Jahanfar S, Jaafar SH: Effects of restricted caffeine intake by mother on fetal, neonatal and pregnancy outcomes. In., vol. 2015; 2015.
250. Jalambadani Z, Borji A, Bakaeian M: Examining the effect of mindfulness-based art therapy (MBAT) on stress and lifestyle of Iranian pregnant women. *J Obstet Gynaecol* 2020, 40(6):779-783.
251. Jallo N, Thacker LR, 2nd, Menzies V, Stojanovic P, Svikis DS: A Stress Coping App for Hospitalized Pregnant Women at Risk for Preterm Birth. *MCN Am J Matern Child Nurs* 2017, 42(5):257-262.
252. Jarde A, Lewis-Mikhael AM, Moayyedi P, Stearns JC, Collins SM, Beyene J, McDonald SD: Pregnancy outcomes in women taking probiotics or prebiotics: a systematic review and meta-analysis. *BMC Pregnancy Childbirth* 2018, 18(1):14.
253. Jasemzadeh M, Khafaie MA, Jaafarzadeh N, Araban M: Effectiveness of a theory-based mobile phone text message intervention for improving protective behaviors of pregnant women against air pollution: a randomized controlled trial. *Environ Sci Pollut Res Int* 2018, 25(7):6648-6655.
254. Jesse DE, Gaynes BN, Feldhousen EB, Newton ER, Bunch S, Hollon SD: Performance of a Culturally Tailored Cognitive-Behavioral Intervention Integrated in a Public Health Setting to Reduce Risk of Antepartum Depression: A Randomized Controlled Trial. *J Midwifery Womens Health* 2015, 60(5):578-592.
255. Johnsen M, Klingenberg C, Brand M, Revhaug A, Andreassen G: Antenatal breastmilk expression for women with diabetes in pregnancy - a feasibility study. *Int Breastfeed J* 2021, 16(1):56.
256. Jois RS, Tan JK, Silva D: Do probiotics in pregnancy reduce the risk of group B streptococcal colonisation? *J Paediatr Child Health* 2020, 56(9):1468-1472.
257. Jones DL, Rodriguez VJ, Mandell LN, Lee TK, Weiss SM, Peltzer K: Sexual risk behavior among HIV-infected women in South Africa at 12-months postpartum. *AIDS Care* 2019, 31(9):1114-1123.
258. Jones LV, Ray A, Moy FM, Buckley BS: Techniques of monitoring blood glucose during pregnancy for women with pre-existing diabetes. In., vol. 2019; 2019.
259. Jones MH, Barik S, Mangune HH, Jones P, Gregory SJ, Spring JE: Do birth plans adversely affect the outcome of labour? *British Journal of Midwifery* 1998, 6(1):38-41.
260. Jung ME, Stork MJ, Stapleton J, Bourne JE, Martin Ginis KA: A systematic review of behavioural interventions to increase maternal calcium intake. In., vol. 12; 2016.

261. Kabanywanyi AM, Macarthur JR, Stolk WA, Habbema JD, Mshinda H, Bloland PB, Abdulla S, Kachur SP: Malaria in pregnant women in an area with sustained high coverage of insecticide-treated bed nets. *Malar J* 2008, 7:133.

262. Kalafat E, Leslie K, Bhide A, Thilaganathan B, Khalil A: Pregnancy outcomes following home blood pressure monitoring in gestational hypertension. *Pregnancy Hypertens* 2019, 18:14-20.

263. Kalafat E, Mir I, Perry H, Thilaganathan B, Khalil A: Is home blood-pressure monitoring in hypertensive disorders of pregnancy consistent with clinic recordings? *Ultrasound Obstet Gynecol* 2018, 52(4):515-521.

264. Kantrowitz-Gordon I, McCurry SM, Landis CA, Lee R, Wi D: Online prenatal trial in mindfulness sleep management (OPTIMISM): protocol for a pilot randomized controlled trial. *Pilot Feasibility Stud* 2020, 6(1):128.

265. Kardel KR, Henriksen T, Iversen PO: No effect of energy supply during childbirth on delivery outcomes in nulliparous women: a randomised, double-blind, placebo-controlled trial. *J Obstet Gynaecol* 2010, 30(3):248-252.

266. Karimi F, Babazadeh R, Roudsari RL, Asgharipour N, Esmaily H: Comparing the Effectiveness of Sexual Counseling Based on PLISSITand BETTER Models on Sexual Selfdisclosure in Women with Sexual Problems after Childbirth: A Randomized Trial. *Iran J Nurs Midwifery Res* 2021, 26(1):68-74.

267. Katz J, Tielsch JM, Khatry SK, Shrestha L, Breysse P, Zeger SL, Kozuki N, Checkley W, LeClerq SC, Mullany LC: Impact of Improved Biomass and Liquid Petroleum Gas Stoves on Birth Outcomes in Rural Nepal: Results of 2 Randomized Trials. *Glob Health Sci Pract* 2020, 8(3):372-382.

268. Kavanagh J, Kelly AJ, Thomas J: Sexual intercourse for cervical ripening and induction of labour. *Cochrane Database Syst Rev* 2001(2):CD003093.

269. Kavanagh J, Kelly AJ, Thomas J: Breast stimulation for cervical ripening and induction of labour. In., vol. 2010; 2005.

270. Kazemi A, Ehsanpour S, Zahraei NSN, Hasanzadeh A, Beigi NMA, Malverdi Z: Impact of health belief modification on intention to make smoke free home among pregnant women. *Journal of Research in Medical Sciences* 2011, 16(6).

271. Keats EC, Akseer N, Thurairajah P, Cousens S, Bhutta ZA, Global Young Women's Nutrition Investigators G: Multiple-micronutrient supplementation in pregnant adolescents in low- and middle-income countries: a systematic review and a meta-analysis of individual participant data. *Nutr Rev* 2022, 80(2):141-156.

272. Keller C, Records K, Ainsworth B, Permana P, Coonrod DV: Interventions for weight management in postpartum women. In., vol. 37; 2008.

273. Kelly AJ, Kavanagh J, Thomas J: Castor oil, bath and/or enema for cervical priming and induction of labour. In., vol. 2013; 2013.

274. Kennelly MA, Ainscough K, Lindsay KL, O'Sullivan E, Gibney ER, McCarthy M, Segurado R, DeVito G, Maguire O, Smith T *et al*: Pregnancy exercise and nutrition with smartphone application support a randomized controlled trial. In*: 2018*.

275. Khatun F, Lee TW, Lee HJ, Park J, Song JE, Kim S: Does a nurse-led postpartum self-care program for first-time mothers in Bangladesh improve postpartum fatigue, depressive mood, and maternal functioning?: a non-synchronized quasi-experimental study. *Korean Journal of Women Health Nursing* 2021, 27(3):196-208.

276. Kim HK, Niederdeppe J, Graham M, Olson C, Gay G: Effects of Online Self-Regulation Activities on Physical Activity Among Pregnant and Early Postpartum Women. *J Health Commun* 2015, 20(10):1115-1124.

277. Kimber L, McNabb M, Mc Court C, Haines A, Brocklehurst P: Massage or music for pain relief in labour: a pilot randomised placebo controlled trial. *Eur J Pain* 2008, 12(8):961-969.

278. King SE, Yeh PT, Rhee DK, Tuncalp O, Rogers LM, Narasimhan M: Self-management of iron and folic acid supplementation during pre-pregnancy, pregnancy and postnatal periods: a systematic review. *BMJ Glob Health* 2021, 6(5).

For peer review only

279. Kinser P, Jallo N, Huberty J, Jones E, Thacker L, Moyer S, Laird B, Rider A, Lanni S, Drozd F *et al*: Study protocol for a multisite randomized controlled trial of an internet and mobile-based intervention for preventing and reducing perinatal depressive symptoms. *Res Nurs Health* 2021, 44(1):13-23.

280. Kinser PA, Thacker LR, Rider A, Moyer S, Amstadter AB, Mazzeo SE, Bodnar-Deren S, Starkweather A: Feasibility, Acceptability, and Preliminary Effects of "Mindful Moms": A Mindful Physical Activity Intervention for Pregnant Women with Depression. *Nurs Res* 2021, 70(2):95-105.

281. Kirca AS, Kanza Gul D: Effects of self-acupressure on pregnancy-related constipation: A single-blind randomized controlled study. *Explore (NY)* 2021, 17(5):463-468.

282. Kirihara N, Kamitomo M, Tabira T, Hashimoto T, Taniguchi H, Maeda T: Effect of probiotics on perinatal outcome in patients at high risk of preterm birth. *J Obstet Gynaecol Res* 2018, 44(2):241-247.

283. Kitt JA, Fox RL, Cairns AE, Mollison J, Burchert HH, Kenworthy Y, McCourt A, Suriano K, Lewandowski AJ, Mackillop L *et al*: Short-Term Postpartum Blood Pressure Self-Management and Long-Term Blood Pressure Control: A Randomized Controlled Trial. *Hypertension* 2021, 78(2):469-479.

284. Kobayashi M, Ogawa K, Morisaki N, Tani Y, Horikawa R, Fujiwara T: Dietary n-3 Polyunsaturated Fatty Acids in Late Pregnancy and Postpartum Depressive Symptom among Japanese Women. *Front Psychiatry* 2017, 8(NOV):241.

285. Koivusalo SB, Rono K, Klemetti MM, Roine RP, Lindstrom J, Erkkola M, Kaaja RJ, Poyhonen-Alho M, Tiitinen A, Huvinen E *et al*: Gestational Diabetes Mellitus Can Be Prevented by Lifestyle Intervention: The Finnish Gestational Diabetes Prevention Study (RADIEL): A Randomized Controlled Trial. *Diabetes Care* 2016, 39(1):24-30.

286. Kolivand M, Rahimi MA, Keramat A, Shariati M, Emamian MH: Effect of a new self-care guide package on maternal and neonatal outcomes in gestational diabetes: A randomized control trial. *J Diabetes* 2019, 11(2):139-147.

287. Kolivand M, Rahimi MA, Shariati M, Keramat A, Emamian MH: The Effect of Self-care Educational/Training Interventions on the Outcomes of Gestational Diabetes: A Review Article. *Iran J Public Health* 2018, 47(12):1805-1815.

288. Korn L, Koren G, Yaakov A, Madar G, Blau A: Evaluating the Effectiveness of Childbirth Preparation Courses on Women's Self-Efficacy among Ultra-Orthodox Jewish Religious Women in Israel. *Healthcare (Basel)* 2021, 9(7).

289. Korte JE, Kisa R, Vrana-Diaz CJ, Malek AM, Buregyeya E, Matovu JKB, Kagaayi J, Musoke W, Chemusto H, Mukama SC *et al*: HIV Oral Self-Testing for Male Partners of Women Attending Antenatal Care in Central Uganda: Uptake of Testing and Linkage to Care in a Randomized Trial. *J Acquir Immune Defic Syndr* 2020, 84(3):271-279.

290. Koshida S, Tokoro S, Katsura D, Tsuji S, Murakami T, Takahashi K: Fetal movement counting is associated with the reduction of delayed maternal reaction after perceiving decreased fetal movements: a prospective study. *Sci Rep* 2021, 11(1):10818.

291. Koyuncu SB, Bulbul M: The impact of yoga on fear of childbirth and childbirth self-efficacy among third trimester pregnants. *Complement Ther Clin Pract* 2021, 44:101438.

292. Krauss-Silva L, Moreira ME, Alves MB, Braga A, Camacho KG, Batista MR, Almada-Horta A, Rebello MR, Guerra F: A randomised controlled trial of probiotics for the prevention of spontaneous preterm delivery associated with bacterial vaginosis: preliminary results. *Trials* 2011, 12:239.

293. Kumar R, Farzeen M, Hafeez A, Achakzai BK, Vankwani M, Lal M, Iqbal R, Somrongthong R: Effectiveness of a health education intervention on the use of long-lasting insecticidal nets for the prevention of malaria in pregnant women of Pakistan: a quasi-experimental study. *Malar J* 2020, 19(1):232.

294. Kuppermann M, Kaimal AJ, Blat C, Gonzalez J, Thiet MP, Bermingham Y, Altshuler AL, Bryant AS, Bacchetti P, Grobman WA: Effect of a Patient-Centered Decision Support Tool on Rates of Trial of Labor After Previous Cesarean Delivery: The PROCEED Randomized Clinical Trial. *JAMA* 2020, 323(21):2151-2159.

For peer review only

295. Kwena Z, Kimbo L, Darbes LA, Hatcher AM, Helova A, Owino G, Thirumurthy H, Bukusi EA, Braun T, Kilgore M *et al*: Testing strategies for couple engagement in prevention of mother-to-child transmission of HIV and family health in Kenya: study protocol for a randomized controlled trial. *Trials* 2021, 22(1):19.
296. Labrecque M, Eason E, Marcoux S: Randomized trial of perineal massage during pregnancy: perineal symptoms three months after delivery. *Am J Obstet Gynecol* 2000, 182(1 Pt 1):76-80.
297. Labrecque M, Nouwen A, Bergeron M, Rancourt JF: A randomized controlled trial of nonpharmacologic approaches for relief of low back pain during labor. *J Fam Pract* 1999, 48(4):259-263.
298. Landon MB, Spong CY, Thom E, Carpenter MW, Ramin SM, Casey B, Wapner RJ, Varner MW, Rouse DJ, Thorp JM, Jr. *et al*: A multicenter, randomized trial of treatment for mild gestational diabetes. *N Engl J Med* 2009, 361(14):1339-1348.
299. Lanou H, Huybregts L, Roberfroid D, Nikiema L, Kouanda S, Van Camp J, Kolsteren P: Prenatal nutrient supplementation and postnatal growth in a developing nation: an RCT. *Pediatrics* 2014, 133(4):e1001-1008.
300. Lanssens D, Thijs IM, Gyselaers W, consortium PI: Design of the Pregnancy REmote MOnitoring II study (PREMOM II): a multicenter, randomized controlled trial of remote monitoring for gestational hypertensive disorders. *BMC Pregnancy Childbirth* 2020, 20(1):626.
301. Lauzon L, Hodnett ED, Kellie FJ: Antenatal education for self-diagnosis of the onset of active labour at term. In., vol. 2017; 1998.
302. Lawrence T, Aveyard P, Cheng KK, Griffin C, Johnson C, Croghan E: Does stage-based smoking cessation advice in pregnancy result in long-term quitters? 18-month postpartum follow-up of a randomized controlled trial. *Addiction* 2005, 100(1):107-116.
303. Lee JT, Tsai JL: Transtheoretical model-based postpartum sexual health education program improves women's sexual behaviors and sexual health. *J Sex Med* 2012, 9(4):986-996.
304. Lee M, Miller SM, Wen KY, Hui SK, Roussi P, Hernandez E: Cognitive-behavioral intervention to promote smoking cessation for pregnant and postpartum inner city women. *J Behav Med* 2015, 38(6):932-943.
305. Lertbunnaphong T, Lapthanapat N, Leetheeragul J, Hakularb P, Ownon A: Postpartum blood loss: visual estimation versus objective quantification with a novel birthing drape. *Singapore Med J* 2016, 57(6):325-328.
306. Leung SS, Lam TH: Group antenatal intervention to reduce perinatal stress and depressive symptoms related to intergenerational conflicts: a randomized controlled trial. *Int J Nurs Stud* 2012, 49(11):1391-1402.
307. Levitt C, Shaw E, Wong S, Kaczorowski J: Systematic review of the literature on postpartum care: Effectiveness of interventions for smoking relapse prevention, cessation, and reduction in postpartum women. In., vol. 34; 2007.
308. Lim K, Chan SY, Lim SL, Tai BC, Tsai C, Wong SR, Ang SM, Yew TW, Tai ES, Yong EL: A Smartphone App to Restore Optimal Weight (SPAROW) in Women With Recent Gestational Diabetes Mellitus: Randomized Controlled Trial. *JMIR Mhealth Uhealth* 2021, 9(3):e22147.
309. Lim S, Hill B, Pirotta S, O'Reilly S, Moran L: What Are the Most Effective Behavioural Strategies in Changing Postpartum Women's Physical Activity and Healthy Eating Behaviours? A Systematic Review and Meta-Analysis. *J Clin Med* 2020, 9(1).
310. Lim S, O'Reilly S, Behrens H, Skinner T, Ellis I, Dunbar JA: Effective strategies for weight loss in post-partum women: A systematic review and meta-analysis. In., vol. 16; 2015.
311. Lin YC, Gau ML, Kao GH, Lee HC: Efficacy of an Ergonomic Ankle Support Aid for Squatting Position in Improving Pushing Skills and Birth Outcomes During the Second Stage of Labor: A Randomized Controlled Trial. *J Nurs Res* 2018, 26(6):376-384.
312. Linden K, Berg M, Adolfsson A, Sparud-Lundin C: Person-centred, web-based support in pregnancy and early motherhood for women with Type 1 diabetes mellitus: a randomized controlled trial. *Diabet Med* 2018, 35(2):232-241.
313. Liston RM, Bloom K, Zimmer P: The psychological effects of counting fetal movements. *Birth* 1994, 21(3):135-140.

314. Liu J, Wilcox S, Wingard E, Turner-McGrievy G, Hutto B, Burgis J: A Behavioral Lifestyle Intervention to Limit Gestational Weight Gain in Pregnant Women with Overweight and Obesity. *Obesity (Silver Spring)* 2021, 29(4):672-680.

315. Liu N, Wang J, Chen Dd, Sun Wj, Li P, Zhang W: Effects of exercise on pregnancy and postpartum fatigue: A systematic review and meta-analysis. In., vol. 253; 2020.

316. Liu P, Wen W, Yu KF, Gao X, Lo ECM, Wong MCM: Effectiveness of a family-centered behavioral and educational counselling approach to improve periodontal health of pregnant women: a randomized controlled trial. *BMC Oral Health* 2020, 20(1):284.

317. Liu YH, Lee CS, Yu CH, Chen CH: Effects of music listening on stress, anxiety, and sleep quality for sleep-disturbed pregnant women. *Women Health* 2016, 56(3):296-311.

318. Llorente AM, Jensen CL, Voigt RG, Fraley JK, Berretta MC, Heird WC: Effect of maternal docosahexaenoic acid supplementation on postpartum depression and information processing. *Am J Obstet Gynecol* 2003, 188(5):1348-1353.

319. Logan S, Browne J, McKenzie H, Templeton A, Bhattacharya S: Evaluation of endocervical, first-void urine and self-administered vulval swabs for the detection of Chlamydia trachomatis in a miscarriage population. *BJOG* 2005, 112(1):103-106.

320. Loughnan SA, Sie A, Hobbs MJ, Joubert AE, Smith J, Haskelberg H, Mahoney AEJ, Kladnitski N, Holt CJ, Milgrom J *et al*: A randomized controlled trial of 'MUMentum Pregnancy': Internet-delivered cognitive behavioral therapy program for antenatal anxiety and depression. *J Affect Disord* 2019, 243:381-390.

321. Loukopoulou AN, Vardavas CI, Farmakides G, Rosolymos C, Chrelias C, Tzatzarakis M, Tsatsakis A, Myridakis A, Lyberi M, Behrakis PK: Counselling for smoking cessation during pregnancy reduces tobacco-specific nitrosamine (NNAL) concentrations: A randomized controlled trial. *Eur J Midwifery* 2018, 2(November):14.

322. Lowndes TA, Egan SJ, McEvoy PM: Efficacy of brief guided self-help cognitive behavioral treatment for perfectionism in reducing perinatal depression and anxiety: a randomized controlled trial. *Cogn Behav Ther* 2019, 48(2):106-120.

323. Lucas R, Zhang Y, Walsh SJ, Evans H, Young E, Starkweather A: Efficacy of a Breastfeeding Pain Self-Management Intervention: A Pilot Randomized Controlled Trial. *Nurs Res* 2019, 68(2):E1-E10.

324. Lucena L, Frange C, Pinto ACA, Andersen ML, Tufik S, Hachul H: Mindfulness interventions during pregnancy: A narrative review. In., vol. 18; 2020.

325. Maddady SM, Charandabi SM-A, Shafaei FS, Mirghafourvand M: Comparing the Effects of Hot Shower and Intravenous Injection of Hyoscine on the Pain Intensity and Duration of Active Phase of Labour in Nulliparous Women. *Journal of Clinical and Diagnostic Research* 2018, 12(8).

326. Madden K, Middleton P, Cyna AM, Matthewson M, Jones L: Hypnosis for pain management during labour and childbirth. In., vol. 2016; 2016.

327. Magwood O, Kpade V, Thavorn K, Oliver S, Mayhew AD, Pottie K: Effectiveness of home-based records on maternal, newborn and child health outcomes: A systematic review and meta-analysis. *PLoS One* 2019, 14(1):e0209278.

328. Mahnaz E, Nasim B, Sonia O: Effect of a structured educational package on women's sexual function during pregnancy. *Int J Gynaecol Obstet* 2020, 148(2):225-230.

329. Malekuti J, Mirghafourvand M, Samadi K, Abbasalizadeh F, Khodaei L: Comparison of the effect of Myrtus communis herbal and anti-hemorrhoid ointments on the hemorrhoid symptoms and quality of life in postpartum women with grade I and II internal hemorrhoid: A triple-blinded randomized controlled clinical trial. *J Complement Integr Med* 2019, 16(4).

330. Maman S, Moodley D, McNaughton-Reyes HL, Groves AK, Kagee A, Moodley P: Efficacy of enhanced HIV counseling for risk reduction during pregnancy and in the postpartum period: a randomized controlled trial. *PLoS One* 2014, 9(5):e97092.

For peer review only

331. Mangesi L, Hofmeyr GJ, Smith V, Smyth RMD: Fetal movement counting for assessment of fetal wellbeing. In., vol. 2015; 2015.
332. Mao HJ, Li HJ, Chiu H, Chan WC, Chen SL: Effectiveness of antenatal emotional self-management training program in prevention of postnatal depression in Chinese women. *Perspect Psychiatr Care* 2012, 48(4):218-224.
333. Marc I, Toureche N, Ernst E, Hodnett ED, Blanchet C, Dodin S, Njoya MM: Mind-body interventions during pregnancy for preventing or treating women's anxiety. In., vol. 2011; 2011.
334. Marchant T, Schellenberg JA, Edgar T, Nathan R, Abdulla S, Mukasa O, Mponda H, Lengeler C: Socially marketed insecticide-treated nets improve malaria and anaemia in pregnancy in southern Tanzania. *Trop Med Int Health* 2002, 7(2):149-158.
335. Martin J, MacDonald-Wicks L, Hure A, Smith R, Collins CE: Reducing postpartum weight retention and improving breastfeeding outcomes in overweight women: a pilot randomised controlled trial. *Nutrients* 2015, 7(3):1464-1479.
336. Martis R, Crowther CA, Shepherd E, Alsweiler J, Downie MR, Brown J: Treatments for women with gestational diabetes mellitus: An overview of Cochrane systematic reviews. In., vol. 2018; 2018.
337. Marufu TC, Ahankari A, Coleman T, Lewis S: Maternal smoking and the risk of still birth: systematic review and meta-analysis. *BMC Public Health* 2015, 15(1):239.
338. Mary M, Jafarey S, Dabash R, Kamal I, Rabbani A, Abbas D, Durocher J, Tan YL, Winikoff B: The Safety and Feasibility of a Family First Aid Approach for the Management of Postpartum Hemorrhage in Home Births: A Pre-post Intervention Study in Rural Pakistan. *Matern Child Health J* 2021, 25(1):118-126.
339. Masoumi SZ, Kazemi F, Oshvandi K, Jalali M, Esmaeili-Vardanjani A, Rafiei H: Effect of Training Preparation for Childbirth on Fear of Normal Vaginal Delivery and Choosing the Type of Delivery Among Pregnant Women in Hamadan, Iran: A Randomized Controlled Trial. *Journal of family & reproductive health* 2016, 10(3).
340. Masters SH, Agot K, Obonyo B, Napierala Mavedzenge S, Maman S, Thirumurthy H: Promoting Partner Testing and Couples Testing through Secondary Distribution of HIV Self-Tests: A Randomized Clinical Trial. *PLoS Med* 2016, 13(11):e1002166.
341. Masulli M, Vitacolonna E, Fraticelli F, Della Pepa G, Mannucci E, Monami M: Effects of probiotic supplementation during pregnancy on metabolic outcomes: A systematic review and meta-analysis of randomized controlled trials. In., vol. 162; 2020.
342. Masuzawa Y, Kataoka Y, Nakamura S, Yaju Y: Cooling the lower abdomen to reduce postpartum blood loss: A randomized controlled trial. *PLoS One* 2017, 12(10):e0186365.
343. Matei A, Saccone G, Vogel JP, Armson AB: Primary and secondary prevention of preterm birth: a review of systematic reviews and ongoing randomized controlled trials. In., vol. 236; 2019.
344. Matenchuk B, Khurana R, Cai C, Boule NG, Slater L, Davenport MH: Prenatal bed rest in developed and developing regions: a systematic review and meta-analysis. *CMAJ Open* 2019, 7(3):E435-E445.
345. Matthews LT, Jaggernath M, Kriel Y, Smith PM, O'Neil K, Haberer JE, Hendrix C, Baeten JM, Ware NC, Wirth K *et al*: Protocol for a longitudinal study to evaluate the use of tenofovir-based PrEP for safer conception and pregnancy among women in South Africa. *BMJ Open* 2019, 9(7):e027227.
346. Matvienko-Sikar K, Lee L, Murphy G, Murphy L: The effects of mindfulness interventions on prenatal well-being: A systematic review. *Psychol Health* 2016, 31(12):1415-1434.
347. McBride CM, Baucom DH, Peterson BL, Pollak KI, Palmer C, Westman E, Lyna P: Prenatal and postpartum smoking abstinence a partner-assisted approach. *Am J Prev Med* 2004, 27(3):232-238.
348. McBride CM, Curry SJ, Lando HA, Pirie PL, Grothaus LC, Nelson JC: Prevention of relapse in women who quit smoking during pregnancy. *Am J Public Health* 1999, 89(5):706-711.
349. McCarthy EA, Walker SP, Ugoni A, Lappas M, Leong O, Shub A: Self-weighing and simple dietary advice for overweight and obese pregnant women to reduce obstetric complications without impact on quality of life: A randomised controlled trial. In., vol. 123; 2016.

For peer review only

350. McCloskey RJ, Reno R: Complementary health approaches for postpartum depression: A systematic review. In., vol. 17; 2019.
351. McGrath A, Sharpe L, Lah S, Parratt K: Evaluation of a Decision Aid for Women with Epilepsy Who Are Considering Pregnancy: A Randomized Controlled Trial. *Med Decis Making* 2017, 37(5):589-599.
352. McLeod D, Pullon S, Benn C, Cookson T, Dowell A, Viccars A, White S, Green R, Crooke M: Can support and education for smoking cessation and reduction be provided effectively by midwives within primary maternity care? *Midwifery* 2004, 20(1):37-50.
353. Megregian M, Emeis C, Nieuwenhuijze M: The Impact of Shared Decision-Making in Perinatal Care: A Scoping Review. *J Midwifery Womens Health* 2020, 65(6):777-788.
354. Middleton P, Gomersall JC, Gould JF, Shepherd E, Olsen SF, Makrides M: Omega-3 fatty acid addition during pregnancy. In., vol. 2018; 2018.
355. Midhet F, Becker S: Impact of community-based interventions on maternal and neonatal health indicators: Results from a community randomized trial in rural Balochistan, Pakistan. *Reprod Health* 2010, 7(1):30.
356. Milgrom J, Holt C, Holt CJ, Ross J, Ericksen J, Gemmill AW: Feasibility study and pilot randomised trial of an antenatal depression treatment with infant follow-up. *Arch Womens Ment Health* 2015, 18(5):717-730.
357. Ming WK, Ding W, Zhang CJP, Zhong L, Long Y, Li Z, Sun C, Wu Y, Chen H, Chen H *et al*: The effect of exercise during pregnancy on gestational diabetes mellitus in normal-weight women: a systematic review and meta-analysis. *BMC Pregnancy Childbirth* 2018, 18(1):440.
358. Minschart C, Maes T, De Block C, Van Pottelbergh I, Myngheer N, Abrams P, Vinck W, Leuridan L, Mathieu C, Billen J *et al*: Mobile-Based Lifestyle Intervention in Women with Glucose Intolerance after Gestational Diabetes Mellitus (MELINDA), A Multicenter Randomized Controlled Trial: Methodology and Design. *J Clin Med* 2020, 9(8).
359. Miquelutti MA, Cecatti JG, Makuch MY: Upright position during the first stage of labor: a randomised controlled trial. *Acta Obstet Gynecol Scand* 2007, 86(5):553-558.
360. Miquelutti MA, Cecatti JG, Makuch MY: Evaluation of a birth preparation program on lumbopelvic pain, urinary incontinence, anxiety and exercise: a randomized controlled trial. *BMC Pregnancy Childbirth* 2013, 13:154.
361. Miquelutti MA, Silveira C, Cecatti JG: Kinesiologic tape for labor pain control: Randomized controlled trial. *Physiother Theory Pract* 2019, 35(7):614-621.
362. Mir AM, Wajid A, Gull S: Helping rural women in Pakistan to prevent postpartum hemorrhage: a quasi experimental study. *BMC Pregnancy Childbirth* 2012, 12:120.
363. Mobarakabadi SS, Shahbazzadegan S, Ozgoli G: The effect of P6 acupressure on nausea and vomiting of pregnancy: A randomized, single-blind, placebo-controlled trial. *Advances in Integrative Medicine* 2020, 7(2):67-72.
364. Mofenson LM, Baggaley RC, Mameletzis I: Tenofovir disoproxil fumarate safety for women and their infants during pregnancy and breastfeeding. *AIDS* 2017, 31(2):213-232.
365. Mohammadi F, Malakooti J, Babapoor J, Mohammad-Alizadeh-Charandabi S: The effect of a home-based exercise intervention on postnatal depression and fatigue: A randomized controlled trial. *Int J Nurs Pract* 2015, 21(5):478-485.
366. Monim Alzurfi N, Shadhan Al-Ogaili S: Effectiveness of Breathing as a Non-Pharmacological Method to Reduce Pain Severity among Woman During Labor. *Medico Legal Update* 2021, 21(1):674-677.
367. Moore L, Campbell R, Whelan A, Mills N, Lupton P, Misselbrook E, Frohlich J: Self help smoking cessation in pregnancy: cluster randomised controlled trial. *BMJ* 2002, 325(7377):1383.
368. Moradi M, Niazi A, Heydarian Miri H, Lopez V: The effect of evening primrose oil on labor induction and cervical ripening: A systematic review and meta-analysis. In., vol. 35; 2021.
369. Morison PN, Bacardi-Gascon M, Lopez-Corrales M, Jimenez-Cruz A: Combined dietary-exercise intervention for gestational weight gain and birthweight: a meta-analysis. *Asia Pac J Clin Nutr* 2018, 27(4):860-868.

For peer review only

370. Motahari-Tabari NS, Nasiri-Amiri F, Faramarzi M, Shirvani MA, Bakhtiari A, Omidvar S: The Effectiveness of Information-Motivation-Behavioral Skills Model on Self-Care Practices in Early Pregnancy to Prevent Gestational Diabetes Mellitus in Iranian Overweight and Obese Women: A Randomized Controlled Trial. *Int Q Community Health Educ* 2021:272684X211020300.

371. Motlagh AE, Babazadeh R, Akhlaghi F, Esmaily H: Effect of an educational intervention program based on bandura's self-efficacy theory on self-care, self-efficacy, and blood sugar levels in mothers with pre-diabetes during pregnancy. In., vol. 9; 2019.

372. Munkhondya BMJ, Munkhondya TE, Chirwa E, Wang H: Efficacy of companion-integrated childbirth preparation for childbirth fear, self-efficacy, and maternal support in primigravid women in Malawi. *BMC Pregnancy Childbirth* 2020, 20(1):48.

373. Munro S, Stacey D, Lewis KB, Bansback N: Choosing treatment and screening options congruent with values: Do decision aids help? Sub-analysis of a systematic review. In., vol. 99; 2016.

374. Murphy HR, Rayman G, Lewis K, Kelly S, Johal B, Duffield K, Fowler D, Campbell PJ, Temple RC: Effectiveness of continuous glucose monitoring in pregnant women with diabetes: randomised clinical trial. *BMJ* 2008, 337(7675):a1680.

375. N.Y VDW, C H, W VD: Preventing prenatal alcohol use via health counseling by midwives and Internet-based computer tailored feedback: A randomized controlled trial. In., vol. 19; 2012.

376. Napolitano MA, Harrington CB, Patchen L, Ellis LP, Ma T, Chang K, Gaminian A, Bailey CP, Evans WD: Feasibility of a Digital Intervention to Promote Healthy Weight Management among Postpartum African American/Black Women. *Int J Environ Res Public Health* 2021, 18(4).

377. Nassar N, Roberts CL, Raynes-Greenow CH, Barratt A, Peat B, Decision Aid for Breech Presentation Trial C: Evaluation of a decision aid for women with breech presentation at term: a randomised controlled trial [ISRCTN14570598]. *BJOG* 2007, 114(3):325-333.

378. Naughton F, Cooper S, Foster K, Emery J, Leonardi-Bee J, Sutton S, Jones M, Ussher M, Whitemore R, Leighton M *et al*: Large multi-centre pilot randomized controlled trial testing a low-cost, tailored, self-help smoking cessation text message intervention for pregnant smokers (MiQuit). *Addiction* 2017, 112(7):1238-1249.

379. Naughton F, Prevost AT, Gilbert H, Sutton S: Randomized controlled trial evaluation of a tailored leaflet and SMS text message self-help intervention for pregnant smokers (MiQuit). *Nicotine Tob Res* 2012, 14(5):569-577.

380. Naughton F, Prevost AT, Sutton S: Self-help smoking cessation interventions in pregnancy: A systematic review and meta-analysis. In., vol. 103; 2008.

381. Nazari M, Basiri F, Kaveh MH, Ghaem H: Investigating the Effect of Fordyce Happiness Cognitive-Behavioral Training on Perceived Stress and Happiness in Pregnant Females: Applying the Self-Efficacy Theory. *Iranian Red Crescent Medical Journal* 2018, In Press(In Press).

382. Ndyomugyenyi R, Clarke SE, Hutchison CL, Hansen KS, Magnussen P: Efficacy of malaria prevention during pregnancy in an area of low and unstable transmission: an individually-randomised placebo-controlled trial using intermittent preventive treatment and insecticide-treated nets in the Kabale Highlands, southwestern Uganda. *Trans R Soc Trop Med Hyg* 2011, 105(11):607-616.

383. Nelson DB, Bellamy S, Gray TS, Nachamkin I: Self-collected versus provider-collected vaginal swabs for the diagnosis of bacterial vaginosis: an assessment of validity and reliability. *J Clin Epidemiol* 2003, 56(9):862-866.

384. Neri I, Dante G, Pignatti L, Salvioli C, Facchinetti F: Castor oil for induction of labour: a retrospective study. *J Matern Fetal Neonatal Med* 2018, 31(16):2105-2108.

385. Ngichabe SK, Gatinu BW, Nyangore MA, Karuga R, Wanyonyi SZ, Kiarie JN: Reminder Systems for Self Uterine Massage in the Prevention of Postpartum Blood Loss. *East Afr Med J* 2012, 89(4):128-133.

386. Nicholson WK, Beckham AJ, Hatley K, Diamond M, Johnson LS, Green SL, Tate D: The Gestational Diabetes Management System (GooDMomS): development, feasibility and lessons learned from a patient-informed, web-based pregnancy and postpartum lifestyle intervention. *BMC Pregnancy Childbirth* 2016, 16(1):277.

For peer review only

387. Nielsen JN, Gittelsohn J, Anliker J, O'Brien K: Interventions to improve diet and weight gain among pregnant adolescents and recommendations for future research. *J Am Diet Assoc* 2006, 106(11):1825-1840.

388. Nieminen K, Andersson G, Wijma B, Ryding EL, Wijma K: Treatment of nulliparous women with severe fear of childbirth via the Internet: a feasibility study. *J Psychosom Obstet Gynaecol* 2016, 37(2):37-43.

389. Nilsson C, Lundgren I, Smith V, Vehvilainen-Julkunen K, Nicoletti J, Devane D, Bernloehr A, van Limbeek E, Lalor J, Begley C: Women-centred interventions to increase vaginal birth after caesarean section (VBAC): A systematic review. *Midwifery* 2015, 31(7):657-663.

390. Njagi JK, Magnussen P, Estambale B, Ouma J, Mugo B: Prevention of anaemia in pregnancy using insecticide-treated bednets and sulfadoxine-pyrimethamine in a highly malarious area of Kenya: a randomized controlled trial. *Transactions of the Royal Society of Tropical Medicine and Hygiene* 2003, 97(3):277-282.

391. Nwebube C, Glover V, Stewart L: Prenatal listening to songs composed for pregnancy and symptoms of anxiety and depression: a pilot study. *BMC Complement Altern Med* 2017, 17(1):256.

392. O'Kelly SM, Moore ZE: Antenatal maternal education for improving postnatal perineal healing for women who have birthed in a hospital setting. In., vol. 2017; 2017.

393. Oakeshott P, Hay P, Hay S, Steinke F, Rink E, Thomas B, Oakeley P, Kerry S: Detection of Chlamydia trachomatis infection in early pregnancy using self-administered vaginal swabs and first pass urines: A cross-sectional community-based survey. *British Journal of General Practice* 2002, 52(483).

394. Offianan AT, Penali LK, Coulibaly M, Tiacoh N, Ako A, Adji E, Coulibaly B, Koffi D, Sarr D, Jambou R *et al*: Comparative efficacy of uncontrolled and controlled intermittent preventive treatment during pregnancy (IPTp) with combined use of LLTNs in high resistance area to sulfadoxine-pyrimethamine in Cote d'Ivoire. *Infect Drug Resist* 2012, 5(1):53-63.

395. Oken E, Ning Y, Rifas-Shiman SL, Radesky JS, Rich-Edwards JW, Gillman MW: Associations of physical activity and inactivity before and during pregnancy with glucose tolerance. *Obstet Gynecol* 2006, 108(5):1200-1207.

396. Okesene-Gafa KAM, Li M, McKinlay CJD, Taylor RS, Rush EC, Wall CR, Wilson J, Murphy R, Taylor R, Thompson JMD *et al*: Effect of antenatal dietary interventions in maternal obesity on pregnancy weight-gain and birthweight: Healthy Mums and Babies (HUMBA) randomized trial. *Am J Obstet Gynecol* 2019, 221(2):152 e151-152 e113.

397. Oladapo OT, Blum J, Abalos E, Okusanya BO: Advance misoprostol distribution to pregnant women for preventing and treating postpartum haemorrhage. In., vol. 2020; 2020.

398. Oliveira FS, Vieira F, Guimaraes JV, Aredes ND, Campbell SH: Lanolin and prenatal health education for prevention of nipple pain and trauma: Randomized clinical trial. *Enferm Clin (Engl Ed)* 2021, 31(2):82-90.

399. Olson CM, Groth SW, Graham ML, Reschke JE, Strawderman MS, Fernandez ID: The effectiveness of an online intervention in preventing excessive gestational weight gain: the e-moms roc randomized controlled trial. *BMC Pregnancy Childbirth* 2018, 18(1):148.

400. Olson CM, Strawderman MS, Graham ML: Use of an Online Diet Goal-Setting Tool: Relationships With Gestational Weight Gain. *J Nutr Educ Behav* 2019, 51(4):391-399.

401. Ong MJ, Guelfi KJ, Hunter T, Wallman KE, Fournier PA, Newnham JP: Supervised home-based exercise may attenuate the decline of glucose tolerance in obese pregnant women. *Diabetes Metab* 2009, 35(5):418-421.

402. Ononge S, Campbell OM, Kaharuza F, Lewis JJ, Fielding K, Mirembe F: Effectiveness and safety of misoprostol distributed to antenatal women to prevent postpartum haemorrhage after child-births: a stepped-wedge cluster-randomized trial. *BMC Pregnancy Childbirth* 2015, 15:315.

403. Oostdam N, Van Poppel MNM, Wouters MGAJ, Van Mechelen W: Interventions for preventing gestational diabetes mellitus: A systematic review and meta-analysis. In., vol. 20; 2011.

404. Osaki K, Hattori T, Kosen S: The role of home-based records in the establishment of a continuum of care for mothers, newborns, and children in Indonesia. *Glob Health Action* 2013, 6(1):1-12.

For peer review only

405. Osório S, Júnior L, Nicolau A: Assessment of the effectiveness of non-pharmacological methods in pain relief during labor. *Revista da Rede de Enfermagem do Nordeste* 2014, 15:174-184.
406. Osterman RL, Carle AC, Ammerman RT, Gates D: Single-session motivational intervention to decrease alcohol use during pregnancy. *J Subst Abuse Treat* 2014, 47(1):10-19.
407. Owais S, Chow CHT, Furtado M, Frey BN, Van Lieshout RJ: Non-pharmacological interventions for improving postpartum maternal sleep: A systematic review and meta-analysis. In., vol. 41; 2018.
408. Palacios C, Kostiuk LK, Pena-Rosas JP: Vitamin D supplementation for women during pregnancy. *Cochrane Database Syst Rev* 2019, 7:CD008873.
409. Palnati M, Marcus BH, Pekow P, Rosal MC, Manson JE, Chasan-Taber L: The Impact of a Lifestyle Intervention on Postpartum Weight Retention Among At-Risk Hispanic Women. *Am J Prev Med* 2021, 61(1):44-54.
410. Pan WL, Gau ML, Lee TY, Jou HJ, Liu CY, Wen TK: Mindfulness-based programme on the psychological health of pregnant women. *Women Birth* 2019, 32(1):e102-e109.
411. Parikh R, Rao SR, Kukde R, O'Connor GT, Patel A, Hibberd PL: Assessing the Respiratory Effects of Air Pollution from Biomass Cookstoves on Pregnant Women in Rural India. *Int J Environ Res Public Health* 2020, 18(1).
412. Park SJ, Lee J: The effects of health care programs for gestational diabetes mellitus in South Korea: a systematic review. *Korean Journal of Women Health Nursing* 2020, 26(4):274-284.
413. Passerini L, Casey GJ, Biggs BA, Cong DT, Phu LB, Phuc TQ, Carone M, Montresor A: Increased birth weight associated with regular pre-pregnancy deworming and weekly iron-folic acid supplementation for Vietnamese women. *PLoS Negl Trop Dis* 2012, 6(4):e1608.
414. Patel A, Goudar SS, Geller SE, Kodkany BS, Edlavitch SA, Wagh K, Patted SS, Naik VA, Moss N, Derman RJ: Drape estimation vs. visual assessment for estimating postpartum hemorrhage. *Int J Gynaecol Obstet* 2006, 93(3):220-224.
415. Peaceman AM, Clifton RG, Phelan S, Gallagher D, Evans M, Redman LM, Knowler WC, Joshipura K, Haire-Joshu D, Yanovski SZ *et al*: Lifestyle Interventions Limit Gestational Weight Gain in Women with Overweight or Obesity: LIFE-Moms Prospective Meta-Analysis. *Obesity (Silver Spring)* 2018, 26(9):1396-1404.
416. Pena-Rosas JP, De-Regil LM, Garcia-Casal MN, Dowswell T: Daily oral iron supplementation during pregnancy. *Cochrane Database Syst Rev* 2015(7):CD004736.
417. Peña-Rosas JP, De-Regil LM, Malave HG, Flores-Urrutia MC, Dowswell T: Intermittent oral iron supplementation during pregnancy. In., vol. 2015; 2015.
418. Pereira Gomes Morais E, Riera R, Porfírio GJM, Macedo CR, Sarmento Vasconcelos V, de Souza Pedrosa A, Torloni MR: Chewing gum for enhancing early recovery of bowel function after caesarean section. In., vol. 2016; 2016.
419. Phelan S, Hagobian T, Brannen A, Stewart A, Schmid B, Cedars L: Promoting Weight Loss Before Pregnancy: Feasible or Futile? *Californian Journal of Health Promotion* 2013, 11(2).
420. Phelan S, Phipps MG, Abrams B, Darroch F, Grantham K, Schaffner A, Wing RR: Does behavioral intervention in pregnancy reduce postpartum weight retention? Twelve-month outcomes of the Fit for Delivery randomized trial. *Am J Clin Nutr* 2014, 99(2):302-311.
421. Phipps MG, Raker CA, Ware CF, Zlotnick C: Randomized controlled trial to prevent postpartum depression in adolescent mothers. *Am J Obstet Gynecol* 2013, 208(3):192 e191-196.
422. Pilarczyk R, Strozik M, Hirnle L: Diagnostic equivalency of mobile CTG devices and remote analysis to conventional on-site nonstress test. *Adv Clin Exp Med* 2020, 29(1):33-44.
423. Pineles BL, Hsu S, Park E, Samet JM: Systematic review and meta-analyses of perinatal death and maternal exposure to tobacco smoke during pregnancy. In., vol. 184; 2016.
424. Polsky S, Garcetti R, Pyle L, Joshee P, Demmitt JK, Snell-Bergeon JK: Continuous glucose monitor use with and without remote monitoring in pregnant women with type 1 diabetes: A pilot study. *PLoS One* 2020, 15(4):e0230476.

For peer review only

425. Ragnar I, Altman D, Tyden T, Olsson SE: Comparison of the maternal experience and duration of labour in two upright delivery positions--a randomised controlled trial. *BJOG* 2006, 113(2):165-170.

426. Rajbhandari S, Hodgins S, Sanghvi H, McPherson R, Pradhan YV, Baqui AH, Misoprostol Study G: Expanding uterotonic protection following childbirth through community-based distribution of misoprostol: operations research study in Nepal. *Int J Gynaecol Obstet* 2010, 108(3):282-288.

427. Ramakrishnan U, Grant FK, Goldenberg T, Bui V, Imdad A, Bhutta ZA: Effect of multiple micronutrient supplementation on pregnancy and infant outcomes: a systematic review. *Paediatr Perinat Epidemiol* 2012, 26 Suppl 1(SUPPL. 1):153-167.

428. Raman P, Shepherd E, Dowswell T, Middleton P, Crowther CA: Different methods and settings for glucose monitoring for gestational diabetes during pregnancy. In., vol. 2017; 2017.

429. Ramanathan K, Sirala Jagadeesh N, Vishwanath U, Dayal C, Chandrababu R, Hayter M: Efficacy of supplementation of probiotics on maternal glycaemic control – A systematic review and meta-analysis of randomized controlled trials. In., vol. 10; 2021.

430. Rashtchi V, Maryami N, Molaei B: Comparison of entonox and transcutaneous electrical nerve stimulation (TENS) in labor pain: a randomized clinical trial study. *J Matern Fetal Neonatal Med* 2022, 35(16):3124-3128.

431. Rasmussen L, Christensen ML, Poulsen CW, Rud C, Christensen AS, Andersen JR, Kampmann U, Ovesen PG: Effect of High Versus Low Carbohydrate Intake in the Morning on Glycemic Variability and Glycemic Control Measured by Continuous Blood Glucose Monitoring in Women with Gestational Diabetes Mellitus-A Randomized Crossover Study. *Nutrients* 2020, 12(2).

432. Rasouli M, Pourheidari M, Hamzeh Gardesh Z: Effect of Self-care Before and During Pregnancy to Prevention and Control Preeclampsia in High-risk Women. *Int J Prev Med* 2019, 10:21.

433. Redman LM, Drews KL, Klein S, Horn LV, Wing RR, Pi-Sunyer X, Evans M, Joshipura K, Arteaga SS, Cahill AG *et al*: Attenuated early pregnancy weight gain by prenatal lifestyle interventions does not prevent gestational diabetes in the LIFE-Moms consortium. *Diabetes Res Clin Pract* 2021, 171:108549.

434. Revello MG, Tibaldi C, Masuelli G, Frisina V, Sacchi A, Furione M, Arossa A, Spinillo A, Klersy C, Ceccarelli M *et al*: Prevention of Primary Cytomegalovirus Infection in Pregnancy. *EBioMedicine* 2015, 2(9):1205-1210.

435. Rezaeean SM, Abedian Z, Latifnejad-Roudsari R, Mazloum SR, Abbasi Z: The Effect of Prenatal Self-Care Based on Orem's Theory on Preterm Birth Occurrence in Women at Risk for Preterm Birth. *Iran J Nurs Midwifery Res* 2020, 25(3):242-248.

436. Rigla M, Martinez-Sarriegui I, Garcia-Saez G, Pons B, Hernando ME: Gestational Diabetes Management Using Smart Mobile Telemedicine. *J Diabetes Sci Technol* 2018, 12(2):260-264.

437. Robertson N, Okano S, Kumar S: Sleep in the Supine Position during Pregnancy Is Associated with Fetal Cerebral Redistribution. *J Clin Med* 2020, 9(6).

438. Rodriguez-Blanque R, Sanchez-Garcia JC, Sanchez-Lopez AM, Mur-Villar N, Aguilar-Cordero MJ: The influence of physical activity in water on sleep quality in pregnant women: A randomised trial. *Women Birth* 2018, 31(1):e51-e58.

439. Rondung E, Ternstrom E, Hildingsson I, Haines HM, Sundin O, Ekdahl J, Karlstrom A, Larsson B, Segeblad B, Baylis R *et al*: Comparing Internet-Based Cognitive Behavioral Therapy With Standard Care for Women With Fear of Birth: Randomized Controlled Trial. *JMIR Ment Health* 2018, 5(3):e10420.

440. Rong L, Wang R, Ouyang YQ, Redding SR: Efficacy of yoga on physiological and psychological discomforts and delivery outcomes in Chinese primiparas. *Complement Ther Clin Pract* 2021, 44:101434.

441. Rotheram-Borus MJ, Tomlinson M, le Roux IM, Harwood JM, Comulada S, O'Connor MJ, Weiss RE, Worthman CM: A cluster randomised controlled effectiveness trial evaluating perinatal home visiting among South African mothers/infants. *PLoS One* 2014, 9(10):e105934.

442. Rumbold A, Ota E, Hori H, Miyazaki C, Crowther CA: Vitamin E supplementation in pregnancy. In., vol. 2016; 2015.

For peer review only

443. Rungsiprakarn P, Laopaiboon M, Sangkomkamhang US, Lumbiganon P, Pratt JJ: Interventions for treating constipation in pregnancy. In., vol. 2015; 2015.

444. Saastad E, Israel P, Ahlborg T, Gunnes N, Froen JF: Fetal movement counting--effects on maternal-fetal attachment: a multicenter randomized controlled trial. *Birth* 2011, 38(4):282-293.

445. Saccone G, Berghella V, Venturella R, D'Alessandro P, Arduino B, Raffone A, Giudicepietro A, Visentin S, Vitagliano A, Martinelli P *et al*: Effects of exercise during pregnancy in women with short cervix: Secondary analysis from the Italian Pessary Trial in singletons. *Eur J Obstet Gynecol Reprod Biol* 2018, 229:132-136.

446. Saccone G, Caissutti C, Ciardulli A, Berghella V: Uterine massage for preventing postpartum hemorrhage at cesarean delivery: Which evidence? In., vol. 223; 2018.

447. Sagedal LR, Overby NC, Bere E, Torstveit MK, Lohne-Seiler H, Smastuen M, Hillesund ER, Henriksen T, Vistad I: Lifestyle intervention to limit gestational weight gain: the Norwegian Fit for Delivery randomised controlled trial. *BJOG* 2017, 124(1):97-109.

448. Sajitha AT, Philip AT, Sarika TK: Effectiveness of Prenatal Education Regarding Practice on Antenatal Exercises and Minor Ailments among Pregnant Mothers. *Journal of Clinical and Diagnostic Research* 2018, 12(9).

449. Salam RA, Zuberi NF, Bhutta ZA: Pyridoxine (vitamin B6) supplementation during pregnancy or labour for maternal and neonatal outcomes. In., vol. 2016; 2015.

450. Samawi L, Williams PP, Myers B, Fuhr DC: Effectiveness of psychological interventions to reduce alcohol consumption among pregnant and postpartum women: a systematic review. In., vol. 24; 2021.

451. Sampselle CM, Miller JM, Mims BL, Delancey JO, Ashton-Miller JA, Antonakos CL: Effect of pelvic muscle exercise on transient incontinence during pregnancy and after birth. *Obstet Gynecol* 1998, 91(3):406-412.

452. Sandborg J, Soderstrom E, Henriksson P, Bendtsen M, Henstrom M, Leppanen MH, Maddison R, Migueles JH, Blomberg M, Lof M: Effectiveness of a Smartphone App to Promote Healthy Weight Gain, Diet, and Physical Activity During Pregnancy (HealthyMoms): Randomized Controlled Trial. *JMIR Mhealth Uhealth* 2021, 9(3):e26091.

453. Sanghvi H, Ansari N, Prata NJ, Gibson H, Ehsan AT, Smith JM: Prevention of postpartum hemorrhage at home birth in Afghanistan. *Int J Gynaecol Obstet* 2010, 108(3):276-281.

454. Sapkota D, Baird K, Saito A, Anderson D: Interventions for reducing and/or controlling domestic violence among pregnant women in low- and middle-income countries: A systematic review. In., vol. 8; 2019.

455. Sapkota D, Baird K, Saito A, Rijal P, Anderson D: Antenatal-Based Pilot Psychosocial Intervention to Enhance Mental Health of Pregnant Women Experiencing Domestic and Family Violence in Nepal. *J Interpers Violence* 2022, 37(5-6):NP3605-NP3627.

456. Sarkar A, Mburu G, Shivkumar PV, Sharma P, Campbell F, Behera J, Dargan R, Mishra SK, Mehra S: Feasibility of supervised self-testing using an oral fluid-based HIV rapid testing method: a cross-sectional, mixed method study among pregnant women in rural India. *J Int AIDS Soc* 2016, 19(1):20993.

457. Scott EM, Feig DS, Murphy HR, Law GR: Continuous glucose monitoring in pregnancy: Importance of analyzing temporal profiles to understand clinical outcomes. In*: 2020*.

458. Secher AL, Ringholm L, Andersen HU, Damm P, Mathiesen ER: The effect of real-time continuous glucose monitoring in pregnant women with diabetes: a randomized controlled trial. *Diabetes Care* 2013, 36(7):1877-1883.

459. Sehhatie F, Mirgafourvand M, Vosoughi Niri Z: The Effect of Non-Pharmaceutical Methods of Labor Pain Relief on Mothers' Postpartum Depression: A Randomized Controlled Trial. *International Journal of Women's Health and Reproduction Sciences* 2015, 3(1):48-55.

460. Seneviratne SN, Jiang Y, Derraik J, McCowan L, Parry GK, Biggs JB, Craigie S, Gusso S, Peres G, Rodrigues RO *et al*: Effects of antenatal exercise in overweight and obese pregnant women on maternal and perinatal outcomes: a randomised controlled trial. *BJOG* 2016, 123(4):588-597.

For peer review only

461. Senol DK, Aslan E: The Effects of Cold Application to the Perineum on Pain Relief After Vaginal Birth. *Asian Nurs Res (Korean Soc Nurs Sci)* 2017, 11(4):276-282.
462. Sercekus P, Baskale H: Effects of antenatal education on fear of childbirth, maternal self-efficacy and parental attachment. *Midwifery* 2016, 34:166-172.
463. Serra R, Peñailillo R, Monteiro LJ, Monckeberg M, Peña M, Moyano L, Brunner C, Vega G, Choolani M, Illanes SE: Supplementation of omega 3 during pregnancy and the risk of preterm birth: A systematic review and meta-analysis. In., vol. 13; 2021.
464. Sethi V, Bhanot A, Bhattacharjee S, Gope R, Sarangi D, Nath V, Nair N, Singh U, Daniel A, Parhi RN *et al*: Integrated multisectoral strategy to improve girls' and women's nutrition before conception, during pregnancy and after birth in India (Swabhimaan): protocol for a prospective, non-randomised controlled evaluation. *BMJ Open* 2019, 9(11):e031632.
465. Shahsavan F, Akbari N, Gharraee B, Abolghasemi J, Khedmat L: The effect of internet-based guided self-help cognitive-behavioral therapies on Iranian women's psychological symptoms and preferred method of childbirth. *Perspect Psychiatr Care* 2021, 57(1):138-147.
466. Sharps PW, Bullock LF, Campbell JC, Alhusen JL, Ghazarian SR, Bhandari SS, Schminkey DL: Domestic Violence Enhanced Perinatal Home Visits: The DOVE Randomized Clinical Trial. *J Womens Health (Larchmt)* 2016, 25(11):1129-1138.
467. Shedmake PV, Wakode SR: A Hospital-Based Randomized Controlled Trial-Comparing the Outcome of Normal Delivery Between Squatting and Lying Down Positions During Labour. *J Obstet Gynaecol India* 2021, 71(4):393-398.
468. Shirah BH, Shirah HA, Fallata AH, Alobidy SN, Hawsawi MMA: Hemorrhoids during pregnancy: Sitz bath vs. ano-rectal cream: A comparative prospective study of two conservative treatment protocols. *Women Birth* 2018, 31(4):e272-e277.
469. Shivalli S, Srivastava RK, Singh GP: Trials of Improved Practices (TIPs) to Enhance the Dietary and Iron-Folate Intake during Pregnancy- A Quasi Experimental Study among Rural Pregnant Women of Varanasi, India. *PLoS One* 2015, 10(9):e0137735.
470. Shorey S, Chee CYI, Ng ED, Lau Y, Dennis CL, Chan YH: Evaluation of a Technology-Based Peer-Support Intervention Program for Preventing Postnatal Depression (Part 1): Randomized Controlled Trial. *J Med Internet Res* 2019, 21(8):e12410.
471. Shorten A, Shorten B, Keogh J, West S, Morris J: Making choices for childbirth: a randomized controlled trial of a decision-aid for informed birth after cesarean. *Birth* 2005, 32(4):252-261.
472. Sibley L, Buffington ST, Haileyesus D: The American College of Nurse-Midwives' home-based lifesaving skills program: a review of the Ethiopia field test. *J Midwifery Womens Health* 2004, 49(4):320-328.
473. Siddika N, Balogun HA, Amegah AK, Jaakkola JJK: Prenatal ambient air pollution exposure and the risk of stillbirth: Systematic review and meta-analysis of the empirical evidence. In., vol. 73; 2016.
474. Sidiki NNA, Payne VK, Cedric Y, Nadia NAC: Effect of Impregnated Mosquito Bed Nets on the Prevalence of Malaria among Pregnant Women in Foumban Subdivision, West Region of Cameroon. *J Parasitol Res* 2020, 2020:7438317.
475. Silver RM, Hunter S, Reddy UM, Facco F, Gibbins KJ, Grobman WA, Mercer BM, Haas DM, Simhan HN, Parry S *et al*: Prospective Evaluation of Maternal Sleep Position Through 30 Weeks of Gestation and Adverse Pregnancy Outcomes. *Obstet Gynecol* 2019, 134(4):667-676.
476. Simpson SA, Coulman E, Gallagher D, Jewell K, Cohen D, Newcombe RG, Huang C, Robles-Zurita JA, Busse M, Owen-Jones E *et al*: Healthy eating and lifestyle in pregnancy (HELP): a cluster randomised trial to evaluate the effectiveness of a weight management intervention for pregnant women with obesity on weight at 12 months postpartum. *Int J Obes (Lond)* 2021, 45(8):1728-1739.
477. Skouteris H, Hartley-Clark L, McCabe M, Milgrom J, Kent B, Herring SJ, Gale J: Preventing excessive gestational weight gain: a systematic review of interventions. *Obes Rev* 2010, 11(11):757-768.

For peer review only

478. Skouteris H, Morris H, Nagle C, Nankervis A: Behavior modification techniques used to prevent gestational diabetes: A systematic review of the literature topical collection on obesity. In., vol. 14; 2014.
479. Skovbjerg S, Birk D, Bruggisser S, Wolf ALA, Fjorback L: Mindfulness-based stress reduction adapted to pregnant women with psychosocial vulnerabilities-a protocol for a randomized feasibility study in a Danish hospital-based outpatient setting. *Pilot Feasibility Stud* 2021, 7(1):118.
480. Slade P, West H, Thomson G, Lane S, Spiby H, Edwards RT, Charles JM, Garrett C, Flanagan B, Treadwell M *et al*: STRAWB2 (Stress and Wellbeing After Childbirth): a randomised controlled trial of targeted self-help materials to prevent post-traumatic stress disorder following childbirth. *BJOG* 2020, 127(7):886-896.
481. Smith CA, Collins CT, Crowther CA: Aromatherapy for pain management in labour. *Cochrane Database Syst Rev* 2011, 14(7):CD009215.
482. Smith CA, Collins CT, Levett KM, Armour M, Dahlen HG, Tan AL, Mesgarpour B: Acupuncture or acupressure for pain management during labour. In., vol. 2020; 2020.
483. Smith CA, Hill E, Denejkina A, Thornton C, Dahlen HG: The effectiveness and safety of complementary health approaches to managing postpartum pain: A systematic review and meta-analysis. In., vol. 11; 2022.
484. Smith CA, Levett KM, Collins CT, Armour M, Dahlen HG, Suganuma M: Relaxation techniques for pain management in labour. In., vol. 2018; 2018.
485. Smith CA, Levett KM, Collins CT, Dahlen HG, Ee CC, Suganuma M: Massage, reflexology and other manual methods for pain management in labour. In., vol. 2018; 2018.
486. Smith ER, Shankar AH, Wu LS, Aboud S, Adu-Afarwuah S, Ali H, Agustina R, Arifeen S, Ashorn P, Bhutta ZA *et al*: Modifiers of the effect of maternal multiple micronutrient supplementation on stillbirth, birth outcomes, and infant mortality: a meta-analysis of individual patient data from 17 randomised trials in low-income and middle-income countries. *Lancet Glob Health* 2017, 5(11):e1090-e1100.
487. Smith JM, Gubin R, Holston MM, Fullerton J, Prata N: Misoprostol for postpartum hemorrhage prevention at home birth: an integrative review of global implementation experience to date. *BMC Pregnancy Childbirth* 2013, 13:44.
488. Smith SA, Michel Y: A pilot study on the effects of aquatic exercises on discomforts of pregnancy. *J Obstet Gynecol Neonatal Nurs* 2006, 35(3):315-323.
489. Smyth RMD, Aflaifel N, Bamigboye AA: Interventions for varicose veins and leg oedema in pregnancy. In., vol. 2015; 2015.
490. Sobhgol SS, Priddis H, Smith CA, Dahlen HG: Evaluation of the effect of an antenatal pelvic floor muscle exercise programme on female sexual function during pregnancy and the first 3 months following birth: study protocol for a pragmatic randomised controlled trial. *Trials* 2019, 20(1):144.
491. Soltani H, Scott AM: Antenatal breast expression in women with diabetes: outcomes from a retrospective cohort study. *Int Breastfeed J* 2012, 7(1):18.
492. Srisopa P, Cong X, Russell B, Lucas R: The Role of Emotion Regulation in Pain Management Among Women From Labor to Three Months Postpartum: An Integrative Review. *Pain Manag Nurs* 2021, 22(6):783-790.
493. Stade BC, Bailey C, Dzendoletas D, Sgro M, Dowswell T, Bennett D: Psychological and/or educational interventions for reducing alcohol consumption in pregnant women and women planning pregnancy. In.; 2009.
494. Steen M, Briggs M, King D: Alleviating postnatal perineal trauma: To cool or not to cool? In., vol. 14; 2006.
495. Steen M, Cooper K, Marchant P, Griffiths-Jones M, Walker J: A randomised controlled trial to compare the effectiveness of ice-packs and Epifoam with cooling maternity gel pads at alleviating postnatal perineal trauma. *Midwifery* 2000, 16(1):48-55.
496. Steen M, Robinson M, Robertson S, Raine G: Pre and post survey findings from the Mind 'Building resilience programme for better mental health: Pregnant women and new mothers'. In., vol. 13; 2015.
497. Stith BJ, Buls SM, Keim SA, Thung SF, Klebanoff MA, Landon MB, Gabbe SG, Gandhi KK, Oza-Frank R: Moms in motion: weight loss intervention for postpartum mothers after gestational diabetes: a randomized controlled trial. *BMC Pregnancy Childbirth* 2021, 21(1):461.

For peer review only

498. Strauss RA, Eucker B, Savitz DA, Thorp JM, Jr.: Diagnosis of bacterial vaginosis from self-obtained vaginal swabs. *Infect Dis Obstet Gynecol* 2005, 13(1):31-35.

499. Sun Y, Li Y, Wang J, Chen Q, Bazzano AN, Cao F: Effectiveness of Smartphone-Based Mindfulness Training on Maternal Perinatal Depression: Randomized Controlled Trial. *J Med Internet Res* 2021, 23(1):e23410.

500. Sun YC, Hung YC, Chang Y, Kuo SC: Effects of a prenatal yoga programme on the discomforts of pregnancy and maternal childbirth self-efficacy in Taiwan. *Midwifery* 2010, 26(6):e31-36.

501. Sungkar A, Purwosunu Y, Aziz MF, Pratomo H, Sutrisna B, Sekizawa A: Influence of early self-diagnosis and treatment of bacterial vaginosis on preterm birth rate. *Int J Gynaecol Obstet* 2012, 117(3):264-267.

502. Suntarattiwong P, Mott JA, Mohanty S, Sinthuwattanawibool C, Srisantiroj N, Patamasingh Na Ayudhaya O, Klungthong C, Fernandez S, Kim L, Hunt D *et al*: Feasibility and Performance of Self-Collected Nasal Swabs for Detection of Influenza Virus, Respiratory Syncytial Virus, and Human Metapneumovirus. *J Infect Dis* 2021, 224(5):831-838.

503. Surkan PJ, Hamdani SU, Huma ZE, Nazir H, Atif N, Rowther AA, Chaudhri R, Zafar S, Mullany LC, Malik A *et al*: Cognitive-behavioral therapy-based intervention to treat symptoms of anxiety in pregnancy in a prenatal clinic using non-specialist providers in Pakistan: design of a randomised trial. *BMJ Open* 2020, 10(4):e037590.

504. Sushko K, Menezes HT, Strachan P, Butt M, Sherifali D: Self-management education among women with pre-existing diabetes in pregnancy: A scoping review. In., vol. 117; 2021.

505. Syngelaki A, Sequeira Campos M, Roberge S, Andrade W, Nicolaides KH: Diet and exercise for preeclampsia prevention in overweight and obese pregnant women: systematic review and meta-analysis. In., vol. 32; 2019.

506. Szkwara JM, Milne N, Rathbone E: A prospective quasi-experimental controlled study evaluating the use of dynamic elastomeric fabric orthoses to manage common postpartum ailments during postnatal care. *Womens Health (Lond)* 2020, 16:1745506520927196.

507. Szumilewicz A, Dornowski M, Piernicka M, Worska A, Kuchta A, Kortas J, Bludnicka M, Radziminski L, Jastrzebski Z: High-Low Impact Exercise Program Including Pelvic Floor Muscle Exercises Improves Pelvic Floor Muscle Function in Healthy Pregnant Women - A Randomized Control Trial. *Front Physiol* 2018, 9:1867.

508. Taavoni S, Sheikhan F, Abdolahian S, Ghavi F: Birth ball or heat therapy? A randomized controlled trial to compare the effectiveness of birth ball usage with sacrum-perineal heat therapy in labor pain management. *Complement Ther Clin Pract* 2016, 24:99-102.

509. Taheri Z, Mazaheri MA, Khorsandi M, Hassanzadeh A, Amiri M: Effect of educational intervention on self-efficacy for choosing delivery method among pregnant women in 2013. *International Journal of Preventive Medicine* 2014, 5(10).

510. Tan PC, Andi A, Azmi N, Noraihan MN: Effect of coitus at term on length of gestation, induction of labor, and mode of delivery. *Obstet Gynecol* 2006, 108(1):134-140.

511. Tan XYJ, Choong SYX, Cheng LJ, Lau Y: Relaxation interventions for improving sleep outcomes in perinatal women: A systematic review and meta-analysis of randomized controlled trials. In., vol. 103; 2021.

512. Tanvig MH, Jensen DM, Andersen MS, Ovesen PG, Jorgensen JS, Vinter CA: Vitamin D levels were significantly higher during and after lifestyle intervention in pregnancy: A randomized controlled trial. *Acta Obstet Gynecol Scand* 2020, 99(3):350-356.

513. Tatala SR, Ash D, Makola D, Latham M, Ndosi G, Grohn Y: Effect of micronutrient fortified beverage on nutritional anaemia during pregnancy. *East Afr Med J* 2002, 79(11):598-603.

514. ter Kuile FO, Terlouw DJ, Phillips-Howard PA, Hawley WA, Friedman JF, Kariuki SK, Shi YP, Kolczak MS, Lal AA, Vulule JM *et al*: Reduction of malaria during pregnancy by permethrin-treated bed nets in an area of intense perennial malaria transmission in western Kenya. *Am J Trop Med Hyg* 2003, 68(4 Suppl):50-60.

515. Thompson MG, Ferber JR, Odouli R, David D, Shifflett P, Meece JK, Naleway AL, Bozeman S, Spencer SM, Fry AM *et al*: Results of a pilot study using self-collected mid-turbinate nasal swabs for detection of influenza virus infection among pregnant women. *Influenza Other Respir Viruses* 2015, 9(3):155-160.

For peer review only

516. Timur Tashan S, Kafkasli A: The effect of bitter almond oil and massaging on striae gravidarum in primiparaous women. *J Clin Nurs* 2012, 21(11-12):1570-1576.

517. Tinius R, Edens K, Link K, Jones MS, Lyons S, Rebelle T, Pearson KJ, Maples J: Effect of Evidence-Based Materials and Access to Local Resources on Physical Activity Levels, Beliefs, and Motivation During Pregnancy in a Rural Setting. *J Phys Act Health* 2020, 17(10):947-957.

518. Tixier H, Boucard C, Ferdynus C, Douvier S, Sagot P: Interest of using an underbuttocks drape with collection pouch for early diagnosis of postpartum hemorrhage. *Arch Gynecol Obstet* 2011, 283(1):25-29.

519. Todd CS, Chowdhury Z, Mahmud Z, Islam N, Shabnam S, Parvin M, Bernholc A, Martinez A, Aktar B, Afsana K *et al*: Maternal nutrition intervention and maternal complications in 4 districts of Bangladesh: A nested cross-sectional study. *PLoS Med* 2019, 16(10):e1002927.

520. Tragea C, Chrousos GP, Alexopoulos EC, Darviri C: A randomized controlled trial of the effects of a stress management programme during pregnancy. *Complement Ther Med* 2014, 22(2):203-211.

521. Tran K, Padwal R, Khan N, Wright MD, Chan WS: Home blood pressure monitoring in the diagnosis and treatment of hypertension in pregnancy: a systematic review and meta-analysis. *CMAJ Open* 2021, 9(2):E642-E650.

522. Tsai YJ, Hsu YY, Hou TW, Chang CH: Effects of a Web-Based Antenatal Care System on Maternal Stress and Self-Efficacy During Pregnancy: A Study in Taiwan. *J Midwifery Womens Health* 2018, 63(2):205-213.

523. Tseng YF, Chen CH, Lee CS: Effects of listening to music on postpartum stress and anxiety levels. *J Clin Nurs* 2010, 19(7-8):1049-1055.

524. Tucker KL, Bowen L, Crawford C, Mallon P, Hinton L, Lee MM, Oke J, Taylor KS, Heneghan C, Bankhead C *et al*: The feasibility and acceptability of self-testing for proteinuria during pregnancy: A mixed methods approach. *Pregnancy Hypertens* 2018, 12:161-168.

525. Tucker KL, Taylor KS, Crawford C, Hodgkinson JA, Bankhead C, Carver T, Ewers E, Glogowska M, Greenfield SM, Ingram L *et al*: Blood pressure self-monitoring in pregnancy: examining feasibility in a prospective cohort study. *BMC Pregnancy Childbirth* 2017, 17(1):442.

526. Tumminia A, Milluzzo A, Festa C, Fresa R, Pintaudi B, Scavini M, Vitacolonna E, Napoli A, Sciacca L: Efficacy of flash glucose monitoring in pregnant women with poorly controlled pregestational diabetes (FlashMom): A randomized pilot study. *Nutr Metab Cardiovasc Dis* 2021, 31(6):1851-1859.

527. Uğurlu M, Yavan T, Karaşahin KE: The effect of an education and counseling program on maternal/neonatal outcomes in pregnant women at risk of preeclampsia. *Puerto Rico Health Sciences Journal* 2021, 40(3).

528. Vakilian K, Atarha M, Bekhradi R, Chaman R: Healing advantages of lavender essential oil during episiotomy recovery: a clinical trial. *Complement Ther Clin Pract* 2011, 17(1):50-53.

529. Vamos CA, Thompson EL, Avendano M, Daley EM, Quinonez RB, Boggess K: Oral health promotion interventions during pregnancy: A systematic review. In., vol. 43; 2015.

530. Van der Pligt P, Willcox J, Hesketh KD, Ball K, Wilkinson S, Crawford D, Campbell K: Systematic review of lifestyle interventions to limit postpartum weight retention: Implications for future opportunities to prevent maternal overweight and obesity following childbirth. In., vol. 14; 2013.

531. Van Der Windt M, Van Der Kleij RMJJ, Snoek KM, Dykgraaf RHM, Laven JSE, Schoenmakers S, Steegers-Theunissen RPM: Impact of a blended periconception lifestyle care approach combining face-to-face counseling with ehealth. *Reproductive Sciences* 2020, 27(1).

532. Van Horn L, Peaceman A, Kwasny M, Vincent E, Fought A, Josefson J, Spring B, Neff LM, Gernhofer N: Dietary Approaches to Stop Hypertension Diet and Activity to Limit Gestational Weight: Maternal Offspring Metabolics Family Intervention Trial, a Technology Enhanced Randomized Trial. *Am J Prev Med* 2018, 55(5):603-614.

533. Van Parys AS, Verhamme A, Temmerman M, Verstraelen H: Intimate partner violence and pregnancy: A systematic review of interventions. In., vol. 9; 2014.

For peer review only

534. van Poppel MN, Jelsma JGM, Simmons D, Devlieger R, Jans G, Galjaard S, Corcoy R, Adelantado JM, Dunne F, Harreiter J *et al*: Mediators of Lifestyle Behaviour Changes in Obese Pregnant Women. Secondary Analyses from the DALI Lifestyle Randomised Controlled Trial. *Nutrients* 2019, 11(2).

535. Varisoglu Y, Gungor Satilmis I: The Effects of Listening to Music on Breast Milk Production by Mothers of Premature Newborns in the Neonatal Intensive Care Unit: A Randomized Controlled Study. *Breastfeed Med* 2020, 15(7):465-470.

536. Vasegh Rahimparvar SF, Hamzehkhani M, Geranmayeh M, Rahimi R: Effect of educational software on self-efficacy of pregnant women to cope with labor: a randomized controlled trial. *Arch Gynecol Obstet* 2012, 286(1):63-70.

537. Vauloup-Fellous C, Picone O, Cordier AG, Parent-du-Chatelet I, Senat MV, Frydman R, Grangeot-Keros L: Does hygiene counseling have an impact on the rate of CMV primary infection during pregnancy? Results of a 3-year prospective study in a French hospital. *J Clin Virol* 2009, 46 Suppl 4(SUPPL. 4):S49-53.

538. Vazquez JC: Constipation, haemorrhoids, and heartburn in pregnancy. *BMJ Clin Evid* 2010, 2010.

539. Veisani Y, Jenabi E, Delpisheh A, Khazaei S: Effect of prenatal smoking cessation interventions on birth weight: meta-analysis. In., vol. 32; 2019.

540. Vesco KK, Karanja N, King JC, Gillman MW, Leo MC, Perrin N, McEvoy CT, Eckhardt CL, Smith KS, Stevens VJ: Efficacy of a group-based dietary intervention for limiting gestational weight gain among obese women: a randomized trial. *Obesity (Silver Spring)* 2014, 22(9):1989-1996.

541. Vodopivec-Jamsek V, de Jongh T, Gurol-Urganci I, Atun R, Car J: Mobile phone messaging for preventive health care. In., vol. 2017; 2012.

542. Vrana-Diaz CJ, Korte JE, Gebregziabher M, Richey L, Selassie A, Sweat M, Gichangi A: Relationship Gender Equality and Couples' Uptake of Oral Human Immunodeficiency Virus Self-Testing Kits Delivered by Pregnant Women in Kenya. *Sex Transm Dis* 2019, 46(9):588-593.

543. Wan CS, Nankervis A, Teede H, Aroni R: Dietary intervention strategies for ethnic Chinese women with gestational diabetes mellitus: A systematic review and meta-analysis. In., vol. 76; 2019.

544. Wang X, Xu X, Luo J, Chen Z, Feng S: Effect of app-based audio guidance pelvic floor muscle training on treatment of stress urinary incontinence in primiparas: A randomized controlled trial. *Int J Nurs Stud* 2020, 104:103527.

545. Wang Z, Liu Q, Min L, Mao X: The effectiveness of the laid-back position on lactation-related nipple problems and comfort: a meta-analysis. *BMC Pregnancy Childbirth* 2021, 21(1):248.

546. Wannakosit S, Phupong V: Sexual behavior in pregnancy: comparing between sexual education group and nonsexual education group. *J Sex Med* 2010, 7(10):3434-3438.

547. Warland J, Dorrian J, Morrison JL, O'Brien LM: Maternal sleep during pregnancy and poor fetal outcomes: A scoping review of the literature with meta-analysis. In., vol. 41; 2018.

548. Webber GC, Chirangi B: Women's health in women's hands: a pilot study assessing the feasibility of providing women with medications to reduce postpartum hemorrhage and sepsis in rural Tanzania. *Health Care Women Int* 2014, 35(7-9):758-770.

549. Weeks AD, Ditai J, Ononge S, Faragher B, Frye LJ, Durocher J, Mirembe FM, Byamugisha J, Winikoff B, Alfirevic Z: The MamaMiso study of self-administered misoprostol to prevent bleeding after childbirth in rural Uganda: a community-based, placebo-controlled randomised trial. *BMC Pregnancy Childbirth* 2015, 15:219.

550. Werner A, Uldbjerg N, Zachariae R, Rosen G, Nohr EA: Self-hypnosis for coping with labour pain: a randomised controlled trial. *BJOG* 2013, 120(3):346-353.

551. Whitford HM, Alder B, Jones M: A longitudinal follow up of women in their practice of perinatal pelvic floor exercises and stress urinary incontinence in North-East Scotland. *Midwifery* 2007, 23(3):298-308.

552. Willcox JC, Wilkinson SA, Lappas M, Ball K, Crawford D, McCarthy EA, Fjeldsoe B, Whittaker R, Maddison R, Campbell KJ: A mobile health intervention promoting healthy gestational weight gain for women entering

For peer review only

pregnancy at a high body mass index: the txt4two pilot randomised controlled trial. *BJOG* 2017, 124(11):1718-1728.

553. Windsor R, Woodby L, Miller T, Hardin M: Effectiveness of Smoking Cessation and Reduction in Pregnancy Treatment (SCRIPT) methods in Medicaid-supported prenatal care: Trial III. *Health Educ Behav* 2011, 38(4):412-422.

554. Windsor RA, Cutter G, Morris J, Reese Y, Manzella B, Bartlett EE, Samuelson C, Spanos D: The effectiveness of smoking cessation methods for smokers in public health maternity clinics: a randomized trial. *Am J Public Health* 1985, 75(12):1389-1392.

555. Wing DA, Rumney PJ, Preslicka CW, Chung JH: Daily cranberry juice for the prevention of asymptomatic bacteriuria in pregnancy: a randomized, controlled pilot study. *J Urol* 2008, 180(4):1367-1372.

556. Witt AM, Bolman M, Kredit S: Mothers Value and Utilize Early Outpatient Education on Breast Massage and Hand Expression in Their Self-Management of Engorgement. *Breastfeed Med* 2016, 11(9):433-439.

557. Woodley SJ, Lawrenson P, Boyle R, Cody JD, Mørkved S, Kernohan A, Hay-Smith EJC: Pelvic floor muscle training for preventing and treating urinary and faecal incontinence in antenatal and postnatal women. In., vol. 2020; 2020.

558. Woolhouse H, Mercuri K, Judd F, Brown SJ: Antenatal mindfulness intervention to reduce depression, anxiety and stress: a pilot randomised controlled trial of the MindBabyBody program in an Australian tertiary maternity hospital. *BMC Pregnancy Childbirth* 2014, 14(1):369.

559. Wulff V, Hepp P, Wolf OT, Balan P, Hagenbeck C, Fehm T, Schaal NK: The effects of a music and singing intervention during pregnancy on maternal well-being and mother-infant bonding: a randomised, controlled study. *Arch Gynecol Obstet* 2021, 303(1):69-83.

560. Yakoob MY, Qadir M, Hany OE: Vitamin A Supplementation for Prevention and Treatment of Malaria during Pregnancy and Childhood: A Systematic Review and Meta-analysis. *J Epidemiol Glob Health* 2018, 8(1-2):20-28.

561. Yan CF, Hung YC, Gau ML, Lin KC: Effects of a stability ball exercise programme on low back pain and daily life interference during pregnancy. *Midwifery* 2014, 30(4):412-419.

562. Yang X, Tian H, Zhang F, Zhang C, Li Y, Leng J, Wang L, Liu G, Dong L, Yu Z *et al*: A randomised translational trial of lifestyle intervention using a 3-tier shared care approach on pregnancy outcomes in Chinese women with gestational diabetes mellitus but without diabetes. *J Transl Med* 2014, 12(1):290.

563. Yaping X, Huifen Z, Meijing Z, Huibin H, Chunhong L, Fengfeng H, Jingjing W: Effects of Moderate-Intensity Aerobic Exercise on Blood Glucose Levels and Pregnancy Outcomes in Patients With Gestational Diabetes Mellitus: A Randomized Controlled Trial. *Diabetes Ther* 2021, 12(9):2585-2598.

564. Yerlikaya-Schatten G, Trimmal L, Rosicky I, Husslein P, Schatten C, Eppel D, Eppel W, Tura A, Gobl CS: Effects of gum chewing on glycaemic control in women with gestational diabetes mellitus: A randomized controlled trial. Impact of chewing on hyperglycaemia in women with GDM. *Eur J Obstet Gynecol Reprod Biol* 2020, 247:61-65.

565. Yew TW, Chi C, Chan SY, van Dam RM, Whitton C, Lim CS, Foong PS, Fransisca W, Teoh CL, Chen J *et al*: A Randomized Controlled Trial to Evaluate the Effects of a Smartphone Application-Based Lifestyle Coaching Program on Gestational Weight Gain, Glycemic Control, and Maternal and Neonatal Outcomes in Women With Gestational Diabetes Mellitus: The SMART-GDM Study. *Diabetes Care* 2021, 44(2):456-463.

566. Yuksel H, Cayir Y, Kosan Z, Tastan K: Effectiveness of breathing exercises during the second stage of labor on labor pain and duration: a randomized controlled trial. *J Integr Med* 2017, 15(6):456-461.

567. Zairina E, Abramson MJ, McDonald CF, Li J, Dharmasiri T, Stewart K, Walker SP, Paul E, George J: Telehealth to improve asthma control in pregnancy: A randomized controlled trial. *Respirology* 2016, 21(5):867-874.

568. Zairina E, Stewart K, Abramson MJ, George J: The effectiveness of non-pharmacological healthcare interventions for asthma management during pregnancy: a systematic review. *BMC Pulm Med* 2014, 14(1):46.

For peer review only

569. Zakarija-Grkovic I, Stewart F: Treatments for breast engorgement during lactation. *Cochrane Database Syst Rev* 2020, 9:CD006946.

570. Zang Y, Lu H, Zhang H, Huang J, Ren L, Li C: Effects of upright positions during the second stage of labour for women without epidural analgesia: A meta-analysis. In., vol. 76; 2020.

571. Zang Y, Lu H, Zhang H, Huang J, Zhao Y, Ren L: Benefits and risks of upright positions during the second stage of labour: An overview of systematic reviews. In., vol. 114; 2021.

572. Zarei MR, Khachian A, Mohammadi N, Haghani H, Rostami F: The effect of self-care education program on wound healing in women undergoing cesarean section. *Annals of Tropical Medicine and Public Health* 2018, 11(Special Issue).

573. Zarenejad M, Yazdkhasti M, Rahimzadeh M, Mehdizadeh Tourzani Z, Esmaelzadeh-Saeieh S: The effect of mindfulness-based stress reduction on maternal anxiety and self-efficacy: A randomized controlled trial. *Brain Behav* 2020, 10(4):e01561.

574. Zelkowitz P, Feeley N, Shrier I, Stremler R, Westreich R, Dunkley D, Steele R, Rosberger Z, Lefebvre F, Papageorgiou A: The cues and care randomized controlled trial of a neonatal intensive care unit intervention: effects on maternal psychological distress and mother-infant interaction. *J Dev Behav Pediatr* 2011, 32(8):591-599.

575. Zemestani M, Fazeli Nikoo Z: Effectiveness of mindfulness-based cognitive therapy for comorbid depression and anxiety in pregnancy: a randomized controlled trial. *Arch Womens Ment Health* 2020, 23(2):207-214.

576. Zhang MM, Zou Y, Li SM, Wang L, Sun YH, Shi L, Lu L, Bao YP, Li SX: The efficacy and safety of omega-3 fatty acids on depressive symptoms in perinatal women: a meta-analysis of randomized placebo-controlled trials. *Transl Psychiatry* 2020, 10(1):193.

577. Zhang X, Lin P, Sun J, Sun Y, Shao D, Cao D, Cao F: Prenatal stress self-help mindfulness intervention via social media: a randomized controlled trial. *J Ment Health* 2021:1-10.

578. Zhao Y, Lin Q, Zhu X, Wang J: Randomized Clinical Trial of a Prenatal Breastfeeding and Mental Health Mixed Management Intervention. *J Hum Lact* 2021, 37(4):761-774.

579. Zhu YP, Wang WJ, Zhang SL, Dai B, Ye DW: Effects of gum chewing on postoperative bowel motility after caesarean section: A meta-analysis of randomised controlled trials. In., vol. 121; 2014.

580. Zlotnick C, Tzilos G, Miller I, Seifer R, Stout R: Randomized controlled trial to prevent postpartum depression in mothers on public assistance. *J Affect Disord* 2016, 189:263-268.

For peer review only

Supplementary File S7. Complex interventions and their characteristics (N=35)

A. Self-care activities featured as a component of complex interventions

Self-care activity	No. of studies (%)
**Diet & nutrition**	
Dietary adjustment	19 (54%)
Dietary intake - Iodine	1 (3%)
Supplement intake - Calcium	2 (6%)
Supplement intake - Iron and/or folic acid (IFA)	5 (14%)
Supplement intake - not otherwise specified	2 (6%)
Caffeine intake reduction	1 (3%)
**Physical activity**	
Birthing/exercise balls	3 (9%)
Pelvic floor exercises	1 (3%)
Yoga	2 (6%)
Physical activity (not otherwise specified)	14 (40%)
**Lifestyle adjustments**	
Air pollution avoidance	1 (3%)
Alcohol use reduction	6 (17%)
Avoiding strenuous activities	3 (9%)
Deworming	1 (3%)
Drug use reduction	2 (6%)
Oral hygiene	2 (6%)
Insecticide-treated nets	1 (3%)
Sleep hygiene	4 (11%)
Smoking cessation	5 (14%)
TORCH prevention	1 (3%)
Urogenital hygiene	3 (9%)
Other risk reduction strategies (traffic accident, X-ray exposure)	1 (3%)
General self-care to relieve physiological discomforts (not otherwise specified)	6 (17%)
**Complementary & alternative therapies**	
Acupressure	1 (3%)
Aromatherapy	2 (6%)
General massage	9 (26%)
Shower/bath	2 (6%)
**Psycho-social strategies**	
Interpersonal relationship & support network	10 (29%)
Mind-body relaxation	16 (46%)
Music therapy	4 (11%)
Other emotion management strategies	11 (31%)
**Breast & nipple care**	
Breast/nipple care (not otherwise specified)	3 (9%)
Breastfeeding techniques	12 (34%)
**Sexual health & postpartum family planning**	
Condom use	2 (6%)
HIV disclosure	2 (6%)

Self-care activity	No. of studies (%)
Intimate partner violence (IPV) prevention	3 (9%)
Safe and healthy sexual life	2 (6%)
Sexual negotiation	1 (3%)
STI prevention (not otherwise specified)	1 (3%)
**Self-monitoring**	
Foetal movement monitoring	2 (6%)
Home-based records	2 (6%)
Recognising signs of labour	6 (17%)
Recognising signs of complications	6 (17%)
Self-monitoring of uterine contraction	1 (3%)
Self-monitoring weight	5 (14%)
Self-monitoring blood glucose	2 (6%)
Self-monitoring blood pressure	1 (3%)
**Self-management of medication**	
Self-medication	2 (6%)
Adherence to PMTCT & ART	1 (3%)
**Other self-management skills**	
Birth plans	7 (20%)
Birth position	5 (14%)
Techniques during childbirth (not otherwise specified)	3 (9%)
Postoperative wound care	1 (3%)

Note: Each of these self-care activities is often a component of a larger intervention, which feature more than 3 different self-care activities.

B. Health issues targeted by complex interventions

Target outcomes	No. of studies (%)
Care-seeking behaviours	1 (3%)
Diabetes mellitus	2 (6%)
Fatigue	1 (3%)
HIV/STDs	2 (6%)
Hypertensive disorders of pregnancy	1 (3%)
Low birth weight	1 (3%)
Nutritional deficiencies	2 (6%)
Physiological discomforts	1 (3%)
Positive childbirth experience	9 (26%)
Postpartum health	1 (3%)
Preconception health	3 (9%)
Preterm birth	1 (3%)
Psychological wellbeing	11 (31%)
Various maternal & neonatal outcomes	3 (9%)
Weight management	3 (9%)

Supplementary File S8. List of all self-care interventions identified from included studies

Self-care intervention	Description	No. of studies (%) N=545	Health issues / outcomes targeted by intervention
**Diet & nutrition**		**143 (26%)**	
Dietary adjustment	Modifying daily intakes of vegetables, fruit, water, salt, fat, sugar, etc. or total calorie consumption	75 (14%)	Birth weight, diabetes mellitus, hypertensive disorders of pregnancy, nutritional deficiencies, physiological discomforts, preconception health, preterm birth, psychological wellbeing, weight management
Dietary intake - Calcium	From diet (dairy products and green leafy vegetables)	1 (0.2%)	Various maternal & neonatal outcomes
Dietary intake - Omega-3 fatty acids	From diet (fish, nuts and seeds, plant oils)	1 (0.2%)	Psychological wellbeing
Supplement intake - Calcium		5 (1%)	Hypertensive disorders of pregnancy, nutritional deficiencies, preterm birth
Supplement intake - Iodine		2 (0.4%)	Various maternal & neonatal outcomes
Supplement intake - Iron and/or folic acid (IFA)	Potential interaction with some medications e.g. malaria chemoprophylaxis<br>Including self-management of weekly IFA supplements (n=2)	12 (2%)	Diabetes mellitus, hypertensive disorders of pregnancy, low birth weight, nutritional deficiencies, preconception health, preterm birth
Supplement intake - Lipid-based nutrients	Fortified products which provide energy mainly in the form of lipids, and additionally other nutrients (protein, essential fatty acids, micronutrients)	2 (0.4%)	Various maternal & neonatal outcomes
Supplement intake - Multiple-micronutrients	Supplements containing 13-15 micronutrients (vitamins and minerals) in one tablet, including UNIMMAP formulation	9 (2%)	Diabetes mellitus, nutritional deficiencies, preterm birth, other maternal & neonatal outcomes
Supplement intake - Omega-3 fatty acids	Including DHA, EHA, fish oils etc.	11 (2%)	Diabetes mellitus, preeclampsia, preterm birth, postpartum depression
Supplement intake - Vitamin A	Only recommended in areas with endemic vitamin A deficiency or night blindness	2 (0.4%)	Bacterial vaginosis, malaria, nutritional deficiencies, preterm birth
Supplement intake - Vitamin B		1 (0.2%)	Various maternal & neonatal outcomes
Supplement intake - Vitamin C		2 (0.4%)	Urinary tract infections, other maternal & neonatal outcomes
Supplement intake - Vitamin D		2 (0.4%)	Diabetes mellitus, other maternal & neonatal outcomes
Supplement intake - Vitamin E		1 (0.2%)	Various maternal & neonatal outcomes
Supplement intake - Zinc		2 (0.4%)	Preterm birth, other maternal & neonatal outcomes
Supplement intake - not otherwise specified		1 (0.2%)	Hypertensive disorders of pregnancy
Caffeine intake reduction		1 (0.2%)	Various maternal & neonatal outcomes
Energy drinks during childbirth	Drinks containing various nutrients and electrolytes	1 (0.2%)	Positive labour outcomes
Fibre supplement	Psyllium, methycellulose, calcium polycarbophil etc. that function as bulk-forming laxatives	3 (1%)	Constipation, haemorrhoids, physiological discomforts
Gum chewing	Sugar-free gum for chewing to induce satiation or bowel movement	5 (1%)	Diabetes mellitus, positive childbirth experience, postoperative bowel function
Probiotics	Products containing Lactobacillus, Bifidobacterium and/or Enterococcus sp.	19 (3%)	Bacterial vaginosis, birth weight, diabetes mellitus, group B streptococcus infection, mastitis, preterm birth, psychological wellbeing, weight management
Other dietary/nutritional products	Fruit juices, herbal products, any other product containing natural ingredients that were not regulated as medicine	13 (2%)	Diabetes mellitus, induction of labour, mastitis, physiological discomforts, postpartum healing & pain relief, psychological wellbeing, sleep quality, urinary tract infections
**Physical activity**		**132 (24%)**	
Ankle weights	Worn during daily activities as a resistance exercise	1 (0.2%)	Weight management
Aquatic exercises	Exercises conduct under water (e.g. in a swimming pool)	4 (1%)	Labour pain, lumbopelvic pain, physiological discomforts, psychological wellbeing, sleep quality
Birthing/exercise balls	Exercises using Swiss balls, exercise balls or peanut balls, etc.	7 (1%)	Childbirth self-efficiency (CBSE), labour pain, lumbopelvic pain, positive childbirth experience, positive labour outcomes

Self-care intervention	Description	No. of studies (%) N=545	Health issues / outcomes targeted by intervention
Pelvic floor exercises	Exercises aimed at strengthening pelvic floor muscles, e.g. Kegel exercises	17 (3%)	Diabetes mellitus, lumbopelvic pain, pelvic floor disorders, physiological discomforts, positive childbirth experience, sexual functioning, weight management
Yoga	Physical component (yoga poses) only; meditation component was classified as mind-body relaxation	15 (3%)	Diabetes mellitus, labour pain, lumbopelvic pain, physiological discomforts, positive childbirth experience, psychological wellbeing, sleep quality
Physical activity (not otherwise specified)		99 (18%)	Birth weight, cardiometabolic fitness, diabetes mellitus, fatigue, foetal health, hypertensive disorders of pregnancy, lumbopelvic pain, pelvic floor disorders, physiological discomforts, preconception health, preeclampsia, preterm birth, psychological wellbeing, sleep quality, weight management
**Lifestyle adjustments**		**90 (17%)**	
Air pollution avoidance	Avoiding going outdoor when air pollution levels are high	3 (1%)	Air pollution exposure, stillbirth
Alcohol use reduction	Self-initiated abstinence or relapse prevention without pharmacological treatment	7 (1%)	Alcohol consumption
Avoiding strenuous activities	Sleeping with pillows, ergonomic postures in daily activities (lifting, bending), etc.	2 (0.4%)	Lumbopelvic pain
Bed rest during pregnancy	Ranging from selected activity restriction to strict bed rest	1 (0.2%)	Various maternal & neonatal outcomes
Clean cookstoves	Cookstoves using cleaner energy sources (electricity, ethanol or biogas) instead of open fire, kerosene or biomass	5 (1%)	Hypertensive disorders of pregnancy, low birth weight, respiratory irritation
General self-care to relieve physiological discomforts (not otherwise specified)		3 (1%)	Psychological wellbeing
Hand hygiene	Washing hands, avoiding exposure to saliva from young children (sharing food and utensils, kissing)	5 (1%)	Cytomegalovirus infection
Oral hygiene	Tooth brushing, flossing, using oral rinses, etc.	6 (1%)	Periodontal diseases
Mosquito-repellent strategies - Insecticide-treated nets	Bed nets impregnated with insecticides (e.g. pyrroles, pyrethroids)	12 (2%)	Low birth weight, malaria, preterm birth
Mosquito-repellent strategies - Other	Use of insecticides, mosquito coils, fans, insect-repellent cream, wearing long sleeves, burning local plants, etc.	1 (0.2%)	Malaria
Secondhand smoke exposure prevention	Establishing a smoke-free home/office, communicating with smokers	3 (1%)	Secondhand smoke exposure
Sleep hygiene	Sleeping schedules, good sleep habits, improving sleeping environment	4 (1%)	Fatigue, sleep quality
Sleeping position	Choosing to left lateral recumbent sleeping position / avoiding supine position, raising the head to relieve heartburn	6 (1%)	Hypertensive disorders of pregnancy, low birth weight, physiological discomforts, stillbirth
Smoking cessation	Self-initiated cessation or relapse prevention without pharmacological treatment	32 (6%)	Low birth weight, neonatal death, smoking status, stillbirth, other maternal & neonatal outcomes
Urogenital hygiene	Hygiene behaviours (daily cleaning, using clean water, using pads)	2 (0.4%)	Sexual functioning, urinary tract infections
**Complementary & alternative therapies**		**64 (12%)**	
Acupressure	Applying pressure on specific acupoints. Only manoeuvres that can be administered by the woman herself or her partner are included; reflexology by HCWs is excluded.	13 (2%)	Breast engorgement, labour pain, mastitis, perineal healing & pain relief, physiological discomforts, positive labour outcomes, postpartum healing & pain relief
Aromatherapy	Including the use of essential oils	7 (1%)	Labour pain, perineal healing & pain relief, psychological wellbeing, sleep quality
Bright light therapy	Exposure to natural light (getting outdoor) or artificial light (using a light therapy lamp)	3 (1%)	Psychological wellbeing
Compress - cold	Ice packs, cryotherapy	9 (2%)	Labour pain, perineal healing & pain relief

Self-care intervention	Description	No. of studies (%) N=545	Health issues / outcomes targeted by intervention
Compress - hot	Hot water packs, heat therapy	3 (1%)	Labour pain, perineal healing & pain relief
Compression stockings	Stockings designed to promote circulation in the legs via gentle compression	1 (0.2%)	Physiological discomforts
Continuous positive airway pressure (CPAP)	Delivering positive airway pressure to keep airway open during sleep. CPAP machines are available over-the-counter and do not require specialised skills to operate.	1 (0.2%)	Diabetes mellitus, obstructive sleep apnoea
General massage	Any form of body massage	17 (3%)	Labour pain, lumbopelvic pain, physiological discomforts, positive childbirth experience, postpartum healing & pain relief, psychological wellbeing, sleep quality, striae gravidarum
Kinesiologic taping	Adhesive tape applied to the specific region of the back to provide support	1 (0.2%)	Labour pain
Pelvic support devices	Any device worn at the lower abdominal level to support the pelvic or lumbosacral region e.g. pelvic belts/girdles, abdominal binders, elastomeric fabric orthoses etc.	5 (1%)	C-section wound healing, lumbopelvic pain, postpartum healing & pain relief
Perineal massage	Can be administered by either the woman herself or the partner in the antenatal or postpartum period	4 (1%)	Positive childbirth experience, postpartum healing & pain relief, prevention of perineal injury
Shower/bath	Including birthing pools, shower before labour, foot baths and Sitz baths	15 (3%)	Duration of labour, haemorrhoids, labour pain, lumbopelvic pain, perineal healing & pain relief, physiological discomforts, positive childbirth experience, positive labour outcomes
Topical products	Ointments, creams, etc. that do not contain medicinal ingredients and are available over-the-counter	5 (1%)	Haemorrhoids, postpartum healing & pain relief, striae gravidarum
Transcutaneous electrical nerve stimulation (TENS)	Delivering small electrical impulses through electrodes placed on the skin. TENS machines are available over-the-counter and do not require specialised skills to operate.	9 (2%)	Labour pain, lumbopelvic pain, postpartum healing & pain relief
**Psycho-social strategies**		**97 (18%)**	
Interpersonal relationship & support network	Establishing social support, relationship management, communication with others	23 (4%)	Diabetes mellitus, intimate partner violence (IPV), psychological wellbeing
Mind-body relaxation	Breathing exercises, guided imagery, progressive muscle relaxation, self-hypnosis, mindfulness (in meditation exercises or daily practices)	63 (12%)	Breast & nipple pain, diabetes mellitus, hypertensive disorders of pregnancy, labour pain, lumbopelvic pain, physiological discomforts, positive childbirth experience, positive labour outcomes, postpartum healing & pain relief, psychological wellbeing, sleep quality
Music therapy	Listening to music (lullabies, classical music, nature round), either standalone or combined with other techniques (meditation, breathing)	11 (2%)	Labour pain, positive childbirth experience, postpartum healing & pain relief, psychological wellbeing, sleep quality
Other emotion management strategies	Cognitive restructuring, problem-solving, mood-tracking, etc.	32 (6%)	Diabetes mellitus, hypertensive disorders of pregnancy, positive childbirth experience, psychological wellbeing
**Breast & nipple care**		**22 (4%)**	
Breast compress (hot & cold)	Warm compress (warm towels, heat packs) to promote milk flow before breastfeeding; cold compress (cold gel packs, cold cabbage leaf) to stop/reduce milk flow in case of engorgement	2 (0.4%)	Breast engorgement
Breast milk expression	Can be conducted prenatally or postnatally (for colostrum extraction, a few hours after birth), using manual extraction, breast pumps or via breastfeeding	5 (1%)	Breast engorgement, various neonatal outcomes
Breast massage	Massaging techniques to promote blood circulation and lymphatic drainage	5 (1%)	Breast & nipple pain, breast engorgement, mastitis
Breast/nipple shells	Plastic parts worn over the breast to protect the nipples	2 (0.4%)	Breast & nipple pain
Hydrogel dressings	Adhesive hydrogel discs or pads applied over breast or nipple to form a	2 (0.4%)	Breast & nipple pain

Self-care intervention	Description	No. of studies (%) N=545	Health issues / outcomes targeted by intervention
	protective cushion, support healing and provide cooling effect		
Lanolin ointment	Applied over nipple to form a protective layer and promote healing	5 (1%)	Breast & nipple pain
Breast/nipple care (not otherwise specified)		1 (0.2%)	Breast & nipple pain
Antenatal education on breastfeeding techniques	Any technique for effective breastfeeding (e.g. timing, holding the newborn, latching) taught in the antenatal period	6 (1%)	Breast & nipple pain, breast engorgement, psychological wellbeing
**Sexual health & postpartum family planning**		**12 (2%)**	
Condom use	For STI prevention during pregnancy	2 (0.4%)	HIV/STI
HIV disclosure	Deciding when and how to disclose HIV status to family and partner	1 (0.2%)	HIV
Intimate partner violence (IPV) prevention	Safety planning, managing conflicts with partner	6 (1%)	IPV, psychological wellbeing, sexual functioning
Safe and healthy sexual life	Safe sex positions, sexual restrictions during pregnancy, restarting sex after pregnancy, coping with changing sexuality	5 (1%)	Sexual functioning
Sexual negotiation	Sexual disclosure to partners, refusing risky/unprotected sex	2 (0.4%)	HIV/STI, IPV
STI prevention (not otherwise specified)		3 (1%)	HIV/STI, sexual functioning
**Self-monitoring**		**69 (13%)**	
Foetal heartbeat self-monitoring	Using a home cardiotocography device	1 (0.2%)	Detection of abnormal foetal heart rate
Foetal movement monitoring	Counting or observing foetal movements	7 (1%)	Perinatal mortality, psychological wellbeing
Home-based records	Home-based records to keep track of multiple health indicators, such as weight, mood, diet, physical activity, medication use, vaccination records, etc.	4 (1%)	Diabetes mellitus, health service utilisation, psychological wellbeing, other maternal & neonatal outcomes
Recognising signs of labour	Differentiate false vs true labour, recognising when to notify health providers	2 (0.4%)	Positive childbirth experience
Self-assessment of foetal presentation	Self-palpation to detect spontaneous reversion to breech presentation	1 (0.2%)	Breech presentation
Self-measurement of symphysis-fundus height	Measuring distance from the symphysis pubis to the uterine fundus with a tape measure	1 (0.2%)	Intrauterine growth restriction
Self-monitoring lung function	Using handheld respiratory devices	1 (0.2%)	Asthma control
Self-monitoring of ketonuria	Using urine dipsticks	1 (0.2%)	Diabetes mellitus
Self-monitoring of uterine contraction	Self-palpation and counting contractions	1 (0.2%)	Uterine contraction awareness
Self-monitoring weight	Often accompanying lifestyle interventions for weight loss	17 (3%)	Preconception health, weight management, other maternal & neonatal outcomes
Self-monitoring blood glucose (Continuous glucose monitoring or CGM)	Using a CGM device that measures interstitial blood glucose levels. Only interventions where CGM data is accessible for pregnant women for lifestyle adjustments or insulin dose self-titration were included.	8 (1%)	Diabetes mellitus
Self-monitoring blood glucose (Flash glucose monitoring or FGM)	Similar to CGM but only displaying glucose results when activated via phone or device	1 (0.2%)	Diabetes mellitus
Self-monitoring blood glucose (not otherwise specified)	Any interventions involving SMBG using glucometers, CGM or FGM devices	16 (3%)	Diabetes mellitus, other maternal & neonatal outcomes
Self-monitoring blood pressure	Blood pressure monitors are available over-the-counter	12 (2%)	Diabetes mellitus, hypertensive disorders of pregnancy, postpartum hypertension
**Self-management of medication**		**17 (3%)**	

For peer review only - http://bmjopen.bmj.com/site/about/guidelines.xhtml

Self-care intervention	Description	No. of studies (%) N=545	Health issues / outcomes targeted by intervention
Self-adjustment of antihypertensive medication	Guided by a blood pressure-based algorithm	1 (0.2%)	Postpartum hypertension
Self-administration of antibiotics	Distributed during antenatal visits to be administered by the woman after birth	1 (0.2%)	Postpartum infection
Self-administration of misoprostol	Distributed during antenatal visits to be administered by the woman after birth	10 (2%)	Postpartum haemorrhage
Self-administration of malaria intermittent preventive treatment for pregnancy (IPTp)	All doses distributed to be administered at home, without direct observation	2 (0.4%)	Low birth weight, malaria
Self-administration of HIV oral pre-exposure prophylaxis (PrEP)	Daily oral tenofovir disoproxil fumarate/emtricitabine (TDF/FTC) for women with high risk of HIV acquisition	4 (1%)	HIV prevention
**Self-testing or sampling**		**18 (3%)**	
pH-sensitive panty liner	To identify vaginal discharge of abnormal pH	1 (0.2%)	Amniotic fluid leakage
Self-collection of nasal samples	Self-collecting samples to be sent to a central laboratory for testing, as opposed to clinic-based collection by health providers	2 (0.4%)	Viral upper respiratory tract infections
Self-collection of urine/vaginal samples	Self-collecting samples at clinic visits, as opposed to collection by health providers	5 (1%)	Bacterial vaginosis, chlamydia, human papillovirus
Self-testing for HIV	For couple self-testing, studies only providing testing rates by the partner were excluded.	5 (1%)	HIV
Self-testing for malaria	Using rapid diagnostic test kits	1 (0.2%)	Malaria
Self-testing for proteinuria	Using urinalysis dipstick-based test kits	2 (0.4%)	Hypertensive disorders of pregnancy, proteinuria
Self-testing of vaginal pH	Using pH-sensitive dipsticks	2 (0.4%)	Bacterial vaginosis, preterm birth
**Other self-management skills**		**53 (10%)**	
Birth plans	Written plans to communicate preferences and goals during labour and childbirth	2 (0.4%)	Positive labour outcomes, psychological wellbeing
Birth position	Choosing upright versus recumbent position during childbirth	12 (2%)	Duration of labour, positive labour outcomes
Techniques during childbirth (not otherwise specified)	Correct pushing techniques and timing, walking and changing positions frequently, emptying bladder	3 (1%)	Lumbopelvic pain, positive childbirth experience
Estimation of blood loss during birth by family members	Either by visual estimation, gravimetric methods or using a calibrated drape/collecting pouch. Can be administered by a family member if birth attendants are not available	6 (1%)	Postpartum haemorrhage
First-aid for complications by family members - Naturally-induced oxytocin release	To increase uterine tone in postpartum haemorrhage. Achieved via breast, nipple or uterine stimulation. Can be self-administered or administered by partner if birth attendants are not available	6 (1%)	Postpartum haemorrhage
First-aid for complications by family members (not otherwise specified)	Basic first aid training for major postpartum complications (postpartum haemorrhage, postpartum infection, seizures, etc.)	1 (0.2%)	Various maternal & neonatal outcomes
Labour induction - Castor oil	Stimulates cervical ripening via PGE2 pathway	3 (1%)	Duration of labour
Labour induction - Naturally-induced oxytocin release	Via breast or nipple stimulation, uterine stimulation or coitus	4 (1%)	Duration of labour
Making medical decisions	Making decisions for oneself or the foetus/newborn e.g. prenatal screening, mode of delivery, etc.	12 (2%)	Decisional conflict, positive childbirth experience
Postoperative wound care	Cleaning the wound area, identifying signs of infection. Applicable to C-section/episiotomy wounds and perineal injuries	2 (0.4%)	C-section wound healing, postpartum healing & pain relief
Self-management of asthma	Self-managing symptoms and medications, personalised action plan for attack episodes	3 (1%)	Asthma control

Supplementary File S9. Categorising self-care interventions based on WHO's classification framework

Category	N	%
**1.1 Individual agency**		
Interventions to promote awareness about self-care	0	0%
Interventions to promote autonomy and/or confidence to engage in self-care *	325	56%
Interventions for improving self-care capacities and capabilities *		
Interventions for improving health and digital literacy	0	0%
Interventions to promote sustained adoption of self-care practices and behaviours	0	0%
**1.2 Health information seeking**		
Acquiring health education for informed health decision-making *	22	4%
Improving health literacy to support health decision-making *		
Individuals seeking health information via on-demand information services	0	0%
Individuals health decision-making support via online or mobile tools	46	8%
**1.3 Social and community support**		
Peer mentorship and counselling	1	0.2%
Peer-to-peer action to support access to and the uptake and use of self-care interventions	42	7%
Individuals sharing health data with peers	3	1%
**1.4 Personal health tracking**		
Home-based record for health and diagnostic data	39	7%
Self-monitoring of health	70	12%
Active data capture/documentation by self-care user	32	6%
Passive data capture/documentation by device	9	2%
Use of diagnostic device at community location for self-measuring health indicator	0	0%
Tracking rational and responsible self-use of medicines and therapeutics	8	1%
**1.5 Self-diagnosis of health conditions**		
Self-testing, including to inform prevention	11	2%
Self-examining for health conditions	1	0%
Self-collection of samples to send for external testing	7	1%
Using online symptom checkers and health information	0	0%
**1.6 Self-management of health**		
Self-care for long-term health conditions	86	15%
Self-care for short-term health conditions	189	33%
Self-medication/treatment without medical prescription including for side effects	16	3%
Self-regulation of health conditions	148	26%
Self-care prevention including risk avoidance and support for physical and mental health and well-being	296	51%
Health management performed by caregivers	16	3%
Individuals procurement of safe over-the-counter medicines and products for self-care	106	18%

For peer review only

Category	N	%
**1.7 Individuals linkage to health system**		
Individuals-initiated communication with health system	5	1%
Individuals sharing health data with health workers	38	7%
Individuals identifying location of health facilities/structures	0	0%
Individuals receiving feedback from health workers	60	10%
**1.8 Individuals financial transactions for health** ***-not within the scope of inclusion of this review-***		
Expenses for over-the-counter health medicines and products that do not require prescription	0	0%
Availability or management of vouchers to individuals(s) for health services	0	0%
Expenses for prescription medicines	0	0%
Access to health insurance and/or adequate health insurance coverage	0	0%
Expenses for indirect health costs	0	0%

* These categories were considered to be equivalent during classifying, as there was insufficient information on how to differentiate between the two categories.

Discussion

The categories was based on WHO's guideline on classification of self-care interventions[1], which was published in 2021 as an initiative to standardise the language used in researching and reporting about self-care interventions. The framework describes four broad types of self-care interventions (interventions by self-carers and caregivers, by health workers, by health programme managers and by health policymakers/ legislators/regulators). For our review, we used the categories under self-care interventions for self-carers and caregivers. In classifying each study, we examined the description of its intervention in both the article and the associated protocol (if available).

As no standardised definition was provided in WHO's framework, liberty was taken in interpreting the meaning of these self-care categories when classifying our studies. For example, for interventions under category "Individual agency", we assume that these refer to interventions to raise public awareness or health/digital literacy about self-care, such as a public campaign or education programme. Therefore, digital platforms or education workshops targeting specific health issues (e.g. diabetes management or weight maintenance) is not classified under these categories.

Percentages were calculated based on the total number of studies (n=580). The percentages in the table above do not add up to 100% in some instances because some interventions can match to multiple WHO categories. For example, an intervention to increase physical activities involving a support group and a mobile app will be classified under "Self-regulation of health conditions" and "Peer-to-peer action to support access to and the uptake and use of self-care interventions".

There are several likely reasons for an absence of studies (0%) under some categories.

(a) For category 1.8 - Individuals financial transactions for health, the focus is on economic interventions, which is determined *a priori* as outside the scope of our review.

For peer review only

(b) For other categories, it can be interpreted as our review did not find any corresponding evidence of effectiveness or safety in the literature. Nonetheless, it is possible that qualitative or economic evidence still exists for these categories.

Reference

1 World Health Organization. Classification of self-care interventions for health: a shared language to describe the uses of self-care interventions. Geneva, 2021 https://www.who.int/publications/i/item/9789240039469 (accessed April 6, 2022).

For peer review only

Supplementary File S10: Preferred Reporting Items for Systematic reviews and Meta-Analyses extension for Scoping Reviews (PRISMA-ScR) Checklist

SECTION	ITEM	PRISMA-ScR CHECKLIST ITEM	REPORTED ON PAGE #
**TITLE**			
Title	1	Identify the report as a scoping review.	1
**ABSTRACT**			
Structured summary	2	Provide a structured summary that includes (as applicable): background, objectives, eligibility criteria, sources of evidence, charting methods, results, and conclusions that relate to the review questions and objectives.	2-3
**INTRODUCTION**			
Rationale	3	Describe the rationale for the review in the context of what is already known. Explain why the review questions/objectives lend themselves to a scoping review approach.	4
Objectives	4	Provide an explicit statement of the questions and objectives being addressed with reference to their key elements (e.g., population or participants, concepts, and context) or other relevant key elements used to conceptualize the review questions and/or objectives.	4
**METHODS**			
Protocol and registration	5	Indicate whether a review protocol exists; state if and where it can be accessed (e.g., a Web address); and if available, provide registration information, including the registration number.	5<br>Supp File S1
Eligibility criteria	6	Specify characteristics of the sources of evidence used as eligibility criteria (e.g., years considered, language, and publication status), and provide a rationale.	5-7<br>Supp File S3
Information sources*	7	Describe all information sources in the search (e.g., databases with dates of coverage and contact with authors to identify additional sources), as well as the date the most recent search was executed.	5<br>Supp File S2
Search	8	Present the full electronic search strategy for at least 1 database, including any limits used, such that it could be repeated.	Supp File S2
Selection of sources of evidence†	9	State the process for selecting sources of evidence (i.e., screening and eligibility) included in the scoping review.	6-7
Data charting process‡	10	Describe the methods of charting data from the included sources of evidence (e.g., calibrated forms or forms that have been tested by the team before their use, and whether data charting was done independently or in duplicate) and any processes for obtaining and confirming data from investigators.	6-7<br>Supp File S4
Data items	11	List and define all variables for which data were sought and any assumptions and simplifications made.	7<br>Supp File S4
Critical appraisal of individual sources of evidence§	12	If done, provide a rationale for conducting a critical appraisal of included sources of evidence; describe the methods used and how this information was used in any data synthesis (if appropriate).	N/A
Synthesis of results	13	Describe the methods of handling and summarizing the data that were charted.	7
**RESULTS**			
Selection of sources of evidence	14	Give numbers of sources of evidence screened, assessed for eligibility, and included in the review, with reasons for exclusions at each stage, ideally using a flow diagram.	8<br>Fig 1
Characteristics of sources of evidence	15	For each source of evidence, present characteristics for which data were charted and provide the citations.	N/A
Critical appraisal within sources of evidence	16	If done, present data on critical appraisal of included sources of evidence (see item 12).	N/A
Results of individual sources of evidence	17	For each included source of evidence, present the relevant data that were charted that relate to the review questions and objectives.	N/A

For peer review only

SECTION	ITEM	PRISMA-ScR CHECKLIST ITEM	REPORTED ON PAGE #
Synthesis of results	18	Summarize and/or present the charting results as they relate to the review questions and objectives.	8-12
**DISCUSSION**			
Summary of evidence	19	Summarize the main results (including an overview of concepts, themes, and types of evidence available), link to the review questions and objectives, and consider the relevance to key groups.	13-4
Limitations	20	Discuss the limitations of the scoping review process.	14-15
Conclusions	21	Provide a general interpretation of the results with respect to the review questions and objectives, as well as potential implications and/or next steps.	15
**FUNDING**			
Funding	22	Describe sources of funding for the included sources of evidence, as well as sources of funding for the scoping review. Describe the role of the funders of the scoping review.	16

From: Tricco AC, Lillie E, Zarin W, O'Brien KK, Colquhoun H, Levac D, et al. PRISMA Extension for Scoping Reviews (PRISMAScR): Checklist and Explanation. Ann Intern Med. 2018;169:467–473. doi: 10.7326/M18-0850.

For peer review only

BMJ Open

Self-care interventions for preconception, antenatal, intrapartum and postpartum care: a scoping review

Journal:	*BMJ Open*
Manuscript ID	bmjopen-2022-068713.R1
Article Type:	Original research
Date Submitted by the Author:	05-Feb-2023
Complete List of Authors:	Nguyen, Phi-Yen; Monash University, Methods in Evidence Synthesis Unit; Burnet Institute, International Development<br>Caddy, Cassandra; Burnet Institute, International Development<br>Wilson, Alyce; Burnet Institute, International Development<br>Blackburn, Kara; Burnet Institute, International Development<br>Page, Matthew; Monash University, School of Public Health and Preventive Medicine<br>Gülmezoglu, Ahmet Metin ; Concept Foundation<br>Narasimhan, Manjulaa; World Health Organization, Department of Reproductive Health and Research<br>Bonet, Mercedes; World Health Organization, Department of Reproductive Health and Research<br>Tunçalp, Özge; World Health Organization, Department of Reproductive Health and Research<br>Vogel, Joshua; Burnet Institute, International Development
**Primary Subject Heading**:	Sexual health
Secondary Subject Heading:	Evidence based practice, Global health, Public health, Reproductive medicine
Keywords:	Antenatal < GENETICS, PUBLIC HEALTH, SEXUAL MEDICINE, REPRODUCTIVE MEDICINE, Maternal medicine < OBSTETRICS, Health policy < HEALTH SERVICES ADMINISTRATION & MANAGEMENT

SCHOLARONE™
Manuscripts

I, the Submitting Author has the right to grant and does grant on behalf of all authors of the Work (as defined in the below author licence), an exclusive licence and/or a non-exclusive licence for contributions from authors who are: i) UK Crown employees; ii) where BMJ has agreed a CC-BY licence shall apply, and/or iii) in accordance with the terms applicable for US Federal Government officers or employees acting as part of their official duties; on a worldwide, perpetual, irrevocable, royalty-free basis to BMJ Publishing Group Ltd ("BMJ") its licensees and where the relevant Journal is co-owned by BMJ to the co-owners of the Journal, to publish the Work in this journal and any other BMJ products and to exploit all rights, as set out in our licence.

The Submitting Author accepts and understands that any supply made under these terms is made by BMJ to the Submitting Author unless you are acting as an employee on behalf of your employer or a postgraduate student of an affiliated institution which is paying any applicable article publishing charge ("APC") for Open Access articles. Where the Submitting Author wishes to make the Work available on an Open Access basis (and intends to pay the relevant APC), the terms of reuse of such Open Access shall be governed by a Creative Commons licence – details of these licences and which Creative Commons licence will apply to this Work are set out in our licence referred to above.

Other than as permitted in any relevant BMJ Author's Self Archiving Policies, I confirm this Work has not been accepted for publication elsewhere, is not being considered for publication elsewhere and does not duplicate material already published. I confirm all authors consent to publication of this Work and authorise the granting of this licence.

For peer review only

Self-care interventions for preconception, antenatal, intrapartum and postpartum care: a scoping review

Phi-Yen Nguyen[1,2], Cassandra Caddy[1], Alyce N. Wilson[1], Kara Blackburn[1], Matthew J. Page[2], A. Metin Gülmezoglu[3], Manjulaa Narasimhan[4], Mercedes Bonet [4], Özge Tunçalp[4], Joshua P. Vogel[1] *

1. Maternal, Child and Adolescent Health Program, Burnet Institute, Melbourne, Australia

2. Methods in Evidence Synthesis Unit, School of Public Health and Preventive Medicine, Monash University, Melbourne, Australia

3. Concept Foundation, Geneva, Switzerland

4. Department of Sexual and Reproductive Health and Research, including UNDP/UNFPA/UNICEF/WHO/World Bank Special Programme of Research, Development and Research Training in Human Reproduction (HRP), World Health Organization, Geneva, Switzerland

***Correspondence to:** A/P Joshua P. Vogel, Global Women's and Newborn's Health Group, Burnet Institute, 85 Commercial Rd, Melbourne, Victoria, Australia 3004.

Email address: joshua.vogel@burnet.edu.au

Total word count: 3,751 words (excluding tables and figures)

Keywords: self-care, pregnancy, preconception, antenatal, intrapartum, postpartum, postnatal, maternal outcomes, neonatal outcomes, infants, newborns

For peer review only - http://bmjopen.bmj.com/site/about/guidelines.xhtml

ABSTRACT (300 words)

Objective: To identify current and emerging self-care interventions aimed at improving maternity health care

Design: Scoping review

Data sources: Medline, Embase, Emcare, PsycINFO, Cochrane CENTRAL/CDSR, CINAHL Plus

Eligibility criteria: Evidence syntheses, interventional or observational studies describing any tool, resource or strategy to facilitate self-care in women preparing to get pregnant, currently pregnant, giving birth, or postpartum.

Data extraction/synthesis: Screening and data collection were conducted independently by two reviewers. Self-care interventions were identified based on pre-defined criteria and inductively organised into 11 categories. Characteristics of study design, interventions, participants and outcomes were recorded.

Results: We identified eligible 580 studies. Many included studies evaluated interventions in high-income countries (45%) and during antenatal care (76%). Self-care categories featuring highest numbers of studies were diet and nutrition (26% of all studies), physical activity (24%), psycho-social strategies (18%) and other lifestyle adjustments (17%). Few studies featured self-care interventions for sexual health and postpartum family planning (2%), self-management of medication (3%), and self-testing/sampling (3%). Several venues to introduce self-care were described: at health facilities (44%), community venues (14%), digital platforms (18%), via partner/peer support (7%), or with over-the-counter products (13%). Involvement of health and community workers were described in 38% and 8% of studies, who supported self-care interventions by providing therapeutics for home use, training or counselling. The most common categories of outcomes evaluated were neonatal outcomes (e.g. birth weight) (31%), maternal mental health (26%), and labour outcomes (e.g. duration of labour) (22%).

For peer review only - http://bmjopen.bmj.com/site/about/guidelines.xhtml

Conclusion: Self-care interventions in maternal care are diverse in their applications, implementation characteristics and intended outcomes. Many self-care interventions were implemented with support from the health system at initial stages of use and uptake. Some promising self-care interventions require further primary research, though several are more matured and up-to-date evidence syntheses are needed. Research on self-care in the pre-conception period is lacking.

Strengths

- Mapping all potential self-care practices across the spectrum of pregnancy care, from preconception, antenatal, intrapartum to postnatal care
- Use of the new WHO self-care classification system to organise and categorise findings

Limitations

- Not screening for primary studies reported within an included evidence synthesis
- Not including interventions that took place after 42 days postpartum, or those that related primarily to care of newborns and breastfeeding (which should require dedicated reviews)
- Search limited to published reports and reports written in English only

For peer review only

For peer review only - http://bmjopen.bmj.com/site/about/guidelines.xhtml

BACKGROUND

Self-care is defined by the World Health Organization (WHO) as “the ability of individuals, families and communities to promote health, prevent disease, maintain health, and to cope with illness and disability with or without the support of a health worker” [1]. Self-care may include, but is not limited to, health literacy, self-awareness of physical and mental health, physical activity, healthy eating, good hygiene, and rational use of medicines and diagnostics [2]. By extension, self-care interventions are those that can support or promote people’s self-care [3]. Self-care interventions can act to complement or even broaden delivery of health services, ideally improving health outcomes and care experiences [4,5]. Humanitarian crises, emergencies and pandemics introduce additional challenges to the provision of routine healthcare services [6,7]. In this context, self-care interventions can help ensure health and well-being is maintained with reduced in-person contact [8].

In 2021, WHO published a guideline on self-care interventions for health and wellbeing, which included 13 recommendations on selected self-care interventions to improve preconception, antenatal, intrapartum and postpartum care [3]. By encouraging women to engage in these self-care interventions, the overall efficiency of healthcare delivery can be enhanced. In addition, there are interventions for pregnant and postpartum women that are not currently included in WHO’s self-care guideline, but have the potential of transitioning into a self-care paradigm. Examples include prescription medicines that can be made available over-the-counter, self-collection of samples for testing, or home testing kits for certain diseases. Some systematic reviews have been conducted on self-care interventions in pregnancy [9–11]; however these previous reviews focused on a few selected interventions and outcomes, and mainly the antenatal or postpartum periods. No previous review has explored the broad and heterogeneous body of research across preconception, antenatal, intrapartum and postpartum periods, in order to identify those that may be effective or applicable from a self-care perspective. We therefore aimed to identify all current and emerging self-care interventions via a scoping review that have been evaluated in the context of preconception, antenatal, intrapartum and postpartum care.

For peer review only - http://bmjopen.bmj.com/site/about/guidelines.xhtml

METHODS

The scoping review was conducted in accordance with the methodological guide for scoping reviews by Peters and colleagues [12], and reported in accordance with the PRISMA guideline for Scoping Reviews [13] (Supplementary File S1). The protocol was registered online at https://doi.org/10.17605/OSF.IO/97X25 (Supplementary File S2).

Electronic searches

The search strategy was developed in consultation with an information specialist (Supplementary File S3) and combined self-care concepts with terms related to preconception, antenatal, intrapartum and postpartum care, based on the framework proposed by Narasimhan and colleagues [4]. The terms and concepts used were informed by previous reviews of pregnancy care and self-care for other health conditions [14–16] and by inputs from team members with clinical and public health background. The following databases were systematically searched to identify published studies from inception to October 17th, 2021: Medline, Embase, Emcare and PsycINFO (via Ovid), CENTRAL/Cochrane Database of Systematic Reviews (via Cochrane Library) and CINAHL Plus (via EBSCOhost). The last date of search was October 17th, 2021. Reference lists of included studies were manually screened to identify other relevant studies. Grey literature was not searched after considering the volume and variable quality of such evidence [17].

Study selection

Two reviewers (PN and CC) first independently screened all titles and abstracts to exclude irrelevant studies, and then independently reviewed potentially eligible full texts based on pre-determined

For peer review only - http://bmjopen.bmj.com/site/about/guidelines.xhtml

eligibility criteria. All stages of screening were conducted using Covidence software [18]. Any disagreement was resolved by discussion or adjudication by a third reviewer (JPV).

The eligibility criteria for inclusion were: (A) Participants: women preparing to get pregnant, currently pregnant, giving birth, or in the postpartum period (up to 42 days after birth), of any age. Studies in which partners or family members carried out self-care for the pregnant woman, were also eligible. Interventions for abortion were not eligible. We also excluded interventions that were only directed at newborns and postnatal breastfeeding interventions for improving neonatal outcomes, though antenatal programmes related to preparation for the postnatal period were eligible. (B) Interventions: For this review, a self-care intervention was defined as any tool, resource or strategy designed to promote or facilitate self-care by a woman intending to get pregnant, or a currently pregnant, labouring or in the postpartum period, for the purpose of improving the quality or coverage of maternity health care, and/or improving her health, well-being and care experiences. To be eligible, interventions must have been aimed at reducing risks of pregnancy, childbirth or postpartum complications and/or enabling a positive pregnancy, childbirth or postpartum experience. Each intervention was evaluated independently by two reviewers for inclusion based on a set of pre-specified principles (Supplementary File S4). (C) Comparator: placebo, no treatment or standard care; single-arm interventional studies were also eligible. (D) Outcomes: outcomes related to maternal physical, psychological or emotional health, behavioural change affecting a mother's own health, or diagnostic accuracy of tests for women. Studies reporting economic outcomes only were not included, and neither were feasibility studies evaluating only retention rates and acceptability. (E) Types of studies: randomized trials, quasi-experimental studies and cohort studies, scoping reviews, systematic reviews, meta-analyses and overviews of systematic reviews. Systematic reviews and overviews must have stated some form of search strategy and presented all included studies in a systematic manner to be eligible for inclusion. Ongoing studies (defined as trials with protocols published from 2019 onwards which were not completed) were eligible; no restriction on date of publication was applied otherwise. Case series, case studies, case reports, dissertations and theses, reports published as

For peer review only - http://bmjopen.bmj.com/site/about/guidelines.xhtml

abstracts only, correspondence letters, editorials, and qualitative studies were excluded. Only studies with full texts written in English were eligible.

Data collection and analysis

Two reviewers independently extracted data using a standardised extraction form we developed to suit the review (Supplementary File S5). Data extracted included characteristics of each study's population, interventions, and outcomes measured. Study designs were classified as evidence syntheses, interventional studies, observational studies or diagnostic accuracy studies. Countries where studies took place were classified based on the World Bank 2021 country income levels [19]. For population characteristics, we recorded whether the studies included women under the age of 18, and whether those with high-risk pregnancies were included. For each study, the self-care activities that the intervention aimed to promote or facilitate, and the period of pregnancy during which the intervention took place (preconception, antenatal, intrapartum or postpartum period) were recorded. An inductive thematic analysis process was undertaken [20]. After all self-care interventions were identified, we analysed their descriptions and identified themes among groups of interventions. For example, interventions involving taking dietary supplements or adjusting diet intakes were grouped under the theme "Diet and nutrition". These themes were refined until all interventions were accounted for, creating the final set of self-care categories. In addition, we recorded the venues of access to self-care, i.e. the setting or location where participants were orientated to or carried out self-care, based on WHO's framework [21]. We also recorded whether any external support person was required to promote or facilitate self-care. These information were recorded for primary studies only, as evidence synthesis studies may include studies with different characteristics. Any disagreement was resolved via consensus or adjudication by a third reviewer (JPV). In line with scoping review methodology [12], quality assessment of included studies was not performed. Descriptive statistics (frequency and percentage) were used to report study, participant and intervention characteristics. Studies that feature more than three self-care interventions were analysed and reported separately. R

For peer review only - http://bmjopen.bmj.com/site/about/guidelines.xhtml

v4.1.2 was used to calculate the frequencies of various intervention characteristics, participant characteristics and outcomes for each self-care intervention, aggregate these frequencies by self-care categories and present them as bar charts.

Patient and Public Involvement Statement: Patients were not included in the design, conduct and plans for dissemination of results of this review.

RESULTS

A total of 12,215 records from six databases were retrieved, together with an additional 182 records from manual reference screening (Fig. 1). In total, 10,712 reports were excluded at the title/abstract screening stage and another 743 reports at full text screening (Supplementary File S6). The final sample consisted of 580 unique studies (616 reports) (Supplementary File S7), 18 (3%) of which were ongoing studies.

Fig. 1. PRISMA flowchart of record retrieval and selection

Characteristics of included studies

Among 580 studies, two-thirds were interventional studies (n=365, 63%), many of which reported results of randomised trials (n=306, 53%). Evidence syntheses (n=165) accounted for 28% of all studies, out of which 109 were meta-analyses (19%). Observational studies were less common (n=32, 6%) and were mainly retrospective or prospective cohort studies (n=25, 4%). Study designs used were largely similar across all self-care categories, with some exceptions (Fig. 2A, Supplementary File S8-A). In self-management of medication, the proportion of non-randomised studies (n=7, 41%) was similar to that of randomised trials (n=6, 35%). In self-testing and self-collection of samples, many studies (n=10, 56%) were diagnostic accuracy studies.

For peer review only - http://bmjopen.bmj.com/site/about/guidelines.xhtml

For peer review only

Most studies took place in high-income countries (n=260, 45%), followed by middle-income (n=146, 25%) and low-income countries (n=11, 2%); 163 studies (28%) were reviews with no geographic restriction, and usually included studies from multiple countries. Countries with the most studies were the United States of America (USA) (n=86, 15%), Iran (n=46, 8%), United Kingdom (UK) (n=33, 6%) and Australia (n=30, 5%). Self-care interventions most commonly took place in the antenatal (n=439, 76%) and postpartum periods (n=119, 21%). Few studies related to the preconception (n=21, 4%) or intrapartum periods (n=62, 11%) (Fig. 2B, Supplementary File S8-B).

Fig. 2. Characteristics of included studies

Thirty-eight studies (7%) included women under the age of 18. A third of included studies (n=170, 29%) included women with high-risk pregnancies. High-risk pregnancies were variably defined – the most common definitions included pregnant women with gestational or existing diabetes (n=54, 9%), obesity or overweight (n=35, 6%) and ex-/current smokers (n=31, 5%).

Self-care interventions and their characteristics

Among included studies, 35 studies (6%) described a complex intervention involving more than three different self-care activities (Supplementary File S9). The remaining 545 studies (94%), which featured up to three self-care activities, constituted the final sample for identification of self-care interventions. A total of 112 unique self-care interventions were identified and organised into 11 broad categories (Fig. 3; Supplementary File S10). The most common self-care categories identified included diet and nutrition (n=143/545, 26%), physical activity (n=132, 24%), psycho-social strategies (n=97, 18%) and lifestyle adjustments (n=90, 17%). Few studies featured self-care interventions under the categories of sexual health and family planning (2%), self-management of medication (3%), and self-testing/sampling (3%). All identified interventions demonstrate various degrees of interaction with the health system [4], from little to no direct contact (self-

For peer review only

For peer review only - http://bmjopen.bmj.com/site/about/guidelines.xhtml

awareness/regulation), some interaction (self-management of health conditions) and close linkage with the health system for follow-up (self-testing & diagnosis) (Fig. 3).

Fig. 3. All self-care interventions identified from included studies and their interactions with the health system

The identified interventions correspond to a range of categories from WHO's classification of self-care interventions (Supplementary File S11). The most common WHO categories were interventions for improving capacity and promoting autonomy in self-care (n=325, 56%), self-care prevention including risk avoidance and support for physical and mental health and well-being (n=296, 51%) and self-care for short-term health conditions (n=189, 33%). Some WHO categories did not feature amongst included studies, such as on-demand information services for health information, diagnostic devices in community locations, online symptom checkers for diagnosis, and interventions enabling individuals to identify location of health facilities/structures. Notably, we identified no studies that targeted self-care outside the context of a specific health issue, such as campaigns to promote awareness about self-care or programmes to improve health and digital literacy.

Among 545 studies under analysis, self-care was accessible through several avenues as described in the WHO classification framework [20]. These avenues include health facilities (n=238/545, 44%), digital platforms (n=97, 18%), community and non-facility venues (n=74, 14%), partner or peer support (n=37, 7%) or over-the-counter (OTC) products e.g. nutritional supplements, topical products, or support devices (n=69, 13%). In 80 studies (15%), there was insufficient detail to determine that the aforementioned modes of access were used. No studies were identified where the intervention took place at the workplace, in schools, or in emergency or humanitarian settings.

Across the self-care categories, provision of information and demonstration of self-care were most commonly done in health facilities, especially for self-testing (94% of studies in this category) and self-monitoring (58%). Provision of similar services outside health facilities, such as via community health workers or at community-based venues, was mainly for the support of self-management of medication (41%) and physical activity (28%). Home-based self-care (i.e. direct access to self-care

For peer review only - http://bmjopen.bmj.com/site/about/guidelines.xhtml

resources and information at home) was less frequent, mainly in the categories of breast or nipple care (23%) and lifestyle adjustments (20%). Digital platforms were popular in some categories, notably self-monitoring (51%). Use of OTC products was common in complementary/alternative therapies (39%) and breast and nipple care (27%). Lastly, peer or partner support was relatively less common across categories (7%), and we observed that partner support (1%) was relatively less frequent than support from peers (e.g. other women, members of support groups) (6%) (Fig. 4A, Supplementary File S8-C).

A large number of studies described the involvement of health workers in promoting or facilitating self-care (n=208/545, 38%), especially for sexual health & family planning (67% of studies in this category), psychosocial strategies (45%) or physical activity (44%) (Fig. 4B, Supplementary File S8-D). Few studies mentioned involvement of trained community health workers (n=41, 8%), non-trained community members (n=25, 5%) or family members (n=3, 1%).

Fig. 4. Characteristics of self-care interventions

Types of outcomes in self-care studies

Included studies used a diverse range of outcomes, including both maternal and neonatal outcomes (Table 1)., the most commonly measured ones were neonatal outcomes (birth weight, Apgar scores, preterm birth, admission to neonatal intensive care unit and other neonatal complications) (n=177, 31%); maternal mental wellbeing and quality of life (n=150, 26%), and labour outcomes (duration of labour stages, labour pain, mode of delivery or labour induction methods) (n=125, 22%). A total of 147 studies (25%) measured behavioural outcomes, mainly related to reductions in risky behaviours such as smoking or alcohol consumption. Maternal mortality (n=20, 3%) and perinatal mortality (n=67, 12%) were less commonly evaluated.

For peer review only - http://bmjopen.bmj.com/site/about/guidelines.xhtml

Table 1. Evidence map for outcomes measured

Categories / Outcome	Maternal mortality	Perinatal mortality	Neonatal outcomes	Labour outcomes	Anthropometric & cardiometabolic outcomes	Diabetes & other metabolic disorders	Hypertensive disorders of pregnancy	Postpartum infection & haemorrhage	Urogenital outcomes	Nutritional deficiencies	Gastrointestinal outcomes	Other maternal complications	Infectious diseases	Other minor physiological discomforts	Mental wellbeing & quality of life	Behavioural change	Breastfeeding	Other
Diet & nutrition	5	29	71	27	53	47	34	4	9	11	12	14	3	3	21	42	7	0
Physical activity	0	6	48	33	58	45	22	7	5	1	1	9	0	13	40	50	7	0
Lifestyle adjustments	0	9	16	1	0	1	4	0	1	0	1	5	12	9	11	49	1	0
Complementary & alternative therapies	0	3	15	26	1	1	2	2	14	0	6	3	0	10	23	1	8	0
Psycho-social strategies	0	3	16	23	1	6	5	1	4	0	1	4	0	9	75	8	7	2
Breast & nipple care	0	3	6	4	0	2	1	1	0	0	0	0	0	16	1	2	17	1
Sexual health & family planning	0	0	1	0	0	0	0	0	0	0	0	1	1	0	7	9	0	0
Self-monitoring	3	18	39	28	23	27	24	3	0	0	0	8	0	0	15	17	1	1
Self-management of medication	6	4	7	0	1	0	1	5	0	0	0	4	4	0	1	12	0	0
Self-testing or sampling	1	2	4	1	1	0	1	0	0	0	0	0	1	0	0	5	0	1
Other self-management skills	5	5	16	27	0	1	5	17	3	0	0	10	0	1	17	4	1	3
**All self-care categories**	**20**<br>**3%**	**67**<br>**12%**	**177**<br>**31%**	**125**<br>**22%**	**78**<br>**13%**	**79**<br>**14%**	**65**<br>**11%**	**33**<br>**6%**	**32**<br>**6%**	**11**<br>**2%**	**17**<br>**3%**	**43**<br>**7%**	**20**<br>**3%**	**46**<br>**8%**	**150**<br>**26%**	**147**<br>**25%**	**34**<br>**6%**	**8**<br>**1%**

Note: Number in each cell denotes the number of studies featuring the corresponding self-care category (row) and measuring the corresponding outcome (column). Percentages are based on total studies (N=580). For further details on the definition of each outcome category, see Supplementary File S5.

For peer review only - http://bmjopen.bmj.com/site/about/guidelines.xhtml

DISCUSSION

Main findings

In this scoping review, 580 studies were identified pertaining to 112 self-care interventions across preconception, antenatal, intrapartum and postpartum periods. These interventions encompassed multiple modalities, settings and levels of provider involvement. Most self-care interventions required close engagement with health facilities, in which healthcare workers or community health workers would teach self-care skills or provide therapeutics for home use. Previous studies also suggest that healthcare services are a critical starting point for successful self-care approaches [22–24]. As such, self-care should be viewed as an extension of, and not a separate entity to, formal healthcare services. Our findings also reflect a broader trend towards task-sharing in order to increase maternity care coverage [7,25]. While the majority of self-care interventions were implemented in the antenatal period, many of these studies aimed to equip pregnant individuals with self-care skill via workshops or counselling, which can be used in the intrapartum (e.g. relaxation methods during labour) or postpartum periods (e.g. pelvic floor exercises, breast and nipple care). Few studies examined the potential benefits of self-care at the pre-conception period. Few interventions featured digital platforms (e.g. websites, mobile apps); those identified were mostly used for communication between the pregnant individual and a remote health worker, or as a platform for the pregnant individual to record her medical information and receive automatic, individualised health advice. While digital technologies seem to currently play a limited role, they have great potential to catalyse and promote maternity-related self-care [26].

Most studies concentrated on self-care interventions related to lifestyle modification - such as diet, nutrition/supplement intake, physical activity and sleep habits - as well as strategies to optimise mental and social well-being. The established evidence base for lifestyle interventions was also reflected in previous systematic reviews on self-care for preeclampsia and gestational diabetes [9,11].

For peer review only - http://bmjopen.bmj.com/site/about/guidelines.xhtml

This might be attributable to a perceived lower risk of harm when conducting trials investigating lifestyle interventions, compared to clinical or pharmacological interventions. Moreover, lifestyle interventions tend to take place in their own living environments and in their own time, and hence may be more amenable to self-care approaches. Conversely, there were relatively few studies that explored self-monitoring, self-testing or self-management of medications or pregnancy complications. While studies of these self-care approaches can be complex or technically challenging, they could provide solutions to improving care in remote and low-resource settings [6]. While advanced distribution and self-administration of misoprostol for postpartum haemorrhage prevention has been well-explored [27–29], there was limited or no research on similar approaches to other therapeutics, such as use of anthelminthics, malaria prophylaxis, antimalarials, antiretroviral therapy, or vitamin and micronutrient supplementation. Although many studies reported on the prevalence and determinants of self-medication during pregnancy [30–32], none investigated interventions aimed at promoting safe self-medication practices, or how to seek information when self-medicating. There were also few self-care interventions aimed at communicable disease detection and prevention during or after pregnancy. Future research is warranted to investigate how these aspects of clinical care during and after pregnancy could be approached from a self-care perspective.

We identified studies describing self-testing options for urinary tract infections and HIV, but few or no studies on malaria, syphilis or chlamydia self-testing, for which rapid test technologies are available but not yet scaled up or tested in pregnancy [33–35]. Similarly, self-collection of vaginal samples for reproductive tract infections is an emerging alternative to clinic-based sampling [36]. While some studies have investigated diagnostic accuracy of self-sampling for reproductive tract infections in pregnancy, evidence on their benefits, harms, and effective linkages with health systems is currently lacking [37]. Lastly, we identified self-care studies on insecticide-treated nets for malaria, mostly from the sub-Saharan Africa region. However there was little research on other low-cost strategies amenable to a self-care approach (indoor residual spraying and insect repellents), or from other malaria-endemic regions such as Asia and Latin America [38].

For peer review only - http://bmjopen.bmj.com/site/about/guidelines.xhtml

Last but not least, we found no eligible studies on traditional and indigenous self-care practices. While important qualitative research has been conducted on this topic [39,40] , we hypothesise that there is a relative lack of studies exploring the health effects of these types of self-care practices. Future research on these topics could also provide new opportunities for improving cultural safety of maternity care services.

Strengths and limitations

To our knowledge, this is the first scoping review that maps all potential self-care practices across the spectrum of pregnancy care, from preconception, antenatal, intrapartum to postnatal care. We analysed studies based on multiple characteristics, as well as applying the new WHO self-care classification system to organise, categorise and facilitate interpretation of findings.

This scoping review was intended to present an overview of the research landscape, and not to critically appraise or meta-analyse available evidence. The total number of studies identified for a given self-care intervention may not correlate with evidence quality, or its effectiveness. We reported the results for complex interventions (i.e. interventions with more than three different self-care practices) separately (Supplementary File S9), since segregating each complex intervention into multiple individual interventions would overestimate their representation in the sample and disregard potential synergy between the self-care components. Besides, because these studies did not introduce any new self-care intervention, or a substantive number of studies for any single self-care intervention that we had already identified, it is unlikely that including them into the main analysis would change our results and conclusion. Although the number of such studies is small (n=35, 6%), they represent how self-care can be implemented in real-life, and future reviews should study their effects using appropriate methods e.g. component network meta-analysis [41].

Some limitations must nonetheless be acknowledged. We did not formally screen those primary studies reported within an included evidence synthesis, and we assessed intervention and outcomes characteristics based on the review's summary description of the included studies. It is possible that

For peer review only - http://bmjopen.bmj.com/site/about/guidelines.xhtml

the number of primary studies was there underestimated, or the characteristics of individual primary studies might differ. Our assessment found 45 primary studies (8% of all included reports) that were also included within an evidence synthesis included in this review. Given their small number, we consider it unlikely that these overlapping studies would substantively affect the overall results. Hence, the distribution of study designs (Fig. 2A, Supplementary File S8-A) should be considered in addition to the volume of evidence available for each intervention. We opted to focus on maternity-related self-care, thus excluding interventions that took place after 42 days postpartum, or those that related primarily to care of newborns and breastfeeding. Our research objectives also mean that we did not include qualitative studies. These exclusions do not discount their importance, but rather acknowledge that these topics require separate, dedicated reviews. Our search of published reports means that self-care interventions that have only been evaluated in unpublished studies or grey literature would not have been identified. With 45 reports excluded as not written in English (3.3% of all full-text reports), language bias is possible.

Future research

This review was conducted for the purpose of guiding further evidence synthesis activities and assisting the WHO with guideline development in this research area. This scoping review identified two distinct groups of self-care interventions, which have different pathways forward. The first group are those interventions with a substantial number of primary studies, where no systematic review has been conducted or only outdated systematic reviews exist. For these interventions – such as use of clean cookstoves to reduce adverse birth outcomes associated with indoor air pollution, self-care interventions to reduce postpartum depression, or self-collection of vaginal samples to test for sexually transmitted diseases - up-to-date systematic reviews are warranted to inform guidelines, clinical practice and self-care programs. The second group are interventions with limited or no primary studies, or where available primary studies were not designed to fully deliver the intervention in a self-care approach. For these interventions, well-designed primary studies that compare a self-

For peer review only - http://bmjopen.bmj.com/site/about/guidelines.xhtml

care approach with standard care are needed. Examples include self-testing for proteinuria, or self-measurement of vaginal pH to detect early signs of bacterial vaginosis. Some self-care interventions may be particularly useful in limited-resource settings, and thus should be evaluated in such settings.

CONCLUSION

Self-care interventions during pre-conception, antepartum, intrapartum and postpartum periods are diverse in scope and implementation characteristics. Many self-care interventions were implemented with support from the health system at initial stages of use and uptake. Some promising self-care interventions require further primary research, though several are more matured and up-to-date evidence syntheses are needed. Research on self-care in the pre-conception period is lacking. Future research should explore task-sharing from clinical care to home or community settings, or effective self-management of complications where access to care is unavailable.

List of abbreviations

HIV: Human Immunodeficiency Virus

OTC: over-the-counter

WHO: World Health Organization

DECLARATIONS

Ethics approval and consent to participate: Not applicable. This study does not involve human participants and ethical approval was not required.

Consent for publication: Not applicable. This study does not collect personal information from an identifiable individual and informed consent was not required.

For peer review only - http://bmjopen.bmj.com/site/about/guidelines.xhtml

Availability of data and materials: Data supporting the findings and conclusion of this study are included in this published article and its supplementary files. The underlying dataset and analytic code are available at osf.io/26f7y.

Competing interests: None declared.

Funding: This project was funded by UNDP/UNFPA/UNICEF/WHO/World Bank Special Programme of Research, Development and Research Training in Human Reproduction (HRP), Department of Sexual and Reproductive Health and Research, World Health Organization (# PO 202724071). While members of our authorship team (MN, MB, OT) are employed by HRP/WHO, the funder organization had no role in the design and conduct of the study; collection, management, analysis, and interpretation of the data; preparation, review, or approval of the manuscript; and decision to submit the manuscript for publication.

Acknowledgement: We thank Steve McDonald (Cochrane Australia) for his guidance in formulating the search strategy.

Author contributions

The corresponding author attests that all listed authors meet authorship criteria and that no others meeting the criteria have been omitted. P-YN and JPV conceptualised the study, wrote the protocol and statistical plan and organised collaborative meetings. P-YN and CC screened the literature and extracted the data. P-YN analysed the data, wrote the original draft and revised the paper. MJP advised on the design of the study. CC, AW, KB, MJP, AMG, MN, MB, OT, JPV participated in technical and clinical discussions, and reviewed and revised the paper.

Supplementary Files

Supplementary File S1: PRISMA-ScR checklist

Supplementary File S2: Protocol as registered on Open Science Framework

For peer review only - http://bmjopen.bmj.com/site/about/guidelines.xhtml

Supplementary File S3: Search strategy for all databases

Supplementary File S4: Defining self-care interventions, a conceptual framework and examples for making judgements on specific interventions

Supplementary File S5: Data collection form

Supplementary File S6: List of excluded full texts

Supplementary File S7: Reference list of included studies

Supplementary File S8: Complex interventions and their characteristics

Supplementary File S9: List of all self-care interventions identified from included studies

Supplementary File S10: Categorising self-care interventions based on WHO's classification framework

For peer review only

For peer review only - http://bmjopen.bmj.com/site/about/guidelines.xhtml

References

1 World Health Organization. Self-care interventions for health. Fact sheet. 2021.https://www.who.int/news-room/fact-sheets/detail/self-care-health-interventions (accessed 29 May 2021).

2 International Self-Care Foundation. The Seven Pillars of Self-Care. https://isfglobal.org/practise-self-care/the-seven-pillars-of-self-care/ (accessed 13 Jan 2022).

3 World Health Organization. Guideline on Self-Care Interventions for Health and Well-Being. Geneva: 2021. https://www.who.int/publications/i/item/9789240030909

4 Narasimhan M, Allotey P, Hardon A. Self care interventions to advance health and wellbeing: A conceptual framework to inform normative guidance. *BMJ* 2019;**365**. doi:10.1136/bmj.l688

5 Narasimhan M, Logie CH, Gauntley A, *et al.* Self-care interventions for sexual and reproductive health and rights for advancing universal health coverage. *Sex Reprod Heal matters* 2020;**28**. doi:10.1080/26410397.2020.1778610

6 Pavlidis P, Eddy K, Phung L, *et al.* Clinical guidelines for caring for women with COVID-19 during pregnancy, childbirth and the immediate postpartum period. Women and Birth. 2020. doi:10.1016/j.wombi.2020.10.015

7 Gülmezoglu AM, Ammerdorffer A, Narasimhan M, *et al.* Self-care and remote care during pregnancy: A new paradigm? *Heal Res Policy Syst* 2020;**18**:1–3. doi:10.1186/S12961-020-00627-4

8 World Health Organization. Maintaining essential health services: operational guidance for the COVID-19 context, interim guidance. 2020.https://www.who.int/publications/i/item/WHO-2019-nCoV-essential_health_services-2020.2 (accessed 21 Jan 2022).

9 Rasouli M, Pourheidari M, Gardesh Z. Effect of Self-care Before and During Pregnancy to Prevention and Control Preeclampsia in High-risk Women. *Int J Prev Med* 2019;**10**. doi:10.4103/IJPVM.IJPVM_300_17

10 Dalrymple KV, Flynn AC, Poston L, *et al.* Preventing postpartum weight retention: Lifestyle interventions in pregnancy, postpartum or both. A systematic review of the literature. *Proc Nutr Soc* 2018;**77**:77. doi:10.1017/S002966511800191X

11 Kolivand M, Rahimi MA, Shariati M, *et al.* The Effect of Self-care Educational/Training Interventions on the Outcomes of Gestational Diabetes: A Review Article. *Iran J Public Health* 2018;**47**:1805.https://www.ncbi.nlm.nih.gov/pmc/articles/PMC6379603/ (accessed 27 Apr 2022).

12 Peters MDJ, Godfrey CM, Khalil H, *et al.* Guidance for conducting systematic scoping reviews. *Int J Evid Based Healthc* 2015;**13**:141–6. doi:10.1097/XEB.0000000000000050

13 Tricco AC, Lillie E, Zarin W, *et al.* PRISMA extension for scoping reviews (PRISMA-ScR): Checklist and explanation. Ann. Intern. Med. 2018;**169**:467–73. doi:10.7326/M18-0850

14 Abalos E, Chamillard M, Diaz V, *et al.* Antenatal care for healthy pregnant women: a mapping of interventions from existing guidelines to inform the development of new WHO guidance on antenatal care. *BJOG* 2016;**123**:519–28. doi:10.1111/1471-0528.13820

15 Donald M, Kahlon BK, Beanlands H, *et al.* Self-management interventions for adults with chronic kidney disease: a scoping review. *BMJ Open* 2018;**8**:e019814.

For peer review only - http://bmjopen.bmj.com/site/about/guidelines.xhtml

doi:10.1136/BMJOPEN-2017-019814

16 Foster G, Taylor SJC, Eldridge SE, *et al.* Self-management education programmes by lay leaders for people with chronic conditions. *Cochrane Database Syst Rev* 2007.

17 Benzies KM, Premji S, Hayden KA, *et al.* State-of-the-Evidence Reviews: Advantages and Challenges of Including Grey Literature. *Worldviews Evidence-Based Nurs* 2006;**3**:55–61. doi:10.1111/J.1741-6787.2006.00051.X

18 Veritas Health Innovation. Covidence Systematic Review Software, Melbourne Australia. 2020.www.covidence.org (accessed 22 Jul 2021).

19 World Bank. The World by Income and Region. 2021.https://datatopics.worldbank.org/world-development-indicators/the-world-by-income-and-region.html (accessed 20 Jan 2022).

20 Braun V, Clarke V. *Thematic analysis.* 2012. doi:10.1037/13620-004

21 World Health Organization. Classification of self-care interventions for health: a shared language to describe the uses of self-care interventions. Geneva: 2021. https://www.who.int/publications/i/item/9789240039469 (accessed 6 Apr 2022).

22 Bell J, Dziekan G, Pollack C, *et al.* Self-Care in the Twenty First Century: A Vital Role for the Pharmacist. *Adv Ther* 2016;**33**:1691–703. doi:10.1007/S12325-016-0395-5

23 Davies NJ. Improving self-management for patients with long-term conditions. *Nurs Stand* 2010;**24**. doi:10.7748/NS2010.02.24.25.49.C7562

24 Lorig KR, Holman HR. Self-Management Education: History, Definition, Outcomes, and Mechanisms. *Ann Behav Med* 2003;**26**:1–7.https://academic.oup.com/abm/article/26/1/1/4630312 (accessed 13 Jan 2022).

25 Dawson AJ, Buchan J, Duffield C, *et al.* Task shifting and sharing in maternal and reproductive health in low-income countries: a narrative synthesis of current evidence. *Health Policy Plan* 2014;**29**:396–408. doi:10.1093/HEAPOL/CZT026

26 DeNicola N, Grossman D, Marko K, *et al.* Telehealth Interventions to Improve Obstetric and Gynecologic Health Outcomes: A Systematic Review. *Obstet Gynecol* 2020;**135**:371–82. doi:10.1097/AOG.0000000000003646

27 Oladapo OT, Blum J, Abalos E, *et al.* Advance misoprostol distribution to pregnant women for preventing and treating postpartum haemorrhage. *Cochrane Database Syst Rev* 2020;**2020**. doi:10.1002/14651858.CD009336.PUB3

28 Ononge S, Campbell OMR, Kaharuza F, *et al.* Effectiveness and safety of misoprostol distributed to antenatal women to prevent postpartum haemorrhage after child-births: A stepped-wedge cluster-randomized trial. *BMC Pregnancy Childbirth* 2015;**15**. doi:10.1186/S12884-015-0750-6

29 Weeks AD, Ditai J, Ononge S, *et al.* The MamaMiso study of self-administered misoprostol to prevent bleeding after childbirth in rural Uganda: A community-based, placebo-controlled randomised trial. *BMC Pregnancy Childbirth* 2015;**15**:1–10. doi:10.1186/S12884-015-0650-9

30 Atmadani RN, Nkoka O, Yunita SL, *et al.* Self-medication and knowledge among pregnant women attending primary healthcare services in Malang, Indonesia: A cross-sectional study. *BMC Pregnancy Childbirth* 2020;**20**:1–11. doi:10.1186/S12884-020-2736-2

31 Niriayo YL, Mohammed K, Asgedom SW, *et al.* Self-medication practice and contributing

For peer review only

For peer review only - http://bmjopen.bmj.com/site/about/guidelines.xhtml

factors among pregnant women. *PLoS One* 2021;**16**:e0251725. doi:10.1371/JOURNAL.PONE.0251725

32 Mohseni M, Azami-Aghdash S, Sheyklo SG, *et al.* Prevalence and reasons of self-medication in pregnant women: A systematic review and meta-analysis. Int. J. Community Based Nurs. Midwifery. 2018;**6**.

33 Berthod D, Rochat J, Voumard R, *et al.* Self-diagnosis of malaria by travellers: A cohort study on the use of malaria rapid diagnostic tests provided by a Swiss travel clinic. *Malar J* 2017;**16**:1–8. doi:10.1186/S12936-017-2079-2

34 Wang C, Cheng W, Li C, *et al.* Syphilis Self-testing: A Nationwide Pragmatic Study Among Men Who Have Sex With Men in China. *Clin Infect Dis* 2020;**70**:2178–86. doi:10.1093/CID/CIZ603

35 Sacks-Davis R, Gold J, Aitken CK, *et al.* Home-based chlamydia testing of young people attending a music festival - Who will pee and post? *BMC Public Health* 2010;**10**:1–9. doi:10.1186/1471-2458-10-376

36 Fajardo-Bernal L, Aponte-Gonzalez J, Vigil P, *et al.* Home-based versus clinic-based specimen collection in the management of Chlamydia trachomatis and Neisseria gonorrhoeae infections. *Cochrane Database Syst Rev* 2015;**2015**. doi:10.1002/14651858.CD011317.PUB2

37 Siegfried N, Narasimhan M, Logie CH, *et al.* Prioritising gender, equity, and human rights in a GRADE-based framework to inform future research on self care for sexual and reproductive health and rights. *BMJ Glob Heal* 2020;**5**:2128. doi:10.1136/BMJGH-2019-002128

38 Menéndez C, D'Alessandro U, ter Kuile FO. Reducing the burden of malaria in pregnancy by preventive strategies. *Lancet Infect Dis* 2007;**7**:126–35. doi:10.1016/S1473-3099(07)70024-5

39 Raman S, Nicholls R, Ritchie J, *et al.* Eating soup with nails of pig: thematic synthesis of the qualitative literature on cultural practices and beliefs influencing perinatal nutrition in low and middle income countries. *BMC Pregnancy Childbirth* 2016;**16**. doi:10.1186/S12884-016-0991-Z

40 Sarmiento I, Paredes-Solís S, Dion A, *et al.* Maternal health and Indigenous traditional midwives in southern Mexico: contextualisation of a scoping review. *BMJ Open* 2021;**11**:e054542. doi:10.1136/BMJOPEN-2021-054542

41 Welton NJ, Caldwell DM, Adamopoulos E, *et al.* Mixed Treatment Comparison Meta-Analysis of Complex Interventions: Psychological Interventions in Coronary Heart Disease. *Am J Epidemiol* 2009;**169**:1158–65. doi:10.1093/AJE/KWP014

For peer review only - http://bmjopen.bmj.com/site/about/guidelines.xhtml

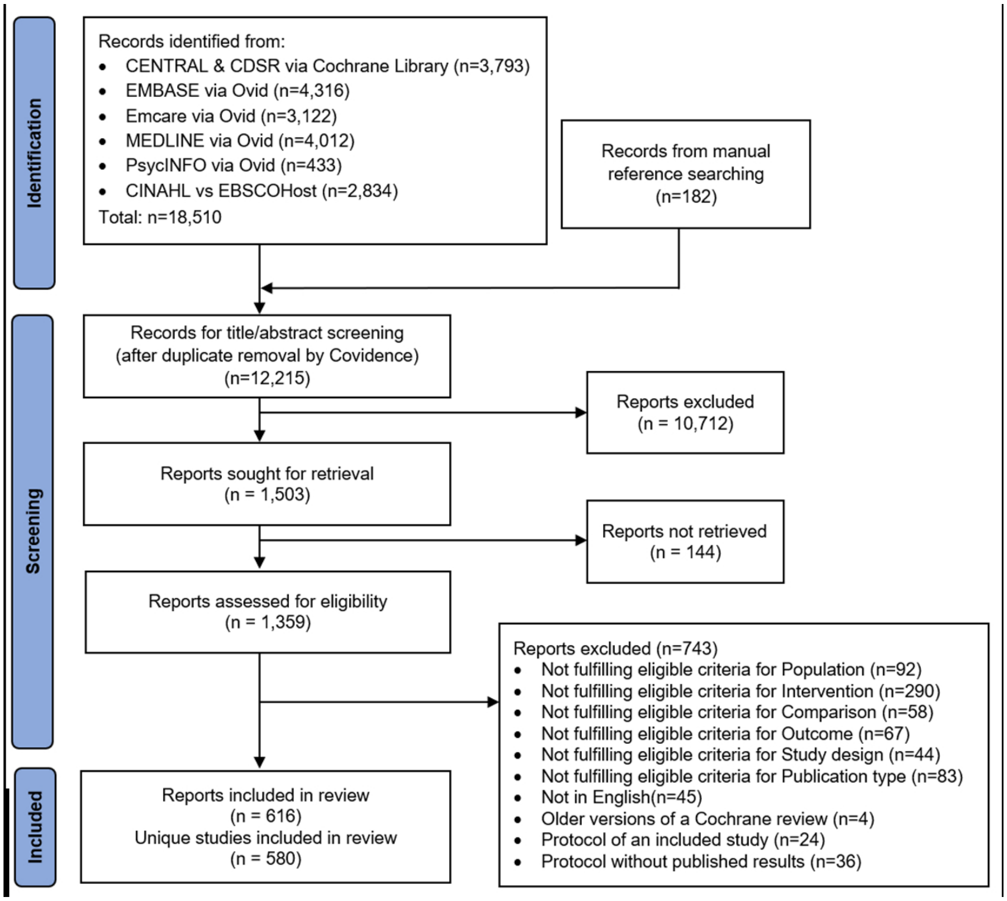

Fig. 1. PRISMA flowchart of record retrieval and selection

102x91mm (300 x 300 DPI)

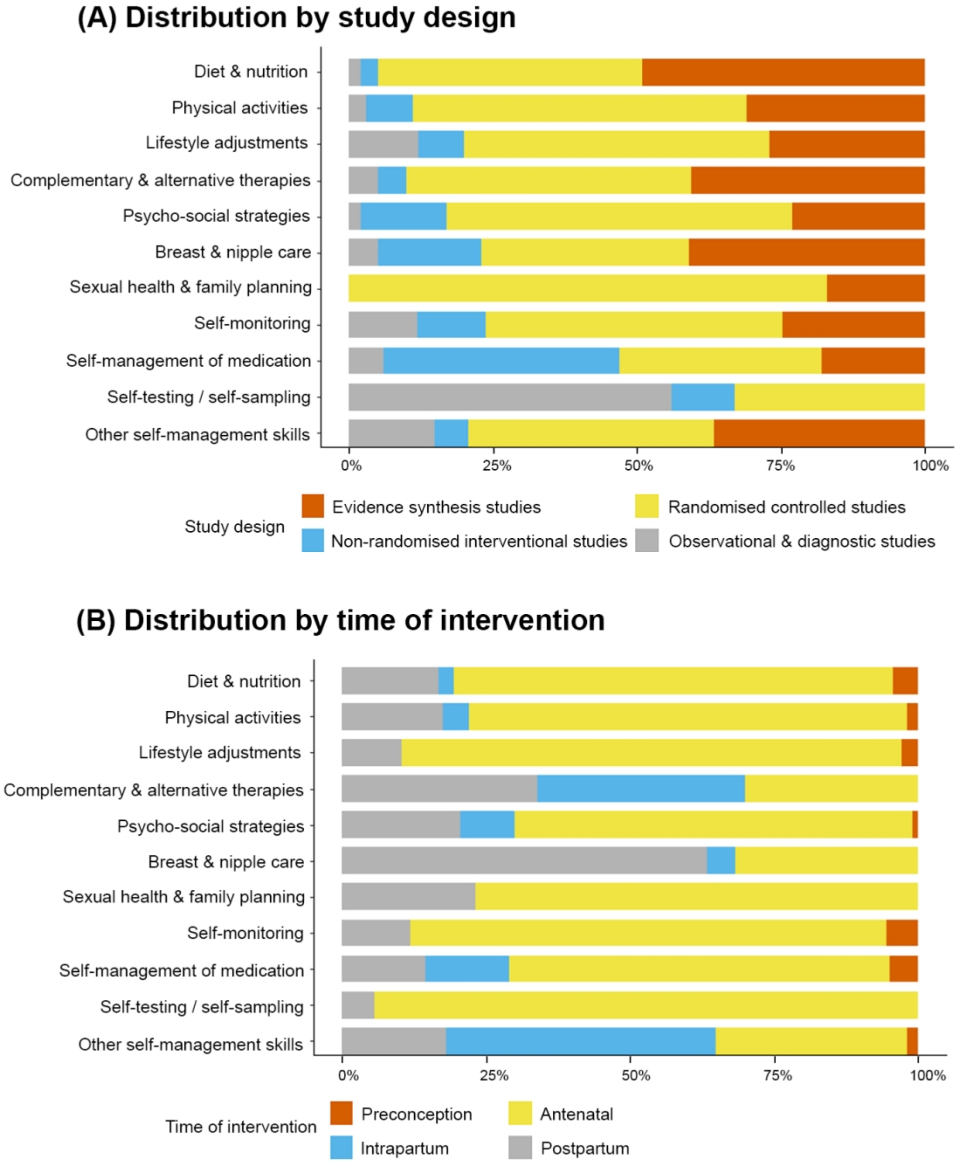

Fig. 2. Characteristics of included studies

118x145mm (300 x 300 DPI)

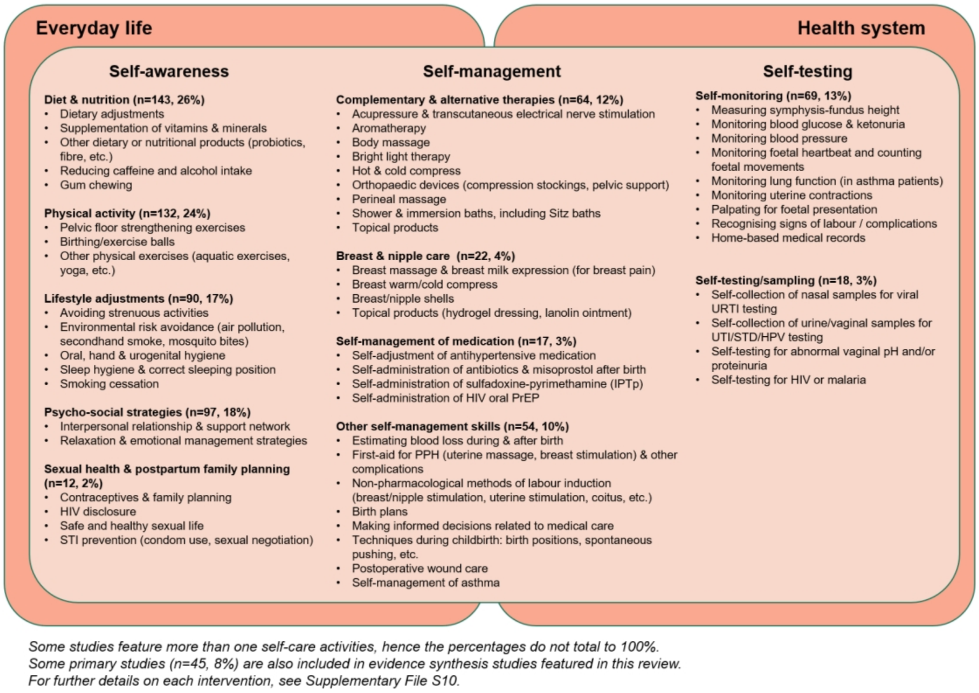

Fig. 3. All self-care interventions identified from included studies and their interactions with the health system

149x105mm (300 x 300 DPI)

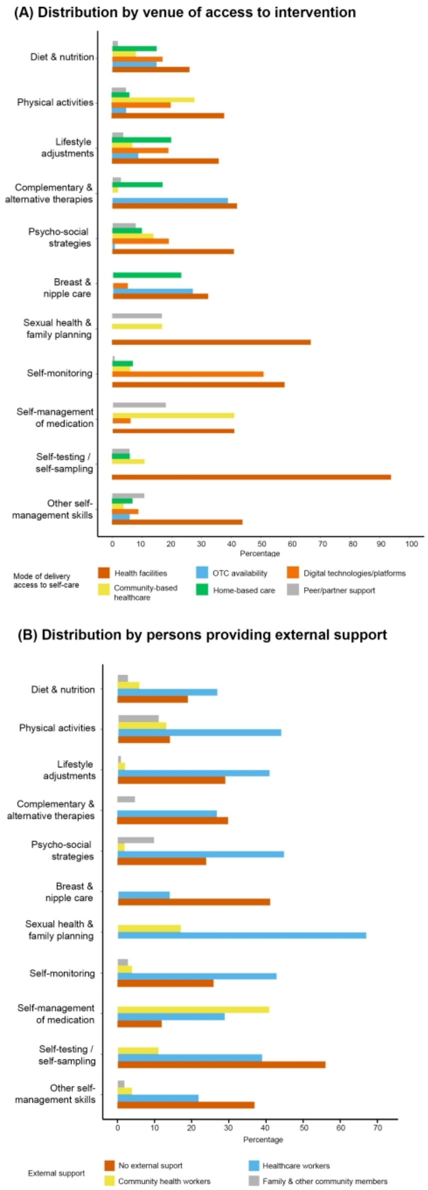

Fig. 4. Characteristics of self-care interventions

131x360mm (300 x 300 DPI)

Title page

Supplementary File S1: Preferred Reporting Items for Systematic reviews and Meta-Analyses extension for Scoping Reviews (PRISMA-ScR) Checklist 2

Supplementary File S2: Protocol as registered on Open Science Framework 4

Supplementary File S3: Search strategy for all databases 8

Supplementary File S4: Defining self-care interventions, a conceptual framework and examples for making judgements on specific interventions 30

Supplementary File S5: Data collection form 34

Supplementary File S6: List of excluded full texts 39

Supplementary File S7: Reference list of included studies 64

Supplementary File S8. Characteristics of studies by category 96

Supplementary File S9: Complex interventions and their characteristics 97

Supplementary File S10: List of all self-care interventions identified from included studies 100

Supplementary File S11: Categorising self-care interventions based on WHO's classification framework 107

For peer review only - http://bmjopen.bmj.com/site/about/guidelines.xhtml

Supplementary File S1. Preferred Reporting Items for Systematic reviews and Meta-Analyses extension for Scoping Reviews (PRISMA-ScR) Checklist

SECTION	ITEM	PRISMA-ScR CHECKLIST ITEM	REPORTED ON PAGE #
**TITLE**			
Title	1	Identify the report as a scoping review.	1
**ABSTRACT**			
Structured summary	2	Provide a structured summary that includes (as applicable): background, objectives, eligibility criteria, sources of evidence, charting methods, results, and conclusions that relate to the review questions and objectives.	2-3
**INTRODUCTION**			
Rationale	3	Describe the rationale for the review in the context of what is already known. Explain why the review questions/objectives lend themselves to a scoping review approach.	4
Objectives	4	Provide an explicit statement of the questions and objectives being addressed with reference to their key elements (e.g., population or participants, concepts, and context) or other relevant key elements used to conceptualize the review questions and/or objectives.	4
**METHODS**			
Protocol and registration	5	Indicate whether a review protocol exists; state if and where it can be accessed (e.g., a Web address); and if available, provide registration information, including the registration number.	5<br>Supp File S1
Eligibility criteria	6	Specify characteristics of the sources of evidence used as eligibility criteria (e.g., years considered, language, and publication status), and provide a rationale.	5-7<br>Supp File S3
Information sources*	7	Describe all information sources in the search (e.g., databases with dates of coverage and contact with authors to identify additional sources), as well as the date the most recent search was executed.	5<br>Supp File S2
Search	8	Present the full electronic search strategy for at least 1 database, including any limits used, such that it could be repeated.	Supp File S2
Selection of sources of evidence†	9	State the process for selecting sources of evidence (i.e., screening and eligibility) included in the scoping review.	6-7
Data charting process‡	10	Describe the methods of charting data from the included sources of evidence (e.g., calibrated forms or forms that have been tested by the team before their use, and whether data charting was done independently or in duplicate) and any processes for obtaining and confirming data from investigators.	6-7<br>Supp File S4
Data items	11	List and define all variables for which data were sought and any assumptions and simplifications made.	7<br>Supp File S4
Critical appraisal of individual sources of evidence§	12	If done, provide a rationale for conducting a critical appraisal of included sources of evidence; describe the methods used and how this information was used in any data synthesis (if appropriate).	N/A
Synthesis of results	13	Describe the methods of handling and summarizing the data that were charted.	7
**RESULTS**			
Selection of sources of evidence	14	Give numbers of sources of evidence screened, assessed for eligibility, and included in the review, with reasons for exclusions at each stage, ideally using a flow diagram.	8<br>Fig 1
Characteristics of sources of evidence	15	For each source of evidence, present characteristics for which data were charted and provide the citations.	N/A
Critical appraisal within sources of evidence	16	If done, present data on critical appraisal of included sources of evidence (see item 12).	N/A
Results of individual sources of evidence	17	For each included source of evidence, present the relevant data that were charted that relate to the review questions and objectives.	N/A
Synthesis of results	18	Summarize and/or present the charting results as they relate to the review questions and objectives.	8-12
**DISCUSSION**			
Summary of evidence	19	Summarize the main results (including an overview of concepts, themes, and types of evidence available), link to the review questions and objectives, and consider the relevance to key groups.	13-4

For peer review only - http://bmjopen.bmj.com/site/about/guidelines.xhtml

SECTION	ITEM	PRISMA-ScR CHECKLIST ITEM	REPORTED ON PAGE #
Limitations	20	Discuss the limitations of the scoping review process.	14-15
Conclusions	21	Provide a general interpretation of the results with respect to the review questions and objectives, as well as potential implications and/or next steps.	15
**FUNDING**			
Funding	22	Describe sources of funding for the included sources of evidence, as well as sources of funding for the scoping review. Describe the role of the funders of the scoping review.	16

From: Tricco AC, Lillie E, Zarin W, O'Brien KK, Colquhoun H, Levac D, et al. PRISMA Extension for Scoping Reviews (PRISMAScR): Checklist and Explanation. Ann Intern Med. 2018;169:467–473. doi: 10.7326/M18-0850.

For peer review only

Supplementary File S2. Protocol as registered on Open Science Framework

Title: Self-care interventions for antenatal, intrapartum and postnatal care: protocol for a scoping review

1. INTRODUCTION

Self-care is defined by the World Health Organization (WHO) as "the ability of individuals, families and communities to promote health, prevent disease, maintain health, and to cope with illness and disability with or without the support of a healthcare provider" (WHO, 2021). Self-care interventions are tools that support self-care, which include "evidence-based, high-quality drugs, devices, diagnostics and/or digital interventions that can be provided fully or partially outside formal health services and be used with or without a health worker" (WHO, 2021). Self-care interventions offer a solution to reduce health disparities and inequity in health access, especially in low-resource settings. By co-opting the pregnant woman and her family, self-care interventions can enhance the overall efficiency of healthcare delivery, while also improving service uptake and adherence to treatment.

There is a large and heterogeneous body of research on maternal and perinatal health interventions that are useful (or promising) from a self-care perspective. In this context, a scoping review can help identify established or emerging self-care interventions that could be considered for developing recommendations.

2. OBJECTIVES

- To identify self-care interventions in antenatal, intrapartum and postnatal care with sufficient evidence that may warrant a WHO recommendation
- To identify emerging self-care interventions that are promising research areas and should be prioritised
- To describe and classify these interventions by self-care concepts, behaviours, mode of delivery and other characteristics

3. METHODS

This protocol was adapted from the methodological guide for scoping reviews (Peters et al., 2015). The review will be reported using the PRISMA for Scoping Reviews extension (Tricco et al., 2018).

3.1. Electronic searches

We will systematically search Medline, Embase, Emcare and PsycINFO (via Ovid), CENTRAL and CDSR (via Cochrane Library) and CINAHL Plus (via EBSCOhost) to identify published studies from inception to current. We have developed a search strategy which combines various self-care concepts with terms related to pregnancy care, based on the framework proposed by Narasimhan et al. (2019). The terms and concepts used were informed by previous reviews of pregnancy care and self-care for other health conditions (e.g. chronic diseases). References of included articles will be manually screened to identify studies.

Table 1. Categories of self-care interventions

Self-management	Self-testing	Self-awareness

For peer review only - http://bmjopen.bmj.com/site/about/guidelines.xhtml

Self-medication	Self-sampling	Self-help
Self-treatment	Self-screening	Self-education
Self-examination	Self-diagnosis	Self-regulation
Self-injection	Self-collection	Self-efficacy
Self-use	Self-monitoring	Self-determination

2. Study selection

Two reviewers will independently screen titles and abstracts to remove irrelevant studies and then review full texts based on eligible criteria below. All stages of screening will be conducted in Covidence (Veritas Health Innovation, Australia). Any disagreement is resolved by discussion or adjudication to a third reviewer.

The eligibility criteria for inclusion are:

3.1 Participants: Women preparing to get pregnant, pregnant women, women giving birth, or women in the postnatal period (up to 42 days after birth), of any age. Studies examining interventions only directed at newborns will be excluded.

3.2 Interventions

For the purpose of this review, a self-care intervention is defined as any tool, resource or strategy designed to promote or facilitate self-care practices by the pregnant woman for the purpose of improving the quality or coverage of maternity health care, and/or improving the health, well-being and experiences of the woman and her baby.

A self-care practice include those that can be taken by a pregnant woman of her own accord towards the goals of (a) information-seeking and improving health literacy, (b) promoting mental health, self-efficacy and self-awareness, (c) increasing physical activity, (d) maintaining a healthy diet, (e) reducing risk and exposure avoidance, (d) good hygiene, and (7) safe and appropriate use of medical products and services.

This definition framework is summarised in Figure 1.

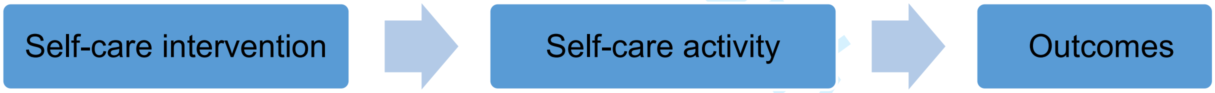

Annex 2 further elaborates on this conceptual framework and outlines the principles that guide the reviewers in judging whether an intervention is considered a self-care intervention.

3.3 Comparator: No intervention, placebo or standard/clinical care; single-arm interventional studies are eligible.

3.4 Outcomes: These interventions must be aimed at reducing risks of any pregnancy, childbirth or postnatal complications and/or enabling a positive pregnancy, childbirth or postnatal experience. The outcomes can be relating to physical, psychological or emotional health, behavioural change or product performance. Interventions measuring social, economic and financial outcomes are excluded from the scope of this review. Feasibility studies evaluating only retention rates and acceptability are excluded.

3.5 Types of studies:

We will include RCTs, quasi-experimental studies and cohort studies, as well as systematic reviews and meta-analyses for mapping. Reviews must state some forms of search strategy

For peer review only - http://bmjopen.bmj.com/site/about/guidelines.xhtml

and present all included studies in a systematic manner to be eligible. No date restriction will be applied. Case series, case studies, case reports, dissertations and theses, reports published as abstracts only, correspondence letters and editorials, qualitative studies will be excluded. Only studies with full texts written in English are eligible.

Quality assessment

In line with scoping review methodology (Peters et al., 2015), quality assessment will not be performed.

Data extraction and analysis

We will collect the following data on study characteristics (study design, country of origin, publication year), population characteristics (generic vs condition-specific, age, individual vs group, cultural/socioeconomic disadvantaged groups), intervention characteristics (provider, location, mode of delivery, period of pregnancy, routine vs condition-specific care, basic description), type of outcomes (physical, psychological or emotional). Interventions will be organised into themes, which are adapted from an earlier WHO's guideline on antenatal care (WHO, 2016) and will be further modified based on an iterative process of analysing retrieved interventions. The current proposed themes are as follows: (1) lifestyle adjustments, including nutritional interventions and physical activities; (2) interventions for emotional and spiritual wellbeing; (3) self-monitoring and assessment to aid decision-making; (4) preventive measures against pregnancy complications; and (5) interventions to alleviate common physiological symptoms.

Two reviewers will perform data extraction independently using a predefined template. Any disagreement is resolved via discussion or adjudication to a third reviewer. Given the rapid and scoping nature of this review, authors will not be contacted for missing data.

Results will be summarised as an evidence map accompanied by a narrative summary. Based on a 2D-intervention-outcome framework (Saran et al, 2018), the evidence map will visually display the type and quantity of evidence for each intervention, classified by outcome type, type of evidence, findings of evidence and sample size. The narrative summary will discuss the scope and strength of the evidence base. We will focus on identifying self-care interventions with high-quality evidence to be taken to scale, interventions that require further evidence synthesis with systematic reviews, and interventions that lack primary evidence.

References

Narasimhan, M., Allotey, P., & Hardon, A. (2019). Self care interventions to advance health and wellbeing: A conceptual framework to inform normative guidance. *BMJ (Online)*, *365*. https://doi.org/10.1136/bmj.l688

Peters, M. D. J., Godfrey, C. M., Khalil, H., McInerney, P., Parker, D., & Soares, C. B. (2015). Guidance for conducting systematic scoping reviews. *International Journal of Evidence-Based Healthcare*, *13*(3), 141–146. https://doi.org/10.1097/XEB.0000000000000050

Saran A, White H. Evidence and gap maps: a comparison of different approaches. Campbell Systematic Reviews. 2018 Jan;14(1):1-38.

Tricco, A. C., Lillie, E., Zarin, W., O'Brien, K. K., Colquhoun, H., Levac, D., Moher, D., Peters, M. D. J., Horsley, T., Weeks, L., Hempel, S., Akl, E. A., Chang, C., McGowan, J., Stewart, L., Hartling, L., Aldcroft, A., Wilson, M. G., Garritty, C., … Straus, S. E. (2018). PRISMA extension for scoping reviews (PRISMA-ScR): Checklist and explanation. In *Annals of Internal Medicine* (Vol.

For peer review only - http://bmjopen.bmj.com/site/about/guidelines.xhtml

169, Issue 7, pp. 467–473). American College of Physicians. https://doi.org/10.7326/M18-0850

WHO. (2016). *Recommendations on antenatal care for a positive pregnancy experience*. World Health Organization. https://www.who.int/publications-detail-redirect/9789241549912

WHO. (2019). *What do we mean by self-care?* World Health Organization. http://www.who.int/reproductivehealth/self-care-interventions/definitions/en/

WHO. (2021). *Guideline on Self-Care Interventions for Health and Well-Being*. World Health Organization. https://www.who.int/publications-detail-redirect/9789240030909

For peer review only

Supplementary File S3. Search strategy for all databases

Cochrane Library <October 17, 2021>

#1	(self next care):ti,ab,kw
#2	(selfcare):ti,ab,kw
#3	MeSH descriptor: [Self Care] explode all trees
#4	((home or "home-based" or homebased) NEAR/3 care):ti,ab,kw
#5	(self near/1 manag*):ti,ab,kw
#6	(selfmanag*):ti,ab,kw
#7	MeSH descriptor: [Self-Management] explode all trees
#8	((home or "home-based" or homebased) near/3 manag*):ti,ab,kw
#9	(self NEXT medicat*):ti,ab,kw
#10	(selfmedicat*):ti,ab,kw
#11	MeSH descriptor: [Self Medication] explode all trees
#12	((home or "home-based" or homebased) near/3 (medicat* or medicine)):ti,ab,kw
#13	(self NEXT treat*):ti,ab,kw
#14	(selftreat*):ti,ab,kw
#15	((home or "home-based" or homebased) near/3 treat*):ti,ab,kw
#16	(self NEXT examin*):ti,ab,kw
#17	(selfexamin*):ti,ab,kw
#18	MeSH descriptor: [Self-Examination] explode all trees
#19	((home or "home-based" or homebased) near/3 exam*):ti,ab,kw
#20	(self NEXT inject*):ti,ab,kw
#21	(selfinject*):ti,ab,kw
#22	((home or "home-based" or homebased) near/3 inject*):ti,ab,kw
#23	(self NEXT administ*):ti,ab,kw
#24	(selfadminist*):ti,ab,kw
#25	MeSH descriptor: [Self Administration] explode all trees
#26	((home or "home-based" or homebased) near/3 (administ* not administrator*)):ti,ab,kw
#27	(self NEXT (use or usage)):ti,ab,kw
#28	(selfuse or selfusage):ti,ab,kw
#29	(self NEXT test*):ti,ab,kw
#30	(selftest*):ti,ab,kw
#31	MeSH descriptor: [Self-Testing] explode all trees
#32	((home or "home-based" or homebased) near/3 (test* or kits or kit)):ti,ab,kw
#33	(self NEXT sampl*):ti,ab,kw
#34	(selfsampl*):ti,ab,kw
#35	((home or "home-based" or homebased) near/3 sampl*):ti,ab,kw
#36	(self NEXT screen*):ti,ab,kw
#37	(selfscreen*):ti,ab,kw
#38	((home or "home-based" or homebased) near/3 screen*):ti,ab,kw
#39	(self NEXT diagnos*):ti,ab,kw
#40	(selfdiagnos*):ti,ab,kw
#41	((home or "home-based" or homebased) near/3 diagnos*):ti,ab,kw
#42	(self NEXT collect*):ti,ab,kw
#43	(selfcollect*):ti,ab,kw
#44	((home or "home-based" or homebased) near/3 collect*):ti,ab,kw
#45	(self NEXT monitor*):ti,ab,kw
#46	(selfmonitor*):ti,ab,kw
#47	((home or "home-based" or homebased) near/3 monitor*):ti,ab,kw
#48	MeSH descriptor: [Blood Glucose Self-Monitoring] explode all trees
#49	(SMBG):ti,ab,kw

For peer review only - http://bmjopen.bmj.com/site/about/guidelines.xhtml

#50	(self NEXT measur*):ti,ab,kw
#51	(selfmeasur*):ti,ab,kw
#52	((home or "home-based" or homebased) near/3 measur*):ti,ab,kw
#53	(self NEXT assess*):ti,ab,kw
#54	(selfassess*):ti,ab,kw
#55	MeSH descriptor: [Self-Assessment] explode all trees
#56	((home or "home-based" or homebased) near/3 assess*):ti,ab,kw
#57	(self NEXT evaluat*):ti,ab,kw
#58	(selfevaluat*):ti,ab,kw
#59	MeSH descriptor: [Diagnostic Self Evaluation] explode all trees
#60	((home or "home-based" or homebased) near/3 evaluat*):ti,ab,kw
#61	(self NEXT aware*):ti,ab,kw
#62	(selfaware*):ti,ab,kw
#63	(self NEXT help*):ti,ab,kw
#64	(selfhelp*):ti,ab,kw
#65	MeSH descriptor: [Self-Help Devices] explode all trees
#66	(self NEXT educat*):ti,ab,kw
#67	(selfeducat*):ti,ab,kw
#68	(self NEXT regulat*):ti,ab,kw
#69	(selfregulat*):ti,ab,kw
#70	MeSH descriptor: [Self-Control] explode all trees
#71	((home or "home-based" or homebased) near/3 control):ti,ab,kw
#72	(self NEXT efficac*):ti,ab,kw
#73	(selfeffica*):ti,ab,kw
#74	MeSH descriptor: [Self Efficacy] explode all trees
#75	(self NEXT determin*):ti,ab,kw
#76	(selfdetermin*):ti,ab,kw
#77	MeSH descriptor: [Personal Autonomy] explode all trees
#78	MeSH descriptor: [Self Disclosure] explode all trees
#79	(self NEXT advoca*):ti,ab,kw
#80	(selfadvoca*):ti,ab,kw
#81	(self NEXT reliance):ti,ab,kw
#82	(selfreliance):ti,ab,kw
#83	(self-adjust* NEAR/3 (medication* or drug* or dosage* or doses or dosing)):ti,ab,kw
#84	(person NEXT cent* NEAR/3 care):ti,ab,kw
#85	(patient NEXT orient* NEAR/3 care):ti,ab,kw
#86	(patient NEXT cent* NEAR/3 care):ti,ab,kw
#87	MeSH descriptor: [Patient-Centered Care] explode all trees
#88	(family cent* NEAR/3 care):ti,ab,kw
#89	("non-pharmacological" or "non-pharmaceutical" or "non-clinical" or "non-medical"):ti,ab,kw
#90	((prevention or preventive) near/3 (infectious or infection*)):ti,kw
#91	(yeast or candida):ti,kw and (prevent* or reduc* or test* or diagnos*):ti,kw
#92	(postpartum NEAR/1 (anemia or anaemia)):ti,kw
#93	(hpv or papillo*):ti,kw and (prevent* or reduc*):ti,kw
#94	(cmv or cytomegalo*):ti,kw and (prevent* or reduc*):ti,kw
#95	(torch and prevent*):ti,kw
#96	(zika and (prevent* or reduc* or practices or strategies)):ti,kw
#97	(malaria:ti) and (prevent* or reduc* or practices or strategies):ti,kw
#98	((mosquito* or "vector-borne") and (prevent* or reduc* or practices or strategies)):ti,kw
#99	(sti or stis or std or stds or "sexually-transmitted"):ti,kw and (prevention or reduc* or practices or strategies):ti,kw
#100	#1 OR #2 OR #3 OR #4 OR #5 OR #6 OR #7 OR #8 OR #9 OR #10 OR #11 OR #12 OR #13 OR #14 OR #15 OR #16 OR #17 OR #18 OR #19 OR #20 OR #21 OR #22 OR #23 OR #24 OR #25 OR #26 OR #27

For peer review only - http://bmjopen.bmj.com/site/about/guidelines.xhtml

	OR #28 OR #29 OR #30 OR #31 OR #32 OR #33 OR #34 OR #35 OR #36 OR #37 OR #38 OR #39 OR #40 OR #41 OR #42 OR #43 OR #44 OR #45 OR #46 OR #47 OR #48 OR #49 OR #50 OR #51 OR #52 OR #53 OR #54 OR #55 OR #56 OR #57 OR #58 OR #59 OR #60 OR #61 OR #62 OR #63 OR #64 OR #65 OR #66 OR #67 OR #68 OR #69 OR #70 OR #71 OR #72 OR #73 OR #74 OR #75 OR #76 OR #77 OR #78 OR #79 OR #80 OR #81 OR #82 OR #83 OR #84 OR #85 OR #86 OR #87 OR #88 OR #89 OR #90 OR #91 OR #92 OR #93 OR #94 OR #95 OR #96 OR #97 OR #98 OR #99
#101	(antenatal or "ante-natal"):TI,KW
#102	(prenatal or "pre-natal"):TI,KW
#103	(perinatal or "peri-natal"):TI,KW
#104	(intrapartum or "intra-partum"):TI,KW
#105	(postpartum or "post-partum"):TI,KW
#106	(postnatal or "post-natal"):TI,KW
#107	(preconception or pre-conception):TI,KW
#108	("pregnancy care" or (pregnancy NEXT outcom*) or (pregnancy NEXT complication*)):TI,KW
#109	("maternal care" or (maternal NEXT outcom*) or (maternal NEXT complication*)):TI,KW
#110	("obstetric care" or (obstetric NEXT outcom*) or (obstetric NEXT complication*)):TI,KW
#111	(birth or childbirth):ti,kw
#112	(labor or labour):ti,kw
#113	(Nulligrav* or nullipar* or primigrav* or primipar* or multigrav* or multipar*):ti,kw
#114	pregnan*:ti,kw
#115	MeSH descriptor: [Pregnancy] explode all trees
#116	MeSH descriptor: [Pregnancy Outcome] explode all trees
#117	MeSH descriptor: [Pregnancy Complications] explode all trees
#118	MeSH descriptor: [Preconception Care] explode all trees
#119	MeSH descriptor: [Prenatal Care] explode all trees
#120	MeSH descriptor: [Perinatal Care] explode all trees
#121	MeSH descriptor: [Postnatal Care] explode all trees
#122	MeSH descriptor: [Prenatal Diagnosis] explode all trees
#123	MeSH descriptor: [Pregnancy Tests] explode all trees
#124	MeSH descriptor: [Prenatal Exposure Delayed Effects] explode all trees
#125	MeSH descriptor: [Prenatal Education] explode all trees
#126	MeSH descriptor: [Maternal Health Services] explode all trees
#127	MeSH descriptor: [Delivery, Obstetric] explode all trees
#128	#101 OR #102 OR #103 OR #104 OR #105 OR #106 OR #107 OR #108 OR #109 OR #110 OR #111 OR #112 OR #113 OR #114 OR #115 OR #116 OR #117 OR #118 OR #119 OR #120 OR #121 OR #122 OR #123 OR #124 OR #125 OR #126 R #127
#129	#100 AND #128
#130	(clinicaltrials.gov):so
#131	(www.who.int):so
#132	#129 not (#130 OR #131)

For peer review only - http://bmjopen.bmj.com/site/about/guidelines.xhtml

CINAHL Plus via EBSCOHost <1937 to October 17, 2021>

S1	TI "self care" OR SU "self care" OR TI selfcare OR SU selfcare
S2	TI (home or "home-based" or homebased) N3 care) OR SU (home or "home-based" or homebased) N3 care)
S3	(MH "Self Care+")
S4	TI "self manag*" OR SU "self manag*" OR TI selfmanag* OR SU selfmanag*
S5	TI (home or "home-based" or homebased) N3 manag*) OR SU (home or "home-based" or homebased) N3 manag*)
S6	(MH "Self-Management")
S7	TI "self medicat*" OR SU "self medicat*" OR TI selfmedicat* OR SU selfmedicat*
S8	TI (home or "home-based" or homebased) N3 (medicat* or medicine)) OR SU (home or "home-based" or homebased) N3 (medicat* or medicine))
S9	(MH "Self Medication")
S10	TI "self treat*" OR SU "self treat*" OR TI selftreat* OR SU selftreat*
S11	TI (home or "home-based" or homebased) N3 treat*) OR SU (home or "home-based" or homebased) N3 treat*)
S12	TI "self examin*" OR SU "self examin*" OR TI selfexamin* OR SU selfexamin*
S13	TI (home or "home-based" or homebased) N3 exam*) OR SU (home or "home-based" or homebased) N3 exam*)
S14	(MH "Vulvar Self-Examination")
S15	TI "self inject*" OR SU "self inject*" OR TI "selfinject*" OR SU "selfinject*"
S16	TI (home or "home-based" or homebased) N3 inject*) OR SU (home or "home-based" or homebased) N3 inject*)
S17	TI "self administ*" OR SU "self administ*" OR TI selfadminist* OR SU selfadminist*"
S18	TI (home or "home-based" or homebased) N3 (administ* not administrator*)) OR SU (home or "home-based" or homebased) N3 (administ* not administrator*))
S19	(MH "Self Administration+")
S20	TI ("self use" or "self usage") OR SU ("self use" or "self usage")
S21	TI (selfuse or selfusage) OR SU (selfuse or selfuage)
S22	TI "self test*" OR SU "self test*" OR TI selftest* OR SU selftest*
S23	TI (home or "home-based" or homebased) N3 (test* or kits or kit)) OR SU (home or "home-based" or homebased) N3 (test* or kits or kit))
S24	(MH "Self-Testing")
S25	TI "self sampl*" OR SU "self sampl*" OR TI selfsampl* OR SU selfsampl*
S26	TI (home or "home-based" or homebased) N3 sampl*) OR SU (home or "home-based" or homebased) N3 sampl*)
S27	TI "self screen*" OR SU "self screen*" OR TI selfscreen* OR SU selfscreen*
S28	TI (home or "home-based" or homebased) N3 screen*) OR SU (home or "home-based" or homebased) N3 screen*)
S29	TI "self diagnos*" OR SU "self diagnos*" OR TI selfdiagnos* OR SU selfdiagnos*
S30	TI (home or "home-based" or homebased) N3 diagnos*) OR SU (home or "home-based" or homebased) N3 diagnos*)
S31	(MH "Self-Diagnosis+")
S32	TI "self collect*" OR SU "self collect*" OR TI selfcollect* OR SU selfcollect*
S33	TI (home or "home-based" or homebased) N3 collect*) OR SU (home or "home-based" or homebased) N3 collect*)
S34	TI "self monitor*" OR SU "self monitor*" OR TI selfmonitor* OR SU selfmonitor*
S35	TI (home or "home-based" or homebased) N3 monitor*) OR SU (home or "home-based" or homebased) N3 monitor*)
S36	(MH "Blood Glucose Self-Monitoring")
S37	TI "self assess*" OR SU "self assess*" OR TI selfassess* OR SU selfassess*
S38	TI (home or "home-based" or homebased) N3 assess*) OR SU (home or "home-based" or homebased) N3 assess*)
S39	(MH "Self Assessment")
S40	TI "self evaluat*" OR SU "self evaluat*" OR TI "selfevaluat*" OR SU "selfevaluat*"

For peer review only - http://bmjopen.bmj.com/site/about/guidelines.xhtml

S41	TI (home or "home-based" or homebased) N3 evaluat*) OR SU (home or "home-based" or homebased) N3 evaluat*)
S42	TI "self aware*" OR SU "self aware*"
S43	TI selfaware* OR SU selfaware*
S44	(MH "Self-Awareness")
S45	TI "self help*" OR SU "self help*"
S46	TI selfhelp* OR SU selfhelp*
S47	TI "self educat*" OR SU "self educat*"
S48	TI selfeducat* OR SU selfeducat*
S49	TI "self regulat*" OR SU "self regulat*"
S50	TI selfregulat* OR SU selfregulat*
S51	(MH "Self Regulation+")
S52	TI "self-control" OR SU "self-control" OR TI selfcontrol OR SU selfcontrol
S53	TI (home or "home-based" or homebased) N3 control) OR SU (home or "home-based" or homebased) N3 control)
S54	TI "self efficac*" OR SU "self efficac*"
S55	TI selfeffica* OR SU selfeffica*
S56	TI "self determin*" OR SU "self determin*"
S57	TI selfdetermin* OR SU selfdetermin*
S58	(MH "Patient Autonomy")
S59	TI ("patient-cent*" N2 care) OR SU ("patient-cent*" N2 care)
S60	TI ("person-cent*" N2 care) OR SU ("person-cent*" N2 care)
S61	(MH "Breast Self-Examination")
S62	TI "patient-orient*" N2 care OR SU "patient-orient*" N2 care
S63	TI SMBG OR SU SMBG
S64	(MH "Self Disclosure")
S65	TI "selfrelian*" OR SU "selfrelian*"
S66	TI "self relian*" OR SU "self relian*"
S67	TI "self advoca*" OR SU "self advoca*"
S68	TI "selfadvoca*" OR SU "selfadvoca*"
S69	(TI (self-adjust* N3 (medication* or drug* or dosage* or doses or dosing))) OR AB ((self-adjust* N3 (medication* or drug* or dosage* or doses or dosing)))
S70	TI "self measur*" OR SU "self measur*" OR TI "selfmeasur*" OR SU "selfmeasur*"
S71	TI (home or "home-based" or homebased) N3 measur*) OR SU (home or "home-based" or homebased) N3 measur*)
S72	TI (("non-pharmacological" or "non-pharmaceutical" or "non-clinical" or "non-medical")) OR SU (("non-pharmacological" or "non-pharmaceutical" or "non-clinical" or "non-medical"))
S73	TI (((prevention or preventive) N3 (infectious or infection*)))
S74	TI ((yeast or candida) and (prevent* or reduc* or test* or diagnos*)) OR SU ((yeast or candida) and (prevent* or reduc* or test* or diagnos*))
S75	TI (postpartum N1 (anemia or anaemia)) OR SU (postpartum N1 (anemia or anaemia))
S76	TI (hpv or papillo*) and (prevent* or reduc*)) OR SU (hpv or papillo*). and (prevent* or reduc*))
S77	TI ((cmv or cytomegalo*) AND (prevent* or reduc*)) OR SU ((cmv or cytomegalo*) AND (prevent* or reduc*))
S78	TI (torch and prevent*) OR SU (torch and prevent*)
S79	TI (zika and (prevent* or reduc* or practices or strategies)) OR SU (zika and (prevent* or reduc* or practices or strategies))
S80	TI (malaria) and (TI (prevent* or reduc* or practices or strategies) OR SU (prevent* or reduc* or practices or strategies))
S81	TI ((mosquito* or "vector-borne") and (prevent* or reduc* or practices or strategies)) OR SU ((mosquito* or "vector-borne") and (prevent* or reduc* or practices or strategies))
S82	TI ((sti or stis or std or stds or "sexually-transmitted") and (prevention or reduc* or practices or strategies)) OR SU ((sti or stis or std or stds or "sexually-transmitted") and (prevention or reduc* or practices or strategies))
S83	S1 OR S2 OR S3 OR S4 OR S5 OR S6 OR S7 OR S8 OR S9 OR S10 OR S11 OR S12 OR S13 OR S14 OR S15 OR S16 OR S17 OR S18 OR S19 OR S20 OR S21 OR S22 OR S23 OR S24 OR S25 OR S26 OR

	S27 OR S28 OR S29 OR S30 OR S31 OR S32 OR S33 OR S34 OR S35 OR S36 OR S37 OR S38 OR S39 OR S40 OR S41 OR S42 OR S43 OR S44 OR S45 OR S46 OR S47 OR S48 OR S49 OR S50 OR S51 OR S52 OR S53 OR S54 OR S55 OR S56 OR S57 OR S58 OR S59 OR S60 OR S61 OR S62 OR S63 OR S64 OR S65 OR S66 OR S67 OR S68 OR S69 OR S70 OR S71 OR S72 OR S73 OR S74 OR S75 OR S76 OR S77 OR S78 OR S79 OR S80 OR S81 OR S82
S84	TI (antenatal or "ante-natal") OR SU (antenatal or "ante-natal")
S85	TI (prenatal or "pre-natal") OR SU (prenatal or "pre-natal")
S86	TI (perinatal or "peri-natal") OR SU (perinatal or "peri-natal")
S87	TI (intrapartum or "intra-partum") OR SU (intrapartum or "intra-partum")
S88	TI (postnatal or "post-natal") OR SU (postnatal or "post-natal")
S89	TI (postpartum or "post-partum") OR SU (postpartum or "post-partum")
S90	TI (preconception or "pre-conception") OR SU (preconception or "pre-conception")
S91	TI (pregnancy N1 (care or outcome* or complication*)) OR SU (pregnancy N1 (care or outcome* or complication*))
S92	TI (maternal N1 (care or outcome* or complication*)) OR SU (maternal N1 (care or outcome* or complication*))
S93	TI (obstetric N1 (care or outcome* or complication*)) OR SU (obstetric N1 (care or outcome* or complication*))
S94	TI (birth or childbirth) OR SU (birth or childbirth)
S95	TI (labor or labour) OR SU (labor or labour)
S96	TI (pregnan*) OR SU (pregnan*)
S97	(MH "Pregnancy Outcomes")
S98	(MH "Pregnancy Complications+")
S99	TI (Nulligrav* or nullipar* or primigrav* or primipar* or multigrav* or multipar*) OR SU (Nulligrav* or nullipar* or primigrav* or primipar* or multigrav* or multipar*)
S100	(MH "Postnatal Period+") OR (MH "Periconceptual Period")
S101	(MH "Pregnancy+") OR (MH "Pregnancy, Multiple") OR (MH "Pregnancy Trimesters+")
S102	(MH "Pregnancy Tests+")
S103	(MH "Prenatal Diagnosis+")
S104	(MH "Prenatal Exposure Delayed Effects") OR (MH "Prenatal Nutritional Physiology")
S105	(MH "Perinatal Risk (Saba CCC)+")
S106	(MH "Postpartum Care (Saba CCC)") OR (MH "Postnatal Care+")
S107	(MH "Prenatal Care") OR (MH "Prenatal Care (Iowa NIC)")
S108	(MH "Intrapartum Care+")
S109	(MH "Childbirth education")
S110	(MH "Maternal Nutritional Physiology+") OR (MH "Maternal Exposure")
S111	(MH "Maternal Health Services+")
S112	S84 OR S85 OR S86 OR S87 OR S88 OR S89 OR S90 OR S91 OR S92 OR S93 OR S94 OR S95 OR S96 OR S97 OR S98 OR S99 OR S100 OR S101 OR S102 OR S103 OR S104 OR S105 OR S106 OR S107 OR S108 OR S109 OR S110 OR S111
S113	(MH "Systematic Review") OR (MH "Meta Analysis")
S114	MH (random assignment) OR MH (cluster sample) OR MH (crossover design) OR MH (placebos)
S115	PT (Systematic review) or PT (clinical trial) OR PT (nursing interventions) OR PT (randomized controlled trial)
S116	(MH "Study Design+") OR (MH "Clinical Research+") OR (MH "Comparative Studies+") OR (MH "Epidemiological Research") OR (MH "Evaluation Research+") OR (MH "Multicenter Studies") OR (MH "Field Studies") OR (MH "Pilot Studies") OR (MH "Secondary Analysis")
S117	TI ((metaanalysis or "meta-analysis")) OR SU ((metaanalysis or "meta-analysis"))
S118	TI (((literature or systematic or quantitative or integrative) N2 (overview or review))) OR SU (((literature or systematic or quantitative or integrative) N2 (overview or review)))
S119	TI ((clinical or randomised or randomized or experimental or control or controlled or blind*) N10 (trial or study)) OR SU ((clinical or randomised or randomized or experimental or control or controlled or blind*) N10 (trial or study))
S120	TI (trial) AND (AB (randomised or randomized or (random* N3 allocat*) or placebo or control or controlled or blinded or blinding) OR SU (randomised or randomized or (random* N3 allocat*) or placebo or control or controlled or blinded or blinding))

For peer review only

For peer review only - http://bmjopen.bmj.com/site/about/guidelines.xhtml

S121	AB ((experiment* or intervention* or study or control or controlled or allocat*) N3 group*)
S122	TI ((pretest or pre-test or posttest or post-test or pre-post)) OR SU ((pretest or pre-test or posttest or post-test or pre-post))
S123	TI ((quasi-experiment* or quasiexperiment*)) OR SU ((quasi-experiment* or quasiexperiment*))
S124	TI (((prospective or retrospective or observational or longitudinal or observational or population or interventional) n4 (analysis or stud* or cohort))) OR SU (((prospective or retrospective or observational or longitudinal or observational or population or interventional) n4 (analysis or stud* or cohort)))
S125	TI (((cohort or evaluation or comparative or pilot) n3 (study or analysis))) OR SU (((cohort or evaluation or comparative or pilot) n3 (study or analysis)))
S126	TI cross-sectional OR SU cross-sectional
S127	TI mixed-methods OR SU mixed-methods
S128	S113 OR S114 OR S115 OR S116 OR S117 OR S118 OR S119 OR S120 OR S121 OR S122 OR S123 OR S124 OR S125 OR S126 OR S127
S129	(MH "Case Studies") OR (MH "Case Control Studies+")
S130	(MH "Qualitative Studies+") OR (MH "Time and Motion Studies+") OR (MH "Genome wide association study+")
S131	PT masters thesis or PT doctoral dissertation OR PT editorial OR PT commentary OR PT case study
S132	TI ("case stud*") or AB ("case stud*" N5 (method* or design))
S133	TI ("case series") or AB ("case series" N5 (method* or design))
S134	TI ("case control*") or AB ("case control*" N5 (method* or design))
S135	TI (editorial or comment or letter or conference or proceeding* or thesis or dissertation)
S136	(((MH "Animals+") OR (MH "Animal Studies") OR (TI "animal model*")) NOT (MH "human"))
S137	AB ((rat or rats or mice or mouse or animal) N3 (model or study or method* or design)) or TI (mice or mouse or rat or rats or "animal study")
S138	S129 OR S130 OR S131 OR S132 OR S133 OR S134 OR S135 OR S136 OR S137
S139	S83 and S112 and S128 NOT S138

For peer review only

Ovid MEDLINE(R) and Epub Ahead of Print, In-Process, In-Data-Review & Other Non-Indexed Citations, Daily and Versions(R) <1946 to October 17, 2021>

1	self-care.ti,kf.
2	selfcare.ti,kf.
3	exp self care/
4	((home or "home-based" or homebased) adj3 care).ti,kf.
5	self-manag*.ti,kf.
6	selfmanag*.ti,kf.
7	exp Self-Management/
8	((home or "home-based" or homebased) adj3 manag*).ti,kf.
9	self-medicat*.ti,kf.
10	selfmedicat*.ti,kf.
11	((home or "home-based" or homebased) adj3 (medicat* or medicine)).ti,kf.
12	exp self medication/
13	self-treat*.ti,kf.
14	selftreat*.ti,kf.
15	((home or "home-based" or homebased) adj3 treat*).ti,kf.
16	self-examin*.ti,kf.
17	selfexamin*.ti,kf.
18	exp self examination/
19	((home or "home-based" or homebased) adj3 exam*).ti,kf.
20	self-inject*.ti,kf.
21	selfinject*.ti,kf.
22	exp self injection/
23	((home or "home-based" or homebased) adj3 inject*).ti,kf.
24	self-administ*.ti,kf.
25	selfadminist*.ti,kf.
26	exp Self Administration/
27	((home or "home-based" or homebased) adj3 (administ* not administrator*)).ti,kf.
28	("self-use" or "self-usage").ti,kf.
29	(selfuse or selfusage).ti,kf.
30	self-test*.ti,kf.
31	selftest*.ti,kf.
32	exp Self-Testing/
33	((home or "home-based" or homebased) adj3 (test* or kits or kit)).ti,kf.
34	self-sampl*.ti,kf.
35	selfsampl*.ti,kf.
36	((home or "home-based" or homebased) adj3 sampl*).ti,kf.
37	self-screen*.ti,kf.
38	selfscreen*.ti,kf.
39	((home or "home-based" or homebased) adj3 screen*).ti,kf.
40	self-diagnos*.ti,kf.
41	selfdiagnos*.ti,kf.
42	((home or "home-based" or homebased) adj3 diagnos*).ti,kf.
43	self-collect*.ti,kf.
44	selfcollect*.ti,kf.
45	((home or "home-based" or homebased) adj3 collect*).ti,kf.
46	self-monitor*.ti,kf.
47	selfmonitor*.ti,kf.
48	((home or "home-based" or homebased) adj3 monitor*).ti,kf.
49	exp Blood Glucose Self-Monitoring/
50	SMBG.ti,kf.

51	self-measur*.ti,kf.
52	selfmeasu*.ti,kf.
53	((home or "home-based" or homebased) adj3 measur*).ti,kf.
54	self-control*.ti,kf.
55	selfcontrol.ti,kf.
56	exp self-control/
57	((home or "home-based" or homebased) adj3 control).ti,kf.
58	self-assess*.ti,kf.
59	selfassess.ti,kf.
60	exp Self-assessment/
61	((home or "home-based" or homebased) adj3 assess*).ti,kf.
62	self-evaluat*.ti,kf.
63	selfevaluat*.ti,kf.
64	exp self evaluation/
65	exp Diagnostic Self Evaluation/
66	((home or "home-based" or homebased) adj3 evaluat*).ti,kf.
67	Self-aware*.ti,kf.
68	selfaware*.ti,kf.
69	self-help*.ti,kf.
70	selfhelp*.ti,kf.
71	exp self help/
72	self-educat*.ti,kf.
73	selfeducat*.ti,kf.
74	self-regulat*.ti,kf.
75	selfregulat*.ti,kf.
76	exp self concept/
77	exp self disclosure/
78	self-effica*.ti,kf.
79	selfeffica*.ti,kf.
80	exp Self-efficacy/
81	self-determin*.ti,kf.
82	selfdetermin*.ti,kf.
83	self-reliance.ti,kf.
84	selfreliance.ti,kf.
85	self-advoc*.ti,kf.
86	selfadvoc*.ti,kf.
87	(self-adjust* adj3 (medication* or drug* or dosage* or doses or dosing)).ti,ab,kf.
88	(patient-cent* adj1 care).ti,kf.
89	(person-cent* adj1 care).ti,kf.
90	(patient-oriented adj1 care).ti,kf.
91	exp Patient-Centered Care/
92	(family-cent* adj1 care).ti,kf.
93	("non-pharmacological" or "non-pharmaceutical" or "non-clinical" or "non-medical").ti,kf.
94	((prevention or preventive) adj3 (infectious or infection*)).ti,kf.
94	(yeast or candida).ti,kf. and (prevent* or reduc* or test* or diagnos*).ti,kf.
95	(postpartum adj1 (anemia or anaemia)).ti,kf.
96	(hpv or papillo*).ti,kf. and (prevent* or reduc*).ti,kf.
97	(cmv or cytomegalo*).ti,kf. and (prevent* or reduc*).ti,kf.
98	torch.ti,kf. and prevent*.ti,ab,kf.
99	(zika and (prevent* or reduc* or practices or strategies)).ti,kf.
100	(malaria.ti.) and (prevent* or reduc* or practices or strategies).ti,kf.
101	((mosquito* or "vector-borne") and (prevent* or reduc* or practices or strategies)).ti,kf.

For peer review only - http://bmjopen.bmj.com/site/about/guidelines.xhtml

102	(sti or stis or std or stds or "sexually-transmitted").ti,kf. and (prevention or reduc* or practices or strategies).ti,kf.
103	or/1-102
104	antenatal.ti,kf.
105	(prenatal or pre-natal).ti,kf.
106	(perinatal or peri-natal).ti,kf.
107	(intrapartum or intra-partum).ti,kf.
108	(postpartum or post-partum).ti,kf.
109	(postnatal or post-natal).ti,kf.
110	(preconception or pre-conception).ti,kf.
111	("pregnancy care" or "pregnancy outcom*" or "pregnancy complication*").ti,kf.
112	("maternal care" or "maternal outcom*" or "maternal complication*").ti,kf.
113	("obstetric care" or "obstetric outcom*" or "obstetric complication*").ti,kf.
114	birth.ti,kf.
115	childbirth.ti,kf.
116	labour.ti,kf.
117	labor.ti,kf.
118	(Nulligrav* or nullipar* or primigrav* or primipar* or multigrav* or multipar*).ti,kf.
119	pregnan*.ti,kf.
120	exp pregnancy/
121	exp pregnancy outcome/
122	exp Pregnancy Complications/
123	exp Preconception care/
124	exp perinatal care/
125	exp prenatal care/
126	exp postnatal care/
127	exp pregnancy tests/
128	exp prenatal diagnosis/
129	exp Prenatal Exposure Delayed Effects/
130	exp prenatal education/
131	exp maternal health services/
132	exp Delivery, Obstetric/
133	or/104-132
134	exp Systematic Review/ or exp meta-analysis/
135	((literature or systematic or quantitative or integrative) adj2 (overview or review)).ti,ab,kf,sh.
136	(metaanalysis or meta-analysis).ti,ab,kf,sh.
137	exp Clinical trial/ or Clinical Study/ or exp Comparative Study/ or exp Evaluation Study/ or exp Multicenter Study/ or exp Observational Study/ or exp Evaluation Studies as Topic/
138	exp Epidemiologic Research Design/ not (Genome-Wide Association Study/ or exp Reproducibility of Results/)
139	exp Epidemiologic Study Characteristics/
140	((clinical or randomised or randomized or experimental or controlled or control or blind*) adj10 (trial or study)).ti,kf.
141	trial.ti. and (randomised or randomized or (random* adj3 allocat*) or placebo or control or controlled or blinded or blinding).ab,kf.
142	((experiment* or intervention* or study or control or controlled or allocat*) adj3 group*).ab.
143	(pretest or "pre-test" or posttest or "post-test" or "pre-post" or "quasi-experiment*" or quasiexperiment* or "cross-sectional" or "mixed-method*").ti,kf.
144	((prospective or retrospective or observational or longitudinal or observational or population or interventional) adj4 (analysis or stud* or cohort)).ti,kf.
145	((cohort or evaluation or comparative or pilot) adj3 (study or analysis)).ti,kf.
146	or/134-145
147	case study.ti. or ("case study" adj5 (method* or design)).ab.
148	case report.ti. or ("case report" adj5 (method* or design)).ab.

For peer review only - http://bmjopen.bmj.com/site/about/guidelines.xhtml

For peer review

149	case series.ti. or ("case series" adj5 (method* or design)).ab.
150	case control.ti. or ("case control" adj5 (method* or design)).ab.
151	(editorial or "conference abstract" or "conference review" or "conference paper" or "poster abstract*" or erratum or commentary or (letter adj3 editor)).ti.
152	(Clinical Conference or Editorial or Letter or Meeting Abstracts or Newspaper Article).pt.
153	exp Case-Control Studies/ or exp Case Reports/ or exp case Studies/ or exp Clinical conference/
154	(animals/ or exp animal experiment/) not (humans/ and animals/)
155	((rat or rats or mice or mouse or animal) adj3 (model or study or method* or design)).ab. or (mice or mouse or rat or rats or "animal study").ti.
156	or/147-155
157	(103 and 133 and 146) not 156

For peer review only

For peer review only - http://bmjopen.bmj.com/site/about/guidelines.xhtml

Embase Classic+Embase <1947 to October 17, 2021>

1	self-care.ti,kw.
2	selfcare.ti,kw.
3	exp self care/
4	((home or "home-based" or homebased) adj3 care).ti,kw.
5	self-manag*.ti,kw.
6	selfmanag*.ti,kw.
7	((home or "home-based" or homebased) adj3 manag*).ti,kw.
8	self-medicat*.ti,kw.
9	selfmedicat*.ti,kw.
10	exp self medication/
11	((home or "home-based" or homebased) adj3 (medicat* or medicine)).ti,kw.
12	self-treat*.ti,kw.
13	selftreat*.ti,kw.
14	((home or "home-based" or homebased) adj3 treat*).ti,kw.
15	self-examin*.ti,kw.
16	selfexamin*.ti,kw.
17	exp self examination/
18	((home or "home-based" or homebased) adj3 exam*).ti,kw.
19	self-inject*.ti,kw.
20	selfinject*.ti,kw.
21	exp self injection/
22	((home or "home-based" or homebased) adj3 inject*).ti,kw.
23	self-administ*.ti,kw.
24	selfadminist*.ti,kw.
25	exp drug self administration/
26	((home or "home-based" or homebased) adj3 (administ* not administrator*)).ti,kw.
27	("self-use" or "self usage").ti,kw.
28	(selfuse or selfusage).ti,kw.
29	self-test*.ti,kw.
30	selftest*.ti,kw.
31	((home or "home-based" or homebased) adj3 (test* or kits or kit)).ti,kw.
32	self-sampl*.ti,kw.
33	selfsampl*.ti,kw.
34	((home or "home-based" or homebased) adj3 sampl*).ti,kw.
35	self-screen*.ti,kw.
36	selfscreen*.ti,kw.
37	((home or "home-based" or homebased) adj3 screen*).ti,kw.
38	self-diagnos*.ti,kw.
39	selfdiagnos*.ti,kw.
40	((home or "home-based" or homebased) adj3 diagnos*).ti,kw.
41	self-collect*.ti,kw.
42	selfcollect*.ti,kw.
43	((home or "home-based" or homebased) adj3 collect*).ti,kw.
44	self-monitor*.ti,kw.
45	selfmonitor*.ti,kw.
46	exp self monitoring/
47	((home or "home-based" or homebased) adj3 monitor*).ti,kw.
48	*self monitoring of blood glucose/
49	*self monitoring of blood pressure/
50	SMBG.ti,kw.
51	self-measur*.ti,kw.

For peer review only - http://bmjopen.bmj.com/site/about/guidelines.xhtml

52	selfmeasu*.ti,kw.
53	((home or "home-based" or homebased) adj3 measur*).ti,kw.
54	self-control*.ti,kw.
55	selfcontrol.ti,kw.
56	exp self-control/
57	((home or "home-based" or homebased) adj3 control).ti,kw.
58	self-assess*.ti,kw.
59	selfassess.ti,kw.
60	((home or "home-based" or homebased) adj3 assess*).ti,kw.
61	self-evaluat*.ti,kw.
62	selfevaluat*.ti,kw.
63	exp self evaluation/
64	((home or "home-based" or homebased) adj3 evaluat*).ti,kw.
65	Self-aware*.ti,kw.
66	selfaware*.ti,kw.
67	self-help*.ti,kw.
68	selfhelp*.ti,kw.
69	exp self help/
70	self-educat*.ti,kw.
71	selfeducat*.ti,kw.
72	self-regulat*.ti,kw.
73	selfregulat*.ti,kw.
74	exp self esteem/
75	exp self disclosure/
76	self-effica*.ti,kw.
77	selfeffica*.ti,kw.
78	self-determin*.ti,kw.
79	selfdetermin*.ti,kw.
80	self-reliance.ti,kw.
81	selfreliance.ti,kw.
82	self-advoc*.ti,kw.
83	selfadvoc*.ti,kw.
84	(self-adjust* adj3 (medication* or drug* or dosage* or doses or dosing)).tw.
85	(patient-cent* adj1 care).ti,kw.
86	(person-cent* adj1 care).ti,kw.
87	(patient-oriented adj1 care).ti,kw.
88	(family-cent* adj1 care).ti,kw.
89	("non-pharmacological" or "non-pharmaceutical" or "non-clinical" or "non-medical").ti,kw.
90	((prevention or preventive) adj3 (infectious or infection*)).ti,kw.
91	(yeast or candida).ti,kw. and (prevent* or reduc* or test* or diagnos*).ti,kw.
92	(postpartum adj1 (anemia or anaemia)).ti,kw.
93	(hpv or papillo*).ti,kw. and (prevent* or reduc*).ti,kw.
94	(cmv or cytomegalo*).ti,kw. and (prevent* or reduc*).ti,kw.
95	torch.ti,kw. and prevent*.ti,ab,kw.
96	(zika and (prevent* or reduc* or practices or strategies)).ti,kw.
97	(malaria.ti.) and (prevent* or reduc* or practices or strategies).ti,kw.
98	((mosquito* or "vector-borne") and (prevent* or reduc* or practices or strategies)).ti,kw.
99	(sti or stis or std or stds or "sexually-transmitted").ti,kw. and (prevention or reduc* or practices or strategies).ti,kw.
100	or/1-99
101	(preconception or pre-conception).ti,kw.
102	antenatal.ti,kw.
103	(prenatal or pre-natal).ti,kw.

For peer review only - http://bmjopen.bmj.com/site/about/guidelines.xhtml

104	(perinatal or peri-natal).ti,kw.
105	(intrapartum or intra-partum).ti,kw.
106	(postpartum or post-partum).ti,kw.
107	(postnatal or post-natal).ti,kw.
108	("pregnancy care" or "pregnancy outcom*" or "pregnancy complication*").ti,kw.
109	("maternal care" or "maternal outcom*" or "maternal complication*").ti,kw.
110	("obstetric care" or "obstetric outcom*" or "obstetric complication*").ti,kw.
111	birth.ti,kw.
112	childbirth.ti,kw.
113	labour.ti,kw.
114	labor.ti,kw.
115	(Nulligrav* or nullipar* or primigrav* or primipar* or multigrav* or multipar*).ti,kw.
116	pregnan*.ti,kw.
117	exp pregnancy/
118	exp pregnancy complication/
119	exp pregnancy outcome/
120	exp obstetric procedure/
121	exp maternal health services/
122	exp prenatal exposure/
123	exp childbirth education/
124	or/101-123
125	exp "systematic review"/ or exp meta analysis/ or "systematic review (topic)"/ or "meta analysis (topic)"/
126	(review or meta-analysis).pt.
127	(metaanalysis or meta-analysis).ti,ab,kw,sh.
128	((literature or systematic or quantitative or integrative) adj2 (overview or review)).ti,ab,kw,sh.
129	exp clinical study/ or exp comparative study/ or exp experimental study/ or exp feasibility study/ or exp field study/ or exp observational study/ or exp quasi experimental study/ or exp pilot study/ or exp experimental study/ or exp comparative study/
130	clinical trial (topic)/ or parallel design/ or crossover procedure/ or triple blind procedure/ or double blind procedure/ or single blind procedure/
131	exp methodology/ and (randomization/ or placebo/)
132	control group/ or nonequivalent control group/
133	intention to treat analysis/ or secondary analysis/
134	pretest posttest control group design/ or experimental design/ or quantitative study/ or participatory research/
135	cohort analysis/ or correlational study/ or cross-sectional study/
136	((clinical or randomised or randomized or experimental or controlled or control or blind*) adj10 (trial or study)).ti,kw.
137	trial.ti. and (randomised or randomized or (random* adj3 allocat*) or placebo or control or controlled or blinded or blinding).ab,kw.
138	((experiment* or intervention* or study or control or controlled or allocat*) adj3 group*).ab.
139	((prospective or retrospective or observational or longitudinal or observational or population or interventional) adj4 (analysis or stud* or cohort)).ti,kw.
140	((cohort or evaluation or comparative or pilot) adj3 (study or analysis)).ti,kw.
141	(pretest or "pre-test" or posttest or "post-test" or "pre-post" or "quasi-experiment*" or quasiexperiment* or "cross-sectional" or "mixed-method*").ti,kw.
142	or/125-141
143	exp case control study/ or exp case report/ or exp case study/
144	case study.ti. or ("case study" adj5 (method* or design)).ab.
145	case report.ti. or ("case report" adj5 (method* or design)).ab.
146	case series.ti. or ("case series" adj5 (method* or design)).ab.
147	case control.ti. or ("case control" adj5 (method* or design)).ab.
148	(editorial or conference or erratum or letter or note).pt.
149	(editorial or "conference abstract" or "conference review" or "conference paper" or "poster abstract*" or erratum or commentary or (letter adj3 editor)).ti.

For peer review only - http://bmjopen.bmj.com/site/about/guidelines.xhtml

150	(animals/ or exp animal experiment/) not (humans/ and animals/)
151	((rat or rats or mice or mouse or animal) adj3 (model or study or method* or design)).ab. or (mice or mouse or rat or rats or "animal study").ti.
152	or/143-151
153	(100 and 124 and 142) not 152

For peer review only

For peer review only - http://bmjopen.bmj.com/site/about/guidelines.xhtml

Ovid Emcare <1995 to 2021 Week 41>

1	self-care.ti,kw.
2	selfcare.ti,kw.
3	exp self care/
4	((home or "home-based" or homebased) adj3 care).ti,kw.
5	self-manag*.ti,kw.
6	selfmanag*.ti,kw.
7	((home or "home-based" or homebased) adj3 manag*).ti,kw.
8	self-medicat*.ti,kw.
9	selfmedicat*.ti,kw.
10	exp self medication/
11	((home or "home-based" or homebased) adj3 (medicat* or medicine)).ti,kw.
12	self-treat*.ti,kw.
13	selftreat*.ti,kw.
14	((home or "home-based" or homebased) adj3 treat*).ti,kw.
15	self-examin*.ti,kw.
16	selfexamin*.ti,kw.
17	exp self examination/
18	((home or "home-based" or homebased) adj3 exam*).ti,kw.
19	self-inject*.ti,kw.
20	selfinject*.ti,kw.
21	exp self injection/
22	((home or "home-based" or homebased) adj3 inject*).ti,kw.
23	self-administ*.ti,kw.
24	selfadminist*.ti,kw.
25	exp drug self administration/
26	((home or "home-based" or homebased) adj3 (administ* not administrator*)).ti,kw.
27	("self-use" or "self usage").ti,kw.
28	(selfuse or selfusage).ti,kw.
29	self-test*.ti,kw.
30	selftest*.ti,kw.
31	((home or "home-based" or homebased) adj3 (test* or kits or kit)).ti,kw.
32	self-sampl*.ti,kw.
33	selfsampl*.ti,kw.
34	((home or "home-based" or homebased) adj3 sampl*).ti,kw.
35	self-screen*.ti,kw.
36	selfscreen*.ti,kw.
37	((home or "home-based" or homebased) adj3 screen*).ti,kw.
38	self-diagnos*.ti,kw.
39	selfdiagnos*.ti,kw.
40	((home or "home-based" or homebased) adj3 diagnos*).ti,kw.
41	self-collect*.ti,kw.
42	selfcollect*.ti,kw.
43	((home or "home-based" or homebased) adj3 collect*).ti,kw.
44	self-monitor*.ti,kw.
45	selfmonitor*.ti,kw.
46	exp self monitoring/
47	((home or "home-based" or homebased) adj3 monitor*).ti,kw.
48	*self monitoring of blood glucose/
49	*self monitoring of blood pressure/
50	SMBG.ti,kw.
51	self-measur*.ti,kw.

For peer review only - http://bmjopen.bmj.com/site/about/guidelines.xhtml

52	selfmeasu*.ti,kw.
53	((home or "home-based" or homebased) adj3 measur*).ti,kw.
54	self-control*.ti,kw.
55	selfcontrol.ti,kw.
56	exp self-control/
57	((home or "home-based" or homebased) adj3 control).ti,kw.
58	self-assess*.ti,kw.
59	selfassess.ti,kw.
60	((home or "home-based" or homebased) adj3 assess*).ti,kw.
61	self-evaluat*.ti,kw.
62	selfevaluat*.ti,kw.
63	exp self evaluation/
64	((home or "home-based" or homebased) adj3 evaluat*).ti,kw.
65	Self-aware*.ti,kw.
66	selfaware*.ti,kw.
67	self-help*.ti,kw.
68	selfhelp*.ti,kw.
69	exp self help/
70	self-educat*.ti,kw.
71	selfeducat*.ti,kw.
72	self-regulat*.ti,kw.
73	selfregulat*.ti,kw.
74	exp self esteem/
75	exp self disclosure/
76	self-effica*.ti,kw.
77	selfeffica*.ti,kw.
78	self-determin*.ti,kw.
79	selfdetermin*.ti,kw.
80	self-reliance.ti,kw.
81	selfreliance.ti,kw.
82	self-advoc*.ti,kw.
83	selfadvoc*.ti,kw.
84	(self-adjust* adj3 (medication* or drug* or dosage* or doses or dosing)).tw.
85	(patient-cent* adj1 care).ti,kw.
86	(person-cent* adj1 care).ti,kw.
87	(patient-oriented adj1 care).ti,kw.
88	(family-cent* adj1 care).ti,kw.
89	("non-pharmacological" or "non-pharmaceutical" or "non-clinical" or "non-medical").ti,kw.
90	((prevention or preventive) adj3 (infectious or infection*)).ti,kw.
91	(yeast or candida).ti,kw. and (prevent* or reduc* or test* or diagnos*).ti,kw.
92	(postpartum adj1 (anemia or anaemia)).ti,kw.
93	(hpv or papillo*).ti,kw. and (prevent* or reduc*).ti,kw.
94	(cmv or cytomegalo*).ti,kw. and (prevent* or reduc*).ti,kw.
95	torch.ti,kw. and prevent*.ti,ab,kw.
96	(zika and (prevent* or reduc* or practices or strategies)).ti,kw.
97	(malaria.ti.) and (prevent* or reduc* or practices or strategies).ti,kw.
98	((mosquito* or "vector-borne") and (prevent* or reduc* or practices or strategies)).ti,kw.
99	(sti or stis or std or stds or "sexually-transmitted").ti,kw. and (prevention or reduc* or practices or strategies).ti,kw.
100	or/1-99
101	(preconception or pre-conception).ti,kw.
102	antenatal.ti,kw.
103	(prenatal or pre-natal).ti,kw.

104	(perinatal or peri-natal).ti,kw.
105	(intrapartum or intra-partum).ti,kw.
106	(postpartum or post-partum).ti,kw.
107	(postnatal or post-natal).ti,kw.
108	("pregnancy care" or "pregnancy outcom*" or "pregnancy complication*").ti,kw.
109	("maternal care" or "maternal outcom*" or "maternal complication*").ti,kw.
110	("obstetric care" or "obstetric outcom*" or "obstetric complication*").ti,kw.
111	birth.ti,kw.
112	childbirth.ti,kw.
113	labour.ti,kw.
114	labor.ti,kw.
115	(Nulligrav* or nullipar* or primigrav* or primipar* or multigrav* or multipar*).ti,kw.
116	pregnan*.ti,kw.
117	exp pregnancy/
118	exp pregnancy complication/
119	exp pregnancy outcome/
120	exp obstetric procedure/
121	exp maternal health services/
122	exp prenatal exposure/
123	exp childbirth education/
124	or/101-123
125	exp "systematic review"/ or exp meta analysis/ or "systematic review (topic)"/ or "meta analysis (topic)"/
126	(review or meta-analysis).pt.
127	(metaanalysis or meta-analysis).ti,ab,kw,sh.
128	((literature or systematic or quantitative or integrative) adj2 (overview or review)).ti,ab,kw,sh.
129	exp clinical study/ or exp comparative study/ or exp experimental study/ or exp feasibility study/ or exp field study/ or exp observational study/ or exp quasi experimental study/ or exp pilot study/ or exp experimental study/ or exp comparative study/
130	clinical trial (topic)/ or parallel design/ or crossover procedure/ or triple blind procedure/ or double blind procedure/ or single blind procedure/
131	exp methodology/ and (randomization/ or placebo/)
132	control group/ or nonequivalent control group/
133	intention to treat analysis/ or secondary analysis/
134	pretest posttest control group design/ or experimental design/ or quantitative study/ or participatory research/
135	cohort analysis/ or correlational study/ or cross-sectional study/
136	((clinical or randomised or randomized or experimental or controlled or control or blind*) adj10 (trial or study)).ti,kw.
137	trial.ti. and (randomised or randomized or (random* adj3 allocat*) or placebo or control or controlled or blinded or blinding).ab,kw.
138	((experiment* or intervention* or study or control or controlled or allocat*) adj3 group*).ab.
139	((prospective or retrospective or observational or longitudinal or observational or population or interventional) adj4 (analysis or stud* or cohort)).ti,kw.
140	((cohort or evaluation or comparative or pilot) adj3 (study or analysis)).ti,kw.
141	(pretest or "pre-test" or posttest or "post-test" or "pre-post" or "quasi-experiment*" or quasiexperiment* or "cross-sectional" or "mixed-method*").ti,kw.
142	or/125-141
143	exp case control study/ or exp case report/ or exp case study/
144	case study.ti. or ("case study" adj5 (method* or design)).ab.
145	case report.ti. or ("case report" adj5 (method* or design)).ab.
146	case series.ti. or ("case series" adj5 (method* or design)).ab.
147	case control.ti. or ("case control" adj5 (method* or design)).ab.
148	(editorial or conference or erratum or letter or note).pt.
149	(editorial or "conference abstract" or "conference review" or "conference paper" or "poster abstract*" or erratum or commentary or (letter adj3 editor)).ti.

For peer review only - http://bmjopen.bmj.com/site/about/guidelines.xhtml

150	(animals/ or exp animal experiment/) not (humans/ and animals/)
151	((rat or rats or mice or mouse or animal) adj3 (model or study or method* or design)).ab. or (mice or mouse or rat or rats or "animal study").ti.
152	or/143-151
153	(100 and 124 and 142) not 152

For peer review only

For peer review only - http://bmjopen.bmj.com/site/about/guidelines.xhtml

Ovid APA PsycInfo <1806 to October Week 2 2021>

1	self-care.ti,id.
2	selfcare.ti,id.
3	exp self care/
4	((home or "home-based" or homebased) adj3 care).ti,id.
5	self-manag*.ti,id.
6	selfmanag*.ti,id.
7	exp Self-Management/
8	((home or "home-based" or homebased) adj3 manag*).ti,id.
9	self-medicat*.ti,id.
10	selfmedicat*.ti,id.
11	exp self-medication/
12	((home or "home-based" or homebased) adj3 (medicat* or medicine)).ti,id.
13	self-treat*.ti,id.
14	selftreat*.ti,id.
15	((home or "home-based" or homebased) adj3 treat*).ti,id.
16	self-examin*.ti,id.
17	selfexamin*.ti,id.
18	exp self examination/
19	exp "Self-Examination (Medical)"/
20	((home or "home-based" or homebased) adj3 exam*).ti,id.
21	self-inject*.ti,id.
22	selfinject*.ti,id.
23	((home or "home-based" or homebased) adj3 inject*).ti,id.
24	self-administ*.ti,id.
25	selfadminist*.ti,id.
26	exp drug self administration/
27	((home or "home-based" or homebased) adj3 (administ* not administrator*)).ti,id.
28	("self-use" or "self-usage").ti,id.
29	(selfuse or selfusage).ti,id.
30	self-test*.ti,id.
31	selftest*.ti,id.
32	((home or "home-based" or homebased) adj3 (test* or kits or kit)).ti,id.
33	self-sampl*.ti,id.
34	selfsampl*.ti,id.
35	((home or "home-based" or homebased) adj3 sampl*).ti,id.
36	self-screen*.ti,id.
37	selfscreen*.ti,id.
38	((home or "home-based" or homebased) adj3 screen*).ti,id.
39	self-diagnos*.ti,id.
40	selfdiagnos*.ti,id.
41	((home or "home-based" or homebased) adj3 diagnos*).ti,id.
42	self-collect*.ti,id.
43	selfcollect*.ti,id.
44	((home or "home-based" or homebased) adj3 collect*).ti,id.
45	self-monitor*.ti,id.
46	selfmonitor*.ti,id.
47	exp self-monitoring/
48	((home or "home-based" or homebased) adj3 monitor*).ti,id.
49	SMBG.ti,id.
50	self-measur*.ti,id.
51	selfmeasu*.ti,id.

For peer review only - http://bmjopen.bmj.com/site/about/guidelines.xhtml

52	((home or "home-based" or homebased) adj3 measur*).ti,id.
53	self-control*.ti,id.
54	selfcontrol.ti,id.
55	exp self-control/
56	((home or "home-based" or homebased) adj3 control).ti,id.
57	self-assess*.ti,id.
58	selfassess.ti,id.
59	((home or "home-based" or homebased) adj3 assess*).ti,id.
60	self-evaluat*.ti,id.
61	selfevaluat*.ti,id.
62	exp self-evaluation/
63	((home or "home-based" or homebased) adj3 evaluat*).ti,id.
64	Self-aware*.ti,id.
65	selfaware*.ti,id.
66	self-perception/
67	self-help*.ti,id.
68	selfhelp*.ti,id.
69	exp self help/
70	self-educat*.ti,id.
71	selfeducat*.ti,id.
72	exp self-knowledge/
73	self-regulat*.ti,id.
74	selfregulat*.ti,id.
75	exp self-regulation/
76	exp emotional control/
77	exp emotional regulation/
78	exp emotional adjustment/
79	exp self esteem/
80	exp self disclosure/
81	self-effica*.ti,id.
82	selfeffica*.ti,id.
83	exp self-efficacy/
84	self-determin*.ti,id.
85	selfdetermin*.ti,id.
86	self-reliance.ti,id.
87	selfreliance.ti,id.
88	self-advoc*.ti,id.
89	selfadvoc*.ti,id.
90	exp self-advocacy/
91	(self-adjust* adj3 (medication* or drug* or dosage* or doses or dosing)).ti,id.
92	(patient-cent* adj1 care).ti,id.
93	(person-cent* adj1 care).ti,id.
94	(patient-oriented adj1 care).ti,id.
95	exp Patient Centered Care/
96	(family-cent* adj1 care).ti,id.
97	("non-pharmacological" or "non-pharmaceutical" or "non-clinical" or "non-medical").ti,id.
98	((prevention or preventive) adj3 (infectious or infection*)).ti,id.
99	(yeast or candida).ti,id. and (prevent* or reduc* or test* or diagnos*).ti,id.
100	(postpartum adj1 (anemia or anaemia)).ti,id.
101	(hpv or papillo*).ti,id. and (prevent* or reduc*).ti,id.
102	(cmv or cytomegalo*).ti,id. and (prevent* or reduc*).ti,id.
103	torch.ti,id. and prevent*.ti,ab,id.

For peer review only - http://bmjopen.bmj.com/site/about/guidelines.xhtml

104	(zika and (prevent* or reduc* or practices or strategies)).ti,id.
105	(malaria.ti.) and (prevent* or reduc* or practices or strategies).ti,id.
106	((mosquito* or "vector-borne") and (prevent* or reduc* or practices or strategies)).ti,id.
107	(sti or stis or std or stds or "sexually-transmitted").ti,id. and (prevention or reduc* or practices or strategies).ti,id.
108	or/1-107
109	antenatal.ti,id.
110	(prenatal or pre-natal).ti,id.
111	(perinatal or peri-natal).ti,id.
112	(intrapartum or intra-partum).ti,id.
113	(postpartum or post-partum).ti,id.
114	(postnatal or post-natal).ti,id.
115	(preconception or pre-conception).ti,id.
116	("pregnancy care" or "pregnancy outcom*" or "pregnancy complication*").ti,id.
117	("maternal care" or "maternal outcom*" or "maternal complication*").ti,id.
118	("obstetric care" or "obstetric outcom*" or "obstetric complication*").ti,id.
119	pregnan*.ti,id.
120	(childbirth or birth).ti,id.
121	(labor or labour).ti,id.
122	(Nulligrav* or nullipar* or primigrav* or primipar* or multigrav* or multipar*).ti,id.
123	exp pregnancy/
124	exp prenatal care/ or exp prenatal diagnosis/ or exp prenatal exposure/
125	exp perinatal period/ or exp antepartum period/ or exp intrapartum period exp postnatal period/ or exp perinatal period/
126	or/109-125
127	exp Systematic Review/ or exp meta-analysis/
128	((literature or systematic or quantitative or integrative) adj2 (overview or review)).ti,ab,id,sh.
129	(metaanalysis or meta-analysis).ti,ab,id,sh.
130	exp experimental design/ not exp single-case experimental design/
131	exp cohort analysis/ or exp mixed methods research/ or exp quantitative methods/ or exp quasi-experimental methods/
132	((clinical or randomised or randomized or experimental or controlled or control or blind*) adj10 (trial or study)).ti,id.
133	trial.ti. and (randomised or randomized or (random* adj3 allocat*) or placebo or control or controlled or blinded or blinding).ab,id.
134	((experiment* or intervention* or study or control or controlled or allocat*) adj3 group*).ab.
135	(pretest or "pre-test" or posttest or "post-test" or "pre-post" or "quasi-experiment*" or quasiexperiment* or "cross-sectional" or "mixed-method*").ti,id.
136	((prospective or retrospective or observational or longitudinal or observational or population or interventional) adj4 (analysis or stud* or cohort)).ti,id.
137	((cohort or evaluation or comparative or pilot) adj3 (study or analysis)).ti,id.
138	or/127-137
139	(editorial or "newspaper article" or conference* or commentary).ti.
140	case study.ti. or ("case study" adj5 (method* or design)).ab.
141	case report.ti. or ("case report" adj5 (method* or design)).ab.
142	case series.ti. or ("case series" adj5 (method* or design)).ab.
143	case control.ti. or ("case control" adj5 (method* or design)).ab.
144	exp Case Report/ or exp Case-control studies/ or exp cross-sectional studies/
145	(animals/ or exp animal experiment/) not (humans/ and animals/)
146	((rat or rats or mice or mouse or animal) adj3 (model or study or method* or design)).ab. or (mice or mouse or rat or rats or "animal study").ti.
147	or/139-146
148	(108 and 126 and 138) not 147

For peer review only - http://bmjopen.bmj.com/site/about/guidelines.xhtml

Supplementary File S4. Defining self-care interventions, a conceptual framework and examples for making judgements on specific interventions

Definitions of self-care in WHO Guideline on Self-care Interventions for Health and Wellbeing (2021)

Self-care is the ability of individuals, families and communities to promote health, prevent disease, maintain health, and cope with illness and disability with or without the support of a health worker.

Self-care recognizes individuals as active agents in managing their own health care, in areas including health promotion; disease prevention and control; self-medication; providing care to dependent persons, and rehabilitation, including palliative care.

Self-care interventions are evidence-based, quality tools that support self-care. They include medicines, counselling, diagnostics and/or digital technologies which can be accessed fully or partially outside of formal health services. Depending on the intervention, they can be used with or without the direct supervision of health workers.

Working definition of self-care in this review

For the purpose of this review, a *self-care intervention* is defined as any tool, resource or strategy designed to promote or facilitate self-care by women preparing to get pregnant, currently pregnant, labouring/birthing or in the postpartum period (up to 42 days after birth), for the purpose of improving the quality or coverage of maternity health care, and/or improving the health, well-being and experiences of the pregnant woman and her newborn.

A *self-care activity* includes those that can be taken by a preconceiving, pregnant, labouring/birthing or postpartum woman of her own accord towards the goals of (a) information-seeking and improving health literacy, (b) promoting mental health, self-efficacy and self-awareness, (c) increasing physical activity, (d) maintaining a healthy diet, (e) reducing risk and exposure avoidance, (d) good hygiene, and (7) safe and appropriate use of products, services, diagnostics and medicines.

This definition framework is summarised as:

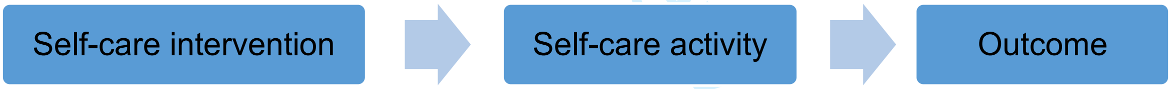

General principles

The following text considers several components of interventions that may have bearing on whether an intervention could be considered a self-care intervention or not.

1. **Place of access**

   The self-care intervention might take place partially or fully outside formal health settings (e.g. outpatient services, clinics and hospitals). These places of access include, but are not limited to pharmacies, home, school and/or digital platforms.

2. **Intersection with health systems**

   The intervention can be entirely initiated by the woman, or may involve some element of health worker (HW) support or engagement. In all instances, the woman must have the autonomy of her own self-care independent of a HW's presence.

   (a) If the intervention relates to an informed action that can only be undertaken by the preconceiving, pregnant, labouring/birthing or postpartum woman (e.g. physical activity, daily dietary intake, etc.), it is considered self-care. Such an intervention may utilise

For peer review only - http://bmjopen.bmj.com/site/about/guidelines.xhtml

measures delivered by HW to facilitate or support the individual's self-care activity, for example by providing information, skills or tools.

> Example: Smoking cessation
>
> HW-led interventions aimed at promoting self-help for smoking cessation were eligible. These include HWs providing self-help materials (brochures, videos, online sources) or conducting educational sessions to give self-help instructions so that the woman can initiate smoking cessation at home.
>
> Interventions requiring intensive and regular contacts with physicians, therapists or nurses and without a fixed number of sessions were not considered self-care interventions. Interventions solely focusing on motivation enhancing e.g. motivational interviewing are not considered self-care interventions as these are not designed to develop skills and self-efficacy.

> Example: Acupuncture, transcutaneous electrical nerve stimulation (TENS), acupressure and massage
>
> Acupuncture is eligible if women are provided with and trained to operate a suitable device. TENS is eligible as TENS machines are easy to operate without prior training. Acupressure and massage, which do not require specific devices, are also eligible if administered by the woman or her partner.

> Example: Pain relief during labour
>
> Non-pharmacological therapies (e.g. aromatherapy, body massage, showering or water baths) are considered self-care if taught to the pregnant women or their caregivers during birth preparation workshops, or instructed to carry out at home, prior to admission. Studies where these interventions were administered by birth attendants or clinic staff are not eligible.

(b) If the intervention relates to care usually provided within the health system (e.g. testing, prescribing or administering medications), only those interventions where the woman self-manages some or all aspects of such care were considered as self-care. In studies, these interventions were usually compared against standard clinical care i.e. care delivered by physicians, nurses or community health workers.

> Example: Misoprostol for postpartum haemorrhage (PPH) prevention
>
> The administration of misoprostol by HWs (whether in facility or community settings) for PPH prevention is received by the pregnant woman - she does not self-administer. Therefore, it is not a self-care intervention, even if misoprostol administration takes place in the context of birth at home or in the community.
>
> Conversely, advanced distribution of misoprostol during antenatal visits to be used after childbirth, often accompanied by brief advice on how and when to use misoprostol safely, is considered a self-care intervention. This is because the intervention aims to enable the mother or her partners to self-administer misoprostol at home, when a skilled birth attendant, CHW or lay health worker is not available.

(c) Studies that investigated pharmacokinetics, efficacy and safety of pharmacological agents were excluded because their findings did not allow distinguishing between self-medication and being prescribed the medication by health providers. On the other hand, interventions aimed at promoting self-management or improving adherence to multi-dose therapies at home were eligible.

For peer review only - http://bmjopen.bmj.com/site/about/guidelines.xhtml

> Example: Prophylactic agents for malaria and HIV prevention
>
> Studies involving self-administration of IPTp and daily oral HIV PrEP at home (as opposed to supervised administration at antenatal clinics) are eligible. However, trials investigating pharmacokinetics, efficacy and safety of individual agents (sulfadoxine-pyrimethamine for malaria, tenofovir/emtricitabine for HIV) in pregnant and postpartum cohorts did not sufficiently show that these agents can be effectively implemented in a self-care approach, and were thus excluded.

3. Interventions beyond the pregnancy period

(a) Preconception studies

In our review, preconception interventions were eligible only if they were designed to improve pregnancy outcomes and reduce risks of maternal or perinatal complications. Interventions to improve the chance of successful conception (e.g. in couples undertaking *in vitro* fertilisation therapy) were excluded.

Interventions to manage existing medical conditions were excluded unless they were framed in the context of preparation for pregnancy and evaluated pregnancy-related outcomes.

> Example: Type 1 diabetes mellitus (T1DM)
>
> Uncontrolled glucose levels during pregnancy may increase risks of miscarriage or birth defects. Therefore, interventions aimed at achieving glycaemic control in women with T1DM who wish to get pregnant were eligible. However, general studies of self-care interventions among women with T1DM (not otherwise specified) were excluded.

(b) Postpartum studies

The postpartum period is defined as the first 42 days after birth. Interventions initiated outside this time frame were excluded.

Interventions designed to facilitate newborn care (i.e. infant nutrition, postnatal infant monitoring, kangaroo care, etc.) and breastfeeding (i.e. measures to increase lactation, breastfeeding techniques, etc.) were excluded from our review as their broad scope warrants a separate focused review.

4. Role of digital health

Digital interventions were possibly considered self-care interventions as they can provide platforms for the woman to seek information, communicate with health providers and access external support, ultimately creating a supporting environment for self-care. However, use of digital communication alone does not determine whether an intervention is self-care intervention or not, but rather what practice is facilitated by its use.

(a) Client-to-client technology

- Digital platforms to connect the pregnant or postpartum women to support groups (e.g. trained volunteers, other mothers) were considered self-care interventions. The underlying assumption is that when the women use these platforms, she is actively seeking information and support from sources outside the health system.

(b) Client-to-provider technology

- The use of digital trackers to enable the woman to take appropriate actions for self-management of her health conditions (e.g. taking her own physical measurements or self-adjusting medications based on these measurements) is a self-care intervention.

For peer review only - http://bmjopen.bmj.com/site/about/guidelines.xhtml

- If the digital trackers feed information directly to the clinician for decision-making, without involving the patient in the process, it is not a self-care intervention.

> Example: Blood glucose monitoring
>
> Interventions where women check their own blood glucose levels using a glucometer were self-care interventions.
>
> Interventions using automatic monitoring systems (i.e. sensors that automatically measure glucose levels in interstitial fluid and transmit readings to mobile devices) were evaluated on a case-by-case basis. If the woman was taught to adjust her lifestyle, calibrate her medications or notify her clinician of hypoglycaemic episodes, the intervention is a self-care intervention. If the data was masked to the participants during the study duration, the study was excluded.
>
> Interventions where blood glucose levels were tested by a health worker in inpatient or outpatient settings were not self-care interventions.

(c) Provider-to-client technology

- The use of mHealth/eHealth to provide self-help information, instructions or reminders to women to encourage adoption of a healthy lifestyle (or otherwise change behaviour) is a self-care intervention.
- The use of telehealth for virtual consults to replace face-to-face antenatal or postnatal visits is not a self-care intervention, because virtual delivery only replaces the mode of communication between the patient and the clinician, but still requires a health worker to provide effective care.

5. Mixed intervention

If an intervention consists of a mix of self-care and non-self-care interventions, the programme was evaluated on a case-by-case basis. The factors considered to judge eligibility are:

- Whether self-care components constitute a majority of the intervention
- Whether the intervention as a whole places an emphasis on self-care or explicitly promotes self-care
- Whether the intervention represents task-sharing certain aspects of clinical care to the women or their family members

For peer review only - http://bmjopen.bmj.com/site/about/guidelines.xhtml

Supplementary File S5. Data collection form

	Field	Format	Option/Example
**General characteristics**	Study ID	Free text	e.g. McManus_2018
	Reviewer's initial	Free text	
	Title	Free text	
	Trial register number (if any)	Free text	
**Linking**	ID of other reports from the same studies	Free text	
	Ongoing study	Y/N	Yes if the protocol was published as a journal article from 2019 onwards and trial register records did not indicate study completion
**Methods**	Study design	Multiple choice	O Meta-analysis and network meta-analysis<br>O Systematic review without meta-analysis<br>O Scoping review<br>O Overview of reviews<br>O Randomised controlled trial<br>O Non-randomised interventional study (including pretest-posttest, quasi-randomised trial, etc.)<br>O Retrospective & prospective observational cohort study<br>O Cross-sectional study<br>O Diagnostic test accuracy study
	Country	Free text	N/A for reviews and meta-analyses
**Participants**	<18 years of age	Y/N	Yes if the study inclusion criteria specified a minimum age <18 or adolescent girls

For peer review only - http://bmjopen.bmj.com/site/about/guidelines.xhtml

	Field	Format	Option/Example
	High-risk pregnancies	Y/N	Yes if the study inclusion criteria included: age >35 years old, presence of risk behaviours (substance use, smoking), obesity or overweight, pre-existing or pregnancy-induced chronic conditions (diabetes mellitus, hypertension, thyroid disease, or other co-morbidities), history of pregnancy complications or pregnancy loss, HIV-positive status, and multiple pregnancies
**Interventions**	Self-care activities	List any that is applicable	Refer to Supplementary File S10 for the full list of self-care activities
	Modes of access to and delivery of self-care interventions	List any that is applicable	▪ Health facilities e.g. self-diagnostic tests provided and taken in facilities, and information on and demonstration of self-care interventions provided in health facilities<br>▪ Community and non-facility-based healthcare e.g. products and services delivered to home by community health workers; activities taking place at community centres; traditional, complementary or alternative medicines<br>▪ Peer and/or partner counselling and support<br>▪ Over-the-counter availability of drugs and therapeutics, medical devices and diagnostics through pharmacies and drug stores<br>▪ Digital technologies and platforms e.g. telemedicine, individuals tracking their personal health data, eHealth including health information and peer support, ordering tests or drugs online<br>▪ Healthcare during emergency and humanitarian responses e.g. in shelters and refugee camps and settlements<br>▪ Home-based care i.e. direct access to health products, tools and information at home<br>▪ School: education, support and counselling taking place at school<br>▪ Workplace: health, safety and well-being interventions provided within the workplace, such as first aid andinterventions to support mental health and avoid burnout
	Time of intervention	List any that is applicable	▪ Preconception<br>▪ Antenatal<br>▪ Intrapartum<br>▪ Postpartum: Up to 42 days postpartum

For peer review only

	Field	Format	Option/Example
			▪ N/A for reviews and meta-analyses
	Any external support in the uptake or implementation of self-care	List any that is applicable	▪ None<br>▪ Family: partner & immediate family<br>▪ Community members: non-familial, non-medically trained community members, including peer support groups, friends, social leaders<br>▪ CHWs: informally-trained health workers such as traditional birth attendants or doulas<br>▪ HCWs: doctors, nurses, midwives, allied health practitioners, therapists and research students involved in delivering care.<br>HCWs that were only involved in facilitating study conduct (i.e. recruiting, explaining the study procedures, training participants on using the self-care tools etc.), without delivering actual medical care, were not considered as having supported delivery of the intervention.<br>▪ N/A for reviews and meta-analyses
**Outcomes**	Maternal mortality	Y/N	Maternal death
	Perinatal mortality	Y/N	Miscarriage, stillbirth, neonatal death
	Neonatal outcomes	Y/N	Birth weight, Apgar score, preterm birth, birth injuries, gestational age at delivery & other neonatal complications/outcomes, etc.
	Labour outcomes	Y/N	Duration of labour, labour pain, onset of labour (spontaneous, induced or pre-labour C-section); mode of delivery (spontaneous vaginal, instrumental – forceps/vacuum, C-section planned vs unplanned), etc.
	Anthropometric & cardiometabolic outcomes	Y/N	Maternal weight & BMI, lipid profile, inflammatory biomarkers, etc.
	Diabetes & other metabolic disorders	Y/N	GDM/T2DM diagnosis, various glycaemic biomarkers & serum indicators, etc.
	Hypertensive disorders of pregnancy	Y/N	Preeclampsia, eclampsia, gestational/postpartum hypertension, HELLP syndrome, blood pressure measurements etc.
	Postpartum infection & haemorrhage	Y/N	Postpartum haemorrhage, amount of blood loss, puerperal sepsis, endometritis, surgical site infection, etc.

For peer review only

	Field	Format	Option/Example
	Urogenital outcomes	Y/N	Urinary incontinence, urinary tract infections, pelvic floor disorders, postpartum perineal issues etc.
	Nutritional deficiencies	Y/N	Any nutritional deficiencies or serum levels of micronutrients, iron-deficiency anaemia, etc.
	Gastrointestinal outcomes	Y/N	Nausea & vomiting, constipation, etc.
	Other maternal complications	Y/N	Renal dysfunction, liver dysfunction & any other maternal complications not elsewhere classified
	Infectious diseases	Y/N	Malaria, HIV, STI, etc.
	Other minor physiological discomforts	Y/N	Breast & nipple issues, lumbopelvic pain & other minor ailments not elsewhere classified
	Mental wellbeing & quality of life	Y/N	Depression, anxiety, sexual functioning, childbirth fear/readiness
	Behavioural change	Y/N	Smoking cessation, alcohol use reduction, contraceptive use, supplement intake, physical activity & any other health-promoting or risk-avoiding behaviours
	Breastfeeding*	Y/N	Breastfeeding patterns (exclusive/full/partial), duration, initiation or continuation
	Other	Y/N	Diagnostic performance of tests, accordance with gold standards, intimate partner violence
**Self-care category (based on WHO classification [1])**	Individual Agency	List any that is applicable	▪ Interventions to promote awareness about self-care<br>▪ Interventions to promote autonomy and/or confidence to engage in self-care<br>▪ Interventions for improving self-care capacities and capabilities<br>▪ Interventions for improving health and digital literacy<br>▪ Interventions to promote sustained adoption of self-care practices and behaviours
	Health information-seeking	List any that is applicable	▪ Acquiring health education for informed health decision-making<br>▪ Improving health literacy to support health decision-making<br>▪ Individuals seeking health information via on-demand information services<br>▪ Individuals health decision-making support via online or mobile tools
	Social and community support	List any that is applicable	▪ Peer mentorship and counselling<br>▪ Peer-to-peer action to support access to and the uptake and use of self-care interventions<br>▪ Individuals sharing health data with peers

For peer review only - http://bmjopen.bmj.com/site/about/guidelines.xhtml

	Field	Format	Option/Example
	Personal health tracking	List any that is applicable	▪ Home-based record for health and diagnostic data<br>▪ Self-monitoring of health<br>▪ Active data capture/documentation by self-care user<br>▪ Passive data capture/documentation by device<br>▪ Use of diagnostic device at community location for<br>▪ self-measuring health indicator<br>▪ Tracking rational and responsible self-use of medicines and therapeutics
	Self-diagnosis of health conditions	List any that is applicable	▪ Self-testing, including to inform prevention<br>▪ Self-examining for health conditions<br>▪ Self-collection of samples to send for external testing<br>▪ Using online symptom checkers and health information
	Self-management of health	List any that is applicable	▪ Self-care for long-term health conditions<br>▪ Self-care for short-term health conditions<br>▪ Self-medication/treatment without medical prescription including for side effects<br>▪ Self-regulation of health conditions<br>▪ Self-care prevention including risk avoidance and support for physical and mental health and well-being<br>▪ Health management performed by caregivers<br>▪ Individuals procurement of safe over-the-counter medicines and products for self-care
	Individuals linkage to health system	List any that is applicable	▪ Individuals-initiated communication with health system<br>▪ Individuals sharing health data with health workers<br>▪ Individuals identifying location of health facilities/structures<br>▪ Individuals receiving feedback from health workers
	Individuals financial transactions for health	-	Not eligible under this review

**Although breastfeeding interventions were not eligible for inclusion, breastfeeding outcomes were recorded as they were often evaluated in interventions targeting other health issues, such as smoking cessation, diabetes management, breast & nipple care, etc.*

[1] World Health Organization. Classification of self-care interventions for health: a shared language to describe the uses of self-care interventions. Geneva, 2021 https://www.who.int/publications/i/item/9789240039469 (accessed April 6, 2022).

For peer review only - http://bmjopen.bmj.com/site/about/guidelines.xhtml

Supplementary File S6. List of excluded full texts

Author/Year	Title	Reasons for exclusion
Abbaspoor_2020	The effect of education through mobile phone short message service on promoting self-care in pre-diabetic pregnant women: A randomized controlled trial	Comparison - no appropriate control
Akanbi_2005	Effect of self-medication with antimalarial drugs on malaria infection in pregnant women in south-western Nigeria	Comparison - no appropriate control
Alemi_1996	Electronic self-help and support groups	Comparison - no appropriate control
Barbato_2014	Web-Based Treatment for Women With Stress Urinary Incontinence	Comparison - no appropriate control
Bradizza_2017	Smoking Cessation for Pregnant Smokers: Development and Pilot Test of an Emotion Regulation Treatment Supplement to Standard Smoking Cessation for Negative Affect Smokers	Comparison - no appropriate control
Burgess_2021	Feasibility of Using Blood Pressure Self-Monitoring and the Epic MyChart Blood Pressure Flowsheet to Monitor Blood Pressure after Preeclampsia	Comparison - no appropriate control
Bussiere-Cote_2019	Twice-Daily vs 4-Times-Daily Glucose Testing in Women With Gestational Diabetes Mellitus: a Pilot Study	Comparison - no appropriate control
Cao_2012	Comprehensive intensive therapy for Chinese gestational diabetes benefits both newborns and mothers	Comparison - no appropriate control
Chi_2016	Randomized comparison of group versus individual educational interventions for pregnant women to reduce their secondhand smoke exposure	Comparison - no appropriate control
Chung_2009	Validation and compliance of a home monitoring device in pregnancy: Microlife WatchBP home	Comparison - no appropriate control
Clapp_1989	The effects of maternal exercise on early pregnancy outcome	Comparison - no appropriate control
Cuneo_2017	Heart sounds at home: feasibility of an ambulatory fetal heart rhythm surveillance program for anti-SSA-positive pregnancies	Comparison - no appropriate control
Darvall_2020	A Pedometer-Guided Physical Activity Intervention for Obese Pregnant Women (the Fit MUM Study): randomized Feasibility Study	Comparison - no appropriate control
Dettinger_2019	PrEP Implementation for Mothers in Antenatal Care (PrIMA): Study protocol of a cluster randomised trial	Comparison - no appropriate control
Dyson_1998	Monitoring women at risk for preterm labor	Comparison - no appropriate control
Ganapathy_2016	Remote monitoring of blood pressure to reduce the risk of preeclampsia related complications with an innovative use of mobile technology	Comparison - no appropriate control
Gao_2012	Effects of an interpersonal-psychotherapy-oriented childbirth education programme for Chinese first-time childbearing women at 3-month follow up: randomised controlled trial	Comparison - no appropriate control
Gao_2021	An psychoeducation programme based on self-efficacy theory to improve experience of natural birth for primigravid women: Study protocol for a randomised controlled trial	Comparison - no appropriate control
Ghasemi_2021	Comparing the effect of individual counseling with counseling on social application on self-care and quality of life of women with gestational diabetes	Comparison - no appropriate control
Heuvel_2019	SAFE@HOME - Feasibility study of a telemonitoring platform combining blood pressure and preeclampsia symptoms in pregnancy care	Comparison - no appropriate control
Heuvel_2019	Validation of the iHealth Track and Omron HEM-9210T automated blood pressure devices for use in pregnancy	Comparison - no appropriate control
Hoppe_2016	An evaluation of diabetes targeted apps for Android smartphone in relation to behaviour change techniques	Comparison - no appropriate control
Humphries_2021	Decision Analysis in SHared decision making for Thromboprophylaxis during Pregnancy (DASH-TOP): a sequential explanatory mixed methods pilot study protocol	Comparison - no appropriate control
Iwama_2016	Maternal clinic and home blood pressure measurements during pregnancy and infant birth weight: The BOSHI study	Comparison - no appropriate control
Jiao_2019	Web-based versus home-based postnatal psychoeducational interventions for first-time mothers: A randomised controlled trial	Comparison - no appropriate control
Johnson_2018	Impacting diabetes self-management in women with gestational diabetes mellitus using short messaging reminders	Comparison - no appropriate control
Kerner_2004	Maternal self-administered fetal heart rate monitoring and transmission from home in high-risk pregnancies	Comparison - no appropriate control
Kominiarek_2019	A feasibility study of activity tracking devices in pregnancy	Comparison - no appropriate control
Lan_2017	Home blood pressure measurement in women with pregnancy-related hypertensive disorders	Comparison - no appropriate control
Lihme_2017	Self-monitoring of blood pressure during pregnancy: An observational study in the obstetrical waiting room	Comparison - no appropriate control
Limruangrong_2011	Effectiveness of a self-regulation program on diet control, exercise, and two-hour postprandial blood glucose levels in Thais with gestational diabetes mellitus	Comparison - no appropriate control
Malm_2014	Women's experiences of two different self-assessment methods for monitoring fetal movements in full-term pregnancy - a crossover trial	Comparison - no appropriate control
Manfredi_2011	Dissemination strategies to improve implementation of the PHS smoking cessation guideline in MCH public health clinics: experimental evaluation results and contextual factors	Comparison - no appropriate control

For peer review only - http://bmjopen.bmj.com/site/about/guidelines.xhtml

Author/Year	Title	Reasons for exclusion
Mantzana_2014	Applicability of self-obtained urine and vaginal samples for HPV-16, -18, -31 and -45 cervical cancer screening in pregnancy: A pilot cross-sectional study	Comparison - no appropriate control
Mendez-Figueroa_2017	Gestational Diabetes Mellitus and Frequency of Blood Glucose Monitoring: A Randomized Controlled Trial	Comparison - no appropriate control
Mikami_2017	Provisional criteria for the diagnosis of hypertension in pregnancy using home blood pressure measurements	Comparison - no appropriate control
Navaee_2020	Effect of pre-cesarean foot reflexology massage on anxiety of primiparous women	Comparison - no appropriate control
Pauley_2018	Gestational Weight Gain Intervention Impacts Determinants of Healthy Eating and Exercise in Overweight/Obese Pregnant Women	Comparison - no appropriate control
Pyra_2018	Tenofovir and tenofovir-diphosphate concentrations during pregnancy among HIV-uninfected women using oral preexposure prophylaxis	Comparison - no appropriate control
Raynes-Greenow_2009	Do audio-guided decision aids improve outcomes? A randomized controlled trial of an audio-guided decision aid compared with a booklet decision aid for Australian women considering labour analgesia	Comparison - no appropriate control
Roske_2008	Postpartum smoking cessation and relapse prevention intervention: A structural equation modeling application to behavioral and non-behavioral outcomes of a randomized controlled trial	Comparison - no appropriate control
Saffari_2020	Effect of a health-education program using motivational interviewing on oral health behavior and self-efficacy in pregnant women: a randomized controlled trial	Comparison - no appropriate control
Salomon_2004	Fetal home monitoring for the prenatal management of gastroschisis	Comparison - no appropriate control
Shieh_2017	Feasibility and Potential Benefits of a Self-Monitoring Enhanced Lifestyle Intervention to Prevent Excessive Gestational Weight Gain in Women Who Are Overweight or Obese	Comparison - no appropriate control
Smith_2014	Advance distribution of misoprostol for prevention of postpartum hemorrhage (PPH) at home births in two districts of Liberia	Comparison - no appropriate control
Svensson_2009	Randomised-controlled trial of two antenatal education programmes	Comparison - no appropriate control
Takeuchi_2016	Randomised controlled trial using smartphone website vs leaflet to support antenatal perineal massage practice for pregnant women	Comparison - no appropriate control
Tremonti_2017	Reliability of home blood pressure monitoring devices in pregnancy	Comparison - no appropriate control
Tucker_2018	How do home and clinic blood pressure readings compare in pregnancy? A systematic review and individual patient data meta-analysis	Comparison - no appropriate control
Twedt_2015	Sleep Duration and Blood Glucose Control in Women With Gestational Diabetes Mellitus	Comparison - no appropriate control
Ussher_2017	The London Exercise And Pregnant smokers (LEAP) trial: a randomised controlled trial of physical activity for smoking cessation in pregnancy with an economic evaluation	Comparison - no appropriate control
Vakilian_2018	Controlled breathing with or without lavender aromatherapy for labor pain at the first stage: a randomized clinical trial	Comparison - no appropriate control
Valbo_1991	Smoking cessation in pregnancy Mode of intervention and effect	Comparison - no appropriate control
Valiani_2014	Comparison of childbirth training workshop effects on knowledge, attitude, and delivery method between mothers and couples groups referring to Isfahan health centers in Iran	Comparison - no appropriate control
Vallely_2016	Feasibility and acceptability of clean birth kits containing misoprostol for self-administration to prevent postpartum hemorrhage in rural Papua New Guinea	Comparison - no appropriate control
Waugh_2003	Patient initiated home blood pressure recordings are accurate in hypertensive pregnant women	Comparison - no appropriate control
Wu_2021	Application of diversified and quantitative management model of exercise intervention in patients with gestational diabetes mellitus	Comparison - no appropriate control
Young_2000	The effectiveness of weekly iron supplementation in pregnant women of rural northern Malawi	Comparison - no appropriate control
Chen_2020	Non-pharmacological interventions for postpartum depression: A protocol for systematic review and network meta-analysis	Design - not a systematic review
Chow_2021	Appraisal of systematic reviews on interventions for postpartum depression: systematic review	Design - not a systematic review
Cottrell_2010	An updated review of of evidence to discourage douching	Design - not a systematic review
Davies_2018	Global and national guidance for the use of pre-exposure prophylaxis during peri-conception, pregnancy and breastfeeding	Design - not a systematic review
Dennis_2014	Psychosocial interventions for the treatment of perinatal depression	Design - not a systematic review
Devoe_2000	Home uterine activity monitoring: A critical review	Design - not a systematic review
Dominguez-Solis_2021	Non-pharmacological interventions to reduce anxiety in pregnancy, labour and postpartum: A systematic review	Design - not a systematic review
Edmonds_2014	Shared decision-making and decision support: Their role in obstetrics and gynecology	Design - not a systematic review
Evans_2018	Systematic review and meta-analysis of non-pharmacological interventions to reduce the symptoms of mild to moderate anxiety in pregnant women	Design - not a systematic review

For peer review only - http://bmjopen.bmj.com/site/about/guidelines.xhtml

Author/Year	Title	Reasons for exclusion
Feig_2018	Continuous glucose monitoring in pregnant women with Type 1 diabetes: benefits for mothers, using pumps or pens, and their babies	Design - not a systematic review
Flash_2017	Pre-exposure prophylaxis for HIV prevention in women: Current perspectives	Design - not a systematic review
Grange_2020	Smoking and smoking cessation in pregnancy Synthesis of a systematic review	Design - not a systematic review
Greaves_2019	Tailored Intervention for Smoking Reduction and Cessation for Young and Socially Disadvantaged Women During Pregnancy	Design - not a systematic review
Horgan_2019	Pre-exposure prophylaxis for HIV prevention during pregnancy and lactation: forget not the women and children	Design - not a systematic review
Hoyme_2010	Prevention of Preterm Birth Is Possible by Vaginal pH Screening, Early Diagnosis of Bacterial Vaginosis or Abnormal Vaginal Flora and Treatment	Design - not a systematic review
Joubert_2012	Personal continuous glucose monitoring (CGM) in diabetes management: Review of the literature and implementation for practical use	Design - not a systematic review
Jovanovic_2008	Using meal-based self-monitoring of blood glucose as a tool to improve outcomes in pregnancy complicated by diabetes	Design - not a systematic review
Koukoulithras_2021	The Effectiveness of Non-Pharmaceutical Interventions Upon Pregnancy-Related Low Back Pain: A Systematic Review and Meta-Analysis	Design - not a systematic review
Lambermon_2020	Maternal self-care in the early postpartum period: An integrative review	Design - not a systematic review
Melvin_2000	Recommended cessation counselling for pregnant women who smoke: A review of the evidence	Design - not a systematic review
Menendez_2007	Reducing the burden of malaria in pregnancy by preventive strategies	Design - not a systematic review
Montag_2012	A review of evidence-based approaches for reduction of alcohol consumption in Native women who are pregnant or of reproductive age	Design - not a systematic review
Moradi_2020	COVID-19 and self-care strategies for women with gestational diabetes mellitus	Design - not a systematic review
Nosova_2020	Leveraging technology for the treatment of type 1 diabetes in pregnancy: A review of past, current, and future therapeutic tools	Design - not a systematic review
Rodbard_2017	Continuous glucose monitoring: A review of recent studies demonstrating improved glycemic outcomes	Design - not a systematic review
Sachs_1991	Home monitoring of uterine activity Does it prevent prematurity?	Design - not a systematic review
Siebern_2012	Non-Pharmacological Treatment of Insomnia	Design - not a systematic review
Stalter_2021	Safety review of tenofovir disoproxil fumarate/emtricitabine pre-exposure prophylaxis for pregnant women at risk of HIV infection	Design - not a systematic review
Zinsser_2020	Changing behaviour in pregnant women: A scoping review	Design - not a systematic review
Cairns_2020	Implementing self-management: A mixed methods study of women's experiences of a postpartum hypertension intervention (SNAP-HT)	Design - qualitative study
Chiarelli_2003	Acceptability of a urinary continence promotion programme to women in postpartum	Design - qualitative study
Gosha_1986	A self-help group for new mothers: an evaluation	Design - qualitative study
Gueguen_2021	Hypnosis for labour and childbirth: A meta-integration of qualitative and quantitative studies	Design - qualitative study
Jidong_2021	Psychological interventions for maternal depression among women of African and Caribbean origin: a systematic review	Design - qualitative study
Labrecque_2001	Women's views on the practice of prenatal perineal massage	Design - qualitative study
Lee_2008	A pilot intervention for pregnant women in Sichuan, China on passive smoking	Design - qualitative study
McCaffrey_2020	The role and outcomes of music listening for women in childbirth: An integrative review	Design - qualitative study
Moulaei_2021	The Development and Usability Assessment of an mHealth Application to Encourage Self-Care in Pregnant Women against COVID-19	Design - qualitative study
Price_1991	Comparison of three antismoking interventions among pregnant women in an urban setting: A randomized trial	Design - qualitative study
Radestad_2021	Midwives' experiences of using the Mindfetalness method when talking with pregnant women about fetal movements	Design - qualitative study
Shorey_2019	A systematic mixed-studies review on mindfulness-based childbirth education programs and maternal outcomes	Design - qualitative study
Stonbraker_2020	Establishing content for a digital educational support group for new adolescent mothers in the Dominican Republic: A user-centered design approach	Design - qualitative study
Wallace_2009	A pregnancy wellness guide to enhance care through self-assessment, personal reflection, and self-referral	Design - qualitative study
Yoshida_2001	Postnatal depression in Japanese mothers and the reconsideration of 'Satogaeri bunben'	Design - qualitative study
Bivia-Roig_2020	Efficacy of an internet-based intervention to promote a healthy lifestyle on the reproductive parameters of overweight and obese women: study protocol for a randomised controlled trial	Intervention - conception/fertility
Boedt_2021	Systematic development of a mobile preconception lifestyle programme for couples undergoing IVF: the PreLiFe-programme	Intervention - conception/fertility

For peer review only - http://bmjopen.bmj.com/site/about/guidelines.xhtml

Author/Year	Title	Reasons for exclusion
Galletly_1996	Improved pregnancy rates for obese, infertile women following a group treatment program: An open pilot study	Intervention - conception/fertility
Kim_2020	Effectiveness of non-pharmacological interventions for overweight or obese infertile women: A systematic review and meta-analysis	Intervention - conception/fertility
Soltani_2015	Maternal obesity management using mobile technology: A feasibility study to evaluate a text messaging based complex intervention during pregnancy	Intervention - conception/fertility
Sridhar_2020	Non-pharmacological anxiety reduction with immersive virtual reality for first-trimester dilation and curettage: a pilot study	Intervention - conception/fertility
Taghavi_2021	Pharmacological and non?pharmacological strategies for obese women with subfertility	Intervention - conception/fertility
Ahn_2004	The Effects of a Home-Visiting Discharge Education on Maternal Self-esteem, Maternal Attachment, PostpartumDepression and Family Function in theMothers of NICU Infants	Intervention - newborn care
Da?li_2021	The effect of oxytocin massage and music on breast milk production and anxiety level of the mothers of premature infants who are in the neonatal intensive care unit: A self-controlled trial	Intervention - newborn care
Deave_2019	A study to evaluate the effectiveness of Best Beginnings' Baby Buddy phone app in England: a protocol paper	Intervention - newborn care
Dol_2021	Effectiveness of a Postpartum Text Message Program (Essential Coaching for Every Mother) on Maternal Psychosocial Outcomes: Protocol for a Randomized Controlled Trial	Intervention - newborn care
Jamalivand_2017	Comparing the effect of electronic software and training booklet on maternal self-confidence and awareness about newborn care: A randomized controlled clinical trial	Intervention - newborn care
Missler_2020	Effectiveness of a psycho-educational intervention for expecting parents to prevent postpartum parenting stress, depression and anxiety: a randomized controlled trial	Intervention - newborn care
Mohammadi_2018	The effect of stress inoculation training on breastfeeding self-efficacy and perceived stress of mothers with low birth weight infants: A clinical trial	Intervention - newborn care
Ngai_2009	The effects of a childbirth psychoeducation program on learned resourcefulness, maternal role competence and perinatal depression: a quasi-experiment	Intervention - newborn care
Ondersma_2018	Computer-delivered indirect screening and brief intervention for drug use in the perinatal period: A randomized trial	Intervention - newborn care
Shorey_2015	A randomized controlled trial of the effectiveness of a postnatal psychoeducation programme on self-efficacy, social support and postnatal depression among primiparas	Intervention - newborn care
Shorey_2017	Effectiveness of the 'Home-but not Alone' mobile health application educational programme on parental outcomes: a randomized controlled trial, study protocol	Intervention - newborn care
Allard_2015	Gestational Diabetes Mellitus Identification Based on Self-Monitoring of Blood Glucose	Intervention - no intervention identified
Barbosa_2016	Educational technologies to encourage (self) care in postpartum women	Intervention - no intervention identified
Barrera_2015	Online prevention of postpartum depression for Spanish- and English-speaking pregnant women: a pilot randomized controlled trial	Intervention - no intervention identified
Bessinger_2002	Substrate utilization and hormonal responses to moderate intensity exercise during pregnancy and after delivery	Intervention - no intervention identified
Black-Olien_1993	The effects of prenatal programs on postpartum emotional well-being	Intervention - no intervention identified
Carlson_2018	Antepartum Care of Women Who Are Obese During Pregnancy: Systematic Review of the Current Evidence	Intervention - no intervention identified
Chung_2020	Medical help-seeking strategies for perinatal women with obstetric and mental health problems and changes in medical decision making based on online health information: Path analysis	Intervention - no intervention identified
Clapp_2008	Long-term outcome after exercising throughout pregnancy: fitness and cardiovascular risk	Intervention - no intervention identified
Cosson_2017	Poor reliability and poor adherence to self-monitoring of blood glucose are common in women with gestational diabetes mellitus and may be associated with poor pregnancy outcomes	Intervention - no intervention identified
Dennis_2007	Psychosocial and psychological interventions for treating antenatal depression	Intervention - no intervention identified
Dennis-Tiwary_2017	Salutary effects of an attention bias modification mobile application on biobehavioral measures of stress and anxiety during pregnancy	Intervention - no intervention identified
Dol_2020	Impact of mobile health interventions during the perinatal period on maternal psychosocial outcomes: A systematic review	Intervention - no intervention identified
Ekelof_2021	Depressive symptoms postpartum is associated with physical activity level the year prior to giving birth - A retrospective observational study	Intervention - no intervention identified
Feng_2021	Psychological or educational eHealth interventions on depression, anxiety or stress following preterm birth: a systematic review	Intervention - no intervention identified
Gbagbo_2020	Implications of self-medication in pregnancy for Safe Motherhood and Sustainable Development Goal-3 in selected Ghanaian communities	Intervention - no intervention identified
Gribel_2020	Influence of non-pharmacological obstetric interventions on adverse outcomes of childbirth under regional analgesia	Intervention - no intervention identified
Hall_2011	The use of complementary and alternative medicine by pregnant women: a literature review	Intervention - no intervention identified
Hewage_2020	Barriers to Gestational Diabetes Management and Preferred Interventions for Women With Gestational Diabetes in Singapore: Mixed Methods Study	Intervention - no intervention identified

For peer review only - http://bmjopen.bmj.com/site/about/guidelines.xhtml

Author/Year	Title	Reasons for exclusion
Hong_2021	Perspectives on antenatal education associated with pregnancy outcomes: Systematic review and meta-analysis	Intervention - no intervention identified
Hoyme_2020	Reduced incidence of early preterm birth in the State of Thuringia following an intravaginal pH-self-monitoring screening program	Intervention - no intervention identified
Igwesi-Chidobe_2021	Community-based non-pharmacological interventions for improving pain, disability and quality of life in pregnant women with musculoskeletal conditions: Protocol for a systematic review with meta-analyses	Intervention - no intervention identified
Iyawa_2021	Mobile apps for self-management in pregnancy: a systematic review	Intervention - no intervention identified
Jung_2021	Psychosocial support interventions for women with gestational diabetes mellitus: a systematic review	Intervention - no intervention identified
Kim_2017	Effectiveness of Psychosocial and Educational Prenatal and Postnatal Care Interventions for Married Immigrant Women in Korea: Systematic Review and Meta-analysis	Intervention - no intervention identified
Lau_2016	Efficacy of Internet-Based Self-Monitoring Interventions on Maternal and Neonatal Outcomes in Perinatal Diabetic Women: A Systematic Review and Meta-Analysis	Intervention - no intervention identified
Leis_2009	A systematic review of home-based interventions to prevent and treat postpartum depression	Intervention - no intervention identified
Leventhal_1989	Active coping reduces reports of pain from childbirth	Intervention - no intervention identified
Lin_2018	Effectiveness of self-help psychological interventions for treating and preventing postpartum depression: a meta-analysis	Intervention - no intervention identified
Mackiewicz-Seghete_2020	Protocol for a mechanistic study of mindfulness based cognitive therapy during pregnancy	Intervention - no intervention identified
Manandhar_2004	Effect of a participatory intervention with women's groups on birth outcomes in Nepal: cluster-randomised controlled trial	Intervention - no intervention identified
Martin_2017	Adherence-Specific Social Support Enhances Adherence to Calcium Supplementation Regimens among Pregnant Women	Intervention - no intervention identified
Masoumi_2018	Effect of a sex education program on females' sexual satisfaction during pregnancy: a randomized clinical trial	Intervention - no intervention identified
Mekonnen_2021	Adherence to iron with folic acid supplementation among pregnant women attending antenatal care in public health centers in simada district, northwest ethiopia: using health belief model perspective	Intervention - no intervention identified
Mertens_2019	Effect of Lifestyle Coaching Including Telemonitoring and Telecoaching on Gestational Weight Gain and Postnatal Weight Loss: A Systematic Review	Intervention - no intervention identified
Mohamadirizi_2018	The effect of religious-spiritual support on childbirth self-efficacy	Intervention - no intervention identified
Nadal_2021	Educational films for improving screening and self-management of gestational diabetes in India and Uganda (GUIDES): study protocol for a cluster-randomised controlled trial	Intervention - no intervention identified
Naughton_2015	Use and effectiveness of lapse prevention strategies among pregnant smokers	Intervention - no intervention identified
Navidian_2017	The effect of home-based supportive-educational counseling on primigravidas' postpartum stress	Intervention - no intervention identified
Nazik_2013	The Prevention And Reduction Of Postpartum Complications: Orem's Model	Intervention - no intervention identified
Newham_2012	State-trait anxiety inventory (STAI) scores during pregnancy following intervention with complementary therapies	Intervention - no intervention identified
Notley_2019	Development of a complex intervention for the maintenance of postpartum smoking abstinence: process for defining evidence-based intervention	Intervention - no intervention identified
O'Connell_2021	Interventions for fear of childbirth including tocophobia	Intervention - no intervention identified
Ogrodniczuk_2003	Preventing Postnatal Depression: a Review of Research Findings	Intervention - no intervention identified
Olander_2015	Person-centred care in interventions to limit weight gain in pregnant women with obesity - a systematic review	Intervention - no intervention identified
Olayiwola_2013	Living Smart, Living Fit: a patient-centered program to improve perinatal outcomes in a community health center population	Intervention - no intervention identified
Ouedraogo_2013	Maternal anemia in pregnancy: Assessing the effect of routine preventive measures in a malaria-endemic area	Intervention - no intervention identified
Ratnayake_2018	Validity of over the counter finger stick glucose measurement devices in comparison with laboratory venous plasma glucose measurements on pregnant women with diabetes	Intervention - no intervention identified
Sayakhot_2016	Use of a web-based educational intervention to improve knowledge of healthy diet and lifestyle in women with Gestational Diabetes Mellitus compared to standard clinic-based education	Intervention - no intervention identified
Silverstein_2011	Problem-solving education to prevent depression among low-income mothers of preterm infants: a randomized controlled pilot trial	Intervention - no intervention identified
Sinclair_2017	A systematic literature review of computer-based behavioural change interventions to inform the design of an online VBAC intervention for the OptiBIRTH European randomised trial (project HEALTH -- F3 -- 2012-305208)	Intervention - no intervention identified
Song_2015	A systematic review of psychosocial interventions for women with postpartum stress	Intervention - no intervention identified
Sun_2020	A mushroom diet reduced the risk of pregnancy-induced hyper tension and macrosomia: A randomized clinical trial	Intervention - no intervention identified

For peer review only - http://bmjopen.bmj.com/site/about/guidelines.xhtml

Author/Year	Title	Reasons for exclusion
Westerhoff_2019	About:blank? Online Interventions for Postpartum Depression	Intervention - no intervention identified
Windsor_2016	A Process Evaluation of the WV Smoking Cessation and Reduction in Pregnancy Treatment (SCRIPT) Dissemination Initiative: Assessing the Fidelity and Impact of Delivery for State-Wide, Home-Based Healthy Start Services	Intervention - no intervention identified
Wulp_2016	Correlates of partner support to abstain from prenatal alcohol use: a cross-sectional survey among Dutch partners of pregnant women	Intervention - no intervention identified
Wylie_2018	Evaluation of patient- versus provider-collected vaginal swabs for microbiome analysis during pregnancy	Intervention - no intervention identified
Abbas_2020	Testing a home-based model of care using misoprostol for prevention and treatment of postpartum hemorrhage: Results from a randomized placebo-controlled trial conducted in Badakhshan province, Afghanistan	Intervention - not self-care
Abdollahi_2020	Effect of Psychotherapy on Reduction of Fear of Childbirth and Pregnancy Stress: A Randomized Controlled Trial	Intervention - not self-care
Abuogi_2020	HIV status disclosure patterns and male partner reactions among pregnant women with HIV on lifelong ART in Western Kenya	Intervention - not self-care
Afandi_2019	The value of Continuous Glucose Monitoring and Self-Monitoring of Blood Glucose in patients with Gestational Diabetes Mellitus during Ramadan fasting	Intervention - not self-care
Agarwal_2012	Evaluation of isosorbide mononitrate for cervical ripening prior to induction of labor for postdated pregnancy in an outpatient setting	Intervention - not self-care
Ahmad_2021	The Knowledge of Danger Signs of Obstetric Complications among Women in Rural India: Evaluating an Integrated Microfinance and Health Literacy Program	Intervention - not self-care
Akbarzadeh_2015	Comparison of the Effects of Maternal Supportive Care and Acupressure (at BL32 Acupoint) on Labor Length and Infant's Apgar Score	Intervention - not self-care
Akpan_2018	Antenatal Deworming and Materno-Perinatal Outcomes in Calabar, Nigeria	Intervention - not self-care
Alfirevic_2020	Home versus inpatient induction of labour for improving birth outcomes	Intervention - not self-care
Alimoradi_2019	Comparing the effect of auricular acupressure and body acupressure on pain and duration of the first stage of labor: study protocol for a randomized controlled trial	Intervention - not self-care
Amanak_2019	The impact of prenatal education based on the Roy adaptation model on gestational hypertension, adaptation to pregnancy and pregnancy outcomes	Intervention - not self-care
Anderson_2021	Effects of a Home-Based Intervention on HIV Prevention Health Behaviors in Pregnant/Postpartum Kenyan Women: Estimating Moderating Effects of Depressive Symptoms	Intervention - not self-care
Aquino_2020	Exploring the Use of Telemonitoring for Patients at High Risk for Hypertensive Disorders of Pregnancy in the Antepartum and Postpartum Periods: scoping Review	Intervention - not self-care
Ayerle_2018	Effects of the birthing room environment on vaginal births and client-centred outcomes for women at term planning a vaginal birth: BE-UP, a multicentre randomised controlled trial	Intervention - not self-care
Bain_2014	Interventions for managing asthma in pregnancy	Intervention - not self-care
Baljon_2020	Effectiveness of breathing exercises, foot reflexology and back massage (BRM) on labour pain, anxiety, duration, satisfaction, stress hormones and newborn outcomes among primigravidae during the first stage of labour in Saudi Arabia: a study protocol for	Intervention - not self-care
Bar-Zeev_2019	Improving smoking cessation care in pregnancy at Aboriginal Medical Services: 'ICAN QUIT in Pregnancy' step-wedge cluster randomised study	Intervention - not self-care
Batool_2018	Comparison of the efficacy of IV iron versus oral iron therapy in Postpartum Anemia	Intervention - not self-care
Beckmann_1996	Accuracy of maternal perception of preterm uterine activity	Intervention - not self-care
Beiranvand_2014	The effects of religion and spirituality on postoperative pain, hemodynamic functioning and anxiety after cesarean section	Intervention - not self-care
Bhandal_2006	Intravenous versus oral iron therapy for postpartum anaemia	Intervention - not self-care
Birdsall_2002	Benefits of monitoring blood glucose by collection of blood on filter paper strips	Intervention - not self-care
Bishop_2016	Evaluating acupuncture and standard care for pregnant women with back pain (ease back): a pilot randomised trial	Intervention - not self-care
Bolinder_2016	Novel glucose-sensing technology and hypoglycaemia in type 1 diabetes: a multicentre, non-masked, randomised controlled trial	Intervention - not self-care
Bollapragada_2006	IMOP: Randomised placebo controlled trial of outpatient cervical ripening with isosorbide mononitrate (IMN) prior to induction of labour - Clinical trial with analyses of efficacy, cost effectiveness and acceptability	Intervention - not self-care
Bollapragada_2009	Randomized placebo-controlled trial of outpatient (at Home) cervical ripening with isosorbide mononitrate (IMN) prior to induction of labor-clinical trial with analyses of efficacy and acceptability: the IMOP study	Intervention - not self-care
Brown_1999	A randomized comparison of home uterine activity monitoring in the outpatient management of women treated for preterm labor	Intervention - not self-care
Bryce_2009	CATCH: development of a home-based midwifery intervention to support young pregnant smokers to quit	Intervention - not self-care
Bullarbo_2007	Outpatient vaginal administration of the nitric oxide donor isosorbide mononitrate for cervical ripening and labor induction postterm: a randomized controlled study	Intervention - not self-care
Bullock_2009	Baby BEEP: a randomized controlled trial of nurses' individualized social support for poor rural pregnant smokers	Intervention - not self-care
ButlerTobah_2019	Randomized comparison of a reduced-visit prenatal care model enhanced with remote monitoring	Intervention - not self-care
Carter_2020	Pilot Randomized Controlled Trial of Diabetes Group Prenatal Care	Intervention - not self-care

For peer review only - http://bmjopen.bmj.com/site/about/guidelines.xhtml

Author/Year	Title	Reasons for exclusion
Catling-Paull_2011	Non-clinical interventions that increase the uptake and success of vaginal birth after caesarean section: a systematic review	Intervention - not self-care
Chen_2003	Continuous glucose monitoring for the evaluation and improved control of gestational diabetes mellitus	Intervention - not self-care
Choko_2017	Investigating interventions to increase uptake of HIV testing and linkage into care or prevention for male partners of pregnant women in antenatal clinics in Blantyre, Malawi: Study protocol for a cluster randomised trial	Intervention - not self-care
Choko_2019	HIV self-testing alone or with additional interventions, including financial incentives, and linkage to care or prevention among male partners of antenatal care clinic attendees in Malawi: An adaptive multi-arm, multi-stage cluster randomised trial	Intervention - not self-care
Choko_2021	Partner-delivered HIV self-test kits with and without financial incentives in antenatal care and index patients with HIV in Malawi: a three-arm, cluster-randomised controlled trial	Intervention - not self-care
CHUMS-Group_1995	A multicenter randomized controlled trial of home uterine monitoring: active versus sham device The Collaborative Home Uterine Monitoring Study (CHUMS) Group	Intervention - not self-care
Chung_2012	Randomized non-invasive sham-controlled pilot trial of electroacupuncture for postpartum depression	Intervention - not self-care
Clarke_2020	OptiBIRTH: a cluster randomised trial of a complex intervention to increase vaginal birth after caesarean section	Intervention - not self-care
Colton_1995	A metaanalysis of home uterine activity monitoring	Intervention - not self-care
Corey_2019	Bedside Music Therapy for Women during Antepartum and Postpartum Hospitalization	Intervention - not self-care
Corna_2019	Supporting maternal mental health of Rohingya refugee women during the perinatal period to promote child health and wellbeing: A field study in Cox's Bazar	Intervention - not self-care
Corwin_1996	Multicenter randomized clinical trial of home uterine activity monitoring: Pregnancy outcomes for all women randomized	Intervention - not self-care
Cottrell_2006	Vaginal douching practices of women in eight Florida panhandle counties	Intervention - not self-care
Cummins_2016	Telephone Intervention for Pregnant Smokers: A Randomized Controlled Trial	Intervention - not self-care
Cypryk_2006	Evaluation of metabolic control in women with gestational diabetes mellitus by the continuous glucose monitoring system: a pilot study	Intervention - not self-care
Davey_2020	Emerging evidence from a systematic review of safety of pre-exposure prophylaxis for pregnant and postpartum women: where are we now and where are we heading?	Intervention - not self-care
Dellicour_2016	Effectiveness of the delivery of interventions to prevent malaria in pregnancy in Kenya	Intervention - not self-care
Desai_2018	Prevention versus treatment for malaria in pregnant women	Intervention - not self-care
deSousa_2019	Building trust to childbirth: Assessment of a nursing intervention programme	Intervention - not self-care
Dolatian_2010	The effect of reflexology on pain intensity of labor	Intervention - not self-care
Dowswell_2009	Transcutaneous electrical nerve stimulation (TENS) for pain management in labour	Intervention - not self-care
Doyle_2015	Home based educational intervention to improve perinatal outcomes for a disadvantaged community: a randomised control trial	Intervention - not self-care
El-Khouly_2017	Comparison of intravenous ferrous sucrose and oral ferrous sulphate in treatment of postpartum iron deficiency anemia	Intervention - not self-care
Evans_2021	A systematic review of supportive interventions to promote women's comfort and well-being during induction of labour	Intervention - not self-care
Facco_2019	Can We Use Home Sleep Testing for the Evaluation of Sleep Apnea in Obese Pregnant Women?	Intervention - not self-care
Fantinelli_2019	Assessment of psychological dimensions in telemedicine care for gestational diabetes mellitus: A systematic review of qualitative and quantitative studies	Intervention - not self-care
Fealy_2019	The Support for New Mums Project: A protocol for a pilot randomized controlled trial designed to test a postnatal psychoeducation smartphone application	Intervention - not self-care
Fenwick_2013	Study protocol for reducing childbirth fear: a midwife-led psycho-education intervention	Intervention - not self-care
Ferrara_2016	The Comparative Effectiveness of Diabetes Prevention Strategies to Reduce Postpartum Weight Retention in Women With Gestational Diabetes Mellitus: The Gestational Diabetes' Effects on Moms (GEM) Cluster Randomized Controlled Trial	Intervention - not self-care
Field_2013	Peer support and interpersonal psychotherapy groups experienced decreased prenatal depression, anxiety and cortisol	Intervention - not self-care
Firouzan_2020	The effect of midwifery led counseling based on Gamble's approach on childbirth fear and self-efficacy in nulligravida women	Intervention - not self-care
Ford_2001	Effects of a prenatal care intervention on the self-concept and self-efficacy of adolescent mothers	Intervention - not self-care
Ford_2002	Effects of a prenatal care intervention for adolescent mothers on birth weight, repeat pregnancy, and educational outcomes at one year postpartum	Intervention - not self-care
Foster_2016	Evaluating Acupuncture and Standard carE for pregnant women with Back pain (EASE Back): A feasibility study and pilot randomised trial	Intervention - not self-care
Fottrel_2013	The effect of increased coverage of participatory women's groups on neonatal mortality in Bangladesh: A cluster randomized trial	Intervention - not self-care
Fraser_1997	Randomized controlled trial of a prenatal vaginal birth after cesarean section education and support program Childbirth Alternatives Post-Cesarean Study Group	Intervention - not self-care
Garg_2015	A comparative study to evaluate the efficacy and safety of single dose intravenous iron carboxymaltose vs multidose iron sucrose in postpartum cases of severe iron deficiency anemia	Intervention - not self-care
Given_2013	Comparing patient-generated blood glucose diary records with meter memory in diabetes: a systematic review	Intervention - not self-care

For peer review only - http://bmjopen.bmj.com/site/about/guidelines.xhtml

Author/Year	Title	Reasons for exclusion
Given_2015	Tele-Mum: A Feasibility Study for a Randomized Controlled Trial Exploring the Potential for Telemedicine in the Diabetes Care of Those with Gestational Diabetes	Intervention - not self-care
Grady_2004	Pregnancy outcomes of adolescents enrolled in a CenteringPregnancy program	Intervention - not self-care
Green_1992	Telephone fetal heart rate monitoring in South Australia	Intervention - not self-care
Greve_2020	A pragmatic real-life study of flash glucose monitoring versus self-monitoring of blood glucose	Intervention - not self-care
Griffiths_2021	Interventions for preventing nausea and vomiting in women undergoing regional anaesthesia for caesarean section	Intervention - not self-care
Guittier_2009	Moxibustion for breech version: a randomized controlled trial	Intervention - not self-care
Guo_2019	Evaluating the effects of mobile health intervention on weight management, glycemic control and pregnancy outcomes in patients with gestational diabetes mellitus	Intervention - not self-care
Gutman_2020	A cluster randomized trial of delivery of intermittent preventive treatment of malaria in pregnancy at the community level in Burkina Faso	Intervention - not self-care
Gyorkos_2006	Lack of risk of adverse birth outcomes after deworming in pregnant women	Intervention - not self-care
Habib_2008	Outpatient cervical ripening with nitric oxide donor isosorbide mononitrate prior to induction of labor	Intervention - not self-care
Hajesmaeel-Gohari_2021	Virtual reality applications to assist pregnant women: a scoping review	Intervention - not self-care
Hanjani_2015	The Effect of Foot Reflexology on Anxiety, Pain, and Outcomes of the Labor in Primigravida Women	Intervention - not self-care
Haq_2005	Control of postpartum and post abortal haemorrhage with uterine packing	Intervention - not self-care
Heazell_2017	Can promoting awareness of fetal movements and focusing interventions reduce fetal mortality? A stepped-wedge cluster randomised trial (AFFIRM)	Intervention - not self-care
Hedderson_2018	A Tailored Letter Based on Electronic Health Record Data Improves Gestational Weight Gain Among Women With Gestational Diabetes Mellitus: The Gestational Diabetes' Effects on Moms (GEM) Cluster-Randomized Controlled Trial	Intervention - not self-care
Heuvel_2018	eHealth as the Next-Generation Perinatal Care: An Overview of the Literature	Intervention - not self-care
Hickling_1990	A non-pharmacological treatment of vascular headache during pregnancy	Intervention - not self-care
Holm_2019	Automated blood pressure self-measurement station compared to office blood pressure measurement for first trimester screening of pre-eclampsia	Intervention - not self-care
Holt_2017	Improving help-seeking for postnatal depression and anxiety: a cluster randomised controlled trial of motivational interviewing	Intervention - not self-care
Homko_2007	Use of an internet-based telemedicine system to manage underserved women with gestational diabetes mellitus	Intervention - not self-care
Homko_2012	Impact of a telemedicine system with automated reminders on outcomes in women with gestational diabetes mellitus	Intervention - not self-care
Horey_2004	Information for pregnant women about caesarean birth	Intervention - not self-care
Hughes_2018	Prediabetes in pregnancy, can early intervention improve outcomes? A feasibility study for a parallel randomised clinical trial	Intervention - not self-care
Ickovics_2011	Effects of group prenatal care on psychosocial risk in pregnancy: Results from a randomised controlled trial	Intervention - not self-care
Imhoff-Kunsch_2012	Antihelminthics in pregnancy and maternal, newborn and child health	Intervention - not self-care
Ivan_2015	Effect of Deworming on Disease Progression Markers in HIV-1-Infected Pregnant Women on Antiretroviral Therapy: A Longitudinal Observational Study From Rwanda	Intervention - not self-care
Izadirad_2018	Improving prenatal care in pregnant women in Iranshahr, Iran: Applying Health Belief Model	Intervention - not self-care
Jankowski_1987	Self-administered medications for obstetric patients	Intervention - not self-care
Jijimole_2018	Comparison of the effect of control, presence of a skilled birth attendant and reflexotherapy on labour outcomes in terms of pain, duration of labour and birth satisfaction among primigravid women-a pilot study	Intervention - not self-care
Johnsen_2018	Using eHealth to Increase Autonomy Supportive Care: a Multicenter Intervention Study in Antenatal Care	Intervention - not self-care
Johnson_2020	Computerized intervention for reducing intimate partner victimization for perinatal women seeking mental health treatment: A multisite randomized clinical trial protocol	Intervention - not self-care
Karaçam_2012	The Use of Perineal Massage in the Second Stage of Labor and Follow-Up of Postpartum Perineal Outcomes	Intervention - not self-care
Kellie_2020	Mechanical and surgical interventions for treating primary postpartum haemorrhage	Intervention - not self-care
Kelman_2018	A proof?of?concept pilot randomized comparative trial of brief Internet?based compassionate mind training and cognitive?behavioral therapy for perinatal and intending to become pregnant women	Intervention - not self-care
Kempe_1998	Home uterine activity monitoring in the prevention of very low birth weight reprinted from Public Health Reports, September/October 1997, Volume 112, published by the US Public Health Service	Intervention - not self-care
Kim_2019	Effects of a Web-Based Self-Management Program on the Behavior and Blood Glucose Levels of Women with Gestational Diabetes Mellitus	Intervention - not self-care
Krakowiak_2016	Home-Based HIV Testing Among Pregnant Couples Increases Partner Testing and Identification of Serodiscordant Partnerships	Intervention - not self-care
Kuo_2016	Auricular acupressure relieves anxiety and fatigue, and reduces cortisol levels in post-caesarean section women: a single-blind, randomised controlled study	Intervention - not self-care
Lane_2019	Real-Time Continuous Glucose Monitoring in Gestational Diabetes: A Randomized Controlled Trial	Intervention - not self-care
Larocque_2006	Should deworming be included in antenatal packages in hookworm-endemic areas of developing countries?	Intervention - not self-care
Larsen_2012	The Effectiveness of Childbirth Classes for Increasing Self-Efficacy in Women and Support Persons	Intervention - not self-care

For peer review only - http://bmjopen.bmj.com/site/about/guidelines.xhtml

Author/Year	Title	Reasons for exclusion
Lathrop_2014	A pilot study of prenatal care visits blended group and individual for women with low income	Intervention - not self-care
Li_2020	Internet-based intervention for postpartum depression in China ("Mommy go"): Protocol for a randomized controlled trial	Intervention - not self-care
Loayza_2011	Biofeedback for pain management during labour	Intervention - not self-care
Lumley_2006	Aiming to increase birth weight: a randomised trial of pre-pregnancy information, advice and counselling in inner-urban Melbourne	Intervention - not self-care
Luoba_2005	Earth-eating and reinfection with intestinal helminths among pregnant and lactating women in western Kenya	Intervention - not self-care
Ma_2011	Effects of Sanyinjiao (SP6) with electroacupuncture on labour pain in women during labour	Intervention - not self-care
Majewska_2021	Flash glucose monitoring in gestational diabetes mellitus: study protocol for a randomised controlled trial	Intervention - not self-care
Maloni_2010	Antepartum bed rest for pregnancy complications: Efficacy and safety for preventing preterm birth	Intervention - not self-care
Mark_2019	Male Partner Linkage to Clinic-Based Services for Sexually Transmitted Infections and Human Immunodeficiency Virus Services Following Couple Home-Based Education and Testing	Intervention - not self-care
Martin_2015	Providing Information and Support to Postnatal Women Who Have Experienced a Cesarean Section: A Pilot Study	Intervention - not self-care
Marwa_2019	The effects of HIV self-testing kits in increasing uptake of male partner testing among pregnant women attending antenatal clinics in Kenya: A randomized controlled trial	Intervention - not self-care
Mburu_2018	A model for predicting utilization of mHealth interventions in low-resource settings: case of maternal and newborn care in Kenya	Intervention - not self-care
McKellar_2017	Capture my mood: a feasibility study to develop a visual scale for women to self-monitor their mental wellbeing following birth	Intervention - not self-care
Meher_2005	Bed rest with or without hospitalisation for hypertension during pregnancy	Intervention - not self-care
Mendelson_2013	Impact of a preventive intervention for perinatal depression on mood regulation, social support, and coping	Intervention - not self-care
Mhajna_2020	Wireless, remote solution for home fetal and maternal heart rate monitoring	Intervention - not self-care
Milani_2015	The effects of postpartum home care on constipation and hemorrhoids at sixty days postpartum	Intervention - not self-care
Miller_2021	Reproducing fear: the effect of birth stories on nulligravid women's birth preferences	Intervention - not self-care
Mofid_2017	A Double-Blind Randomized Controlled Trial of Maternal Postpartum Deworming to Improve Infant Weight Gain in the Peruvian Amazon	Intervention - not self-care
Mohebbi_2019	Self-management intervention program based on the health belief model (Hbm) among women with gestational diabetes mellitus: A quazi-experimental study	Intervention - not self-care
Momenyan_2021	Immersive virtual reality analgesia in un-medicated laboring women (during stage 1 and 2): a randomized controlled trial	Intervention - not self-care
Monincx_2001	Maternal health, antenatal and at 8 weeks after delivery, in home versus in-hospital fetal monitoring in high-risk pregnancies	Intervention - not self-care
MooreSimas_2018	A Systematic Review of Integrated Care Interventions Addressing Perinatal Depression Care in Ambulatory Obstetric Care Settings	Intervention - not self-care
Morrison_2005	Women's health groups to improve perinatal care in rural Nepal	Intervention - not self-care
Mourad-Youssif_2010	Can the Non-pneumatic Anti-Shock Garment (NASG) reduce adverse maternal outcomes from postpartum hemorrhage? Evidence from Egypt and Nigeria	Intervention - not self-care
Msyamboza_2009	Community-based distribution of sulfadoxine-pyrimethamine for intermittent preventive treatment of malaria during pregnancy improved coverage but reduced antenatal attendance in southern Malawi	Intervention - not self-care
Mundell_2011	The impact of structured support groups for pregnant South African women recently diagnosed HIV positive	Intervention - not self-care
Nasso_2018	The Gestational Diabetes Group Program	Intervention - not self-care
Navaee_2015	Effect of role play education on primiparous women's fear of natural delivery and their decision on the mode of delivery	Intervention - not self-care
Ndibazza_2010	Effects of Deworming during Pregnancy on Maternal and Perinatal Outcomes in Entebbe, Uganda: A Randomized Controlled Trial	Intervention - not self-care
Ndibazza_2015	Effects of Deworming during Pregnancy on Maternal and Perinatal Outcomes in Entebbe, Uganda: A Randomized Controlled Trial	Intervention - not self-care
Nezamnia_2020	Effectiveness of cognitive-behavioral therapy on sexual function and sexual self-efficacy in pregnant women: An RCT	Intervention - not self-care
Norman_2018	Awareness of fetal movements and care package to reduce fetal mortality (AFFIRM): a stepped wedge, cluster-randomised trial	Intervention - not self-care
Ojengbede_2011	Assessing the role of the non-pneumatic anti-shock garment in reducing mortality from postpartum hemorrhage in Nigeria	Intervention - not self-care
Ondersma_2007	Computer-based brief intervention a randomized trial with postpartum women	Intervention - not self-care
Ondersma_2015	Computer-Delivered Screening and Brief Intervention forAlcohol Use in Pregnancy: A Pilot Randomized Trial	Intervention - not self-care
Orobaton_2016	Scaling-up the use of sulfadoxine-pyrimethamine for the preventive treatment of malaria in pregnancy: results and lessons on scalability, costs and programme impact from three local government areas in Sokoto State, Nigeria	Intervention - not self-care
Osoti_2015	Home-based HIV testing for men preferred over clinic-based testing by pregnant women and their male partners, a nested cross-sectional study	Intervention - not self-care
Osterman_2012	Effects of a motivational interviewing intervention to decrease prenatal alcohol use	Intervention - not self-care

For peer review only - http://bmjopen.bmj.com/site/about/guidelines.xhtml

Author/Year	Title	Reasons for exclusion
Palmer_2017	A retrospective cohort study of hospital versus home care for pregnant women with preterm prelabor rupture of membranes	Intervention - not self-care
Pantoja_2015	Oxytocin for preventing postpartum haemorrhage (PPH) in non-facility birth settings	Intervention - not self-care
Penn_2018	Non?pneumatic anti?shock garment (NASG) as a first aid for preventing or reversing hypovolemic shock secondary to obstetric hemorrhage	Intervention - not self-care
Perichart-Perera_2009	A medical nutrition therapy program improves perinatal outcomes in Mexican pregnant women with gestational diabetes and type 2 diabetes mellitus	Intervention - not self-care
Pikee_2021	New Innovation: Use of Flash Glucose Monitoring for Evaluating Glycaemic Variability, Patient Satisfaction and Clinical Utility in Pregnant Women with Diabetes	Intervention - not self-care
Raams_2018	Task shifting in active management of the third stage of labor: A systematic review	Intervention - not self-care
Redulla_2018	Bed rest with and without hospitalization in multiple pregnancy for improving perinatal outcomes	Intervention - not self-care
Reid_2002	A two-centred pragmatic randomised controlled trial of two interventions of postnatal support	Intervention - not self-care
Runjati_2020	The effect of postpartum coping skill classes (PCSC) on stress level, cortisol levels, maternal self-efficacy, and baby's growth and development In Semarang, Central Java	Intervention - not self-care
Ryo_2012	Fetal movement counting at home with a fetal movement acceleration measurement recorder: A preliminary report	Intervention - not self-care
Salam_2021	Effect of mass deworming with antihelminthics for soil?transmitted helminths during pregnancy	Intervention - not self-care
Sandeford_2021	Protocol for probiotic therapy vs placebo for preterm prelabour rupture of membranes to prolong pregnancy duration (Pro-PPROM) trial	Intervention - not self-care
Sathe_2016	Procedures and Uterine-Sparing Surgeries for Managing Postpartum Hemorrhage: A Systematic Review	Intervention - not self-care
Schachman_2004	Baby boot camp: facilitating maternal role adaptation among military wives	Intervention - not self-care
Scott_2018	Accuracy, User Acceptability, and Safety Evaluation for the FreeStyle Libre Flash Glucose Monitoring System When Used by Pregnant Women with Diabetes	Intervention - not self-care
Sekhavatpour_2020	The effect of spiritual self-care training on the quality of life of mothers of preterm infants: A randomized controlled trial	Intervention - not self-care
Senoun_2014	Planned home versus hospital care for preterm prelabour rupture of the membranes (PPROM) prior to 37 weeks' gestation	Intervention - not self-care
Shah_1993	Evaluation of the home-based maternal record: a WHO collaborative study	Intervention - not self-care
Shirvani_2021	Importance of childbirth preparation classes in improving childbirth self-efficacy and reducing worry in primigravidas	Intervention - not self-care
Shirzad_2020	Effect of "motivational interviewing" and "information, motivation, and behavioral skills" counseling interventions on choosing the mode of delivery in pregnant women: a study protocol for a randomized controlled trial	Intervention - not self-care
Snell_2006	An exploratory study in the UK of the effectiveness of three different pain management regimens for post-caesarean section women	Intervention - not self-care
Solhi_2019	Effect of Health Literacy Education on Self-Care in Pregnant Women: A Randomized Controlled Clinical Trial	Intervention - not self-care
Sosa_2001	Bed rest in singleton pregnancies for preventing preterm birth	Intervention - not self-care
Stamp_1995	Evaluation of antenatal and postnatal support to overcome postnatal depression: a randomized, controlled trial	Intervention - not self-care
Stock_2014	Home cervical ripening with dinoprostone gel in nulliparous women with singleton pregnancies	Intervention - not self-care
Stotts_2002	One-to-One A motivational intervention for resistant pregnant smokers	Intervention - not self-care
Stotts_2004	Impact of a motivational intervention on mechanisms of change in low-income pregnant smokers	Intervention - not self-care
Sultan_2019	Oral vs intravenous iron therapy for postpartum anemia: a systematic review and meta-analysis	Intervention - not self-care
Svahn-Ekdahl_2021	Maintenance of physical activity level, functioning and health after non-pharmacological treatment of pelvic girdle pain with either transcutaneous electrical nerve stimulation or acupuncture: A randomised controlled trial	Intervention - not self-care
Tandon_2014	Six-month outcomes from a randomized controlled trial to prevent perinatal depression in low-income home visiting clients	Intervention - not self-care
Tappin_2005	Randomised controlled trial of home based motivational interviewing by midwives to help pregnant smokers quit or cut down	Intervention - not self-care
Thayer_2017	Effects of deworming on child and maternal health: a literature review and meta-analysis	Intervention - not self-care
Thomas_2004	Effectiveness of a comprehensive psychoeducational intervention with pregnant and parenting adolescents: A pilot study	Intervention - not self-care
Thome_2013	Evaluation of a family nursing intervention for distressed pregnant women and their partners: A single group before and after study	Intervention - not self-care
Thomson_2016	Gestational Weight Gain: results from the Delta Healthy Sprouts Comparative Impact Trial	Intervention - not self-care
Toohill_2014	A randomized controlled trial of a psycho-education intervention by midwives in reducing childbirth fear in pregnant women	Intervention - not self-care
Torlesse_2001	Albendazole therapy and reduced decline in haemoglobin concentration during pregnancy (Sierra Leone)	Intervention - not self-care
Trevillion_2020	An exploratory parallel-group randomised controlled trial of antenatal Guided Self-Help (plus usual care) versus usual care alone for pregnant women with depression: DAWN trial	Intervention - not self-care
Triebwasser_2020	Successful implementation of text-based blood pressure monitoring for postpartum hypertension	Intervention - not self-care
Tripathy_2016	Effect of participatory women's groups facilitated by Accredited Social Health Activists on birth outcomes in rural eastern India: a cluster-randomised controlled trial	Intervention - not self-care

For peer review only - http://bmjopen.bmj.com/site/about/guidelines.xhtml

Author/Year	Title	Reasons for exclusion
Troy_2003	The effectiveness of a self-care intervention for the management of postpartum fatigue	Intervention - not self-care
Turan_2018	Development and Piloting of a Home-Based Couples Intervention During Pregnancy and Postpartum in Southwestern Kenya	Intervention - not self-care
Turawa_2020	Interventions for preventing postpartum constipation	Intervention - not self-care
Urquhart_2017	Home uterine monitoring for detecting preterm labour	Intervention - not self-care
van-der-Ploeg_1996	Transcutaneous nerve stimulation (TENS) during the first stage of labour: a randomized clinical trial	Intervention - not self-care
Varnfield_2021	MTHer, an mHealth System to Support Women with Gestational Diabetes Mellitus: Feasibility and Acceptability Study	Intervention - not self-care
Verma_2011	Intravenous iron therapy versus oral iron in postpartum patients in rural area	Intervention - not self-care
Voormolen_2018	Continuous glucose monitoring during diabetic pregnancy (GlucoMOMS): a multicentre randomized controlled trial	Intervention - not self-care
Walia_2020	Effect of sharing health messages on antenatal care behavior among women involved in microfinance-based self-help groups in Bihar India	Intervention - not self-care
Walia_2021	Routine deworming during antenatal care decreases risk of neonatal mortality and low birthweight: A retrospective cohort of survey data	Intervention - not self-care
Wapner_1995	A randomized multicenter trial assessing a home uterine activity monitoring device used in the absence of daily nursing contact	Intervention - not self-care
Watson_1990	Management of preterm labor patients at home: does daily uterine activity monitoring and nursing support make a difference?	Intervention - not self-care
Webb_2012	The effect of anthelmintic treatment during pregnancy on HIV plasma viral load: results from a randomized, double-blind, placebo-controlled trial in Uganda	Intervention - not self-care
Wei_2016	Effect of a CGMS and SMBG on Maternal and Neonatal Outcomes in Gestational Diabetes Mellitus: a Randomized Controlled Trial	Intervention - not self-care
Willcox_2011	Is parasite clearance clinically important after malaria treatment in a high transmission area? A 3-month follow-up of home-based management with herbal medicine or ACT	Intervention - not self-care
Williams_2012	Associations between preconception counseling and maternal behaviors before and during pregnancy	Intervention - not self-care
Witt_2016	Therapeutic Breast Massage in Lactation for the Management of Engorgement, Plugged Ducts, and Mastitis	Intervention - not self-care
Witteveen_2020	Effectiveness of a guided ACT-based self-help resilience training for depressive symptoms during pregnancy: Study protocol of a randomized controlled trial embedded in a prospective cohort	Intervention - not self-care
Wyck_2007	Intravenous Ferric Carboxymaltose Compared With Oral Iron in the Treatment of Postpartum Anemia: A Randomized Controlled Trial	Intervention - not self-care
Xiu_2020	Psychological intervention for patients with high-risk pregnancy can improve their self-management ability and pregnancy outcome	Intervention - not self-care
Yang_2018	Medical nutrition treatment of women with gestational diabetes mellitus by a telemedicine system based on smartphones	Intervention - not self-care
Yu_2019	Application and Utility of Continuous Glucose Monitoring in Pregnancy: A Systematic Review	Intervention - not self-care
Zaharieva_2020	Continuous Glucose Monitoring Versus Self-Monitoring of Blood Glucose to Assess Glycemia in Gestational Diabetes	Intervention - not self-care
Zaharieva_2021	Continuous Glucose Monitoring Versus Self-Monitoring of Blood Glucose to Assess Glycemia in Gestational Diabetes	Intervention - not self-care
Zandinava_2017	Effect of educational package on Self-Care behavior, quality of life, and blood glucose levels in pregnantwomen with gestational diabetes: A randomized controlled trial	Intervention - not self-care
Zhang_2021	The effect of individualized exercise prescriptions combined with dietary management on blood glucose in the second-and-third trimester of gestational diabetes mellitus	Intervention - not self-care
Abobaker_2020	Study the effect of non-pharmaceutical approach on traumatic nipple for lactating mothers	No full text available
Abraham-Justice_2010	The effects of a postpartum education program on self-care and healthcare-seeking behaviors in mothers	No full text available
Afshar_2012	Effect of a sex educational package on sexual function in pregnant women: a multicenter randomized controlled trial	No full text available
Agot_2018	Secondary distribution of HIV self-tests as a way to promote HIV testing among male partners of young women: subgroup analysis from a randomized trial	No full text available
Alipour_2017	The effectiveness of relaxation techniques on depression, anxiety and stress in pregnant women: based on self-efficacy theory	No full text available
Arpin_2007	Evaluation of pain relief as well as nurses' and mothers' satisfaction following the implementation of an automedication program in obstetrics	No full text available
Artal_1996	Home uterine activity monitoring and its role in reducing the incidence of prematurity	No full text available
Askari_2016	Effect of back massage with sesames oil on pain and length of delivery in primiparous women	No full text available
Asklund_2019	The use of an app with a pfmt programme among pregnant and postnatal women for preventive use and treatment of urinary incontinence	No full text available
Attanayake_2016	Cervical ripening with self administered iso sorbide mononitrate vaginally, in uncomplicated singleton pregnancies at 39 weeks gestation: a double blind randomised controlled trial	No full text available
BabaDizavandi_2012	The effect of music therapy on labour pain in nulliparous women in mashad, Iran in 2010	No full text available
Bahri_2015	Educational intervention to improve oral health beliefs and behaviors during pregnancy: a randomized-controlled trial	No full text available
Bais_2018	A randomized, double-blind controlled clinical trial of light therapy for pregnant women with major depressive disorder	No full text available

For peer review only - http://bmjopen.bmj.com/site/about/guidelines.xhtml

Author/Year	Title	Reasons for exclusion
Barfoot_2015	A pilot randomised trial of 4 physiotherapy interventions for pregnancy related pelvic girdle pain	No full text available
Beeharry_2017	A multimedia approach to diabetes structured education: the effects of “Keeping Healthy After Gestational Diabetes (GDM)”, a multimedia based education program on patient knowledge, self-efficacy and acceptability	No full text available
Berry_2015	A Postpartum Community-Based Weight Management Intervention Designed for Low-Income Women: Feasibility and Initial Efficacy Testing	No full text available
Bertz_2010	Dietary restriction or dietary restriction and exercise, but not exercise intervention alone, reduces weight and fat mass in overweight and obese women postpartum	No full text available
Bjorn_2013	Relaxation strategies during pregnancy Health benefits	No full text available
Blondel_1992	Home uterine activity monitoring in France: a randomized, controlled trial	No full text available
Buhling_2004	Introductory experience with the continuous glucose monitoring system (CGMS; Medtronic Minimed) in detecting hyperglycemia by comparing the self-monitoring of blood glucose (SMBG) in non-pregnant women and in pregnant women with impaired glucose tolerance	No full text available
Bung_1993	[Regular exercise therapy in disorders of carbohydrate metabolism in pregnancy--results of a prospective, randomized longitudinal study]	No full text available
Burke_2019	Feasibility and acceptability of home use of a smartphone-based urine testing application among women in prenatal care	No full text available
Cairns_2017	A novel self-management intervention for adjustment of postnatal antihypertensive treatment	No full text available
Cairns_2017	Self-management of postnatal antihypertensive treatment: a pilot randomised controlled trial	No full text available
Carissoli_2017	Enhancing psychological wellbeing of women approaching the childbirth: A controlled study with a mobile application	No full text available
Cohn_2014	Mind in Labor: Effects of Mind/Body Training on Childbirth Appraisals and Pain Medication Use During Labor	No full text available
Das_2015	Effect of a behavioral intervention with cereal fiber or resistant starch on gestational weight gain: a randomized clinical trial	No full text available
DavimRMB_2008	Non pharmacological strategies evaluation in parturients' pain relief	No full text available
DavimRMB_2008	Showering as a non pharmacological strategy to relief the parturients pain	No full text available
Dyson_1988	The role of home uterine monitoring in the prevention of preterm birth in high risk patients	No full text available
Dyson_1990	Preterm birth prevention - the role of education - palpation vs home uterine monitoring in non-multiple gestations	No full text available
Dyson_1991	Prevention of preterm birth in high-risk patients: the role of education and provider contact versus home uterine monitoring	No full text available
El-Refaey_2020	The effect of tailored psycho-educational program on pregnant women's anxiety and knowledge about self-care management regarding minor discomforts	No full text available
Epp_2019	A Review of the Effects of Physical Therapy on Self-Esteem in Postpartum Women With Lumbopelvic Dysfunction	No full text available
Ershoff_1990	Pregnancy and medical cost outcomes of a self-help prenatal smoking cessation program in an HMO	No full text available
Ershoff_1995	Relapse prevention among women who stop smoking early in pregnancy: A randomized clinical trial of a self-help intervention	No full text available
Eva_2021	Virtual reality in obstetrics as a non pharmacological method	No full text available
Evans_2020	Non-pharmacological interventions to reduce the symptoms of mild to moderate anxiety in pregnant women A systematic review and narrative synthesis of women’s views on the acceptability of and satisfaction with interventions	No full text available
Evans_2020	Developing a complex intervention to support pregnant women with mild to moderate anxiety: application of the Medical Research Council framework	No full text available
Evies_2007	Effects of education for self-care on the metabolic control of pregnant diabetic patients	No full text available
Fakhri_2017	Effect of education based on health belief model with relaxation on anxiety of nulliparouse women	No full text available
Faraji_2016	The impact of supplementation with fish oil on lipid profile of pregnant mothers: a randomized controlled trial	No full text available
Farajnejad_2020	Aromatherapy in postpartum depression: a clinical trial based on precede model	No full text available
Fawcett_1993	Effects of information on adaptation to cesarean birth	No full text available
Feingold_1993	Contribution of home uterine activity monitoring to the reduction of preterm delivery rate	No full text available
Frye_2015	On the trail of misoprostol in the community: a secondary analysis of self-administered misoprostol for the prevention of postpartum hemorrhage in Uganda	No full text available
Geelhoed-Duijvestijn_2016	Real-time continuous glucose monitoring during pregnancy in women with type 1 diabetes: is RTCGM related to better glycaemic control and key obstetric outcomes?	No full text available
Ghasemi_2018	The effect of group counseling with cognitive-behavioral approach on self-efficacy of pregnant women's choice of vaginal delivery	No full text available
Gookin_1994	Randomized comparison of home uterine activity monitoring and routine care in patients discharged after treatment for preterm labor	No full text available
Greene_1999	A postpartum self-medication program: Effect on narcotic use	No full text available
Grimes_1992	Randomized controlled trials of home uterine activity monitoring: a review and critique	No full text available
Guerra_2005	Educational for self care of pregnant diabetic patients	No full text available
Hannover_2004	Interventions to prevent health risks due to tobacco smoke in pregnant women, women postpartum and their infants	No full text available

For peer review only - http://bmjopen.bmj.com/site/about/guidelines.xhtml

Author/Year	Title	Reasons for exclusion
Hanson_1984	Self-monitoring of blood glucose by diabetic women during the third trimester of pregnancy	No full text available
Hartmann_1996	A randomized controlled trial of smoking cessation intervention in pregnancy in an academic clinic	No full text available
Henrique_2018	Pain, anxiety, and stress during childbirth-management without medication	No full text available
Hill_1990	Home uterine activity monitoring is associated with a reduction in preterm birth	No full text available
Hjalmarson_1991	Stopping smoking in pregnancy: effect of a self-help manual in controlled trial	No full text available
Hodnett_1999	Home?based social support for socially disadvantaged mothers	No full text available
Holden_2016	Yoga for pregnancy related back pain	No full text available
Hollingsworth_1987	Postprandial walking exercise in pregnant insulin-dependent (type I) diabetic women: reduction of plasma lipid levels but absence of a significant effect on glycemic control	No full text available
Horio_1998	Fetal monitor for non-stress-test screening at home	No full text available
Howell_2011	Mothers avoiding depression through empowerment intervention trial (made it)	No full text available
Hoyme_1998	Reduction of prematurity by pH-screening	No full text available
Hsieh_2017	Efficacy of Warm Showers on Postpartum Fatigue Among Vaginal-Birth Taiwanese Women: A Quasi-Experimental Design	No full text available
Huddle_1987	Diabetes in pregnancy: The use of home blood glucose monitoring and intensive monitoring to ensure favourable perinatal outcome	No full text available
Iams_1987	A prospective random trial of home uterine activity monitoring in pregnancies at increased risk of preterm labor	No full text available
Iams_1988	A prospective random trial of home uterine activity monitoring in pregnancies at increased risk of preterm labor Part II	No full text available
Iams_1992	Randomized controlled trials of home uterine activity monitoring: a review and critique	No full text available
Iams_1995	Current status of home uterine activity monitoring	No full text available
Igarashi_2010	Effects of aromatherapy for self-care during pregnancy	No full text available
Jack_2019	Effect of Addition of an Intimate Partner Violence Intervention to a Nurse Home Visitation Program on Maternal Quality of Life: a Randomized Clinical Trial	No full text available
Jaqin_2019	Differences in self-efficacy before and after antenatal education	No full text available
Jeon_2018	Effects of an educational program on knowledge and Efficacy of self-care during pregnancy among marriage immigrant women in South Korea	No full text available
Johnson_2000	Preventing smoking relapse in postpartum women	No full text available
Johnston_2006	The Community Perinatal Care Study: home visiting and nursing support for pregnant women	No full text available
Kaplan_2011	The impact of a behavioral intervention on weight gain during pregnancy	No full text available
Kavanagh_2020	Does an online wellbeing program (Baby Steps) prvent perinatal distress in first-time fathers and mothers?	No full text available
Kaveh_2012	Impact of Education on Nutrition and Exercise on the Level of Knowledge and Metabolic Control Indicators (FBS & PPBS) of Gestational Diabetes Mellitus (GDM) Patients	No full text available
Knuppel_1990	Preventing preterm birth in twin gestation: home uterine activity monitoring and perinatal nursing support	No full text available
Kyung_2003	Effects of postpartum care program for primiparous women and care-givers on the knowledge and behavior of postpartum care and postpartum recovery in primiparous women	No full text available
Lara_2010	Retention rates and potential predictors in a longitudinal randomized control trial to prevent postpartum depression	No full text available
Larsen_2001	A prospective study of self-efficacy expectancies and labour pain	No full text available
Lee_2018	Implementation and evaluation of gestational diabetes management using mobile health care service-a pilot study	No full text available
Lehugeur_2017	Non-Pharmacological Management Of Relief In Deliveries Assisted By An Obstetric Nurse	No full text available
Lillington_1995	Evaluation of a smoking cessation program for pregnant minority women	No full text available
Lipatov_2019	Possibility of non-pharmacological and pharmacological therapies for placental insufficiency	No full text available
Liu_2010	Effects of music therapy on labour pain and anxiety in Taiwanese first-time mothers	No full text available
Lombard_2011	A randomized controlled trial investigating self-weighing and the prevention of excess weight gain in early pregnancy	No full text available
Lowe_1998	Evaluation of antenatal smoking cessation programs for pregnant women	No full text available
Lumluk_2011	Effect of antenatal education for better self-correct diagnosis of true labor: a randomized control study	No full text available
Luo_2015	Influence of Orem self-care model on birth outcomes of gestational diabetes mellitus patients	No full text available
Luoto_2012	Physical activity and dietary counseling and supervised group exercise for first-time pregnant women - a feasibility study of a controlled trial	No full text available
Maloni_1998	Prescription of activity restriction to treat high-risk pregnancies	No full text available
Marquez-Sterling_2000	Physical and psychological changes with vigorous exercise in sedentary primigravidae	No full text available
Millan-Ferro_2017	Participation of latinos with type 2 diabetes in a culturally and linguistically oriented virtual diabetes self-care and education program	No full text available
Miller_2004	Improving the identification and treatment of postpartum depression in a managed care organization	No full text available
Mirzakhani_2015	The effect of exercise with special ball during pregnancy on duration of active phase of first stage of birth in nulliparous women	No full text available
Mohammadi_2021	The effect of pregnancy training classes based on bandura self-efficacy theory on postpartum depression and anxiety and type of delivery	No full text available

For peer review only - http://bmjopen.bmj.com/site/about/guidelines.xhtml

Author/Year	Title	Reasons for exclusion
Morrison_2003	Current status of home uterine activity monitoring	No full text available
Mortazavi_2021	Effectiveness of solution-focused counseling therapy on pregnancy anxiety and fear of childbirth: A randomized clinical trial	No full text available
Mou_1991	Multicenter randomized clinical trial of home uterine activity monitoring for detection of preterm labor	No full text available
Mullen_1999	Maternal smoking during pregnancy and evidence-based intervention to promote cessation	No full text available
Naef_1998	Home blood pressure monitoring for pregnant patients with hypertension	No full text available
Nagey_1993	Randomized comparison of home uterine activity monitoring and routine care in patients discharged after treatment for preterm labor	No full text available
Nehbandani_2018	Comparison the effects of aromatherapy with rose extract and lavender on the pain of the active phase of labor in primipara women	No full text available
Newman_1988	Randomized trial of home uterine activity monitoring	No full text available
Norheim_2001	Acupressure treatment of morning sickness in pregnancy A randomised, double-blind, placebo-controlled study	No full text available
O'Connor_1992	Effectiveness of a pregnancy smoking cessation program	No full text available
Pasinlioglu_2004	Health education for pregnant women: The role of background characteristics	No full text available
Pawalia_2018	Behavioral Intervention during Pregnancy for Preventing Abdominal Obesity and Pregnancy Complications in Indian Women: Protocol for a Randomised Controlled Trial	No full text available
Petersen_1992	Smoking reduction during pregnancy by a program of self-help and clinical support	No full text available
Pont_2019	Prenatal yoga program on primigravida 3rd trimester reducing complaints in the first stage and self-efficacy in the labor	No full text available
Pormosayebi_2018	The effect of training based on health belief model (HBM) in preventing exposure to polluted air in pregnant women	No full text available
Posadas_2014	Intake of vitamin C, probiotics, flavonoids and nutritional status in pregnant women with urinary tract infection	No full text available
Pratt_2016	Effect of a brief educational intervention concerning the human papillomavirus on the knowledge and attitudes of antenatal patients in New Providence, The Bahamas	No full text available
Ramezanpoor_2019	The effectiveness of an educational intervention based on social cognitive theory on fruit and vegetable intake in pregnant women	No full text available
Ratner_2000	Twelve-month follow-up of a smoking relapse prevention intervention for postpartum women	No full text available
Rees_1995	Effect of relaxation with guided imagery on anxiety, depression, and self-esteem in primiparas	No full text available
Reichmann_2009	Home uterine activity monitoring: an evidence review of its utility in multiple gestations	No full text available
Reyhani_2014	Investigating the effects of spiritual self-care training on psychological stress of mothers with preterm infants admitted in neonatal intensive care unit	No full text available
Rezaeean_2017	Application of Orem's theory for promotion of self-care behaviors of pregnant women at risk for preterm delivery: A clinical trial	No full text available
Sanyal_2019	Management of gestational diabetes in a resource-limited setting in India	No full text available
Seto_2019	The Accuracy of Self-Screening of Group B Streptococcus in Pregnant Women-A Randomized Crossover Study	No full text available
Shobeiri_2016	The effect of orem's self care model on control of preeclampsia in pregnant women: A randomized clinical trial	No full text available
Smith_1990	Reliability of compact electronic blood pressure monitors for hypertensive pregnant women	No full text available
Smith_1992	Patient's acceptance of monitoring fetal movement: A randomized comparison of charting techniques	No full text available
Smith_1993	Educational efforts in preventing preterm delivery among inner city adolescents	No full text available
Smith_1994	Reduction of preterm births in a low-income population utilizing home uterine activity monitoring	No full text available
Sunderji_1991	Earlier detection of preterm labor: multicenter prospective randomized clinical trial of home uterine activity monitoring	No full text available
Swaroopa_2017	Effect of antenatal education in improving maternal confidence and reducing anxiety about labor in primigravida women attending saveetha medical college and hospital,chennai,tamil nadu, india	No full text available
Taheri_2021	The effect of health belief-based education on physical activates of nulliparous women: a randomized control trial	No full text available
Tian_2013	Effects of birth ball exercises on labor pain and childbirth satisfaction	No full text available
Unger_2015	Mobile wach: developing and evaluating a humancomputer hybrid mobile messaging system for women's and children's health in Kenya	No full text available
Valbo_1994	Smoking cessation in pregnancy: The effect of self-help manuals	No full text available
Vasaiya_2020	Comparison between Effect of Foot Exercise and Warm Water Foot Soak on Foot Edema among Antenatal Women	No full text available
Wahyuni_2020	The effect of early mobilization towards self-care levels in post-sectio caesarea mother with self-care theory approach	No full text available
Wardani_2018	The effectiveness of acupressure at LI 4 and SP 6 point on uterine contraction in the first stage of labor on primiparous women	No full text available
Wilson_2020	Making BUMP work: intervention development in a large randomised controlled trial of blood pressure self-monitoring in pregnancy	No full text available
Wing_2010	Comparison of urinary cytokines after ingestion of cranberry juice cocktail in pregnant subjects: a pilot study	No full text available

For peer review only - http://bmjopen.bmj.com/site/about/guidelines.xhtml

Author/Year	Title	Reasons for exclusion
Wirz-Justice_2011	A randomized, double-blind, placebo-controlled study of light therapy for antepartum depression	No full text available
Yildirim_2004	The effect of breathing and skin stimulation techniques on labour pain perception of Turkish women	No full text available
Zardosht_2021	Effect of chamomile oil on cesarean section pain in primiparous women: a randomized clinical trial	No full text available
Zeng_2008	A research on relationship between fetal growth restriction and vitamin nutritional status in late pregnancy	No full text available
Ainscough_2016	Impact of an mHealth supported healthy lifestyle intervention on behavioural stage of change in overweight and obese pregnancy	Not a peer-reviewed journal article
Al-Hashmi_2017	The Effectiveness of a Health Education Intervention on Self-Efficacy for Adherence to Healthy Behaviors among Women with Gestational Diabetes	Not a peer-reviewed journal article
Andalib_2016	Evaluation of the effect of corrected nutrition on reduction of stress in gestational diabetes	Not a peer-reviewed journal article
Apay_2015	The Effect of the Care Given Using Orem's Self-Care Model on the Postpartum Self- Evaluation	Not a peer-reviewed journal article
Barrett_2017	Home Monitoring of Fasting and Postprandial Triglycerides in Late Pregnancy: A Pilot Study	Not a peer-reviewed journal article
Brixval_2016	Antenatal education in small classes may increase childbirth self-efficacy: Results from a Danish randomised trial	Not a peer-reviewed journal article
Buckingham-Schutt_2019	The Behavioral Wellness in Pregnancy Study: a theory-based multi-component intervention to promote appropriate weight gain and healthy lifestyle behaviors in previously sedentary pregnant women	Not a peer-reviewed journal article
Butler_2019	Assessing and managing risk online: a clinician's perspective on internet-delivered intervention, 'MUMentum', for perinatal distress, anxiety, and depression	Not a peer-reviewed journal article
Chappell_2021	Self-monitoring of blood pressure in women with pregnancy hypertension: the BUMP2 multicentre randomised controlled trial	Not a peer-reviewed journal article
Chaudhry_2020	Development and assessment of smartphone based intervention (TechMotherCare) for maternal depression in Pakistan	Not a peer-reviewed journal article
Cheung_2011	A pilot structured behavioural intervention trial to increase physical activity among women with recent gestational diabetes	Not a peer-reviewed journal article
Chiarelli_2002	A physiotherapist-led programme of postpartum pelvic floor exercises reduced urinary incontinence at 3 months	Not a peer-reviewed journal article
Dahlen_2011	Moxibustion for cephalic version: a feasibility study	Not a peer-reviewed journal article
Dal_2021	The effect of pregnancy yoga on breastfeeding self-efficacy and prenatal attachment: A semi-experimental study	Not a peer-reviewed journal article
Dias_2011	Effectiveness of perineal exercises in controlling urinary incontinence and improving pelvic floor muscle function during pregnancy	Not a peer-reviewed journal article
Draffin_2013	Gestational diabetes: things you need to know (but maybe don't): development of an educational DVD for women recently diagnosed with gestational diabetes	Not a peer-reviewed journal article
Durmaz_2016	The Self-Care Training is Increasing Body Image Perception Self- Esteem and Self-Care Abilities in Pregnant Adolescents: A Pretest- Posttest Quasi-Experimental Study	Not a peer-reviewed journal article
Ericksen_2020	Treatment for antenatal anxiety and depression with Beating the Blues before Birth BBB© positively impacts infant postnatal development at 9 months, a pilot RCT	Not a peer-reviewed journal article
ErtekinPinar_2016	Effects of coping with stress training on pregnant women's depression, stress and coping with stress levels	Not a peer-reviewed journal article
Fancourt_2017	Associations between singing to babies and symptoms of postnatal depression, wellbeing, self-esteem and mother-infant bond	Not a peer-reviewed journal article
Farrell_2013	The use of logs and forms for the tracking of RT-CGM devices in the conceptt trial	Not a peer-reviewed journal article
Fox_2021	Short term postpartum blood pressure management and long-term blood pressure control: a randomised controlled trial	Not a peer-reviewed journal article
Freeman_1986	Randomised trial of self hypnosis for analgesia in labour	Not a peer-reviewed journal article
Goldberg_1986	Gestational diabetes: impact of home glucose monitoring on neonatal birth weight	Not a peer-reviewed journal article
Gunther_2019	The effect of lifestyle counselling on dietary behavior of pregnant women - Secondary results of the randomised controlled GeliS trial	Not a peer-reviewed journal article
Hadar_2021	Mobile Self-Operated Home Ultrasound System for Remote Fetal Assessment During Pregnancy	Not a peer-reviewed journal article
Harrison_2014	How effective is self-weighing in the setting of a lifestyle intervention to reduce gestational weight gain and postpartum weight retention?	Not a peer-reviewed journal article
Herring_2017	Intervening during and after pregnancy to prevent weight retention among African American women	Not a peer-reviewed journal article
Hill_2012	Nutrient intake of pregnant women with type 1 diabetes in the DAPIT Trial; relationships with anthropometry and glycaemic control	Not a peer-reviewed journal article
Hoffmann_2020	Does an antenatal lifestyle intervention in routine care improve maternal and infant health outcomes in the first year postpartum-12 months follow-up of the cluster-randomised GeliS trial	Not a peer-reviewed journal article
Hoirisch-Clapauch_2016	A protocol combining daily walking and a lowglycemic index diet increases the rate of take-home babies in women with consecutive first-trimester miscarriages	Not a peer-reviewed journal article
Houshmandpour_2019	The effect of midwife-oriented group counseling, based on orem's model on self-care and empowerment, in primiparous women: A clinical trial	Not a peer-reviewed journal article

For peer review only - http://bmjopen.bmj.com/site/about/guidelines.xhtml

Author/Year	Title	Reasons for exclusion
Howharn_2008	Effects of childbirth preparation classes on self-efficacy in coping with labor pain in Thai primiparas	Not a peer-reviewed journal article
Hoyme_2002	Results and potential consequences of the thuringia prematurity preventional campaign 2000	Not a peer-reviewed journal article
Hunt_2018	Evaluation of a home-use oral glucose tolerance test kit for screening for gestational diabetes	Not a peer-reviewed journal article
Hutchesson_2019	Be Healthe for your Heart Study Protocol: preventing cardiovascular disease among women with a history of pre-eclampsia	Not a peer-reviewed journal article
Ip_2005	The effect of a theory-based intervention on promoting self-efficacy for childbirth among pregnant women in Hong Kong	Not a peer-reviewed journal article
Keytash_2015	Impact of preconception care on overweight/obese women: long-term effects of a behavioural change intervention	Not a peer-reviewed journal article
Kirop_2021	Feasibility and Acceptability of a Telephone Support Intervention During Early Postnatal Period Among Teenage Mothers in Western Kenya: A Pilot Randomised Controlled Trial	Not a peer-reviewed journal article
Kisa_2019	Repeat HIV testing of individuals with discrepant HIV self-test results in Central Uganda	Not a peer-reviewed journal article
Lee_2016	Did self-sampling improve the adherence to group B streptococci screening in pregnant women?	Not a peer-reviewed journal article
Lineberger_1987	Pregnant adolescents attending prenatal parent education classes: self-concept, anxiety and depression levels	Not a peer-reviewed journal article
Loukopoulou_2012	Urinary nicotine and cotinine as a method for assessing the effectiveness of an intervention to aid smoking cessation during pregnancy	Not a peer-reviewed journal article
Makvandi_2018	Effect of normal physiologic childbirth program in mother-friendly hospitals on duration of labor	Not a peer-reviewed journal article
Mayer_1990	A randomized evaluation of smoking cessation interventions for pregnant women at a WIC clinic	Not a peer-reviewed journal article
McGready_2001	A double-blind randomized therapeutic trial of insect repellents for the prevention of malaria in pregnancy	Not a peer-reviewed journal article
McManus_2017	The snap-HT trial: self-management of antihypertensive medication postpartum - Can women do it better?	Not a peer-reviewed journal article
McManus_2018	109 Using blood pressure selfmonitoring in pregnancy: A systematic review and individual patient data metaanalysis	Not a peer-reviewed journal article
Miquelutti_2012	Evaluation of the efficacy of an antenatal birth preparation program	Not a peer-reviewed journal article
Mirghafourvand_2019	Effectiveness of self-care training on pregnancy consequences in gestational diabetes: A randomized controlled clinical trial	Not a peer-reviewed journal article
Mollart_2019	Are women interested in Acupressure to increase spontaneous onset of labour?	Not a peer-reviewed journal article
Montazeri_2020	The effect of writing therapy on anxiety in pregnant women: a randomized controlled trial	Not a peer-reviewed journal article
Murphy_2016	Asthma self-management education and inhaled corticosteroid use during pregnancy and postpartum from 2004 to 2014	Not a peer-reviewed journal article
Neoh_2017	The conceptt-diet study: an analysis of diet and glycaemia in women with type 1 diabetes before and during pregnancy	Not a peer-reviewed journal article
Nguyen_2017	Feasibility and impacts of integrating nutrition interventions into an existing maternal, neonatal, and child health platform in Bangladesh	Not a peer-reviewed journal article
Nicklas_2016	Beta-testing a mobile health program designed to increase postpartum weight loss in women at elevated risk for cardiometabolic disease	Not a peer-reviewed journal article
Offiah_2020	A feasibility study on the prevention of post-partum stress urinary incontinence by midwife-delivered antenatal pelvic floor muscle training: a cluster randomised controlled trial	Not a peer-reviewed journal article
Parry_2017	Early vs Late wake therapy improves mood in antepartum vs Postpartum depression by differentially altering melatonin and sleep timing	Not a peer-reviewed journal article
Pintye_2019	Self-tests for at-home partner testing are acceptable & utilized among pregnant women	Not a peer-reviewed journal article
Porreco_2020	Fetal Movement Counting and Perinatal Mortality: A Systematic Review and Meta-analysis	Not a peer-reviewed journal article
Roche_2018	Analysis of comparable home/clinic blood pressure readings in the LEANBH (Learning to Evaluate and manage ANtenatal Blood pressure at Home) population	Not a peer-reviewed journal article
Rowe_2019	Online and mobile psychoeducation initiatives to prevent postnatal mental health problems: research and evaluation	Not a peer-reviewed journal article
Rubertsson_2020	A randomized controlled study during pregnacy comparing internetbased cognitive behavioral therapy and counseling by standard care for fear of birth	Not a peer-reviewed journal article
Ruggiero_1997	A stage-matched smoking cessation program for pregnant smokers	Not a peer-reviewed journal article
Sanavi_2014	Effective education to decrease elective caesarean section	Not a peer-reviewed journal article
Secher_2014	Real-time continuous glucose monitoring as a tool to prevent severe hypoglycaemia in selected pregnant women with Type 1 diabetes - an observational study	Not a peer-reviewed journal article

For peer review only - http://bmjopen.bmj.com/site/about/guidelines.xhtml

Author/Year	Title	Reasons for exclusion
Slade_2019	Preventing post-traumatic stress disorder: the Stress and Wellbeing after Childbirth Study(STRAWB2)	Not a peer-reviewed journal article
Smith_2014	Investigation of a lifestyle change strategy for high-risk women with a history of gestational diabetes	Not a peer-reviewed journal article
Smith_2014	The Blossom Project Online: Use of a behaviorally-based website to promote physical activity and prevent excessive gestational weight gain in previously sedentary pregnant women	Not a peer-reviewed journal article
Suhaimi_2016	A low-gi diet improves glucose self-monitoring in women with gestational diabetes mellitus	Not a peer-reviewed journal article
Tappin_2000	The effect of home-based motivational interviewing on the smoking behaviour of pregnant women: a pilot randomized controlled efficacy study	Not a peer-reviewed journal article
Tucker_2019	Self-monitoring of blood pressure during pregnancy: the BUMP trials	Not a peer-reviewed journal article
Varrassi_1989	Effects of physical activity on maternal plasma beta-endorphin levels and perception of labor pain	Not a peer-reviewed journal article
Wapner_1996	The CHUMS (Collaborative Home Uterine Monitoring Study) clinical trial - What does it really say?	Not a peer-reviewed journal article
Weeks_2013	Self-administration of misoprostol to prevent bleeding after homebirths in uganda: a pilot placebo-controlled, randomised trial	Not a peer-reviewed journal article
Wei_2013	Earlier management with continuous monitoring blood glucose decrease gestational weight gain in gestational diabetes mellitus: a randomised clinical trial	Not a peer-reviewed journal article
Wong_2015	A randomized controlled trial of an educational intervention to improve influenza vaccine uptake among pregnant women	Not a peer-reviewed journal article
Wroblewski_2004	Implementing a comprehensive postpartum depression support program	Not a peer-reviewed journal article
Wu_2018	The application of online pelvic floor training during the rehabilitation of postpartum pelvic floor	Not a peer-reviewed journal article
XiangHu_2020	Effects of exercise therapy for pregnancy-related low back pain and pelvic pain: A protocol for systematic review and meta-analysis	Not a peer-reviewed journal article
Yang_2018	Effectiveness of aerobic gymnastic exercise on stress, fatigue, and sleep quality during postpartum: a pilot randomized controlled trial	Not a peer-reviewed journal article
Yazdkhasti_2018	The effect of localized heat and cold therapy on pain intensity, duration of phases of labor, and birth outcomes among primiparous females: a randomized, controlled trial	Not a peer-reviewed journal article
Zlotnick_2001	Postpartum Depression in Women Receiving Public Assistance: Pilot Study of an Interpersonal-Therapy-Oriented Group Intervention	Not a peer-reviewed journal article
Ahn_2011	Effects of breast massage on breast pain, breast-milk sodium, and newborn suckling in early postpartum mothers	Not in English
Bakhteh_2018	Effect of cognitive behavioral therapy on self-efficacy in women with gestational diabetes: A randomized clinical trial	Not in English
Chae_2021	[Maternal Health Effects of Internet-Based Education Interventions during the Postpartum Period: A Systematic Review]	Not in English
deSouzaMelo_2020	Maternal and perinatal parameters after non-pharmacological interventions: a randomised, controlled clinical trial	Not in English
Gayeski_2010	Non-pharmacological approach to pain relief during labor as hard-light care technology: a systematic review	Not in English
Ghaderpanah_2017	The effect of 5A model on behavior change of physical activity in overweight pregnant women	Not in English
Ghazaie_2016	The effectiveness of cognitive-behavioral therapy on fear of childbirth, fear of pain, self-efficacy of childbirth and tendency to caesarean in nulliparous women	Not in English
Hanjani_2013	The effect of foot reflexology on pain intensity and duration of labor on primiparous	Not in English
Hung_2017	Non-pharmacological interventions for pregnancy-related sleep disturbances	Not in English
Imano_2013	Evaluation of program to improve mental and physical health	Not in English
Jeon_2018	Effects of a postnatal care program on self-efficacy, self-management, and glycemic control in women with gestational diabetes mellitus	Not in English
Khadivzadeh_2015	Effects of self-care education on perceived stress in women with gestational diabetes under insulin treatment	Not in English
Kharazi_2018	Effect of an educational intervention based on self-efficacy theory and health literacy strategies on pregnancy outcomes: A randomized clinical trial	Not in English
Khodaparast_2020	Examining the effect of educational intervention based on self-regulation model on perceived stress in women with gestational diabetes: A clinical trial study	Not in English
Khojasteh_2018	Comparison of in-person counseling and telephone support on delivery self-efficacy in primiparous women	Not in English
Kim_2014	Effects of an integrated self-management program on self-management, glycemic control, and maternal identity in women with gestational diabetes mellitus	Not in English
Kim_2021	Effects of nursing intervention programs for women with gestational diabetes: a systematic review of randomized controlled trials	Not in English
KimSue_2011	Effects of a Coaching-based Childbirth Program on Anxiety and Childbirth Self-efficacy among Primigravida Women	Not in English
Ko_2014	Effects of a coaching program on comprehensive lifestyle modification for women with gestational diabetes mellitus	Not in English

For peer review only - http://bmjopen.bmj.com/site/about/guidelines.xhtml

Author/Year	Title	Reasons for exclusion
Lawani_2003	Effect of antenatal gymnastics on childbirth: a study on 50 sedentary women in the Republic of Benin during the second and third quarters of pregnancy	Not in English
Lee_2017	Development and Validation of a Postpartum Care Mobile Application for First-time Mothers	Not in English
Lee_2018	Effects of family centered postpartum care for postpartum depression among previously infertile couples	Not in English
Liang_2019	Effectiveness of a Labor-Admission Education Program on Anxiety, Uncertainty, Locus of Labor Control, and Labor Outcomes in Pregnant Women	Not in English
Madady_2017	Effect of hot shower and intravenous injection of hyoscine on childbirth experience of nulliparous women: a randomized clinical trial	Not in English
Mafetoni_2014	NON-PHARMACOLOGICAL METHODS FOR PAIN RELIEF DURING LABOR: INTEGRATIVE REVIEW	Not in English
Makhouli_2018	Implementation of targeted education to promote normal vaginal delivery: a randomized controlled trial	Not in English
Manabe_2006	Implementation and evaluation of health training guidance for the purpose of improving self-care behavior among primiparae	Not in English
Mascarenhas_2019	Scientific evidence on non-pharmacological methods for relief of labor pain	Not in English
Mazloomy-Mahmoodabad_2020	The Effect of Nutrition Education on Gestational Weight Gain based on the Pender's Health Promotion Model: a randomized clinical trial study	Not in English
Mohamadkhani-Shahri_2017	Effect of massage aromatherapy with Lavender oil on pain intensity of active phase of labor in nulliparous women	Not in English
Mojahed_2018	The effect of motivational interviewing on self-efficacy to quit hookah smoking in pregnant women	Not in English
Moller_2003	Current aspects of the Thuringia prematurity preventional campaign 2000	Not in English
Niazi_2018	Comparison of the effects of purslane cream and Lanolin for treatment of breast fissure on lactation self-efficacy: A randomized clinical trial	Not in English
Ozgoli_2016	Comparison of peppermint and clove essential oil aroma on pain intensity and anxiety at first stage of labor	Not in English
Pour_2012	Comparison of effect of transcutaneous electrical nerve stimulation and acupressure in decreasing labor pain in primiparous women	Not in English
Rezaeian_2017	The relationship of prenatal Self-Care behaviors with stress, anxiety and depression in women at risk of preterm delivery	Not in English
Rostampour_2020	The effect of couples' supportive counseling on self-care behavior in women with insulin-treated gestational diabetes: A randomized clinical trial	Not in English
Safat_2018	Efficacy of cognitive behavioral therapy-based self-management intervention on labor outcome in nulliparous women	Not in English
Saling_2000	New method for the self assessment of the pH-value of the vaginal secretions by an indicator coated panty liner - First preliminary communication	Not in English
Saling_2001	A simple, efficient and inexpensive program for preventing prematurity	Not in English
Shim_2012	[Effects of a yoga-focused prenatal program on stress, anxiety, self confidence and labor pain in pregnant women with in vitro fertilization treatment]	Not in English
Shimada_2005	A randomized controlled trial on evaluating effectiveness of perineal massage during pregnancy in primiparous women	Not in English
Siegmund-Schultze_2005	pH self assessment to reduce the risk of preterm birth - A pilot study becomes a model project	Not in English
Wan_2016	Clinical observation of analgesia in delivery treated with acupoint pressure therapy	Not in English
Wang_2015	Effects of a Mobile Web-based Pregnancy Health Care Educational Program for Mothers at an Advanced Maternal Age	Not in English
Khunpradit_2011	Non-clinical interventions for reducing unnecessary caesarean section	Older versions of a Cochrane review
Lauzon_1998	Antenatal education for self?diagnosis of the onset of active labour at term	Older versions of a Cochrane review
Moy_2014	Techniques of monitoring blood glucose during pregnancy for women with pre-existing diabetes	Older versions of a Cochrane review
Moy_2017	Techniques of monitoring blood glucose during pregnancy for women with pre-existing diabetes	Older versions of a Cochrane review
Pintye_2018	Preexposure Prophylaxis for Human Immunodeficiency Virus (HIV) Prevention Among HIV-uninfected Pregnant Women: Estimated Coverage Using Risk-based Versus Regional Prevalence Approaches	Outcome - feasibility/acceptability only
Smith_2016	Programmes for advance distribution of misoprostol to prevent post-partum haemorrhage: A rapid literature review of factors affecting implementation	Outcome - feasibility/acceptability only
Steinberg_2021	Optimizing Engagement in an mHealth Intervention for Diabetes Support During Pregnancy: the Role of Baseline Patient Health and Behavioral Characteristics	Outcome - feasibility/acceptability only
Aba_2017	Antenatal Education on Pregnant Adolescents in Turkey: Prenatal Adaptation, Postpartum Adaptation, and Newborn Perceptions	Outcome - no health or behavioural outcomes
Aveyard_2006	A randomized controlled trial of smoking cessation for pregnant women to test the effect of a transtheoretical model-based intervention on movement in stage and interaction with baseline stage	Outcome - no health or behavioural outcomes
Bastani_2010	Impact of preconception health education on health locus of control and self-efficacy in women	Outcome - no health or behavioural outcomes
Carolan-Olah_2016	Educational and intervention programmes for gestational diabetes mellitus (GDM) management: An integrative review	Outcome - no health or behavioural outcomes
Chang_2004	Effect of Taegyo-focused prenatal education on maternal-fetal attachment and self-efficacy related to childbirth	Outcome - no health or behavioural outcomes

For peer review only - http://bmjopen.bmj.com/site/about/guidelines.xhtml

Author/Year	Title	Reasons for exclusion
Chen_2021	The accuracy and influencing factors for preference of self-sampling in group B streptococcus screening: a cross-sectional study	Outcome - no health or behavioural outcomes
Chillrud_2021	The effect of clean cooking interventions on mother and child personal exposure to air pollution: results from the Ghana Randomized Air Pollution and Health Study (GRAPHS)	Outcome - no health or behavioural outcomes
Clarke_2005	Alternative site self blood glucose testing is preferred by women with gestational diabetes	Outcome - no health or behavioural outcomes
Conde_2019	Effect of Electronic Video Education on Patient's Self-Assessed Knowledge About Obesity and Pelvic Floor Disorders: A Randomized Controlled Trial	Outcome - no health or behavioural outcomes
Damodaran_2015	Effect of Progressive Muscle Relaxation Technique in Terms oi Anxiety and Physiological Parameters of Antenatal Mothers with Pregnancy-Induced Hypertension	Outcome - no health or behavioural outcomes
Dean_2012	A pilot study using interactive SMS support groups to prevent mother-to-child HIV transmission in South Africa	Outcome - no health or behavioural outcomes
Dickerson_2010	Pregnancy and village outreach tibet: a descriptive report of a community- and home-based maternal-newborn outreach program in rural Tibet	Outcome - no health or behavioural outcomes
Elsinga_2008	The effect of preconception counselling on lifestyle and other behaviour before and during pregnancy	Outcome - no health or behavioural outcomes
Evans_2014	Initial outcomes from a 4-week follow-up study of the Text4baby program in the military women's population: randomized controlled trial	Outcome - no health or behavioural outcomes
Grym_2019	Feasibility of smart wristbands for continuous monitoring during pregnancy and one month after birth 11 Medical and Health Sciences 1117 Public Health and Health Services	Outcome - no health or behavioural outcomes
Haakstad_2018	How does a lifestyle intervention during pregnancy influence perceived barriers to leisure-time physical activity? The Norwegian fit for delivery study, a randomized controlled trial	Outcome - no health or behavioural outcomes
Harris_2015	A Pilot Study of Home-Based Smoking Cessation Programs for Rural, Appalachian, Pregnant Smokers	Outcome - no health or behavioural outcomes
Harrison_2020	A consumer co-created infographic improves short-term knowledge about physical activity and self-efficacy to exercise in women with gestational diabetes mellitus: a randomised trial	Outcome - no health or behavioural outcomes
Hasani_2021	The effect of counseling based on health promotion awareness on mental health and self-esteem in women with ectopic pregnancy: a randomized controlled clinical trial	Outcome - no health or behavioural outcomes
Hausenblas_2008	Development and evaluation of a multimedia CD-ROM for exercise during pregnancy and postpartum	Outcome - no health or behavioural outcomes
Hazra_2020	Effects of health behaviour change intervention through women's self-help groups on maternal and newborn health practices and related inequalities in rural india: A quasi-experimental study	Outcome - no health or behavioural outcomes
Howarth_2019	Low-cost, self-paced, educational programmes increase birth satisfaction in first-time mothers	Outcome - no health or behavioural outcomes
Jack_2015	Reducing Preconception Risks Among African American Women with Conversational Agent Technology	Outcome - no health or behavioural outcomes
Ke_2021	Perioperative mobile application for mothers undergoing Cesarean delivery: a prospective cohort study on patient engagement	Outcome - no health or behavioural outcomes
Korukcu_2017	The effect of the mindfulness-based transition to motherhood program in pregnant women with preterm premature rupture of membranes	Outcome - no health or behavioural outcomes
Koury_2019	A 3Dimensional Anatomical Education Model in Postpartum Perineal Laceration Care: A PrePost Intervention Study	Outcome - no health or behavioural outcomes
Kuonza_2010	Non-adherence to the single dose nevirapine regimen for the prevention of mother-to-child transmission of HIV in Bindura town, Zimbabwe: a cross-sectional analytic study	Outcome - no health or behavioural outcomes
Lawrence_2003	Design and evaluation of interventions promoting periconceptional multivitamin use	Outcome - no health or behavioural outcomes
Ledford_2016	Mobile application as a prenatal education and engagement tool: A randomized controlled pilot	Outcome - no health or behavioural outcomes
Lepper_2018	Effectiveness of a Prison-Based Healthy Pregnancy Curriculum Delivered to Pregnant Inmates	Outcome - no health or behavioural outcomes
Liu_2019	Effects of exercise on pregnant women's quality of life: A systematic review	Outcome - no health or behavioural outcomes
Logsdon_2020	Partnering with new mothers to develop a smart phone app to prevent maternal mortality after hospital discharge: A pilot study	Outcome - no health or behavioural outcomes
Mahomed_2000	Home-based mother's record: operational feasibility, understanding and usage in a rural community in Zimbabwe	Outcome - no health or behavioural outcomes
Medley_2018	Interventions during pregnancy to prevent preterm birth: an overview of Cochrane systematic reviews	Outcome - no health or behavioural outcomes
Menard_2012	Self-collected vaginal swabs for the quantitative real-time polymerase chain reaction assay of Atopobium vaginae and Gardnerella vaginalis and the diagnosis of bacterial vaginosis	Outcome - no health or behavioural outcomes
Mendelson_2018	A mindfulness intervention to reduce maternal distress in neonatal intensive care: a mixed methods pilot study	Outcome - no health or behavioural outcomes
Mollart_2016	A feasibility randomised controlled trial of acupressure to assist spontaneous labour for primigravid women experiencing a post-date pregnancy	Outcome - no health or behavioural outcomes
Mozumdar_2018	Increasing knowledge of home based maternal and newborn care using self-help groups: Evidence from rural Uttar Pradesh, India	Outcome - no health or behavioural outcomes

For peer review only - http://bmjopen.bmj.com/site/about/guidelines.xhtml

Author/Year	Title	Reasons for exclusion
Muhammad_2019	Pelvic Floor Muscle Exercise Education and Factors Associated with Implementation among Antenatal Women in Hospital Universiti Sains Malaysia	Outcome - no health or behavioural outcomes
Murphy_2005	Asthma self-management skills and the use of asthma education during pregnancy	Outcome - no health or behavioural outcomes
Nadeau_2021	Beta Testing of a Gestational Diabetes Risk Reduction Intervention for American Indian and Alaska Native TeensPre-eclampsia Educational Tool Impact on Knowledge, Anxiety, and Satisfaction in Pregnant Women: A Randomized Trial	Outcome - no health or behavioural outcomes
Ockhuijsen_2015	Exploring a self-help coping intervention for pregnant women with a miscarriage history	Outcome - no health or behavioural outcomes
Osterman_2011	Feasibility of Using Motivational Interviewing to Decrease Alcohol Consumption During Pregnancy	Outcome - no health or behavioural outcomes
Parsa_2019	Improving the knowledge of pregnant women using a pre-eclampsia app: A controlled before and after study	Outcome - no health or behavioural outcomes
Paterno_2020	Feasibility of a pilot, randomized controlled trial using a personalized health monitoring device with pregnant women for behavioral sleep research	Outcome - no health or behavioural outcomes
PetrovFieril_2016	Hemodynamic responses to single sessions of aerobic exercise and resistance exercise in pregnancy	Outcome - no health or behavioural outcomes
Prado_2016	Effects of maternal and child lipid-based nutrient supplements on infant development: a randomized trial in Malawi	Outcome - no health or behavioural outcomes
Reilly_2021	Attitudes and engagement of pregnant and postnatal women with a web-based emotional health tool (mummatters): Cross-sectional study	Outcome - no health or behavioural outcomes
Rigaud_2017	Assessing a Tool for Self-Monitoring Hydration Using Urine Color in Pregnant and Breastfeeding Women: A Cross-Sectional, Online Survey	Outcome - no health or behavioural outcomes
Robins_2019	Treatment Fidelity in Mind–Body Interventions	Outcome - no health or behavioural outcomes
Ross-McGill_2000	Antenatal home blood pressure monitoring: a pilot randomised controlled trial	Outcome - no health or behavioural outcomes
Sapiano_2012	Attitudes towards preconception care in Maltese women with type 1 diabetes mellitus	Outcome - no health or behavioural outcomes
Sercekus_2010	Effects of antenatal education on maternal prenatal and postpartum adaptation	Outcome - no health or behavioural outcomes
Shahnazi_2016	Effect of educational intervention on perceived susceptibility self-efficacy and DMFT of pregnantwomen	Outcome - no health or behavioural outcomes
Sharps_2013	Engaging and retaining abused women in perinatal home visitation programs	Outcome - no health or behavioural outcomes
Snyder_2019	Mental and physical support (MAPS) for moms: preliminary findings from a prenatal health support program	Outcome - no health or behavioural outcomes
Szymanski_2018	Uterine Artery Doppler Velocimetry During Individually Prescribed Exercise in Pregnancy	Outcome - no health or behavioural outcomes
Thackeray_2017	Effectiveness of message framing on women's intention to perform cytomegalovirus prevention behaviors: A cross-sectional study	Outcome - no health or behavioural outcomes
Troy_1995	Development of a self-care guide for postpartum fatigue	Outcome - no health or behavioural outcomes
Truong_2019	Community pharmacist counseling in early pregnancy—Results from the SafeStart feasibility study	Outcome - no health or behavioural outcomes
Ventura_2012	Cortisol and anxiety response to a relaxing intervention on pregnant women awaiting amniocentesis	Outcome - no health or behavioural outcomes
Weis_2012	Mentors offering maternal support: a support intervention for military mothers	Outcome - no health or behavioural outcomes
Weis_2017	Mentors Offering Maternal Support Reduces Prenatal, Pregnancy-Specific Anxiety in a Sample of Military Women	Outcome - no health or behavioural outcomes
Aveyard-2005	The influence of in-pregnancy smoking cessation programmes on partner quitting and women's social support mobilization: A randomized controlled trial	Outcome - socioeconomic/financial
Ammerman_2011	An Open Trial of In-Home CBT for Depressed Mothers in Home Visitation	Population - beyond 42 days postpartum
Armstrong_2004	The effectiveness of a pram-walking exercise programme in reducing depressive symptomatology for postnatal women	Population - beyond 42 days postpartum
Ayers_2015	Brief Online Self-help Exercises for Postnatal Women to Improve Mood: A Pilot Study	Population - beyond 42 days postpartum
Ayers_2018	Evaluation of expressive writing for postpartum health: a randomised controlled trial	Population - beyond 42 days postpartum
Bennion_2020	Impact of an Internet-Based Lifestyle Intervention on Behavioral and Psychosocial Factors During Postpartum Weight Loss	Population - beyond 42 days postpartum
Berry_2013	Rationale, design, and methodology for the optimizing outcomes in women with gestational diabetes mellitus and their infants study	Population - beyond 42 days postpartum
Bick_2019	Protocol for a two-arm feasibility RCT to support postnatal maternal weight management and positive lifestyle behaviour in women from an ethnically diverse inner city population: The SWAN feasibility trial	Population - beyond 42 days postpartum
Brandon_2012	Self-help booklets for preventing postpartum smoking relapse: a randomized trial	Population - beyond 42 days postpartum

For peer review only - http://bmjopen.bmj.com/site/about/guidelines.xhtml

Author/Year	Title	Reasons for exclusion
Branquinho_2020	A blended cognitive–behavioral intervention for the treatment of postpartum depression: study protocol for a randomized controlled trial	Population - beyond 42 days postpartum
Carson_2013	Evaluation of home testing to improve follow up after gestational diabetes (Fingerstick Assessments of sugar two-months postpartum or FAST)	Population - beyond 42 days postpartum
Chen_2000	Effects of support group intervention in postnatally distressed women A controlled study in Taiwan	Population - beyond 42 days postpartum
Cramp_2006	Moms in motion: a group-mediated cognitive-behavioral physical activity intervention	Population - beyond 42 days postpartum
Cramp_2009	Sustaining self-regulatory efficacy and psychological outcome expectations for postnatal exercise: effects of a group-mediated cognitive behavioural intervention	Population - beyond 42 days postpartum
Crawley_2018	Feasibility and acceptability of expressive writing with postpartum women: A randomised controlled trial	Population - beyond 42 days postpartum
Daley_2008	Feasibility of an exercise intervention for women with postnatal depression: a pilot randomised controlled trial	Population - beyond 42 days postpartum
Daley_2012	The effectiveness of exercise as a treatment for postnatal depression: study protocol	Population - beyond 42 days postpartum
Daley_2015	A pragmatic randomized controlled trial to evaluate the effectiveness of a facilitated exercise intervention as a treatment for postnatal depression: the PAM-PeRS trial	Population - beyond 42 days postpartum
Daley_2020	Feasibility and acceptability of a brief routine weight management intervention for postnatal women embedded within the national child immunisation programme in primary care: randomised controlled cluster feasibility trial	Population - beyond 42 days postpartum
Daley_2021	Practice nurse-supported weight self-management delivered within the national child immunisation programme for postnatal women: a feasibility cluster RCT	Population - beyond 42 days postpartum
Danaher_2012	Web-based intervention for postpartum depression: Formative research and design of the mommoodbooster program	Population - beyond 42 days postpartum
Danaher_2013	MomMoodBooster web-based intervention for postpartum depression: feasibility trial results	Population - beyond 42 days postpartum
Dennis_2003	The effect of peer support on postpartum depression: a pilot randomized controlled trial	Population - beyond 42 days postpartum
deRosset_2013	Mama Sana Usted Sana: Lessons learned from a postpartum weight loss intervention for Hispanic women with infants six months or less	Population - beyond 42 days postpartum
Diebold_2021	Testing the effects of a prenatal depression preventive intervention on parenting and young children's self-regulation and functioning (EPIC): protocol for a longitudinal observational study	Population - beyond 42 days postpartum
Dunning_2013	Wide Awake Parenting: Study protocol for a randomised controlled trial of a parenting program for the management of post-partum fatigue	Population - beyond 42 days postpartum
Fjeldsoe_2010	MobileMums: A randomized controlled trial of an SMS-based physical activity intervention	Population - beyond 42 days postpartum
Fleming_1992	The effects of a social support group on depression, maternal attitudes and behavior in new mothers	Population - beyond 42 days postpartum
Fonseco_2019	Be a Mom, a WebBased Intervention to Prevent Postpartum Depression: The Enhancement of SelfRegulatory Skills and Its Association With Postpartum Depressive Symptoms	Population - beyond 42 days postpartum
Gammer_2020	A randomized controlled trial of an online, compassion-based intervention for maternal psychological well-being in the first year postpartum	Population - beyond 42 days postpartum
Giallo_2015	The efficacy of an intervention for the management of postpartum fatigue	Population - beyond 42 days postpartum
Golmakani_2015	The effect of pelvic floor muscle exercises program on sexual self-efficacy in primiparous women after delivery	Population - beyond 42 days postpartum
Gutke_2010	Specific muscle stabilizing as home exercises for persistent pelvic girdle pain after pregnancy: a randomized, controlled clinical trial	Population - beyond 42 days postpartum
Herring_2014	Using Technology to Promote Postpartum Weight Loss in Urban, Low-Income Mothers: A Pilot Randomized Controlled Trial	Population - beyond 42 days postpartum
Herring_2020	Feasibility of using a peer coach to deliver a behavioral intervention for promoting postpartum weight loss in Black and Latina mothers	Population - beyond 42 days postpartum
Kim_2021	Self-management mobile virtual reality program for women with gestational diabetes	Population - beyond 42 days postpartum
Koniak-Griffin_2003	Nurse visitation for adolescent mothers: two-year infant health and maternal outcomes	Population - beyond 42 days postpartum
Krummel_2010	Lessons Learned from the Mothers' Overweight Management Study in 4 West Virginia WIC Offices	Population - beyond 42 days postpartum
LeCheminant_2014	Effect of resistance training on body composition, self-efficacy, depression, and activity in postpartum women	Population - beyond 42 days postpartum
Mailey_2019	Is a general or specific exercise recommendation more effective for promoting physical activity among postpartum mothers?	Population - beyond 42 days postpartum
Mitchell_2018	Feasibility and acceptability of a brief online self-compassion intervention for mothers of infants	Population - beyond 42 days postpartum
Monteiro_2021	Be a Mom, a WebBased Intervention to Promote Positive Mental Health Among Postpartum Women With Low Risk for Postpartum Depression: Exploring Psychological Mechanisms of Change	Population - beyond 42 days postpartum
Morrell_2009	Clinical effectiveness of health visitor training in psychologically informed approaches for depression in postnatal women: pragmatic cluster randomised trial in primary care	Population - beyond 42 days postpartum

For peer review only - http://bmjopen.bmj.com/site/about/guidelines.xhtml

Author/Year	Title	Reasons for exclusion
Muijsers_2020	Blood pressure after PREeclampsia/HELLP by SELF monitoring (BP-PRESELF): Rationale and design of a multicenter randomized controlled trial	Population - beyond 42 days postpartum
Murphy_2017	Pilot trial of a parenting and self-care intervention for HIV-positive mothers: the IMAGE program	Population - beyond 42 days postpartum
Peacock_2015	A randomised controlled trial to delay or prevent type 2 diabetes after gestational diabetes: walking for exercise and nutrition to prevent diabetes for you	Population - beyond 42 days postpartum
Perez-Blasco_2013	Effects of a mindfulness-based intervention on psychological distress, well-being, and maternal self-efficacy in breast-feeding mothers: results of a pilot study	Population - beyond 42 days postpartum
Phelan_2017	Effect of an Internet-Based Program on Weight Loss for Low-Income Postpartum Women: a Randomized Clinical Trial	Population - beyond 42 days postpartum
Power_2019	Engagement and Weight Loss in a Web and Mobile Program for Low-Income Postpartum Women: fit Moms/Mamás Activas	Population - beyond 42 days postpartum
Rich-Edwards_2019	Randomized Trial to Reduce Cardiovascular Risk in Women with Recent Preeclampsia	Population - beyond 42 days postpartum
Rollo_2020	The feasibility and preliminary efficacy of an ehealth lifestyle program in women with recent gestational diabetes mellitus: a pilot study	Population - beyond 42 days postpartum
Sampson_2020	Reducing depression among mothers in a residential treatment setting: building evidence for the PST4PPD intervention	Population - beyond 42 days postpartum
Swanson_2009	Resolution of depression and grief during the first year after miscarriage: a randomized controlled clinical trial of couples-focused interventions	Population - beyond 42 days postpartum
Teychenne_2021	Mums on the Move: A pilot randomised controlled trial of a home-based physical activity intervention for mothers at risk of postnatal depression	Population - beyond 42 days postpartum
Timlin_2017	A preliminary randomised control trial of the effects of Dru yoga on psychological well-being in Northern Irish first time mothers	Population - beyond 42 days postpartum
Tripette_2014	Home-Based Active Video Games to Promote Weight Loss during the Postpartum Period	Population - beyond 42 days postpartum
Walker_2012	Ethnic-Specific Weight-Loss Interventions for Low-Income Postpartum Women: Findings and Lessons	Population - beyond 42 days postpartum
Watson_2005	The feasibility and effectiveness of pram walking groups for postpartum women in western Sydney	Population - beyond 42 days postpartum
Wilkinson_2015	Trial for Reducing Weight Retention in New Mums: a randomised controlled trial evaluating a low intensity, postpartum weight management programme	Population - beyond 42 days postpartum
Abbasi_2017	Comparing the effect of e-learning and educational booklet on the childbirth self-efficacy: a randomized controlled clinical trial	Population - not targeting pregnant women
Aleman_2017	Brief counseling on secondhand smoke exposure in pregnant women in Argentina and uruguay	Population - not targeting pregnant women
Althabe_2016	A Multifaceted Strategy to Implement Brief Smoking Cessation Counseling During Antenatal Care in Argentina and Uruguay: A Cluster Randomized Trial	Population - not targeting pregnant women
Baradaran-Akbarzadeh_2018	The effect of educational package on sexual function in cold temperament women of reproductive age	Population - not targeting pregnant women
Briggs_2016	A systematic review of interventions to improve knowledge and self-management skills concerning contraception, pregnancy and breastfeeding in people with rheumatoid arthritis	Population - not targeting pregnant women
Chomat_2019	Women's circles as a culturally safe psychosocial intervention in Guatemalan indigenous communities: a community-led pilot randomised trial	Population - not targeting pregnant women
DeNicola_2020	Telehealth Interventions to Improve Obstetric and Gynecologic Health Outcomes: A Systematic Review	Population - not targeting pregnant women
Fallin-Bennett_2018	Pilot Tobacco Treatment Intervention for Women in Residential Treatment for Substance Use Disorder	Population - not targeting pregnant women
Fisher_2018	Addressing multiple modifiable risks through structured community-based Learning Clubs to improve maternal and infant health and infant development in rural Vietnam: protocol for a parallel group cluster randomised controlled trial	Population - not targeting pregnant women
Ghetti_2019	Longitudinal Study of music Therapy's Effectiveness for Premature infants and their caregivers (LongSTEP): protocol for an international randomised trial	Population - not targeting pregnant women
Harvey_2008	Hygiene interventions for prevention of cytomegalovirus infection among childbearing women: Systematic review	Population - not targeting pregnant women
Hillemeier_2008	Improving women's preconceptional health: findings from a randomized trial of the Strong Healthy Women intervention in the Central Pennsylvania women's health study	Population - not targeting pregnant women
Johnson_2018	Protocol for the ROSE sustainment (ROSES) study, a sequential multiple assignment randomized trial to determine the minimum necessary intervention to maintain a postpartum depression prevention program in prenatal clinics serving low-income women	Population - not targeting pregnant women
Karimiankakolaki_2019	Designing an educational intervention on second-hand smoke in smoker men on the exposure of pregnant wives: a protocol for a randomized controlled trial	Population - not targeting pregnant women
Karyotaki_2021	The effects of psychological treatment of perinatal depression: an overview	Population - not targeting pregnant women
Mackintosh_2020	Interventions to increase patient and family involvement in escalation of care for acute life-threatening illness in community health and hospital settings	Population - not targeting pregnant women
Makaruk_2019	The Effectiveness of Regular Exercise Programs in the Prevention of Gestational Diabetes Mellitus-A Systematic Review	Population - not targeting pregnant women

For peer review only - http://bmjopen.bmj.com/site/about/guidelines.xhtml

Author/Year	Title	Reasons for exclusion
Matkin_2019	Telephone counselling for smoking cessation	Population - not targeting pregnant women
McRobbie_2016	Tackling obesity in areas of high social deprivation: clinical effectiveness and cost-effectiveness of a task-based weight management group programme - A randomised controlled trial and economic evaluation	Population - not targeting pregnant women
Mutale_2021	Addition of HIV self-test kits to partner notification services to increase HIV testing of male partners of pregnant women in Zambia: two parallel randomised trials	Population - not targeting pregnant women
Nayak_2019	Randomized Trial of an Innovative Electronic Screening and Brief Intervention for Reducing Drinking among Women of Childbearing Age	Population - not targeting pregnant women
Nguyen_2018	Engagement of Husbands in a Maternal Nutrition Program Substantially Contributed to Greater Intake of Micronutrient Supplements and Dietary Diversity during Pregnancy: Results of a Cluster-Randomized Program Evaluation in Bangladesh	Population - not targeting pregnant women
O'Dea_2015	Can the Onset of Type 2 Diabetes Be Delayed by a Group-Based Lifestyle Intervention in Women with Prediabetes following Gestational Diabetes Mellitus (GDM)? Findings from a Randomized Control Mixed Methods Trial	Population - not targeting pregnant women
Okeibunor_2011	Preventing malaria in pregnancy through community-directed interventions: Evidence from Akwa Ibom State, Nigeria	Population - not targeting pregnant women
Ovretveit_2021	Innovations in self care and close care made during COVID 19 pandemic: a narrative review	Population - not targeting pregnant women
Pettifor_2020	HIV self-testing among young women in rural South Africa: A randomized controlled trial comparing clinic-based HIV testing to the choice of either clinic testing or HIV self-testing with secondary distribution to peers and partners	Population - not targeting pregnant women
Pinchoff_2019	Evidence-Based Process for Prioritizing Positive Behaviors for Promotion: Zika Prevention in Latin America and the Caribbean and Applicability to Future Health Emergency Responses	Population - not targeting pregnant women
Pintye_2019	Acceptability and outcomes of distributing HIV selftests for male partner testing in Kenyan maternal and child health and family planning clinics	Population - not targeting pregnant women
Shen_2021	Clinical Evaluation of a Self-Testing Kit for Vaginal Infection Diagnosis	Population - not targeting pregnant women
Taylor_2019	Be Healthe for Your Heart: protocol for a Pilot Randomized Controlled Trial Evaluating a Web-Based Behavioral Intervention to Improve the Cardiovascular Health of Women With a History of Preeclampsia	Population - not targeting pregnant women
Tieu_2017	Preconception care for diabetic women for improving maternal and infant health	Population - not targeting pregnant women
van-der-Zwan_2019	The Effect of Heart Rate Variability Biofeedback Training on Mental Health of Pregnant and Non-Pregnant Women: a Randomized Controlled Trial	Population - not targeting pregnant women
Vézina-Im_2016	Implementation intentions intervention to promote fruit and vegetable intake in childbearing age women at risk for gestational diabetes mellitus: 6-month follow-up of a randomized controlled trial	Population - not targeting pregnant women
Youngkin_2010	Promoting self-care and secondary prevention in women's health: A study to test the accuracy of a home self-test system for bacterial vaginosis	Population - not targeting pregnant women
Adolfsson_2014	A web-based support for pregnant women and new mothers with type 1 diabetes mellitus in Sweden (MODIAB-Web): Study protocol for a randomized controlled trial	Protocol - results already included
Bais_2016	Bright light therapy in pregnant women with major depressive disorder: study protocol for a randomized, double-blind, controlled clinical trial	Protocol - results already included
Band_2019	Intervention planning and modification of the BUMP intervention: A digital intervention for the early detection of raised blood pressure in pregnancy	Protocol - results already included
Clifton_2016	Design of lifestyle intervention trials to prevent excessive gestational weight gain in women with overweight or obesity	Protocol - results already included
Cooper_2015	Pilot study to evaluate a tailored text message intervention for pregnant smokers (MiQuit): Study protocol for a randomised controlled trial	Protocol - results already included
Dodd_2011	Limiting weight gain in overweight and obese women during pregnancy to improve health outcomes: the LIMIT randomised controlled trial	Protocol - results already included
Dol_2019	Impact of mobile health interventions during the perinatal period for mothers in low- and middle-income countries: a systematic review protocol	Protocol - results already included
Evans_2019	Design of a novel digital intervention to promote healthy weight management among postpartum African American women	Protocol - results already included
Garmendia_2018	Effectiveness on maternal and offspring metabolic control of a home-based dietary counseling intervention and DHA supplementation in obese/overweight pregnant women (MIGHT study): A randomized controlled trial-Study protocol	Protocol - results already included
Griffiths_2016	Are digital interventions for smoking cessation in pregnancy effective? A systematic review protocol	Protocol - results already included
Grzeskowiak_2014	A randomized controlled trial to assess the clinical and cost effectiveness of a nurse-led Antenatal Asthma Management Service in South Australia (AAMS study)	Protocol - results already included
Jones_2012	Pain management for women in labour: an overview of systematic reviews	Protocol - results already included
Kinser_2020	Protocol for Pilot Study on Self-Management of Depressive Symptoms in Pregnancy	Protocol - results already included
Lim_2019	Smart Phone APP to Restore Optimal Weight (SPAROW): protocol for a randomised controlled trial for women with recent gestational diabetes	Protocol - results already included
Loukopoulou_2011	Design and study protocol of the maternal smoking cessation during pregnancy study, (M-SCOPE)	Protocol - results already included

For peer review only - http://bmjopen.bmj.com/site/about/guidelines.xhtml

Author/Year	Title	Reasons for exclusion
Lucas_2019	Promoting self-management of breast and nipple pain in breastfeeding women: Protocol of a pilot randomized controlled trial	Protocol - results already included
Miquelutti_2015	Developing strategies to be added to the protocol for antenatal care: an exercise and birth preparation program	Protocol - results already included
Navas_2018	Effectiveness and safety of moderate-intensity aerobic water exercise during pregnancy for reducing use of epidural analgesia during labor: protocol for a randomized clinical trial	Protocol - results already included
Sapkota_2019	Counselling-based psychosocial intervention to improve the mental health of abused pregnant women: a protocol for randomised controlled feasibility trial in a tertiary hospital in eastern Nepal	Protocol - results already included
Ternstrom_2017	A randomized controlled study comparing internet-based cognitive behavioral therapy and counselling by standard care for fear of birth - A study protocol	Protocol - results already included
Trevillion_2015	Depression: an exploratory parallel-group randomised controlled trial of antenatal guided self help for women (dawn trial)	Protocol - results already included
Whitemore_2019	Effectiveness and cost-effectiveness of a tailored text-message programme (MiQuit) for smoking cessation in pregnancy: Study protocol for a randomised controlled trial (RCT) and meta-analysis	Protocol - results already included
Willcox_2015	Testing the feasibility of a mobile technology intervention promoting healthy gestational weight gain in pregnant women (txt4two) - study protocol for a randomised controlled trial	Protocol - results already included
Zairina_2015	Study protocol for a randomised controlled trial evaluating the efficacy of a telehealth program - management of asthma with supportive telehealth of respiratory function in pregnancy (MASTERY©)	Protocol - results already included
Aslani_2014	Impact of Computer-Based Pregnancy-Induced Hypertension and Diabetes Decision Aids on Empowering Pregnant Women	Protocol - results not published
Baird_2016	Southampton PRegnancy Intervention for the Next Generation (SPRING): protocol for a randomised controlled trial	Protocol - results not published
Bei_2019	A scalable cognitive behavioural program to promote healthy sleep during pregnancy and postpartum periods: Protocol of a randomised controlled trial (the SEED project)	Protocol - results not published
Browne_2015	Uterine Tonus Assessment by Midwives versus Patient self-assessment in the active management of the third stage of labor (UTAMP): Study protocol for a randomized controlled trial	Protocol - results not published
Caramlau_2011	Mums 4 Mums: structured telephone peer-support for women experiencing postnatal depression Pilot and exploratory RCT of its clinical and cost effectiveness	Protocol - results not published
Carolan-Olah_2015	Development and initial testing of a GDM information website for multi-ethnic women with GDM	Protocol - results not published
Chang_2015	Electronic personal maternity records: Both web and smartphone services	Protocol - results not published
Dallosso_2018	Baby Steps - A structured group education programme with accompanying mobile web application designed to promote physical activity in women with a history of gestational diabetes: Study protocol for a randomised controlled trial	Protocol - results not published
Davis_2018	Optimizing Gestational Weight Gain With the Eating4Two Smartphone App: Protocol for a Randomized Controlled Trial	Protocol - results not published
Edwards_2019	Can an educational web-intervention, co-created by service users alongside self-efficacy theory, affect nulliparous women's experiences of early labour? A study protocol for a randomised control trial (the L-TEL Trial)	Protocol - results not published
Hawkins_2019	Feasibility of a Sleep Self-Management Intervention in Pregnancy Using a Personalized Health Monitoring Device: Protocol for a Pilot Randomized Controlled Trial	Protocol - results not published
Koushede_2013	Group-based antenatal birth and parent preparation for improving birth outcomes and parenting resources: study protocol for a randomised trial	Protocol - results not published
LeBlanc_2016	Prepare, a randomized trial to promote and evaluate weight loss among overweight and obese women planning pregnancy: Study design and rationale	Protocol - results not published
Lenz_2018	Mindfulness-based Stress Reduction in Pregnancy: an App-Based Programme to Improve the Health of Mothers and Children (MINDFUL/PMI Study)	Protocol - results not published
Martin_2018	A pilot randomized controlled trial of a remotely-delivered behavioral health coaching program to limit weight gain in pregnancy and reduce postpar-tum weight retention	Protocol - results not published
Monteiro_2019	Non-pharmacological interventions for treating sexual dysfunction in postpartum women: A systematic review protocol	Protocol - results not published
Nagle_2013	Primary prevention of gestational diabetes for women who are overweight and obese: a randomised controlled trial	Protocol - results not published
Nwolise_2017	Exploring the acceptability and feasibility of a preconception and diabetes information app for women with pregestational diabetes: A mixed-methods study protocol	Protocol - results not published
Pallitto_2016	Testing a counselling intervention in antenatal care for women experiencing partner violence: a study protocol for a randomized controlled trial in Johannesburg, South Africa	Protocol - results not published
Pascual-Morena_2019	Exercise vs metformin for gestational diabetes mellitus: Protocol for a network meta-analysis	Protocol - results not published
Patten_2019	The Healthy Pregnancies Project: Study protocol and baseline characteristics for a cluster-randomized controlled trial of a community intervention to reduce tobacco use among Alaska Native pregnant women	Protocol - results not published
Pelaez-Crisologo_2009	Different techniques of blood glucose monitoring in women with gestational diabetes for improving maternal and infant health	Protocol - results not published
Prabhu_2020	The effect of a prenatal psychosocial education program on postnatal depression, stress, and parenting self-efficacy in women in South India - A study protocol for a randomised controlled trial	Protocol - results not published

For peer review only - http://bmjopen.bmj.com/site/about/guidelines.xhtml

Author/Year	Title	Reasons for exclusion
Qian_2021	Effectiveness of non-pharmacological interventions for reducing postpartum fatigue: a systematic review protocol	Protocol - results not published
Reynolds_2016	MAHILA: a protocol for evaluating a nurse-delivered mHealth intervention for women with HIV and psychosocial risk factors in India	Protocol - results not published
Roberts_2004	Protocol for a randomised controlled trial of a decision aid for the management of pain in labour and childbirth	Protocol - results not published
Sacristan-Martin_2019	A mindfulness and compassion-based program applied to pregnant women and their partners to decrease depression symptoms during pregnancy and postpartum: study protocol for a randomized controlled trial	Protocol - results not published
Seo_2021	The Development of the Postpartum Depression Self-Management Mobile Application "happy Mother"	Protocol - results not published
Singh_2017	Internet-delivered blood glucose awareness training (BGAT) for women with type 1 diabetes (T1D) contemplating pregnancy: results from the Bump2be trial	Protocol - results not published
Tielsch_2015	Designs of two randomized, community-based trials to assess the impact of alternative cookstove installation on respiratory illness among young children and reproductive outcomes in rural Nepal	Protocol - results not published
Tomfohr-Madsen_2016	Mindfulness-based cognitive therapy for psychological distress in pregnancy: study protocol for a randomized controlled trial	Protocol - results not published
Tomlinson_2011	An effectiveness study of an integrated, community-based package for maternal, newborn, child and HIV care in South Africa: study protocol for a randomized controlled trial	Protocol - results not published
Veringa_2016	I've Changed My Mind', Mindfulness-Based Childbirth and Parenting (MBCP) for pregnant women with a high level of fear of childbirth and their partners: study protocol of the quasi-experimental controlled trial	Protocol - results not published
Volrathongchai_2013	Non-pharmacological interventions for women with postpartum fatigue	Protocol - results not published
Wilcox_2018	A randomized controlled trial to prevent excessive gestational weight gain and promote postpartum weight loss in overweight and obese women: Health In Pregnancy and Postpartum (HIPP)	Protocol - results not published
Yeung_2019	Birth ball for pregnant women in labour research protocol: A multi-centre randomised controlled trial	Protocol - results not published

For peer review only

Supplementary File S7. Reference list of included studies

1. Aaronson NK, Ershoff DH, Danaher BG: Smoking cessation in pregnancy: a self-help approach. *Addict Behav* 1985, 10(1):103-108.
2. Abdel-Aleem H, Singata M, Abdel-Aleem M, Mshweshwe N, Williams X, Hofmeyr GJ: Uterine massage to reduce postpartum hemorrhage after vaginal delivery. *Int J Gynaecol Obstet* 2010, 111(1):32-36.
3. Abedi P, Jahanfar S, Namvar F, Lee J: Breastfeeding or nipple stimulation for reducing postpartum haemorrhage in the third stage of labour. In., vol. 2016; 2016.
4. Adams J, Frawley J, Steel A, Broom A, Sibbritt D: Use of pharmacological and non-pharmacological labour pain management techniques and their relationship to maternal and infant birth outcomes: examination of a nationally representative sample of 1835 pregnant women. *Midwifery* 2015, 31(4):458-463.
5. Adler SP, Finney JW, Manganello AM, Best AM: Prevention of child-to-mother transmission of cytomegalovirus among pregnant women. *J Pediatr* 2004, 145(4):485-491.
6. Afravi S, Abbaspoor Z, Montazeri S, Cheraghian B: The effect of Hugo point pressure on postpartum pain in multiparous women. *Family Medicine & Primary Care Review* 2019, 21(1):7-11.
7. Ahmadpour P, Mosavi S, Mohammad-Alizadeh-Charandabi S, Jahanfar S, Mirghafourvand M: Evaluation of the birth plan implementation: a parallel convergent mixed study. *Reprod Health* 2020, 17(1):138.
8. Ajibaye O, Balogun EO, Olukosi YA, Orok BA, Oyebola KM, Iwalokun BA, Aina OO, Shittu O, Adeneye AK, Ojewunmi OO *et al*: Impact of training of mothers, drug shop attendants and voluntary health workers on effective diagnosis and treatment of malaria in Lagos, Nigeria. *Trop Parasitol* 2019, 9(1):36-44.
9. Ajuzieogu OV, Amucheazi A, Ezike HA, Achi J, Abam DS: The efficacy of chewing gum on postoperative ileus following cesarean section in Enugu, South East Nigeria: A randomized controlled clinical trial. *Niger J Clin Pract* 2014, 17(6):739-742.
10. Akselsson A, Lindgren H, Georgsson S, Pettersson K, Steineck G, Skokic V, Radestad I: Mindfetalness to increase women's awareness of fetal movements and pregnancy outcomes: a cluster-randomised controlled trial including 39 865 women. *BJOG* 2020, 127(7):829-837.
11. Al Hashmi I, Nandy K, Seshan V: Non-medical strategies to improve pregnancy outcomes of women with gestational diabetes mellitus: a literature review. In., vol. 19; 2019.
12. Al Khamis S, Asimakopoulou K, Newton T, Daly B: The effect of dental health education on pregnant women's adherence with toothbrushing and flossing - A randomized control trial. *Community Dent Oral Epidemiol* 2017, 45(5):469-477.
13. Al-Hashmi I, Hodge F, Nandy K, Thomas E, Brecht ML: The Effect of a Self-Efficacy-Enhancing Intervention on Perceived Self-Efficacy and Actual Adherence to Healthy Behaviours Among Women with Gestational Diabetes Mellitus. *Sultan Qaboos Univ Med J* 2018, 18(4):e513-e519.
14. Alekseev NP, Vladimir, II, Nadezhda TE: Pathological postpartum breast engorgement: prediction, prevention, and resolution. *Breastfeed Med* 2015, 10(4):203-208.
15. Alexander DA, Northcross A, Karrison T, Morhasson-Bello O, Wilson N, Atalabi OM, Dutta A, Adu D, Ibigbami T, Olamijulo J *et al*: Pregnancy outcomes and ethanol cook stove intervention: A randomized-controlled trial in Ibadan, Nigeria. *Environ Int* 2018, 111:152-163.
16. Alfadhli E, Osman E, Basri T: Use of a real time continuous glucose monitoring system as an educational tool for patients with gestational diabetes. *Diabetol Metab Syndr* 2016, 8(1):48.
17. Alizadeh S, Riazi H, Majd HA, Ozgoli G: The effect of sexual health education on sexual activity, sexual quality of life, and sexual violence in pregnancy: a prospective randomized controlled trial. *BMC Pregnancy Childbirth* 2021, 21(1):334.
18. Alnuaimi K, Abuidhail J, Abuzaid H: The effects of an educational programme about preeclampsia on women's awareness: a randomised control trial. *Int Nurs Rev* 2020, 67(4):501-511.

For peer review only - http://bmjopen.bmj.com/site/about/guidelines.xhtml

19. Altazan AD, Redman LM, Burton JH, Beyl RA, Cain LE, Sutton EF, Martin CK: Mood and quality of life changes in pregnancy and postpartum and the effect of a behavioral intervention targeting excess gestational weight gain in women with overweight and obesity: a parallel-arm randomized controlled pilot trial. *BMC Pregnancy Childbirth* 2019, 19(1):50.
20. Ambardekar S, Shochet T, Bracken H, Coyaji K, Winikoff B: Calibrated delivery drape versus indirect gravimetric technique for the measurement of blood loss after delivery: a randomized trial. *BMC Pregnancy Childbirth* 2014, 14(1):276.
21. Anderson L, Kynoch K, Kildea S, Lee N: Effectiveness of breast massage for the treatment of women with breastfeeding problems: a systematic review. *JBI Database System Rev Implement Rep* 2019, 17(8):1668-1694.
22. Anderson NH, Gordon A, Li M, Cronin RS, Thompson JMD, Raynes-Greenow CH, Heazell AEP, Stacey T, Culling VM, Wilson J *et al*: Association of Supine Going-to-Sleep Position in Late Pregnancy With Reduced Birth Weight: A Secondary Analysis of an Individual Participant Data Meta-analysis. *JAMA Netw Open* 2019, 2(10):e1912614.
23. Antoszewski B, Sobczak M, Kasielska-Trojan A: Self-assessment of striae gravidarum prophylaxis. *Postepy Dermatol Alergol* 2015, 32(6):459-464.
24. Aprilina HD, Krislinggardini K, Isnaini N, Suratmi S: The Effect of Cabbage Leaves Compress on Breast Engorgement in Postpartum Mother. *Open Access Macedonian Journal of Medical Sciences* 2021, 9(T4):124-128.
25. Araban M, Baharzadeh K, Karimy M: Nutrition modification aimed at enhancing dietary iron and folic acid intake: an application of health belief model in practice. *Eur J Public Health* 2017, 27(2):287-292.
26. Araban M, Tavafian SS, Zarandi SM, Hidarnia AR, Burri A, Montazeri A: A behavioral strategy to minimize air pollution exposure in pregnant women: a randomized controlled trial. *Environ Health Prev Med* 2017, 22(1):26.
27. Ardilouze A, Bouchard P, Hivert MF, Simard C, Allard C, Garant MP, Menard J, Ouellet A, Houde G, Pesant MH *et al*: Self-Monitoring of Blood Glucose: A Complementary Method Beyond the Oral Glucose Tolerance Test to Identify Hyperglycemia During Pregnancy. *Can J Diabetes* 2019, 43(8):627-635.
28. Arimori N: Randomized controlled trial of decision aids for women considering prenatal testing: The effect of the Ottawa Personal Decision Guide on decisional conflict. *Japan Journal of Nursing Science* 2006, 3(2):119-130.
29. Arthur C, Di Corleto E, Ballard E, Kothari A: A randomized controlled trial of daily weighing in pregnancy to control gestational weight gain. *BMC Pregnancy Childbirth* 2020, 20(1):223.
30. Asci O, Rathfisch G: Effect of lifestyle interventions of pregnant women on their dietary habits, lifestyle behaviors, and weight gain: a randomized controlled trial. *J Health Popul Nutr* 2016, 35:7.
31. Ashorn P, Alho L, Ashorn U, Cheung YB, Dewey KG, Harjunmaa U, Lartey A, Nkhoma M, Phiri N, Phuka J *et al*: The impact of lipid-based nutrient supplement provision to pregnant women on newborn size in rural Malawi: a randomized controlled trial. *Am J Clin Nutr* 2015, 101(2):387-397.
32. Ashworth DC, Maule SP, Stewart F, Nathan HL, Shennan AH, Chappell LC: Setting and techniques for monitoring blood pressure during pregnancy. In., vol. 2020; 2020.
33. Avery MD, Leon AS, Kopher RA: Effects of a partially home-based exercise program for women with gestational diabetes. *Obstet Gynecol* 1997, 89(1):10-15.
34. Avsar TS, McLeod H, Jackson L: Health outcomes of smoking during pregnancy and the postpartum period: an umbrella review. *BMC Pregnancy Childbirth* 2021, 21(1):254.
35. Bagharpoosh M, Sangestani G, Goodarzi M: Effect of progressive muscle relaxation technique on pain relief during labor. *Acta Medica Iranica* 2006, 44(3).
36. Bais B, Kamperman AM, Bijma HH, Hoogendijk WJ, Souman JL, Knijff E, Lambregtse-van den Berg MP: Effects of bright light therapy for depression during pregnancy: a randomised, double-blind controlled trial. *BMJ Open* 2020, 10(10):e038030.

For peer review only - http://bmjopen.bmj.com/site/about/guidelines.xhtml

37. Balami AD, Said SM, Zulkefli NAM, Norsa'adah B, Audu B: Improving malaria preventive practices and pregnancy outcomes through a health education intervention: A randomized controlled trial. *Malar J* 2021, 20(1):55.

38. Bao H, Yu P, Song X, Zhou Y, Zhu Y, Xu X: The influence of home-based exercise on gestational diabetes: a meta-analysis of randomized controlled trials. *J Matern Fetal Neonatal Med* 2020, 33(13):2300-2305.

39. Barakat R, Vargas M, Brik M, Fernandez I, Gil J, Coteron J, Santacruz B: Does Exercise During Pregnancy Affect Placental Weight?: A Randomized Clinical Trial. *Eval Health Prof* 2018, 41(3):400-414.

40. Barber V, Calvert A, Vandrevala T, Star C, Khalil A, Griffiths P, Heath PT, Jones CE: Prevention of Acquisition of Cytomegalovirus Infection in Pregnancy through Hygiene-based Behavioral Interventions: A Systematic Review and Gap Analysis. In.; 2020.

41. Barroso CS, Yockey A, Degon E, Gautam Poudel P, Brown SD, Hedderson MM, Moreno-Hunt C, Ehrlich SF: Efficacious lifestyle interventions for appropriate gestational weight gain in women with overweight or obesity set in the health care system: a scoping review. In.; 2021.

42. Bartu A, Sharp J, Ludlow J, Doherty DA: Postnatal home visiting for illicit drug-using mothers and their infants: a randomised controlled trial. *Aust N Z J Obstet Gynaecol* 2006, 46(5):419-426.

43. Baruth M, Schlaff RA, Deere S, Walker JL, Dressler BL, Wagner SF, Boggs A, Simon HA: The Feasibility and Efficacy of a Behavioral Intervention to Promote Appropriate Gestational Weight Gain. *Matern Child Health J* 2019, 23(12):1604-1612.

44. Bastani F, Hidarnia A, Kazemnejad A, Vafaei M, Kashanian M: A randomized controlled trial of the effects of applied relaxation training on reducing anxiety and perceived stress in pregnant women. *J Midwifery Womens Health* 2005, 50(4):e36-40.

45. Bastani FF, Sharami SH, Aski SK, Milani F, Khakifirooz B, Shakiba M, Attari SM, Omidkhah T: The Effect of Simultaneous Application of Transcutaneous Electrical Nerve Stimulation (TENS) on Specific Lumbar and Acupuncture Points on Labour Pain Relief: A Randomised Clinical Trial. *Journal of Clinical and Diagnostic Research* 2020.

46. Bastiaenen CH, de Bie RA, Vlaeyen JW, Goossens ME, Leffers P, Wolters PM, Bastiaanssen JM, Brandt PA, Essed GG: Long-term effectiveness and costs of a brief self-management intervention in women with pregnancy-related low back pain after delivery. *BMC Pregnancy Childbirth* 2008, 8:19.

47. Batra P, Mangione CM, Cheng E, Steers WN, Nguyen TA, Bell D, Kuo AA, Gregory KD: A Cluster Randomized Controlled Trial of the MyFamilyPlan Online Preconception Health Education Tool. *Am J Health Promot* 2018, 32(4):897-905.

48. Baumel A, Tinkelman A, Mathur N, Kane JM: Digital Peer-Support Platform (7Cups) as an Adjunct Treatment for Women With Postpartum Depression: Feasibility, Acceptability, and Preliminary Efficacy Study. *JMIR Mhealth Uhealth* 2018, 6(2):e38.

49. Beckmann MM, Stock OM: Antenatal perineal massage for reducing perineal trauma. *Cochrane Database Syst Rev* 2013(4):CD005123.

50. Beetham KS, Giles C, Noetel M, Clifton V, Jones JC, Naughton G: The effects of vigorous intensity exercise in the third trimester of pregnancy: A systematic review and meta-analysis. In., vol. 19; 2019.

51. Beleza ACS, Ferreira C, Meirelles M, Santos C, Nakanno AM: The effect of cryotherapy in perineal pain after vaginal childbirth with episiotomy. *Physiotherapy (united kingdom)* 2011, 97.

52. Bellussi F, Po G, Livi A, Saccone G, De Vivo V, Oliver EA, Berghella V: Fetal Movement Counting and Perinatal Mortality: A Systematic Review and Meta-analysis. *Obstet Gynecol* 2020, 135(2):453-462.

53. Berghella V, Di Mascio D: Evidence-based labor management: before labor (Part 1). In., vol. 2; 2020.

54. Bergman E, Axelsson O, Petzold M, Sonesson C, Kieler H: Self-administered symphysis-fundus measurements analyzed with a novel statistical method for detection of intrauterine growth restriction: a clinical evaluation. *Acta Obstet Gynecol Scand* 2011, 90(8):890-896.

For peer review only - http://bmjopen.bmj.com/site/about/guidelines.xhtml

55. Bergstrom S: External cephalic version and daily post-versional maternal self-assessment of fetal presentation. A prospective study. *Gynecol Obstet Invest* 1992, 33(1):15-18.

56. Berta M, Lindgren H, Christensson K, Mekonnen S, Adefris M: Effect of maternal birth positions on duration of second stage of labor: systematic review and meta-analysis. *BMC Pregnancy Childbirth* 2019, 19(1):466.

57. Bhutta ZA, Rizvi A, Raza F, Hotwani S, Zaidi S, Moazzam Hossain S, Soofi S, Bhutta S: A comparative evaluation of multiple micronutrient and iron-folic acid supplementation during pregnancy in Pakistan: impact on pregnancy outcomes. *Food Nutr Bull* 2009, 30(4 Suppl):S496-505.

58. Biana CB, Cecagno D, Porto AR, Cecagno S, Marques VA, Soares MC: Non-pharmacological therapies applied in pregnancy and labor: an integrative review. *Rev Esc Enferm USP* 2021, 55:e03681.

59. Bilgin CN, Ak B, Ayhan F, Kocyigit F, Yorgun S, Topcuoglu MA: Effect of childbirth education on the perceptions of childbirth and breastfeeding self-efficacy and the obstetric outcomes of nulliparous women. *Health Care Women Int* 2020, 41(2):188-204.

60. Bitzer EM, Schneider A, Wenzlaff P, Hoyme UB, Siegmund-Schultze E: Self-testing of vaginal pH to prevent preterm delivery: a controlled trial. *Dtsch Arztebl Int* 2011, 108(6):81-86.

61. Bornstein J, Ohel G, Sorokin Y, Reape KZ, Shnaider O, Kessary-Shoham H, Ophir E: Effectiveness of a novel home-based testing device for the detection of rupture of membranes. *Am J Perinatol* 2009, 26(1):45-50.

62. Boryri T, Navidian A, Zehi F: Assessing the effect of self-care education on anxiety and depression among pregnant women with a history of spontaneous abortion. In., vol. 9; 2020.

63. Bowen L, Pealing L, Tucker K, McManus RJ, Chappell LC: Adherence with blood pressure self-monitoring in women with pregnancy hypertension, and comparisons to clinic readings: A secondary analysis of OPTIMUM-BP. *Pregnancy Hypertens* 2021, 25:68-74.

64. Brennan M, Young G, Devane D: Topical preparations for preventing stretch marks in pregnancy. *Cochrane Database Syst Rev* 2012, 11:CD000066.

65. Bright KS, Mughal MK, Wajid A, Lane-Smith M, Murray L, Roy N, Van Zanten SV, McNeil DA, Stuart S, Kingston D: Internet-based interpersonal psychotherapy for stress, anxiety, and depression in prenatal women: study protocol for a pilot randomized controlled trial. *Trials* 2019, 20(1):814.

66. Brik M, Fernandez-Buhigas I, Martin-Arias A, Vargas-Terrones M, Barakat R, Santacruz B: Does exercise during pregnancy impact on maternal weight gain and fetal cardiac function? A randomized controlled trial. *Ultrasound Obstet Gynecol* 2019, 53(5):583-589.

67. Brown J, Alwan NA, West J, Brown S, McKinlay CJD, Farrar D, Crowther CA: Lifestyle interventions for the treatment of women with gestational diabetes. In., vol. 2017; 2017.

68. Brustman LE, Langer O, Anyaegbunam A, Belle C, Merkatz IR: Education does not improve patient perception of preterm uterine contractility. *International Journal of Gynecology & Obstetrics* 1991, 34(3):290-290.

69. Buppasiri P, Lumbiganon P, Thinkhamrop J, Ngamjarus C, Laopaiboon M, Medley N: Calcium supplementation (other than for preventing or treating hypertension) for improving pregnancy and infant outcomes. In., vol. 2015; 2015.

70. Byrne J, Hauck Y, Fisher C, Bayes S, Schutze R: Effectiveness of a Mindfulness-Based Childbirth Education pilot study on maternal self-efficacy and fear of childbirth. *J Midwifery Womens Health* 2014, 59(2):192-197.

71. Cahill AG, Haire-Joshu D, Cade WT, Stein RI, Woolfolk CL, Moley K, Mathur A, Schechtman K, Klein S: Weight Control Program and Gestational Weight Gain in Disadvantaged Women with Overweight or Obesity: A Randomized Clinical Trial. *Obesity (Silver Spring)* 2018, 26(3):485-491.

72. Callaghan S: The role of fetal movement counting and 'kick charts' to reduce stillbirths in pregnancies ≥28 weeks' gestation. In., vol. 26; 2018.

73. Callaway LK, McIntyre HD, Barrett HL, Foxcroft K, Tremellen A, Lingwood BE, Tobin JM, Wilkinson S, Kothari A, Morrison M *et al*: Probiotics for the Prevention of Gestational Diabetes Mellitus in Overweight and Obese Women: Findings From the SPRING Double-Blind Randomized Controlled Trial. *Diabetes Care* 2019, 42(3):364-371.

For peer review only - http://bmjopen.bmj.com/site/about/guidelines.xhtml

74. Can HO, Saruhan A: Evaluation of the effects of ice massage applied to large intestine 4 (hegu) on postpartum pain during the active phase of labor. *Iran J Nurs Midwifery Res* 2015, 20(1):129-138.

75. Cankaya S, Simsek B: Effects of Antenatal Education on Fear of Birth, Depression, Anxiety, Childbirth Self-Efficacy, and Mode of Delivery in Primiparous Pregnant Women: A Prospective Randomized Controlled Study. *Clin Nurs Res* 2021, 30(6):818-829.

76. Carducci B, Keats EC, Bhutta ZA: Zinc supplementation for improving pregnancy and infant outcome. In., vol. 2021; 2021.

77. Carlson LM, Harris S, Hardisty EE, Hocutt G, Vargo D, Campbell E, Davis E, Gilmore K, Vora NL: Use of a novel computerized decision aid for aneuploidy screening: a randomized controlled trial. *Genet Med* 2019, 21(4):923-929.

78. Carrascosa MDC, Navas A, Artigues C, Ortas S, Portells E, Soler A, Bennasar-Veny M, Leiva A, Aquanatal T: Effect of aerobic water exercise during pregnancy on epidural use and pain: A multi-centre, randomised, controlled trial. *Midwifery* 2021, 103:103105.

79. Chamberlain C, O'Mara-Eves A, Porter J, Coleman T, Perlen SM, Thomas J, McKenzie JE: Psychosocial interventions for supporting women to stop smoking in pregnancy. In., vol. 2017; 2017.

80. Chang G, McNamara TK, Orav EJ, Koby D, Lavigne A, Ludman B, Vincitorio NA, Wilkins-Haug L: Brief intervention for prenatal alcohol use: a randomized trial. *Obstet Gynecol* 2005, 105(5 Pt 1):991-998.

81. Chankhunaphas W, Charoenkwan K: Effect of elastic abdominal binder on pain and functional recovery after caesarean delivery: a randomised controlled trial. *J Obstet Gynaecol* 2020, 40(4):473-478.

82. Chao AS, Chao A, Wang TH, Chang YC, Peng HH, Chang SD, Chao A, Chang CJ, Lai CH, Wong AMK: Pain relief by applying transcutaneous electrical nerve stimulation (TENS) on acupuncture points during the first stage of labor: a randomized double-blind placebo-controlled trial. *Pain* 2007, 127(3):214-220.

83. Charkamyani F, Hosseinkhani A, Neisani Samani L, Khedmat L: Reducing the Adverse Maternal and Fetal Outcomes in IVF Women by Exercise Interventions During Pregnancy. *Res Q Exerc Sport* 2019, 90(4):589-599.

84. Chatzakis C, Goulis DG, Mareti E, Eleftheriades M, Zavlanos A, Dinas K, Sotiriadis A: Prevention of gestational diabetes mellitus in overweight or obese pregnant women: A network meta-analysis. In., vol. 158; 2019.

85. Chen I, Opiyo N, Tavender E, Mortazhejri S, Rader T, Petkovic J, Yogasingam S, Taljaard M, Agarwal S, Laopaiboon M *et al*: Non-clinical interventions for reducing unnecessary caesarean section. *Cochrane Database Syst Rev* 2018, 9:CD005528.

86. Chen M, Chang Q, Duan T, He J, Zhang L, Liu X: Uterine massage to reduce blood loss after vaginal delivery: a randomized controlled trial. *Obstet Gynecol* 2013, 122(2 Pt 1):290-295.

87. Chen Y, Yue R, Zhang B, Li Z, Shui J, Huang X: Effects of probiotics on blood glucose, biomarkers of inflammation and oxidative stress in pregnant women with gestational diabetes mellitus: A meta-analysis of randomized controlled trials. *Med Clin (Barc)* 2020, 154(6):199-206.

88. Chi YC, Wu CL, Chen CY, Lyu SY, Lo FE, Morisky DE: Randomized trial of a secondhand smoke exposure reduction intervention among hospital-based pregnant women. *Addict Behav* 2015, 41:117-123.

89. Chirakalwasan N, Amnakkittikul S, Wanitcharoenkul E, Charoensri S, Saetung S, Chanprasertyothin S, Chailurkit LO, Panburana P, Bumrungphuet S, Thakkinstian A *et al*: Continuous Positive Airway Pressure Therapy in Gestational Diabetes With Obstructive Sleep Apnea: A Randomized Controlled Trial. *J Clin Sleep Med* 2018, 14(3):327-336.

90. Choi HS, Lee KE, Shin Y: Effects of an emotional regulation program on maladaptive cognitive emotion regulation, stress, anxiety, and postpartum depression among South Korean mothers of premature infants in the neonatal intensive care unit. *Child Health Nurs Res* 2021, 27(1):24-33.

91. Choi J, Lee JH, Vittinghoff E, Fukuoka Y: mHealth Physical Activity Intervention: A Randomized Pilot Study in Physically Inactive Pregnant Women. *Matern Child Health J* 2016, 20(5):1091-1101.

For peer review only - http://bmjopen.bmj.com/site/about/guidelines.xhtml

92. Christian P, Klemm R, Shamim AA, Ali H, Rashid M, Shaikh S, Wu L, Mehra S, Labrique A, Katz J *et al*: Effects of vitamin A and beta-carotene supplementation on birth size and length of gestation in rural Bangladesh: a cluster-randomized trial. *Am J Clin Nutr* 2013, 97(1):188-194.

93. Chughtai A, Navaee M, Hadi M, Yaghoubinia F: Comparing the Effect of Aromatherapy With Essential Oils of Rosa damascena and Lavender Alone and in Combination on Severity of Pain in the First Phase of Labor in Primiparous Women. 2018.

94. Clasen T, Checkley W, Peel JL, Balakrishnan K, McCracken JP, Rosa G, Thompson LM, Barr DB, Clark ML, Johnson MA *et al*: Design and Rationale of the HAPIN Study: A Multicountry Randomized Controlled Trial to Assess the Effect of Liquefied Petroleum Gas Stove and Continuous Fuel Distribution. *Environ Health Perspect* 2020, 128(4):47008.

95. Cluett ER, Burns E, Cuthbert A: Immersion in water during labour and birth. In., vol. 2018; 2018.

96. Coentro VS, Perrella SL, Lai CT, Rea A, Murray K, Geddes DT: Impact of Nipple Shield Use on Milk Transfer and Maternal Nipple Pain. *Breastfeed Med* 2021, 16(3):222-229.

97. Coleman J, Okere M, Seffah J, Kember A, O'Brien LM, Borazjani A, Butler M, Wells J, MacRitchie S, Isaac A *et al*: The Ghana PrenaBelt trial: a double-blind, sham-controlled, randomised clinical trial to evaluate the effect of maternal positional therapy during third-trimester sleep on birth weight. *BMJ Open* 2019, 9(4):e022981.

98. Coleman T, Clark M, Welch C, Whitemore R, Leonardi-Bee J, Cooper S, Hewitt C, Jones M, Sutton S, Watson J *et al*: Effectiveness of offering tailored text message, self-help smoking cessation support to pregnant women who want information on stopping smoking: MiQuit3 randomised controlled trial and meta-analysis. *Addiction* 2022, 117(4):1079-1094.

99. Crawford TJ, Crowther CA, Alsweiler J, Brown J: Antenatal dietary supplementation with myo-inositol in women during pregnancy for preventing gestational diabetes. In., vol. 2015; 2015.

100. Crepinsek MA, Taylor EA, Michener K, Stewart F: Interventions for preventing mastitis after childbirth. In., vol. 2020; 2020.

101. Crockett K, Zlotnick C, Davis M, Payne N, Washington R: A depression preventive intervention for rural low-income African-American pregnant women at risk for postpartum depression. *Arch Womens Ment Health* 2008, 11(5-6):319-325.

102. Cronin RS, Li M, Thompson JMD, Gordon A, Raynes-Greenow CH, Heazell AEP, Stacey T, Culling VM, Bowring V, Anderson NH *et al*: An Individual Participant Data Meta-analysis of Maternal Going-to-Sleep Position, Interactions with Fetal Vulnerability, and the Risk of Late Stillbirth. *EClinicalMedicine* 2019, 10:49-57.

103. Czech I, Fuchs P, Fuchs A, Lorek M, Tobolska-Lorek D, Drosdzol-Cop A, Sikora J: Pharmacological and Non-Pharmacological Methods of Labour Pain Relief-Establishment of Effectiveness and Comparison. *Int J Environ Res Public Health* 2018, 15(12).

104. D'Almeida A, Carter JP, Anatol A, Prost C: Effects of a combination of evening primrose oil (gamma linolenic acid) and fish oil (eicosapentaenoic + docahexaenoic acid) versus magnesium, and versus placebo in preventing pre-eclampsia. *Women Health* 1992, 19(2-3):117-131.

105. da Silva SG, Hallal PC, Domingues MR, Bertoldi AD, Silveira MFD, Bassani D, da Silva ICM, da Silva BGC, Coll CVN, Evenson K: A randomized controlled trial of exercise during pregnancy on maternal and neonatal outcomes: results from the PAMELA study. *Int J Behav Nutr Phys Act* 2017, 14(1):175.

106. Dabiri F, Shahi A: The Effect of LI4 Acupressure on Labor Pain Intensity and Duration of Labor: A Randomized Controlled Trial. *Oman Med J* 2014, 29(6):425-429.

107. Dahlen HG, Homer CS, Cooke M, Upton AM, Nunn R, Brodrick B: Perineal outcomes and maternal comfort related to the application of perineal warm packs in the second stage of labor: a randomized controlled trial. *Birth* 2007, 34(4):282-290.

108. Dai L, Shen Q, Redding SR, Ouyang YQ: Simulation-based childbirth education for Chinese primiparas: A pilot randomized controlled trial. *Patient Educ Couns* 2021, 104(9):2266-2274.

For peer review only - http://bmjopen.bmj.com/site/about/guidelines.xhtml

109. Daley AJ, Macarthur C, Winter H: The role of exercise in treating postpartum depression: a review of the literature. *J Midwifery Womens Health* 2007, 52(1):56-62.

110. Dashtinejad E, Abedi P, Afshari P: Comparison of the effect of breast pump stimulation and oxytocin administration on the length of the third stage of labor, postpartum hemorrhage, and anemia: a randomized controlled trial. *BMC Pregnancy Childbirth* 2018, 18(1):293.

111. Davidson SJ, Barrett HL, Price SA, Callaway LK, Dekker Nitert M: Probiotics for preventing gestational diabetes. In., vol. 2021; 2021.

112. de Vries H, Bakker M, Mullen PD, van Breukelen G: The effects of smoking cessation counseling by midwives on Dutch pregnant women and their partners. *Patient Educ Couns* 2006, 63(1-2):177-187.

113. Dehcheshmeh FS, Rafiei H: Complementary and alternative therapies to relieve labor pain: A comparative study between music therapy and Hoku point ice massage. *Complement Ther Clin Pract* 2015, 21(4):229-232.

114. Deliktas A, Kukulu K: A meta-analysis of the effect on maternal health of upright positions during the second stage of labour, without routine epidural analgesia. In., vol. 74; 2018.

115. Demirel G, Guler H: The Effect of Uterine and Nipple Stimulation on Induction With Oxytocin and the Labor Process. *Worldviews Evid Based Nurs* 2015, 12(5):273-280.

116. Dennis CL, Dowswell T: Interventions (other than pharmacological, psychosocial or psychological) for treating antenatal depression. In., vol. 2013; 2013.

117. Dennis CL, Jackson K, Watson J: Interventions for treating painful nipples among breastfeeding women. In., vol. 2014; 2014.

118. Dennis CL, Schottle N, Hodnett E, McQueen K: An all-purpose nipple ointment versus lanolin in treating painful damaged nipples in breastfeeding women: a randomized controlled trial. *Breastfeed Med* 2012, 7(6):473-479.

119. Dennis CLE: Treatment of postpartum depression, Part 2: A critical review of nonbiological interventions. In., vol. 65; 2004.

120. Dennis CLE: Preventing postpartum depression part I: A review of biological interventions. In., vol. 49; 2004.

121. Desai V, Kozyrskyj AL, Lau S, Sanni O, Dennett L, Walter J, Ospina MB: Effectiveness of Probiotic, Prebiotic, and Synbiotic Supplementation to Improve Perinatal Mental Health in Mothers: A Systematic Review and Meta-Analysis. In., vol. 12; 2021.

122. Dettinger JC, Kinuthia J, Pintye J, Abuna F, Begnel E, Mugwanya K, Sila J, Lagat H, Baeten JM, John-Stewart G: Perinatal outcomes following maternal pre-exposure prophylaxis (PrEP) use during pregnancy: results from a large PrEP implementation program in Kenya. *J Int AIDS Soc* 2019, 22(9):e25378.

123. Deussen AR, Ashwood P, Martis R, Stewart F, Grzeskowiak LE: Relief of pain due to uterine cramping/involution after birth. In., vol. 2020; 2020.

124. Diaz V, Abalos E, Carroli G: Methods for blood loss estimation after vaginal birth. In., vol. 2018; 2018.

125. DiClemente RJ, Wingood GM, Rose E, Sales JM, Crosby RA: Evaluation of an HIV/STD sexual risk-reduction intervention for pregnant African American adolescents attending a prenatal clinic in an urban public hospital: preliminary evidence of efficacy. *J Pediatr Adolesc Gynecol* 2010, 23(1):32-38.

126. DiMarco MA, Menke EM, McNamara T: Evaluating a support group for perinatal loss. *MCN Am J Matern Child Nurs* 2001, 26(3):135-140.

127. Dineva M, Fishpool H, Rayman MP, Mendis J, Bath SC: Systematic review and meta-analysis of the effects of iodine supplementation on thyroid function and child neurodevelopment in mildly-to-moderately iodine-deficient pregnant women. In., vol. 112; 2020.

128. Dodd JM, Turnbull D, McPhee AJ, Deussen AR, Grivell RM, Yelland LN, Crowther CA, Wittert G, Owens JA, Robinson JS *et al*: Antenatal lifestyle advice for women who are overweight or obese: LIMIT randomised trial. *BMJ* 2014, 348:g1285.

129. Dodd V, Chalmers C: Comparing the use of hydrogel dressings to lanolin ointment with lactating mothers. *J Obstet Gynecol Neonatal Nurs* 2003, 32(4):486-494.

For peer review only - http://bmjopen.bmj.com/site/about/guidelines.xhtml

130. Doering JJ, Dogan S: A Postpartum Sleep and Fatigue Intervention Feasibility Pilot Study. *Behav Sleep Med* 2018, 16(2):185-201.

131. Dokmak F, Michalek IM, Boulvain M, Desseauve D: Squatting position in the second stage of labor: A systematic review and meta-analysis. In., vol. 254; 2020.

132. Dougall G, Franssen M, Tucker KL, Yu LM, Hinton L, Rivero-Arias O, Abel L, Allen J, Band RJ, Chisholm A *et al*: Blood pressure monitoring in high-risk pregnancy to improve the detection and monitoring of hypertension (the BUMP 1 and 2 trials): protocol for two linked randomised controlled trials. *BMJ Open* 2020, 10(1):e034593.

133. Downe S, Finlayson K, Melvin C, Spiby H, Ali S, Diggle P, Gyte G, Hinder S, Miller V, Slade P *et al*: Self-hypnosis for intrapartum pain management in pregnant nulliparous women: a randomised controlled trial of clinical effectiveness. *BJOG* 2015, 122(9):1226-1234.

134. Downs DS, Dinallo JM, Birch LL, Paul IM, Ulbrecht JS: Randomized Face-to-Face vs. Home Exercise Interventions in Pregnant Women with Gestational Diabetes. *Psychol Sport Exerc* 2017, 30:73-81.

135. Downs DS, Savage JS, Rivera DE, Pauley AM, Leonard KS, Hohman EE, Guo P, McNitt KM, Stetter C, Kunselman A: Adaptive, behavioral intervention impact on weight gain, physical activity, energy intake, and motivational determinants: results of a feasibility trial in pregnant women with overweight/obesity. *J Behav Med* 2021, 44(5):605-621.

136. Draffin CR, Alderdice FA, McCance DR, Maresh M, Harper R, Patterson CC, Bernatavicius G, Brennan SF, Gough A, McSorley O *et al*: Impact of an educational DVD on anxiety and glycaemic control in women diagnosed with gestational diabetes mellitus (GDM): A randomised controlled trial. *Diabetes Res Clin Pract* 2017, 126:164-171.

137. Dritsa M, Dupuis G, Lowensteyn I, Da Costa D: Effects of home-based exercise on fatigue in postpartum depressed women: who is more likely to benefit and why? *J Psychosom Res* 2009, 67(2):159-163.

138. Dugas M, Shorten A, Dube E, Wassef M, Bujold E, Chaillet N: Decision aid tools to support women's decision making in pregnancy and birth: a systematic review and meta-analysis. *Soc Sci Med* 2012, 74(12):1968-1978.

139. Duncan LG, Cohn MA, Chao MT, Cook JG, Riccobono J, Bardacke N: Benefits of preparing for childbirth with mindfulness training: a randomized controlled trial with active comparison. *BMC Pregnancy Childbirth* 2017, 17(1):140.

140. Dutra LRDV, Araújo AMPdH, Micussi MTABC: Non-pharmacological therapies for postpartum analgesia: a systematic review. *Brazilian Journal Of Pain* 2019, 2(1).

141. East CE, Dolan WJ, Forster DA: Antenatal breast milk expression by women with diabetes for improving infant outcomes. In., vol. 2014; 2014.

142. East CE, Dorward ED, Whale RE, Liu J: Local cooling for relieving pain from perineal trauma sustained during childbirth. *Cochrane Database Syst Rev* 2020, 10(10):CD006304.

143. Ebrahimian A, Rahmani Bilandi R: Comparisons of the Effects of Watching Virtual Reality Videos and Chewing Gum on the Length of Delivery Stages and Maternal Childbirth Satisfaction: A Randomized Controlled Trial. *Iran J Med Sci* 2021, 46(1):15-22.

144. Edwards MJ, Geiser T, Chafin C, Weatherby NL, Smith CM: S.M.A.R.T. mothers are resisting tobacco: prenatal smoking cessation in WIC mothers. *J Allied Health* 2009, 38(3):170-176.

145. Ehrlich SF, Hedderson MM, Brown SD, Sternfeld B, Chasan-Taber L, Feng J, Adams J, Ching J, Crites Y, Quesenberry CP *et al*: Moderate intensity sports and exercise is associated with glycaemic control in women with gestational diabetes. *Diabetes Metab* 2017, 43(5):416-423.

146. Ejike BU, Ohaeri CC, Amaechi EC: Home management practices and its impact on malaria prevalence amongst pregnant women in South-Eastern Nigeria. *Asian Pacific Journal of Tropical Disease* 2017, 7(2):68-70.

147. Ekhtiari YS, Majlessi F, Foroushani AR, Shakibazadeh E: Effect of a self-care educational program based on the health belief model on reducing low birth weight among pregnant Iranian women. *International Journal of Preventive Medicine* 2014, 5(1).

For peer review only - http://bmjopen.bmj.com/site/about/guidelines.xhtml

148. El-Rafie MM, Khafagy GM, Gamal MG: Effect of aerobic exercise during pregnancy on antenatal depression. *Int J Womens Health* 2016, 8:53-57.

149. Epidural, Position Trial Collaborative G: Upright versus lying down position in second stage of labour in nulliparous women with low dose epidural: BUMPES randomised controlled trial. *BMJ* 2017, 359:j4471.

150. Erchick DJ, Agrawal NK, Khatry SK, Katz J, LeClerq SC, Reynolds MA, Mullany LC: Adherence to and acceptability of three alcohol-free, antiseptic oral rinses: A community-based pilot randomized controlled trial among pregnant women in rural Nepal. *Community Dent Oral Epidemiol* 2020, 48(6):501-512.

151. Ershoff DH, Mullen PD, Quinn VP: A randomized trial of a serialized self-help smoking cessation program for pregnant women in an HMO. *Am J Public Health* 1989, 79(2):182-187.

152. Ershoff DH, Quinn VP, Boyd NR, Stern J, Gregory M, Wirtschafter D: The Kaiser Permanente prenatal smoking-cessation trial11The full text of this article is available via AJPM Online at http://www.elsevier.com/locate/ajpmonline. *American Journal of Preventive Medicine* 1999, 17(3):161-168.

153. Espersen T, Klebe JG: Self-monitoring of blood glucose in pregnant diabetics. A comparative study of the blood glucose level and course of pregnancy in pregnant diabetics on an out-patient regime before and after the introduction of methods for home analysis of blood glucose. *Acta Obstet Gynecol Scand* 1985, 64(1):11-14.

154. Facchinetti F, Pedrielli G, Benoni G, Joppi M, Verlato G, Dante G, Balduzzi S, Cuzzolin L: Herbal supplements in pregnancy: unexpected results from a multicentre study. *Hum Reprod* 2012, 27(11):3161-3167.

155. Fathi-Ashtiani A, Ahmadi A, Ghobari-Bonab B, Azizi MP, Saheb-Alzamani SM: Randomized Trial of Psychological Interventions to Preventing Postpartum Depression among Iranian First-time Mothers. *Int J Prev Med* 2015, 6:109.

156. Feder L, Niolon PH, Campbell J, Whitaker DJ, Brown J, Rostad W, Bacon S: An Intimate Partner Violence Prevention Intervention in a Nurse Home Visitation Program: A Randomized Clinical Trial. *J Womens Health (Larchmt)* 2018, 27(12):1482-1490.

157. Feng G, Simpson JA, Chaluluka E, Molyneux ME, Rogerson SJ: Decreasing burden of malaria in pregnancy in Malawian women and its relationship to use of intermittent preventive therapy or bed nets. *PLoS One* 2010, 5(8):e12012.

158. Fernandez L, Cardenas N, Arroyo R, Manzano S, Jimenez E, Martin V, Rodriguez JM: Prevention of Infectious Mastitis by Oral Administration of Lactobacillus salivarius PS2 During Late Pregnancy. *Clin Infect Dis* 2016, 62(5):568-573.

159. Field T, Diego M, Delgado J, Medina L: Yoga and social support reduce prenatal depression, anxiety and cortisol. *J Bodyw Mov Ther* 2013, 17(4):397-403.

160. Flannery C, Fredrix M, Olander EK, McAuliffe FM, Byrne M, Kearney PM: Effectiveness of physical activity interventions for overweight and obesity during pregnancy: A systematic review of the content of behaviour change interventions. In., vol. 16; 2019.

161. Fogarty S, McInerney C, Hay P: Pregnancy-related Pelvic Girdle Pain and Pregnancy Massage: Findings from a Subgroup Analysis of an Observational Study. *Int J Ther Massage Bodywork* 2020, 13(2):1-8.

162. Fokam EB, Ngimuh L, Anchang-Kimbi JK, Wanji S: Assessment of the usage and effectiveness of intermittent preventive treatment and insecticide-treated nets on the indicators of malaria among pregnant women attending antenatal care in the Buea Health District, Cameroon. *Malar J* 2016, 15(1):172.

163. Foudil-Bey I, Murphy MSQ, Dunn S, Keely EJ, El-Chaar D: Evaluating antenatal breastmilk expression outcomes: a scoping review. *Int Breastfeed J* 2021, 16(1):25.

164. Franciscatto LG, Silva CM, Barcellos RB, Angeli S, Silva MS, Almeida SE, Rossetti ML: Comparison of urine and self-collected vaginal samples for detecting human papillomavirus DNA in pregnant women. *Int J Gynaecol Obstet* 2014, 125(1):69-72.

165. Fritel X, de Tayrac R, Bader G, Savary D, Gueye A, Deffieux X, Fernandez H, Richet C, Guilhot J, Fauconnier A: Preventing Urinary Incontinence With Supervised Prenatal Pelvic Floor Exercises: A Randomized Controlled Trial. *Obstet Gynecol* 2015, 126(2):370-377.

For peer review only - http://bmjopen.bmj.com/site/about/guidelines.xhtml

166. Fujioka N, Kobayashi T, Turale S: Short-term behavioral changes in pregnant women after a quit-smoking program via e-learning: a descriptive study from Japan. *Nurs Health Sci* 2012, 14(3):304-311.

167. Gallo RBS, Santana LS, Marcolin AC, Duarte G, Quintana SM: Sequential application of non-pharmacological interventions reduces the severity of labour pain, delays use of pharmacological analgesia, and improves some obstetric outcomes: a randomised trial. *J Physiother* 2018, 64(1):33-40.

168. Gamble C, Ekwaru JP, ter Kuile FO: Insecticide-treated nets for preventing malaria in pregnancy. *Cochrane Database Syst Rev* 2006(2):CD003755.

169. Garmendia ML, Casanello P, Flores M, Kusanovic JP, Uauy R: The effects of a combined intervention (docosahexaenoic acid supplementation and home-based dietary counseling) on metabolic control in obese and overweight pregnant women: the MIGHT study. *Am J Obstet Gynecol* 2021, 224(5):526 e521-526 e525.

170. Garnaes KK, Morkved S, Salvesen KA, Salvesen O, Moholdt T: Exercise training during pregnancy reduces circulating insulin levels in overweight/obese women postpartum: secondary analysis of a randomised controlled trial (the ETIP trial). *BMC Pregnancy Childbirth* 2018, 18(1):18.

171. Garnweidner-Holme L, Henriksen L, Torheim LE, Lukasse M: Effect of the Pregnant+ Smartphone App on the Dietary Behavior of Women With Gestational Diabetes Mellitus: Secondary Analysis of a Randomized Controlled Trial. *JMIR Mhealth Uhealth* 2020, 8(11):e18614.

172. Gau ML, Chang CY, Tian SH, Lin KC: Effects of birth ball exercise on pain and self-efficacy during childbirth: a randomised controlled trial in Taiwan. *Midwifery* 2011, 27(6):e293-300.

173. Gedde-Dahl M, Fors EA: Impact of self-administered relaxation and guided imagery techniques during final trimester and birth. *Complement Ther Clin Pract* 2012, 18(1):60-65.

174. Geisinger ML, Geurs NC, Bain JL, Kaur M, Vassilopoulos PJ, Cliver SP, Hauth JC, Reddy MS: Oral health education and therapy reduces gingivitis during pregnancy. *J Clin Periodontol* 2014, 41(2):141-148.

175. Germeroth LJ, Benno MT, Kolko Conlon RP, Emery RL, Cheng Y, Grace J, Salk RH, Levine MD: Trial design and methodology for a non-restricted sequential multiple assignment randomized trial to evaluate combinations of perinatal interventions to optimize women's health. *Contemp Clin Trials* 2019, 79:111-121.

176. Ghaffari M, Rakhshanderou S, Safari-Moradabadi A, Torabi S: Oral and dental health care during pregnancy: Evaluating a theory-driven intervention. *Oral Dis* 2018, 24(8):1606-1614.

177. Ghahremani L, Alipoor M, Amoee S, Keshavarzi S: Health Promoting Behaviors and Self-efficacy of Physical Activity During Pregnancy: An Interventional Study. *International Journal of Women's Health and Reproduction Sciences* 2017, 5(3):181-185.

178. Ghiasvand F, Riazi H, Hajian S, Kazemi E, Firoozi A: The effect of a self-care program based on the teach back method on the postpartum quality of life. *Electron Physician* 2017, 9(4):4180-4189.

179. Ghouri F, Hollywood A, Ryan K: A systematic review of non-antibiotic measures for the prevention of urinary tract infections in pregnancy. *BMC Pregnancy Childbirth* 2018, 18(1):99.

180. Gielen AC, Windsor R, Faden RR, O'Campo P, Repke J, Davis M: Evaluation of a smoking cessation intervention for pregnant women in an urban prenatal clinic. *Health Educ Res* 1997, 12(2):247-254.

181. Gilad R, Hochner H, Savitsky B, Porat S, Hochner-Celnikier D: Castor oil for induction of labor in post-date pregnancies: A randomized controlled trial. *Women Birth* 2018, 31(1):e26-e31.

182. Gilbert L, Gross J, Lanzi S, Quansah DY, Puder J, Horsch A: How diet, physical activity and psychosocial well-being interact in women with gestational diabetes mellitus: an integrative review. *BMC Pregnancy Childbirth* 2019, 19(1):60.

183. Gilinsky A, Swanson V, Power K: Interventions delivered during antenatal care to reduce alcohol consumption during pregnancy: A systematic review. In., vol. 19; 2011.

184. Gilinsky AS, Dale H, Robinson C, Hughes AR, McInnes R, Lavallee D: Efficacy of physical activity interventions in post-natal populations: systematic review, meta-analysis and content coding of behaviour change techniques. *Health Psychol Rev* 2015, 9(2):244-263.

For peer review only - http://bmjopen.bmj.com/site/about/guidelines.xhtml

185. Gille C, Boer B, Marschal M, Urschitz MS, Heinecke V, Hund V, Speidel S, Tarnow I, Mylonas I, Franz A *et al*: Effect of probiotics on vaginal health in pregnancy. EFFPRO, a randomized controlled trial. *Am J Obstet Gynecol* 2016, 215(5):608 e601-608 e607.
186. Godfrey KM, Barton SJ, El-Heis S, Kenealy T, Nield H, Baker PN, Chong YS, Cutfield W, Chan SY, Ni PSG: Myo-Inositol, Probiotics, and Micronutrient Supplementation From Preconception for Glycemia in Pregnancy: NiPPeR International Multicenter Double-Blind Randomized Controlled Trial. *Diabetes Care* 2021, 44(5):1091-1099.
187. Goetz M, Schiele C, Muller M, Matthies LM, Deutsch TM, Spano C, Graf J, Zipfel S, Bauer A, Brucker SY *et al*: Effects of a Brief Electronic Mindfulness-Based Intervention on Relieving Prenatal Depression and Anxiety in Hospitalized High-Risk Pregnant Women: Exploratory Pilot Study. *J Med Internet Res* 2020, 22(8):e17593.
188. Goksin I, Ayaz-Alkaya S: The effect of progressive muscle relaxation on the postpartum depression risk and general comfort levels in primiparas. *Stress Health* 2020, 36(3):322-329.
189. Gomes F, King SE, Dallmann D, Golan J, da Silva ACF, Hurley KM, Bergeron G, Bourassa MW, Mehta S: Interventions to increase adherence to micronutrient supplementation during pregnancy: a systematic review. *Ann N Y Acad Sci* 2021, 1493(1):41-58.
190. Goodman JH, Guarino A, Chenausky K, Klein L, Prager J, Petersen R, Forget A, Freeman M: CALM Pregnancy: results of a pilot study of mindfulness-based cognitive therapy for perinatal anxiety. *Arch Womens Ment Health* 2014, 17(5):373-387.
191. Grant SM, Wolever TM, O'Connor DL, Nisenbaum R, Josse RG: Effect of a low glycaemic index diet on blood glucose in women with gestational hyperglycaemia. *Diabetes Res Clin Pract* 2011, 91(1):15-22.
192. Green SM, Donegan E, McCabe RE, Streiner DL, Agako A, Frey BN: Cognitive behavioral therapy for perinatal anxiety: A randomized controlled trial. *Aust N Z J Psychiatry* 2020, 54(4):423-432.
193. Grenvik JM, Rosenthal E, Saccone G, Della Corte L, Quist-Nelson J, Gerkin RD, Gimovsky AC, Kwan M, Mercier R, Berghella V: Peanut ball for decreasing length of labor: A systematic review and meta-analysis of randomized controlled trials. *Eur J Obstet Gynecol Reprod Biol* 2019, 242:159-165.
194. Griffith RJ, Alsweiler J, Moore AE, Brown S, Middleton P, Shepherd E, Crowther CA: Interventions to prevent women from developing gestational diabetes mellitus: an overview of Cochrane Reviews. In., vol. 2020; 2020.
195. Griffiths SE, Parsons J, Naughton F, Fulton EA, Tombor I, Brown KE: Are digital interventions for smoking cessation in pregnancy effective? A systematic review and meta-analysis. In., vol. 12; 2018.
196. Grzeskowiak LE, Smith B, Roy A, Dekker GA, Clifton VL: An observational study of the impact of an antenatal asthma management service on asthma control during pregnancy. *Eur J Obstet Gynecol Reprod Biol* 2016, 197:48-53.
197. Guelfi KJ, Ong MJ, Crisp NA, Fournier PA, Wallman KE, Grove JR, Doherty DA, Newnham JP: Regular Exercise to Prevent the Recurrence of Gestational Diabetes Mellitus: A Randomized Controlled Trial. In*: 2016*.
198. Gunther J, Hoffmann J, Kunath J, Spies M, Meyer D, Stecher L, Rosenfeld E, Kick L, Rauh K, Hauner H: Effects of a Lifestyle Intervention in Routine Care on Prenatal Dietary Behavior-Findings from the Cluster-Randomized GeliS Trial. *J Clin Med* 2019, 8(7).
199. Guo W, Zhang B, Wang X: Lifestyle interventions for gestational diabetes mellitus to control blood glucose: a meta-analysis of randomized studies. *International Journal of Diabetes in Developing Countries* 2017, 38(1):26-35.
200. Gupta JK, Sood A, Hofmeyr GJ, Vogel JP: Position in the second stage of labour for women without epidural anaesthesia. In., vol. 2017; 2017.
201. Gustafsson MK, Romundstad PR, Stafne SN, Helvik AS, Stunes AK, Morkved S, Salvesen KA, Thorsby PM, Mosti MP, Syversen U: The effect of an exercise program in pregnancy on vitamin D status among healthy, pregnant Norwegian women: a randomized controlled trial. *BMC Pregnancy Childbirth* 2019, 19(1):76.
202. Gutke A, Betten C, Degerskär K, Pousette S, Fagevik Olsén M: Treatments for pregnancy-related lumbopelvic pain: A systematic review of physiotherapy modalities. In., vol. 94; 2015.

For peer review only - http://bmjopen.bmj.com/site/about/guidelines.xhtml

203. Hagan R, Evans SF, Pope S: Preventing postnatal depression in mothers of very preterm infants: a randomised controlled trial. *BJOG* 2004, 111(7):641-647.

204. Haghighat M, Mirghafourvand M, Mohammad-Alizadeh-Charandabi S, Malakouti J, Erfani M: The Effect of Spiritual Counseling on Stress and Anxiety in Pregnancy: A Randomized Controlled Clinical Trial. *Iranian Red Crescent Medical Journal* 2018, In Press(In Press).

205. Haire-Joshu D, Cahill AG, Stein RI, Cade WT, Woolfolk CL, Moley K, Mathur A, Schwarz CD, Schechtman KB, Klein S: Randomized Controlled Trial of Home-Based Lifestyle Therapy on Postpartum Weight in Underserved Women with Overweight or Obesity. *Obesity (Silver Spring)* 2019, 27(4):535-541.

206. Hajek P, West R, Lee A, Foulds J, Owen L, Eiser JR, Main N: Randomized controlled trial of a midwife-delivered brief smoking cessation intervention in pregnancy. *Addiction* 2001, 96(3):485-494.

207. Halse RE, Wallman KE, Dimmock JA, Newnham JP, Guelfi KJ: Home-Based Exercise Improves Fitness and Exercise Attitude and Intention in Women with GDM. *Med Sci Sports Exerc* 2015, 47(8):1698-1704.

208. Halvorsen S, Haakstad LA, Edvardsen E, Bo K: Effect of aerobic dance on cardiorespiratory fitness in pregnant women: a randomised controlled trial. *Physiotherapy* 2013, 99(1):42-48.

209. Hannover W, Thyrian JR, Roske K, Grempler J, Rumpf HJ, John U, Hapke U: Smoking cessation and relapse prevention for postpartum women: results from a randomized controlled trial at 6, 12, 18 and 24 months. *Addict Behav* 2009, 34(1):1-8.

210. Harding KB, Peña-Rosas JP, Webster AC, Yap CMY, Payne BA, Ota E, De-Regil LM: Iodine supplementation for women during the preconception, pregnancy and postpartum period. In., vol. 2017; 2017.

211. Hardy I, Lloyd A, Morisset AS, Camirand Lemyre F, Baillargeon JP, Fraser WD: Healthy for My Baby Research Protocol- a Randomized Controlled Trial Assessing a Preconception Intervention to Improve the Lifestyle of Overweight Women and Their Partners. *Front Public Health* 2021, 9:670304.

212. Harrison CL, Lombard CB, Teede HJ: Limiting postpartum weight retention through early antenatal intervention: the HeLP-her randomised controlled trial. *Int J Behav Nutr Phys Act* 2014, 11(1):134.

213. Haruna M, Matsuzaki M, Ota E, Shiraishi M, Hanada N, Mori R: Guided imagery for treating hypertension in pregnancy. *Cochrane Database Syst Rev* 2019, 4(4):CD011337.

214. Haugland KS, Rasmussen S, Daltveit AK: Group intervention for women with pelvic girdle pain in pregnancy. A randomized controlled trial. *Acta Obstet Gynecol Scand* 2006, 85(11):1320-1326.

215. Haver J, Ansari N, Zainullah P, Kim YM, Tappis H: Misoprostol for Prevention of Postpartum Hemorrhage at Home Birth in Afghanistan: Program Expansion Experience. *J Midwifery Womens Health* 2016, 61(2):196-202.

216. Hawkins JS, Casey BM, Lo JY, Moss K, McIntire DD, Leveno KJ: Weekly compared with daily blood glucose monitoring in women with diet-treated gestational diabetes. *Obstet Gynecol* 2009, 113(6):1307-1312.

217. Heffron R, Mugo N, Hong T, Celum C, Marzinke MA, Ngure K, Asiimwe S, Katabira E, Bukusi EA, Odoyo J *et al*: Pregnancy outcomes and infant growth among babies with in-utero exposure to tenofovir-based preexposure prophylaxis for HIV prevention. *AIDS* 2018, 32(12):1707-1713.

218. Heller HM, Hoogendoorn AW, Honig A, Broekman BFP, van Straten A: The Effectiveness of a Guided Internet-Based Tool for the Treatment of Depression and Anxiety in Pregnancy (MamaKits Online): Randomized Controlled Trial. *J Med Internet Res* 2020, 22(3):e15172.

219. Henrique AJ, Gabrielloni MC, Rodney P, Barbieri M: Non-pharmacological interventions during childbirth for pain relief, anxiety, and neuroendocrine stress parameters: A randomized controlled trial. *Int J Nurs Pract* 2018, 24(3):e12642.

220. Herbec A, Brown J, Tombor I, Michie S, West R: Pilot randomized controlled trial of an internet-based smoking cessation intervention for pregnant smokers ('MumsQuit'). *Drug Alcohol Depend* 2014, 140:130-136.

221. Herring SJ, Cruice JF, Bennett GG, Rose MZ, Davey A, Foster GD: Preventing excessive gestational weight gain among African American women: A randomized clinical trial. *Obesity (Silver Spring)* 2016, 24(1):30-36.

For peer review only - http://bmjopen.bmj.com/site/about/guidelines.xhtml

222. Heslehurst N, Hayes L, Jones D, Newham J, Olajide J, McLeman L, McParlin C, de Brun C, Azevedo L: The effectiveness of smoking cessation, alcohol reduction, diet and physical activity interventions in changing behaviours during pregnancy: A systematic review of systematic reviews. *PLoS One* 2020, 15(5):e0232774.
223. Hill B, Skouteris H, Fuller-Tyszkiewicz M: Interventions designed to limit gestational weight gain: a systematic review of theory and meta-analysis of intervention components. *Obes Rev* 2013, 14(6):435-450.
224. Hirshberg A, Downes K, Srinivas S: Comparing standard office-based follow-up with text-based remote monitoring in the management of postpartum hypertension: a randomised clinical trial. *BMJ Qual Saf* 2018, 27(11):871-877.
225. Ho M, Chang YY, Chang WC, Lin HC, Wang MH, Lin WC, Chiu TH: Oral Lactobacillus rhamnosus GR-1 and Lactobacillus reuteri RC-14 to reduce Group B Streptococcus colonization in pregnant women: A randomized controlled trial. *Taiwan J Obstet Gynecol* 2016, 55(4):515-518.
226. Hofmeyr GJ, Abdel-Aleem H, Abdel-Aleem MA: Uterine massage for preventing postpartum haemorrhage. *Cochrane Database Syst Rev* 2013(7):CD006431.
227. Hofmeyr GJ, Lawrie TA, Atallah ÁN, Torloni MR: Calcium supplementation during pregnancy for preventing hypertensive disorders and related problems. In., vol. 2018; 2018.
228. Hofmeyr GJ, Manyame S, Medley N, Williams MJ: Calcium supplementation commencing before or early in pregnancy, for preventing hypertensive disorders of pregnancy. *Cochrane Database Syst Rev* 2019, 9:CD011192.
229. Holden SC, Manor B, Zhou J, Zera C, Davis RB, Yeh GY: Prenatal Yoga for Back Pain, Balance, and Maternal Wellness: A Randomized, Controlled Pilot Study. *Glob Adv Health Med* 2019, 8:2164956119870984.
230. Hollenbach D, Broker R, Herlehy S, Stuber K: Non-pharmacological interventions for sleep quality and insomnia during pregnancy: A systematic review. *J Can Chiropr Assoc* 2013, 57(3):260-270.
231. Homko CJ, Sivan E, Reece EA: The impact of self-monitoring of blood glucose on self-efficacy and pregnancy outcomes in women with diet-controlled gestational diabetes. *The Diabetes educator* 2002, 28(3).
232. Howarth AM, Swain NR: Skills-based childbirth preparation increases childbirth self-efficacy for first time mothers. *Midwifery* 2019, 70:100-105.
233. Howell EA, Balbierz A, Wang J, Parides M, Zlotnick C, Leventhal H: Reducing postpartum depressive symptoms among black and Latina mothers: a randomized controlled trial. *Obstet Gynecol* 2012, 119(5):942-949.
234. Howell EA, Bodnar-Deren S, Balbierz A, Loudon H, Mora PA, Zlotnick C, Wang J, Leventhal H: An intervention to reduce postpartum depressive symptoms: a randomized controlled trial. *Arch Womens Ment Health* 2014, 17(1):57-63.
235. Hsiao WH, Paterno MT, Iradukunda F, Hawkins M: The Preliminary Efficacy of a Sleep Self-management Intervention Using a Personalized Health Monitoring Device during Pregnancy. *Behav Sleep Med* 2021, 19(6):705-716.
236. Hu Y, Lu H, Huang J, Zang Y: Efficacy and safety of non-pharmacological interventions for labour pain management: A systematic review and Bayesian network meta-analysis. In., vol. 30; 2021.
237. Huang CM, Wu HL, Huang SH, Chien LY, Guo JL: Transtheoretical model-based passive smoking prevention programme among pregnant women and mothers of young children. *Eur J Public Health* 2013, 23(5):777-782.
238. Huang TT, Yeh CY, Tsai YC: A diet and physical activity intervention for preventing weight retention among Taiwanese childbearing women: a randomised controlled trial. *Midwifery* 2011, 27(2):257-264.
239. Hughes BL, Gans KM, Raker C, Hipolito ER, Rouse DJ: A brief prenatal intervention of behavioral change to reduce the risk of maternal cytomegalovirus: A randomized controlled trial. In*: 2017*.
240. Huhn EA, Linder T, Eppel D, Weisshaupt K, Klapp C, Schellong K, Henrich W, Yerlikaya-Schatten G, Rosicky I, Husslein P *et al*: Effectiveness of real-time continuous glucose monitoring to improve glycaemic control and pregnancy outcome in patients with gestational diabetes mellitus: a study protocol for a randomised controlled trial. *BMJ Open* 2020, 10(11):e040498.

For peer review only - http://bmjopen.bmj.com/site/about/guidelines.xhtml

241. Hui AL, Ludwig S, Gardiner P, Sevenhuysen G, Murray R, Morris M, Shen GX: Community-based Exercise and Dietary Intervention During Pregnancy:A Pilot Study. *Canadian Journal of Diabetes* 2006, 30(2):1-7.

242. Hussein N, Kai J, Qureshi N: The effects of preconception interventions on improving reproductive health and pregnancy outcomes in primary care: A systematic review. In., vol. 22; 2016.

243. Huvinen E, Koivusalo SB, Meinila J, Valkama A, Tiitinen A, Rono K, Stach-Lempinen B, Eriksson JG: Effects of a Lifestyle Intervention During Pregnancy and First Postpartum Year: Findings From the RADIEL Study. *J Clin Endocrinol Metab* 2018, 103(4):1669-1677.

244. Ibrahim HA-F, Said HII, Elgzar WTI: Effect of upright and ambulant positions versus lying down during the active first stage of labor on birth outcomes among nulliparous women: randomized controlled clinical trial. *Frontiers of Nursing* 2020, 7(3):239-248.

245. Ip WY, Tang CS, Goggins WB: An educational intervention to improve women's ability to cope with childbirth. *J Clin Nurs* 2009, 18(15):2125-2135.

246. Isbir GG, Inci F, Onal H, Yildiz PD: The effects of antenatal education on fear of childbirth, maternal self-efficacy and post-traumatic stress disorder (PTSD) symptoms following childbirth: an experimental study. *Appl Nurs Res* 2016, 32:227-232.

247. Islam S, Mohanty SK: Understanding the association between gradient of cooking fuels and low birth weight in India. *SSM Popul Health* 2021, 13:100732.

248. Jackson KT, Dennis CL: Lanolin for the treatment of nipple pain in breastfeeding women: a randomized controlled trial. *Matern Child Nutr* 2017, 13(3).

249. Jahanfar S, Jaafar SH: Effects of restricted caffeine intake by mother on fetal, neonatal and pregnancy outcomes. In., vol. 2015; 2015.

250. Jalambadani Z, Borji A, Bakaeian M: Examining the effect of mindfulness-based art therapy (MBAT) on stress and lifestyle of Iranian pregnant women. *J Obstet Gynaecol* 2020, 40(6):779-783.

251. Jallo N, Thacker LR, 2nd, Menzies V, Stojanovic P, Svikis DS: A Stress Coping App for Hospitalized Pregnant Women at Risk for Preterm Birth. *MCN Am J Matern Child Nurs* 2017, 42(5):257-262.

252. Jarde A, Lewis-Mikhael AM, Moayyedi P, Stearns JC, Collins SM, Beyene J, McDonald SD: Pregnancy outcomes in women taking probiotics or prebiotics: a systematic review and meta-analysis. *BMC Pregnancy Childbirth* 2018, 18(1):14.

253. Jasemzadeh M, Khafaie MA, Jaafarzadeh N, Araban M: Effectiveness of a theory-based mobile phone text message intervention for improving protective behaviors of pregnant women against air pollution: a randomized controlled trial. *Environ Sci Pollut Res Int* 2018, 25(7):6648-6655.

254. Jesse DE, Gaynes BN, Feldhousen EB, Newton ER, Bunch S, Hollon SD: Performance of a Culturally Tailored Cognitive-Behavioral Intervention Integrated in a Public Health Setting to Reduce Risk of Antepartum Depression: A Randomized Controlled Trial. *J Midwifery Womens Health* 2015, 60(5):578-592.

255. Johnsen M, Klingenberg C, Brand M, Revhaug A, Andreassen G: Antenatal breastmilk expression for women with diabetes in pregnancy - a feasibility study. *Int Breastfeed J* 2021, 16(1):56.

256. Jois RS, Tan JK, Silva D: Do probiotics in pregnancy reduce the risk of group B streptococcal colonisation? *J Paediatr Child Health* 2020, 56(9):1468-1472.

257. Jones DL, Rodriguez VJ, Mandell LN, Lee TK, Weiss SM, Peltzer K: Sexual risk behavior among HIV-infected women in South Africa at 12-months postpartum. *AIDS Care* 2019, 31(9):1114-1123.

258. Jones LV, Ray A, Moy FM, Buckley BS: Techniques of monitoring blood glucose during pregnancy for women with pre-existing diabetes. In., vol. 2019; 2019.

259. Jones MH, Barik S, Mangune HH, Jones P, Gregory SJ, Spring JE: Do birth plans adversely affect the outcome of labour? *British Journal of Midwifery* 1998, 6(1):38-41.

260. Jung ME, Stork MJ, Stapleton J, Bourne JE, Martin Ginis KA: A systematic review of behavioural interventions to increase maternal calcium intake. In., vol. 12; 2016.

For peer review only - http://bmjopen.bmj.com/site/about/guidelines.xhtml

261. Kabanywanyi AM, Macarthur JR, Stolk WA, Habbema JD, Mshinda H, Bloland PB, Abdulla S, Kachur SP: Malaria in pregnant women in an area with sustained high coverage of insecticide-treated bed nets. *Malar J* 2008, 7:133.

262. Kalafat E, Leslie K, Bhide A, Thilaganathan B, Khalil A: Pregnancy outcomes following home blood pressure monitoring in gestational hypertension. *Pregnancy Hypertens* 2019, 18:14-20.

263. Kalafat E, Mir I, Perry H, Thilaganathan B, Khalil A: Is home blood-pressure monitoring in hypertensive disorders of pregnancy consistent with clinic recordings? *Ultrasound Obstet Gynecol* 2018, 52(4):515-521.

264. Kantrowitz-Gordon I, McCurry SM, Landis CA, Lee R, Wi D: Online prenatal trial in mindfulness sleep management (OPTIMISM): protocol for a pilot randomized controlled trial. *Pilot Feasibility Stud* 2020, 6(1):128.

265. Kardel KR, Henriksen T, Iversen PO: No effect of energy supply during childbirth on delivery outcomes in nulliparous women: a randomised, double-blind, placebo-controlled trial. *J Obstet Gynaecol* 2010, 30(3):248-252.

266. Karimi F, Babazadeh R, Roudsari RL, Asgharipour N, Esmaily H: Comparing the Effectiveness of Sexual Counseling Based on PLISSITand BETTER Models on Sexual Selfdisclosure in Women with Sexual Problems after Childbirth: A Randomized Trial. *Iran J Nurs Midwifery Res* 2021, 26(1):68-74.

267. Katz J, Tielsch JM, Khatry SK, Shrestha L, Breysse P, Zeger SL, Kozuki N, Checkley W, LeClerq SC, Mullany LC: Impact of Improved Biomass and Liquid Petroleum Gas Stoves on Birth Outcomes in Rural Nepal: Results of 2 Randomized Trials. *Glob Health Sci Pract* 2020, 8(3):372-382.

268. Kavanagh J, Kelly AJ, Thomas J: Sexual intercourse for cervical ripening and induction of labour. *Cochrane Database Syst Rev* 2001(2):CD003093.

269. Kavanagh J, Kelly AJ, Thomas J: Breast stimulation for cervical ripening and induction of labour. In., vol. 2010; 2005.

270. Kazemi A, Ehsanpour S, Zahraei NSN, Hasanzadeh A, Beigi NMA, Malverdi Z: Impact of health belief modification on intention to make smoke free home among pregnant women. *Journal of Research in Medical Sciences* 2011, 16(6).

271. Keats EC, Akseer N, Thurairajah P, Cousens S, Bhutta ZA, Global Young Women's Nutrition Investigators G: Multiple-micronutrient supplementation in pregnant adolescents in low- and middle-income countries: a systematic review and a meta-analysis of individual participant data. *Nutr Rev* 2022, 80(2):141-156.

272. Keller C, Records K, Ainsworth B, Permana P, Coonrod DV: Interventions for weight management in postpartum women. In., vol. 37; 2008.

273. Kelly AJ, Kavanagh J, Thomas J: Castor oil, bath and/or enema for cervical priming and induction of labour. In., vol. 2013; 2013.

274. Kennelly MA, Ainscough K, Lindsay KL, O'Sullivan E, Gibney ER, McCarthy M, Segurado R, DeVito G, Maguire O, Smith T *et al*: Pregnancy exercise and nutrition with smartphone application support a randomized controlled trial. In*: 2018*.

275. Khatun F, Lee TW, Lee HJ, Park J, Song JE, Kim S: Does a nurse-led postpartum self-care program for first-time mothers in Bangladesh improve postpartum fatigue, depressive mood, and maternal functioning?: a non-synchronized quasi-experimental study. *Korean Journal of Women Health Nursing* 2021, 27(3):196-208.

276. Kim HK, Niederdeppe J, Graham M, Olson C, Gay G: Effects of Online Self-Regulation Activities on Physical Activity Among Pregnant and Early Postpartum Women. *J Health Commun* 2015, 20(10):1115-1124.

277. Kimber L, McNabb M, Mc Court C, Haines A, Brocklehurst P: Massage or music for pain relief in labour: a pilot randomised placebo controlled trial. *Eur J Pain* 2008, 12(8):961-969.

278. King SE, Yeh PT, Rhee DK, Tuncalp O, Rogers LM, Narasimhan M: Self-management of iron and folic acid supplementation during pre-pregnancy, pregnancy and postnatal periods: a systematic review. *BMJ Glob Health* 2021, 6(5).

For peer review only - http://bmjopen.bmj.com/site/about/guidelines.xhtml

279. Kinser P, Jallo N, Huberty J, Jones E, Thacker L, Moyer S, Laird B, Rider A, Lanni S, Drozd F *et al*: Study protocol for a multisite randomized controlled trial of an internet and mobile-based intervention for preventing and reducing perinatal depressive symptoms. *Res Nurs Health* 2021, 44(1):13-23.

280. Kinser PA, Thacker LR, Rider A, Moyer S, Amstadter AB, Mazzeo SE, Bodnar-Deren S, Starkweather A: Feasibility, Acceptability, and Preliminary Effects of "Mindful Moms": A Mindful Physical Activity Intervention for Pregnant Women with Depression. *Nurs Res* 2021, 70(2):95-105.

281. Kirca AS, Kanza Gul D: Effects of self-acupressure on pregnancy-related constipation: A single-blind randomized controlled study. *Explore (NY)* 2021, 17(5):463-468.

282. Kirihara N, Kamitomo M, Tabira T, Hashimoto T, Taniguchi H, Maeda T: Effect of probiotics on perinatal outcome in patients at high risk of preterm birth. *J Obstet Gynaecol Res* 2018, 44(2):241-247.

283. Kitt JA, Fox RL, Cairns AE, Mollison J, Burchert HH, Kenworthy Y, McCourt A, Suriano K, Lewandowski AJ, Mackillop L *et al*: Short-Term Postpartum Blood Pressure Self-Management and Long-Term Blood Pressure Control: A Randomized Controlled Trial. *Hypertension* 2021, 78(2):469-479.

284. Kobayashi M, Ogawa K, Morisaki N, Tani Y, Horikawa R, Fujiwara T: Dietary n-3 Polyunsaturated Fatty Acids in Late Pregnancy and Postpartum Depressive Symptom among Japanese Women. *Front Psychiatry* 2017, 8(NOV):241.

285. Koivusalo SB, Rono K, Klemetti MM, Roine RP, Lindstrom J, Erkkola M, Kaaja RJ, Poyhonen-Alho M, Tiitinen A, Huvinen E *et al*: Gestational Diabetes Mellitus Can Be Prevented by Lifestyle Intervention: The Finnish Gestational Diabetes Prevention Study (RADIEL): A Randomized Controlled Trial. *Diabetes Care* 2016, 39(1):24-30.

286. Kolivand M, Rahimi MA, Keramat A, Shariati M, Emamian MH: Effect of a new self-care guide package on maternal and neonatal outcomes in gestational diabetes: A randomized control trial. *J Diabetes* 2019, 11(2):139-147.

287. Kolivand M, Rahimi MA, Shariati M, Keramat A, Emamian MH: The Effect of Self-care Educational/Training Interventions on the Outcomes of Gestational Diabetes: A Review Article. *Iran J Public Health* 2018, 47(12):1805-1815.

288. Korn L, Koren G, Yaakov A, Madar G, Blau A: Evaluating the Effectiveness of Childbirth Preparation Courses on Women's Self-Efficacy among Ultra-Orthodox Jewish Religious Women in Israel. *Healthcare (Basel)* 2021, 9(7).

289. Korte JE, Kisa R, Vrana-Diaz CJ, Malek AM, Buregyeya E, Matovu JKB, Kagaayi J, Musoke W, Chemusto H, Mukama SC *et al*: HIV Oral Self-Testing for Male Partners of Women Attending Antenatal Care in Central Uganda: Uptake of Testing and Linkage to Care in a Randomized Trial. *J Acquir Immune Defic Syndr* 2020, 84(3):271-279.

290. Koshida S, Tokoro S, Katsura D, Tsuji S, Murakami T, Takahashi K: Fetal movement counting is associated with the reduction of delayed maternal reaction after perceiving decreased fetal movements: a prospective study. *Sci Rep* 2021, 11(1):10818.

291. Koyuncu SB, Bulbul M: The impact of yoga on fear of childbirth and childbirth self-efficacy among third trimester pregnants. *Complement Ther Clin Pract* 2021, 44:101438.

292. Krauss-Silva L, Moreira ME, Alves MB, Braga A, Camacho KG, Batista MR, Almada-Horta A, Rebello MR, Guerra F: A randomised controlled trial of probiotics for the prevention of spontaneous preterm delivery associated with bacterial vaginosis: preliminary results. *Trials* 2011, 12:239.

293. Kumar R, Farzeen M, Hafeez A, Achakzai BK, Vankwani M, Lal M, Iqbal R, Somrongthong R: Effectiveness of a health education intervention on the use of long-lasting insecticidal nets for the prevention of malaria in pregnant women of Pakistan: a quasi-experimental study. *Malar J* 2020, 19(1):232.

294. Kuppermann M, Kaimal AJ, Blat C, Gonzalez J, Thiet MP, Bermingham Y, Altshuler AL, Bryant AS, Bacchetti P, Grobman WA: Effect of a Patient-Centered Decision Support Tool on Rates of Trial of Labor After Previous Cesarean Delivery: The PROCEED Randomized Clinical Trial. *JAMA* 2020, 323(21):2151-2159.

For peer review only - http://bmjopen.bmj.com/site/about/guidelines.xhtml

295. Kwena Z, Kimbo L, Darbes LA, Hatcher AM, Helova A, Owino G, Thirumurthy H, Bukusi EA, Braun T, Kilgore M *et al*: Testing strategies for couple engagement in prevention of mother-to-child transmission of HIV and family health in Kenya: study protocol for a randomized controlled trial. *Trials* 2021, 22(1):19.
296. Labrecque M, Eason E, Marcoux S: Randomized trial of perineal massage during pregnancy: perineal symptoms three months after delivery. *Am J Obstet Gynecol* 2000, 182(1 Pt 1):76-80.
297. Labrecque M, Nouwen A, Bergeron M, Rancourt JF: A randomized controlled trial of nonpharmacologic approaches for relief of low back pain during labor. *J Fam Pract* 1999, 48(4):259-263.
298. Landon MB, Spong CY, Thom E, Carpenter MW, Ramin SM, Casey B, Wapner RJ, Varner MW, Rouse DJ, Thorp JM, Jr. *et al*: A multicenter, randomized trial of treatment for mild gestational diabetes. *N Engl J Med* 2009, 361(14):1339-1348.
299. Lanou H, Huybregts L, Roberfroid D, Nikiema L, Kouanda S, Van Camp J, Kolsteren P: Prenatal nutrient supplementation and postnatal growth in a developing nation: an RCT. *Pediatrics* 2014, 133(4):e1001-1008.
300. Lanssens D, Thijs IM, Gyselaers W, consortium PI: Design of the Pregnancy REmote MOnitoring II study (PREMOM II): a multicenter, randomized controlled trial of remote monitoring for gestational hypertensive disorders. *BMC Pregnancy Childbirth* 2020, 20(1):626.
301. Lauzon L, Hodnett ED, Kellie FJ: Antenatal education for self-diagnosis of the onset of active labour at term. In., vol. 2017; 1998.
302. Lawrence T, Aveyard P, Cheng KK, Griffin C, Johnson C, Croghan E: Does stage-based smoking cessation advice in pregnancy result in long-term quitters? 18-month postpartum follow-up of a randomized controlled trial. *Addiction* 2005, 100(1):107-116.
303. Lee JT, Tsai JL: Transtheoretical model-based postpartum sexual health education program improves women's sexual behaviors and sexual health. *J Sex Med* 2012, 9(4):986-996.
304. Lee M, Miller SM, Wen KY, Hui SK, Roussi P, Hernandez E: Cognitive-behavioral intervention to promote smoking cessation for pregnant and postpartum inner city women. *J Behav Med* 2015, 38(6):932-943.
305. Lertbunnaphong T, Lapthanapat N, Leetheeragul J, Hakularb P, Ownon A: Postpartum blood loss: visual estimation versus objective quantification with a novel birthing drape. *Singapore Med J* 2016, 57(6):325-328.
306. Leung SS, Lam TH: Group antenatal intervention to reduce perinatal stress and depressive symptoms related to intergenerational conflicts: a randomized controlled trial. *Int J Nurs Stud* 2012, 49(11):1391-1402.
307. Levitt C, Shaw E, Wong S, Kaczorowski J: Systematic review of the literature on postpartum care: Effectiveness of interventions for smoking relapse prevention, cessation, and reduction in postpartum women. In., vol. 34; 2007.
308. Lim K, Chan SY, Lim SL, Tai BC, Tsai C, Wong SR, Ang SM, Yew TW, Tai ES, Yong EL: A Smartphone App to Restore Optimal Weight (SPAROW) in Women With Recent Gestational Diabetes Mellitus: Randomized Controlled Trial. *JMIR Mhealth Uhealth* 2021, 9(3):e22147.
309. Lim S, Hill B, Pirotta S, O'Reilly S, Moran L: What Are the Most Effective Behavioural Strategies in Changing Postpartum Women's Physical Activity and Healthy Eating Behaviours? A Systematic Review and Meta-Analysis. *J Clin Med* 2020, 9(1).
310. Lim S, O'Reilly S, Behrens H, Skinner T, Ellis I, Dunbar JA: Effective strategies for weight loss in post-partum women: A systematic review and meta-analysis. In., vol. 16; 2015.
311. Lin YC, Gau ML, Kao GH, Lee HC: Efficacy of an Ergonomic Ankle Support Aid for Squatting Position in Improving Pushing Skills and Birth Outcomes During the Second Stage of Labor: A Randomized Controlled Trial. *J Nurs Res* 2018, 26(6):376-384.
312. Linden K, Berg M, Adolfsson A, Sparud-Lundin C: Person-centred, web-based support in pregnancy and early motherhood for women with Type 1 diabetes mellitus: a randomized controlled trial. *Diabet Med* 2018, 35(2):232-241.
313. Liston RM, Bloom K, Zimmer P: The psychological effects of counting fetal movements. *Birth* 1994, 21(3):135-140.

For peer review only - http://bmjopen.bmj.com/site/about/guidelines.xhtml

314. Liu J, Wilcox S, Wingard E, Turner-McGrievy G, Hutto B, Burgis J: A Behavioral Lifestyle Intervention to Limit Gestational Weight Gain in Pregnant Women with Overweight and Obesity. *Obesity (Silver Spring)* 2021, 29(4):672-680.

315. Liu N, Wang J, Chen Dd, Sun Wj, Li P, Zhang W: Effects of exercise on pregnancy and postpartum fatigue: A systematic review and meta-analysis. In., vol. 253; 2020.

316. Liu P, Wen W, Yu KF, Gao X, Lo ECM, Wong MCM: Effectiveness of a family-centered behavioral and educational counselling approach to improve periodontal health of pregnant women: a randomized controlled trial. *BMC Oral Health* 2020, 20(1):284.

317. Liu YH, Lee CS, Yu CH, Chen CH: Effects of music listening on stress, anxiety, and sleep quality for sleep-disturbed pregnant women. *Women Health* 2016, 56(3):296-311.

318. Llorente AM, Jensen CL, Voigt RG, Fraley JK, Berretta MC, Heird WC: Effect of maternal docosahexaenoic acid supplementation on postpartum depression and information processing. *Am J Obstet Gynecol* 2003, 188(5):1348-1353.

319. Logan S, Browne J, McKenzie H, Templeton A, Bhattacharya S: Evaluation of endocervical, first-void urine and self-administered vulval swabs for the detection of Chlamydia trachomatis in a miscarriage population. *BJOG* 2005, 112(1):103-106.

320. Loughnan SA, Sie A, Hobbs MJ, Joubert AE, Smith J, Haskelberg H, Mahoney AEJ, Kladnitski N, Holt CJ, Milgrom J *et al*: A randomized controlled trial of 'MUMentum Pregnancy': Internet-delivered cognitive behavioral therapy program for antenatal anxiety and depression. *J Affect Disord* 2019, 243:381-390.

321. Loukopoulou AN, Vardavas CI, Farmakides G, Rosolymos C, Chrelias C, Tzatzarakis M, Tsatsakis A, Myridakis A, Lyberi M, Behrakis PK: Counselling for smoking cessation during pregnancy reduces tobacco-specific nitrosamine (NNAL) concentrations: A randomized controlled trial. *Eur J Midwifery* 2018, 2(November):14.

322. Lowndes TA, Egan SJ, McEvoy PM: Efficacy of brief guided self-help cognitive behavioral treatment for perfectionism in reducing perinatal depression and anxiety: a randomized controlled trial. *Cogn Behav Ther* 2019, 48(2):106-120.

323. Lucas R, Zhang Y, Walsh SJ, Evans H, Young E, Starkweather A: Efficacy of a Breastfeeding Pain Self-Management Intervention: A Pilot Randomized Controlled Trial. *Nurs Res* 2019, 68(2):E1-E10.

324. Lucena L, Frange C, Pinto ACA, Andersen ML, Tufik S, Hachul H: Mindfulness interventions during pregnancy: A narrative review. In., vol. 18; 2020.

325. Maddady SM, Charandabi SM-A, Shafaei FS, Mirghafourvand M: Comparing the Effects of Hot Shower and Intravenous Injection of Hyoscine on the Pain Intensity and Duration of Active Phase of Labour in Nulliparous Women. *Journal of Clinical and Diagnostic Research* 2018, 12(8).

326. Madden K, Middleton P, Cyna AM, Matthewson M, Jones L: Hypnosis for pain management during labour and childbirth. In., vol. 2016; 2016.

327. Magwood O, Kpade V, Thavorn K, Oliver S, Mayhew AD, Pottie K: Effectiveness of home-based records on maternal, newborn and child health outcomes: A systematic review and meta-analysis. *PLoS One* 2019, 14(1):e0209278.

328. Mahnaz E, Nasim B, Sonia O: Effect of a structured educational package on women's sexual function during pregnancy. *Int J Gynaecol Obstet* 2020, 148(2):225-230.

329. Malekuti J, Mirghafourvand M, Samadi K, Abbasalizadeh F, Khodaei L: Comparison of the effect of Myrtus communis herbal and anti-hemorrhoid ointments on the hemorrhoid symptoms and quality of life in postpartum women with grade I and II internal hemorrhoid: A triple-blinded randomized controlled clinical trial. *J Complement Integr Med* 2019, 16(4).

330. Maman S, Moodley D, McNaughton-Reyes HL, Groves AK, Kagee A, Moodley P: Efficacy of enhanced HIV counseling for risk reduction during pregnancy and in the postpartum period: a randomized controlled trial. *PLoS One* 2014, 9(5):e97092.

For peer review only - http://bmjopen.bmj.com/site/about/guidelines.xhtml

331. Mangesi L, Hofmeyr GJ, Smith V, Smyth RMD: Fetal movement counting for assessment of fetal wellbeing. In., vol. 2015; 2015.
332. Mao HJ, Li HJ, Chiu H, Chan WC, Chen SL: Effectiveness of antenatal emotional self-management training program in prevention of postnatal depression in Chinese women. *Perspect Psychiatr Care* 2012, 48(4):218-224.
333. Marc I, Toureche N, Ernst E, Hodnett ED, Blanchet C, Dodin S, Njoya MM: Mind-body interventions during pregnancy for preventing or treating women's anxiety. In., vol. 2011; 2011.
334. Marchant T, Schellenberg JA, Edgar T, Nathan R, Abdulla S, Mukasa O, Mponda H, Lengeler C: Socially marketed insecticide-treated nets improve malaria and anaemia in pregnancy in southern Tanzania. *Trop Med Int Health* 2002, 7(2):149-158.
335. Martin J, MacDonald-Wicks L, Hure A, Smith R, Collins CE: Reducing postpartum weight retention and improving breastfeeding outcomes in overweight women: a pilot randomised controlled trial. *Nutrients* 2015, 7(3):1464-1479.
336. Martis R, Crowther CA, Shepherd E, Alsweiler J, Downie MR, Brown J: Treatments for women with gestational diabetes mellitus: An overview of Cochrane systematic reviews. In., vol. 2018; 2018.
337. Marufu TC, Ahankari A, Coleman T, Lewis S: Maternal smoking and the risk of still birth: systematic review and meta-analysis. *BMC Public Health* 2015, 15(1):239.
338. Mary M, Jafarey S, Dabash R, Kamal I, Rabbani A, Abbas D, Durocher J, Tan YL, Winikoff B: The Safety and Feasibility of a Family First Aid Approach for the Management of Postpartum Hemorrhage in Home Births: A Pre-post Intervention Study in Rural Pakistan. *Matern Child Health J* 2021, 25(1):118-126.
339. Masoumi SZ, Kazemi F, Oshvandi K, Jalali M, Esmaeili-Vardanjani A, Rafiei H: Effect of Training Preparation for Childbirth on Fear of Normal Vaginal Delivery and Choosing the Type of Delivery Among Pregnant Women in Hamadan, Iran: A Randomized Controlled Trial. *Journal of family & reproductive health* 2016, 10(3).
340. Masters SH, Agot K, Obonyo B, Napierala Mavedzenge S, Maman S, Thirumurthy H: Promoting Partner Testing and Couples Testing through Secondary Distribution of HIV Self-Tests: A Randomized Clinical Trial. *PLoS Med* 2016, 13(11):e1002166.
341. Masulli M, Vitacolonna E, Fraticelli F, Della Pepa G, Mannucci E, Monami M: Effects of probiotic supplementation during pregnancy on metabolic outcomes: A systematic review and meta-analysis of randomized controlled trials. In., vol. 162; 2020.
342. Masuzawa Y, Kataoka Y, Nakamura S, Yaju Y: Cooling the lower abdomen to reduce postpartum blood loss: A randomized controlled trial. *PLoS One* 2017, 12(10):e0186365.
343. Matei A, Saccone G, Vogel JP, Armson AB: Primary and secondary prevention of preterm birth: a review of systematic reviews and ongoing randomized controlled trials. In., vol. 236; 2019.
344. Matenchuk B, Khurana R, Cai C, Boule NG, Slater L, Davenport MH: Prenatal bed rest in developed and developing regions: a systematic review and meta-analysis. *CMAJ Open* 2019, 7(3):E435-E445.
345. Matthews LT, Jaggernath M, Kriel Y, Smith PM, O'Neil K, Haberer JE, Hendrix C, Baeten JM, Ware NC, Wirth K *et al*: Protocol for a longitudinal study to evaluate the use of tenofovir-based PrEP for safer conception and pregnancy among women in South Africa. *BMJ Open* 2019, 9(7):e027227.
346. Matvienko-Sikar K, Lee L, Murphy G, Murphy L: The effects of mindfulness interventions on prenatal well-being: A systematic review. *Psychol Health* 2016, 31(12):1415-1434.
347. McBride CM, Baucom DH, Peterson BL, Pollak KI, Palmer C, Westman E, Lyna P: Prenatal and postpartum smoking abstinence a partner-assisted approach. *Am J Prev Med* 2004, 27(3):232-238.
348. McBride CM, Curry SJ, Lando HA, Pirie PL, Grothaus LC, Nelson JC: Prevention of relapse in women who quit smoking during pregnancy. *Am J Public Health* 1999, 89(5):706-711.
349. McCarthy EA, Walker SP, Ugoni A, Lappas M, Leong O, Shub A: Self-weighing and simple dietary advice for overweight and obese pregnant women to reduce obstetric complications without impact on quality of life: A randomised controlled trial. In., vol. 123; 2016.

For peer review only - http://bmjopen.bmj.com/site/about/guidelines.xhtml

350. McCloskey RJ, Reno R: Complementary health approaches for postpartum depression: A systematic review. In., vol. 17; 2019.

351. McGrath A, Sharpe L, Lah S, Parratt K: Evaluation of a Decision Aid for Women with Epilepsy Who Are Considering Pregnancy: A Randomized Controlled Trial. *Med Decis Making* 2017, 37(5):589-599.

352. McLeod D, Pullon S, Benn C, Cookson T, Dowell A, Viccars A, White S, Green R, Crooke M: Can support and education for smoking cessation and reduction be provided effectively by midwives within primary maternity care? *Midwifery* 2004, 20(1):37-50.

353. Megregian M, Emeis C, Nieuwenhuijze M: The Impact of Shared Decision-Making in Perinatal Care: A Scoping Review. *J Midwifery Womens Health* 2020, 65(6):777-788.

354. Middleton P, Gomersall JC, Gould JF, Shepherd E, Olsen SF, Makrides M: Omega-3 fatty acid addition during pregnancy. In., vol. 2018; 2018.

355. Midhet F, Becker S: Impact of community-based interventions on maternal and neonatal health indicators: Results from a community randomized trial in rural Balochistan, Pakistan. *Reprod Health* 2010, 7(1):30.

356. Milgrom J, Holt C, Holt CJ, Ross J, Ericksen J, Gemmill AW: Feasibility study and pilot randomised trial of an antenatal depression treatment with infant follow-up. *Arch Womens Ment Health* 2015, 18(5):717-730.

357. Ming WK, Ding W, Zhang CJP, Zhong L, Long Y, Li Z, Sun C, Wu Y, Chen H, Chen H *et al*: The effect of exercise during pregnancy on gestational diabetes mellitus in normal-weight women: a systematic review and meta-analysis. *BMC Pregnancy Childbirth* 2018, 18(1):440.

358. Minschart C, Maes T, De Block C, Van Pottelbergh I, Myngheer N, Abrams P, Vinck W, Leuridan L, Mathieu C, Billen J *et al*: Mobile-Based Lifestyle Intervention in Women with Glucose Intolerance after Gestational Diabetes Mellitus (MELINDA), A Multicenter Randomized Controlled Trial: Methodology and Design. *J Clin Med* 2020, 9(8).

359. Miquelutti MA, Cecatti JG, Makuch MY: Upright position during the first stage of labor: a randomised controlled trial. *Acta Obstet Gynecol Scand* 2007, 86(5):553-558.

360. Miquelutti MA, Cecatti JG, Makuch MY: Evaluation of a birth preparation program on lumbopelvic pain, urinary incontinence, anxiety and exercise: a randomized controlled trial. *BMC Pregnancy Childbirth* 2013, 13:154.

361. Miquelutti MA, Silveira C, Cecatti JG: Kinesiologic tape for labor pain control: Randomized controlled trial. *Physiother Theory Pract* 2019, 35(7):614-621.

362. Mir AM, Wajid A, Gull S: Helping rural women in Pakistan to prevent postpartum hemorrhage: a quasi experimental study. *BMC Pregnancy Childbirth* 2012, 12:120.

363. Mobarakabadi SS, Shahbazzadegan S, Ozgoli G: The effect of P6 acupressure on nausea and vomiting of pregnancy: A randomized, single-blind, placebo-controlled trial. *Advances in Integrative Medicine* 2020, 7(2):67-72.

364. Mofenson LM, Baggaley RC, Mameletzis I: Tenofovir disoproxil fumarate safety for women and their infants during pregnancy and breastfeeding. *AIDS* 2017, 31(2):213-232.

365. Mohammadi F, Malakooti J, Babapoor J, Mohammad-Alizadeh-Charandabi S: The effect of a home-based exercise intervention on postnatal depression and fatigue: A randomized controlled trial. *Int J Nurs Pract* 2015, 21(5):478-485.

366. Monim Alzurfi N, Shadhan Al-Ogaili S: Effectiveness of Breathing as a Non-Pharmacological Method to Reduce Pain Severity among Woman During Labor. *Medico Legal Update* 2021, 21(1):674-677.

367. Moore L, Campbell R, Whelan A, Mills N, Lupton P, Misselbrook E, Frohlich J: Self help smoking cessation in pregnancy: cluster randomised controlled trial. *BMJ* 2002, 325(7377):1383.

368. Moradi M, Niazi A, Heydarian Miri H, Lopez V: The effect of evening primrose oil on labor induction and cervical ripening: A systematic review and meta-analysis. In., vol. 35; 2021.

369. Morison PN, Bacardi-Gascon M, Lopez-Corrales M, Jimenez-Cruz A: Combined dietary-exercise intervention for gestational weight gain and birthweight: a meta-analysis. *Asia Pac J Clin Nutr* 2018, 27(4):860-868.

For peer review only - http://bmjopen.bmj.com/site/about/guidelines.xhtml

370. Motahari-Tabari NS, Nasiri-Amiri F, Faramarzi M, Shirvani MA, Bakhtiari A, Omidvar S: The Effectiveness of Information-Motivation-Behavioral Skills Model on Self-Care Practices in Early Pregnancy to Prevent Gestational Diabetes Mellitus in Iranian Overweight and Obese Women: A Randomized Controlled Trial. *Int Q Community Health Educ* 2021:272684X211020300.

371. Motlagh AE, Babazadeh R, Akhlaghi F, Esmaily H: Effect of an educational intervention program based on bandura's self-efficacy theory on self-care, self-efficacy, and blood sugar levels in mothers with pre-diabetes during pregnancy. In., vol. 9; 2019.

372. Munkhondya BMJ, Munkhondya TE, Chirwa E, Wang H: Efficacy of companion-integrated childbirth preparation for childbirth fear, self-efficacy, and maternal support in primigravid women in Malawi. *BMC Pregnancy Childbirth* 2020, 20(1):48.

373. Munro S, Stacey D, Lewis KB, Bansback N: Choosing treatment and screening options congruent with values: Do decision aids help? Sub-analysis of a systematic review. In., vol. 99; 2016.

374. Murphy HR, Rayman G, Lewis K, Kelly S, Johal B, Duffield K, Fowler D, Campbell PJ, Temple RC: Effectiveness of continuous glucose monitoring in pregnant women with diabetes: randomised clinical trial. *BMJ* 2008, 337(7675):a1680.

375. N.Y VDW, C H, W VD: Preventing prenatal alcohol use via health counseling by midwives and Internet-based computer tailored feedback: A randomized controlled trial. In., vol. 19; 2012.

376. Napolitano MA, Harrington CB, Patchen L, Ellis LP, Ma T, Chang K, Gaminian A, Bailey CP, Evans WD: Feasibility of a Digital Intervention to Promote Healthy Weight Management among Postpartum African American/Black Women. *Int J Environ Res Public Health* 2021, 18(4).

377. Nassar N, Roberts CL, Raynes-Greenow CH, Barratt A, Peat B, Decision Aid for Breech Presentation Trial C: Evaluation of a decision aid for women with breech presentation at term: a randomised controlled trial [ISRCTN14570598]. *BJOG* 2007, 114(3):325-333.

378. Naughton F, Cooper S, Foster K, Emery J, Leonardi-Bee J, Sutton S, Jones M, Ussher M, Whitemore R, Leighton M *et al*: Large multi-centre pilot randomized controlled trial testing a low-cost, tailored, self-help smoking cessation text message intervention for pregnant smokers (MiQuit). *Addiction* 2017, 112(7):1238-1249.

379. Naughton F, Prevost AT, Gilbert H, Sutton S: Randomized controlled trial evaluation of a tailored leaflet and SMS text message self-help intervention for pregnant smokers (MiQuit). *Nicotine Tob Res* 2012, 14(5):569-577.

380. Naughton F, Prevost AT, Sutton S: Self-help smoking cessation interventions in pregnancy: A systematic review and meta-analysis. In., vol. 103; 2008.

381. Nazari M, Basiri F, Kaveh MH, Ghaem H: Investigating the Effect of Fordyce Happiness Cognitive-Behavioral Training on Perceived Stress and Happiness in Pregnant Females: Applying the Self-Efficacy Theory. *Iranian Red Crescent Medical Journal* 2018, In Press(In Press).

382. Ndyomugyenyi R, Clarke SE, Hutchison CL, Hansen KS, Magnussen P: Efficacy of malaria prevention during pregnancy in an area of low and unstable transmission: an individually-randomised placebo-controlled trial using intermittent preventive treatment and insecticide-treated nets in the Kabale Highlands, southwestern Uganda. *Trans R Soc Trop Med Hyg* 2011, 105(11):607-616.

383. Nelson DB, Bellamy S, Gray TS, Nachamkin I: Self-collected versus provider-collected vaginal swabs for the diagnosis of bacterial vaginosis: an assessment of validity and reliability. *J Clin Epidemiol* 2003, 56(9):862-866.

384. Neri I, Dante G, Pignatti L, Salvioli C, Facchinetti F: Castor oil for induction of labour: a retrospective study. *J Matern Fetal Neonatal Med* 2018, 31(16):2105-2108.

385. Ngichabe SK, Gatinu BW, Nyangore MA, Karuga R, Wanyonyi SZ, Kiarie JN: Reminder Systems for Self Uterine Massage in the Prevention of Postpartum Blood Loss. *East Afr Med J* 2012, 89(4):128-133.

386. Nicholson WK, Beckham AJ, Hatley K, Diamond M, Johnson LS, Green SL, Tate D: The Gestational Diabetes Management System (GooDMomS): development, feasibility and lessons learned from a patient-informed, web-based pregnancy and postpartum lifestyle intervention. *BMC Pregnancy Childbirth* 2016, 16(1):277.

For peer review only - http://bmjopen.bmj.com/site/about/guidelines.xhtml

387. Nielsen JN, Gittelsohn J, Anliker J, O'Brien K: Interventions to improve diet and weight gain among pregnant adolescents and recommendations for future research. *J Am Diet Assoc* 2006, 106(11):1825-1840.

388. Nieminen K, Andersson G, Wijma B, Ryding EL, Wijma K: Treatment of nulliparous women with severe fear of childbirth via the Internet: a feasibility study. *J Psychosom Obstet Gynaecol* 2016, 37(2):37-43.

389. Nilsson C, Lundgren I, Smith V, Vehvilainen-Julkunen K, Nicoletti J, Devane D, Bernloehr A, van Limbeek E, Lalor J, Begley C: Women-centred interventions to increase vaginal birth after caesarean section (VBAC): A systematic review. *Midwifery* 2015, 31(7):657-663.

390. Njagi JK, Magnussen P, Estambale B, Ouma J, Mugo B: Prevention of anaemia in pregnancy using insecticide-treated bednets and sulfadoxine-pyrimethamine in a highly malarious area of Kenya: a randomized controlled trial. *Transactions of the Royal Society of Tropical Medicine and Hygiene* 2003, 97(3):277-282.

391. Nwebube C, Glover V, Stewart L: Prenatal listening to songs composed for pregnancy and symptoms of anxiety and depression: a pilot study. *BMC Complement Altern Med* 2017, 17(1):256.

392. O'Kelly SM, Moore ZE: Antenatal maternal education for improving postnatal perineal healing for women who have birthed in a hospital setting. In., vol. 2017; 2017.

393. Oakeshott P, Hay P, Hay S, Steinke F, Rink E, Thomas B, Oakeley P, Kerry S: Detection of Chlamydia trachomatis infection in early pregnancy using self-administered vaginal swabs and first pass urines: A cross-sectional community-based survey. *British Journal of General Practice* 2002, 52(483).

394. Offianan AT, Penali LK, Coulibaly M, Tiacoh N, Ako A, Adji E, Coulibaly B, Koffi D, Sarr D, Jambou R *et al*: Comparative efficacy of uncontrolled and controlled intermittent preventive treatment during pregnancy (IPTp) with combined use of LLTNs in high resistance area to sulfadoxine-pyrimethamine in Cote d'Ivoire. *Infect Drug Resist* 2012, 5(1):53-63.

395. Oken E, Ning Y, Rifas-Shiman SL, Radesky JS, Rich-Edwards JW, Gillman MW: Associations of physical activity and inactivity before and during pregnancy with glucose tolerance. *Obstet Gynecol* 2006, 108(5):1200-1207.

396. Okesene-Gafa KAM, Li M, McKinlay CJD, Taylor RS, Rush EC, Wall CR, Wilson J, Murphy R, Taylor R, Thompson JMD *et al*: Effect of antenatal dietary interventions in maternal obesity on pregnancy weight-gain and birthweight: Healthy Mums and Babies (HUMBA) randomized trial. *Am J Obstet Gynecol* 2019, 221(2):152 e151-152 e113.

397. Oladapo OT, Blum J, Abalos E, Okusanya BO: Advance misoprostol distribution to pregnant women for preventing and treating postpartum haemorrhage. In., vol. 2020; 2020.

398. Oliveira FS, Vieira F, Guimaraes JV, Aredes ND, Campbell SH: Lanolin and prenatal health education for prevention of nipple pain and trauma: Randomized clinical trial. *Enferm Clin (Engl Ed)* 2021, 31(2):82-90.

399. Olson CM, Groth SW, Graham ML, Reschke JE, Strawderman MS, Fernandez ID: The effectiveness of an online intervention in preventing excessive gestational weight gain: the e-moms roc randomized controlled trial. *BMC Pregnancy Childbirth* 2018, 18(1):148.

400. Olson CM, Strawderman MS, Graham ML: Use of an Online Diet Goal-Setting Tool: Relationships With Gestational Weight Gain. *J Nutr Educ Behav* 2019, 51(4):391-399.

401. Ong MJ, Guelfi KJ, Hunter T, Wallman KE, Fournier PA, Newnham JP: Supervised home-based exercise may attenuate the decline of glucose tolerance in obese pregnant women. *Diabetes Metab* 2009, 35(5):418-421.

402. Ononge S, Campbell OM, Kaharuza F, Lewis JJ, Fielding K, Mirembe F: Effectiveness and safety of misoprostol distributed to antenatal women to prevent postpartum haemorrhage after child-births: a stepped-wedge cluster-randomized trial. *BMC Pregnancy Childbirth* 2015, 15:315.

403. Oostdam N, Van Poppel MNM, Wouters MGAJ, Van Mechelen W: Interventions for preventing gestational diabetes mellitus: A systematic review and meta-analysis. In., vol. 20; 2011.

404. Osaki K, Hattori T, Kosen S: The role of home-based records in the establishment of a continuum of care for mothers, newborns, and children in Indonesia. *Glob Health Action* 2013, 6(1):1-12.

For peer review only - http://bmjopen.bmj.com/site/about/guidelines.xhtml

405. Osório S, Júnior L, Nicolau A: Assessment of the effectiveness of non-pharmacological methods in pain relief during labor. *Revista da Rede de Enfermagem do Nordeste* 2014, 15:174-184.

406. Osterman RL, Carle AC, Ammerman RT, Gates D: Single-session motivational intervention to decrease alcohol use during pregnancy. *J Subst Abuse Treat* 2014, 47(1):10-19.

407. Owais S, Chow CHT, Furtado M, Frey BN, Van Lieshout RJ: Non-pharmacological interventions for improving postpartum maternal sleep: A systematic review and meta-analysis. In., vol. 41; 2018.

408. Palacios C, Kostiuk LK, Pena-Rosas JP: Vitamin D supplementation for women during pregnancy. *Cochrane Database Syst Rev* 2019, 7:CD008873.

409. Palnati M, Marcus BH, Pekow P, Rosal MC, Manson JE, Chasan-Taber L: The Impact of a Lifestyle Intervention on Postpartum Weight Retention Among At-Risk Hispanic Women. *Am J Prev Med* 2021, 61(1):44-54.

410. Pan WL, Gau ML, Lee TY, Jou HJ, Liu CY, Wen TK: Mindfulness-based programme on the psychological health of pregnant women. *Women Birth* 2019, 32(1):e102-e109.

411. Parikh R, Rao SR, Kukde R, O'Connor GT, Patel A, Hibberd PL: Assessing the Respiratory Effects of Air Pollution from Biomass Cookstoves on Pregnant Women in Rural India. *Int J Environ Res Public Health* 2020, 18(1).

412. Park SJ, Lee J: The effects of health care programs for gestational diabetes mellitus in South Korea: a systematic review. *Korean Journal of Women Health Nursing* 2020, 26(4):274-284.

413. Passerini L, Casey GJ, Biggs BA, Cong DT, Phu LB, Phuc TQ, Carone M, Montresor A: Increased birth weight associated with regular pre-pregnancy deworming and weekly iron-folic acid supplementation for Vietnamese women. *PLoS Negl Trop Dis* 2012, 6(4):e1608.

414. Patel A, Goudar SS, Geller SE, Kodkany BS, Edlavitch SA, Wagh K, Patted SS, Naik VA, Moss N, Derman RJ: Drape estimation vs. visual assessment for estimating postpartum hemorrhage. *Int J Gynaecol Obstet* 2006, 93(3):220-224.

415. Peaceman AM, Clifton RG, Phelan S, Gallagher D, Evans M, Redman LM, Knowler WC, Joshipura K, Haire-Joshu D, Yanovski SZ *et al*: Lifestyle Interventions Limit Gestational Weight Gain in Women with Overweight or Obesity: LIFE-Moms Prospective Meta-Analysis. *Obesity (Silver Spring)* 2018, 26(9):1396-1404.

416. Pena-Rosas JP, De-Regil LM, Garcia-Casal MN, Dowswell T: Daily oral iron supplementation during pregnancy. *Cochrane Database Syst Rev* 2015(7):CD004736.

417. Peña-Rosas JP, De-Regil LM, Malave HG, Flores-Urrutia MC, Dowswell T: Intermittent oral iron supplementation during pregnancy. In., vol. 2015; 2015.

418. Pereira Gomes Morais E, Riera R, Porfírio GJM, Macedo CR, Sarmento Vasconcelos V, de Souza Pedrosa A, Torloni MR: Chewing gum for enhancing early recovery of bowel function after caesarean section. In., vol. 2016; 2016.

419. Phelan S, Hagobian T, Brannen A, Stewart A, Schmid B, Cedars L: Promoting Weight Loss Before Pregnancy: Feasible or Futile? *Californian Journal of Health Promotion* 2013, 11(2).

420. Phelan S, Phipps MG, Abrams B, Darroch F, Grantham K, Schaffner A, Wing RR: Does behavioral intervention in pregnancy reduce postpartum weight retention? Twelve-month outcomes of the Fit for Delivery randomized trial. *Am J Clin Nutr* 2014, 99(2):302-311.

421. Phipps MG, Raker CA, Ware CF, Zlotnick C: Randomized controlled trial to prevent postpartum depression in adolescent mothers. *Am J Obstet Gynecol* 2013, 208(3):192 e191-196.

422. Pilarczyk R, Strozik M, Hirnle L: Diagnostic equivalency of mobile CTG devices and remote analysis to conventional on-site nonstress test. *Adv Clin Exp Med* 2020, 29(1):33-44.

423. Pineles BL, Hsu S, Park E, Samet JM: Systematic review and meta-analyses of perinatal death and maternal exposure to tobacco smoke during pregnancy. In., vol. 184; 2016.

424. Polsky S, Garcetti R, Pyle L, Joshee P, Demmitt JK, Snell-Bergeon JK: Continuous glucose monitor use with and without remote monitoring in pregnant women with type 1 diabetes: A pilot study. *PLoS One* 2020, 15(4):e0230476.

For peer review only - http://bmjopen.bmj.com/site/about/guidelines.xhtml

425. Ragnar I, Altman D, Tyden T, Olsson SE: Comparison of the maternal experience and duration of labour in two upright delivery positions--a randomised controlled trial. *BJOG* 2006, 113(2):165-170.

426. Rajbhandari S, Hodgins S, Sanghvi H, McPherson R, Pradhan YV, Baqui AH, Misoprostol Study G: Expanding uterotonic protection following childbirth through community-based distribution of misoprostol: operations research study in Nepal. *Int J Gynaecol Obstet* 2010, 108(3):282-288.

427. Ramakrishnan U, Grant FK, Goldenberg T, Bui V, Imdad A, Bhutta ZA: Effect of multiple micronutrient supplementation on pregnancy and infant outcomes: a systematic review. *Paediatr Perinat Epidemiol* 2012, 26 Suppl 1(SUPPL. 1):153-167.

428. Raman P, Shepherd E, Dowswell T, Middleton P, Crowther CA: Different methods and settings for glucose monitoring for gestational diabetes during pregnancy. In., vol. 2017; 2017.

429. Ramanathan K, Sirala Jagadeesh N, Vishwanath U, Dayal C, Chandrababu R, Hayter M: Efficacy of supplementation of probiotics on maternal glycaemic control – A systematic review and meta-analysis of randomized controlled trials. In., vol. 10; 2021.

430. Rashtchi V, Maryami N, Molaei B: Comparison of entonox and transcutaneous electrical nerve stimulation (TENS) in labor pain: a randomized clinical trial study. *J Matern Fetal Neonatal Med* 2022, 35(16):3124-3128.

431. Rasmussen L, Christensen ML, Poulsen CW, Rud C, Christensen AS, Andersen JR, Kampmann U, Ovesen PG: Effect of High Versus Low Carbohydrate Intake in the Morning on Glycemic Variability and Glycemic Control Measured by Continuous Blood Glucose Monitoring in Women with Gestational Diabetes Mellitus-A Randomized Crossover Study. *Nutrients* 2020, 12(2).

432. Rasouli M, Pourheidari M, Hamzeh Gardesh Z: Effect of Self-care Before and During Pregnancy to Prevention and Control Preeclampsia in High-risk Women. *Int J Prev Med* 2019, 10:21.

433. Redman LM, Drews KL, Klein S, Horn LV, Wing RR, Pi-Sunyer X, Evans M, Joshipura K, Arteaga SS, Cahill AG *et al*: Attenuated early pregnancy weight gain by prenatal lifestyle interventions does not prevent gestational diabetes in the LIFE-Moms consortium. *Diabetes Res Clin Pract* 2021, 171:108549.

434. Revello MG, Tibaldi C, Masuelli G, Frisina V, Sacchi A, Furione M, Arossa A, Spinillo A, Klersy C, Ceccarelli M *et al*: Prevention of Primary Cytomegalovirus Infection in Pregnancy. *EBioMedicine* 2015, 2(9):1205-1210.

435. Rezaeean SM, Abedian Z, Latifnejad-Roudsari R, Mazloum SR, Abbasi Z: The Effect of Prenatal Self-Care Based on Orem's Theory on Preterm Birth Occurrence in Women at Risk for Preterm Birth. *Iran J Nurs Midwifery Res* 2020, 25(3):242-248.

436. Rigla M, Martinez-Sarriegui I, Garcia-Saez G, Pons B, Hernando ME: Gestational Diabetes Management Using Smart Mobile Telemedicine. *J Diabetes Sci Technol* 2018, 12(2):260-264.

437. Robertson N, Okano S, Kumar S: Sleep in the Supine Position during Pregnancy Is Associated with Fetal Cerebral Redistribution. *J Clin Med* 2020, 9(6).

438. Rodriguez-Blanque R, Sanchez-Garcia JC, Sanchez-Lopez AM, Mur-Villar N, Aguilar-Cordero MJ: The influence of physical activity in water on sleep quality in pregnant women: A randomised trial. *Women Birth* 2018, 31(1):e51-e58.

439. Rondung E, Ternstrom E, Hildingsson I, Haines HM, Sundin O, Ekdahl J, Karlstrom A, Larsson B, Segeblad B, Baylis R *et al*: Comparing Internet-Based Cognitive Behavioral Therapy With Standard Care for Women With Fear of Birth: Randomized Controlled Trial. *JMIR Ment Health* 2018, 5(3):e10420.

440. Rong L, Wang R, Ouyang YQ, Redding SR: Efficacy of yoga on physiological and psychological discomforts and delivery outcomes in Chinese primiparas. *Complement Ther Clin Pract* 2021, 44:101434.

441. Rotheram-Borus MJ, Tomlinson M, le Roux IM, Harwood JM, Comulada S, O'Connor MJ, Weiss RE, Worthman CM: A cluster randomised controlled effectiveness trial evaluating perinatal home visiting among South African mothers/infants. *PLoS One* 2014, 9(10):e105934.

442. Rumbold A, Ota E, Hori H, Miyazaki C, Crowther CA: Vitamin E supplementation in pregnancy. In., vol. 2016; 2015.

For peer review only

443. Rungsiprakarn P, Laopaiboon M, Sangkomkamhang US, Lumbiganon P, Pratt JJ: Interventions for treating constipation in pregnancy. In., vol. 2015; 2015.
444. Saastad E, Israel P, Ahlborg T, Gunnes N, Froen JF: Fetal movement counting--effects on maternal-fetal attachment: a multicenter randomized controlled trial. *Birth* 2011, 38(4):282-293.
445. Saccone G, Berghella V, Venturella R, D'Alessandro P, Arduino B, Raffone A, Giudicepietro A, Visentin S, Vitagliano A, Martinelli P *et al*: Effects of exercise during pregnancy in women with short cervix: Secondary analysis from the Italian Pessary Trial in singletons. *Eur J Obstet Gynecol Reprod Biol* 2018, 229:132-136.
446. Saccone G, Caissutti C, Ciardulli A, Berghella V: Uterine massage for preventing postpartum hemorrhage at cesarean delivery: Which evidence? In., vol. 223; 2018.
447. Sagedal LR, Overby NC, Bere E, Torstveit MK, Lohne-Seiler H, Smastuen M, Hillesund ER, Henriksen T, Vistad I: Lifestyle intervention to limit gestational weight gain: the Norwegian Fit for Delivery randomised controlled trial. *BJOG* 2017, 124(1):97-109.
448. Sajitha AT, Philip AT, Sarika TK: Effectiveness of Prenatal Education Regarding Practice on Antenatal Exercises and Minor Ailments among Pregnant Mothers. *Journal of Clinical and Diagnostic Research* 2018, 12(9).
449. Salam RA, Zuberi NF, Bhutta ZA: Pyridoxine (vitamin B6) supplementation during pregnancy or labour for maternal and neonatal outcomes. In., vol. 2016; 2015.
450. Samawi L, Williams PP, Myers B, Fuhr DC: Effectiveness of psychological interventions to reduce alcohol consumption among pregnant and postpartum women: a systematic review. In., vol. 24; 2021.
451. Sampselle CM, Miller JM, Mims BL, Delancey JO, Ashton-Miller JA, Antonakos CL: Effect of pelvic muscle exercise on transient incontinence during pregnancy and after birth. *Obstet Gynecol* 1998, 91(3):406-412.
452. Sandborg J, Soderstrom E, Henriksson P, Bendtsen M, Henstrom M, Leppanen MH, Maddison R, Migueles JH, Blomberg M, Lof M: Effectiveness of a Smartphone App to Promote Healthy Weight Gain, Diet, and Physical Activity During Pregnancy (HealthyMoms): Randomized Controlled Trial. *JMIR Mhealth Uhealth* 2021, 9(3):e26091.
453. Sanghvi H, Ansari N, Prata NJ, Gibson H, Ehsan AT, Smith JM: Prevention of postpartum hemorrhage at home birth in Afghanistan. *Int J Gynaecol Obstet* 2010, 108(3):276-281.
454. Sapkota D, Baird K, Saito A, Anderson D: Interventions for reducing and/or controlling domestic violence among pregnant women in low- and middle-income countries: A systematic review. In., vol. 8; 2019.
455. Sapkota D, Baird K, Saito A, Rijal P, Anderson D: Antenatal-Based Pilot Psychosocial Intervention to Enhance Mental Health of Pregnant Women Experiencing Domestic and Family Violence in Nepal. *J Interpers Violence* 2022, 37(5-6):NP3605-NP3627.
456. Sarkar A, Mburu G, Shivkumar PV, Sharma P, Campbell F, Behera J, Dargan R, Mishra SK, Mehra S: Feasibility of supervised self-testing using an oral fluid-based HIV rapid testing method: a cross-sectional, mixed method study among pregnant women in rural India. *J Int AIDS Soc* 2016, 19(1):20993.
457. Scott EM, Feig DS, Murphy HR, Law GR: Continuous glucose monitoring in pregnancy: Importance of analyzing temporal profiles to understand clinical outcomes. In*: 2020*.
458. Secher AL, Ringholm L, Andersen HU, Damm P, Mathiesen ER: The effect of real-time continuous glucose monitoring in pregnant women with diabetes: a randomized controlled trial. *Diabetes Care* 2013, 36(7):1877-1883.
459. Sehhatie F, Mirgafourvand M, Vosoughi Niri Z: The Effect of Non-Pharmaceutical Methods of Labor Pain Relief on Mothers' Postpartum Depression: A Randomized Controlled Trial. *International Journal of Women's Health and Reproduction Sciences* 2015, 3(1):48-55.
460. Seneviratne SN, Jiang Y, Derraik J, McCowan L, Parry GK, Biggs JB, Craigie S, Gusso S, Peres G, Rodrigues RO *et al*: Effects of antenatal exercise in overweight and obese pregnant women on maternal and perinatal outcomes: a randomised controlled trial. *BJOG* 2016, 123(4):588-597.

For peer review only - http://bmjopen.bmj.com/site/about/guidelines.xhtml

461. Senol DK, Aslan E: The Effects of Cold Application to the Perineum on Pain Relief After Vaginal Birth. *Asian Nurs Res (Korean Soc Nurs Sci)* 2017, 11(4):276-282.

462. Sercekus P, Baskale H: Effects of antenatal education on fear of childbirth, maternal self-efficacy and parental attachment. *Midwifery* 2016, 34:166-172.

463. Serra R, Peñailillo R, Monteiro LJ, Monckeberg M, Peña M, Moyano L, Brunner C, Vega G, Choolani M, Illanes SE: Supplementation of omega 3 during pregnancy and the risk of preterm birth: A systematic review and meta-analysis. In., vol. 13; 2021.

464. Sethi V, Bhanot A, Bhattacharjee S, Gope R, Sarangi D, Nath V, Nair N, Singh U, Daniel A, Parhi RN *et al*: Integrated multisectoral strategy to improve girls' and women's nutrition before conception, during pregnancy and after birth in India (Swabhimaan): protocol for a prospective, non-randomised controlled evaluation. *BMJ Open* 2019, 9(11):e031632.

465. Shahsavan F, Akbari N, Gharraee B, Abolghasemi J, Khedmat L: The effect of internet-based guided self-help cognitive-behavioral therapies on Iranian women's psychological symptoms and preferred method of childbirth. *Perspect Psychiatr Care* 2021, 57(1):138-147.

466. Sharps PW, Bullock LF, Campbell JC, Alhusen JL, Ghazarian SR, Bhandari SS, Schminkey DL: Domestic Violence Enhanced Perinatal Home Visits: The DOVE Randomized Clinical Trial. *J Womens Health (Larchmt)* 2016, 25(11):1129-1138.

467. Shedmake PV, Wakode SR: A Hospital-Based Randomized Controlled Trial-Comparing the Outcome of Normal Delivery Between Squatting and Lying Down Positions During Labour. *J Obstet Gynaecol India* 2021, 71(4):393-398.

468. Shirah BH, Shirah HA, Fallata AH, Alobidy SN, Hawsawi MMA: Hemorrhoids during pregnancy: Sitz bath vs. ano-rectal cream: A comparative prospective study of two conservative treatment protocols. *Women Birth* 2018, 31(4):e272-e277.

469. Shivalli S, Srivastava RK, Singh GP: Trials of Improved Practices (TIPs) to Enhance the Dietary and Iron-Folate Intake during Pregnancy- A Quasi Experimental Study among Rural Pregnant Women of Varanasi, India. *PLoS One* 2015, 10(9):e0137735.

470. Shorey S, Chee CYI, Ng ED, Lau Y, Dennis CL, Chan YH: Evaluation of a Technology-Based Peer-Support Intervention Program for Preventing Postnatal Depression (Part 1): Randomized Controlled Trial. *J Med Internet Res* 2019, 21(8):e12410.

471. Shorten A, Shorten B, Keogh J, West S, Morris J: Making choices for childbirth: a randomized controlled trial of a decision-aid for informed birth after cesarean. *Birth* 2005, 32(4):252-261.

472. Sibley L, Buffington ST, Haileyesus D: The American College of Nurse-Midwives' home-based lifesaving skills program: a review of the Ethiopia field test. *J Midwifery Womens Health* 2004, 49(4):320-328.

473. Siddika N, Balogun HA, Amegah AK, Jaakkola JJK: Prenatal ambient air pollution exposure and the risk of stillbirth: Systematic review and meta-analysis of the empirical evidence. In., vol. 73; 2016.

474. Sidiki NNA, Payne VK, Cedric Y, Nadia NAC: Effect of Impregnated Mosquito Bed Nets on the Prevalence of Malaria among Pregnant Women in Foumban Subdivision, West Region of Cameroon. *J Parasitol Res* 2020, 2020:7438317.

475. Silver RM, Hunter S, Reddy UM, Facco F, Gibbins KJ, Grobman WA, Mercer BM, Haas DM, Simhan HN, Parry S *et al*: Prospective Evaluation of Maternal Sleep Position Through 30 Weeks of Gestation and Adverse Pregnancy Outcomes. *Obstet Gynecol* 2019, 134(4):667-676.

476. Simpson SA, Coulman E, Gallagher D, Jewell K, Cohen D, Newcombe RG, Huang C, Robles-Zurita JA, Busse M, Owen-Jones E *et al*: Healthy eating and lifestyle in pregnancy (HELP): a cluster randomised trial to evaluate the effectiveness of a weight management intervention for pregnant women with obesity on weight at 12 months postpartum. *Int J Obes (Lond)* 2021, 45(8):1728-1739.

477. Skouteris H, Hartley-Clark L, McCabe M, Milgrom J, Kent B, Herring SJ, Gale J: Preventing excessive gestational weight gain: a systematic review of interventions. *Obes Rev* 2010, 11(11):757-768.

For peer review only - http://bmjopen.bmj.com/site/about/guidelines.xhtml

478. Skouteris H, Morris H, Nagle C, Nankervis A: Behavior modification techniques used to prevent gestational diabetes: A systematic review of the literature topical collection on obesity. In., vol. 14; 2014.
479. Skovbjerg S, Birk D, Bruggisser S, Wolf ALA, Fjorback L: Mindfulness-based stress reduction adapted to pregnant women with psychosocial vulnerabilities-a protocol for a randomized feasibility study in a Danish hospital-based outpatient setting. *Pilot Feasibility Stud* 2021, 7(1):118.
480. Slade P, West H, Thomson G, Lane S, Spiby H, Edwards RT, Charles JM, Garrett C, Flanagan B, Treadwell M *et al*: STRAWB2 (Stress and Wellbeing After Childbirth): a randomised controlled trial of targeted self-help materials to prevent post-traumatic stress disorder following childbirth. *BJOG* 2020, 127(7):886-896.
481. Smith CA, Collins CT, Crowther CA: Aromatherapy for pain management in labour. *Cochrane Database Syst Rev* 2011, 14(7):CD009215.
482. Smith CA, Collins CT, Levett KM, Armour M, Dahlen HG, Tan AL, Mesgarpour B: Acupuncture or acupressure for pain management during labour. In., vol. 2020; 2020.
483. Smith CA, Hill E, Denejkina A, Thornton C, Dahlen HG: The effectiveness and safety of complementary health approaches to managing postpartum pain: A systematic review and meta-analysis. In., vol. 11; 2022.
484. Smith CA, Levett KM, Collins CT, Armour M, Dahlen HG, Suganuma M: Relaxation techniques for pain management in labour. In., vol. 2018; 2018.
485. Smith CA, Levett KM, Collins CT, Dahlen HG, Ee CC, Suganuma M: Massage, reflexology and other manual methods for pain management in labour. In., vol. 2018; 2018.
486. Smith ER, Shankar AH, Wu LS, Aboud S, Adu-Afarwuah S, Ali H, Agustina R, Arifeen S, Ashorn P, Bhutta ZA *et al*: Modifiers of the effect of maternal multiple micronutrient supplementation on stillbirth, birth outcomes, and infant mortality: a meta-analysis of individual patient data from 17 randomised trials in low-income and middle-income countries. *Lancet Glob Health* 2017, 5(11):e1090-e1100.
487. Smith JM, Gubin R, Holston MM, Fullerton J, Prata N: Misoprostol for postpartum hemorrhage prevention at home birth: an integrative review of global implementation experience to date. *BMC Pregnancy Childbirth* 2013, 13:44.
488. Smith SA, Michel Y: A pilot study on the effects of aquatic exercises on discomforts of pregnancy. *J Obstet Gynecol Neonatal Nurs* 2006, 35(3):315-323.
489. Smyth RMD, Aflaifel N, Bamigboye AA: Interventions for varicose veins and leg oedema in pregnancy. In., vol. 2015; 2015.
490. Sobhgol SS, Priddis H, Smith CA, Dahlen HG: Evaluation of the effect of an antenatal pelvic floor muscle exercise programme on female sexual function during pregnancy and the first 3 months following birth: study protocol for a pragmatic randomised controlled trial. *Trials* 2019, 20(1):144.
491. Soltani H, Scott AM: Antenatal breast expression in women with diabetes: outcomes from a retrospective cohort study. *Int Breastfeed J* 2012, 7(1):18.
492. Srisopa P, Cong X, Russell B, Lucas R: The Role of Emotion Regulation in Pain Management Among Women From Labor to Three Months Postpartum: An Integrative Review. *Pain Manag Nurs* 2021, 22(6):783-790.
493. Stade BC, Bailey C, Dzendoletas D, Sgro M, Dowswell T, Bennett D: Psychological and/or educational interventions for reducing alcohol consumption in pregnant women and women planning pregnancy. In.; 2009.
494. Steen M, Briggs M, King D: Alleviating postnatal perineal trauma: To cool or not to cool? In., vol. 14; 2006.
495. Steen M, Cooper K, Marchant P, Griffiths-Jones M, Walker J: A randomised controlled trial to compare the effectiveness of ice-packs and Epifoam with cooling maternity gel pads at alleviating postnatal perineal trauma. *Midwifery* 2000, 16(1):48-55.
496. Steen M, Robinson M, Robertson S, Raine G: Pre and post survey findings from the Mind 'Building resilience programme for better mental health: Pregnant women and new mothers'. In., vol. 13; 2015.
497. Stith BJ, Buls SM, Keim SA, Thung SF, Klebanoff MA, Landon MB, Gabbe SG, Gandhi KK, Oza-Frank R: Moms in motion: weight loss intervention for postpartum mothers after gestational diabetes: a randomized controlled trial. *BMC Pregnancy Childbirth* 2021, 21(1):461.

For peer review only - http://bmjopen.bmj.com/site/about/guidelines.xhtml

498. Strauss RA, Eucker B, Savitz DA, Thorp JM, Jr.: Diagnosis of bacterial vaginosis from self-obtained vaginal swabs. *Infect Dis Obstet Gynecol* 2005, 13(1):31-35.

499. Sun Y, Li Y, Wang J, Chen Q, Bazzano AN, Cao F: Effectiveness of Smartphone-Based Mindfulness Training on Maternal Perinatal Depression: Randomized Controlled Trial. *J Med Internet Res* 2021, 23(1):e23410.

500. Sun YC, Hung YC, Chang Y, Kuo SC: Effects of a prenatal yoga programme on the discomforts of pregnancy and maternal childbirth self-efficacy in Taiwan. *Midwifery* 2010, 26(6):e31-36.

501. Sungkar A, Purwosunu Y, Aziz MF, Pratomo H, Sutrisna B, Sekizawa A: Influence of early self-diagnosis and treatment of bacterial vaginosis on preterm birth rate. *Int J Gynaecol Obstet* 2012, 117(3):264-267.

502. Suntarattiwong P, Mott JA, Mohanty S, Sinthuwattanawibool C, Srisantiroj N, Patamasingh Na Ayudhaya O, Klungthong C, Fernandez S, Kim L, Hunt D *et al*: Feasibility and Performance of Self-Collected Nasal Swabs for Detection of Influenza Virus, Respiratory Syncytial Virus, and Human Metapneumovirus. *J Infect Dis* 2021, 224(5):831-838.

503. Surkan PJ, Hamdani SU, Huma ZE, Nazir H, Atif N, Rowther AA, Chaudhri R, Zafar S, Mullany LC, Malik A *et al*: Cognitive-behavioral therapy-based intervention to treat symptoms of anxiety in pregnancy in a prenatal clinic using non-specialist providers in Pakistan: design of a randomised trial. *BMJ Open* 2020, 10(4):e037590.

504. Sushko K, Menezes HT, Strachan P, Butt M, Sherifali D: Self-management education among women with pre-existing diabetes in pregnancy: A scoping review. In., vol. 117; 2021.

505. Syngelaki A, Sequeira Campos M, Roberge S, Andrade W, Nicolaides KH: Diet and exercise for preeclampsia prevention in overweight and obese pregnant women: systematic review and meta-analysis. In., vol. 32; 2019.

506. Szkwara JM, Milne N, Rathbone E: A prospective quasi-experimental controlled study evaluating the use of dynamic elastomeric fabric orthoses to manage common postpartum ailments during postnatal care. *Womens Health (Lond)* 2020, 16:1745506520927196.

507. Szumilewicz A, Dornowski M, Piernicka M, Worska A, Kuchta A, Kortas J, Bludnicka M, Radziminski L, Jastrzebski Z: High-Low Impact Exercise Program Including Pelvic Floor Muscle Exercises Improves Pelvic Floor Muscle Function in Healthy Pregnant Women - A Randomized Control Trial. *Front Physiol* 2018, 9:1867.

508. Taavoni S, Sheikhan F, Abdolahian S, Ghavi F: Birth ball or heat therapy? A randomized controlled trial to compare the effectiveness of birth ball usage with sacrum-perineal heat therapy in labor pain management. *Complement Ther Clin Pract* 2016, 24:99-102.

509. Taheri Z, Mazaheri MA, Khorsandi M, Hassanzadeh A, Amiri M: Effect of educational intervention on self-efficacy for choosing delivery method among pregnant women in 2013. *International Journal of Preventive Medicine* 2014, 5(10).

510. Tan PC, Andi A, Azmi N, Noraihan MN: Effect of coitus at term on length of gestation, induction of labor, and mode of delivery. *Obstet Gynecol* 2006, 108(1):134-140.

511. Tan XYJ, Choong SYX, Cheng LJ, Lau Y: Relaxation interventions for improving sleep outcomes in perinatal women: A systematic review and meta-analysis of randomized controlled trials. In., vol. 103; 2021.

512. Tanvig MH, Jensen DM, Andersen MS, Ovesen PG, Jorgensen JS, Vinter CA: Vitamin D levels were significantly higher during and after lifestyle intervention in pregnancy: A randomized controlled trial. *Acta Obstet Gynecol Scand* 2020, 99(3):350-356.

513. Tatala SR, Ash D, Makola D, Latham M, Ndosi G, Grohn Y: Effect of micronutrient fortified beverage on nutritional anaemia during pregnancy. *East Afr Med J* 2002, 79(11):598-603.

514. ter Kuile FO, Terlouw DJ, Phillips-Howard PA, Hawley WA, Friedman JF, Kariuki SK, Shi YP, Kolczak MS, Lal AA, Vulule JM *et al*: Reduction of malaria during pregnancy by permethrin-treated bed nets in an area of intense perennial malaria transmission in western Kenya. *Am J Trop Med Hyg* 2003, 68(4 Suppl):50-60.

515. Thompson MG, Ferber JR, Odouli R, David D, Shifflett P, Meece JK, Naleway AL, Bozeman S, Spencer SM, Fry AM *et al*: Results of a pilot study using self-collected mid-turbinate nasal swabs for detection of influenza virus infection among pregnant women. *Influenza Other Respir Viruses* 2015, 9(3):155-160.

For peer review only - http://bmjopen.bmj.com/site/about/guidelines.xhtml

516. Timur Tashan S, Kafkasli A: The effect of bitter almond oil and massaging on striae gravidarum in primiparaous women. *J Clin Nurs* 2012, 21(11-12):1570-1576.

517. Tinius R, Edens K, Link K, Jones MS, Lyons S, Rebelle T, Pearson KJ, Maples J: Effect of Evidence-Based Materials and Access to Local Resources on Physical Activity Levels, Beliefs, and Motivation During Pregnancy in a Rural Setting. *J Phys Act Health* 2020, 17(10):947-957.

518. Tixier H, Boucard C, Ferdynus C, Douvier S, Sagot P: Interest of using an underbuttocks drape with collection pouch for early diagnosis of postpartum hemorrhage. *Arch Gynecol Obstet* 2011, 283(1):25-29.

519. Todd CS, Chowdhury Z, Mahmud Z, Islam N, Shabnam S, Parvin M, Bernholc A, Martinez A, Aktar B, Afsana K *et al*: Maternal nutrition intervention and maternal complications in 4 districts of Bangladesh: A nested cross-sectional study. *PLoS Med* 2019, 16(10):e1002927.

520. Tragea C, Chrousos GP, Alexopoulos EC, Darviri C: A randomized controlled trial of the effects of a stress management programme during pregnancy. *Complement Ther Med* 2014, 22(2):203-211.

521. Tran K, Padwal R, Khan N, Wright MD, Chan WS: Home blood pressure monitoring in the diagnosis and treatment of hypertension in pregnancy: a systematic review and meta-analysis. *CMAJ Open* 2021, 9(2):E642-E650.

522. Tsai YJ, Hsu YY, Hou TW, Chang CH: Effects of a Web-Based Antenatal Care System on Maternal Stress and Self-Efficacy During Pregnancy: A Study in Taiwan. *J Midwifery Womens Health* 2018, 63(2):205-213.

523. Tseng YF, Chen CH, Lee CS: Effects of listening to music on postpartum stress and anxiety levels. *J Clin Nurs* 2010, 19(7-8):1049-1055.

524. Tucker KL, Bowen L, Crawford C, Mallon P, Hinton L, Lee MM, Oke J, Taylor KS, Heneghan C, Bankhead C *et al*: The feasibility and acceptability of self-testing for proteinuria during pregnancy: A mixed methods approach. *Pregnancy Hypertens* 2018, 12:161-168.

525. Tucker KL, Taylor KS, Crawford C, Hodgkinson JA, Bankhead C, Carver T, Ewers E, Glogowska M, Greenfield SM, Ingram L *et al*: Blood pressure self-monitoring in pregnancy: examining feasibility in a prospective cohort study. *BMC Pregnancy Childbirth* 2017, 17(1):442.

526. Tumminia A, Milluzzo A, Festa C, Fresa R, Pintaudi B, Scavini M, Vitacolonna E, Napoli A, Sciacca L: Efficacy of flash glucose monitoring in pregnant women with poorly controlled pregestational diabetes (FlashMom): A randomized pilot study. *Nutr Metab Cardiovasc Dis* 2021, 31(6):1851-1859.

527. Uğurlu M, Yavan T, Karaşahin KE: The effect of an education and counseling program on maternal/neonatal outcomes in pregnant women at risk of preeclampsia. *Puerto Rico Health Sciences Journal* 2021, 40(3).

528. Vakilian K, Atarha M, Bekhradi R, Chaman R: Healing advantages of lavender essential oil during episiotomy recovery: a clinical trial. *Complement Ther Clin Pract* 2011, 17(1):50-53.

529. Vamos CA, Thompson EL, Avendano M, Daley EM, Quinonez RB, Boggess K: Oral health promotion interventions during pregnancy: A systematic review. In., vol. 43; 2015.

530. Van der Pligt P, Willcox J, Hesketh KD, Ball K, Wilkinson S, Crawford D, Campbell K: Systematic review of lifestyle interventions to limit postpartum weight retention: Implications for future opportunities to prevent maternal overweight and obesity following childbirth. In., vol. 14; 2013.

531. Van Der Windt M, Van Der Kleij RMJJ, Snoek KM, Dykgraaf RHM, Laven JSE, Schoenmakers S, Steegers-Theunissen RPM: Impact of a blended periconception lifestyle care approach combining face-to-face counseling with ehealth. *Reproductive Sciences* 2020, 27(1).

532. Van Horn L, Peaceman A, Kwasny M, Vincent E, Fought A, Josefson J, Spring B, Neff LM, Gernhofer N: Dietary Approaches to Stop Hypertension Diet and Activity to Limit Gestational Weight: Maternal Offspring Metabolics Family Intervention Trial, a Technology Enhanced Randomized Trial. *Am J Prev Med* 2018, 55(5):603-614.

533. Van Parys AS, Verhamme A, Temmerman M, Verstraelen H: Intimate partner violence and pregnancy: A systematic review of interventions. In., vol. 9; 2014.

For peer review only - http://bmjopen.bmj.com/site/about/guidelines.xhtml

534. van Poppel MN, Jelsma JGM, Simmons D, Devlieger R, Jans G, Galjaard S, Corcoy R, Adelantado JM, Dunne F, Harreiter J *et al*: Mediators of Lifestyle Behaviour Changes in Obese Pregnant Women. Secondary Analyses from the DALI Lifestyle Randomised Controlled Trial. *Nutrients* 2019, 11(2).
535. Varisoglu Y, Gungor Satilmis I: The Effects of Listening to Music on Breast Milk Production by Mothers of Premature Newborns in the Neonatal Intensive Care Unit: A Randomized Controlled Study. *Breastfeed Med* 2020, 15(7):465-470.
536. Vasegh Rahimparvar SF, Hamzehkhani M, Geranmayeh M, Rahimi R: Effect of educational software on self-efficacy of pregnant women to cope with labor: a randomized controlled trial. *Arch Gynecol Obstet* 2012, 286(1):63-70.
537. Vauloup-Fellous C, Picone O, Cordier AG, Parent-du-Chatelet I, Senat MV, Frydman R, Grangeot-Keros L: Does hygiene counseling have an impact on the rate of CMV primary infection during pregnancy? Results of a 3-year prospective study in a French hospital. *J Clin Virol* 2009, 46 Suppl 4(SUPPL. 4):S49-53.
538. Vazquez JC: Constipation, haemorrhoids, and heartburn in pregnancy. *BMJ Clin Evid* 2010, 2010.
539. Veisani Y, Jenabi E, Delpisheh A, Khazaei S: Effect of prenatal smoking cessation interventions on birth weight: meta-analysis. In., vol. 32; 2019.
540. Vesco KK, Karanja N, King JC, Gillman MW, Leo MC, Perrin N, McEvoy CT, Eckhardt CL, Smith KS, Stevens VJ: Efficacy of a group-based dietary intervention for limiting gestational weight gain among obese women: a randomized trial. *Obesity (Silver Spring)* 2014, 22(9):1989-1996.
541. Vodopivec-Jamsek V, de Jongh T, Gurol-Urganci I, Atun R, Car J: Mobile phone messaging for preventive health care. In., vol. 2017; 2012.
542. Vrana-Diaz CJ, Korte JE, Gebregziabher M, Richey L, Selassie A, Sweat M, Gichangi A: Relationship Gender Equality and Couples' Uptake of Oral Human Immunodeficiency Virus Self-Testing Kits Delivered by Pregnant Women in Kenya. *Sex Transm Dis* 2019, 46(9):588-593.
543. Wan CS, Nankervis A, Teede H, Aroni R: Dietary intervention strategies for ethnic Chinese women with gestational diabetes mellitus: A systematic review and meta-analysis. In., vol. 76; 2019.
544. Wang X, Xu X, Luo J, Chen Z, Feng S: Effect of app-based audio guidance pelvic floor muscle training on treatment of stress urinary incontinence in primiparas: A randomized controlled trial. *Int J Nurs Stud* 2020, 104:103527.
545. Wang Z, Liu Q, Min L, Mao X: The effectiveness of the laid-back position on lactation-related nipple problems and comfort: a meta-analysis. *BMC Pregnancy Childbirth* 2021, 21(1):248.
546. Wannakosit S, Phupong V: Sexual behavior in pregnancy: comparing between sexual education group and nonsexual education group. *J Sex Med* 2010, 7(10):3434-3438.
547. Warland J, Dorrian J, Morrison JL, O'Brien LM: Maternal sleep during pregnancy and poor fetal outcomes: A scoping review of the literature with meta-analysis. In., vol. 41; 2018.
548. Webber GC, Chirangi B: Women's health in women's hands: a pilot study assessing the feasibility of providing women with medications to reduce postpartum hemorrhage and sepsis in rural Tanzania. *Health Care Women Int* 2014, 35(7-9):758-770.
549. Weeks AD, Ditai J, Ononge S, Faragher B, Frye LJ, Durocher J, Mirembe FM, Byamugisha J, Winikoff B, Alfirevic Z: The MamaMiso study of self-administered misoprostol to prevent bleeding after childbirth in rural Uganda: a community-based, placebo-controlled randomised trial. *BMC Pregnancy Childbirth* 2015, 15:219.
550. Werner A, Uldbjerg N, Zachariae R, Rosen G, Nohr EA: Self-hypnosis for coping with labour pain: a randomised controlled trial. *BJOG* 2013, 120(3):346-353.
551. Whitford HM, Alder B, Jones M: A longitudinal follow up of women in their practice of perinatal pelvic floor exercises and stress urinary incontinence in North-East Scotland. *Midwifery* 2007, 23(3):298-308.
552. Willcox JC, Wilkinson SA, Lappas M, Ball K, Crawford D, McCarthy EA, Fjeldsoe B, Whittaker R, Maddison R, Campbell KJ: A mobile health intervention promoting healthy gestational weight gain for women entering

For peer review only - http://bmjopen.bmj.com/site/about/guidelines.xhtml

pregnancy at a high body mass index: the txt4two pilot randomised controlled trial. *BJOG* 2017, 124(11):1718-1728.

553. Windsor R, Woodby L, Miller T, Hardin M: Effectiveness of Smoking Cessation and Reduction in Pregnancy Treatment (SCRIPT) methods in Medicaid-supported prenatal care: Trial III. *Health Educ Behav* 2011, 38(4):412-422.

554. Windsor RA, Cutter G, Morris J, Reese Y, Manzella B, Bartlett EE, Samuelson C, Spanos D: The effectiveness of smoking cessation methods for smokers in public health maternity clinics: a randomized trial. *Am J Public Health* 1985, 75(12):1389-1392.

555. Wing DA, Rumney PJ, Preslicka CW, Chung JH: Daily cranberry juice for the prevention of asymptomatic bacteriuria in pregnancy: a randomized, controlled pilot study. *J Urol* 2008, 180(4):1367-1372.

556. Witt AM, Bolman M, Kredit S: Mothers Value and Utilize Early Outpatient Education on Breast Massage and Hand Expression in Their Self-Management of Engorgement. *Breastfeed Med* 2016, 11(9):433-439.

557. Woodley SJ, Lawrenson P, Boyle R, Cody JD, Mørkved S, Kernohan A, Hay-Smith EJC: Pelvic floor muscle training for preventing and treating urinary and faecal incontinence in antenatal and postnatal women. In., vol. 2020; 2020.

558. Woolhouse H, Mercuri K, Judd F, Brown SJ: Antenatal mindfulness intervention to reduce depression, anxiety and stress: a pilot randomised controlled trial of the MindBabyBody program in an Australian tertiary maternity hospital. *BMC Pregnancy Childbirth* 2014, 14(1):369.

559. Wulff V, Hepp P, Wolf OT, Balan P, Hagenbeck C, Fehm T, Schaal NK: The effects of a music and singing intervention during pregnancy on maternal well-being and mother-infant bonding: a randomised, controlled study. *Arch Gynecol Obstet* 2021, 303(1):69-83.

560. Yakoob MY, Qadir M, Hany OE: Vitamin A Supplementation for Prevention and Treatment of Malaria during Pregnancy and Childhood: A Systematic Review and Meta-analysis. *J Epidemiol Glob Health* 2018, 8(1-2):20-28.

561. Yan CF, Hung YC, Gau ML, Lin KC: Effects of a stability ball exercise programme on low back pain and daily life interference during pregnancy. *Midwifery* 2014, 30(4):412-419.

562. Yang X, Tian H, Zhang F, Zhang C, Li Y, Leng J, Wang L, Liu G, Dong L, Yu Z *et al*: A randomised translational trial of lifestyle intervention using a 3-tier shared care approach on pregnancy outcomes in Chinese women with gestational diabetes mellitus but without diabetes. *J Transl Med* 2014, 12(1):290.

563. Yaping X, Huifen Z, Meijing Z, Huibin H, Chunhong L, Fengfeng H, Jingjing W: Effects of Moderate-Intensity Aerobic Exercise on Blood Glucose Levels and Pregnancy Outcomes in Patients With Gestational Diabetes Mellitus: A Randomized Controlled Trial. *Diabetes Ther* 2021, 12(9):2585-2598.

564. Yerlikaya-Schatten G, Trimmal L, Rosicky I, Husslein P, Schatten C, Eppel D, Eppel W, Tura A, Gobl CS: Effects of gum chewing on glycaemic control in women with gestational diabetes mellitus: A randomized controlled trial. Impact of chewing on hyperglycaemia in women with GDM. *Eur J Obstet Gynecol Reprod Biol* 2020, 247:61-65.

565. Yew TW, Chi C, Chan SY, van Dam RM, Whitton C, Lim CS, Foong PS, Fransisca W, Teoh CL, Chen J *et al*: A Randomized Controlled Trial to Evaluate the Effects of a Smartphone Application-Based Lifestyle Coaching Program on Gestational Weight Gain, Glycemic Control, and Maternal and Neonatal Outcomes in Women With Gestational Diabetes Mellitus: The SMART-GDM Study. *Diabetes Care* 2021, 44(2):456-463.

566. Yuksel H, Cayir Y, Kosan Z, Tastan K: Effectiveness of breathing exercises during the second stage of labor on labor pain and duration: a randomized controlled trial. *J Integr Med* 2017, 15(6):456-461.

567. Zairina E, Abramson MJ, McDonald CF, Li J, Dharmasiri T, Stewart K, Walker SP, Paul E, George J: Telehealth to improve asthma control in pregnancy: A randomized controlled trial. *Respirology* 2016, 21(5):867-874.

568. Zairina E, Stewart K, Abramson MJ, George J: The effectiveness of non-pharmacological healthcare interventions for asthma management during pregnancy: a systematic review. *BMC Pulm Med* 2014, 14(1):46.

For peer review only - http://bmjopen.bmj.com/site/about/guidelines.xhtml

569. Zakarija-Grkovic I, Stewart F: Treatments for breast engorgement during lactation. *Cochrane Database Syst Rev* 2020, 9:CD006946.

570. Zang Y, Lu H, Zhang H, Huang J, Ren L, Li C: Effects of upright positions during the second stage of labour for women without epidural analgesia: A meta-analysis. In., vol. 76; 2020.

571. Zang Y, Lu H, Zhang H, Huang J, Zhao Y, Ren L: Benefits and risks of upright positions during the second stage of labour: An overview of systematic reviews. In., vol. 114; 2021.

572. Zarei MR, Khachian A, Mohammadi N, Haghani H, Rostami F: The effect of self-care education program on wound healing in women undergoing cesarean section. *Annals of Tropical Medicine and Public Health* 2018, 11(Special Issue).

573. Zarenejad M, Yazdkhasti M, Rahimzadeh M, Mehdizadeh Tourzani Z, Esmaelzadeh-Saeieh S: The effect of mindfulness-based stress reduction on maternal anxiety and self-efficacy: A randomized controlled trial. *Brain Behav* 2020, 10(4):e01561.

574. Zelkowitz P, Feeley N, Shrier I, Stremler R, Westreich R, Dunkley D, Steele R, Rosberger Z, Lefebvre F, Papageorgiou A: The cues and care randomized controlled trial of a neonatal intensive care unit intervention: effects on maternal psychological distress and mother-infant interaction. *J Dev Behav Pediatr* 2011, 32(8):591-599.

575. Zemestani M, Fazeli Nikoo Z: Effectiveness of mindfulness-based cognitive therapy for comorbid depression and anxiety in pregnancy: a randomized controlled trial. *Arch Womens Ment Health* 2020, 23(2):207-214.

576. Zhang MM, Zou Y, Li SM, Wang L, Sun YH, Shi L, Lu L, Bao YP, Li SX: The efficacy and safety of omega-3 fatty acids on depressive symptoms in perinatal women: a meta-analysis of randomized placebo-controlled trials. *Transl Psychiatry* 2020, 10(1):193.

577. Zhang X, Lin P, Sun J, Sun Y, Shao D, Cao D, Cao F: Prenatal stress self-help mindfulness intervention via social media: a randomized controlled trial. *J Ment Health* 2021:1-10.

578. Zhao Y, Lin Q, Zhu X, Wang J: Randomized Clinical Trial of a Prenatal Breastfeeding and Mental Health Mixed Management Intervention. *J Hum Lact* 2021, 37(4):761-774.

579. Zhu YP, Wang WJ, Zhang SL, Dai B, Ye DW: Effects of gum chewing on postoperative bowel motility after caesarean section: A meta-analysis of randomised controlled trials. In., vol. 121; 2014.

580. Zlotnick C, Tzilos G, Miller I, Seifer R, Stout R: Randomized controlled trial to prevent postpartum depression in mothers on public assistance. *J Affect Disord* 2016, 189:263-268.

For peer review only

Supplementary File S8. Characteristics of studies by category

A. Distribution by study design

Category	Total no. of studies	Observational & diagnostic studies	Non-randomised interventional studies	Randomised controlled trials	Evidence synthesis studies
Diet & nutrition	143	3 (2%)	4 (3%)	66 (46%)	70 (49%)
Physical activities	132	4 (3%)	11 (8%)	76 (58%)	41 (31%)
Lifestyle adjustments	90	11 (12%)	7 (8%)	48 (53%)	24 (27%)
Complementary & alternative therapies	64	3 (5%)	3 (5%)	32 (50%)	26 (41%)
Psycho-social strategies	97	2 (2%)	15 (15%)	58 (60%)	22 (23%)
Breast & nipple care	22	1 (5%)	4 (18%)	8 (36%)	9 (41%)
Sexual health & family planning	12	0 (0%)	0 (0%)	10 (83%)	2 (17%)
Self-monitoring	69	8 (12%)	8 (12%)	36 (52%)	17 (25%)
Self-management of medication	17	1 (6%)	7 (41%)	6 (35%)	3 (18%)
Self-testing or sampling	18	10 (56%)	2 (11%)	6 (33%)	0 (0%)
Other self-management skills	54	8 (15%)	3 (6%)	23 (43%)	20 (37%)

B. Distribution by time of intervention

Category	Total no. of studies	Postpartum	Intrapartum	Antenatal	Preconception
Diet & nutrition	143	27 (19%)	4 (3%)	123 (86%)	7 (5%)
Physical activities	132	25 (19%)	7 (5%)	110 (83%)	3 (2%)
Lifestyle adjustments	90	10 (11%)	0 (0%)	84 (93%)	3 (3%)
Complementary & alternative therapies	64	22 (34%)	23 (36%)	19 (30%)	0 (0%)
Psycho-social strategies	97	21 (22%)	10 (10%)	72 (74%)	1 (1%)
Breast & nipple care	22	14 (64%)	1 (5%)	7 (32%)	0 (0%)
Sexual health & family planning	12	3 (25%)	0 (0%)	10 (83%)	0 (0%)
Self-monitoring	69	9 (13%)	0 (0%)	63 (91%)	4 (6%)
Self-management of medication	17	3 (18%)	3 (18%)	14 (82%)	1 (6%)
Self-testing or sampling	18	1 (6%)	0 (0%)	18 (100%)	0 (0%)
Other self-management skills	54	11 (20%)	28 (52%)	20 (37%)	1 (2%)

**Some studies feature self-care that can be carried out in more than one pregnancy periods; hence the percentages do not total 100%.*

For peer review only - http://bmjopen.bmj.com/site/about/guidelines.xhtml

C. Distribution by venue of access to intervention

Category	Total no. of studies	Peer/partner support	Home-based care	Community-based healthcare	Digital technologies / platforms	OTC availability	Health facilities
Diet & nutrition	143	3 (2%)	22 (15%)	12 (8%)	25 (17%)	21 (15%)	37 (26%)
Physical activities	132	6 (5%)	8 (6%)	37 (28%)	26 (20%)	6 (5%)	50 (38%)
Lifestyle adjustments	90	4 (4%)	18 (20%)	6 (7%)	17 (19%)	8 (9%)	32 (36%)
Complementary & alternative therapies	64	2 (3%)	11 (17%)	1 (2%)	0 (0%)	25 (39%)	27 (42%)
Psycho-social strategies	97	8 (8%)	10 (10%)	14 (14%)	18 (19%)	1 (1%)	40 (41%)
Breast & nipple care	22	0 (0%)	5 (23%)	0 (0%)	1 (5%)	6 (27%)	7 (32%)
Sexual health & family planning	12	2 (17%)	0 (0%)	2 (17%)	0 (0%)	0 (0%)	8 (67%)
Self-monitoring	69	1 (1%)	5 (7%)	4 (6%)	35 (51%)	0 (0%)	40 (58%)
Self-management of medication	17	3 (18%)	0 (0%)	7 (41%)	1 (6%)	0 (0%)	7 (41%)
Self-testing or sampling	18	1 (6%)	1 (6%)	2 (11%)	0 (0%)	0 (0%)	17 (94%)
Other self-management skills	54	6 (11%)	4 (7%)	2 (4%)	5 (9%)	3 (6%)	24 (44%)

**Some studies feature more than one venues of access to intervention; hence the percentages do not total 100%. Evidence synthesis studies were not assessed as the included studies might have different venues of access or there might be insufficient information to determine venues of access for all included studies.*

D. Distribution by persons providing external support

Category	Total no. of studies	Family & other community members	Community health workers	Healthcare workers	No external support
Diet & nutrition	143	4 (3%)	9 (6%)	38 (27%)	27 (19%)
Physical activities	132	15 (11%)	17 (13%)	58 (44%)	19 (14%)
Lifestyle adjustments	90	1 (1%)	2 (2%)	37 (41%)	26 (29%)
Complementary & alternative therapies	64	3 (5%)	0 (0%)	17 (27%)	19 (30%)
Psycho-social strategies	97	10 (10%)	2 (2%)	44 (45%)	23 (24%)
Breast & nipple care	22	0 (0%)	0 (0%)	3 (14%)	9 (41%)
Sexual health & family planning	12	0 (0%)	2 (17%)	8 (67%)	0 (0%)
Self-monitoring	69	2 (3%)	3 (4%)	30 (43%)	18 (26%)
Self-management of medication	17	0 (0%)	7 (41%)	5 (29%)	2 (12%)
Self-testing or sampling	18	0 (0%)	2 (11%)	7 (39%)	10 (56%)
Other self-management skills	54	1 (2%)	2 (4%)	12 (22%)	20 (37%)

**Some studies feature more than one sources of external support; hence the percentages do not total 100%. Evidence synthesis studies were not assessed as the included studies might have different sources of external support or there might be insufficient information to determine sources of external support for all included studies.*

For peer review only

For peer review only - http://bmjopen.bmj.com/site/about/guidelines.xhtml

Supplementary File S9. Complex interventions and their characteristics (N=35)

A. Self-care activities featured as a component of complex interventions

Self-care activity	No. of studies (%)
**Diet & nutrition**	
Dietary adjustment	19 (54%)
Dietary intake - Iodine	1 (3%)
Supplement intake - Calcium	2 (6%)
Supplement intake - Iron and/or folic acid (IFA)	5 (14%)
Supplement intake - not otherwise specified	2 (6%)
Caffeine intake reduction	1 (3%)
**Physical activity**	
Birthing/exercise balls	3 (9%)
Pelvic floor exercises	1 (3%)
Yoga	2 (6%)
Physical activity (not otherwise specified)	14 (40%)
**Lifestyle adjustments**	
Air pollution avoidance	1 (3%)
Alcohol use reduction	6 (17%)
Avoiding strenuous activities	3 (9%)
Deworming	1 (3%)
Drug use reduction	2 (6%)
Oral hygiene	2 (6%)
Insecticide-treated nets	1 (3%)
Sleep hygiene	4 (11%)
Smoking cessation	5 (14%)
TORCH prevention	1 (3%)
Urogenital hygiene	3 (9%)
Other risk reduction strategies (traffic accident, X-ray exposure)	1 (3%)
General self-care to relieve physiological discomforts (not otherwise specified)	6 (17%)
**Complementary & alternative therapies**	
Acupressure	1 (3%)
Aromatherapy	2 (6%)
General massage	9 (26%)
Shower/bath	2 (6%)
**Psycho-social strategies**	
Interpersonal relationship & support network	10 (29%)
Mind-body relaxation	16 (46%)
Music therapy	4 (11%)
Other emotion management strategies	11 (31%)
**Breast & nipple care**	
Breast/nipple care (not otherwise specified)	3 (9%)
Breastfeeding techniques	12 (34%)
**Sexual health & postpartum family planning**	
Condom use	2 (6%)
HIV disclosure	2 (6%)

For peer review only - http://bmjopen.bmj.com/site/about/guidelines.xhtml

Self-care activity	No. of studies (%)
Intimate partner violence (IPV) prevention	3 (9%)
Safe and healthy sexual life	2 (6%)
Sexual negotiation	1 (3%)
STI prevention (not otherwise specified)	1 (3%)
**Self-monitoring**	
Foetal movement monitoring	2 (6%)
Home-based records	2 (6%)
Recognising signs of labour	6 (17%)
Recognising signs of complications	6 (17%)
Self-monitoring of uterine contraction	1 (3%)
Self-monitoring weight	5 (14%)
Self-monitoring blood glucose	2 (6%)
Self-monitoring blood pressure	1 (3%)
**Self-management of medication**	
Self-medication	2 (6%)
Adherence to PMTCT & ART	1 (3%)
**Other self-management skills**	
Birth plans	7 (20%)
Birth position	5 (14%)
Techniques during childbirth (not otherwise specified)	3 (9%)
Postoperative wound care	1 (3%)

Note: Each of these self-care activities is often a component of a larger intervention, which feature more than 3 different self-care activities.

B. Health issues targeted by complex interventions

Target outcomes	No. of studies (%)
Care-seeking behaviours	1 (3%)
Diabetes mellitus	2 (6%)
Fatigue	1 (3%)
HIV/STDs	2 (6%)
Hypertensive disorders of pregnancy	1 (3%)
Low birth weight	1 (3%)
Nutritional deficiencies	2 (6%)
Physiological discomforts	1 (3%)
Positive childbirth experience	9 (26%)
Postpartum health	1 (3%)
Preconception health	3 (9%)
Preterm birth	1 (3%)
Psychological wellbeing	11 (31%)
Various maternal & neonatal outcomes	3 (9%)
Weight management	3 (9%)

For peer review only - http://bmjopen.bmj.com/site/about/guidelines.xhtml

Supplementary File S10. List of all self-care interventions identified from included studies

Self-care intervention	Description	No. of studies (%, N=545)	Health issues / outcomes targeted by intervention	Periods of intervention*
**Diet & nutrition**		**143 (26%)<br>P: 73<br>E: 70**		
Dietary adjustment	Modifying daily intakes of vegetables, fruit, water, salt, fat, sugar, etc. or total calorie consumption	75 (14%)<br>P: 49 †<br>E: 26	Birth weight, diabetes mellitus, hypertensive disorders of pregnancy, nutritional deficiencies, physiological discomforts, preconception health, preterm birth, psychological wellbeing, weight management	Preconception<br>Antenatal<br>Postpartum
Dietary intake - Calcium	From diet (dairy products and green leafy vegetables)	1 (0.2%)<br>P: 0<br>E: 1	Various maternal & neonatal outcomes	Antenatal<br>Postpartum
Dietary intake - Omega-3 fatty acids	From diet (fish, nuts and seeds, plant oils)	1 (0.2%)<br>P: 1<br>E: 0	Psychological wellbeing	Antenatal
Supplement intake - Calcium		5 (1%)<br>P: 0<br>E: 5	Hypertensive disorders of pregnancy, nutritional deficiencies, preterm birth	Preconception<br>Antenatal<br>Postpartum
Supplement intake - Iodine		2 (0.4%)<br>P: 0<br>E: 2	Various maternal & neonatal outcomes	Preconception<br>Antenatal<br>Postpartum
Supplement intake - Iron and/or folic acid (IFA)	Potential interaction with some medications e.g. malaria chemoprophylaxis<br>Including self-management of weekly IFA supplements (n=2)	12 (2%)<br>P: 5<br>E: 7	Diabetes mellitus, hypertensive disorders of pregnancy, low birth weight, nutritional deficiencies, preconception health, preterm birth	Preconception<br>Antenatal<br>Postpartum
Supplement intake - Lipid-based nutrients	Fortified products which provide energy mainly in the form of lipids, and additionally other nutrients (protein, essential fatty acids, micronutrients)	2 (0.4%)<br>P: 2<br>E: 0	Various maternal & neonatal outcomes	Antenatal
Supplement intake - Multiple-micronutrients	Supplements containing 13-15 micronutrients (vitamins and minerals) in one tablet, including UNIMMAP formulation	9 (2%)<br>P: 4<br>E: 5	Diabetes mellitus, nutritional deficiencies, preterm birth, other maternal & neonatal outcomes	Preconception<br>Antenatal
Supplement intake - Omega-3 fatty acids	Including DHA, EHA, fish oils etc.	11 (2%)<br>P: 3<br>E: 8	Diabetes mellitus, preeclampsia, preterm birth, postpartum depression	Preconception<br>Antenatal<br>Postpartum
Supplement intake - Vitamin A	Only recommended in areas with endemic vitamin A deficiency or night blindness	2 (0.4%)<br>P: 1<br>E: 1	Bacterial vaginosis, malaria, nutritional deficiencies, preterm birth	Antenatal<br>Postpartum
Supplement intake - Vitamin B		1 (0.2%)<br>P: 0<br>E: 1	Various maternal & neonatal outcomes	Antenatal<br>Intrapartum
Supplement intake - Vitamin C		2 (0.4%)<br>P: 0<br>E: 2	Urinary tract infections, other maternal & neonatal outcomes	Antenatal
Supplement intake - Vitamin D		2 (0.4%)<br>P: 0<br>E: 2	Diabetes mellitus, other maternal & neonatal outcomes	Preconception<br>Antenatal
Supplement intake - Vitamin E		1 (0.2%)<br>P: 0<br>E: 1	Various maternal & neonatal outcomes	Antenatal
Supplement intake - Zinc		2 (0.4%)<br>P: 0<br>E: 2	Preterm birth, other maternal & neonatal outcomes	Antenatal
Supplement intake - not otherwise specified		1 (0.2%)<br>P: 0<br>E: 1	Hypertensive disorders of pregnancy	Antenatal
Caffeine intake reduction		1 (0.2%)<br>P: 0<br>E: 1	Various maternal & neonatal outcomes	Antenatal
Energy drinks during childbirth	Drinks containing various nutrients and electrolytes	1 (0.2%)<br>P: 1<br>E: 0	Positive labour outcomes	Intrapartum
Fibre supplement	Psyllium, methycellulose, calcium polycarbophil etc. that function as bulk-forming laxatives	3 (1%)<br>P: 1<br>E: 2	Constipation, haemorrhoids, physiological discomforts	Antenatal

For peer review only - http://bmjopen.bmj.com/site/about/guidelines.xhtml

Self-care intervention	Description	No. of studies (%, N=545)	Health issues / outcomes targeted by intervention	Periods of intervention*
Gum chewing	Sugar-free gum for chewing to induce satiation or bowel movement	5 (1%)<br>P: 3<br>E: 2	Diabetes mellitus, positive childbirth experience, postoperative bowel function	Antenatal<br>Intrapartum<br>Postpartum
Probiotics	Products containing Lactobacillus, Bifidobacterium and/or Enterococcus sp.	19 (3%)<br>P: 8<br>E: 11	Bacterial vaginosis, birth weight, diabetes mellitus, group B streptococcus infection, mastitis, preterm birth, psychological wellbeing, weight management	Preconception<br>Antenatal<br>Postpartum
Other dietary/nutritional products	Fruit juices, herbal products, any other product containing natural ingredients that were not regulated as medicine	13 (2%)<br>P: 3<br>E: 10	Diabetes mellitus, induction of labour, mastitis, physiological discomforts, postpartum healing & pain relief, psychological wellbeing, sleep quality, urinary tract infections	Preconception<br>Antenatal<br>Intrapartum<br>Postpartum
**Physical activity**		**132 (24%)<br>P: 91<br>E: 41**		
Ankle weights	Worn during daily activities as a resistance exercise	1 (0.2%)<br>P: 1<br>E: 0	Weight management	Postpartum
Aquatic exercises	Exercises conduct under water (e.g. in a swimming pool)	4 (1%)<br>P: 3<br>E: 1	Labour pain, lumbopelvic pain, physiological discomforts, psychological wellbeing, sleep quality	Antenatal
Birthing/exercise balls	Exercises using Swiss balls, exercise balls or peanut balls, etc.	7 (1%)<br>P: 5<br>E: 2	Childbirth self-efficiency (CBSE), labour pain, lumbopelvic pain, positive childbirth experience, positive labour outcomes	Antenatal<br>Intrapartum
Pelvic floor exercises	Exercises aimed at strengthening pelvic floor muscles, e.g. Kegel exercises	17 (3%)<br>P: 13<br>E: 4	Diabetes mellitus, lumbopelvic pain, pelvic floor disorders, physiological discomforts, positive childbirth experience, sexual functioning, weight management	Antenatal<br>Intrapartum<br>Postpartum
Yoga	Physical component (yoga poses) only; meditation component was classified as mind-body relaxation	15 (3%)<br>P: 8<br>E: 7	Diabetes mellitus, labour pain, lumbopelvic pain, physiological discomforts, positive childbirth experience, psychological wellbeing, sleep quality	Antenatal<br>Intrapartum<br>Postpartum
Physical activity (not otherwise specified)		99 (18%)<br>P: 67<br>E: 32	Birth weight, cardiometabolic fitness, diabetes mellitus, fatigue, foetal health, hypertensive disorders of pregnancy, lumbopelvic pain, pelvic floor disorders, physiological discomforts, preconception health, preeclampsia, preterm birth, psychological wellbeing, sleep quality, weight management	Preconception<br>Antenatal<br>Postpartum
**Lifestyle adjustments**		**90 (17%)<br>P: 66<br>E: 24**		
Air pollution avoidance	Avoiding going outdoor when air pollution levels are high	3 (1%)<br>P: 2<br>E: 1	Air pollution exposure, stillbirth	Antenatal
Alcohol use reduction	Self-initiated abstinence or relapse prevention without pharmacological treatment	7 (1%)<br>P: 3<br>E: 4	Alcohol consumption	Preconception<br>Antenatal<br>Postpartum
Avoiding strenuous activities	Sleeping with pillows, ergonomic postures in daily activities (lifting, bending), etc.	2 (0.4%)<br>P: 2<br>E: 0	Lumbopelvic pain	Antenatal
Bed rest during pregnancy	Ranging from selected activity restriction to strict bed rest	1 (0.2%)<br>P: 0<br>E: 1	Various maternal & neonatal outcomes	Antenatal
Clean cookstoves	Cookstoves using cleaner energy sources (electricity, ethanol or biogas) instead of open fire, kerosene or biomass	5 (1%)<br>P: 5<br>E: 0	Hypertensive disorders of pregnancy, low birth weight, respiratory irritation	Antenatal
General self-care to relieve physiological discomforts (not otherwise specified)		3 (1%)<br>P: 3<br>E: 0	Psychological wellbeing	Antenatal<br>Postpartum
Hand hygiene	Washing hands, avoiding exposure to saliva from young children (sharing food and utensils, kissing)	5 (1%)<br>P: 4<br>E: 1	Cytomegalovirus infection	Preconception<br>Antenatal
Oral hygiene	Tooth brushing, flossing, using oral rinses, etc.	6 (1%)<br>P: 5<br>E: 1	Periodontal diseases	Antenatal

For peer review only - http://bmjopen.bmj.com/site/about/guidelines.xhtml

Self-care intervention	Description	No. of studies (%, N=545)	Health issues / outcomes targeted by intervention	Periods of intervention*
Mosquito-repellent strategies - Insecticide-treated nets	Bed nets impregnated with insecticides (e.g. pyrroles, pyrethroids)	12 (2%)<br>P: 11<br>E: 1	Low birth weight, malaria, preterm birth	Antenatal
Mosquito-repellent strategies - Other	Use of insecticides, mosquito coils, fans, insect-repellent cream, wearing long sleeves, burning local plants, etc.	1 (0.2%)<br>P: 1<br>E: 0	Malaria	Antenatal
Second-hand smoke exposure prevention	Establishing a smoke-free home/office, communicating with smokers	3 (1%)<br>P: 3<br>E: 0	Second-hand smoke exposure	Antenatal
Sleep hygiene	Sleeping schedules, good sleep habits, improving sleeping environment	4 (1%)<br>P: 3<br>E: 1	Fatigue, sleep quality	Antenatal<br>Postpartum
Sleeping position	Choosing to left lateral recumbent sleeping position / avoiding supine position, raising the head to relieve heartburn	6 (1%)<br>P: 2<br>E: 4	Hypertensive disorders of pregnancy, low birth weight, physiological discomforts, stillbirth	Antenatal
Smoking cessation	Self-initiated cessation or relapse prevention without pharmacological treatment	32 (6%)<br>P: 22<br>E: 10	Low birth weight, neonatal death, smoking status, stillbirth, other maternal & neonatal outcomes	Antenatal<br>Postpartum
Urogenital hygiene	Hygiene behaviours (daily cleaning, using clean water, using pads)	2 (0.4%)<br>P: 1<br>E: 1	Sexual functioning, urinary tract infections	Antenatal
**Complementary & alternative therapies**		**64 (12%)**<br>**P: 38**<br>**E: 26**		
Acupressure	Applying pressure on specific acupoints. Only manoeuvres that can be administered by the woman herself or her partner are included; reflexology by HCWs is excluded.	13 (2%)<br>P: 6<br>E: 7	Breast engorgement, labour pain, mastitis, perineal healing & pain relief, physiological discomforts, positive labour outcomes, postpartum healing & pain relief	Antenatal<br>Intrapartum<br>Postpartum
Aromatherapy	Including the use of essential oils	7 (1%)<br>P: 2<br>E: 5	Labour pain, perineal healing & pain relief, psychological wellbeing, sleep quality	Intrapartum<br>Postpartum
Bright light therapy	Exposure to natural light (getting outdoor) or artificial light (using a light therapy lamp)	3 (1%)<br>P: 1<br>E: 2	Psychological wellbeing	Antenatal<br>Postpartum
Compress - cold	Ice packs, cryotherapy	9 (2%)<br>P: 5<br>E: 4	Labour pain, perineal healing & pain relief	Intrapartum<br>Postpartum
Compress - hot	Hot water packs, heat therapy	3 (1%)<br>P: 2<br>E: 1	Labour pain, perineal healing & pain relief	Intrapartum<br>Postpartum
Compression stockings	Stockings designed to promote circulation in the legs via gentle compression	1 (0.2%)<br>P: 0<br>E: 1	Physiological discomforts	Antenatal
Continuous positive airway pressure (CPAP)	Delivering positive airway pressure to keep airway open during sleep. CPAP machines are available over-the-counter and do not require specialised skills to operate.	1 (0.2%)<br>P: 1<br>E: 0	Diabetes mellitus, obstructive sleep apnoea	Antenatal
General massage	Any form of body massage	17 (3%)<br>P: 7<br>E: 10	Labour pain, lumbopelvic pain, physiological discomforts, positive childbirth experience, postpartum healing & pain relief, psychological wellbeing, sleep quality, striae gravidarum	Antenatal<br>Intrapartum<br>Postpartum
Kinesiologic taping	Adhesive tape applied to the specific region of the back to provide support	1 (0.2%)<br>P: 1<br>E: 0	Labour pain	Intrapartum
Pelvic support devices	Any device worn at the lower abdominal level to support the pelvic or lumbosacral region e.g. pelvic belts/girdles, abdominal binders, elastomeric fabric orthoses etc.	5 (1%)<br>P: 4<br>E: 1	C-section wound healing, lumbopelvic pain, postpartum healing & pain relief	Antenatal<br>Postpartum
Perineal massage	Can be administered by either the woman herself or the partner in the antenatal or postpartum period	4 (1%)<br>P: 1<br>E: 3	Positive childbirth experience, postpartum healing & pain relief, prevention of perineal injury	Antenatal<br>Intrapartum
Shower/bath	Including birthing pools, shower before labour, foot baths and Sitz baths	15 (3%)<br>P: 7<br>E: 8	Duration of labour, haemorrhoids, labour pain, lumbopelvic pain, perineal healing & pain relief, physiological discomforts, positive	Antenatal<br>Intrapartum<br>Postpartum

For peer review only - http://bmjopen.bmj.com/site/about/guidelines.xhtml

Self-care intervention	Description	No. of studies (%, N=545)	Health issues / outcomes targeted by intervention	Periods of intervention*
			childbirth experience, positive labour outcomes	
Topical products	Ointments, creams, etc. that do not contain medicinal ingredients and are available over-the-counter	5 (1%)<br>P: 3<br>E: 2	Haemorrhoids, postpartum healing & pain relief, striae gravidarum	Antenatal<br>Postpartum
Transcutaneous electrical nerve stimulation (TENS)	Delivering small electrical impulses through electrodes placed on the skin. TENS machines are available over-the-counter and do not require specialised skills to operate.	9 (2%)<br>P: 4<br>E: 5	Labour pain, lumbopelvic pain, postpartum healing & pain relief	Antenatal<br>Intrapartum<br>Postpartum
**Psycho-social strategies**		**97 (18%)<br>P: 75<br>E: 22**		
Interpersonal relationship & support network	Establishing social support, relationship management, communication with others	23 (4%)<br>P: 20<br>E: 3	Diabetes mellitus, intimate partner violence (IPV), psychological wellbeing	Preconception<br>Antenatal<br>Postpartum
Mind-body relaxation	Breathing exercises, guided imagery, progressive muscle relaxation, self-hypnosis, mindfulness (in meditation exercises or daily practices)	63 (12%)<br>P: 45<br>E: 18	Breast & nipple pain, diabetes mellitus, hypertensive disorders of pregnancy, labour pain, lumbopelvic pain, physiological discomforts, positive childbirth experience, positive labour outcomes, postpartum healing & pain relief, psychological wellbeing, sleep quality	Antenatal<br>Intrapartum<br>Postpartum
Music therapy	Listening to music (lullabies, classical music, nature round), either standalone or combined with other techniques (meditation, breathing)	11 (2%)<br>P: 7<br>E: 4	Labour pain, positive childbirth experience, postpartum healing & pain relief, psychological wellbeing, sleep quality	Antenatal<br>Intrapartum<br>Postpartum
Other emotion management strategies	Cognitive restructuring, problem-solving, mood-tracking, etc.	32 (6%)<br>P: 30<br>E: 2	Diabetes mellitus, hypertensive disorders of pregnancy, positive childbirth experience, psychological wellbeing	Antenatal<br>Postpartum
**Breast & nipple care**		**22 (4%)<br>P: 13<br>E: 9**		
Breast compress (hot & cold)	Warm compress (warm towels, heat packs) to promote milk flow before breastfeeding; cold compress (cold gel packs, cold cabbage leaf) to stop/reduce milk flow in case of engorgement	2 (0.4%)<br>P: 1<br>E: 1	Breast engorgement	Postpartum
Breast milk expression	Can be conducted prenatally or postnatally (for colostrum extraction, a few hours after birth), using manual extraction, breast pumps or via breastfeeding	5 (1%)<br>P: 3<br>E: 2	Breast engorgement, various neonatal outcomes	Antenatal<br>Postpartum
Breast massage	Massaging techniques to promote blood circulation and lymphatic drainage	5 (1%)<br>P: 1<br>E: 4	Breast & nipple pain, breast engorgement, mastitis	Postpartum
Breast/nipple shells	Plastic parts worn over the breast to protect the nipples	2 (0.4%)<br>P: 1<br>E: 1	Breast & nipple pain	Postpartum
Hydrogel dressings	Adhesive hydrogel discs or pads applied over breast or nipple to form a protective cushion, support healing and provide cooling effect	2 (0.4%)<br>P: 1<br>E: 1	Breast & nipple pain	Postpartum
Lanolin ointment	Applied over nipple to form a protective layer and promote healing	5 (1%)<br>P: 4<br>E: 1	Breast & nipple pain	Postpartum
Breast/nipple care (not otherwise specified)		1 (0.2%)<br>P: 1<br>E: 0	Breast & nipple pain	Postpartum
Antenatal education on breastfeeding techniques	Any technique for effective breastfeeding (e.g. timing, holding the newborn, latching) taught in the antenatal period	6 (1%)<br>P: 4<br>E: 2	Breast & nipple pain, breast engorgement, psychological wellbeing	Antenatal<br>Postpartum
**Sexual health & postpartum family planning**		**12 (2%)<br>P: 10<br>E: 2**		
Condom use	For STI prevention during pregnancy	2 (0.4%)<br>P: 2<br>E: 0	HIV/STI	Antenatal

For peer review only - http://bmjopen.bmj.com/site/about/guidelines.xhtml

Self-care intervention	Description	No. of studies (%, N=545)	Health issues / outcomes targeted by intervention	Periods of intervention*
HIV disclosure	Deciding when and how to disclose HIV status to family and partner	1 (0.2%)<br>P: 1<br>E: 0	HIV	Antenatal
Intimate partner violence (IPV) prevention	Safety planning, managing conflicts with partner	6 (1%)<br>P: 4<br>E: 2	IPV, psychological wellbeing, sexual functioning	Antenatal<br>Postpartum
Safe and healthy sexual life	Safe sex positions, sexual restrictions during pregnancy, restarting sex after pregnancy, coping with changing sexuality	5 (1%)<br>P: 5<br>E: 0	Sexual functioning	Antenatal<br>Postpartum
Sexual negotiation	Sexual disclosure to partners, refusing risky/unprotected sex	2 (0.4%)<br>P: 1<br>E: 1	HIV/STI, IPV	Antenatal
STI prevention (not otherwise specified)		3 (1%)<br>P: 3<br>E: 0	HIV/STI, sexual functioning	Antenatal
**Self-monitoring**		**69 (13%)<br>P: 52<br>E: 17**		
Foetal heartbeat self-monitoring	Using a home cardiotocography device	1 (0.2%)<br>P: 1<br>E: 0	Detection of abnormal foetal heart rate	Antenatal
Foetal movement monitoring	Counting or observing foetal movements	7 (1%)<br>P: 4<br>E: 3	Perinatal mortality, psychological wellbeing	Antenatal
Home-based records	Home-based records to keep track of multiple health indicators, such as weight, mood, diet, physical activity, medication use, vaccination records, etc.	4 (1%)<br>P: 2<br>E: 2	Diabetes mellitus, health service utilisation, psychological wellbeing, other maternal & neonatal outcomes	Preconception<br>Antenatal<br>Postpartum
Recognising signs of labour	Differentiate false vs true labour, recognising when to notify health providers	2 (0.4%)<br>P: 0<br>E: 2	Positive childbirth experience	Antenatal
Self-assessment of foetal presentation	Self-palpation to detect spontaneous reversion to breech presentation	1 (0.2%)<br>P: 1<br>E: 0	Breech presentation	Antenatal
Self-measurement of symphysis-fundus height	Measuring distance from the symphysis pubis to the uterine fundus with a tape measure	1 (0.2%)<br>P: 1<br>E: 0	Intrauterine growth restriction	Antenatal
Self-monitoring lung function	Using handheld respiratory devices	1 (0.2%)<br>P: 1<br>E: 0	Asthma control	Antenatal
Self-monitoring of ketonuria	Using urine dipsticks	1 (0.2%)<br>P: 1<br>E: 0	Diabetes mellitus	Antenatal
Self-monitoring of uterine contraction	Self-palpation and counting contractions	1 (0.2%)<br>P: 1<br>E: 0	Uterine contraction awareness	Antenatal
Self-monitoring weight	Often accompanying lifestyle interventions for weight loss	17 (3%)<br>P: 16<br>E: 1	Preconception health, weight management, other maternal & neonatal outcomes	Preconception<br>Antenatal<br>Postpartum
Self-monitoring blood glucose (Continuous glucose monitoring or CGM)	Using a CGM device that measures interstitial blood glucose levels. Only interventions where CGM data is accessible for pregnant women for lifestyle adjustments or insulin dose self-titration were included.	8 (1%)<br>P: 6<br>E: 2	Diabetes mellitus	Preconception<br>Antenatal
Self-monitoring blood glucose (Flash glucose monitoring or FGM)	Similar to CGM but only displaying glucose results when activated via phone or device	1 (0.2%)<br>P: 1<br>E: 0	Diabetes mellitus	Antenatal
Self-monitoring blood glucose (not otherwise specified)	Any interventions involving SMBG using glucometers, CGM or FGM devices	16 (3%)<br>P: 9<br>E: 7	Diabetes mellitus, other maternal & neonatal outcomes	Preconception<br>Antenatal<br>Postpartum
Self-monitoring blood pressure	Blood pressure monitors are available over-the-counter	12 (2%)<br>P: 10<br>E: 2	Diabetes mellitus, hypertensive disorders of pregnancy, postpartum hypertension	Antenatal<br>Postpartum

For peer review only - http://bmjopen.bmj.com/site/about/guidelines.xhtml

Self-care intervention	Description	No. of studies (%, N=545)	Health issues / outcomes targeted by intervention	Periods of intervention*
**Self-management of medication**		**17 (3%)<br>P: 14<br>E: 3**		
Self-adjustment of antihypertensive medication	Guided by a blood pressure-based algorithm	1 (0.2%)<br>P: 1<br>E: 0	Postpartum hypertension	Postpartum
Self-administration of antibiotics	Distributed during antenatal visits to be administered by the woman after birth	1 (0.2%)<br>P: 1<br>E: 0	Postpartum infection	Antenatal<br>Intrapartum
Self-administration of misoprostol	Distributed during antenatal visits to be administered by the woman after birth	10 (2%)<br>P: 8<br>E: 2	Postpartum haemorrhage	Antenatal<br>Intrapartum
Self-administration of malaria intermittent preventive treatment for pregnancy (IPTp)	All doses distributed to be administered at home, without direct observation	2 (0.4%)<br>P: 2<br>E: 0	Low birth weight, malaria	Antenatal
Self-administration of HIV oral pre-exposure prophylaxis (PrEP)	Daily oral tenofovir disoproxil fumarate/emtricitabine (TDF/FTC) for women with high risk of HIV acquisition	4 (1%)<br>P: 3<br>E: 1	HIV prevention	Preconception<br>Antenatal<br>Postpartum
**Self-testing or sampling**		**18 (3%)<br>P: 18<br>E: 0**		
pH-sensitive panty liner	To identify vaginal discharge of abnormal pH	1 (0.2%)<br>P: 1<br>E: 0	Amniotic fluid leakage	Antenatal
Self-collection of nasal samples	Self-collecting samples to be sent to a central laboratory for testing, as opposed to clinic-based collection by health providers	2 (0.4%)<br>P: 2<br>E: 0	Viral upper respiratory tract infections	Antenatal
Self-collection of urine/vaginal samples	Self-collecting samples at clinic visits, as opposed to collection by health providers	5 (1%)<br>P: 5<br>E: 0	Bacterial vaginosis, chlamydia, human papillovirus	Antenatal
Self-testing for HIV	For couple self-testing, studies only providing testing rates by the partner were excluded.	5 (1%)<br>P: 5<br>E: 0	HIV	Antenatal<br>Postpartum
Self-testing for malaria	Using rapid diagnostic test kits	1 (0.2%)<br>P: 1<br>E: 0	Malaria	Antenatal
Self-testing for proteinuria	Using urinalysis dipstick-based test kits	2 (0.4%)<br>P: 2<br>E: 0	Hypertensive disorders of pregnancy, proteinuria	Antenatal
Self-testing of vaginal pH	Using pH-sensitive dipsticks	2 (0.4%)<br>P: 2<br>E: 0	Bacterial vaginosis, preterm birth	Antenatal
**Other self-management skills**		**54 (10%)<br>P: 34<br>E: 20**		
Birth plans	Written plans to communicate preferences and goals during labour and childbirth	2 (0.4%)<br>P: 2<br>E: 0	Positive labour outcomes, psychological wellbeing	Antenatal<br>Intrapartum
Birth position	Choosing upright versus recumbent position during childbirth	12 (2%)<br>P: 6<br>E: 6	Duration of labour, positive labour outcomes	Intrapartum
Techniques during childbirth (not otherwise specified)	Correct pushing techniques and timing, walking and changing positions frequently, emptying bladder	3 (1%)<br>P: 2<br>E: 1	Lumbopelvic pain, positive childbirth experience	Antenatal<br>Intrapartum
Estimation of blood loss during birth by family members	Either by visual estimation, gravimetric methods or using a calibrated drape/collecting pouch. Can be administered by a family member if birth attendants are not available	6 (1%)<br>P: 5<br>E: 1	Postpartum haemorrhage	Intrapartum<br>Postpartum
First-aid for complications by family members - Naturally-induced oxytocin release	To increase uterine tone in postpartum haemorrhage. Achieved via breast, nipple or uterine stimulation. Can be self-administered or administered by partner if birth attendants are not available	6 (1%)<br>P: 3<br>E: 3	Postpartum haemorrhage	Postpartum

For peer review only - http://bmjopen.bmj.com/site/about/guidelines.xhtml

Self-care intervention	Description	No. of studies (%, N=545)	Health issues / outcomes targeted by intervention	Periods of intervention*
First-aid for complications by family members (not otherwise specified)	Basic first aid training for major postpartum complications (postpartum haemorrhage, postpartum infection, seizures, etc.)	1 (0.2%)<br>P: 1<br>E: 0	Various maternal & neonatal outcomes	Antenatal
Labour induction - Castor oil	Stimulates cervical ripening via PGE2 pathway	3 (1%)<br>P: 2<br>E: 1	Duration of labour	Intrapartum
Labour induction - Naturally-induced oxytocin release	Via breast or nipple stimulation, uterine stimulation or coitus	4 (1%)<br>P: 3<br>E: 1	Duration of labour	Antenatal<br>Intrapartum
Making medical decisions	Making decisions for oneself or the foetus/newborn e.g. prenatal screening, mode of delivery, etc.	12 (2%)<br>P: 7<br>E: 5	Decisional conflict, positive childbirth experience	Preconception<br>Antenatal
Postoperative wound care	Cleaning the wound area, identifying signs of infection. Applicable to C-section/episiotomy wounds and perineal injuries	2 (0.4%)<br>P: 1<br>E: 1	C-section wound healing, postpartum healing & pain relief	Antenatal<br>Postpartum
Self-management of asthma	Self-managing symptoms and medications, personalised action plan for attack episodes	3 (1%)<br>P: 2<br>E: 1	Asthma control	Antenatal

* If an intervention involves providing training or educating pregnant women in the antenatal period for self-care skills to be used in the postpartum period, the period of intervention will be classified as both “Antenatal” and “Postpartum”.

[†] P: number of primary studies (interventional, observational and diagnostic accuracy studies); E: number of evidence synthesis studies (systematic reviews, meta-analyses and scoping reviews). Some primary studies (n=45, 8%) are also included in evidence synthesis studies featured in this review.

For peer review only

Supplementary File S11. Categorising self-care interventions based on WHO's classification framework

Category	N	%
**1.1 Individual agency**		
Interventions to promote awareness about self-care	0	0%
Interventions to promote autonomy and/or confidence to engage in self-care *<br>Interventions for improving self-care capacities and capabilities *	325	56%
Interventions for improving health and digital literacy	0	0%
Interventions to promote sustained adoption of self-care practices and behaviours	0	0%
**1.2 Health information seeking**		
Acquiring health education for informed health decision-making *<br>Improving health literacy to support health decision-making *	22	4%
Individuals seeking health information via on-demand information services	0	0%
Individuals health decision-making support via online or mobile tools	46	8%
**1.3 Social and community support**		
Peer mentorship and counselling	1	0.2%
Peer-to-peer action to support access to and the uptake and use of self-care interventions	42	7%
Individuals sharing health data with peers	3	1%
**1.4 Personal health tracking**		
Home-based record for health and diagnostic data	39	7%
Self-monitoring of health	70	12%
Active data capture/documentation by self-care user	32	6%
Passive data capture/documentation by device	9	2%
Use of diagnostic device at community location for self-measuring health indicator	0	0%
Tracking rational and responsible self-use of medicines and therapeutics	8	1%
**1.5 Self-diagnosis of health conditions**		
Self-testing, including to inform prevention	11	2%
Self-examining for health conditions	1	0%
Self-collection of samples to send for external testing	7	1%
Using online symptom checkers and health information	0	0%
**1.6 Self-management of health**		
Self-care for long-term health conditions	86	15%
Self-care for short-term health conditions	189	33%
Self-medication/treatment without medical prescription including for side effects	16	3%
Self-regulation of health conditions	148	26%
Self-care prevention including risk avoidance and support for physical and mental health and well-being	296	51%
Health management performed by caregivers	16	3%
Individuals procurement of safe over-the-counter medicines and products for self-care	106	18%

For peer review only - http://bmjopen.bmj.com/site/about/guidelines.xhtml

Category	N	%
**1.7 Individuals linkage to health system**		
Individuals-initiated communication with health system	5	1%
Individuals sharing health data with health workers	38	7%
Individuals identifying location of health facilities/structures	0	0%
Individuals receiving feedback from health workers	60	10%
**1.8 Individuals financial transactions for health** ***-not within the scope of inclusion of this review-***		
Expenses for over-the-counter health medicines and products that do not require prescription	0	0%
Availability or management of vouchers to individuals(s) for health services	0	0%
Expenses for prescription medicines	0	0%
Access to health insurance and/or adequate health insurance coverage	0	0%
Expenses for indirect health costs	0	0%

* These categories were considered to be equivalent during classifying, as there was insufficient information on how to differentiate between the two categories.

Discussion

The categories was based on WHO's guideline on classification of self-care interventions[1], which was published in 2021 as an initiative to standardise the language used in researching and reporting about self-care interventions. The framework describes four broad types of self-care interventions (interventions by self-carers and caregivers, by health workers, by health programme managers and by health policymakers/ legislators/regulators). For our review, we used the categories under self-care interventions for self-carers and caregivers. In classifying each study, we examined the description of its intervention in both the article and the associated protocol (if available).

As no standardised definition was provided in WHO's framework, liberty was taken in interpreting the meaning of these self-care categories when classifying our studies. For example, for interventions under category "Individual agency", we assume that these refer to interventions to raise public awareness or health/digital literacy about self-care, such as a public campaign or education programme. Therefore, digital platforms or education workshops targeting specific health issues (e.g. diabetes management or weight maintenance) is not classified under these categories.

Percentages were calculated based on the total number of studies (n=580). The percentages in the table above do not add up to 100% in some instances because some interventions can match to multiple WHO categories. For example, an intervention to increase physical activities involving a support group and a mobile app will be classified under "Self-regulation of health conditions" and "Peer-to-peer action to support access to and the uptake and use of self-care interventions".

There are several likely reasons for an absence of studies (0%) under some categories.

(a) For category 1.8 - Individuals financial transactions for health, the focus is on economic interventions, which is determined *a priori* as outside the scope of our review.

For peer review only - http://bmjopen.bmj.com/site/about/guidelines.xhtml

(b) For other categories, it can be interpreted as our review did not find any corresponding evidence of effectiveness or safety in the literature. Nonetheless, it is possible that qualitative or economic evidence still exists for these categories.

Reference

1 World Health Organization. Classification of self-care interventions for health: a shared language to describe the uses of self-care interventions. Geneva, 2021 https://www.who.int/publications/i/item/9789240039469 (accessed April 6, 2022).

For peer review only

Supplementary File S10: Preferred Reporting Items for Systematic reviews and Meta-Analyses extension for Scoping Reviews (PRISMA-ScR) Checklist

SECTION	ITEM	PRISMA-ScR CHECKLIST ITEM	REPORTED ON PAGE #
**TITLE**			
Title	1	Identify the report as a scoping review.	1
**ABSTRACT**			
Structured summary	2	Provide a structured summary that includes (as applicable): background, objectives, eligibility criteria, sources of evidence, charting methods, results, and conclusions that relate to the review questions and objectives.	2-3
**INTRODUCTION**			
Rationale	3	Describe the rationale for the review in the context of what is already known. Explain why the review questions/objectives lend themselves to a scoping review approach.	4
Objectives	4	Provide an explicit statement of the questions and objectives being addressed with reference to their key elements (e.g., population or participants, concepts, and context) or other relevant key elements used to conceptualize the review questions and/or objectives.	4
**METHODS**			
Protocol and registration	5	Indicate whether a review protocol exists; state if and where it can be accessed (e.g., a Web address); and if available, provide registration information, including the registration number.	5<br>Supp File S1
Eligibility criteria	6	Specify characteristics of the sources of evidence used as eligibility criteria (e.g., years considered, language, and publication status), and provide a rationale.	5-7<br>Supp File S3
Information sources*	7	Describe all information sources in the search (e.g., databases with dates of coverage and contact with authors to identify additional sources), as well as the date the most recent search was executed.	5<br>Supp File S2
Search	8	Present the full electronic search strategy for at least 1 database, including any limits used, such that it could be repeated.	Supp File S2
Selection of sources of evidence†	9	State the process for selecting sources of evidence (i.e., screening and eligibility) included in the scoping review.	6-7
Data charting process‡	10	Describe the methods of charting data from the included sources of evidence (e.g., calibrated forms or forms that have been tested by the team before their use, and whether data charting was done independently or in duplicate) and any processes for obtaining and confirming data from investigators.	6-7<br>Supp File S4
Data items	11	List and define all variables for which data were sought and any assumptions and simplifications made.	7<br>Supp File S4
Critical appraisal of individual sources of evidence§	12	If done, provide a rationale for conducting a critical appraisal of included sources of evidence; describe the methods used and how this information was used in any data synthesis (if appropriate).	N/A
Synthesis of results	13	Describe the methods of handling and summarizing the data that were charted.	7
**RESULTS**			
Selection of sources of evidence	14	Give numbers of sources of evidence screened, assessed for eligibility, and included in the review, with reasons for exclusions at each stage, ideally using a flow diagram.	8<br>Fig 1
Characteristics of sources of evidence	15	For each source of evidence, present characteristics for which data were charted and provide the citations.	N/A
Critical appraisal within sources of evidence	16	If done, present data on critical appraisal of included sources of evidence (see item 12).	N/A
Results of individual sources of evidence	17	For each included source of evidence, present the relevant data that were charted that relate to the review questions and objectives.	N/A

For peer review only

SECTION	ITEM	PRISMA-ScR CHECKLIST ITEM	REPORTED ON PAGE #
Synthesis of results	18	Summarize and/or present the charting results as they relate to the review questions and objectives.	8-12
**DISCUSSION**			
Summary of evidence	19	Summarize the main results (including an overview of concepts, themes, and types of evidence available), link to the review questions and objectives, and consider the relevance to key groups.	13-4
Limitations	20	Discuss the limitations of the scoping review process.	14-15
Conclusions	21	Provide a general interpretation of the results with respect to the review questions and objectives, as well as potential implications and/or next steps.	15
**FUNDING**			
Funding	22	Describe sources of funding for the included sources of evidence, as well as sources of funding for the scoping review. Describe the role of the funders of the scoping review.	16

From: Tricco AC, Lillie E, Zarin W, O'Brien KK, Colquhoun H, Levac D, et al. PRISMA Extension for Scoping Reviews (PRISMAScR): Checklist and Explanation. Ann Intern Med. 2018;169:467–473. doi: 10.7326/M18-0850.

For peer review only

BMJ Open

Self-care interventions for preconception, antenatal, intrapartum and postpartum care: a scoping review

Journal:	*BMJ Open*
Manuscript ID	bmjopen-2022-068713.R2
Article Type:	Original research
Date Submitted by the Author:	01-Apr-2023

Complete List of Authors:	Nguyen, Phi-Yen; Monash University, Methods in Evidence Synthesis Unit; Burnet Institute, International Development<br>Caddy, Cassandra; Burnet Institute, International Development<br>Wilson, Alyce; Burnet Institute, International Development<br>Blackburn, Kara; Burnet Institute, International Development<br>Page, Matthew; Monash University, School of Public Health and Preventive Medicine<br>Gülmezoglu, Ahmet Metin ; Concept Foundation<br>Narasimhan, Manjulaa; World Health Organization, Department of Reproductive Health and Research<br>Bonet, Mercedes; World Health Organization, Department of Reproductive Health and Research<br>Tunçalp, Özge; World Health Organization, Department of Reproductive Health and Research<br>Vogel, Joshua; Burnet Institute, International Development

**Primary Subject Heading**:	Sexual health
Secondary Subject Heading:	Evidence based practice, Global health, Public health, Reproductive medicine
Keywords:	Antenatal < GENETICS, PUBLIC HEALTH, SEXUAL MEDICINE, REPRODUCTIVE MEDICINE, Maternal medicine < OBSTETRICS, Health policy < HEALTH SERVICES ADMINISTRATION & MANAGEMENT

SCHOLARONE™
Manuscripts

I, the Submitting Author has the right to grant and does grant on behalf of all authors of the Work (as defined in the below author licence), an exclusive licence and/or a non-exclusive licence for contributions from authors who are: i) UK Crown employees; ii) where BMJ has agreed a CC-BY licence shall apply, and/or iii) in accordance with the terms applicable for US Federal Government officers or employees acting as part of their official duties; on a worldwide, perpetual, irrevocable, royalty-free basis to BMJ Publishing Group Ltd ("BMJ") its licensees and where the relevant Journal is co-owned by BMJ to the co-owners of the Journal, to publish the Work in this journal and any other BMJ products and to exploit all rights, as set out in our licence.

The Submitting Author accepts and understands that any supply made under these terms is made by BMJ to the Submitting Author unless you are acting as an employee on behalf of your employer or a postgraduate student of an affiliated institution which is paying any applicable article publishing charge ("APC") for Open Access articles. Where the Submitting Author wishes to make the Work available on an Open Access basis (and intends to pay the relevant APC), the terms of reuse of such Open Access shall be governed by a Creative Commons licence – details of these licences and which Creative Commons licence will apply to this Work are set out in our licence referred to above.

Other than as permitted in any relevant BMJ Author's Self Archiving Policies, I confirm this Work has not been accepted for publication elsewhere, is not being considered for publication elsewhere and does not duplicate material already published. I confirm all authors consent to publication of this Work and authorise the granting of this licence.

For peer review only

Self-care interventions for preconception, antenatal, intrapartum and postpartum care: a scoping review

Phi-Yen Nguyen[1,2], Cassandra Caddy[1], Alyce N. Wilson[1], Kara Blackburn[1], Matthew J. Page[2], A. Metin Gülmezoglu[3], Manjulaa Narasimhan[4], Mercedes Bonet [4], Özge Tunçalp[4], Joshua P. Vogel[1] *

1. Maternal, Child and Adolescent Health Program, Burnet Institute, Melbourne, Australia

2. Methods in Evidence Synthesis Unit, School of Public Health and Preventive Medicine, Monash University, Melbourne, Australia

3. Concept Foundation, Geneva, Switzerland

4. Department of Sexual and Reproductive Health and Research, including UNDP/UNFPA/UNICEF/WHO/World Bank Special Programme of Research, Development and Research Training in Human Reproduction (HRP), World Health Organization, Geneva, Switzerland

***Correspondence to:** A/P Joshua P. Vogel, Global Women's and Newborn's Health Group, Burnet Institute, 85 Commercial Rd, Melbourne, Victoria, Australia 3004.

Email address: joshua.vogel@burnet.edu.au

Total word count: 3,804 words (excluding tables and figures)

Keywords: self-care, pregnancy, preconception, antenatal, intrapartum, postpartum, postnatal, maternal outcomes, neonatal outcomes, infants, newborns

For peer review only - http://bmjopen.bmj.com/site/about/guidelines.xhtml

ABSTRACT (300 words)

Objective: To identify current and emerging self-care interventions to improve maternity health care

Design: Scoping review

Data sources: Medline, Embase, Emcare, PsycINFO, Cochrane CENTRAL/CDSR, CINAHL Plus (last searched on October 17th, 2021)

Eligibility criteria: Evidence syntheses, interventional or observational studies describing any tool, resource or strategy to facilitate self-care in women preparing to get pregnant, currently pregnant, giving birth, or postpartum.

Data extraction/synthesis: Screening and data collection were conducted independently by two reviewers. Self-care interventions were identified based on pre-defined criteria and inductively organised into 11 categories. Characteristics of study design, interventions, participants and outcomes were recorded.

Results: We identified eligible 580 studies. Many included studies evaluated interventions in high-income countries (45%) and during antenatal care (76%). Self-care categories featuring highest numbers of studies were diet and nutrition (26% of all studies), physical activity (24%), psycho-social strategies (18%) and other lifestyle adjustments (17%). Few studies featured self-care interventions for sexual health and postpartum family planning (2%), self-management of medication (3%), and self-testing/sampling (3%). Several venues to introduce self-care were described: health facilities (44%), community venues (14%), digital platforms (18%), partner/peer support (7%), or over-the-counter products (13%). Involvement of health and community workers were described in 38% and 8% of studies, who supported self-care interventions by providing therapeutics for home use, training or counselling. The most common categories of outcomes evaluated were neonatal outcomes (e.g. birth weight) (31%), maternal mental health (26%), and labour outcomes (e.g. duration of labour) (22%).

For peer review only - http://bmjopen.bmj.com/site/about/guidelines.xhtml

Conclusion: Self-care interventions in maternal care are diverse in their applications, implementation characteristics and intended outcomes. Many self-care interventions were implemented with support from the health system at initial stages of use and uptake. Some promising self-care interventions require further primary research, though several are matured and up-to-date evidence syntheses are needed. Research on self-care in the pre-conception period is lacking.

Strengths

- Mapping all potential self-care practices across the spectrum of pregnancy care, from preconception, antenatal, intrapartum to postnatal care
- Use of the new WHO self-care classification system to organise and categorise findings

Limitations

- Not screening for primary studies reported within an included evidence synthesis
- Not including interventions that took place after 42 days postpartum, or those that related primarily to care of newborns and breastfeeding (which should require dedicated reviews)
- Search limited to published reports and reports written in English only

For peer review only

For peer review only - http://bmjopen.bmj.com/site/about/guidelines.xhtml

BACKGROUND

Self-care is defined by the World Health Organization (WHO) as "the ability of individuals, families and communities to promote health, prevent disease, maintain health, and to cope with illness and disability with or without the support of a health worker" [1]. Self-care may include, but is not limited to, health literacy, self-awareness of physical and mental health, physical activity, healthy eating, good hygiene, and rational use of medicines and diagnostics [2]. By extension, self-care interventions are those that can support or promote people's self-care [3]. Self-care interventions can act to complement or even broaden delivery of health services, ideally improving health outcomes and care experiences [4,5]. Humanitarian crises, emergencies and pandemics introduce additional challenges to the provision of routine healthcare services [6,7]. In this context, self-care interventions can help ensure health and well-being is maintained with reduced in-person contact [8].

In 2021, WHO published a guideline on self-care interventions for health and wellbeing, which included 13 recommendations on selected self-care interventions to improve preconception, antenatal, intrapartum and postpartum care [3]. By encouraging women to engage in these self-care interventions, the overall efficiency of healthcare delivery can be enhanced. In addition, there are interventions for pregnant and postpartum women that are not currently included in WHO's self-care guideline, but have the potential of transitioning into a self-care paradigm. Examples include prescription medicines that can be made available over-the-counter, self-collection of samples for testing, or home testing kits for certain diseases. Some systematic reviews have been conducted on self-care interventions in pregnancy [9–11]; however these previous reviews focused on a few selected interventions and outcomes, and mainly the antenatal or postpartum periods. No previous review has explored the broad and heterogeneous body of research across preconception, antenatal, intrapartum and postpartum periods, in order to identify those that may be effective or applicable from a self-care perspective. We therefore aimed to identify all current and emerging self-care interventions via a scoping review that have been evaluated in the context of preconception, antenatal, intrapartum and postpartum care.

For peer review only - http://bmjopen.bmj.com/site/about/guidelines.xhtml

METHODS

The scoping review was conducted in accordance with the methodological guide for scoping reviews by Peters and colleagues [12], and reported in accordance with the PRISMA guideline for Scoping Reviews [13] (Supplementary File S1). The protocol was registered online at https://doi.org/10.17605/OSF.IO/97X25 (Supplementary File S2).

Electronic searches

The search strategy was developed in consultation with an information specialist (Supplementary File S3) and combined self-care concepts with terms related to preconception, antenatal, intrapartum and postpartum care, based on the framework proposed by Narasimhan and colleagues [4]. The terms and concepts used were informed by previous reviews of pregnancy care and self-care for other health conditions [14–16] and by inputs from team members with clinical and public health background. The following databases were systematically searched to identify published studies from inception to October 17th, 2021: Medline, Embase, Emcare and PsycINFO (via Ovid), CENTRAL/Cochrane Database of Systematic Reviews (via Cochrane Library) and CINAHL Plus (via EBSCOhost). The last date of search was October 17th, 2021. Reference lists of included studies were manually screened to identify other relevant studies. Grey literature was not searched after considering the volume and variable quality of such evidence [17].

Study selection

Two reviewers (PN and CC) first independently screened all titles and abstracts to exclude irrelevant studies, and then independently reviewed potentially eligible full texts based on pre-determined

For peer review only

For peer review only - http://bmjopen.bmj.com/site/about/guidelines.xhtml

eligibility criteria. All stages of screening were conducted using Covidence software [18]. Any disagreement was resolved by discussion or adjudication by a third reviewer (JPV).

The eligibility criteria for inclusion were: (A) Participants: women preparing to get pregnant, currently pregnant, giving birth, or in the postpartum period (up to 42 days after birth), of any age. Studies in which partners or family members carried out self-care for the pregnant woman, were also eligible. Interventions for abortion were not eligible. We also excluded interventions that were only directed at newborns and postnatal breastfeeding interventions for improving neonatal outcomes, though antenatal programmes related to preparation for the postnatal period were eligible. (B) Interventions: For this review, a self-care intervention was defined as any tool, resource or strategy designed to promote or facilitate self-care by a woman intending to get pregnant, or a currently pregnant, labouring or in the postpartum period, for the purpose of improving the quality or coverage of maternity health care, and/or improving her health, well-being and care experiences. To be eligible, interventions must have been aimed at reducing risks of pregnancy, childbirth or postpartum complications and/or enabling a positive pregnancy, childbirth or postpartum experience. Each intervention was evaluated independently by two reviewers for inclusion based on a set of pre-specified principles (Supplementary File S4). (C) Comparator: placebo, no treatment or standard care; single-arm interventional studies were also eligible. (D) Outcomes: outcomes related to maternal physical, psychological or emotional health, behavioural change affecting a mother's own health, or diagnostic accuracy of tests for women. Studies reporting economic outcomes only were not included, and neither were feasibility studies evaluating only retention rates and acceptability. (E) Types of studies: randomized trials, quasi-experimental studies and cohort studies, scoping reviews, systematic reviews, meta-analyses and overviews of systematic reviews. Systematic reviews and overviews must have stated some form of search strategy and presented all included studies in a systematic manner to be eligible for inclusion. Ongoing studies (defined as trials with protocols published from 2019 onwards which were not completed) were eligible; no restriction on date of publication was applied otherwise. Case series, case studies, case reports, dissertations and theses, reports published as

For peer review only - http://bmjopen.bmj.com/site/about/guidelines.xhtml

abstracts only, correspondence letters, editorials, and qualitative studies were excluded. Only studies with full texts written in English were eligible.

Data collection and analysis

Two reviewers independently extracted data using a standardised extraction form we developed to suit the review (Supplementary File S5). Data extracted included characteristics of each study's population, interventions, and outcomes measured. Study designs were classified as evidence syntheses, interventional studies, observational studies or diagnostic accuracy studies. Countries where studies took place were classified based on the World Bank 2021 country income levels [19]. For population characteristics, we recorded whether the studies included women under the age of 18, and whether those with high-risk pregnancies were included. For each study, the self-care activities that the intervention aimed to promote or facilitate, and the period of pregnancy during which the intervention took place (preconception, antenatal, intrapartum or postpartum period) were recorded. An inductive thematic analysis process was undertaken [20]. After all self-care interventions were identified, we analysed their descriptions and identified themes among groups of interventions. For example, interventions involving taking dietary supplements or adjusting diet intakes were grouped under the theme "Diet and nutrition". These themes were refined until all interventions were accounted for, creating the final set of self-care categories. In addition, we matched the interventions to the categories listed in WHO's classification of self-care interventions for self-carers and caregivers [21]. We also recorded the venues of access to self-care, i.e. the setting or location where participants were orientated to or carried out self-care, based on this classification framework [21]. Lastly, we recorded whether any external support person was required to promote or facilitate self-care. These information were recorded for primary studies only, as evidence synthesis studies may include studies with different characteristics. Any disagreement was resolved via consensus or adjudication by a third reviewer (JPV). In line with scoping review methodology [12], quality assessment of included studies was not performed. Descriptive statistics (frequency and percentage)

For peer review only - http://bmjopen.bmj.com/site/about/guidelines.xhtml

156 were used to report study, participant and intervention characteristics. Studies that feature more than three self-care interventions were analysed and reported separately. R v4.1.2 was used to calculate the frequencies of various intervention characteristics, participant characteristics and outcomes for each self-care intervention, aggregate these frequencies by self-care categories and present them as bar charts.

Patient and Public Involvement Statement: Patients were not included in the design, conduct and plans for dissemination of results of this review.

RESULTS

A total of 12,215 records from six databases were retrieved, together with an additional 182 records from manual reference screening (Fig. 1). In total, 10,712 reports were excluded at the title/abstract screening stage and another 743 reports at full text screening (Supplementary File S6). The final sample consisted of 580 unique studies (616 reports) (Supplementary File S7), 18 (3%) of which were ongoing studies.

Fig. 1. PRISMA flowchart of record retrieval and selection

Characteristics of included studies

Among 580 studies, two-thirds were interventional studies (n=365, 63%), many of which reported results of randomised trials (n=306, 53%). Evidence syntheses (n=165) accounted for 28% of all studies, out of which 109 were meta-analyses (19%). Observational studies were less common (n=32, 6%) and were mainly retrospective or prospective cohort studies (n=25, 4%). Study designs used were largely similar across all self-care categories, with some exceptions (Fig. 2A, Supplementary File S8). In self-management of medication, the proportion of non-randomised studies (n=7, 41%)

For peer review only - http://bmjopen.bmj.com/site/about/guidelines.xhtml
For peer review only

was similar to that of randomised trials (n=6, 35%). In self-testing and self-collection of samples, many studies (n=10, 56%) were diagnostic accuracy studies.

Most studies took place in high-income countries (n=260, 45%), followed by middle-income (n=146, 25%) and low-income countries (n=11, 2%); 163 studies (28%) were reviews with no geographic restriction, and usually included studies from multiple countries. Countries with the most studies were the United States of America (USA) (n=86, 15%), Iran (n=46, 8%), United Kingdom (UK) (n=33, 6%) and Australia (n=30, 5%). Self-care interventions most commonly took place in the antenatal (n=439, 76%) and postpartum periods (n=119, 21%). Few studies related to the preconception (n=21, 4%) or intrapartum periods (n=62, 11%) (Fig. 2B, Supplementary File S8).

Fig. 2. Characteristics of included studies

Thirty-eight studies (7%) included women under the age of 18. A third of included studies (n=170, 29%) included women with high-risk pregnancies. High-risk pregnancies were variably defined – the most common definitions included pregnant women with gestational or existing diabetes (n=54, 9%), obesity or overweight (n=35, 6%) and ex-/current smokers (n=31, 5%).

Self-care interventions and their characteristics

Among included studies, 35 studies (6%) described a complex intervention involving more than three different self-care activities (Supplementary File S9). The remaining 545 studies (94%), which featured up to three self-care activities, constituted the final sample for identification of self-care interventions. A total of 112 unique self-care interventions were identified and organised into 11 broad categories (Fig. 3; Supplementary File S10). The most common self-care categories identified included diet and nutrition (n=143/545, 26%), physical activity (n=132, 24%), psycho-social strategies (n=97, 18%) and lifestyle adjustments (n=90, 17%). Few studies featured self-care interventions under the categories of sexual health and family planning (2%), self-management of medication (3%), and self-testing/sampling (3%). All identified interventions demonstrate various

For peer review only - http://bmjopen.bmj.com/site/about/guidelines.xhtml

degrees of interaction with the health system [4], from little to no direct contact (self-awareness/regulation), some interaction (self-management of health conditions) and close linkage with the health system for follow-up (self-testing & diagnosis) (Fig. 3).

Fig. 3. All self-care interventions identified from included studies and their interactions with the health system

The identified interventions correspond to a range of categories from WHO's classification of self-care interventions (Supplementary File S11). The most common WHO categories were interventions for improving capacity and promoting autonomy in self-care (n=325, 56%), self-care prevention including risk avoidance and support for physical and mental health and well-being (n=296, 51%) and self-care for short-term health conditions (n=189, 33%). Some WHO categories did not feature amongst included studies, such as on-demand information services for health information, diagnostic devices in community locations, online symptom checkers for diagnosis, and interventions enabling individuals to identify location of health facilities/structures. Notably, we identified no studies that targeted self-care outside the context of a specific health issue, such as campaigns to promote awareness about self-care or programmes to improve health and digital literacy.

Among 545 studies under analysis, self-care was accessible through several avenues as described in the WHO classification framework [20]. These avenues include health facilities (n=238/545, 44%), digital platforms (n=97, 18%), community and non-facility venues (n=74, 14%), partner or peer support (n=37, 7%) or over-the-counter (OTC) products e.g. nutritional supplements, topical products, or support devices (n=69, 13%). In 80 studies (15%), there was insufficient detail to determine that the aforementioned modes of access were used. No studies were identified where the intervention took place at the workplace, in schools, or in emergency or humanitarian settings.

Across the self-care categories, provision of information and demonstration of self-care were most commonly done in health facilities, especially for self-testing (94% of studies in this category) and self-monitoring (58%). Provision of similar services outside health facilities, such as via community health workers or at community-based venues, was mainly for the support of self-management of

For peer review only - http://bmjopen.bmj.com/site/about/guidelines.xhtml

medication (41%) and physical activity (28%). Home-based self-care (i.e. direct access to self-care resources and information at home) was less frequent, mainly in the categories of breast or nipple care (23%) and lifestyle adjustments (20%). Digital platforms were popular in some categories, notably self-monitoring (51%). Use of OTC products was common in complementary/alternative therapies (39%) and breast and nipple care (27%). Lastly, peer or partner support was relatively less common across categories (7%), and we observed that partner support (1%) was relatively less frequent than support from peers (e.g. other women, members of support groups) (6%) (Fig. 4A, Supplementary File S8).

A large number of studies described the involvement of health workers in promoting or facilitating self-care (n=208/545, 38%), especially for sexual health & family planning (67% of studies in this category), psychosocial strategies (45%) or physical activity (44%) (Fig. 4B, Supplementary File S8). Few studies mentioned involvement of trained community health workers (n=41, 8%), non-trained community members (n=25, 5%) or family members (n=3, 1%).

Fig. 4. Characteristics of self-care interventions

Types of outcomes in self-care studies

Included studies used a diverse range of outcomes, including both maternal and neonatal outcomes (Supplementary Table 1)., the most commonly measured ones were neonatal outcomes (birth weight, Apgar scores, preterm birth, admission to neonatal intensive care unit and other neonatal complications) (n=177, 31%); maternal mental wellbeing and quality of life (n=150, 26%), and labour outcomes (duration of labour stages, labour pain, mode of delivery or labour induction methods) (n=125, 22%). A total of 147 studies (25%) measured behavioural outcomes, mainly related to reductions in risky behaviours such as smoking or alcohol consumption. Maternal mortality (n=20, 3%) and perinatal mortality (n=67, 12%) were less commonly evaluated.

For peer review only - http://bmjopen.bmj.com/site/about/guidelines.xhtml

DISCUSSION

Main findings

In this scoping review, 580 studies were identified pertaining to 112 self-care interventions across preconception, antenatal, intrapartum and postpartum periods. These interventions encompassed multiple modalities, settings and levels of provider involvement. Most self-care interventions required close engagement with health facilities, in which healthcare workers or community health workers would teach self-care skills or provide therapeutics for home use. Previous studies also suggest that healthcare services are a critical starting point for successful self-care approaches [22–24]. As such, self-care should be viewed as an extension of, and not a separate entity to, formal healthcare services. Our findings also reflect a broader trend towards task-sharing in order to increase maternity care coverage [7,25]. While the majority of self-care interventions were implemented in the antenatal period, many of these studies aimed to equip pregnant individuals with self-care skill via workshops or counselling, which can be used in the intrapartum (e.g. relaxation methods during labour) or postpartum periods (e.g. pelvic floor exercises, breast and nipple care). Few studies examined the potential benefits of self-care at the pre-conception period. Few interventions featured digital platforms (e.g. websites, mobile apps); those identified were mostly used for communication between the pregnant individual and a remote health worker, or as a platform for the pregnant individual to record her medical information and receive automatic, individualised health advice. While digital technologies seem to currently play a limited role, they have great potential to catalyse and promote maternity-related self-care [26].

Most studies concentrated on self-care interventions related to lifestyle modification - such as diet, nutrition/supplement intake, physical activity and sleep habits - as well as strategies to optimise mental and social well-being. The established evidence base for lifestyle interventions was also reflected in previous systematic reviews on self-care for preeclampsia and gestational diabetes [9,11]. This might be attributable to a perceived lower risk of harm when conducting trials investigating

For peer review only - http://bmjopen.bmj.com/site/about/guidelines.xhtml

lifestyle interventions, compared to clinical or pharmacological interventions. Moreover, lifestyle interventions tend to take place in their own living environments and in their own time, and hence may be more amenable to self-care approaches. Conversely, there were relatively few studies that explored self-monitoring, self-testing or self-management of medications or pregnancy complications. While studies of these self-care approaches can be complex or technically challenging, they could provide solutions to improving care in remote and low-resource settings [6]. While advanced distribution and self-administration of misoprostol for postpartum haemorrhage prevention has been well-explored [27–29], there was limited or no research on similar approaches to other therapeutics, such as use of anthelminthics, malaria prophylaxis, antimalarials, antiretroviral therapy, or vitamin and micronutrient supplementation. Although many studies reported on the prevalence and determinants of self-medication during pregnancy [30–32], none investigated interventions aimed at promoting safe self-medication practices, or how to seek information when self-medicating. There were also few self-care interventions aimed at communicable disease detection and prevention during or after pregnancy. Future research is warranted to investigate how these aspects of clinical care during and after pregnancy could be approached from a self-care perspective.

We identified studies describing self-testing options for urinary tract infections and HIV, but few or no studies on malaria, syphilis or chlamydia self-testing, for which rapid test technologies are available but not yet scaled up or tested in pregnancy [33–35]. Similarly, self-collection of vaginal samples for reproductive tract infections is an emerging alternative to clinic-based sampling [36]. While some studies have investigated diagnostic accuracy of self-sampling for reproductive tract infections in pregnancy, evidence on their benefits, harms, and effective linkages with health systems is currently lacking [37]. Lastly, we identified self-care studies on insecticide-treated nets for malaria, mostly from the sub-Saharan Africa region. However there was little research on other low-cost strategies amenable to a self-care approach (indoor residual spraying and insect repellents), or from other malaria-endemic regions such as Asia and Latin America [38].

For peer review only - http://bmjopen.bmj.com/site/about/guidelines.xhtml

Last but not least, we found no eligible studies on traditional and indigenous self-care practices. While important qualitative research has been conducted on this topic [39,40] , we hypothesise that there is a relative lack of studies exploring the health effects of these types of self-care practices. Future research on these topics could also provide new opportunities for improving cultural safety of maternity care services.

Strengths and limitations

While there are systematic reviews for some self-care practices, this is the first scoping review that maps all potential self-care practices across the spectrum of pregnancy care, from preconception, antenatal, intrapartum to postnatal care. This scoping review provides a comprehensive summary of the state of research on this topic, and lays the foundation for future systematic reviews and primary research. We analysed studies based on multiple characteristics, as well as applying the new WHO self-care classification system to organise, categorise and facilitate interpretation of findings.

This scoping review was intended to present an overview of the research landscape, and not to critically appraise or meta-analyse available evidence. The total number of studies identified for a given self-care intervention may not correlate with evidence quality, or its effectiveness. We reported the results for complex interventions (i.e. interventions with more than three different self-care practices) separately (Supplementary File S9), since segregating each complex intervention into multiple individual interventions would overestimate their representation in the sample and disregard potential synergy between the self-care components. Besides, because these studies did not introduce any new self-care intervention, or a substantive number of studies for any single self-care intervention that we had already identified, it is unlikely that including them into the main analysis would change our results and conclusion. Although the number of such studies is small (n=35, 6%), they represent how self-care can be implemented in real-life, and future reviews should study their effects using appropriate methods e.g. component network meta-analysis [41] [42].

For peer review only - http://bmjopen.bmj.com/site/about/guidelines.xhtml

For peer review only

Some limitations must nonetheless be acknowledged. We did not formally screen those primary studies reported within an included evidence synthesis, and we assessed intervention and outcomes characteristics based on the review's summary description of the included studies. It is possible that the number of primary studies was there underestimated, or the characteristics of individual primary studies might differ. Our assessment found 45 primary studies (8% of all included reports) that were also included within an evidence synthesis included in this review. Given their small number, we consider it unlikely that these overlapping studies would substantively affect the overall results. Hence, the distribution of study designs (Fig. 2A, Supplementary File S8) should be considered in addition to the volume of evidence available for each intervention. We opted to focus on maternity-related self-care, thus excluding interventions that took place after 42 days postpartum, or those that related primarily to care of newborns and breastfeeding. Our research objectives also mean that we did not include qualitative studies. These exclusions do not discount their importance, but rather acknowledge that these topics require separate, dedicated reviews. Our search of published reports means that self-care interventions that have only been evaluated in unpublished studies or grey literature would not have been identified. With 45 reports excluded as not written in English (3.3% of all full-text reports), language bias is possible.

Future research

This review was conducted for the purpose of guiding further evidence synthesis activities and assisting the WHO with guideline development in this research area. This scoping review identified two distinct groups of self-care interventions, which have different pathways forward. The first group are those interventions with a substantial number of primary studies, where no systematic review has been conducted or only outdated systematic reviews exist. For these interventions – such as use of clean cookstoves to reduce adverse birth outcomes associated with indoor air pollution, self-care interventions to reduce postpartum depression, or self-collection of vaginal samples to test for sexually transmitted diseases - up-to-date systematic reviews are warranted to inform guidelines,

For peer review only - http://bmjopen.bmj.com/site/about/guidelines.xhtml

clinical practice and self-care programs. The second group are interventions with limited or no primary studies, or where available primary studies were not designed to fully deliver the intervention in a self-care approach. For these interventions, well-designed primary studies that compare a self-care approach with standard care are needed. Examples include self-testing for proteinuria, or self-measurement of vaginal pH to detect early signs of bacterial vaginosis. Some self-care interventions may be particularly useful in limited-resource settings, and thus should be evaluated in such settings.

CONCLUSION

Self-care interventions during pre-conception, antepartum, intrapartum and postpartum periods are diverse in scope and implementation characteristics. Many self-care interventions were implemented with support from the health system at initial stages of use and uptake. Some promising self-care interventions require further primary research, though several are more matured and up-to-date evidence syntheses are needed. Research on self-care in the pre-conception period is lacking. Future research should explore task-sharing from clinical care to home or community settings, or effective self-management of complications where access to care is unavailable.

List of abbreviations

HIV: Human Immunodeficiency Virus

OTC: over-the-counter

WHO: World Health Organization

DECLARATIONS

Ethics approval and consent to participate: Not applicable. This study does not involve human participants and ethical approval was not required.

For peer review only

For peer review only - http://bmjopen.bmj.com/site/about/guidelines.xhtml

Consent for publication: Not applicable. This study does not collect personal information from an identifiable individual and informed consent was not required.

Availability of data and materials: Data supporting the findings and conclusion of this study are included in this published article and its supplementary files. The underlying dataset and analytic code are available at osf.io/26f7y

Competing interests: None declared.

Funding: This project was funded by UNDP/UNFPA/UNICEF/WHO/World Bank Special Programme of Research, Development and Research Training in Human Reproduction (HRP), Department of Sexual and Reproductive Health and Research, World Health Organization (# PO 202724071). While members of our authorship team (MN, MB, OT) are employed by HRP/WHO, the funder organization had no role in the design and conduct of the study; collection, management, analysis, and interpretation of the data; preparation, review, or approval of the manuscript; and decision to submit the manuscript for publication.

Acknowledgement: We thank Steve McDonald (Cochrane Australia) for his guidance in formulating the search strategy.

Author contributions

The corresponding author attests that all listed authors meet authorship criteria and that no others meeting the criteria have been omitted. P-YN and JPV conceptualised the study, wrote the protocol and statistical plan and organised collaborative meetings. P-YN and CC screened the literature and extracted the data. P-YN analysed the data, wrote the original draft and revised the paper. MJP advised on the design of the study. CC, AW, KB, MJP, AMG, MN, MB, OT, JPV participated in technical and clinical discussions, and reviewed and revised the paper.

Supplementary Files

For peer review only

For peer review only - http://bmjopen.bmj.com/site/about/guidelines.xhtml

Supplementary Table 1. Evidence map for outcomes measured

Supplementary File S1: Preferred Reporting Items for Systematic reviews and Meta-Analyses extension for Scoping Reviews (PRISMA-ScR) Checklist

Supplementary File S2: Protocol as registered on Open Science Framework

Supplementary File S3: Search strategy for all databases

Supplementary File S4: Defining self-care interventions, a conceptual framework and examples for making judgements on specific interventions

Supplementary File S5: Data collection form

Supplementary File S6: List of excluded full texts

Supplementary File S7: Reference list of included studies

Supplementary File S8. Characteristics of studies by category

Supplementary File S9: Complex interventions and their characteristics

Supplementary File S10: List of all self-care interventions identified from included studies

Supplementary File S11: Categorising self-care interventions based on WHO's classification framework

For peer review only

For peer review only - http://bmjopen.bmj.com/site/about/guidelines.xhtml

References

1 World Health Organization. Self-care interventions for health. Fact sheet. 2021.https://www.who.int/news-room/fact-sheets/detail/self-care-health-interventions (accessed 29 May 2021).

2 International Self-Care Foundation. The Seven Pillars of Self-Care. https://isfglobal.org/practise-self-care/the-seven-pillars-of-self-care/ (accessed 13 Jan 2022).

3 World Health Organization. Guideline on Self-Care Interventions for Health and Well-Being. Geneva: 2021. https://www.who.int/publications/i/item/9789240030909

4 Narasimhan M, Allotey P, Hardon A. Self care interventions to advance health and wellbeing: A conceptual framework to inform normative guidance. *BMJ* 2019;**365**. doi:10.1136/bmj.l688

5 Narasimhan M, Logie CH, Gauntley A, *et al.* Self-care interventions for sexual and reproductive health and rights for advancing universal health coverage. *Sex Reprod Heal matters* 2020;**28**. doi:10.1080/26410397.2020.1778610

6 Pavlidis P, Eddy K, Phung L, *et al.* Clinical guidelines for caring for women with COVID-19 during pregnancy, childbirth and the immediate postpartum period. Women and Birth. 2020. doi:10.1016/j.wombi.2020.10.015

7 Gülmezoglu AM, Ammerdorffer A, Narasimhan M, *et al.* Self-care and remote care during pregnancy: A new paradigm? *Heal Res Policy Syst* 2020;**18**:1–3. doi:10.1186/S12961-020-00627-4

8 World Health Organization. Maintaining essential health services: operational guidance for the COVID-19 context, interim guidance. 2020.https://www.who.int/publications/i/item/WHO-2019-nCoV-essential_health_services-2020.2 (accessed 21 Jan 2022).

9 Rasouli M, Pourheidari M, Gardesh Z. Effect of Self-care Before and During Pregnancy to Prevention and Control Preeclampsia in High-risk Women. *Int J Prev Med* 2019;**10**. doi:10.4103/IJPVM.IJPVM_300_17

10 Dalrymple KV, Flynn AC, Poston L, *et al.* Preventing postpartum weight retention: Lifestyle interventions in pregnancy, postpartum or both. A systematic review of the literature. *Proc Nutr Soc* 2018;**77**:77. doi:10.1017/S002966511800191X

11 Kolivand M, Rahimi MA, Shariati M, *et al.* The Effect of Self-care Educational/Training Interventions on the Outcomes of Gestational Diabetes: A Review Article. *Iran J Public Health* 2018;**47**:1805.https://www.ncbi.nlm.nih.gov/pmc/articles/PMC6379603/ (accessed 27 Apr 2022).

12 Peters MDJ, Godfrey CM, Khalil H, *et al.* Guidance for conducting systematic scoping reviews. *Int J Evid Based Healthc* 2015;**13**:141–6. doi:10.1097/XEB.0000000000000050

13 Tricco AC, Lillie E, Zarin W, *et al.* PRISMA extension for scoping reviews (PRISMA-ScR): Checklist and explanation. Ann. Intern. Med. 2018;**169**:467–73. doi:10.7326/M18-0850

14 Abalos E, Chamillard M, Diaz V, *et al.* Antenatal care for healthy pregnant women: a mapping of interventions from existing guidelines to inform the development of new WHO guidance on antenatal care. *BJOG* 2016;**123**:519–28. doi:10.1111/1471-0528.13820

15 Donald M, Kahlon BK, Beanlands H, *et al.* Self-management interventions for adults with chronic kidney disease: a scoping review. *BMJ Open* 2018;**8**:e019814.

For peer review only - http://bmjopen.bmj.com/site/about/guidelines.xhtml

doi:10.1136/BMJOPEN-2017-019814

16 Foster G, Taylor SJC, Eldridge SE, *et al.* Self-management education programmes by lay leaders for people with chronic conditions. *Cochrane Database Syst Rev* 2007.

17 Benzies KM, Premji S, Hayden KA, *et al.* State-of-the-Evidence Reviews: Advantages and Challenges of Including Grey Literature. *Worldviews Evidence-Based Nurs* 2006;**3**:55–61. doi:10.1111/J.1741-6787.2006.00051.X

18 Veritas Health Innovation. Covidence Systematic Review Software, Melbourne Australia. 2020.www.covidence.org (accessed 22 Jul 2021).

19 World Bank. The World by Income and Region. 2021.https://datatopics.worldbank.org/world-development-indicators/the-world-by-income-and-region.html (accessed 20 Jan 2022).

20 Braun V, Clarke V. *Thematic analysis.* 2012. doi:10.1037/13620-004

21 World Health Organization. Classification of self-care interventions for health: a shared language to describe the uses of self-care interventions. Geneva: 2021. https://www.who.int/publications/i/item/9789240039469 (accessed 6 Apr 2022).

22 Bell J, Dziekan G, Pollack C, *et al.* Self-Care in the Twenty First Century: A Vital Role for the Pharmacist. *Adv Ther* 2016;**33**:1691–703. doi:10.1007/S12325-016-0395-5

23 Davies NJ. Improving self-management for patients with long-term conditions. *Nurs Stand* 2010;**24**. doi:10.7748/NS2010.02.24.25.49.C7562

24 Lorig KR, Holman HR. Self-Management Education: History, Definition, Outcomes, and Mechanisms. *Ann Behav Med* 2003;**26**:1–7.https://academic.oup.com/abm/article/26/1/1/4630312 (accessed 13 Jan 2022).

25 Dawson AJ, Buchan J, Duffield C, *et al.* Task shifting and sharing in maternal and reproductive health in low-income countries: a narrative synthesis of current evidence. *Health Policy Plan* 2014;**29**:396–408. doi:10.1093/HEAPOL/CZT026

26 DeNicola N, Grossman D, Marko K, *et al.* Telehealth Interventions to Improve Obstetric and Gynecologic Health Outcomes: A Systematic Review. *Obstet Gynecol* 2020;**135**:371–82. doi:10.1097/AOG.0000000000003646

27 Oladapo OT, Blum J, Abalos E, *et al.* Advance misoprostol distribution to pregnant women for preventing and treating postpartum haemorrhage. *Cochrane Database Syst Rev* 2020;**2020**. doi:10.1002/14651858.CD009336.PUB3

28 Onongé S, Campbell OMR, Kaharuza F, *et al.* Effectiveness and safety of misoprostol distributed to antenatal women to prevent postpartum haemorrhage after child-births: A stepped-wedge cluster-randomized trial. *BMC Pregnancy Childbirth* 2015;**15**. doi:10.1186/S12884-015-0750-6

29 Weeks AD, Ditai J, Ononge S, *et al.* The MamaMiso study of self-administered misoprostol to prevent bleeding after childbirth in rural Uganda: A community-based, placebo-controlled randomised trial. *BMC Pregnancy Childbirth* 2015;**15**:1–10. doi:10.1186/S12884-015-0650-9

30 Atmadani RN, Nkoka O, Yunita SL, *et al.* Self-medication and knowledge among pregnant women attending primary healthcare services in Malang, Indonesia: A cross-sectional study. *BMC Pregnancy Childbirth* 2020;**20**:1–11. doi:10.1186/S12884-020-2736-2

31 Niriayo YL, Mohammed K, Asgedom SW, *et al.* Self-medication practice and contributing

For peer review only - http://bmjopen.bmj.com/site/about/guidelines.xhtml

For peer review only

factors among pregnant women. *PLoS One* 2021;**16**:e0251725. doi:10.1371/JOURNAL.PONE.0251725

32 Mohseni M, Azami-Aghdash S, Sheyklo SG, *et al.* Prevalence and reasons of self-medication in pregnant women: A systematic review and meta-analysis. Int. J. Community Based Nurs. Midwifery. 2018;**6**.

33 Berthod D, Rochat J, Voumard R, *et al.* Self-diagnosis of malaria by travellers: A cohort study on the use of malaria rapid diagnostic tests provided by a Swiss travel clinic. *Malar J* 2017;**16**:1–8. doi:10.1186/S12936-017-2079-2

34 Wang C, Cheng W, Li C, *et al.* Syphilis Self-testing: A Nationwide Pragmatic Study Among Men Who Have Sex With Men in China. *Clin Infect Dis* 2020;**70**:2178–86. doi:10.1093/CID/CIZ603

35 Sacks-Davis R, Gold J, Aitken CK, *et al.* Home-based chlamydia testing of young people attending a music festival - Who will pee and post? *BMC Public Health* 2010;**10**:1–9. doi:10.1186/1471-2458-10-376

36 Fajardo-Bernal L, Aponte-Gonzalez J, Vigil P, *et al.* Home-based versus clinic-based specimen collection in the management of Chlamydia trachomatis and Neisseria gonorrhoeae infections. *Cochrane Database Syst Rev* 2015;**2015**. doi:10.1002/14651858.CD011317.PUB2

37 Siegfried N, Narasimhan M, Logie CH, *et al.* Prioritising gender, equity, and human rights in a GRADE-based framework to inform future research on self care for sexual and reproductive health and rights. *BMJ Glob Heal* 2020;**5**:2128. doi:10.1136/BMJGH-2019-002128

38 Menéndez C, D'Alessandro U, ter Kuile FO. Reducing the burden of malaria in pregnancy by preventive strategies. *Lancet Infect Dis* 2007;**7**:126–35. doi:10.1016/S1473-3099(07)70024-5

39 Raman S, Nicholls R, Ritchie J, *et al.* Eating soup with nails of pig: thematic synthesis of the qualitative literature on cultural practices and beliefs influencing perinatal nutrition in low and middle income countries. *BMC Pregnancy Childbirth* 2016;**16**. doi:10.1186/S12884-016-0991-Z

40 Sarmiento I, Paredes-Solís S, Dion A, *et al.* Maternal health and Indigenous traditional midwives in southern Mexico: contextualisation of a scoping review. *BMJ Open* 2021;**11**:e054542. doi:10.1136/BMJOPEN-2021-054542

41 Welton NJ, Caldwell DM, Adamopoulos E, *et al.* Mixed Treatment Comparison Meta-Analysis of Complex Interventions: Psychological Interventions in Coronary Heart Disease. *Am J Epidemiol* 2009;**169**:1158–65. doi:10.1093/AJE/KWP014

[dataset] [42] Nguyen PY, Caddy C, Wilson AN, Blackburn K, Page MJ, Gülmezoglu AM, Narasimhan M, Bonet M, Tunçalp O, Vogel JP. Data from: Self-care interventions for preconception, antenatal, intrapartum and postpartum care: a scoping review. Open Science Framework, December 2021. DOI: 10.17605/OSF.IO/26F7Y

For peer review only - http://bmjopen.bmj.com/site/about/guidelines.xhtml

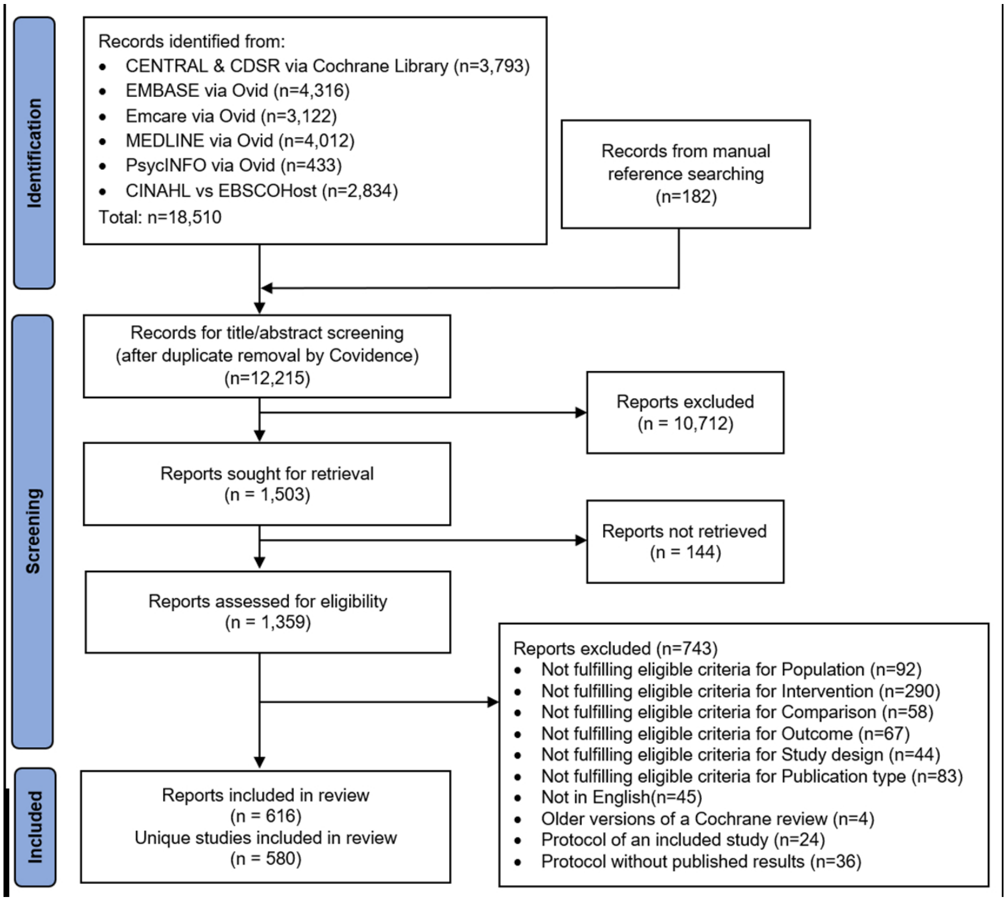

Fig. 1. PRISMA flowchart of record retrieval and selection

102x91mm (300 x 300 DPI)

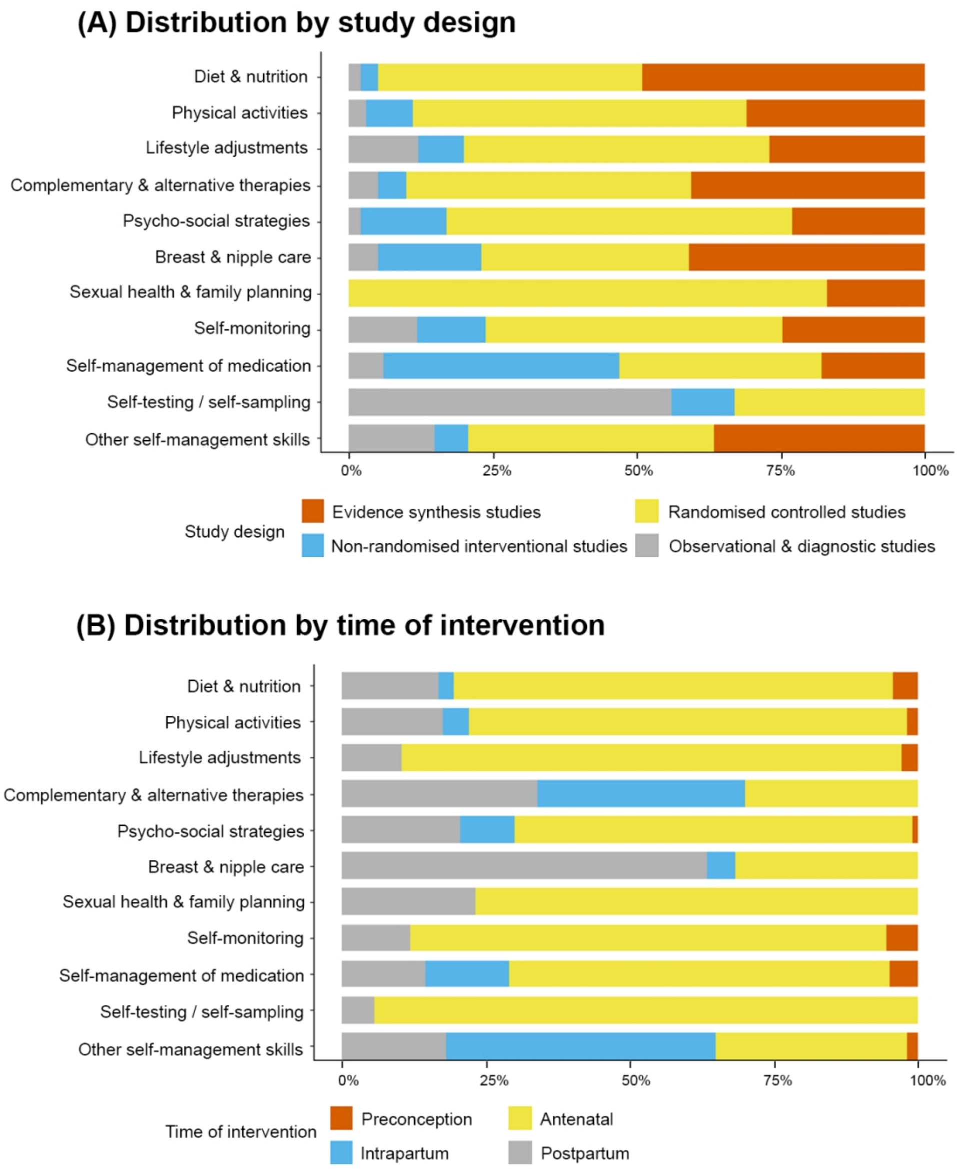

Fig. 2. Characteristics of included studies

118x145mm (300 x 300 DPI)

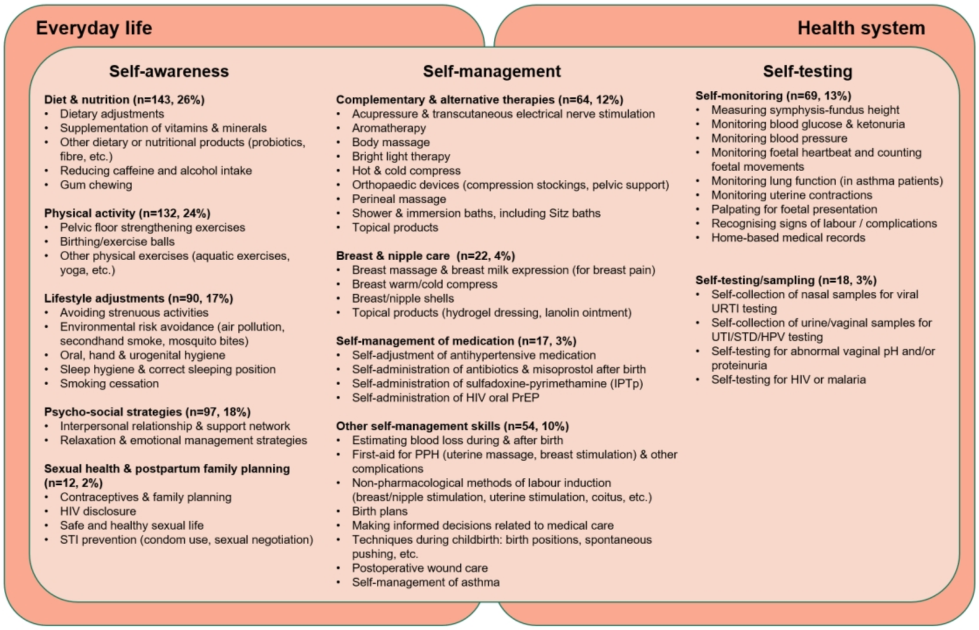

Some studies feature more than one self-care activities, hence the percentages do not total to 100%.
Some primary studies (n=45, 8%) are also included in evidence synthesis studies featured in this review.
For further details on each intervention, see Supplementary File S10.

Fig. 3. All self-care interventions identified from included studies and their interactions with the health system

149x105mm (300 x 300 DPI)

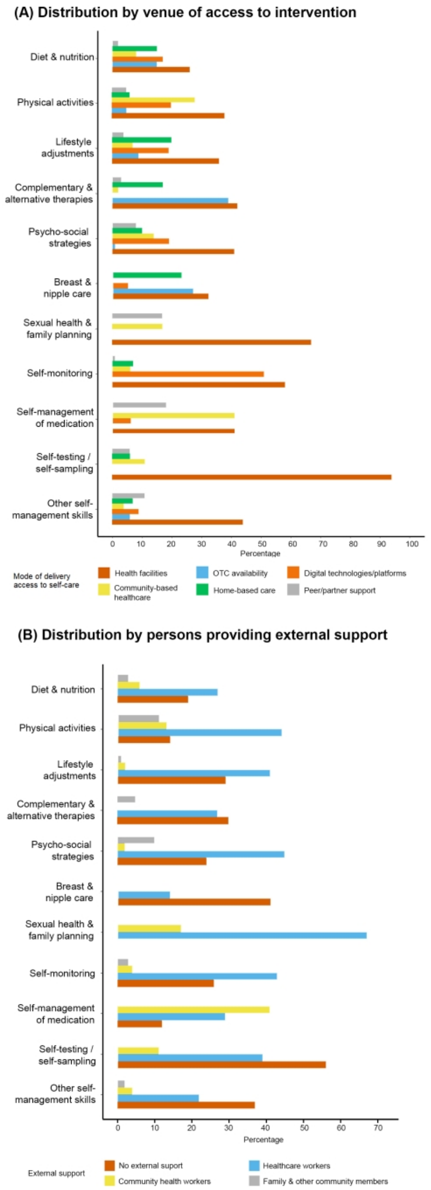

Fig. 4. Characteristics of self-care interventions

131x360mm (300 x 300 DPI)

Supplementary Table 1. Evidence map for outcomes measured

Categories \ Outcome	Maternal mortality	Perinatal mortality	Neonatal outcomes	Labour outcomes	Anthropometric & cardiometabolic outcomes	Diabetes & other metabolic disorders	Hypertensive disorders of pregnancy	Postpartum infection & haemorrhage	Urogenital outcomes	Nutritional deficiencies	Gastrointestinal outcomes	Other maternal complications	Infectious diseases	Other minor physiological discomforts	Mental wellbeing & quality of life	Behavioural change	Breastfeeding	Other
Diet & nutrition	5	29	71	27	53	47	34	4	9	11	12	14	3	3	21	42	7	0
Physical activity	0	6	48	33	58	45	22	7	5	1	1	9	0	13	40	50	7	0
Lifestyle adjustments	0	9	16	1	0	1	4	0	1	0	1	5	12	9	11	49	1	0
Complementary & alternative therapies	0	3	15	26	1	1	2	2	14	0	6	3	0	10	23	1	8	0
Psycho-social strategies	0	3	16	23	1	6	5	1	4	0	1	4	0	9	75	8	7	2
Breast & nipple care	0	3	6	4	0	2	1	1	0	0	0	0	0	16	1	2	17	1
Sexual health & family planning	0	0	1	0	0	0	0	0	0	0	0	1	1	0	7	9	0	0
Self-monitoring	3	18	39	28	23	27	24	3	0	0	0	8	0	0	15	17	1	1
Self-management of medication	6	4	7	0	1	0	1	5	0	0	0	4	4	0	1	12	0	0
Self-testing or sampling	1	2	4	1	1	0	1	0	0	0	0	0	1	0	0	5	0	1
Other self-management skills	5	5	16	27	0	1	5	17	3	0	0	10	0	1	17	4	1	3
**All self-care categories**	**20<br>3%**	**67<br>12%**	**177<br>31%**	**125<br>22%**	**78<br>13%**	**79<br>14%**	**65<br>11%**	**33<br>6%**	**32<br>6%**	**11<br>2%**	**17<br>3%**	**43<br>7%**	**20<br>3%**	**46<br>8%**	**150<br>26%**	**147<br>25%**	**34<br>6%**	**8<br>1%**

Note: Number in each cell denotes the number of studies featuring the corresponding self-care category (row) and measuring the corresponding outcome (column). Percentages are based on total studies (N=580). For further details on the definition of each outcome category, see Supplementary File S5

Title page

Supplementary File S1: Preferred Reporting Items for Systematic reviews and Meta-Analyses extension for Scoping Reviews (PRISMA-ScR) Checklist 2

Supplementary File S2: Protocol as registered on Open Science Framework 4

Supplementary File S3: Search strategy for all databases 8

Supplementary File S4: Defining self-care interventions, a conceptual framework and examples for making judgements on specific interventions 30

Supplementary File S5: Data collection form 34

Supplementary File S6: List of excluded full texts 39

Supplementary File S7: Reference list of included studies 64

Supplementary File S8. Characteristics of studies by category 96

Supplementary File S9: Complex interventions and their characteristics 97

Supplementary File S10: List of all self-care interventions identified from included studies 100

Supplementary File S11: Categorising self-care interventions based on WHO's classification framework 107

For peer review only

For peer review only - http://bmjopen.bmj.com/site/about/guidelines.xhtml

Supplementary File S1. Preferred Reporting Items for Systematic reviews and Meta-Analyses extension for Scoping Reviews (PRISMA-ScR) Checklist

SECTION	ITEM	PRISMA-ScR CHECKLIST ITEM	REPORTED ON PAGE #
**TITLE**			
Title	1	Identify the report as a scoping review.	1
**ABSTRACT**			
Structured summary	2	Provide a structured summary that includes (as applicable): background, objectives, eligibility criteria, sources of evidence, charting methods, results, and conclusions that relate to the review questions and objectives.	2-3
**INTRODUCTION**			
Rationale	3	Describe the rationale for the review in the context of what is already known. Explain why the review questions/objectives lend themselves to a scoping review approach.	4
Objectives	4	Provide an explicit statement of the questions and objectives being addressed with reference to their key elements (e.g., population or participants, concepts, and context) or other relevant key elements used to conceptualize the review questions and/or objectives.	4
**METHODS**			
Protocol and registration	5	Indicate whether a review protocol exists; state if and where it can be accessed (e.g., a Web address); and if available, provide registration information, including the registration number.	5<br>Supp File S1
Eligibility criteria	6	Specify characteristics of the sources of evidence used as eligibility criteria (e.g., years considered, language, and publication status), and provide a rationale.	5-7<br>Supp File S3
Information sources*	7	Describe all information sources in the search (e.g., databases with dates of coverage and contact with authors to identify additional sources), as well as the date the most recent search was executed.	5<br>Supp File S2
Search	8	Present the full electronic search strategy for at least 1 database, including any limits used, such that it could be repeated.	Supp File S2
Selection of sources of evidence†	9	State the process for selecting sources of evidence (i.e., screening and eligibility) included in the scoping review.	6-7
Data charting process‡	10	Describe the methods of charting data from the included sources of evidence (e.g., calibrated forms or forms that have been tested by the team before their use, and whether data charting was done independently or in duplicate) and any processes for obtaining and confirming data from investigators.	6-7<br>Supp File S4
Data items	11	List and define all variables for which data were sought and any assumptions and simplifications made.	7<br>Supp File S4
Critical appraisal of individual sources of evidence§	12	If done, provide a rationale for conducting a critical appraisal of included sources of evidence; describe the methods used and how this information was used in any data synthesis (if appropriate).	N/A
Synthesis of results	13	Describe the methods of handling and summarizing the data that were charted.	7
**RESULTS**			
Selection of sources of evidence	14	Give numbers of sources of evidence screened, assessed for eligibility, and included in the review, with reasons for exclusions at each stage, ideally using a flow diagram.	8<br>Fig 1
Characteristics of sources of evidence	15	For each source of evidence, present characteristics for which data were charted and provide the citations.	N/A
Critical appraisal within sources of evidence	16	If done, present data on critical appraisal of included sources of evidence (see item 12).	N/A
Results of individual sources of evidence	17	For each included source of evidence, present the relevant data that were charted that relate to the review questions and objectives.	N/A
Synthesis of results	18	Summarize and/or present the charting results as they relate to the review questions and objectives.	8-12
**DISCUSSION**			
Summary of evidence	19	Summarize the main results (including an overview of concepts, themes, and types of evidence available), link to the review questions and objectives, and consider the relevance to key groups.	13-4

For peer review only - http://bmjopen.bmj.com/site/about/guidelines.xhtml

SECTION	ITEM	PRISMA-ScR CHECKLIST ITEM	REPORTED ON PAGE #
Limitations	20	Discuss the limitations of the scoping review process.	14-15
Conclusions	21	Provide a general interpretation of the results with respect to the review questions and objectives, as well as potential implications and/or next steps.	15
**FUNDING**			
Funding	22	Describe sources of funding for the included sources of evidence, as well as sources of funding for the scoping review. Describe the role of the funders of the scoping review.	16

From: Tricco AC, Lillie E, Zarin W, O'Brien KK, Colquhoun H, Levac D, et al. PRISMA Extension for Scoping Reviews (PRISMAScR): Checklist and Explanation. Ann Intern Med. 2018;169:467–473. doi: 10.7326/M18-0850.

For peer review only

Supplementary File S2. Protocol as registered on Open Science Framework

Title: Self-care interventions for antenatal, intrapartum and postnatal care: protocol for a scoping review

1. INTRODUCTION

Self-care is defined by the World Health Organization (WHO) as “the ability of individuals, families and communities to promote health, prevent disease, maintain health, and to cope with illness and disability with or without the support of a healthcare provider” (WHO, 2021). Self-care interventions are tools that support self-care, which include “evidence-based, high-quality drugs, devices, diagnostics and/or digital interventions that can be provided fully or partially outside formal health services and be used with or without a health worker” (WHO, 2021). Self-care interventions offer a solution to reduce health disparities and inequity in health access, especially in low-resource settings. By co-opting the pregnant woman and her family, self-care interventions can enhance the overall efficiency of healthcare delivery, while also improving service uptake and adherence to treatment.

There is a large and heterogeneous body of research on maternal and perinatal health interventions that are useful (or promising) from a self-care perspective. In this context, a scoping review can help identify established or emerging self-care interventions that could be considered for developing recommendations.

2. OBJECTIVES

- To identify self-care interventions in antenatal, intrapartum and postnatal care with sufficient evidence that may warrant a WHO recommendation
- To identify emerging self-care interventions that are promising research areas and should be prioritised
- To describe and classify these interventions by self-care concepts, behaviours, mode of delivery and other characteristics

3. METHODS

This protocol was adapted from the methodological guide for scoping reviews (Peters et al., 2015). The review will be reported using the PRISMA for Scoping Reviews extension (Tricco et al., 2018).

3.1. Electronic searches

We will systematically search Medline, Embase, Emcare and PsycINFO (via Ovid), CENTRAL and CDSR (via Cochrane Library) and CINAHL Plus (via EBSCOhost) to identify published studies from inception to current. We have developed a search strategy which combines various self-care concepts with terms related to pregnancy care, based on the framework proposed by Narasimhan et al. (2019). The terms and concepts used were informed by previous reviews of pregnancy care and self-care for other health conditions (e.g. chronic diseases). References of included articles will be manually screened to identify studies.

Table 1. Categories of self-care interventions

Self-management	Self-testing	Self-awareness

Self-medication	Self-sampling	Self-help
Self-treatment	Self-screening	Self-education
Self-examination	Self-diagnosis	Self-regulation
Self-injection	Self-collection	Self-efficacy
Self-use	Self-monitoring	Self-determination

2. Study selection

Two reviewers will independently screen titles and abstracts to remove irrelevant studies and then review full texts based on eligible criteria below. All stages of screening will be conducted in Covidence (Veritas Health Innovation, Australia). Any disagreement is resolved by discussion or adjudication to a third reviewer.

The eligibility criteria for inclusion are:

3.1 Participants: Women preparing to get pregnant, pregnant women, women giving birth, or women in the postnatal period (up to 42 days after birth), of any age. Studies examining interventions only directed at newborns will be excluded.

3.2 Interventions

For the purpose of this review, a self-care intervention is defined as any tool, resource or strategy designed to promote or facilitate self-care practices by the pregnant woman for the purpose of improving the quality or coverage of maternity health care, and/or improving the health, well-being and experiences of the woman and her baby.

A self-care practice include those that can be taken by a pregnant woman of her own accord towards the goals of (a) information-seeking and improving health literacy, (b) promoting mental health, self-efficacy and self-awareness, (c) increasing physical activity, (d) maintaining a healthy diet, (e) reducing risk and exposure avoidance, (d) good hygiene, and (7) safe and appropriate use of medical products and services.

This definition framework is summarised in Figure 1.

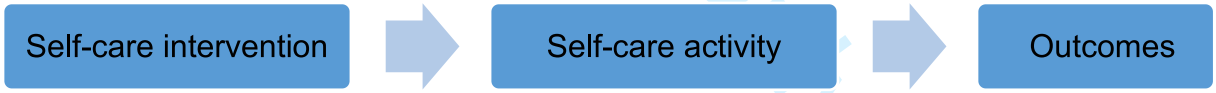

Annex 2 further elaborates on this conceptual framework and outlines the principles that guide the reviewers in judging whether an intervention is considered a self-care intervention.

3.3 Comparator: No intervention, placebo or standard/clinical care; single-arm interventional studies are eligible.

3.4 Outcomes: These interventions must be aimed at reducing risks of any pregnancy, childbirth or postnatal complications and/or enabling a positive pregnancy, childbirth or postnatal experience. The outcomes can be relating to physical, psychological or emotional health, behavioural change or product performance. Interventions measuring social, economic and financial outcomes are excluded from the scope of this review. Feasibility studies evaluating only retention rates and acceptability are excluded.

3.5 Types of studies:

We will include RCTs, quasi-experimental studies and cohort studies, as well as systematic reviews and meta-analyses for mapping. Reviews must state some forms of search strategy

For peer review only - http://bmjopen.bmj.com/site/about/guidelines.xhtml

and present all included studies in a systematic manner to be eligible. No date restriction will be applied. Case series, case studies, case reports, dissertations and theses, reports published as abstracts only, correspondence letters and editorials, qualitative studies will be excluded. Only studies with full texts written in English are eligible.

Quality assessment

In line with scoping review methodology (Peters et al., 2015), quality assessment will not be performed.

Data extraction and analysis

We will collect the following data on study characteristics (study design, country of origin, publication year), population characteristics (generic vs condition-specific, age, individual vs group, cultural/socioeconomic disadvantaged groups), intervention characteristics (provider, location, mode of delivery, period of pregnancy, routine vs condition-specific care, basic description), type of outcomes (physical, psychological or emotional). Interventions will be organised into themes, which are adapted from an earlier WHO's guideline on antenatal care (WHO, 2016) and will be further modified based on an iterative process of analysing retrieved interventions. The current proposed themes are as follows: (1) lifestyle adjustments, including nutritional interventions and physical activities; (2) interventions for emotional and spiritual wellbeing; (3) self-monitoring and assessment to aid decision-making; (4) preventive measures against pregnancy complications; and (5) interventions to alleviate common physiological symptoms.

Two reviewers will perform data extraction independently using a predefined template. Any disagreement is resolved via discussion or adjudication to a third reviewer. Given the rapid and scoping nature of this review, authors will not be contacted for missing data.

Results will be summarised as an evidence map accompanied by a narrative summary. Based on a 2D-intervention-outcome framework (Saran et al, 2018), the evidence map will visually display the type and quantity of evidence for each intervention, classified by outcome type, type of evidence, findings of evidence and sample size. The narrative summary will discuss the scope and strength of the evidence base. We will focus on identifying self-care interventions with high-quality evidence to be taken to scale, interventions that require further evidence synthesis with systematic reviews, and interventions that lack primary evidence.

References

Narasimhan, M., Allotey, P., & Hardon, A. (2019). Self care interventions to advance health and wellbeing: A conceptual framework to inform normative guidance. *BMJ (Online)*, *365*. https://doi.org/10.1136/bmj.l688

Peters, M. D. J., Godfrey, C. M., Khalil, H., McInerney, P., Parker, D., & Soares, C. B. (2015). Guidance for conducting systematic scoping reviews. *International Journal of Evidence-Based Healthcare*, *13*(3), 141–146. https://doi.org/10.1097/XEB.0000000000000050

Saran A, White H. Evidence and gap maps: a comparison of different approaches. Campbell Systematic Reviews. 2018 Jan;14(1):1-38.

Tricco, A. C., Lillie, E., Zarin, W., O'Brien, K. K., Colquhoun, H., Levac, D., Moher, D., Peters, M. D. J., Horsley, T., Weeks, L., Hempel, S., Akl, E. A., Chang, C., McGowan, J., Stewart, L., Hartling, L., Aldcroft, A., Wilson, M. G., Garritty, C., … Straus, S. E. (2018). PRISMA extension for scoping reviews (PRISMA-ScR): Checklist and explanation. In *Annals of Internal Medicine* (Vol.

For peer review only - http://bmjopen.bmj.com/site/about/guidelines.xhtml

169, Issue 7, pp. 467–473). American College of Physicians. https://doi.org/10.7326/M18-0850

WHO. (2016). *Recommendations on antenatal care for a positive pregnancy experience*. World Health Organization. https://www.who.int/publications-detail-redirect/9789241549912

WHO. (2019). *What do we mean by self-care?* World Health Organization. http://www.who.int/reproductivehealth/self-care-interventions/definitions/en/

WHO. (2021). *Guideline on Self-Care Interventions for Health and Well-Being*. World Health Organization. https://www.who.int/publications-detail-redirect/9789240030909

For peer review only

Supplementary File S3. Search strategy for all databases

Cochrane Library <October 17, 2021>

#1	(self next care):ti,ab,kw
#2	(selfcare):ti,ab,kw
#3	MeSH descriptor: [Self Care] explode all trees
#4	((home or "home-based" or homebased) NEAR/3 care):ti,ab,kw
#5	(self near/1 manag*):ti,ab,kw
#6	(selfmanag*):ti,ab,kw
#7	MeSH descriptor: [Self-Management] explode all trees
#8	((home or "home-based" or homebased) near/3 manag*):ti,ab,kw
#9	(self NEXT medicat*):ti,ab,kw
#10	(selfmedicat*):ti,ab,kw
#11	MeSH descriptor: [Self Medication] explode all trees
#12	((home or "home-based" or homebased) near/3 (medicat* or medicine)):ti,ab,kw
#13	(self NEXT treat*):ti,ab,kw
#14	(selftreat*):ti,ab,kw
#15	((home or "home-based" or homebased) near/3 treat*):ti,ab,kw
#16	(self NEXT examin*):ti,ab,kw
#17	(selfexamin*):ti,ab,kw
#18	MeSH descriptor: [Self-Examination] explode all trees
#19	((home or "home-based" or homebased) near/3 exam*):ti,ab,kw
#20	(self NEXT inject*):ti,ab,kw
#21	(selfinject*):ti,ab,kw
#22	((home or "home-based" or homebased) near/3 inject*):ti,ab,kw
#23	(self NEXT administ*):ti,ab,kw
#24	(selfadminist*):ti,ab,kw
#25	MeSH descriptor: [Self Administration] explode all trees
#26	((home or "home-based" or homebased) near/3 (administ* not administrator*)):ti,ab,kw
#27	(self NEXT (use or usage)):ti,ab,kw
#28	(selfuse or selfusage):ti,ab,kw
#29	(self NEXT test*):ti,ab,kw
#30	(selftest*):ti,ab,kw
#31	MeSH descriptor: [Self-Testing] explode all trees
#32	((home or "home-based" or homebased) near/3 (test* or kits or kit)):ti,ab,kw
#33	(self NEXT sampl*):ti,ab,kw
#34	(selfsampl*):ti,ab,kw
#35	((home or "home-based" or homebased) near/3 sampl*):ti,ab,kw
#36	(self NEXT screen*):ti,ab,kw
#37	(selfscreen*):ti,ab,kw
#38	((home or "home-based" or homebased) near/3 screen*):ti,ab,kw
#39	(self NEXT diagnos*):ti,ab,kw
#40	(selfdiagnos*):ti,ab,kw
#41	((home or "home-based" or homebased) near/3 diagnos*):ti,ab,kw
#42	(self NEXT collect*):ti,ab,kw
#43	(selfcollect*):ti,ab,kw
#44	((home or "home-based" or homebased) near/3 collect*):ti,ab,kw
#45	(self NEXT monitor*):ti,ab,kw
#46	(selfmonitor*):ti,ab,kw
#47	((home or "home-based" or homebased) near/3 monitor*):ti,ab,kw
#48	MeSH descriptor: [Blood Glucose Self-Monitoring] explode all trees
#49	(SMBG):ti,ab,kw

For peer review only - http://bmjopen.bmj.com/site/about/guidelines.xhtml

#50	(self NEXT measur*):ti,ab,kw
#51	(selfmeasur*):ti,ab,kw
#52	((home or "home-based" or homebased) near/3 measur*):ti,ab,kw
#53	(self NEXT assess*):ti,ab,kw
#54	(selfassess*):ti,ab,kw
#55	MeSH descriptor: [Self-Assessment] explode all trees
#56	((home or "home-based" or homebased) near/3 assess*):ti,ab,kw
#57	(self NEXT evaluat*):ti,ab,kw
#58	(selfevaluat*):ti,ab,kw
#59	MeSH descriptor: [Diagnostic Self Evaluation] explode all trees
#60	((home or "home-based" or homebased) near/3 evaluat*):ti,ab,kw
#61	(self NEXT aware*):ti,ab,kw
#62	(selfaware*):ti,ab,kw
#63	(self NEXT help*):ti,ab,kw
#64	(selfhelp*):ti,ab,kw
#65	MeSH descriptor: [Self-Help Devices] explode all trees
#66	(self NEXT educat*):ti,ab,kw
#67	(selfeducat*):ti,ab,kw
#68	(self NEXT regulat*):ti,ab,kw
#69	(selfregulat*):ti,ab,kw
#70	MeSH descriptor: [Self-Control] explode all trees
#71	((home or "home-based" or homebased) near/3 control):ti,ab,kw
#72	(self NEXT efficac*):ti,ab,kw
#73	(selfeffica*):ti,ab,kw
#74	MeSH descriptor: [Self Efficacy] explode all trees
#75	(self NEXT determin*):ti,ab,kw
#76	(selfdetermin*):ti,ab,kw
#77	MeSH descriptor: [Personal Autonomy] explode all trees
#78	MeSH descriptor: [Self Disclosure] explode all trees
#79	(self NEXT advoca*):ti,ab,kw
#80	(selfadvoca*):ti,ab,kw
#81	(self NEXT reliance):ti,ab,kw
#82	(selfreliance):ti,ab,kw
#83	(self-adjust* NEAR/3 (medication* or drug* or dosage* or doses or dosing)):ti,ab,kw
#84	(person NEXT cent* NEAR/3 care):ti,ab,kw
#85	(patient NEXT orient* NEAR/3 care):ti,ab,kw
#86	(patient NEXT cent* NEAR/3 care):ti,ab,kw
#87	MeSH descriptor: [Patient-Centered Care] explode all trees
#88	(family cent* NEAR/3 care):ti,ab,kw
#89	("non-pharmacological" or "non-pharmaceutical" or "non-clinical" or "non-medical"):ti,ab,kw
#90	((prevention or preventive) near/3 (infectious or infection*)):ti,kw
#91	(yeast or candida):ti,kw and (prevent* or reduc* or test* or diagnos*):ti,kw
#92	(postpartum NEAR/1 (anemia or anaemia)):ti,kw
#93	(hpv or papillo*):ti,kw and (prevent* or reduc*):ti,kw
#94	(cmv or cytomegalo*):ti,kw and (prevent* or reduc*):ti,kw
#95	(torch and prevent*):ti,kw
#96	(zika and (prevent* or reduc* or practices or strategies)):ti,kw
#97	(malaria:ti) and (prevent* or reduc* or practices or strategies):ti,kw
#98	((mosquito* or "vector-borne") and (prevent* or reduc* or practices or strategies)):ti,kw
#99	(sti or stis or std or stds or "sexually-transmitted"):ti,kw and (prevention or reduc* or practices or strategies):ti,kw
#100	#1 OR #2 OR #3 OR #4 OR #5 OR #6 OR #7 OR #8 OR #9 OR #10 OR #11 OR #12 OR #13 OR #14 OR #15 OR #16 OR #17 OR #18 OR #19 OR #20 OR #21 OR #22 OR #23 OR #24 OR #25 OR #26 OR #27

For peer review only - http://bmjopen.bmj.com/site/about/guidelines.xhtml

	OR #28 OR #29 OR #30 OR #31 OR #32 OR #33 OR #34 OR #35 OR #36 OR #37 OR #38 OR #39 OR #40 OR #41 OR #42 OR #43 OR #44 OR #45 OR #46 OR #47 OR #48 OR #49 OR #50 OR #51 OR #52 OR #53 OR #54 OR #55 OR #56 OR #57 OR #58 OR #59 OR #60 OR #61 OR #62 OR #63 OR #64 OR #65 OR #66 OR #67 OR #68 OR #69 OR #70 OR #71 OR #72 OR #73 OR #74 OR #75 OR #76 OR #77 OR #78 OR #79 OR #80 OR #81 OR #82 OR #83 OR #84 OR #85 OR #86 OR #87 OR #88 OR #89 OR #90 OR #91 OR #92 OR #93 OR #94 OR #95 OR #96 OR #97 OR #98 OR #99
#101	(antenatal or "ante-natal"):TI,KW
#102	(prenatal or "pre-natal"):TI,KW
#103	(perinatal or "peri-natal"):TI,KW
#104	(intrapartum or "intra-partum"):TI,KW
#105	(postpartum or "post-partum"):TI,KW
#106	(postnatal or "post-natal"):TI,KW
#107	(preconception or pre-conception):TI,KW
#108	("pregnancy care" or (pregnancy NEXT outcom*) or (pregnancy NEXT complication*)):TI,KW
#109	("maternal care" or (maternal NEXT outcom*) or (maternal NEXT complication*)):TI,KW
#110	("obstetric care" or (obstetric NEXT outcom*) or (obstetric NEXT complication*)):TI,KW
#111	(birth or childbirth):ti,kw
#112	(labor or labour):ti,kw
#113	(Nulligrav* or nullipar* or primigrav* or primipar* or multigrav* or multipar*):ti,kw
#114	pregnan*:ti,kw
#115	MeSH descriptor: [Pregnancy] explode all trees
#116	MeSH descriptor: [Pregnancy Outcome] explode all trees
#117	MeSH descriptor: [Pregnancy Complications] explode all trees
#118	MeSH descriptor: [Preconception Care] explode all trees
#119	MeSH descriptor: [Prenatal Care] explode all trees
#120	MeSH descriptor: [Perinatal Care] explode all trees
#121	MeSH descriptor: [Postnatal Care] explode all trees
#122	MeSH descriptor: [Prenatal Diagnosis] explode all trees
#123	MeSH descriptor: [Pregnancy Tests] explode all trees
#124	MeSH descriptor: [Prenatal Exposure Delayed Effects] explode all trees
#125	MeSH descriptor: [Prenatal Education] explode all trees
#126	MeSH descriptor: [Maternal Health Services] explode all trees
#127	MeSH descriptor: [Delivery, Obstetric] explode all trees
#128	#101 OR #102 OR #103 OR #104 OR #105 OR #106 OR #107 OR #108 OR #109 OR #110 OR #111 OR #112 OR #113 OR #114 OR #115 OR #116 OR #117 OR #118 OR #119 OR #120 OR #121 OR #122 OR #123 OR #124 OR #125 OR #126 R #127
#129	#100 AND #128
#130	(clinicaltrials.gov):so
#131	(www.who.int):so
#132	#129 not (#130 OR #131)

For peer review only - http://bmjopen.bmj.com/site/about/guidelines.xhtml

CINAHL Plus via EBSCOHost <1937 to October 17, 2021>

S1	TI "self care" OR SU "self care" OR TI selfcare OR SU selfcare
S2	TI (home or "home-based" or homebased) N3 care) OR SU (home or "home-based" or homebased) N3 care)
S3	(MH "Self Care+")
S4	TI "self manag*" OR SU "self manag*" OR TI selfmanag* OR SU selfmanag*
S5	TI (home or "home-based" or homebased) N3 manag*) OR SU (home or "home-based" or homebased) N3 manag*)
S6	(MH "Self-Management")
S7	TI "self medicat*" OR SU "self medicat*" OR TI selfmedicat* OR SU selfmedicat*
S8	TI (home or "home-based" or homebased) N3 (medicat* or medicine)) OR SU (home or "home-based" or homebased) N3 (medicat* or medicine))
S9	(MH "Self Medication")
S10	TI "self treat*" OR SU "self treat*" OR TI selftreat* OR SU selftreat*
S11	TI (home or "home-based" or homebased) N3 treat*) OR SU (home or "home-based" or homebased) N3 treat*)
S12	TI "self examin*" OR SU "self examin*" OR TI selfexamin* OR SU selfexamin*
S13	TI (home or "home-based" or homebased) N3 exam*) OR SU (home or "home-based" or homebased) N3 exam*)
S14	(MH "Vulvar Self-Examination")
S15	TI "self inject*" OR SU "self inject*" OR TI "selfinject*" OR SU "selfinject*"
S16	TI (home or "home-based" or homebased) N3 inject*) OR SU (home or "home-based" or homebased) N3 inject*)
S17	TI "self administ*" OR SU "self administ*" OR TI selfadminist* OR SU selfadminist*"
S18	TI (home or "home-based" or homebased) N3 (administ* not administrator*)) OR SU (home or "home-based" or homebased) N3 (administ* not administrator*))
S19	(MH "Self Administration+")
S20	TI ("self use" or "self usage") OR SU ("self use" or "self usage")
S21	TI (selfuse or selfusage) OR SU (selfuse or selfuage)
S22	TI "self test*" OR SU "self test*" OR TI selftest* OR SU selftest*
S23	TI (home or "home-based" or homebased) N3 (test* or kits or kit)) OR SU (home or "home-based" or homebased) N3 (test* or kits or kit))
S24	(MH "Self-Testing")
S25	TI "self sampl*" OR SU "self sampl*" OR TI selfsampl* OR SU selfsampl*
S26	TI (home or "home-based" or homebased) N3 sampl*) OR SU (home or "home-based" or homebased) N3 sampl*)
S27	TI "self screen*" OR SU "self screen*" OR TI selfscreen* OR SU selfscreen*
S28	TI (home or "home-based" or homebased) N3 screen*) OR SU (home or "home-based" or homebased) N3 screen*)
S29	TI "self diagnos*" OR SU "self diagnos*" OR TI selfdiagnos* OR SU selfdiagnos*
S30	TI (home or "home-based" or homebased) N3 diagnos*) OR SU (home or "home-based" or homebased) N3 diagnos*)
S31	(MH "Self-Diagnosis+")
S32	TI "self collect*" OR SU "self collect*" OR TI selfcollect* OR SU selfcollect*
S33	TI (home or "home-based" or homebased) N3 collect*) OR SU (home or "home-based" or homebased) N3 collect*)
S34	TI "self monitor*" OR SU "self monitor*" OR TI selfmonitor* OR SU selfmonitor*
S35	TI (home or "home-based" or homebased) N3 monitor*) OR SU (home or "home-based" or homebased) N3 monitor*)
S36	(MH "Blood Glucose Self-Monitoring")
S37	TI "self assess*" OR SU "self assess*" OR TI selfassess* OR SU selfassess*
S38	TI (home or "home-based" or homebased) N3 assess*) OR SU (home or "home-based" or homebased) N3 assess*)
S39	(MH "Self Assessment")
S40	TI "self evaluat*" OR SU "self evaluat*" OR TI "selfevaluat*" OR SU "selfevaluat*"

S41	TI (home or "home-based" or homebased) N3 evaluat*) OR SU (home or "home-based" or homebased) N3 evaluat*)
S42	TI "self aware*" OR SU "self aware*"
S43	TI selfaware* OR SU selfaware*
S44	(MH "Self-Awareness")
S45	TI "self help*" OR SU "self help*"
S46	TI selfhelp* OR SU selfhelp*
S47	TI "self educat*" OR SU "self educat*"
S48	TI selfeducat* OR SU selfeducat*
S49	TI "self regulat*" OR SU "self regulat*"
S50	TI selfregulat* OR SU selfregulat*
S51	(MH "Self Regulation+")
S52	TI "self-control" OR SU "self-control" OR TI selfcontrol OR SU selfcontrol
S53	TI (home or "home-based" or homebased) N3 control) OR SU (home or "home-based" or homebased) N3 control)
S54	TI "self efficac*" OR SU "self efficac*"
S55	TI selfeffica* OR SU selfeffica*
S56	TI "self determin*" OR SU "self determin*"
S57	TI selfdetermin* OR SU selfdetermin*
S58	(MH "Patient Autonomy")
S59	TI ("patient-cent*" N2 care) OR SU ("patient-cent*" N2 care)
S60	TI ("person-cent*" N2 care) OR SU ("person-cent*" N2 care)
S61	(MH "Breast Self-Examination")
S62	TI "patient-orient*" N2 care OR SU "patient-orient*" N2 care
S63	TI SMBG OR SU SMBG
S64	(MH "Self Disclosure")
S65	TI "selfrelian*" OR SU "selfrelian*"
S66	TI "self relian*" OR SU "self relian*"
S67	TI "self advoca*" OR SU "self advoca*"
S68	TI "selfadvoca*" OR SU "selfadvoca*"
S69	(TI (self-adjust* N3 (medication* or drug* or dosage* or doses or dosing))) OR AB ((self-adjust* N3 (medication* or drug* or dosage* or doses or dosing)))
S70	TI "self measur*" OR SU "self measur*" OR TI "selfmeasur*" OR SU "selfmeasur*"
S71	TI (home or "home-based" or homebased) N3 measur*) OR SU (home or "home-based" or homebased) N3 measur*)
S72	TI (("non-pharmacological" or "non-pharmaceutical" or "non-clinical" or "non-medical")) OR SU (("non-pharmacological" or "non-pharmaceutical" or "non-clinical" or "non-medical"))
S73	TI (((prevention or preventive) N3 (infectious or infection*)))
S74	TI ((yeast or candida) and (prevent* or reduc* or test* or diagnos*)) OR SU ((yeast or candida) and (prevent* or reduc* or test* or diagnos*))
S75	TI (postpartum N1 (anemia or anaemia)) OR SU (postpartum N1 (anemia or anaemia))
S76	TI (hpv or papillo*) and (prevent* or reduc*)) OR SU (hpv or papillo*). and (prevent* or reduc*))
S77	TI ((cmv or cytomegalo*) AND (prevent* or reduc*)) OR SU ((cmv or cytomegalo*) AND (prevent* or reduc*))
S78	TI (torch and prevent*) OR SU (torch and prevent*)
S79	TI (zika and (prevent* or reduc* or practices or strategies)) OR SU (zika and (prevent* or reduc* or practices or strategies))
S80	TI (malaria) and (TI (prevent* or reduc* or practices or strategies) OR SU (prevent* or reduc* or practices or strategies))
S81	TI ((mosquito* or "vector-borne") and (prevent* or reduc* or practices or strategies)) OR SU ((mosquito* or "vector-borne") and (prevent* or reduc* or practices or strategies))
S82	TI ((sti or stis or std or stds or "sexually-transmitted") and (prevention or reduc* or practices or strategies)) OR SU ((sti or stis or std or stds or "sexually-transmitted") and (prevention or reduc* or practices or strategies))
S83	S1 OR S2 OR S3 OR S4 OR S5 OR S6 OR S7 OR S8 OR S9 OR S10 OR S11 OR S12 OR S13 OR S14 OR S15 OR S16 OR S17 OR S18 OR S19 OR S20 OR S21 OR S22 OR S23 OR S24 OR S25 OR S26 OR

	S27 OR S28 OR S29 OR S30 OR S31 OR S32 OR S33 OR S34 OR S35 OR S36 OR S37 OR S38 OR S39 OR S40 OR S41 OR S42 OR S43 OR S44 OR S45 OR S46 OR S47 OR S48 OR S49 OR S50 OR S51 OR S52 OR S53 OR S54 OR S55 OR S56 OR S57 OR S58 OR S59 OR S60 OR S61 OR S62 OR S63 OR S64 OR S65 OR S66 OR S67 OR S68 OR S69 OR S70 OR S71 OR S72 OR S73 OR S74 OR S75 OR S76 OR S77 OR S78 OR S79 OR S80 OR S81 OR S82
S84	TI (antenatal or "ante-natal") OR SU (antenatal or "ante-natal")
S85	TI (prenatal or "pre-natal") OR SU (prenatal or "pre-natal")
S86	TI (perinatal or "peri-natal") OR SU (perinatal or "peri-natal")
S87	TI (intrapartum or "intra-partum") OR SU (intrapartum or "intra-partum")
S88	TI (postnatal or "post-natal") OR SU (postnatal or "post-natal")
S89	TI (postpartum or "post-partum") OR SU (postpartum or "post-partum")
S90	TI (preconception or "pre-conception") OR SU (preconception or "pre-conception")
S91	TI (pregnancy N1 (care or outcome* or complication*)) OR SU (pregnancy N1 (care or outcome* or complication*))
S92	TI (maternal N1 (care or outcome* or complication*)) OR SU (maternal N1 (care or outcome* or complication*))
S93	TI (obstetric N1 (care or outcome* or complication*)) OR SU (obstetric N1 (care or outcome* or complication*))
S94	TI (birth or childbirth) OR SU (birth or childbirth)
S95	TI (labor or labour) OR SU (labor or labour)
S96	TI (pregnan*) OR SU (pregnan*)
S97	(MH "Pregnancy Outcomes")
S98	(MH "Pregnancy Complications+")
S99	TI (Nulligrav* or nullipar* or primigrav* or primipar* or multigrav* or multipar*) OR SU (Nulligrav* or nullipar* or primigrav* or primipar* or multigrav* or multipar*)
S100	(MH "Postnatal Period+") OR (MH "Periconceptual Period")
S101	(MH "Pregnancy+") OR (MH "Pregnancy, Multiple") OR (MH "Pregnancy Trimesters+")
S102	(MH "Pregnancy Tests+")
S103	(MH "Prenatal Diagnosis+")
S104	(MH "Prenatal Exposure Delayed Effects") OR (MH "Prenatal Nutritional Physiology")
S105	(MH "Perinatal Risk (Saba CCC)+")
S106	(MH "Postpartum Care (Saba CCC)") OR (MH "Postnatal Care+")
S107	(MH "Prenatal Care") OR (MH "Prenatal Care (Iowa NIC)")
S108	(MH "Intrapartum Care+")
S109	(MH "Childbirth education")
S110	(MH "Maternal Nutritional Physiology+") OR (MH "Maternal Exposure")
S111	(MH "Maternal Health Services+")
S112	S84 OR S85 OR S86 OR S87 OR S88 OR S89 OR S90 OR S91 OR S92 OR S93 OR S94 OR S95 OR S96 OR S97 OR S98 OR S99 OR S100 OR S101 OR S102 OR S103 OR S104 OR S105 OR S106 OR S107 OR S108 OR S109 OR S110 OR S111
S113	(MH "Systematic Review") OR (MH "Meta Analysis")
S114	MH (random assignment) OR MH (cluster sample) OR MH (crossover design) OR MH (placebos)
S115	PT (Systematic review) or PT (clinical trial) OR PT (nursing interventions) OR PT (randomized controlled trial)
S116	(MH "Study Design+") OR (MH "Clinical Research+") OR (MH "Comparative Studies+") OR (MH "Epidemiological Research") OR (MH "Evaluation Research+") OR (MH "Multicenter Studies") OR (MH "Field Studies") OR (MH "Pilot Studies") OR (MH "Secondary Analysis")
S117	TI ((metaanalysis or "meta-analysis")) OR SU ((metaanalysis or "meta-analysis"))
S118	TI (((literature or systematic or quantitative or integrative) N2 (overview or review))) OR SU (((literature or systematic or quantitative or integrative) N2 (overview or review)))
S119	TI ((clinical or randomised or randomized or experimental or control or controlled or blind*) N10 (trial or study)) OR SU ((clinical or randomised or randomized or experimental or control or controlled or blind*) N10 (trial or study))
S120	TI (trial) AND (AB (randomised or randomized or (random* N3 allocat*) or placebo or control or controlled or blinded or blinding) OR SU (randomised or randomized or (random* N3 allocat*) or placebo or control or controlled or blinded or blinding))

For peer review only - http://bmjopen.bmj.com/site/about/guidelines.xhtml

S121	AB ((experiment* or intervention* or study or control or controlled or allocat*) N3 group*)
S122	TI ((pretest or pre-test or posttest or post-test or pre-post)) OR SU ((pretest or pre-test or posttest or post-test or pre-post))
S123	TI ((quasi-experiment* or quasiexperiment*)) OR SU ((quasi-experiment* or quasiexperiment*))
S124	TI (((prospective or retrospective or observational or longitudinal or observational or population or interventional) n4 (analysis or stud* or cohort))) OR SU (((prospective or retrospective or observational or longitudinal or observational or population or interventional) n4 (analysis or stud* or cohort)))
S125	TI (((cohort or evaluation or comparative or pilot) n3 (study or analysis))) OR SU (((cohort or evaluation or comparative or pilot) n3 (study or analysis)))
S126	TI cross-sectional OR SU cross-sectional
S127	TI mixed-methods OR SU mixed-methods
S128	S113 OR S114 OR S115 OR S116 OR S117 OR S118 OR S119 OR S120 OR S121 OR S122 OR S123 OR S124 OR S125 OR S126 OR S127
S129	(MH "Case Studies") OR (MH "Case Control Studies+")
S130	(MH "Qualitative Studies+") OR (MH "Time and Motion Studies+") OR (MH "Genome wide association study+")
S131	PT masters thesis or PT doctoral dissertation OR PT editorial OR PT commentary OR PT case study
S132	TI ("case stud*") or AB ("case stud*" N5 (method* or design))
S133	TI ("case series") or AB ("case series" N5 (method* or design))
S134	TI ("case control*") or AB ("case control*" N5 (method* or design))
S135	TI (editorial or comment or letter or conference or proceeding* or thesis or dissertation)
S136	(((MH "Animals+") OR (MH "Animal Studies") OR (TI "animal model*")) NOT (MH "human"))
S137	AB ((rat or rats or mice or mouse or animal) N3 (model or study or method* or design)) or TI (mice or mouse or rat or rats or "animal study")
S138	S129 OR S130 OR S131 OR S132 OR S133 OR S134 OR S135 OR S136 OR S137
S139	S83 and S112 and S128 NOT S138

For peer review only

Ovid MEDLINE(R) and Epub Ahead of Print, In-Process, In-Data-Review & Other Non-Indexed Citations, Daily and Versions(R) <1946 to October 17, 2021>

1	self-care.ti,kf.
2	selfcare.ti,kf.
3	exp self care/
4	((home or "home-based" or homebased) adj3 care).ti,kf.
5	self-manag*.ti,kf.
6	selfmanag*.ti,kf.
7	exp Self-Management/
8	((home or "home-based" or homebased) adj3 manag*).ti,kf.
9	self-medicat*.ti,kf.
10	selfmedicat*.ti,kf.
11	((home or "home-based" or homebased) adj3 (medicat* or medicine)).ti,kf.
12	exp self medication/
13	self-treat*.ti,kf.
14	selftreat*.ti,kf.
15	((home or "home-based" or homebased) adj3 treat*).ti,kf.
16	self-examin*.ti,kf.
17	selfexamin*.ti,kf.
18	exp self examination/
19	((home or "home-based" or homebased) adj3 exam*).ti,kf.
20	self-inject*.ti,kf.
21	selfinject*.ti,kf.
22	exp self injection/
23	((home or "home-based" or homebased) adj3 inject*).ti,kf.
24	self-administ*.ti,kf.
25	selfadminist*.ti,kf.
26	exp Self Administration/
27	((home or "home-based" or homebased) adj3 (administ* not administrator*)).ti,kf.
28	("self-use" or "self-usage").ti,kf.
29	(selfuse or selfusage).ti,kf.
30	self-test*.ti,kf.
31	selftest*.ti,kf.
32	exp Self-Testing/
33	((home or "home-based" or homebased) adj3 (test* or kits or kit)).ti,kf.
34	self-sampl*.ti,kf.
35	selfsampl*.ti,kf.
36	((home or "home-based" or homebased) adj3 sampl*).ti,kf.
37	self-screen*.ti,kf.
38	selfscreen*.ti,kf.
39	((home or "home-based" or homebased) adj3 screen*).ti,kf.
40	self-diagnos*.ti,kf.
41	selfdiagnos*.ti,kf.
42	((home or "home-based" or homebased) adj3 diagnos*).ti,kf.
43	self-collect*.ti,kf.
44	selfcollect*.ti,kf.
45	((home or "home-based" or homebased) adj3 collect*).ti,kf.
46	self-monitor*.ti,kf.
47	selfmonitor*.ti,kf.
48	((home or "home-based" or homebased) adj3 monitor*).ti,kf.
49	exp Blood Glucose Self-Monitoring/
50	SMBG.ti,kf.

51	self-measur*.ti,kf.
52	selfmeasu*.ti,kf.
53	((home or "home-based" or homebased) adj3 measur*).ti,kf.
54	self-control*.ti,kf.
55	selfcontrol.ti,kf.
56	exp self-control/
57	((home or "home-based" or homebased) adj3 control).ti,kf.
58	self-assess*.ti,kf.
59	selfassess.ti,kf.
60	exp Self-assessment/
61	((home or "home-based" or homebased) adj3 assess*).ti,kf.
62	self-evaluat*.ti,kf.
63	selfevaluat*.ti,kf.
64	exp self evaluation/
65	exp Diagnostic Self Evaluation/
66	((home or "home-based" or homebased) adj3 evaluat*).ti,kf.
67	Self-aware*.ti,kf.
68	selfaware*.ti,kf.
69	self-help*.ti,kf.
70	selfhelp*.ti,kf.
71	exp self help/
72	self-educat*.ti,kf.
73	selfeducat*.ti,kf.
74	self-regulat*.ti,kf.
75	selfregulat*.ti,kf.
76	exp self concept/
77	exp self disclosure/
78	self-effica*.ti,kf.
79	selfeffica*.ti,kf.
80	exp Self-efficacy/
81	self-determin*.ti,kf.
82	selfdetermin*.ti,kf.
83	self-reliance.ti,kf.
84	selfreliance.ti,kf.
85	self-advoc*.ti,kf.
86	selfadvoc*.ti,kf.
87	(self-adjust* adj3 (medication* or drug* or dosage* or doses or dosing)).ti,ab,kf.
88	(patient-cent* adj1 care).ti,kf.
89	(person-cent* adj1 care).ti,kf.
90	(patient-oriented adj1 care).ti,kf.
91	exp Patient-Centered Care/
92	(family-cent* adj1 care).ti,kf.
93	("non-pharmacological" or "non-pharmaceutical" or "non-clinical" or "non-medical").ti,kf.
94	((prevention or preventive) adj3 (infectious or infection*)).ti,kf.
94	(yeast or candida).ti,kf. and (prevent* or reduc* or test* or diagnos*).ti,kf.
95	(postpartum adj1 (anemia or anaemia)).ti,kf.
96	(hpv or papillo*).ti,kf. and (prevent* or reduc*).ti,kf.
97	(cmv or cytomegalo*).ti,kf. and (prevent* or reduc*).ti,kf.
98	torch.ti,kf. and prevent*.ti,ab,kf.
99	(zika and (prevent* or reduc* or practices or strategies)).ti,kf.
100	(malaria.ti.) and (prevent* or reduc* or practices or strategies).ti,kf.
101	((mosquito* or "vector-borne") and (prevent* or reduc* or practices or strategies)).ti,kf.

102	(sti or stis or std or stds or "sexually-transmitted").ti,kf. and (prevention or reduc* or practices or strategies).ti,kf.
103	or/1-102
104	antenatal.ti,kf.
105	(prenatal or pre-natal).ti,kf.
106	(perinatal or peri-natal).ti,kf.
107	(intrapartum or intra-partum).ti,kf.
108	(postpartum or post-partum).ti,kf.
109	(postnatal or post-natal).ti,kf.
110	(preconception or pre-conception).ti,kf.
111	("pregnancy care" or "pregnancy outcom*" or "pregnancy complication*").ti,kf.
112	("maternal care" or "maternal outcom*" or "maternal complication*").ti,kf.
113	("obstetric care" or "obstetric outcom*" or "obstetric complication*").ti,kf.
114	birth.ti,kf.
115	childbirth.ti,kf.
116	labour.ti,kf.
117	labor.ti,kf.
118	(Nulligrav* or nullipar* or primigrav* or primipar* or multigrav* or multipar*).ti,kf.
119	pregnan*.ti,kf.
120	exp pregnancy/
121	exp pregnancy outcome/
122	exp Pregnancy Complications/
123	exp Preconception care/
124	exp perinatal care/
125	exp prenatal care/
126	exp postnatal care/
127	exp pregnancy tests/
128	exp prenatal diagnosis/
129	exp Prenatal Exposure Delayed Effects/
130	exp prenatal education/
131	exp maternal health services/
132	exp Delivery, Obstetric/
133	or/104-132
134	exp Systematic Review/ or exp meta-analysis/
135	((literature or systematic or quantitative or integrative) adj2 (overview or review)).ti,ab,kf,sh.
136	(metaanalysis or meta-analysis).ti,ab,kf,sh.
137	exp Clinical trial/ or Clinical Study/ or exp Comparative Study/ or exp Evaluation Study/ or exp Multicenter Study/ or exp Observational Study/ or exp Evaluation Studies as Topic/
138	exp Epidemiologic Research Design/ not (Genome-Wide Association Study/ or exp Reproducibility of Results/)
139	exp Epidemiologic Study Characteristics/
140	((clinical or randomised or randomized or experimental or controlled or control or blind*) adj10 (trial or study)).ti,kf.
141	trial.ti. and (randomised or randomized or (random* adj3 allocat*) or placebo or control or controlled or blinded or blinding).ab,kf.
142	((experiment* or intervention* or study or control or controlled or allocat*) adj3 group*).ab.
143	(pretest or "pre-test" or posttest or "post-test" or "pre-post" or "quasi-experiment*" or quasiexperiment* or "cross-sectional" or "mixed-method*").ti,kf.
144	((prospective or retrospective or observational or longitudinal or observational or population or interventional) adj4 (analysis or stud* or cohort)).ti,kf.
145	((cohort or evaluation or comparative or pilot) adj3 (study or analysis)).ti,kf.
146	or/134-145
147	case study.ti. or ("case study" adj5 (method* or design)).ab.
148	case report.ti. or ("case report" adj5 (method* or design)).ab.

For peer review

For peer review only - http://bmjopen.bmj.com/site/about/guidelines.xhtml

149	case series.ti. or ("case series" adj5 (method* or design)).ab.
150	case control.ti. or ("case control" adj5 (method* or design)).ab.
151	(editorial or "conference abstract" or "conference review" or "conference paper" or "poster abstract*" or erratum or commentary or (letter adj3 editor)).ti.
152	(Clinical Conference or Editorial or Letter or Meeting Abstracts or Newspaper Article).pt.
153	exp Case-Control Studies/ or exp Case Reports/ or exp case Studies/ or exp Clinical conference/
154	(animals/ or exp animal experiment/) not (humans/ and animals/)
155	((rat or rats or mice or mouse or animal) adj3 (model or study or method* or design)).ab. or (mice or mouse or rat or rats or "animal study").ti.
156	or/147-155
157	(103 and 133 and 146) not 156

For peer review only

Embase Classic+Embase <1947 to October 17, 2021>

1	self-care.ti,kw.
2	selfcare.ti,kw.
3	exp self care/
4	((home or "home-based" or homebased) adj3 care).ti,kw.
5	self-manag*.ti,kw.
6	selfmanag*.ti,kw.
7	((home or "home-based" or homebased) adj3 manag*).ti,kw.
8	self-medicat*.ti,kw.
9	selfmedicat*.ti,kw.
10	exp self medication/
11	((home or "home-based" or homebased) adj3 (medicat* or medicine)).ti,kw.
12	self-treat*.ti,kw.
13	selftreat*.ti,kw.
14	((home or "home-based" or homebased) adj3 treat*).ti,kw.
15	self-examin*.ti,kw.
16	selfexamin*.ti,kw.
17	exp self examination/
18	((home or "home-based" or homebased) adj3 exam*).ti,kw.
19	self-inject*.ti,kw.
20	selfinject*.ti,kw.
21	exp self injection/
22	((home or "home-based" or homebased) adj3 inject*).ti,kw.
23	self-administ*.ti,kw.
24	selfadminist*.ti,kw.
25	exp drug self administration/
26	((home or "home-based" or homebased) adj3 (administ* not administrator*)).ti,kw.
27	("self-use" or "self usage").ti,kw.
28	(selfuse or selfusage).ti,kw.
29	self-test*.ti,kw.
30	selftest*.ti,kw.
31	((home or "home-based" or homebased) adj3 (test* or kits or kit)).ti,kw.
32	self-sampl*.ti,kw.
33	selfsampl*.ti,kw.
34	((home or "home-based" or homebased) adj3 sampl*).ti,kw.
35	self-screen*.ti,kw.
36	selfscreen*.ti,kw.
37	((home or "home-based" or homebased) adj3 screen*).ti,kw.
38	self-diagnos*.ti,kw.
39	selfdiagnos*.ti,kw.
40	((home or "home-based" or homebased) adj3 diagnos*).ti,kw.
41	self-collect*.ti,kw.
42	selfcollect*.ti,kw.
43	((home or "home-based" or homebased) adj3 collect*).ti,kw.
44	self-monitor*.ti,kw.
45	selfmonitor*.ti,kw.
46	exp self monitoring/
47	((home or "home-based" or homebased) adj3 monitor*).ti,kw.
48	*self monitoring of blood glucose/
49	*self monitoring of blood pressure/
50	SMBG.ti,kw.
51	self-measur*.ti,kw.

52	selfmeasu*.ti,kw.
53	((home or "home-based" or homebased) adj3 measur*).ti,kw.
54	self-control*.ti,kw.
55	selfcontrol.ti,kw.
56	exp self-control/
57	((home or "home-based" or homebased) adj3 control).ti,kw.
58	self-assess*.ti,kw.
59	selfassess.ti,kw.
60	((home or "home-based" or homebased) adj3 assess*).ti,kw.
61	self-evaluat*.ti,kw.
62	selfevaluat*.ti,kw.
63	exp self evaluation/
64	((home or "home-based" or homebased) adj3 evaluat*).ti,kw.
65	Self-aware*.ti,kw.
66	selfaware*.ti,kw.
67	self-help*.ti,kw.
68	selfhelp*.ti,kw.
69	exp self help/
70	self-educat*.ti,kw.
71	selfeducat*.ti,kw.
72	self-regulat*.ti,kw.
73	selfregulat*.ti,kw.
74	exp self esteem/
75	exp self disclosure/
76	self-effica*.ti,kw.
77	selfeffica*.ti,kw.
78	self-determin*.ti,kw.
79	selfdetermin*.ti,kw.
80	self-reliance.ti,kw.
81	selfreliance.ti,kw.
82	self-advoc*.ti,kw.
83	selfadvoc*.ti,kw.
84	(self-adjust* adj3 (medication* or drug* or dosage* or doses or dosing)).tw.
85	(patient-cent* adj1 care).ti,kw.
86	(person-cent* adj1 care).ti,kw.
87	(patient-oriented adj1 care).ti,kw.
88	(family-cent* adj1 care).ti,kw.
89	("non-pharmacological" or "non-pharmaceutical" or "non-clinical" or "non-medical").ti,kw.
90	((prevention or preventive) adj3 (infectious or infection*)).ti,kw.
91	(yeast or candida).ti,kw. and (prevent* or reduc* or test* or diagnos*).ti,kw.
92	(postpartum adj1 (anemia or anaemia)).ti,kw.
93	(hpv or papillo*).ti,kw. and (prevent* or reduc*).ti,kw.
94	(cmv or cytomegalo*).ti,kw. and (prevent* or reduc*).ti,kw.
95	torch.ti,kw. and prevent*.ti,ab,kw.
96	(zika and (prevent* or reduc* or practices or strategies)).ti,kw.
97	(malaria.ti.) and (prevent* or reduc* or practices or strategies).ti,kw.
98	((mosquito* or "vector-borne") and (prevent* or reduc* or practices or strategies)).ti,kw.
99	(sti or stis or std or stds or "sexually-transmitted").ti,kw. and (prevention or reduc* or practices or strategies).ti,kw.
100	or/1-99
101	(preconception or pre-conception).ti,kw.
102	antenatal.ti,kw.
103	(prenatal or pre-natal).ti,kw.

For peer review only - http://bmjopen.bmj.com/site/about/guidelines.xhtml

104	(perinatal or peri-natal).ti,kw.
105	(intrapartum or intra-partum).ti,kw.
106	(postpartum or post-partum).ti,kw.
107	(postnatal or post-natal).ti,kw.
108	("pregnancy care" or "pregnancy outcom*" or "pregnancy complication*").ti,kw.
109	("maternal care" or "maternal outcom*" or "maternal complication*").ti,kw.
110	("obstetric care" or "obstetric outcom*" or "obstetric complication*").ti,kw.
111	birth.ti,kw.
112	childbirth.ti,kw.
113	labour.ti,kw.
114	labor.ti,kw.
115	(Nulligrav* or nullipar* or primigrav* or primipar* or multigrav* or multipar*).ti,kw.
116	pregnan*.ti,kw.
117	exp pregnancy/
118	exp pregnancy complication/
119	exp pregnancy outcome/
120	exp obstetric procedure/
121	exp maternal health services/
122	exp prenatal exposure/
123	exp childbirth education/
124	or/101-123
125	exp "systematic review"/ or exp meta analysis/ or "systematic review (topic)"/ or "meta analysis (topic)"/
126	(review or meta-analysis).pt.
127	(metaanalysis or meta-analysis).ti,ab,kw,sh.
128	((literature or systematic or quantitative or integrative) adj2 (overview or review)).ti,ab,kw,sh.
129	exp clinical study/ or exp comparative study/ or exp experimental study/ or exp feasibility study/ or exp field study/ or exp observational study/ or exp quasi experimental study/ or exp pilot study/ or exp experimental study/ or exp comparative study/
130	clinical trial (topic)/ or parallel design/ or crossover procedure/ or triple blind procedure/ or double blind procedure/ or single blind procedure/
131	exp methodology/ and (randomization/ or placebo/)
132	control group/ or nonequivalent control group/
133	intention to treat analysis/ or secondary analysis/
134	pretest posttest control group design/ or experimental design/ or quantitative study/ or participatory research/
135	cohort analysis/ or correlational study/ or cross-sectional study/
136	((clinical or randomised or randomized or experimental or controlled or control or blind*) adj10 (trial or study)).ti,kw.
137	trial.ti. and (randomised or randomized or (random* adj3 allocat*) or placebo or control or controlled or blinded or blinding).ab,kw.
138	((experiment* or intervention* or study or control or controlled or allocat*) adj3 group*).ab.
139	((prospective or retrospective or observational or longitudinal or observational or population or interventional) adj4 (analysis or stud* or cohort)).ti,kw.
140	((cohort or evaluation or comparative or pilot) adj3 (study or analysis)).ti,kw.
141	(pretest or "pre-test" or posttest or "post-test" or "pre-post" or "quasi-experiment*" or quasiexperiment* or "cross-sectional" or "mixed-method*").ti,kw.
142	or/125-141
143	exp case control study/ or exp case report/ or exp case study/
144	case study.ti. or ("case study" adj5 (method* or design)).ab.
145	case report.ti. or ("case report" adj5 (method* or design)).ab.
146	case series.ti. or ("case series" adj5 (method* or design)).ab.
147	case control.ti. or ("case control" adj5 (method* or design)).ab.
148	(editorial or conference or erratum or letter or note).pt.
149	(editorial or "conference abstract" or "conference review" or "conference paper" or "poster abstract*" or erratum or commentary or (letter adj3 editor)).ti.

For peer review only - http://bmjopen.bmj.com/site/about/guidelines.xhtml

150	(animals/ or exp animal experiment/) not (humans/ and animals/)
151	((rat or rats or mice or mouse or animal) adj3 (model or study or method* or design)).ab. or (mice or mouse or rat or rats or "animal study").ti.
152	or/143-151
153	(100 and 124 and 142) not 152

For peer review only

For peer review only - http://bmjopen.bmj.com/site/about/guidelines.xhtml

Ovid Emcare <1995 to 2021 Week 41>

1	self-care.ti,kw.
2	selfcare.ti,kw.
3	exp self care/
4	((home or "home-based" or homebased) adj3 care).ti,kw.
5	self-manag*.ti,kw.
6	selfmanag*.ti,kw.
7	((home or "home-based" or homebased) adj3 manag*).ti,kw.
8	self-medicat*.ti,kw.
9	selfmedicat*.ti,kw.
10	exp self medication/
11	((home or "home-based" or homebased) adj3 (medicat* or medicine)).ti,kw.
12	self-treat*.ti,kw.
13	selftreat*.ti,kw.
14	((home or "home-based" or homebased) adj3 treat*).ti,kw.
15	self-examin*.ti,kw.
16	selfexamin*.ti,kw.
17	exp self examination/
18	((home or "home-based" or homebased) adj3 exam*).ti,kw.
19	self-inject*.ti,kw.
20	selfinject*.ti,kw.
21	exp self injection/
22	((home or "home-based" or homebased) adj3 inject*).ti,kw.
23	self-administ*.ti,kw.
24	selfadminist*.ti,kw.
25	exp drug self administration/
26	((home or "home-based" or homebased) adj3 (administ* not administrator*)).ti,kw.
27	("self-use" or "self usage").ti,kw.
28	(selfuse or selfusage).ti,kw.
29	self-test*.ti,kw.
30	selftest*.ti,kw.
31	((home or "home-based" or homebased) adj3 (test* or kits or kit)).ti,kw.
32	self-sampl*.ti,kw.
33	selfsampl*.ti,kw.
34	((home or "home-based" or homebased) adj3 sampl*).ti,kw.
35	self-screen*.ti,kw.
36	selfscreen*.ti,kw.
37	((home or "home-based" or homebased) adj3 screen*).ti,kw.
38	self-diagnos*.ti,kw.
39	selfdiagnos*.ti,kw.
40	((home or "home-based" or homebased) adj3 diagnos*).ti,kw.
41	self-collect*.ti,kw.
42	selfcollect*.ti,kw.
43	((home or "home-based" or homebased) adj3 collect*).ti,kw.
44	self-monitor*.ti,kw.
45	selfmonitor*.ti,kw.
46	exp self monitoring/
47	((home or "home-based" or homebased) adj3 monitor*).ti,kw.
48	*self monitoring of blood glucose/
49	*self monitoring of blood pressure/
50	SMBG.ti,kw.
51	self-measur*.ti,kw.

For peer review only - http://bmjopen.bmj.com/site/about/guidelines.xhtml

52	selfmeasu*.ti,kw.
53	((home or "home-based" or homebased) adj3 measur*).ti,kw.
54	self-control*.ti,kw.
55	selfcontrol.ti,kw.
56	exp self-control/
57	((home or "home-based" or homebased) adj3 control).ti,kw.
58	self-assess*.ti,kw.
59	selfassess.ti,kw.
60	((home or "home-based" or homebased) adj3 assess*).ti,kw.
61	self-evaluat*.ti,kw.
62	selfevaluat*.ti,kw.
63	exp self evaluation/
64	((home or "home-based" or homebased) adj3 evaluat*).ti,kw.
65	Self-aware*.ti,kw.
66	selfaware*.ti,kw.
67	self-help*.ti,kw.
68	selfhelp*.ti,kw.
69	exp self help/
70	self-educat*.ti,kw.
71	selfeducat*.ti,kw.
72	self-regulat*.ti,kw.
73	selfregulat*.ti,kw.
74	exp self esteem/
75	exp self disclosure/
76	self-effica*.ti,kw.
77	selfeffica*.ti,kw.
78	self-determin*.ti,kw.
79	selfdetermin*.ti,kw.
80	self-reliance.ti,kw.
81	selfreliance.ti,kw.
82	self-advoc*.ti,kw.
83	selfadvoc*.ti,kw.
84	(self-adjust* adj3 (medication* or drug* or dosage* or doses or dosing)).tw.
85	(patient-cent* adj1 care).ti,kw.
86	(person-cent* adj1 care).ti,kw.
87	(patient-oriented adj1 care).ti,kw.
88	(family-cent* adj1 care).ti,kw.
89	("non-pharmacological" or "non-pharmaceutical" or "non-clinical" or "non-medical").ti,kw.
90	((prevention or preventive) adj3 (infectious or infection*)).ti,kw.
91	(yeast or candida).ti,kw. and (prevent* or reduc* or test* or diagnos*).ti,kw.
92	(postpartum adj1 (anemia or anaemia)).ti,kw.
93	(hpv or papillo*).ti,kw. and (prevent* or reduc*).ti,kw.
94	(cmv or cytomegalo*).ti,kw. and (prevent* or reduc*).ti,kw.
95	torch.ti,kw. and prevent*.ti,ab,kw.
96	(zika and (prevent* or reduc* or practices or strategies)).ti,kw.
97	(malaria.ti.) and (prevent* or reduc* or practices or strategies).ti,kw.
98	((mosquito* or "vector-borne") and (prevent* or reduc* or practices or strategies)).ti,kw.
99	(sti or stis or std or stds or "sexually-transmitted").ti,kw. and (prevention or reduc* or practices or strategies).ti,kw.
100	or/1-99
101	(preconception or pre-conception).ti,kw.
102	antenatal.ti,kw.
103	(prenatal or pre-natal).ti,kw.

For peer review only - http://bmjopen.bmj.com/site/about/guidelines.xhtml

104	(perinatal or peri-natal).ti,kw.
105	(intrapartum or intra-partum).ti,kw.
106	(postpartum or post-partum).ti,kw.
107	(postnatal or post-natal).ti,kw.
108	("pregnancy care" or "pregnancy outcom*" or "pregnancy complication*").ti,kw.
109	("maternal care" or "maternal outcom*" or "maternal complication*").ti,kw.
110	("obstetric care" or "obstetric outcom*" or "obstetric complication*").ti,kw.
111	birth.ti,kw.
112	childbirth.ti,kw.
113	labour.ti,kw.
114	labor.ti,kw.
115	(Nulligrav* or nullipar* or primigrav* or primipar* or multigrav* or multipar*).ti,kw.
116	pregnan*.ti,kw.
117	exp pregnancy/
118	exp pregnancy complication/
119	exp pregnancy outcome/
120	exp obstetric procedure/
121	exp maternal health services/
122	exp prenatal exposure/
123	exp childbirth education/
124	or/101-123
125	exp "systematic review"/ or exp meta analysis/ or "systematic review (topic)"/ or "meta analysis (topic)"/
126	(review or meta-analysis).pt.
127	(metaanalysis or meta-analysis).ti,ab,kw,sh.
128	((literature or systematic or quantitative or integrative) adj2 (overview or review)).ti,ab,kw,sh.
129	exp clinical study/ or exp comparative study/ or exp experimental study/ or exp feasibility study/ or exp field study/ or exp observational study/ or exp quasi experimental study/ or exp pilot study/ or exp experimental study/ or exp comparative study/
130	clinical trial (topic)/ or parallel design/ or crossover procedure/ or triple blind procedure/ or double blind procedure/ or single blind procedure/
131	exp methodology/ and (randomization/ or placebo/)
132	control group/ or nonequivalent control group/
133	intention to treat analysis/ or secondary analysis/
134	pretest posttest control group design/ or experimental design/ or quantitative study/ or participatory research/
135	cohort analysis/ or correlational study/ or cross-sectional study/
136	((clinical or randomised or randomized or experimental or controlled or control or blind*) adj10 (trial or study)).ti,kw.
137	trial.ti. and (randomised or randomized or (random* adj3 allocat*) or placebo or control or controlled or blinded or blinding).ab,kw.
138	((experiment* or intervention* or study or control or controlled or allocat*) adj3 group*).ab.
139	((prospective or retrospective or observational or longitudinal or observational or population or interventional) adj4 (analysis or stud* or cohort)).ti,kw.
140	((cohort or evaluation or comparative or pilot) adj3 (study or analysis)).ti,kw.
141	(pretest or "pre-test" or posttest or "post-test" or "pre-post" or "quasi-experiment*" or quasiexperiment* or "cross-sectional" or "mixed-method*").ti,kw.
142	or/125-141
143	exp case control study/ or exp case report/ or exp case study/
144	case study.ti. or ("case study" adj5 (method* or design)).ab.
145	case report.ti. or ("case report" adj5 (method* or design)).ab.
146	case series.ti. or ("case series" adj5 (method* or design)).ab.
147	case control.ti. or ("case control" adj5 (method* or design)).ab.
148	(editorial or conference or erratum or letter or note).pt.
149	(editorial or "conference abstract" or "conference review" or "conference paper" or "poster abstract*" or erratum or commentary or (letter adj3 editor)).ti.

For peer review only - http://bmjopen.bmj.com/site/about/guidelines.xhtml

150	(animals/ or exp animal experiment/) not (humans/ and animals/)
151	((rat or rats or mice or mouse or animal) adj3 (model or study or method* or design)).ab. or (mice or mouse or rat or rats or "animal study").ti.
152	or/143-151
153	(100 and 124 and 142) not 152

For peer review only

For peer review only - http://bmjopen.bmj.com/site/about/guidelines.xhtml

Ovid APA PsycInfo <1806 to October Week 2 2021>

1	self-care.ti,id.
2	selfcare.ti,id.
3	exp self care/
4	((home or "home-based" or homebased) adj3 care).ti,id.
5	self-manag*.ti,id.
6	selfmanag*.ti,id.
7	exp Self-Management/
8	((home or "home-based" or homebased) adj3 manag*).ti,id.
9	self-medicat*.ti,id.
10	selfmedicat*.ti,id.
11	exp self-medication/
12	((home or "home-based" or homebased) adj3 (medicat* or medicine)).ti,id.
13	self-treat*.ti,id.
14	selftreat*.ti,id.
15	((home or "home-based" or homebased) adj3 treat*).ti,id.
16	self-examin*.ti,id.
17	selfexamin*.ti,id.
18	exp self examination/
19	exp "Self-Examination (Medical)"/
20	((home or "home-based" or homebased) adj3 exam*).ti,id.
21	self-inject*.ti,id.
22	selfinject*.ti,id.
23	((home or "home-based" or homebased) adj3 inject*).ti,id.
24	self-administ*.ti,id.
25	selfadminist*.ti,id.
26	exp drug self administration/
27	((home or "home-based" or homebased) adj3 (administ* not administrator*)).ti,id.
28	("self-use" or "self-usage").ti,id.
29	(selfuse or selfusage).ti,id.
30	self-test*.ti,id.
31	selftest*.ti,id.
32	((home or "home-based" or homebased) adj3 (test* or kits or kit)).ti,id.
33	self-sampl*.ti,id.
34	selfsampl*.ti,id.
35	((home or "home-based" or homebased) adj3 sampl*).ti,id.
36	self-screen*.ti,id.
37	selfscreen*.ti,id.
38	((home or "home-based" or homebased) adj3 screen*).ti,id.
39	self-diagnos*.ti,id.
40	selfdiagnos*.ti,id.
41	((home or "home-based" or homebased) adj3 diagnos*).ti,id.
42	self-collect*.ti,id.
43	selfcollect*.ti,id.
44	((home or "home-based" or homebased) adj3 collect*).ti,id.
45	self-monitor*.ti,id.
46	selfmonitor*.ti,id.
47	exp self-monitoring/
48	((home or "home-based" or homebased) adj3 monitor*).ti,id.
49	SMBG.ti,id.
50	self-measur*.ti,id.
51	selfmeasu*.ti,id.

For peer review only - http://bmjopen.bmj.com/site/about/guidelines.xhtml

52	((home or "home-based" or homebased) adj3 measur*).ti,id.
53	self-control*.ti,id.
54	selfcontrol.ti,id.
55	exp self-control/
56	((home or "home-based" or homebased) adj3 control).ti,id.
57	self-assess*.ti,id.
58	selfassess.ti,id.
59	((home or "home-based" or homebased) adj3 assess*).ti,id.
60	self-evaluat*.ti,id.
61	selfevaluat*.ti,id.
62	exp self-evaluation/
63	((home or "home-based" or homebased) adj3 evaluat*).ti,id.
64	Self-aware*.ti,id.
65	selfaware*.ti,id.
66	self-perception/
67	self-help*.ti,id.
68	selfhelp*.ti,id.
69	exp self help/
70	self-educat*.ti,id.
71	selfeducat*.ti,id.
72	exp self-knowledge/
73	self-regulat*.ti,id.
74	selfregulat*.ti,id.
75	exp self-regulation/
76	exp emotional control/
77	exp emotional regulation/
78	exp emotional adjustment/
79	exp self esteem/
80	exp self disclosure/
81	self-effica*.ti,id.
82	selfeffica*.ti,id.
83	exp self-efficacy/
84	self-determin*.ti,id.
85	selfdetermin*.ti,id.
86	self-reliance.ti,id.
87	selfreliance.ti,id.
88	self-advoc*.ti,id.
89	selfadvoc*.ti,id.
90	exp self-advocacy/
91	(self-adjust* adj3 (medication* or drug* or dosage* or doses or dosing)).ti,id.
92	(patient-cent* adj1 care).ti,id.
93	(person-cent* adj1 care).ti,id.
94	(patient-oriented adj1 care).ti,id.
95	exp Patient Centered Care/
96	(family-cent* adj1 care).ti,id.
97	("non-pharmacological" or "non-pharmaceutical" or "non-clinical" or "non-medical").ti,id.
98	((prevention or preventive) adj3 (infectious or infection*)).ti,id.
99	(yeast or candida).ti,id. and (prevent* or reduc* or test* or diagnos*).ti,id.
100	(postpartum adj1 (anemia or anaemia)).ti,id.
101	(hpv or papillo*).ti,id. and (prevent* or reduc*).ti,id.
102	(cmv or cytomegalo*).ti,id. and (prevent* or reduc*).ti,id.
103	torch.ti,id. and prevent*.ti,ab,id.

For peer review only - http://bmjopen.bmj.com/site/about/guidelines.xhtml

104	(zika and (prevent* or reduc* or practices or strategies)).ti,id.
105	(malaria.ti.) and (prevent* or reduc* or practices or strategies).ti,id.
106	((mosquito* or "vector-borne") and (prevent* or reduc* or practices or strategies)).ti,id.
107	(sti or stis or std or stds or "sexually-transmitted").ti,id. and (prevention or reduc* or practices or strategies).ti,id.
108	or/1-107
109	antenatal.ti,id.
110	(prenatal or pre-natal).ti,id.
111	(perinatal or peri-natal).ti,id.
112	(intrapartum or intra-partum).ti,id.
113	(postpartum or post-partum).ti,id.
114	(postnatal or post-natal).ti,id.
115	(preconception or pre-conception).ti,id.
116	("pregnancy care" or "pregnancy outcom*" or "pregnancy complication*").ti,id.
117	("maternal care" or "maternal outcom*" or "maternal complication*").ti,id.
118	("obstetric care" or "obstetric outcom*" or "obstetric complication*").ti,id.
119	pregnan*.ti,id.
120	(childbirth or birth).ti,id.
121	(labor or labour).ti,id.
122	(Nulligrav* or nullipar* or primigrav* or primipar* or multigrav* or multipar*).ti,id.
123	exp pregnancy/
124	exp prenatal care/ or exp prenatal diagnosis/ or exp prenatal exposure/
125	exp perinatal period/ or exp antepartum period/ or exp intrapartum period exp postnatal period/ or exp perinatal period/
126	or/109-125
127	exp Systematic Review/ or exp meta-analysis/
128	((literature or systematic or quantitative or integrative) adj2 (overview or review)).ti,ab,id,sh.
129	(metaanalysis or meta-analysis).ti,ab,id,sh.
130	exp experimental design/ not exp single-case experimental design/
131	exp cohort analysis/ or exp mixed methods research/ or exp quantitative methods/ or exp quasi-experimental methods/
132	((clinical or randomised or randomized or experimental or controlled or control or blind*) adj10 (trial or study)).ti,id.
133	trial.ti. and (randomised or randomized or (random* adj3 allocat*) or placebo or control or controlled or blinded or blinding).ab,id.
134	((experiment* or intervention* or study or control or controlled or allocat*) adj3 group*).ab.
135	(pretest or "pre-test" or posttest or "post-test" or "pre-post" or "quasi-experiment*" or quasiexperiment* or "cross-sectional" or "mixed-method*").ti,id.
136	((prospective or retrospective or observational or longitudinal or observational or population or interventional) adj4 (analysis or stud* or cohort)).ti,id.
137	((cohort or evaluation or comparative or pilot) adj3 (study or analysis)).ti,id.
138	or/127-137
139	(editorial or "newspaper article" or conference* or commentary).ti.
140	case study.ti. or ("case study" adj5 (method* or design)).ab.
141	case report.ti. or ("case report" adj5 (method* or design)).ab.
142	case series.ti. or ("case series" adj5 (method* or design)).ab.
143	case control.ti. or ("case control" adj5 (method* or design)).ab.
144	exp Case Report/ or exp Case-control studies/ or exp cross-sectional studies/
145	(animals/ or exp animal experiment/) not (humans/ and animals/)
146	((rat or rats or mice or mouse or animal) adj3 (model or study or method* or design)).ab. or (mice or mouse or rat or rats or "animal study").ti.
147	or/139-146
148	(108 and 126 and 138) not 147

For peer review only - http://bmjopen.bmj.com/site/about/guidelines.xhtml

Supplementary File S4. Defining self-care interventions, a conceptual framework and examples for making judgements on specific interventions

Definitions of self-care in WHO Guideline on Self-care Interventions for Health and Wellbeing (2021)

Self-care is the ability of individuals, families and communities to promote health, prevent disease, maintain health, and cope with illness and disability with or without the support of a health worker.

Self-care recognizes individuals as active agents in managing their own health care, in areas including health promotion; disease prevention and control; self-medication; providing care to dependent persons, and rehabilitation, including palliative care.

Self-care interventions are evidence-based, quality tools that support self-care. They include medicines, counselling, diagnostics and/or digital technologies which can be accessed fully or partially outside of formal health services. Depending on the intervention, they can be used with or without the direct supervision of health workers.

Working definition of self-care in this review

For the purpose of this review, a *self-care intervention* is defined as any tool, resource or strategy designed to promote or facilitate self-care by women preparing to get pregnant, currently pregnant, labouring/birthing or in the postpartum period (up to 42 days after birth), for the purpose of improving the quality or coverage of maternity health care, and/or improving the health, well-being and experiences of the pregnant woman and her newborn.

A *self-care activity* includes those that can be taken by a preconceiving, pregnant, labouring/birthing or postpartum woman of her own accord towards the goals of (a) information-seeking and improving health literacy, (b) promoting mental health, self-efficacy and self-awareness, (c) increasing physical activity, (d) maintaining a healthy diet, (e) reducing risk and exposure avoidance, (d) good hygiene, and (7) safe and appropriate use of products, services, diagnostics and medicines.

This definition framework is summarised as:

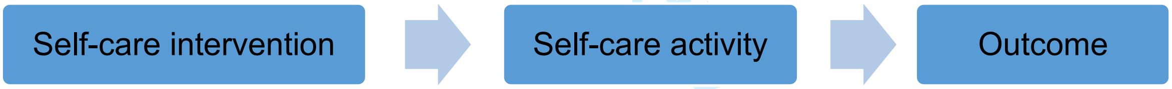

General principles

The following text considers several components of interventions that may have bearing on whether an intervention could be considered a self-care intervention or not.

1. Place of access

The self-care intervention might take place partially or fully outside formal health settings (e.g. outpatient services, clinics and hospitals). These places of access include, but are not limited to pharmacies, home, school and/or digital platforms.

2. Intersection with health systems

The intervention can be entirely initiated by the woman, or may involve some element of health worker (HW) support or engagement. In all instances, the woman must have the autonomy of her own self-care independent of a HW's presence.

(a) If the intervention relates to an informed action that can only be undertaken by the preconceiving, pregnant, labouring/birthing or postpartum woman (e.g. physical activity, daily dietary intake, etc.), it is considered self-care. Such an intervention may utilise

For peer review only - http://bmjopen.bmj.com/site/about/guidelines.xhtml

measures delivered by HW to facilitate or support the individual's self-care activity, for example by providing information, skills or tools.

> Example: Smoking cessation
>
> HW-led interventions aimed at promoting self-help for smoking cessation were eligible. These include HWs providing self-help materials (brochures, videos, online sources) or conducting educational sessions to give self-help instructions so that the woman can initiate smoking cessation at home.
>
> Interventions requiring intensive and regular contacts with physicians, therapists or nurses and without a fixed number of sessions were not considered self-care interventions. Interventions solely focusing on motivation enhancing e.g. motivational interviewing are not considered self-care interventions as these are not designed to develop skills and self-efficacy.

> Example: Acupuncture, transcutaneous electrical nerve stimulation (TENS), acupressure and massage
>
> Acupuncture is eligible if women are provided with and trained to operate a suitable device. TENS is eligible as TENS machines are easy to operate without prior training. Acupressure and massage, which do not require specific devices, are also eligible if administered by the woman or her partner.

> Example: Pain relief during labour
>
> Non-pharmacological therapies (e.g. aromatherapy, body massage, showering or water baths) are considered self-care if taught to the pregnant women or their caregivers during birth preparation workshops, or instructed to carry out at home, prior to admission. Studies where these interventions were administered by birth attendants or clinic staff are not eligible.

(b) If the intervention relates to care usually provided within the health system (e.g. testing, prescribing or administering medications), only those interventions where the woman self-manages some or all aspects of such care were considered as self-care. In studies, these interventions were usually compared against standard clinical care i.e. care delivered by physicians, nurses or community health workers.

> Example: Misoprostol for postpartum haemorrhage (PPH) prevention
>
> The administration of misoprostol by HWs (whether in facility or community settings) for PPH prevention is received by the pregnant woman - she does not self-administer. Therefore, it is not a self-care intervention, even if misoprostol administration takes place in the context of birth at home or in the community.
>
> Conversely, advanced distribution of misoprostol during antenatal visits to be used after childbirth, often accompanied by brief advice on how and when to use misoprostol safely, is considered a self-care intervention. This is because the intervention aims to enable the mother or her partners to self-administer misoprostol at home, when a skilled birth attendant, CHW or lay health worker is not available.

(c) Studies that investigated pharmacokinetics, efficacy and safety of pharmacological agents were excluded because their findings did not allow distinguishing between self-medication and being prescribed the medication by health providers. On the other hand, interventions aimed at promoting self-management or improving adherence to multi-dose therapies at home were eligible.

For peer review only - http://bmjopen.bmj.com/site/about/guidelines.xhtml

Example: Prophylactic agents for malaria and HIV prevention

Studies involving self-administration of IPTp and daily oral HIV PrEP at home (as opposed to supervised administration at antenatal clinics) are eligible. However, trials investigating pharmacokinetics, efficacy and safety of individual agents (sulfadoxine-pyrimethamine for malaria, tenofovir/emtricitabine for HIV) in pregnant and postpartum cohorts did not sufficiently show that these agents can be effectively implemented in a self-care approach, and were thus excluded.

3. Interventions beyond the pregnancy period

(a) Preconception studies

In our review, preconception interventions were eligible only if they were designed to improve pregnancy outcomes and reduce risks of maternal or perinatal complications. Interventions to improve the chance of successful conception (e.g. in couples undertaking *in vitro* fertilisation therapy) were excluded.

Interventions to manage existing medical conditions were excluded unless they were framed in the context of preparation for pregnancy and evaluated pregnancy-related outcomes.

Example: Type 1 diabetes mellitus (T1DM)

Uncontrolled glucose levels during pregnancy may increase risks of miscarriage or birth defects. Therefore, interventions aimed at achieving glycaemic control in women with T1DM who wish to get pregnant were eligible. However, general studies of self-care interventions among women with T1DM (not otherwise specified) were excluded.

(b) Postpartum studies

The postpartum period is defined as the first 42 days after birth. Interventions initiated outside this time frame were excluded.

Interventions designed to facilitate newborn care (i.e. infant nutrition, postnatal infant monitoring, kangaroo care, etc.) and breastfeeding (i.e. measures to increase lactation, breastfeeding techniques, etc.) were excluded from our review as their broad scope warrants a separate focused review.

4. Role of digital health

Digital interventions were possibly considered self-care interventions as they can provide platforms for the woman to seek information, communicate with health providers and access external support, ultimately creating a supporting environment for self-care. However, use of digital communication alone does not determine whether an intervention is self-care intervention or not, but rather what practice is facilitated by its use.

(a) Client-to-client technology

- Digital platforms to connect the pregnant or postpartum women to support groups (e.g. trained volunteers, other mothers) were considered self-care interventions. The underlying assumption is that when the women use these platforms, she is actively seeking information and support from sources outside the health system.

(b) Client-to-provider technology

- The use of digital trackers to enable the woman to take appropriate actions for self-management of her health conditions (e.g. taking her own physical measurements or self-adjusting medications based on these measurements) is a self-care intervention.

For peer review only - http://bmjopen.bmj.com/site/about/guidelines.xhtml

- If the digital trackers feed information directly to the clinician for decision-making, without involving the patient in the process, it is not a self-care intervention.

> Example: Blood glucose monitoring
>
> Interventions where women check their own blood glucose levels using a glucometer were self-care interventions.
>
> Interventions using automatic monitoring systems (i.e. sensors that automatically measure glucose levels in interstitial fluid and transmit readings to mobile devices) were evaluated on a case-by-case basis. If the woman was taught to adjust her lifestyle, calibrate her medications or notify her clinician of hypoglycaemic episodes, the intervention is a self-care intervention. If the data was masked to the participants during the study duration, the study was excluded.
>
> Interventions where blood glucose levels were tested by a health worker in inpatient or outpatient settings were not self-care interventions.

(c) Provider-to-client technology

- The use of mHealth/eHealth to provide self-help information, instructions or reminders to women to encourage adoption of a healthy lifestyle (or otherwise change behaviour) is a self-care intervention.
- The use of telehealth for virtual consults to replace face-to-face antenatal or postnatal visits is not a self-care intervention, because virtual delivery only replaces the mode of communication between the patient and the clinician, but still requires a health worker to provide effective care.

5. Mixed intervention

If an intervention consists of a mix of self-care and non-self-care interventions, the programme was evaluated on a case-by-case basis. The factors considered to judge eligibility are:

- Whether self-care components constitute a majority of the intervention
- Whether the intervention as a whole places an emphasis on self-care or explicitly promotes self-care
- Whether the intervention represents task-sharing certain aspects of clinical care to the women or their family members

For peer review only - http://bmjopen.bmj.com/site/about/guidelines.xhtml

Supplementary File S5. Data collection form

	Field	Format	Option/Example
**General characteristics**	Study ID	Free text	e.g. McManus_2018
	Reviewer's initial	Free text	
	Title	Free text	
	Trial register number (if any)	Free text	
**Linking**	ID of other reports from the same studies	Free text	
	Ongoing study	Y/N	Yes if the protocol was published as a journal article from 2019 onwards and trial register records did not indicate study completion
**Methods**	Study design	Multiple choice	O Meta-analysis and network meta-analysis<br>O Systematic review without meta-analysis<br>O Scoping review<br>O Overview of reviews<br>O Randomised controlled trial<br>O Non-randomised interventional study (including pretest-posttest, quasi-randomised trial, etc.)<br>O Retrospective & prospective observational cohort study<br>O Cross-sectional study<br>O Diagnostic test accuracy study
	Country	Free text	N/A for reviews and meta-analyses
**Participants**	<18 years of age	Y/N	Yes if the study inclusion criteria specified a minimum age <18 or adolescent girls

For peer review only - http://bmjopen.bmj.com/site/about/guidelines.xhtml

	Field	Format	Option/Example
	High-risk pregnancies	Y/N	Yes if the study inclusion criteria included: age >35 years old, presence of risk behaviours (substance use, smoking), obesity or overweight, pre-existing or pregnancy-induced chronic conditions (diabetes mellitus, hypertension, thyroid disease, or other co-morbidities), history of pregnancy complications or pregnancy loss, HIV-positive status, and multiple pregnancies
**Interventions**	Self-care activities	List any that is applicable	Refer to Supplementary File S10 for the full list of self-care activities
	Modes of access to and delivery of self-care interventions	List any that is applicable	▪ Health facilities e.g. self-diagnostic tests provided and taken in facilities, and information on and demonstration of self-care interventions provided in health facilities<br>▪ Community and non-facility-based healthcare e.g. products and services delivered to home by community health workers; activities taking place at community centres; traditional, complementary or alternative medicines<br>▪ Peer and/or partner counselling and support<br>▪ Over-the-counter availability of drugs and therapeutics, medical devices and diagnostics through pharmacies and drug stores<br>▪ Digital technologies and platforms e.g. telemedicine, individuals tracking their personal health data, eHealth including health information and peer support, ordering tests or drugs online<br>▪ Healthcare during emergency and humanitarian responses e.g. in shelters and refugee camps and settlements<br>▪ Home-based care i.e. direct access to health products, tools and information at home<br>▪ School: education, support and counselling taking place at school<br>▪ Workplace: health, safety and well-being interventions provided within the workplace, such as first aid andinterventions to support mental health and avoid burnout
	Time of intervention	List any that is applicable	▪ Preconception<br>▪ Antenatal<br>▪ Intrapartum<br>▪ Postpartum: Up to 42 days postpartum

For peer review only - http://bmjopen.bmj.com/site/about/guidelines.xhtml

	Field	Format	Option/Example
			▪ N/A for reviews and meta-analyses
	Any external support in the uptake or implementation of self-care	List any that is applicable	▪ None<br>▪ Family: partner & immediate family<br>▪ Community members: non-familial, non-medically trained community members, including peer support groups, friends, social leaders<br>▪ CHWs: informally-trained health workers such as traditional birth attendants or doulas<br>▪ HCWs: doctors, nurses, midwives, allied health practitioners, therapists and research students involved in delivering care.<br>HCWs that were only involved in facilitating study conduct (i.e. recruiting, explaining the study procedures, training participants on using the self-care tools etc.), without delivering actual medical care, were not considered as having supported delivery of the intervention.<br>▪ N/A for reviews and meta-analyses
**Outcomes**	Maternal mortality	Y/N	Maternal death
	Perinatal mortality	Y/N	Miscarriage, stillbirth, neonatal death
	Neonatal outcomes	Y/N	Birth weight, Apgar score, preterm birth, birth injuries, gestational age at delivery & other neonatal complications/outcomes, etc.
	Labour outcomes	Y/N	Duration of labour, labour pain, onset of labour (spontaneous, induced or pre-labour C-section); mode of delivery (spontaneous vaginal, instrumental – forceps/vacuum, C-section planned vs unplanned), etc.
	Anthropometric & cardiometabolic outcomes	Y/N	Maternal weight & BMI, lipid profile, inflammatory biomarkers, etc.
	Diabetes & other metabolic disorders	Y/N	GDM/T2DM diagnosis, various glycaemic biomarkers & serum indicators, etc.
	Hypertensive disorders of pregnancy	Y/N	Preeclampsia, eclampsia, gestational/postpartum hypertension, HELLP syndrome, blood pressure measurements etc.
	Postpartum infection & haemorrhage	Y/N	Postpartum haemorrhage, amount of blood loss, puerperal sepsis, endometritis, surgical site infection, etc.

For peer review only

	Field	Format	Option/Example
	Urogenital outcomes	Y/N	Urinary incontinence, urinary tract infections, pelvic floor disorders, postpartum perineal issues etc.
	Nutritional deficiencies	Y/N	Any nutritional deficiencies or serum levels of micronutrients, iron-deficiency anaemia, etc.
	Gastrointestinal outcomes	Y/N	Nausea & vomiting, constipation, etc.
	Other maternal complications	Y/N	Renal dysfunction, liver dysfunction & any other maternal complications not elsewhere classified
	Infectious diseases	Y/N	Malaria, HIV, STI, etc.
	Other minor physiological discomforts	Y/N	Breast & nipple issues, lumbopelvic pain & other minor ailments not elsewhere classified
	Mental wellbeing & quality of life	Y/N	Depression, anxiety, sexual functioning, childbirth fear/readiness
	Behavioural change	Y/N	Smoking cessation, alcohol use reduction, contraceptive use, supplement intake, physical activity & any other health-promoting or risk-avoiding behaviours
	Breastfeeding*	Y/N	Breastfeeding patterns (exclusive/full/partial), duration, initiation or continuation
	Other	Y/N	Diagnostic performance of tests, accordance with gold standards, intimate partner violence
**Self-care category (based on WHO classification [1])**	Individual Agency	List any that is applicable	▪ Interventions to promote awareness about self-care<br>▪ Interventions to promote autonomy and/or confidence to engage in self-care<br>▪ Interventions for improving self-care capacities and capabilities<br>▪ Interventions for improving health and digital literacy<br>▪ Interventions to promote sustained adoption of self-care practices and behaviours
	Health information-seeking	List any that is applicable	▪ Acquiring health education for informed health decision-making<br>▪ Improving health literacy to support health decision-making<br>▪ Individuals seeking health information via on-demand information services<br>▪ Individuals health decision-making support via online or mobile tools
	Social and community support	List any that is applicable	▪ Peer mentorship and counselling<br>▪ Peer-to-peer action to support access to and the uptake and use of self-care interventions<br>▪ Individuals sharing health data with peers

For peer review only - http://bmjopen.bmj.com/site/about/guidelines.xhtml

	Field	Format	Option/Example
	Personal health tracking	List any that is applicable	▪ Home-based record for health and diagnostic data<br>▪ Self-monitoring of health<br>▪ Active data capture/documentation by self-care user<br>▪ Passive data capture/documentation by device<br>▪ Use of diagnostic device at community location for<br>▪ self-measuring health indicator<br>▪ Tracking rational and responsible self-use of medicines and therapeutics
	Self-diagnosis of health conditions	List any that is applicable	▪ Self-testing, including to inform prevention<br>▪ Self-examining for health conditions<br>▪ Self-collection of samples to send for external testing<br>▪ Using online symptom checkers and health information
	Self-management of health	List any that is applicable	▪ Self-care for long-term health conditions<br>▪ Self-care for short-term health conditions<br>▪ Self-medication/treatment without medical prescription including for side effects<br>▪ Self-regulation of health conditions<br>▪ Self-care prevention including risk avoidance and support for physical and mental health and well-being<br>▪ Health management performed by caregivers<br>▪ Individuals procurement of safe over-the-counter medicines and products for self-care
	Individuals linkage to health system	List any that is applicable	▪ Individuals-initiated communication with health system<br>▪ Individuals sharing health data with health workers<br>▪ Individuals identifying location of health facilities/structures<br>▪ Individuals receiving feedback from health workers
	Individuals financial transactions for health	-	Not eligible under this review

**Although breastfeeding interventions were not eligible for inclusion, breastfeeding outcomes were recorded as they were often evaluated in interventions targeting other health issues, such as smoking cessation, diabetes management, breast & nipple care, etc.*

[1] World Health Organization. Classification of self-care interventions for health: a shared language to describe the uses of self-care interventions. Geneva, 2021 https://www.who.int/publications/i/item/9789240039469 (accessed April 6, 2022).

For peer review only - http://bmjopen.bmj.com/site/about/guidelines.xhtml

Supplementary File S6. List of excluded full texts

Author/Year	Title	Reasons for exclusion
Abbaspoor_2020	The effect of education through mobile phone short message service on promoting self-care in pre-diabetic pregnant women: A randomized controlled trial	Comparison - no appropriate control
Akanbi_2005	Effect of self-medication with antimalarial drugs on malaria infection in pregnant women in south-western Nigeria	Comparison - no appropriate control
Alemi_1996	Electronic self-help and support groups	Comparison - no appropriate control
Barbato_2014	Web-Based Treatment for Women With Stress Urinary Incontinence	Comparison - no appropriate control
Bradizza_2017	Smoking Cessation for Pregnant Smokers: Development and Pilot Test of an Emotion Regulation Treatment Supplement to Standard Smoking Cessation for Negative Affect Smokers	Comparison - no appropriate control
Burgess_2021	Feasibility of Using Blood Pressure Self-Monitoring and the Epic MyChart Blood Pressure Flowsheet to Monitor Blood Pressure after Preeclampsia	Comparison - no appropriate control
Bussiere-Cote_2019	Twice-Daily vs 4-Times-Daily Glucose Testing in Women With Gestational Diabetes Mellitus: a Pilot Study	Comparison - no appropriate control
Cao_2012	Comprehensive intensive therapy for Chinese gestational diabetes benefits both newborns and mothers	Comparison - no appropriate control
Chi_2016	Randomized comparison of group versus individual educational interventions for pregnant women to reduce their secondhand smoke exposure	Comparison - no appropriate control
Chung_2009	Validation and compliance of a home monitoring device in pregnancy: Microlife WatchBP home	Comparison - no appropriate control
Clapp_1989	The effects of maternal exercise on early pregnancy outcome	Comparison - no appropriate control
Cuneo_2017	Heart sounds at home: feasibility of an ambulatory fetal heart rhythm surveillance program for anti-SSA-positive pregnancies	Comparison - no appropriate control
Darvall_2020	A Pedometer-Guided Physical Activity Intervention for Obese Pregnant Women (the Fit MUM Study): randomized Feasibility Study	Comparison - no appropriate control
Dettinger_2019	PrEP Implementation for Mothers in Antenatal Care (PrIMA): Study protocol of a cluster randomised trial	Comparison - no appropriate control
Dyson_1998	Monitoring women at risk for preterm labor	Comparison - no appropriate control
Ganapathy_2016	Remote monitoring of blood pressure to reduce the risk of preeclampsia related complications with an innovative use of mobile technology	Comparison - no appropriate control
Gao_2012	Effects of an interpersonal-psychotherapy-oriented childbirth education programme for Chinese first-time childbearing women at 3-month follow up: randomised controlled trial	Comparison - no appropriate control
Gao_2021	An psychoeducation programme based on self-efficacy theory to improve experience of natural birth for primigravid women: Study protocol for a randomised controlled trial	Comparison - no appropriate control
Ghasemi_2021	Comparing the effect of individual counseling with counseling on social application on self-care and quality of life of women with gestational diabetes	Comparison - no appropriate control
Heuvel_2019	SAFE@HOME - Feasibility study of a telemonitoring platform combining blood pressure and preeclampsia symptoms in pregnancy care	Comparison - no appropriate control
Heuvel_2019	Validation of the iHealth Track and Omron HEM-9210T automated blood pressure devices for use in pregnancy	Comparison - no appropriate control
Hoppe_2016	An evaluation of diabetes targeted apps for Android smartphone in relation to behaviour change techniques	Comparison - no appropriate control
Humphries_2021	Decision Analysis in SHared decision making for Thromboprophylaxis during Pregnancy (DASH-TOP): a sequential explanatory mixed methods pilot study protocol	Comparison - no appropriate control
Iwama_2016	Maternal clinic and home blood pressure measurements during pregnancy and infant birth weight: The BOSHI study	Comparison - no appropriate control
Jiao_2019	Web-based versus home-based postnatal psychoeducational interventions for first-time mothers: A randomised controlled trial	Comparison - no appropriate control
Johnson_2018	Impacting diabetes self-management in women with gestational diabetes mellitus using short messaging reminders	Comparison - no appropriate control
Kerner_2004	Maternal self-administered fetal heart rate monitoring and transmission from home in high-risk pregnancies	Comparison - no appropriate control
Kominiarek_2019	A feasibility study of activity tracking devices in pregnancy	Comparison - no appropriate control
Lan_2017	Home blood pressure measurement in women with pregnancy-related hypertensive disorders	Comparison - no appropriate control
Lihme_2017	Self-monitoring of blood pressure during pregnancy: An observational study in the obstetrical waiting room	Comparison - no appropriate control
Limruangrong_2011	Effectiveness of a self-regulation program on diet control, exercise, and two-hour postprandial blood glucose levels in Thais with gestational diabetes mellitus	Comparison - no appropriate control
Malm_2014	Women's experiences of two different self-assessment methods for monitoring fetal movements in full-term pregnancy - a crossover trial	Comparison - no appropriate control
Manfredi_2011	Dissemination strategies to improve implementation of the PHS smoking cessation guideline in MCH public health clinics: experimental evaluation results and contextual factors	Comparison - no appropriate control

For peer review only - http://bmjopen.bmj.com/site/about/guidelines.xhtml

Author/Year	Title	Reasons for exclusion
Mantzana_2014	Applicability of self-obtained urine and vaginal samples for HPV-16, -18, -31 and -45 cervical cancer screening in pregnancy: A pilot cross-sectional study	Comparison - no appropriate control
Mendez-Figueroa_2017	Gestational Diabetes Mellitus and Frequency of Blood Glucose Monitoring: A Randomized Controlled Trial	Comparison - no appropriate control
Mikami_2017	Provisional criteria for the diagnosis of hypertension in pregnancy using home blood pressure measurements	Comparison - no appropriate control
Navaee_2020	Effect of pre-cesarean foot reflexology massage on anxiety of primiparous women	Comparison - no appropriate control
Pauley_2018	Gestational Weight Gain Intervention Impacts Determinants of Healthy Eating and Exercise in Overweight/Obese Pregnant Women	Comparison - no appropriate control
Pyra_2018	Tenofovir and tenofovir-diphosphate concentrations during pregnancy among HIV-uninfected women using oral preexposure prophylaxis	Comparison - no appropriate control
Raynes-Greenow_2009	Do audio-guided decision aids improve outcomes? A randomized controlled trial of an audio-guided decision aid compared with a booklet decision aid for Australian women considering labour analgesia	Comparison - no appropriate control
Roske_2008	Postpartum smoking cessation and relapse prevention intervention: A structural equation modeling application to behavioral and non-behavioral outcomes of a randomized controlled trial	Comparison - no appropriate control
Saffari_2020	Effect of a health-education program using motivational interviewing on oral health behavior and self-efficacy in pregnant women: a randomized controlled trial	Comparison - no appropriate control
Salomon_2004	Fetal home monitoring for the prenatal management of gastroschisis	Comparison - no appropriate control
Shieh_2017	Feasibility and Potential Benefits of a Self-Monitoring Enhanced Lifestyle Intervention to Prevent Excessive Gestational Weight Gain in Women Who Are Overweight or Obese	Comparison - no appropriate control
Smith_2014	Advance distribution of misoprostol for prevention of postpartum hemorrhage (PPH) at home births in two districts of Liberia	Comparison - no appropriate control
Svensson_2009	Randomised-controlled trial of two antenatal education programmes	Comparison - no appropriate control
Takeuchi_2016	Randomised controlled trial using smartphone website vs leaflet to support antenatal perineal massage practice for pregnant women	Comparison - no appropriate control
Tremonti_2017	Reliability of home blood pressure monitoring devices in pregnancy	Comparison - no appropriate control
Tucker_2018	How do home and clinic blood pressure readings compare in pregnancy? A systematic review and individual patient data meta-analysis	Comparison - no appropriate control
Twedt_2015	Sleep Duration and Blood Glucose Control in Women With Gestational Diabetes Mellitus	Comparison - no appropriate control
Ussher_2017	The London Exercise And Pregnant smokers (LEAP) trial: a randomised controlled trial of physical activity for smoking cessation in pregnancy with an economic evaluation	Comparison - no appropriate control
Vakilian_2018	Controlled breathing with or without lavender aromatherapy for labor pain at the first stage: a randomized clinical trial	Comparison - no appropriate control
Valbo_1991	Smoking cessation in pregnancy Mode of intervention and effect	Comparison - no appropriate control
Valiani_2014	Comparison of childbirth training workshop effects on knowledge, attitude, and delivery method between mothers and couples groups referring to Isfahan health centers in Iran	Comparison - no appropriate control
Vallely_2016	Feasibility and acceptability of clean birth kits containing misoprostol for self-administration to prevent postpartum hemorrhage in rural Papua New Guinea	Comparison - no appropriate control
Waugh_2003	Patient initiated home blood pressure recordings are accurate in hypertensive pregnant women	Comparison - no appropriate control
Wu_2021	Application of diversified and quantitative management model of exercise intervention in patients with gestational diabetes mellitus	Comparison - no appropriate control
Young_2000	The effectiveness of weekly iron supplementation in pregnant women of rural northern Malawi	Comparison - no appropriate control
Chen_2020	Non-pharmacological interventions for postpartum depression: A protocol for systematic review and network meta-analysis	Design - not a systematic review
Chow_2021	Appraisal of systematic reviews on interventions for postpartum depression: systematic review	Design - not a systematic review
Cottrell_2010	An updated review of of evidence to discourage douching	Design - not a systematic review
Davies_2018	Global and national guidance for the use of pre-exposure prophylaxis during peri-conception, pregnancy and breastfeeding	Design - not a systematic review
Dennis_2014	Psychosocial interventions for the treatment of perinatal depression	Design - not a systematic review
Devoe_2000	Home uterine activity monitoring: A critical review	Design - not a systematic review
Dominguez-Solis_2021	Non-pharmacological interventions to reduce anxiety in pregnancy, labour and postpartum: A systematic review	Design - not a systematic review
Edmonds_2014	Shared decision-making and decision support: Their role in obstetrics and gynecology	Design - not a systematic review
Evans_2018	Systematic review and meta-analysis of non-pharmacological interventions to reduce the symptoms of mild to moderate anxiety in pregnant women	Design - not a systematic review

For peer review only - http://bmjopen.bmj.com/site/about/guidelines.xhtml

Author/Year	Title	Reasons for exclusion
Feig_2018	Continuous glucose monitoring in pregnant women with Type 1 diabetes: benefits for mothers, using pumps or pens, and their babies	Design - not a systematic review
Flash_2017	Pre-exposure prophylaxis for HIV prevention in women: Current perspectives	Design - not a systematic review
Grange_2020	Smoking and smoking cessation in pregnancy Synthesis of a systematic review	Design - not a systematic review
Greaves_2019	Tailored Intervention for Smoking Reduction and Cessation for Young and Socially Disadvantaged Women During Pregnancy	Design - not a systematic review
Horgan_2019	Pre-exposure prophylaxis for HIV prevention during pregnancy and lactation: forget not the women and children	Design - not a systematic review
Hoyme_2010	Prevention of Preterm Birth Is Possible by Vaginal pH Screening, Early Diagnosis of Bacterial Vaginosis or Abnormal Vaginal Flora and Treatment	Design - not a systematic review
Joubert_2012	Personal continuous glucose monitoring (CGM) in diabetes management: Review of the literature and implementation for practical use	Design - not a systematic review
Jovanovic_2008	Using meal-based self-monitoring of blood glucose as a tool to improve outcomes in pregnancy complicated by diabetes	Design - not a systematic review
Koukoulithras_2021	The Effectiveness of Non-Pharmaceutical Interventions Upon Pregnancy-Related Low Back Pain: A Systematic Review and Meta-Analysis	Design - not a systematic review
Lambermon_2020	Maternal self-care in the early postpartum period: An integrative review	Design - not a systematic review
Melvin_2000	Recommended cessation counselling for pregnant women who smoke: A review of the evidence	Design - not a systematic review
Menendez_2007	Reducing the burden of malaria in pregnancy by preventive strategies	Design - not a systematic review
Montag_2012	A review of evidence-based approaches for reduction of alcohol consumption in Native women who are pregnant or of reproductive age	Design - not a systematic review
Moradi_2020	COVID-19 and self-care strategies for women with gestational diabetes mellitus	Design - not a systematic review
Nosova_2020	Leveraging technology for the treatment of type 1 diabetes in pregnancy: A review of past, current, and future therapeutic tools	Design - not a systematic review
Rodbard_2017	Continuous glucose monitoring: A review of recent studies demonstrating improved glycemic outcomes	Design - not a systematic review
Sachs_1991	Home monitoring of uterine activity Does it prevent prematurity?	Design - not a systematic review
Siebern_2012	Non-Pharmacological Treatment of Insomnia	Design - not a systematic review
Stalter_2021	Safety review of tenofovir disoproxil fumarate/emtricitabine pre-exposure prophylaxis for pregnant women at risk of HIV infection	Design - not a systematic review
Zinsser_2020	Changing behaviour in pregnant women: A scoping review	Design - not a systematic review
Cairns_2020	Implementing self-management: A mixed methods study of women's experiences of a postpartum hypertension intervention (SNAP-HT)	Design - qualitative study
Chiarelli_2003	Acceptability of a urinary continence promotion programme to women in postpartum	Design - qualitative study
Gosha_1986	A self-help group for new mothers: an evaluation	Design - qualitative study
Gueguen_2021	Hypnosis for labour and childbirth: A meta-integration of qualitative and quantitative studies	Design - qualitative study
Jidong_2021	Psychological interventions for maternal depression among women of African and Caribbean origin: a systematic review	Design - qualitative study
Labrecque_2001	Women's views on the practice of prenatal perineal massage	Design - qualitative study
Lee_2008	A pilot intervention for pregnant women in Sichuan, China on passive smoking	Design - qualitative study
McCaffrey_2020	The role and outcomes of music listening for women in childbirth: An integrative review	Design - qualitative study
Moulaei_2021	The Development and Usability Assessment of an mHealth Application to Encourage Self-Care in Pregnant Women against COVID-19	Design - qualitative study
Price_1991	Comparison of three antismoking interventions among pregnant women in an urban setting: A randomized trial	Design - qualitative study
Radestad_2021	Midwives' experiences of using the Mindfetalness method when talking with pregnant women about fetal movements	Design - qualitative study
Shorey_2019	A systematic mixed-studies review on mindfulness-based childbirth education programs and maternal outcomes	Design - qualitative study
Stonbraker_2020	Establishing content for a digital educational support group for new adolescent mothers in the Dominican Republic: A user-centered design approach	Design - qualitative study
Wallace_2009	A pregnancy wellness guide to enhance care through self-assessment, personal reflection, and self-referral	Design - qualitative study
Yoshida_2001	Postnatal depression in Japanese mothers and the reconsideration of 'Satogaeri bunben'	Design - qualitative study
Bivia-Roig_2020	Efficacy of an internet-based intervention to promote a healthy lifestyle on the reproductive parameters of overweight and obese women: study protocol for a randomised controlled trial	Intervention - conception/fertility
Boedt_2021	Systematic development of a mobile preconception lifestyle programme for couples undergoing IVF: the PreLiFe-programme	Intervention - conception/fertility

For peer review only - http://bmjopen.bmj.com/site/about/guidelines.xhtml

Author/Year	Title	Reasons for exclusion
Galletly_1996	Improved pregnancy rates for obese, infertile women following a group treatment program: An open pilot study	Intervention - conception/fertility
Kim_2020	Effectiveness of non-pharmacological interventions for overweight or obese infertile women: A systematic review and meta-analysis	Intervention - conception/fertility
Soltani_2015	Maternal obesity management using mobile technology: A feasibility study to evaluate a text messaging based complex intervention during pregnancy	Intervention - conception/fertility
Sridhar_2020	Non-pharmacological anxiety reduction with immersive virtual reality for first-trimester dilation and curettage: a pilot study	Intervention - conception/fertility
Taghavi_2021	Pharmacological and non?pharmacological strategies for obese women with subfertility	Intervention - conception/fertility
Ahn_2004	The Effects of a Home-Visiting Discharge Education on Maternal Self-esteem, Maternal Attachment, PostpartumDepression and Family Function in theMothers of NICU Infants	Intervention - newborn care
Da?li_2021	The effect of oxytocin massage and music on breast milk production and anxiety level of the mothers of premature infants who are in the neonatal intensive care unit: A self-controlled trial	Intervention - newborn care
Deave_2019	A study to evaluate the effectiveness of Best Beginnings' Baby Buddy phone app in England: a protocol paper	Intervention - newborn care
Dol_2021	Effectiveness of a Postpartum Text Message Program (Essential Coaching for Every Mother) on Maternal Psychosocial Outcomes: Protocol for a Randomized Controlled Trial	Intervention - newborn care
Jamalivand_2017	Comparing the effect of electronic software and training booklet on maternal self-confidence and awareness about newborn care: A randomized controlled clinical trial	Intervention - newborn care
Missler_2020	Effectiveness of a psycho-educational intervention for expecting parents to prevent postpartum parenting stress, depression and anxiety: a randomized controlled trial	Intervention - newborn care
Mohammadi_2018	The effect of stress inoculation training on breastfeeding self-efficacy and perceived stress of mothers with low birth weight infants: A clinical trial	Intervention - newborn care
Ngai_2009	The effects of a childbirth psychoeducation program on learned resourcefulness, maternal role competence and perinatal depression: a quasi-experiment	Intervention - newborn care
Ondersma_2018	Computer-delivered indirect screening and brief intervention for drug use in the perinatal period: A randomized trial	Intervention - newborn care
Shorey_2015	A randomized controlled trial of the effectiveness of a postnatal psychoeducation programme on self-efficacy, social support and postnatal depression among primiparas	Intervention - newborn care
Shorey_2017	Effectiveness of the 'Home-but not Alone' mobile health application educational programme on parental outcomes: a randomized controlled trial, study protocol	Intervention - newborn care
Allard_2015	Gestational Diabetes Mellitus Identification Based on Self-Monitoring of Blood Glucose	Intervention - no intervention identified
Barbosa_2016	Educational technologies to encourage (self) care in postpartum women	Intervention - no intervention identified
Barrera_2015	Online prevention of postpartum depression for Spanish- and English-speaking pregnant women: a pilot randomized controlled trial	Intervention - no intervention identified
Bessinger_2002	Substrate utilization and hormonal responses to moderate intensity exercise during pregnancy and after delivery	Intervention - no intervention identified
Black-Olien_1993	The effects of prenatal programs on postpartum emotional well-being	Intervention - no intervention identified
Carlson_2018	Antepartum Care of Women Who Are Obese During Pregnancy: Systematic Review of the Current Evidence	Intervention - no intervention identified
Chung_2020	Medical help-seeking strategies for perinatal women with obstetric and mental health problems and changes in medical decision making based on online health information: Path analysis	Intervention - no intervention identified
Clapp_2008	Long-term outcome after exercising throughout pregnancy: fitness and cardiovascular risk	Intervention - no intervention identified
Cosson_2017	Poor reliability and poor adherence to self-monitoring of blood glucose are common in women with gestational diabetes mellitus and may be associated with poor pregnancy outcomes	Intervention - no intervention identified
Dennis_2007	Psychosocial and psychological interventions for treating antenatal depression	Intervention - no intervention identified
Dennis-Tiwary_2017	Salutary effects of an attention bias modification mobile application on biobehavioral measures of stress and anxiety during pregnancy	Intervention - no intervention identified
Dol_2020	Impact of mobile health interventions during the perinatal period on maternal psychosocial outcomes: A systematic review	Intervention - no intervention identified
Ekelof_2021	Depressive symptoms postpartum is associated with physical activity level the year prior to giving birth - A retrospective observational study	Intervention - no intervention identified
Feng_2021	Psychological or educational eHealth interventions on depression, anxiety or stress following preterm birth: a systematic review	Intervention - no intervention identified
Gbagbo_2020	Implications of self-medication in pregnancy for Safe Motherhood and Sustainable Development Goal-3 in selected Ghanaian communities	Intervention - no intervention identified
Gribel_2020	Influence of non-pharmacological obstetric interventions on adverse outcomes of childbirth under regional analgesia	Intervention - no intervention identified
Hall_2011	The use of complementary and alternative medicine by pregnant women: a literature review	Intervention - no intervention identified
Hewage_2020	Barriers to Gestational Diabetes Management and Preferred Interventions for Women With Gestational Diabetes in Singapore: Mixed Methods Study	Intervention - no intervention identified

For peer review only - http://bmjopen.bmj.com/site/about/guidelines.xhtml

Author/Year	Title	Reasons for exclusion
Hong_2021	Perspectives on antenatal education associated with pregnancy outcomes: Systematic review and meta-analysis	Intervention - no intervention identified
Hoyme_2020	Reduced incidence of early preterm birth in the State of Thuringia following an intravaginal pH-self-monitoring screening program	Intervention - no intervention identified
Igwesi-Chidobe_2021	Community-based non-pharmacological interventions for improving pain, disability and quality of life in pregnant women with musculoskeletal conditions: Protocol for a systematic review with meta-analyses	Intervention - no intervention identified
Iyawa_2021	Mobile apps for self-management in pregnancy: a systematic review	Intervention - no intervention identified
Jung_2021	Psychosocial support interventions for women with gestational diabetes mellitus: a systematic review	Intervention - no intervention identified
Kim_2017	Effectiveness of Psychosocial and Educational Prenatal and Postnatal Care Interventions for Married Immigrant Women in Korea: Systematic Review and Meta-analysis	Intervention - no intervention identified
Lau_2016	Efficacy of Internet-Based Self-Monitoring Interventions on Maternal and Neonatal Outcomes in Perinatal Diabetic Women: A Systematic Review and Meta-Analysis	Intervention - no intervention identified
Leis_2009	A systematic review of home-based interventions to prevent and treat postpartum depression	Intervention - no intervention identified
Leventhal_1989	Active coping reduces reports of pain from childbirth	Intervention - no intervention identified
Lin_2018	Effectiveness of self-help psychological interventions for treating and preventing postpartum depression: a meta-analysis	Intervention - no intervention identified
Mackiewicz-Seghete_2020	Protocol for a mechanistic study of mindfulness based cognitive therapy during pregnancy	Intervention - no intervention identified
Manandhar_2004	Effect of a participatory intervention with women's groups on birth outcomes in Nepal: cluster-randomised controlled trial	Intervention - no intervention identified
Martin_2017	Adherence-Specific Social Support Enhances Adherence to Calcium Supplementation Regimens among Pregnant Women	Intervention - no intervention identified
Masoumi_2018	Effect of a sex education program on females' sexual satisfaction during pregnancy: a randomized clinical trial	Intervention - no intervention identified
Mekonnen_2021	Adherence to iron with folic acid supplementation among pregnant women attending antenatal care in public health centers in simada district, northwest ethiopia: using health belief model perspective	Intervention - no intervention identified
Mertens_2019	Effect of Lifestyle Coaching Including Telemonitoring and Telecoaching on Gestational Weight Gain and Postnatal Weight Loss: A Systematic Review	Intervention - no intervention identified
Mohamadirizi_2018	The effect of religious-spiritual support on childbirth self-efficacy	Intervention - no intervention identified
Nadal_2021	Educational films for improving screening and self-management of gestational diabetes in India and Uganda (GUIDES): study protocol for a cluster-randomised controlled trial	Intervention - no intervention identified
Naughton_2015	Use and effectiveness of lapse prevention strategies among pregnant smokers	Intervention - no intervention identified
Navidian_2017	The effect of home-based supportive-educational counseling on primigravidas' postpartum stress	Intervention - no intervention identified
Nazik_2013	The Prevention And Reduction Of Postpartum Complications: Orem's Model	Intervention - no intervention identified
Newham_2012	State-trait anxiety inventory (STAI) scores during pregnancy following intervention with complementary therapies	Intervention - no intervention identified
Notley_2019	Development of a complex intervention for the maintenance of postpartum smoking abstinence: process for defining evidence-based intervention	Intervention - no intervention identified
O'Connell_2021	Interventions for fear of childbirth including tocophobia	Intervention - no intervention identified
Ogrodniczuk_2003	Preventing Postnatal Depression: a Review of Research Findings	Intervention - no intervention identified
Olander_2015	Person-centred care in interventions to limit weight gain in pregnant women with obesity - a systematic review	Intervention - no intervention identified
Olayiwola_2013	Living Smart, Living Fit: a patient-centered program to improve perinatal outcomes in a community health center population	Intervention - no intervention identified
Ouedraogo_2013	Maternal anemia in pregnancy: Assessing the effect of routine preventive measures in a malaria-endemic area	Intervention - no intervention identified
Ratnayake_2018	Validity of over the counter finger stick glucose measurement devices in comparison with laboratory venous plasma glucose measurements on pregnant women with diabetes	Intervention - no intervention identified
Sayakhot_2016	Use of a web-based educational intervention to improve knowledge of healthy diet and lifestyle in women with Gestational Diabetes Mellitus compared to standard clinic-based education	Intervention - no intervention identified
Silverstein_2011	Problem-solving education to prevent depression among low-income mothers of preterm infants: a randomized controlled pilot trial	Intervention - no intervention identified
Sinclair_2017	A systematic literature review of computer-based behavioural change interventions to inform the design of an online VBAC intervention for the OptiBIRTH European randomised trial (project HEALTH -- F3 -- 2012-305208)	Intervention - no intervention identified
Song_2015	A systematic review of psychosocial interventions for women with postpartum stress	Intervention - no intervention identified
Sun_2020	A mushroom diet reduced the risk of pregnancy-induced hyper tension and macrosomia: A randomized clinical trial	Intervention - no intervention identified

For peer review only - http://bmjopen.bmj.com/site/about/guidelines.xhtml

Author/Year	Title	Reasons for exclusion
Westerhoff_2019	About:blank? Online Interventions for Postpartum Depression	Intervention - no intervention identified
Windsor_2016	A Process Evaluation of the WV Smoking Cessation and Reduction in Pregnancy Treatment (SCRIPT) Dissemination Initiative: Assessing the Fidelity and Impact of Delivery for State-Wide, Home-Based Healthy Start Services	Intervention - no intervention identified
Wulp_2016	Correlates of partner support to abstain from prenatal alcohol use: a cross-sectional survey among Dutch partners of pregnant women	Intervention - no intervention identified
Wylie_2018	Evaluation of patient- versus provider-collected vaginal swabs for microbiome analysis during pregnancy	Intervention - no intervention identified
Abbas_2020	Testing a home-based model of care using misoprostol for prevention and treatment of postpartum hemorrhage: Results from a randomized placebo-controlled trial conducted in Badakhshan province, Afghanistan	Intervention - not self-care
Abdollahi_2020	Effect of Psychotherapy on Reduction of Fear of Childbirth and Pregnancy Stress: A Randomized Controlled Trial	Intervention - not self-care
Abuogi_2020	HIV status disclosure patterns and male partner reactions among pregnant women with HIV on lifelong ART in Western Kenya	Intervention - not self-care
Afandi_2019	The value of Continuous Glucose Monitoring and Self-Monitoring of Blood Glucose in patients with Gestational Diabetes Mellitus during Ramadan fasting	Intervention - not self-care
Agarwal_2012	Evaluation of isosorbide mononitrate for cervical ripening prior to induction of labor for postdated pregnancy in an outpatient setting	Intervention - not self-care
Ahmad_2021	The Knowledge of Danger Signs of Obstetric Complications among Women in Rural India: Evaluating an Integrated Microfinance and Health Literacy Program	Intervention - not self-care
Akbarzadeh_2015	Comparison of the Effects of Maternal Supportive Care and Acupressure (at BL32 Acupoint) on Labor Length and Infant's Apgar Score	Intervention - not self-care
Akpan_2018	Antenatal Deworming and Materno-Perinatal Outcomes in Calabar, Nigeria	Intervention - not self-care
Alfirevic_2020	Home versus inpatient induction of labour for improving birth outcomes	Intervention - not self-care
Alimoradi_2019	Comparing the effect of auricular acupressure and body acupressure on pain and duration of the first stage of labor: study protocol for a randomized controlled trial	Intervention - not self-care
Amanak_2019	The impact of prenatal education based on the Roy adaptation model on gestational hypertension, adaptation to pregnancy and pregnancy outcomes	Intervention - not self-care
Anderson_2021	Effects of a Home-Based Intervention on HIV Prevention Health Behaviors in Pregnant/Postpartum Kenyan Women: Estimating Moderating Effects of Depressive Symptoms	Intervention - not self-care
Aquino_2020	Exploring the Use of Telemonitoring for Patients at High Risk for Hypertensive Disorders of Pregnancy in the Antepartum and Postpartum Periods: scoping Review	Intervention - not self-care
Ayerle_2018	Effects of the birthing room environment on vaginal births and client-centred outcomes for women at term planning a vaginal birth: BE-UP, a multicentre randomised controlled trial	Intervention - not self-care
Bain_2014	Interventions for managing asthma in pregnancy	Intervention - not self-care
Baljon_2020	Effectiveness of breathing exercises, foot reflexology and back massage (BRM) on labour pain, anxiety, duration, satisfaction, stress hormones and newborn outcomes among primigravidae during the first stage of labour in Saudi Arabia: a study protocol for	Intervention - not self-care
Bar-Zeev_2019	Improving smoking cessation care in pregnancy at Aboriginal Medical Services: 'ICAN QUIT in Pregnancy' step-wedge cluster randomised study	Intervention - not self-care
Batool_2018	Comparison of the efficacy of IV iron versus oral iron therapy in Postpartum Anemia	Intervention - not self-care
Beckmann_1996	Accuracy of maternal perception of preterm uterine activity	Intervention - not self-care
Beiranvand_2014	The effects of religion and spirituality on postoperative pain, hemodynamic functioning and anxiety after cesarean section	Intervention - not self-care
Bhandal_2006	Intravenous versus oral iron therapy for postpartum anaemia	Intervention - not self-care
Birdsall_2002	Benefits of monitoring blood glucose by collection of blood on filter paper strips	Intervention - not self-care
Bishop_2016	Evaluating acupuncture and standard care for pregnant women with back pain (ease back): a pilot randomised trial	Intervention - not self-care
Bolinder_2016	Novel glucose-sensing technology and hypoglycaemia in type 1 diabetes: a multicentre, non-masked, randomised controlled trial	Intervention - not self-care
Bollapragada_2006	IMOP: Randomised placebo controlled trial of outpatient cervical ripening with isosorbide mononitrate (IMN) prior to induction of labour - Clinical trial with analyses of efficacy, cost effectiveness and acceptability	Intervention - not self-care
Bollapragada_2009	Randomized placebo-controlled trial of outpatient (at Home) cervical ripening with isosorbide mononitrate (IMN) prior to induction of labor-clinical trial with analyses of efficacy and acceptability: the IMOP study	Intervention - not self-care
Brown_1999	A randomized comparison of home uterine activity monitoring in the outpatient management of women treated for preterm labor	Intervention - not self-care
Bryce_2009	CATCH: development of a home-based midwifery intervention to support young pregnant smokers to quit	Intervention - not self-care
Bullarbo_2007	Outpatient vaginal administration of the nitric oxide donor isosorbide mononitrate for cervical ripening and labor induction postterm: a randomized controlled study	Intervention - not self-care
Bullock_2009	Baby BEEP: a randomized controlled trial of nurses' individualized social support for poor rural pregnant smokers	Intervention - not self-care
ButlerTobah_2019	Randomized comparison of a reduced-visit prenatal care model enhanced with remote monitoring	Intervention - not self-care
Carter_2020	Pilot Randomized Controlled Trial of Diabetes Group Prenatal Care	Intervention - not self-care

Author/Year	Title	Reasons for exclusion
Catling-Paull_2011	Non-clinical interventions that increase the uptake and success of vaginal birth after caesarean section: a systematic review	Intervention - not self-care
Chen_2003	Continuous glucose monitoring for the evaluation and improved control of gestational diabetes mellitus	Intervention - not self-care
Choko_2017	Investigating interventions to increase uptake of HIV testing and linkage into care or prevention for male partners of pregnant women in antenatal clinics in Blantyre, Malawi: Study protocol for a cluster randomised trial	Intervention - not self-care
Choko_2019	HIV self-testing alone or with additional interventions, including financial incentives, and linkage to care or prevention among male partners of antenatal care clinic attendees in Malawi: An adaptive multi-arm, multi-stage cluster randomised trial	Intervention - not self-care
Choko_2021	Partner-delivered HIV self-test kits with and without financial incentives in antenatal care and index patients with HIV in Malawi: a three-arm, cluster-randomised controlled trial	Intervention - not self-care
CHUMS-Group_1995	A multicenter randomized controlled trial of home uterine monitoring: active versus sham device The Collaborative Home Uterine Monitoring Study (CHUMS) Group	Intervention - not self-care
Chung_2012	Randomized non-invasive sham-controlled pilot trial of electroacupuncture for postpartum depression	Intervention - not self-care
Clarke_2020	OptiBIRTH: a cluster randomised trial of a complex intervention to increase vaginal birth after caesarean section	Intervention - not self-care
Colton_1995	A metaanalysis of home uterine activity monitoring	Intervention - not self-care
Corey_2019	Bedside Music Therapy for Women during Antepartum and Postpartum Hospitalization	Intervention - not self-care
Corna_2019	Supporting maternal mental health of Rohingya refugee women during the perinatal period to promote child health and wellbeing: A field study in Cox's Bazar	Intervention - not self-care
Corwin_1996	Multicenter randomized clinical trial of home uterine activity monitoring: Pregnancy outcomes for all women randomized	Intervention - not self-care
Cottrell_2006	Vaginal douching practices of women in eight Florida panhandle counties	Intervention - not self-care
Cummins_2016	Telephone Intervention for Pregnant Smokers: A Randomized Controlled Trial	Intervention - not self-care
Cypryk_2006	Evaluation of metabolic control in women with gestational diabetes mellitus by the continuous glucose monitoring system: a pilot study	Intervention - not self-care
Davey_2020	Emerging evidence from a systematic review of safety of pre-exposure prophylaxis for pregnant and postpartum women: where are we now and where are we heading?	Intervention - not self-care
Dellicour_2016	Effectiveness of the delivery of interventions to prevent malaria in pregnancy in Kenya	Intervention - not self-care
Desai_2018	Prevention versus treatment for malaria in pregnant women	Intervention - not self-care
deSousa_2019	Building trust to childbirth: Assessment of a nursing intervention programme	Intervention - not self-care
Dolatian_2010	The effect of reflexology on pain intensity of labor	Intervention - not self-care
Dowswell_2009	Transcutaneous electrical nerve stimulation (TENS) for pain management in labour	Intervention - not self-care
Doyle_2015	Home based educational intervention to improve perinatal outcomes for a disadvantaged community: a randomised control trial	Intervention - not self-care
El-Khouly_2017	Comparison of intravenous ferrous sucrose and oral ferrous sulphate in treatment of postpartum iron deficiency anemia	Intervention - not self-care
Evans_2021	A systematic review of supportive interventions to promote women's comfort and well-being during induction of labour	Intervention - not self-care
Facco_2019	Can We Use Home Sleep Testing for the Evaluation of Sleep Apnea in Obese Pregnant Women?	Intervention - not self-care
Fantinelli_2019	Assessment of psychological dimensions in telemedicine care for gestational diabetes mellitus: A systematic review of qualitative and quantitative studies	Intervention - not self-care
Fealy_2019	The Support for New Mums Project: A protocol for a pilot randomized controlled trial designed to test a postnatal psychoeducation smartphone application	Intervention - not self-care
Fenwick_2013	Study protocol for reducing childbirth fear: a midwife-led psycho-education intervention	Intervention - not self-care
Ferrara_2016	The Comparative Effectiveness of Diabetes Prevention Strategies to Reduce Postpartum Weight Retention in Women With Gestational Diabetes Mellitus: The Gestational Diabetes' Effects on Moms (GEM) Cluster Randomized Controlled Trial	Intervention - not self-care
Field_2013	Peer support and interpersonal psychotherapy groups experienced decreased prenatal depression, anxiety and cortisol	Intervention - not self-care
Firouzan_2020	The effect of midwifery led counseling based on Gamble's approach on childbirth fear and self-efficacy in nulligravida women	Intervention - not self-care
Ford_2001	Effects of a prenatal care intervention on the self-concept and self-efficacy of adolescent mothers	Intervention - not self-care
Ford_2002	Effects of a prenatal care intervention for adolescent mothers on birth weight, repeat pregnancy, and educational outcomes at one year postpartum	Intervention - not self-care
Foster_2016	Evaluating Acupuncture and Standard carE for pregnant women with Back pain (EASE Back): A feasibility study and pilot randomised trial	Intervention - not self-care
Fottrel_2013	The effect of increased coverage of participatory women's groups on neonatal mortality in Bangladesh: A cluster randomized trial	Intervention - not self-care
Fraser_1997	Randomized controlled trial of a prenatal vaginal birth after cesarean section education and support program Childbirth Alternatives Post-Cesarean Study Group	Intervention - not self-care
Garg_2015	A comparative study to evaluate the efficacy and safety of single dose intravenous iron carboxymaltose vs multidose iron sucrose in postpartum cases of severe iron deficiency anemia	Intervention - not self-care
Given_2013	Comparing patient-generated blood glucose diary records with meter memory in diabetes: a systematic review	Intervention - not self-care

Author/Year	Title	Reasons for exclusion
Given_2015	Tele-Mum: A Feasibility Study for a Randomized Controlled Trial Exploring the Potential for Telemedicine in the Diabetes Care of Those with Gestational Diabetes	Intervention - not self-care
Grady_2004	Pregnancy outcomes of adolescents enrolled in a CenteringPregnancy program	Intervention - not self-care
Green_1992	Telephone fetal heart rate monitoring in South Australia	Intervention - not self-care
Greve_2020	A pragmatic real-life study of flash glucose monitoring versus self-monitoring of blood glucose	Intervention - not self-care
Griffiths_2021	Interventions for preventing nausea and vomiting in women undergoing regional anaesthesia for caesarean section	Intervention - not self-care
Guittier_2009	Moxibustion for breech version: a randomized controlled trial	Intervention - not self-care
Guo_2019	Evaluating the effects of mobile health intervention on weight management, glycemic control and pregnancy outcomes in patients with gestational diabetes mellitus	Intervention - not self-care
Gutman_2020	A cluster randomized trial of delivery of intermittent preventive treatment of malaria in pregnancy at the community level in Burkina Faso	Intervention - not self-care
Gyorkos_2006	Lack of risk of adverse birth outcomes after deworming in pregnant women	Intervention - not self-care
Habib_2008	Outpatient cervical ripening with nitric oxide donor isosorbide mononitrate prior to induction of labor	Intervention - not self-care
Hajesmaeel-Gohari_2021	Virtual reality applications to assist pregnant women: a scoping review	Intervention - not self-care
Hanjani_2015	The Effect of Foot Reflexology on Anxiety, Pain, and Outcomes of the Labor in Primigravida Women	Intervention - not self-care
Haq_2005	Control of postpartum and post abortal haemorrhage with uterine packing	Intervention - not self-care
Heazell_2017	Can promoting awareness of fetal movements and focusing interventions reduce fetal mortality? A stepped-wedge cluster randomised trial (AFFIRM)	Intervention - not self-care
Hedderson_2018	A Tailored Letter Based on Electronic Health Record Data Improves Gestational Weight Gain Among Women With Gestational Diabetes Mellitus: The Gestational Diabetes' Effects on Moms (GEM) Cluster-Randomized Controlled Trial	Intervention - not self-care
Heuvel_2018	eHealth as the Next-Generation Perinatal Care: An Overview of the Literature	Intervention - not self-care
Hickling_1990	A non-pharmacological treatment of vascular headache during pregnancy	Intervention - not self-care
Holm_2019	Automated blood pressure self-measurement station compared to office blood pressure measurement for first trimester screening of pre-eclampsia	Intervention - not self-care
Holt_2017	Improving help-seeking for postnatal depression and anxiety: a cluster randomised controlled trial of motivational interviewing	Intervention - not self-care
Homko_2007	Use of an internet-based telemedicine system to manage underserved women with gestational diabetes mellitus	Intervention - not self-care
Homko_2012	Impact of a telemedicine system with automated reminders on outcomes in women with gestational diabetes mellitus	Intervention - not self-care
Horey_2004	Information for pregnant women about caesarean birth	Intervention - not self-care
Hughes_2018	Prediabetes in pregnancy, can early intervention improve outcomes? A feasibility study for a parallel randomised clinical trial	Intervention - not self-care
Ickovics_2011	Effects of group prenatal care on psychosocial risk in pregnancy: Results from a randomised controlled trial	Intervention - not self-care
Imhoff-Kunsch_2012	Antihelminthics in pregnancy and maternal, newborn and child health	Intervention - not self-care
Ivan_2015	Effect of Deworming on Disease Progression Markers in HIV-1-Infected Pregnant Women on Antiretroviral Therapy: A Longitudinal Observational Study From Rwanda	Intervention - not self-care
Izadirad_2018	Improving prenatal care in pregnant women in Iranshahr, Iran: Applying Health Belief Model	Intervention - not self-care
Jankowski_1987	Self-administered medications for obstetric patients	Intervention - not self-care
Jijimole_2018	Comparison of the effect of control, presence of a skilled birth attendant and reflexotherapy on labour outcomes in terms of pain, duration of labour and birth satisfaction among primigravid women-a pilot study	Intervention - not self-care
Johnsen_2018	Using eHealth to Increase Autonomy Supportive Care: a Multicenter Intervention Study in Antenatal Care	Intervention - not self-care
Johnson_2020	Computerized intervention for reducing intimate partner victimization for perinatal women seeking mental health treatment: A multisite randomized clinical trial protocol	Intervention - not self-care
Karaçam_2012	The Use of Perineal Massage in the Second Stage of Labor and Follow-Up of Postpartum Perineal Outcomes	Intervention - not self-care
Kellie_2020	Mechanical and surgical interventions for treating primary postpartum haemorrhage	Intervention - not self-care
Kelman_2018	A proof?of?concept pilot randomized comparative trial of brief Internet?based compassionate mind training and cognitive?behavioral therapy for perinatal and intending to become pregnant women	Intervention - not self-care
Kempe_1998	Home uterine activity monitoring in the prevention of very low birth weight reprinted from Public Health Reports, September/October 1997, Volume 112, published by the US Public Health Service	Intervention - not self-care
Kim_2019	Effects of a Web-Based Self-Management Program on the Behavior and Blood Glucose Levels of Women with Gestational Diabetes Mellitus	Intervention - not self-care
Krakowiak_2016	Home-Based HIV Testing Among Pregnant Couples Increases Partner Testing and Identification of Serodiscordant Partnerships	Intervention - not self-care
Kuo_2016	Auricular acupressure relieves anxiety and fatigue, and reduces cortisol levels in post-caesarean section women: a single-blind, randomised controlled study	Intervention - not self-care
Lane_2019	Real-Time Continuous Glucose Monitoring in Gestational Diabetes: A Randomized Controlled Trial	Intervention - not self-care
Larocque_2006	Should deworming be included in antenatal packages in hookworm-endemic areas of developing countries?	Intervention - not self-care
Larsen_2012	The Effectiveness of Childbirth Classes for Increasing Self-Efficacy in Women and Support Persons	Intervention - not self-care

For peer review only - http://bmjopen.bmj.com/site/about/guidelines.xhtml

Author/Year	Title	Reasons for exclusion
Lathrop_2014	A pilot study of prenatal care visits blended group and individual for women with low income	Intervention - not self-care
Li_2020	Internet-based intervention for postpartum depression in China ("Mommy go"): Protocol for a randomized controlled trial	Intervention - not self-care
Loayza_2011	Biofeedback for pain management during labour	Intervention - not self-care
Lumley_2006	Aiming to increase birth weight: a randomised trial of pre-pregnancy information, advice and counselling in inner-urban Melbourne	Intervention - not self-care
Luoba_2005	Earth-eating and reinfection with intestinal helminths among pregnant and lactating women in western Kenya	Intervention - not self-care
Ma_2011	Effects of Sanyinjiao (SP6) with electroacupuncture on labour pain in women during labour	Intervention - not self-care
Majewska_2021	Flash glucose monitoring in gestational diabetes mellitus: study protocol for a randomised controlled trial	Intervention - not self-care
Maloni_2010	Antepartum bed rest for pregnancy complications: Efficacy and safety for preventing preterm birth	Intervention - not self-care
Mark_2019	Male Partner Linkage to Clinic-Based Services for Sexually Transmitted Infections and Human Immunodeficiency Virus Services Following Couple Home-Based Education and Testing	Intervention - not self-care
Martin_2015	Providing Information and Support to Postnatal Women Who Have Experienced a Cesarean Section: A Pilot Study	Intervention - not self-care
Marwa_2019	The effects of HIV self-testing kits in increasing uptake of male partner testing among pregnant women attending antenatal clinics in Kenya: A randomized controlled trial	Intervention - not self-care
Mburu_2018	A model for predicting utilization of mHealth interventions in low-resource settings: case of maternal and newborn care in Kenya	Intervention - not self-care
McKellar_2017	Capture my mood: a feasibility study to develop a visual scale for women to self-monitor their mental wellbeing following birth	Intervention - not self-care
Meher_2005	Bed rest with or without hospitalisation for hypertension during pregnancy	Intervention - not self-care
Mendelson_2013	Impact of a preventive intervention for perinatal depression on mood regulation, social support, and coping	Intervention - not self-care
Mhajna_2020	Wireless, remote solution for home fetal and maternal heart rate monitoring	Intervention - not self-care
Milani_2015	The effects of postpartum home care on constipation and hemorrhoids at sixty days postpartum	Intervention - not self-care
Miller_2021	Reproducing fear: the effect of birth stories on nulligravid women's birth preferences	Intervention - not self-care
Mofid_2017	A Double-Blind Randomized Controlled Trial of Maternal Postpartum Deworming to Improve Infant Weight Gain in the Peruvian Amazon	Intervention - not self-care
Mohebbi_2019	Self-management intervention program based on the health belief model (Hbm) among women with gestational diabetes mellitus: A quazi-experimental study	Intervention - not self-care
Momenyan_2021	Immersive virtual reality analgesia in un-medicated laboring women (during stage 1 and 2): a randomized controlled trial	Intervention - not self-care
Monincx_2001	Maternal health, antenatal and at 8 weeks after delivery, in home versus in-hospital fetal monitoring in high-risk pregnancies	Intervention - not self-care
MooreSimas_2018	A Systematic Review of Integrated Care Interventions Addressing Perinatal Depression Care in Ambulatory Obstetric Care Settings	Intervention - not self-care
Morrison_2005	Women's health groups to improve perinatal care in rural Nepal	Intervention - not self-care
Mourad-Youssif_2010	Can the Non-pneumatic Anti-Shock Garment (NASG) reduce adverse maternal outcomes from postpartum hemorrhage? Evidence from Egypt and Nigeria	Intervention - not self-care
Msyamboza_2009	Community-based distribution of sulfadoxine-pyrimethamine for intermittent preventive treatment of malaria during pregnancy improved coverage but reduced antenatal attendance in southern Malawi	Intervention - not self-care
Mundell_2011	The impact of structured support groups for pregnant South African women recently diagnosed HIV positive	Intervention - not self-care
Nasso_2018	The Gestational Diabetes Group Program	Intervention - not self-care
Navaee_2015	Effect of role play education on primiparous women's fear of natural delivery and their decision on the mode of delivery	Intervention - not self-care
Ndibazza_2010	Effects of Deworming during Pregnancy on Maternal and Perinatal Outcomes in Entebbe, Uganda: A Randomized Controlled Trial	Intervention - not self-care
Ndibazza_2015	Effects of Deworming during Pregnancy on Maternal and Perinatal Outcomes in Entebbe, Uganda: A Randomized Controlled Trial	Intervention - not self-care
Nezamnia_2020	Effectiveness of cognitive-behavioral therapy on sexual function and sexual self-efficacy in pregnant women: An RCT	Intervention - not self-care
Norman_2018	Awareness of fetal movements and care package to reduce fetal mortality (AFFIRM): a stepped wedge, cluster-randomised trial	Intervention - not self-care
Ojengbede_2011	Assessing the role of the non-pneumatic anti-shock garment in reducing mortality from postpartum hemorrhage in Nigeria	Intervention - not self-care
Ondersma_2007	Computer-based brief intervention a randomized trial with postpartum women	Intervention - not self-care
Ondersma_2015	Computer-Delivered Screening and Brief Intervention forAlcohol Use in Pregnancy: A Pilot Randomized Trial	Intervention - not self-care
Orobaton_2016	Scaling-up the use of sulfadoxine-pyrimethamine for the preventive treatment of malaria in pregnancy: results and lessons on scalability, costs and programme impact from three local government areas in Sokoto State, Nigeria	Intervention - not self-care
Osoti_2015	Home-based HIV testing for men preferred over clinic-based testing by pregnant women and their male partners, a nested cross-sectional study	Intervention - not self-care
Osterman_2012	Effects of a motivational interviewing intervention to decrease prenatal alcohol use	Intervention - not self-care

For peer review only - http://bmjopen.bmj.com/site/about/guidelines.xhtml

Author/Year	Title	Reasons for exclusion
Palmer_2017	A retrospective cohort study of hospital versus home care for pregnant women with preterm prelabor rupture of membranes	Intervention - not self-care
Pantoja_2015	Oxytocin for preventing postpartum haemorrhage (PPH) in non-facility birth settings	Intervention - not self-care
Penn_2018	Non?pneumatic anti?shock garment (NASG) as a first aid for preventing or reversing hypovolemic shock secondary to obstetric hemorrhage	Intervention - not self-care
Perichart-Perera_2009	A medical nutrition therapy program improves perinatal outcomes in Mexican pregnant women with gestational diabetes and type 2 diabetes mellitus	Intervention - not self-care
Pikee_2021	New Innovation: Use of Flash Glucose Monitoring for Evaluating Glycaemic Variability, Patient Satisfaction and Clinical Utility in Pregnant Women with Diabetes	Intervention - not self-care
Raams_2018	Task shifting in active management of the third stage of labor: A systematic review	Intervention - not self-care
Redulla_2018	Bed rest with and without hospitalization in multiple pregnancy for improving perinatal outcomes	Intervention - not self-care
Reid_2002	A two-centred pragmatic randomised controlled trial of two interventions of postnatal support	Intervention - not self-care
Runjati_2020	The effect of postpartum coping skill classes (PCSC) on stress level, cortisol levels, maternal self-efficacy, and baby's growth and development In Semarang, Central Java	Intervention - not self-care
Ryo_2012	Fetal movement counting at home with a fetal movement acceleration measurement recorder: A preliminary report	Intervention - not self-care
Salam_2021	Effect of mass deworming with antihelminthics for soil?transmitted helminths during pregnancy	Intervention - not self-care
Sandeford_2021	Protocol for probiotic therapy vs placebo for preterm prelabour rupture of membranes to prolong pregnancy duration (Pro-PPROM) trial	Intervention - not self-care
Sathe_2016	Procedures and Uterine-Sparing Surgeries for Managing Postpartum Hemorrhage: A Systematic Review	Intervention - not self-care
Schachman_2004	Baby boot camp: facilitating maternal role adaptation among military wives	Intervention - not self-care
Scott_2018	Accuracy, User Acceptability, and Safety Evaluation for the FreeStyle Libre Flash Glucose Monitoring System When Used by Pregnant Women with Diabetes	Intervention - not self-care
Sekhavatpour_2020	The effect of spiritual self-care training on the quality of life of mothers of preterm infants: A randomized controlled trial	Intervention - not self-care
Senoun_2014	Planned home versus hospital care for preterm prelabour rupture of the membranes (PPROM) prior to 37 weeks' gestation	Intervention - not self-care
Shah_1993	Evaluation of the home-based maternal record: a WHO collaborative study	Intervention - not self-care
Shirvani_2021	Importance of childbirth preparation classes in improving childbirth self-efficacy and reducing worry in primigravidas	Intervention - not self-care
Shirzad_2020	Effect of "motivational interviewing" and "information, motivation, and behavioral skills" counseling interventions on choosing the mode of delivery in pregnant women: a study protocol for a randomized controlled trial	Intervention - not self-care
Snell_2006	An exploratory study in the UK of the effectiveness of three different pain management regimens for post-caesarean section women	Intervention - not self-care
Solhi_2019	Effect of Health Literacy Education on Self-Care in Pregnant Women: A Randomized Controlled Clinical Trial	Intervention - not self-care
Sosa_2001	Bed rest in singleton pregnancies for preventing preterm birth	Intervention - not self-care
Stamp_1995	Evaluation of antenatal and postnatal support to overcome postnatal depression: a randomized, controlled trial	Intervention - not self-care
Stock_2014	Home cervical ripening with dinoprostone gel in nulliparous women with singleton pregnancies	Intervention - not self-care
Stotts_2002	One-to-One A motivational intervention for resistant pregnant smokers	Intervention - not self-care
Stotts_2004	Impact of a motivational intervention on mechanisms of change in low-income pregnant smokers	Intervention - not self-care
Sultan_2019	Oral vs intravenous iron therapy for postpartum anemia: a systematic review and meta-analysis	Intervention - not self-care
Svahn-Ekdahl_2021	Maintenance of physical activity level, functioning and health after non-pharmacological treatment of pelvic girdle pain with either transcutaneous electrical nerve stimulation or acupuncture: A randomised controlled trial	Intervention - not self-care
Tandon_2014	Six-month outcomes from a randomized controlled trial to prevent perinatal depression in low-income home visiting clients	Intervention - not self-care
Tappin_2005	Randomised controlled trial of home based motivational interviewing by midwives to help pregnant smokers quit or cut down	Intervention - not self-care
Thayer_2017	Effects of deworming on child and maternal health: a literature review and meta-analysis	Intervention - not self-care
Thomas_2004	Effectiveness of a comprehensive psychoeducational intervention with pregnant and parenting adolescents: A pilot study	Intervention - not self-care
Thome_2013	Evaluation of a family nursing intervention for distressed pregnant women and their partners: A single group before and after study	Intervention - not self-care
Thomson_2016	Gestational Weight Gain: results from the Delta Healthy Sprouts Comparative Impact Trial	Intervention - not self-care
Toohill_2014	A randomized controlled trial of a psycho-education intervention by midwives in reducing childbirth fear in pregnant women	Intervention - not self-care
Torlesse_2001	Albendazole therapy and reduced decline in haemoglobin concentration during pregnancy (Sierra Leone)	Intervention - not self-care
Trevillion_2020	An exploratory parallel-group randomised controlled trial of antenatal Guided Self-Help (plus usual care) versus usual care alone for pregnant women with depression: DAWN trial	Intervention - not self-care
Triebwasser_2020	Successful implementation of text-based blood pressure monitoring for postpartum hypertension	Intervention - not self-care
Tripathy_2016	Effect of participatory women's groups facilitated by Accredited Social Health Activists on birth outcomes in rural eastern India: a cluster-randomised controlled trial	Intervention - not self-care

For peer review only - http://bmjopen.bmj.com/site/about/guidelines.xhtml

Author/Year	Title	Reasons for exclusion
Troy_2003	The effectiveness of a self-care intervention for the management of postpartum fatigue	Intervention - not self-care
Turan_2018	Development and Piloting of a Home-Based Couples Intervention During Pregnancy and Postpartum in Southwestern Kenya	Intervention - not self-care
Turawa_2020	Interventions for preventing postpartum constipation	Intervention - not self-care
Urquhart_2017	Home uterine monitoring for detecting preterm labour	Intervention - not self-care
van-der-Ploeg_1996	Transcutaneous nerve stimulation (TENS) during the first stage of labour: a randomized clinical trial	Intervention - not self-care
Varnfield_2021	MTHer, an mHealth System to Support Women with Gestational Diabetes Mellitus: Feasibility and Acceptability Study	Intervention - not self-care
Verma_2011	Intravenous iron therapy versus oral iron in postpartum patients in rural area	Intervention - not self-care
Voormolen_2018	Continuous glucose monitoring during diabetic pregnancy (GlucoMOMS): a multicentre randomized controlled trial	Intervention - not self-care
Walia_2020	Effect of sharing health messages on antenatal care behavior among women involved in microfinance-based self-help groups in Bihar India	Intervention - not self-care
Walia_2021	Routine deworming during antenatal care decreases risk of neonatal mortality and low birthweight: A retrospective cohort of survey data	Intervention - not self-care
Wapner_1995	A randomized multicenter trial assessing a home uterine activity monitoring device used in the absence of daily nursing contact	Intervention - not self-care
Watson_1990	Management of preterm labor patients at home: does daily uterine activity monitoring and nursing support make a difference?	Intervention - not self-care
Webb_2012	The effect of anthelmintic treatment during pregnancy on HIV plasma viral load: results from a randomized, double-blind, placebo-controlled trial in Uganda	Intervention - not self-care
Wei_2016	Effect of a CGMS and SMBG on Maternal and Neonatal Outcomes in Gestational Diabetes Mellitus: a Randomized Controlled Trial	Intervention - not self-care
Willcox_2011	Is parasite clearance clinically important after malaria treatment in a high transmission area? A 3-month follow-up of home-based management with herbal medicine or ACT	Intervention - not self-care
Williams_2012	Associations between preconception counseling and maternal behaviors before and during pregnancy	Intervention - not self-care
Witt_2016	Therapeutic Breast Massage in Lactation for the Management of Engorgement, Plugged Ducts, and Mastitis	Intervention - not self-care
Witteveen_2020	Effectiveness of a guided ACT-based self-help resilience training for depressive symptoms during pregnancy: Study protocol of a randomized controlled trial embedded in a prospective cohort	Intervention - not self-care
Wyck_2007	Intravenous Ferric Carboxymaltose Compared With Oral Iron in the Treatment of Postpartum Anemia: A Randomized Controlled Trial	Intervention - not self-care
Xiu_2020	Psychological intervention for patients with high-risk pregnancy can improve their self-management ability and pregnancy outcome	Intervention - not self-care
Yang_2018	Medical nutrition treatment of women with gestational diabetes mellitus by a telemedicine system based on smartphones	Intervention - not self-care
Yu_2019	Application and Utility of Continuous Glucose Monitoring in Pregnancy: A Systematic Review	Intervention - not self-care
Zaharieva_2020	Continuous Glucose Monitoring Versus Self-Monitoring of Blood Glucose to Assess Glycemia in Gestational Diabetes	Intervention - not self-care
Zaharieva_2021	Continuous Glucose Monitoring Versus Self-Monitoring of Blood Glucose to Assess Glycemia in Gestational Diabetes	Intervention - not self-care
Zandinava_2017	Effect of educational package on Self-Care behavior, quality of life, and blood glucose levels in pregnantwomen with gestational diabetes: A randomized controlled trial	Intervention - not self-care
Zhang_2021	The effect of individualized exercise prescriptions combined with dietary management on blood glucose in the second-and-third trimester of gestational diabetes mellitus	Intervention - not self-care
Abobaker_2020	Study the effect of non-pharmaceutical approach on traumatic nipple for lactating mothers	No full text available
Abraham-Justice_2010	The effects of a postpartum education program on self-care and healthcare-seeking behaviors in mothers	No full text available
Afshar_2012	Effect of a sex educational package on sexual function in pregnant women: a multicenter randomized controlled trial	No full text available
Agot_2018	Secondary distribution of HIV self-tests as a way to promote HIV testing among male partners of young women: subgroup analysis from a randomized trial	No full text available
Alipour_2017	The effectiveness of relaxation techniques on depression, anxiety and stress in pregnant women: based on self-efficacy theory	No full text available
Arpin_2007	Evaluation of pain relief as well as nurses' and mothers' satisfaction following the implementation of an automedication program in obstetrics	No full text available
Artal_1996	Home uterine activity monitoring and its role in reducing the incidence of prematurity	No full text available
Askari_2016	Effect of back massage with sesames oil on pain and length of delivery in primiparous women	No full text available
Asklund_2019	The use of an app with a pfmt programme among pregnant and postnatal women for preventive use and treatment of urinary incontinence	No full text available
Attanayake_2016	Cervical ripening with self administered iso sorbide mononitrate vaginally, in uncomplicated singleton pregnancies at 39 weeks gestation: a double blind randomised controlled trial	No full text available
BabaDizavandi_2012	The effect of music therapy on labour pain in nulliparous women in mashad, Iran in 2010	No full text available
Bahri_2015	Educational intervention to improve oral health beliefs and behaviors during pregnancy: a randomized-controlled trial	No full text available
Bais_2018	A randomized, double-blind controlled clinical trial of light therapy for pregnant women with major depressive disorder	No full text available

For peer review only - http://bmjopen.bmj.com/site/about/guidelines.xhtml

Author/Year	Title	Reasons for exclusion
Barfoot_2015	A pilot randomised trial of 4 physiotherapy interventions for pregnancy related pelvic girdle pain	No full text available
Beeharry_2017	A multimedia approach to diabetes structured education: the effects of "Keeping Healthy After Gestational Diabetes (GDM)", a multimedia based education program on patient knowledge, self-efficacy and acceptability	No full text available
Berry_2015	A Postpartum Community-Based Weight Management Intervention Designed for Low-Income Women: Feasibility and Initial Efficacy Testing	No full text available
Bertz_2010	Dietary restriction or dietary restriction and exercise, but not exercise intervention alone, reduces weight and fat mass in overweight and obese women postpartum	No full text available
Bjorn_2013	Relaxation strategies during pregnancy Health benefits	No full text available
Blondel_1992	Home uterine activity monitoring in France: a randomized, controlled trial	No full text available
Buhling_2004	Introductory experience with the continuous glucose monitoring system (CGMS; Medtronic Minimed) in detecting hyperglycemia by comparing the self-monitoring of blood glucose (SMBG) in non-pregnant women and in pregnant women with impaired glucose tolerance	No full text available
Bung_1993	[Regular exercise therapy in disorders of carbohydrate metabolism in pregnancy--results of a prospective, randomized longitudinal study]	No full text available
Burke_2019	Feasibility and acceptability of home use of a smartphone-based urine testing application among women in prenatal care	No full text available
Cairns_2017	A novel self-management intervention for adjustment of postnatal antihypertensive treatment	No full text available
Cairns_2017	Self-management of postnatal antihypertensive treatment: a pilot randomised controlled trial	No full text available
Carissoli_2017	Enhancing psychological wellbeing of women approaching the childbirth: A controlled study with a mobile application	No full text available
Cohn_2014	Mind in Labor: Effects of Mind/Body Training on Childbirth Appraisals and Pain Medication Use During Labor	No full text available
Das_2015	Effect of a behavioral intervention with cereal fiber or resistant starch on gestational weight gain: a randomized clinical trial	No full text available
DavimRMB_2008	Non pharmacological strategies evaluation in parturients' pain relief	No full text available
DavimRMB_2008	Showering as a non pharmacological strategy to relief the parturients pain	No full text available
Dyson_1988	The role of home uterine monitoring in the prevention of preterm birth in high risk patients	No full text available
Dyson_1990	Preterm birth prevention - the role of education - palpation vs home uterine monitoring in non-multiple gestations	No full text available
Dyson_1991	Prevention of preterm birth in high-risk patients: the role of education and provider contact versus home uterine monitoring	No full text available
El-Refaey_2020	The effect of tailored psycho-educational program on pregnant women's anxiety and knowledge about self-care management regarding minor discomforts	No full text available
Epp_2019	A Review of the Effects of Physical Therapy on Self-Esteem in Postpartum Women With Lumbopelvic Dysfunction	No full text available
Ershoff_1990	Pregnancy and medical cost outcomes of a self-help prenatal smoking cessation program in an HMO	No full text available
Ershoff_1995	Relapse prevention among women who stop smoking early in pregnancy: A randomized clinical trial of a self-help intervention	No full text available
Eva_2021	Virtual reality in obstetrics as a non pharmacological method	No full text available
Evans_2020	Non-pharmacological interventions to reduce the symptoms of mild to moderate anxiety in pregnant women A systematic review and narrative synthesis of women's views on the acceptability of and satisfaction with interventions	No full text available
Evans_2020	Developing a complex intervention to support pregnant women with mild to moderate anxiety: application of the Medical Research Council framework	No full text available
Evies_2007	Effects of education for self-care on the metabolic control of pregnant diabetic patients	No full text available
Fakhri_2017	Effect of education based on health belief model with relaxation on anxiety of nulliparouse women	No full text available
Faraji_2016	The impact of supplementation with fish oil on lipid profile of pregnant mothers: a randomized controlled trial	No full text available
Farajnejad_2020	Aromatherapy in postpartum depression: a clinical trial based on precede model	No full text available
Fawcett_1993	Effects of information on adaptation to cesarean birth	No full text available
Feingold_1993	Contribution of home uterine activity monitoring to the reduction of preterm delivery rate	No full text available
Frye_2015	On the trail of misoprostol in the community: a secondary analysis of self-administered misoprostol for the prevention of postpartum hemorrhage in Uganda	No full text available
Geelhoed-Duijvestijn_2016	Real-time continuous glucose monitoring during pregnancy in women with type 1 diabetes: is RTCGM related to better glycaemic control and key obstetric outcomes?	No full text available
Ghasemi_2018	The effect of group counseling with cognitive-behavioral approach on self-efficacy of pregnant women's choice of vaginal delivery	No full text available
Gookin_1994	Randomized comparison of home uterine activity monitoring and routine care in patients discharged after treatment for preterm labor	No full text available
Greene_1999	A postpartum self-medication program: Effect on narcotic use	No full text available
Grimes_1992	Randomized controlled trials of home uterine activity monitoring: a review and critique	No full text available
Guerra_2005	Educational for self care of pregnant diabetic patients	No full text available
Hannover_2004	Interventions to prevent health risks due to tobacco smoke in pregnant women, women postpartum and their infants	No full text available

Author/Year	Title	Reasons for exclusion
Hanson_1984	Self-monitoring of blood glucose by diabetic women during the third trimester of pregnancy	No full text available
Hartmann_1996	A randomized controlled trial of smoking cessation intervention in pregnancy in an academic clinic	No full text available
Henrique_2018	Pain, anxiety, and stress during childbirth-management without medication	No full text available
Hill_1990	Home uterine activity monitoring is associated with a reduction in preterm birth	No full text available
Hjalmarson_1991	Stopping smoking in pregnancy: effect of a self-help manual in controlled trial	No full text available
Hodnett_1999	Home?based social support for socially disadvantaged mothers	No full text available
Holden_2016	Yoga for pregnancy related back pain	No full text available
Hollingsworth_1987	Postprandial walking exercise in pregnant insulin-dependent (type I) diabetic women: reduction of plasma lipid levels but absence of a significant effect on glycemic control	No full text available
Horio_1998	Fetal monitor for non-stress-test screening at home	No full text available
Howell_2011	Mothers avoiding depression through empowerment intervention trial (made it)	No full text available
Hoyme_1998	Reduction of prematurity by pH-screening	No full text available
Hsieh_2017	Efficacy of Warm Showers on Postpartum Fatigue Among Vaginal-Birth Taiwanese Women: A Quasi-Experimental Design	No full text available
Huddle_1987	Diabetes in pregnancy: The use of home blood glucose monitoring and intensive monitoring to ensure favourable perinatal outcome	No full text available
Iams_1987	A prospective random trial of home uterine activity monitoring in pregnancies at increased risk of preterm labor	No full text available
Iams_1988	A prospective random trial of home uterine activity monitoring in pregnancies at increased risk of preterm labor Part II	No full text available
Iams_1992	Randomized controlled trials of home uterine activity monitoring: a review and critique	No full text available
Iams_1995	Current status of home uterine activity monitoring	No full text available
Igarashi_2010	Effects of aromatherapy for self-care during pregnancy	No full text available
Jack_2019	Effect of Addition of an Intimate Partner Violence Intervention to a Nurse Home Visitation Program on Maternal Quality of Life: a Randomized Clinical Trial	No full text available
Jaqin_2019	Differences in self-efficacy before and after antenatal education	No full text available
Jeon_2018	Effects of an educational program on knowledge and Efficacy of self-care during pregnancy among marriage immigrant women in South Korea	No full text available
Johnson_2000	Preventing smoking relapse in postpartum women	No full text available
Johnston_2006	The Community Perinatal Care Study: home visiting and nursing support for pregnant women	No full text available
Kaplan_2011	The impact of a behavioral intervention on weight gain during pregnancy	No full text available
Kavanagh_2020	Does an online wellbeing program (Baby Steps) prvent perinatal distress in first-time fathers and mothers?	No full text available
Kaveh_2012	Impact of Education on Nutrition and Exercise on the Level of Knowledge and Metabolic Control Indicators (FBS & PPBS) of Gestational Diabetes Mellitus (GDM) Patients	No full text available
Knuppel_1990	Preventing preterm birth in twin gestation: home uterine activity monitoring and perinatal nursing support	No full text available
Kyung_2003	Effects of postpartum care program for primiparous women and care-givers on the knowledge and behavior of postpartum care and postpartum recovery in primiparous women	No full text available
Lara_2010	Retention rates and potential predictors in a longitudinal randomized control trial to prevent postpartum depression	No full text available
Larsen_2001	A prospective study of self-efficacy expectancies and labour pain	No full text available
Lee_2018	Implementation and evaluation of gestational diabetes management using mobile health care service-a pilot study	No full text available
Lehugeur_2017	Non-Pharmacological Management Of Relief In Deliveries Assisted By An Obstetric Nurse	No full text available
Lillington_1995	Evaluation of a smoking cessation program for pregnant minority women	No full text available
Lipatov_2019	Possibility of non-pharmacological and pharmacological therapies for placental insufficiency	No full text available
Liu_2010	Effects of music therapy on labour pain and anxiety in Taiwanese first-time mothers	No full text available
Lombard_2011	A randomized controlled trial investigating self-weighing and the prevention of excess weight gain in early pregnancy	No full text available
Lowe_1998	Evaluation of antenatal smoking cessation programs for pregnant women	No full text available
Lumluk_2011	Effect of antenatal education for better self-correct diagnosis of true labor: a randomized control study	No full text available
Luo_2015	Influence of Orem self-care model on birth outcomes of gestational diabetes mellitus patients	No full text available
Luoto_2012	Physical activity and dietary counseling and supervised group exercise for first-time pregnant women - a feasibility study of a controlled trial	No full text available
Maloni_1998	Prescription of activity restriction to treat high-risk pregnancies	No full text available
Marquez-Sterling_2000	Physical and psychological changes with vigorous exercise in sedentary primigravidae	No full text available
Millan-Ferro_2017	Participation of latinos with type 2 diabetes in a culturally and linguistically oriented virtual diabetes self-care and education program	No full text available
Miller_2004	Improving the identification and treatment of postpartum depression in a managed care organization	No full text available
Mirzakhani_2015	The effect of exercise with special ball during pregnancy on duration of active phase of first stage of birth in nulliparous women	No full text available
Mohammadi_2021	The effect of pregnancy training classes based on bandura self-efficacy theory on postpartum depression and anxiety and type of delivery	No full text available

For peer review only - http://bmjopen.bmj.com/site/about/guidelines.xhtml

Author/Year	Title	Reasons for exclusion
Morrison_2003	Current status of home uterine activity monitoring	No full text available
Mortazavi_2021	Effectiveness of solution-focused counseling therapy on pregnancy anxiety and fear of childbirth: A randomized clinical trial	No full text available
Mou_1991	Multicenter randomized clinical trial of home uterine activity monitoring for detection of preterm labor	No full text available
Mullen_1999	Maternal smoking during pregnancy and evidence-based intervention to promote cessation	No full text available
Naef_1998	Home blood pressure monitoring for pregnant patients with hypertension	No full text available
Nagey_1993	Randomized comparison of home uterine activity monitoring and routine care in patients discharged after treatment for preterm labor	No full text available
Nehbandani_2018	Comparison the effects of aromatherapy with rose extract and lavender on the pain of the active phase of labor in primipara women	No full text available
Newman_1988	Randomized trial of home uterine activity monitoring	No full text available
Norheim_2001	Acupressure treatment of morning sickness in pregnancy A randomised, double-blind, placebo-controlled study	No full text available
O'Connor_1992	Effectiveness of a pregnancy smoking cessation program	No full text available
Pasinlioglu_2004	Health education for pregnant women: The role of background characteristics	No full text available
Pawalia_2018	Behavioral Intervention during Pregnancy for Preventing Abdominal Obesity and Pregnancy Complications in Indian Women: Protocol for a Randomised Controlled Trial	No full text available
Petersen_1992	Smoking reduction during pregnancy by a program of self-help and clinical support	No full text available
Pont_2019	Prenatal yoga program on primigravida 3rd trimester reducing complaints in the first stage and self-efficacy in the labor	No full text available
Pormosayebi_2018	The effect of training based on health belief model (HBM) in preventing exposure to polluted air in pregnant women	No full text available
Posadas_2014	Intake of vitamin C, probiotics, flavonoids and nutritional status in pregnant women with urinary tract infection	No full text available
Pratt_2016	Effect of a brief educational intervention concerning the human papillomavirus on the knowledge and attitudes of antenatal patients in New Providence, The Bahamas	No full text available
Ramezanpoor_2019	The effectiveness of an educational intervention based on social cognitive theory on fruit and vegetable intake in pregnant women	No full text available
Ratner_2000	Twelve-month follow-up of a smoking relapse prevention intervention for postpartum women	No full text available
Rees_1995	Effect of relaxation with guided imagery on anxiety, depression, and self-esteem in primiparas	No full text available
Reichmann_2009	Home uterine activity monitoring: an evidence review of its utility in multiple gestations	No full text available
Reyhani_2014	Investigating the effects of spiritual self-care training on psychological stress of mothers with preterm infants admitted in neonatal intensive care unit	No full text available
Rezaeean_2017	Application of Orem's theory for promotion of self-care behaviors of pregnant women at risk for preterm delivery: A clinical trial	No full text available
Sanyal_2019	Management of gestational diabetes in a resource-limited setting in India	No full text available
Seto_2019	The Accuracy of Self-Screening of Group B Streptococcus in Pregnant Women-A Randomized Crossover Study	No full text available
Shobeiri_2016	The effect of orem's self care model on control of preeclampsia in pregnant women: A randomized clinical trial	No full text available
Smith_1990	Reliability of compact electronic blood pressure monitors for hypertensive pregnant women	No full text available
Smith_1992	Patient's acceptance of monitoring fetal movement: A randomized comparison of charting techniques	No full text available
Smith_1993	Educational efforts in preventing preterm delivery among inner city adolescents	No full text available
Smith_1994	Reduction of preterm births in a low-income population utilizing home uterine activity monitoring	No full text available
Sunderji_1991	Earlier detection of preterm labor: multicenter prospective randomized clinical trial of home uterine activity monitoring	No full text available
Swaroopa_2017	Effect of antenatal education in improving maternal confidence and reducing anxiety about labor in primigravida women attending saveetha medical college and hospital,chennai,tamil nadu, india	No full text available
Taheri_2021	The effect of health belief-based education on physical activates of nulliparous women: a randomized control trial	No full text available
Tian_2013	Effects of birth ball exercises on labor pain and childbirth satisfaction	No full text available
Unger_2015	Mobile wach: developing and evaluating a humancomputer hybrid mobile messaging system for women's and children's health in Kenya	No full text available
Valbo_1994	Smoking cessation in pregnancy: The effect of self-help manuals	No full text available
Vasaiya_2020	Comparison between Effect of Foot Exercise and Warm Water Foot Soak on Foot Edema among Antenatal Women	No full text available
Wahyuni_2020	The effect of early mobilization towards self-care levels in post-sectio caesarea mother with self-care theory approach	No full text available
Wardani_2018	The effectiveness of acupressure at LI 4 and SP 6 point on uterine contraction in the first stage of labor on primiparous women	No full text available
Wilson_2020	Making BUMP work: intervention development in a large randomised controlled trial of blood pressure self-monitoring in pregnancy	No full text available
Wing_2010	Comparison of urinary cytokines after ingestion of cranberry juice cocktail in pregnant subjects: a pilot study	No full text available

For peer review only - http://bmjopen.bmj.com/site/about/guidelines.xhtml

Author/Year	Title	Reasons for exclusion
Wirz-Justice_2011	A randomized, double-blind, placebo-controlled study of light therapy for antepartum depression	No full text available
Yildirim_2004	The effect of breathing and skin stimulation techniques on labour pain perception of Turkish women	No full text available
Zardosht_2021	Effect of chamomile oil on cesarean section pain in primiparous women: a randomized clinical trial	No full text available
Zeng_2008	A research on relationship between fetal growth restriction and vitamin nutritional status in late pregnancy	No full text available
Ainscough_2016	Impact of an mHealth supported healthy lifestyle intervention on behavioural stage of change in overweight and obese pregnancy	Not a peer-reviewed journal article
Al-Hashmi_2017	The Effectiveness of a Health Education Intervention on Self-Efficacy for Adherence to Healthy Behaviors among Women with Gestational Diabetes	Not a peer-reviewed journal article
Andalib_2016	Evaluation of the effect of corrected nutrition on reduction of stress in gestational diabetes	Not a peer-reviewed journal article
Apay_2015	The Effect of the Care Given Using Orem's Self-Care Model on the Postpartum Self- Evaluation	Not a peer-reviewed journal article
Barrett_2017	Home Monitoring of Fasting and Postprandial Triglycerides in Late Pregnancy: A Pilot Study	Not a peer-reviewed journal article
Brixval_2016	Antenatal education in small classes may increase childbirth self-efficacy: Results from a Danish randomised trial	Not a peer-reviewed journal article
Buckingham-Schutt_2019	The Behavioral Wellness in Pregnancy Study: a theory-based multi-component intervention to promote appropriate weight gain and healthy lifestyle behaviors in previously sedentary pregnant women	Not a peer-reviewed journal article
Butler_2019	Assessing and managing risk online: a clinician's perspective on internet-delivered intervention, 'MUMentum', for perinatal distress, anxiety, and depression	Not a peer-reviewed journal article
Chappell_2021	Self-monitoring of blood pressure in women with pregnancy hypertension: the BUMP2 multicentre randomised controlled trial	Not a peer-reviewed journal article
Chaudhry_2020	Development and assessment of smartphone based intervention (TechMotherCare) for maternal depression in Pakistan	Not a peer-reviewed journal article
Cheung_2011	A pilot structured behavioural intervention trial to increase physical activity among women with recent gestational diabetes	Not a peer-reviewed journal article
Chiarelli_2002	A physiotherapist-led programme of postpartum pelvic floor exercises reduced urinary incontinence at 3 months	Not a peer-reviewed journal article
Dahlen_2011	Moxibustion for cephalic version: a feasibility study	Not a peer-reviewed journal article
Dal_2021	The effect of pregnancy yoga on breastfeeding self-efficacy and prenatal attachment: A semi-experimental study	Not a peer-reviewed journal article
Dias_2011	Effectiveness of perineal exercises in controlling urinary incontinence and improving pelvic floor muscle function during pregnancy	Not a peer-reviewed journal article
Draffin_2013	Gestational diabetes: things you need to know (but maybe don't): development of an educational DVD for women recently diagnosed with gestational diabetes	Not a peer-reviewed journal article
Durmaz_2016	The Self-Care Training is Increasing Body Image Perception Self- Esteem and Self-Care Abilities in Pregnant Adolescents: A Pretest- Posttest Quasi-Experimental Study	Not a peer-reviewed journal article
Ericksen_2020	Treatment for antenatal anxiety and depression with Beating the Blues before Birth BBB© positively impacts infant postnatal development at 9 months, a pilot RCT	Not a peer-reviewed journal article
ErtekinPinar_2016	Effects of coping with stress training on pregnant women's depression, stress and coping with stress levels	Not a peer-reviewed journal article
Fancourt_2017	Associations between singing to babies and symptoms of postnatal depression, wellbeing, self-esteem and mother-infant bond	Not a peer-reviewed journal article
Farrell_2013	The use of logs and forms for the tracking of RT-CGM devices in the conceptt trial	Not a peer-reviewed journal article
Fox_2021	Short term postpartum blood pressure management and long-term blood pressure control: a randomised controlled trial	Not a peer-reviewed journal article
Freeman_1986	Randomised trial of self hypnosis for analgesia in labour	Not a peer-reviewed journal article
Goldberg_1986	Gestational diabetes: impact of home glucose monitoring on neonatal birth weight	Not a peer-reviewed journal article
Gunther_2019	The effect of lifestyle counselling on dietary behavior of pregnant women - Secondary results of the randomised controlled GeliS trial	Not a peer-reviewed journal article
Hadar_2021	Mobile Self-Operated Home Ultrasound System for Remote Fetal Assessment During Pregnancy	Not a peer-reviewed journal article
Harrison_2014	How effective is self-weighing in the setting of a lifestyle intervention to reduce gestational weight gain and postpartum weight retention?	Not a peer-reviewed journal article
Herring_2017	Intervening during and after pregnancy to prevent weight retention among African American women	Not a peer-reviewed journal article
Hill_2012	Nutrient intake of pregnant women with type 1 diabetes in the DAPIT Trial; relationships with anthropometry and glycaemic control	Not a peer-reviewed journal article
Hoffmann_2020	Does an antenatal lifestyle intervention in routine care improve maternal and infant health outcomes in the first year postpartum-12 months follow-up of the cluster-randomised GeliS trial	Not a peer-reviewed journal article
Hoirisch-Clapauch_2016	A protocol combining daily walking and a lowglycemic index diet increases the rate of take-home babies in women with consecutive first-trimester miscarriages	Not a peer-reviewed journal article
Houshmandpour_2019	The effect of midwife-oriented group counseling, based on orem's model on self-care and empowerment, in primiparous women: A clinical trial	Not a peer-reviewed journal article

For peer review only - http://bmjopen.bmj.com/site/about/guidelines.xhtml

Author/Year	Title	Reasons for exclusion
Howharn_2008	Effects of childbirth preparation classes on self-efficacy in coping with labor pain in Thai primiparas	Not a peer-reviewed journal article
Hoyme_2002	Results and potential consequences of the thuringia prematurity preventional campaign 2000	Not a peer-reviewed journal article
Hunt_2018	Evaluation of a home-use oral glucose tolerance test kit for screening for gestational diabetes	Not a peer-reviewed journal article
Hutchesson_2019	Be Healthe for your Heart Study Protocol: preventing cardiovascular disease among women with a history of pre-eclampsia	Not a peer-reviewed journal article
Ip_2005	The effect of a theory-based intervention on promoting self-efficacy for childbirth among pregnant women in Hong Kong	Not a peer-reviewed journal article
Keytash_2015	Impact of preconception care on overweight/obese women: long-term effects of a behavioural change intervention	Not a peer-reviewed journal article
Kirop_2021	Feasibility and Acceptability of a Telephone Support Intervention During Early Postnatal Period Among Teenage Mothers in Western Kenya: A Pilot Randomised Controlled Trial	Not a peer-reviewed journal article
Kisa_2019	Repeat HIV testing of individuals with discrepant HIV self-test results in Central Uganda	Not a peer-reviewed journal article
Lee_2016	Did self-sampling improve the adherence to group B streptococci screening in pregnant women?	Not a peer-reviewed journal article
Lineberger_1987	Pregnant adolescents attending prenatal parent education classes: self-concept, anxiety and depression levels	Not a peer-reviewed journal article
Loukopoulou_2012	Urinary nicotine and cotinine as a method for assessing the effectiveness of an intervention to aid smoking cessation during pregnancy	Not a peer-reviewed journal article
Makvandi_2018	Effect of normal physiologic childbirth program in mother-friendly hospitals on duration of labor	Not a peer-reviewed journal article
Mayer_1990	A randomized evaluation of smoking cessation interventions for pregnant women at a WIC clinic	Not a peer-reviewed journal article
McGready_2001	A double-blind randomized therapeutic trial of insect repellents for the prevention of malaria in pregnancy	Not a peer-reviewed journal article
McManus_2017	The snap-HT trial: self-management of antihypertensive medication postpartum - Can women do it better?	Not a peer-reviewed journal article
McManus_2018	109 Using blood pressure selfmonitoring in pregnancy: A systematic review and individual patient data metaanalysis	Not a peer-reviewed journal article
Miquelutti_2012	Evaluation of the efficacy of an antenatal birth preparation program	Not a peer-reviewed journal article
Mirghafourvand_2019	Effectiveness of self-care training on pregnancy consequences in gestational diabetes: A randomized controlled clinical trial	Not a peer-reviewed journal article
Mollart_2019	Are women interested in Acupressure to increase spontaneous onset of labour?	Not a peer-reviewed journal article
Montazeri_2020	The effect of writing therapy on anxiety in pregnant women: a randomized controlled trial	Not a peer-reviewed journal article
Murphy_2016	Asthma self-management education and inhaled corticosteroid use during pregnancy and postpartum from 2004 to 2014	Not a peer-reviewed journal article
Neoh_2017	The conceptt-diet study: an analysis of diet and glycaemia in women with type 1 diabetes before and during pregnancy	Not a peer-reviewed journal article
Nguyen_2017	Feasibility and impacts of integrating nutrition interventions into an existing maternal, neonatal, and child health platform in Bangladesh	Not a peer-reviewed journal article
Nicklas_2016	Beta-testing a mobile health program designed to increase postpartum weight loss in women at elevated risk for cardiometabolic disease	Not a peer-reviewed journal article
Offiah_2020	A feasibility study on the prevention of post-partum stress urinary incontinence by midwife-delivered antenatal pelvic floor muscle training: a cluster randomised controlled trial	Not a peer-reviewed journal article
Parry_2017	Early vs Late wake therapy improves mood in antepartum vs Postpartum depression by differentially altering melatonin and sleep timing	Not a peer-reviewed journal article
Pintye_2019	Self-tests for at-home partner testing are acceptable & utilized among pregnant women	Not a peer-reviewed journal article
Porreco_2020	Fetal Movement Counting and Perinatal Mortality: A Systematic Review and Meta-analysis	Not a peer-reviewed journal article
Roche_2018	Analysis of comparable home/clinic blood pressure readings in the LEANBH (Learning to Evaluate and manage ANtenatal Blood pressure at Home) population	Not a peer-reviewed journal article
Rowe_2019	Online and mobile psychoeducation initiatives to prevent postnatal mental health problems: research and evaluation	Not a peer-reviewed journal article
Rubertsson_2020	A randomized controlled study during pregnacy comparing internetbased cognitive behavioral therapy and counseling by standard care for fear of birth	Not a peer-reviewed journal article
Ruggiero_1997	A stage-matched smoking cessation program for pregnant smokers	Not a peer-reviewed journal article
Sanavi_2014	Effective education to decrease elective caesarean section	Not a peer-reviewed journal article
Secher_2014	Real-time continuous glucose monitoring as a tool to prevent severe hypoglycaemia in selected pregnant women with Type 1 diabetes - an observational study	Not a peer-reviewed journal article

For peer review only - http://bmjopen.bmj.com/site/about/guidelines.xhtml

Author/Year	Title	Reasons for exclusion
Slade_2019	Preventing post-traumatic stress disorder: the Stress and Wellbeing after Childbirth Study(STRAWB2)	Not a peer-reviewed journal article
Smith_2014	Investigation of a lifestyle change strategy for high-risk women with a history of gestational diabetes	Not a peer-reviewed journal article
Smith_2014	The Blossom Project Online: Use of a behaviorally-based website to promote physical activity and prevent excessive gestational weight gain in previously sedentary pregnant women	Not a peer-reviewed journal article
Suhaimi_2016	A low-gi diet improves glucose self-monitoring in women with gestational diabetes mellitus	Not a peer-reviewed journal article
Tappin_2000	The effect of home-based motivational interviewing on the smoking behaviour of pregnant women: a pilot randomized controlled efficacy study	Not a peer-reviewed journal article
Tucker_2019	Self-monitoring of blood pressure during pregnancy: the BUMP trials	Not a peer-reviewed journal article
Varrassi_1989	Effects of physical activity on maternal plasma beta-endorphin levels and perception of labor pain	Not a peer-reviewed journal article
Wapner_1996	The CHUMS (Collaborative Home Uterine Monitoring Study) clinical trial - What does it really say?	Not a peer-reviewed journal article
Weeks_2013	Self-administration of misoprostol to prevent bleeding after homebirths in uganda: a pilot placebo-controlled, randomised trial	Not a peer-reviewed journal article
Wei_2013	Earlier management with continuous monitoring blood glucose decrease gestational weight gain in gestational diabetes mellitus: a randomised clinical trial	Not a peer-reviewed journal article
Wong_2015	A randomized controlled trial of an educational intervention to improve influenza vaccine uptake among pregnant women	Not a peer-reviewed journal article
Wroblewski_2004	Implementing a comprehensive postpartum depression support program	Not a peer-reviewed journal article
Wu_2018	The application of online pelvic floor training during the rehabilitation of postpartum pelvic floor	Not a peer-reviewed journal article
XiangHu_2020	Effects of exercise therapy for pregnancy-related low back pain and pelvic pain: A protocol for systematic review and meta-analysis	Not a peer-reviewed journal article
Yang_2018	Effectiveness of aerobic gymnastic exercise on stress, fatigue, and sleep quality during postpartum: a pilot randomized controlled trial	Not a peer-reviewed journal article
Yazdkhasti_2018	The effect of localized heat and cold therapy on pain intensity, duration of phases of labor, and birth outcomes among primiparous females: a randomized, controlled trial	Not a peer-reviewed journal article
Zlotnick_2001	Postpartum Depression in Women Receiving Public Assistance: Pilot Study of an Interpersonal-Therapy-Oriented Group Intervention	Not a peer-reviewed journal article
Ahn_2011	Effects of breast massage on breast pain, breast-milk sodium, and newborn suckling in early postpartum mothers	Not in English
Bakhteh_2018	Effect of cognitive behavioral therapy on self-efficacy in women with gestational diabetes: A randomized clinical trial	Not in English
Chae_2021	[Maternal Health Effects of Internet-Based Education Interventions during the Postpartum Period: A Systematic Review]	Not in English
deSouzaMelo_2020	Maternal and perinatal parameters after non-pharmacological interventions: a randomised, controlled clinical trial	Not in English
Gayeski_2010	Non-pharmacological approach to pain relief during labor as hard-light care technology: a systematic review	Not in English
Ghaderpanah_2017	The effect of 5A model on behavior change of physical activity in overweight pregnant women	Not in English
Ghazaie_2016	The effectiveness of cognitive-behavioral therapy on fear of childbirth, fear of pain, self-efficacy of childbirth and tendency to caesarean in nulliparous women	Not in English
Hanjani_2013	The effect of foot reflexology on pain intensity and duration of labor on primiparous	Not in English
Hung_2017	Non-pharmacological interventions for pregnancy-related sleep disturbances	Not in English
Imano_2013	Evaluation of program to improve mental and physical health	Not in English
Jeon_2018	Effects of a postnatal care program on self-efficacy, self-management, and glycemic control in women with gestational diabetes mellitus	Not in English
Khadivzadeh_2015	Effects of self-care education on perceived stress in women with gestational diabetes under insulin treatment	Not in English
Kharazi_2018	Effect of an educational intervention based on self-efficacy theory and health literacy strategies on pregnancy outcomes: A randomized clinical trial	Not in English
Khodaparast_2020	Examining the effect of educational intervention based on self-regulation model on perceived stress in women with gestational diabetes: A clinical trial study	Not in English
Khojasteh_2018	Comparison of in-person counseling and telephone support on delivery self-efficacy in primiparous women	Not in English
Kim_2014	Effects of an integrated self-management program on self-management, glycemic control, and maternal identity in women with gestational diabetes mellitus	Not in English
Kim_2021	Effects of nursing intervention programs for women with gestational diabetes: a systematic review of randomized controlled trials	Not in English
KimSue_2011	Effects of a Coaching-based Childbirth Program on Anxiety and Childbirth Self-efficacy among Primigravida Women	Not in English
Ko_2014	Effects of a coaching program on comprehensive lifestyle modification for women with gestational diabetes mellitus	Not in English

For peer review only - http://bmjopen.bmj.com/site/about/guidelines.xhtml

Author/Year	Title	Reasons for exclusion
Lawani_2003	Effect of antenatal gymnastics on childbirth: a study on 50 sedentary women in the Republic of Benin during the second and third quarters of pregnancy	Not in English
Lee_2017	Development and Validation of a Postpartum Care Mobile Application for First-time Mothers	Not in English
Lee_2018	Effects of family centered postpartum care for postpartum depression among previously infertile couples	Not in English
Liang_2019	Effectiveness of a Labor-Admission Education Program on Anxiety, Uncertainty, Locus of Labor Control, and Labor Outcomes in Pregnant Women	Not in English
Madady_2017	Effect of hot shower and intravenous injection of hyoscine on childbirth experience of nulliparous women: a randomized clinical trial	Not in English
Mafetoni_2014	NON-PHARMACOLOGICAL METHODS FOR PAIN RELIEF DURING LABOR: INTEGRATIVE REVIEW	Not in English
Makhouli_2018	Implementation of targeted education to promote normal vaginal delivery: a randomized controlled trial	Not in English
Manabe_2006	Implementation and evaluation of health training guidance for the purpose of improving self-care behavior among primiparae	Not in English
Mascarenhas_2019	Scientific evidence on non-pharmacological methods for relief of labor pain	Not in English
Mazloomy-Mahmoodabad_2020	The Effect of Nutrition Education on Gestational Weight Gain based on the Pender's Health Promotion Model: a randomized clinical trial study	Not in English
Mohamadkhani-Shahri_2017	Effect of massage aromatherapy with Lavender oil on pain intensity of active phase of labor in nulliparous women	Not in English
Mojahed_2018	The effect of motivational interviewing on self-efficacy to quit hookah smoking in pregnant women	Not in English
Moller_2003	Current aspects of the Thuringia prematurity preventional campaign 2000	Not in English
Niazi_2018	Comparison of the effects of purslane cream and Lanolin for treatment of breast fissure on lactation self-efficacy: A randomized clinical trial	Not in English
Ozgoli_2016	Comparison of peppermint and clove essential oil aroma on pain intensity and anxiety at first stage of labor	Not in English
Pour_2012	Comparison of effect of transcutaneous electrical nerve stimulation and acupressure in decreasing labor pain in primiparous women	Not in English
Rezaeian_2017	The relationship of prenatal Self-Care behaviors with stress, anxiety and depression in women at risk of preterm delivery	Not in English
Rostampour_2020	The effect of couples' supportive counseling on self-care behavior in women with insulin-treated gestational diabetes: A randomized clinical trial	Not in English
Safat_2018	Efficacy of cognitive behavioral therapy-based self-management intervention on labor outcome in nulliparous women	Not in English
Saling_2000	New method for the self assessment of the pH-value of the vaginal secretions by an indicator coated panty liner - First preliminary communication	Not in English
Saling_2001	A simple, efficient and inexpensive program for preventing prematurity	Not in English
Shim_2012	[Effects of a yoga-focused prenatal program on stress, anxiety, self confidence and labor pain in pregnant women with in vitro fertilization treatment]	Not in English
Shimada_2005	A randomized controlled trial on evaluating effectiveness of perineal massage during pregnancy in primiparous women	Not in English
Siegmund-Schultze_2005	pH self assessment to reduce the risk of preterm birth - A pilot study becomes a model project	Not in English
Wan_2016	Clinical observation of analgesia in delivery treated with acupoint pressure therapy	Not in English
Wang_2015	Effects of a Mobile Web-based Pregnancy Health Care Educational Program for Mothers at an Advanced Maternal Age	Not in English
Khunpradit_2011	Non-clinical interventions for reducing unnecessary caesarean section	Older versions of a Cochrane review
Lauzon_1998	Antenatal education for self?diagnosis of the onset of active labour at term	Older versions of a Cochrane review
Moy_2014	Techniques of monitoring blood glucose during pregnancy for women with pre-existing diabetes	Older versions of a Cochrane review
Moy_2017	Techniques of monitoring blood glucose during pregnancy for women with pre-existing diabetes	Older versions of a Cochrane review
Pintye_2018	Preexposure Prophylaxis for Human Immunodeficiency Virus (HIV) Prevention Among HIV-uninfected Pregnant Women: Estimated Coverage Using Risk-based Versus Regional Prevalence Approaches	Outcome - feasibility/acceptability only
Smith_2016	Programmes for advance distribution of misoprostol to prevent post-partum haemorrhage: A rapid literature review of factors affecting implementation	Outcome - feasibility/acceptability only
Steinberg_2021	Optimizing Engagement in an mHealth Intervention for Diabetes Support During Pregnancy: the Role of Baseline Patient Health and Behavioral Characteristics	Outcome - feasibility/acceptability only
Aba_2017	Antenatal Education on Pregnant Adolescents in Turkey: Prenatal Adaptation, Postpartum Adaptation, and Newborn Perceptions	Outcome - no health or behavioural outcomes
Aveyard_2006	A randomized controlled trial of smoking cessation for pregnant women to test the effect of a transtheoretical model-based intervention on movement in stage and interaction with baseline stage	Outcome - no health or behavioural outcomes
Bastani_2010	Impact of preconception health education on health locus of control and self-efficacy in women	Outcome - no health or behavioural outcomes
Carolan-Olah_2016	Educational and intervention programmes for gestational diabetes mellitus (GDM) management: An integrative review	Outcome - no health or behavioural outcomes
Chang_2004	Effect of Taegyo-focused prenatal education on maternal-fetal attachment and self-efficacy related to childbirth	Outcome - no health or behavioural outcomes

For peer review only - http://bmjopen.bmj.com/site/about/guidelines.xhtml

Author/Year	Title	Reasons for exclusion
Chen_2021	The accuracy and influencing factors for preference of self-sampling in group B streptococcus screening: a cross-sectional study	Outcome - no health or behavioural outcomes
Chillrud_2021	The effect of clean cooking interventions on mother and child personal exposure to air pollution: results from the Ghana Randomized Air Pollution and Health Study (GRAPHS)	Outcome - no health or behavioural outcomes
Clarke_2005	Alternative site self blood glucose testing is preferred by women with gestational diabetes	Outcome - no health or behavioural outcomes
Conde_2019	Effect of Electronic Video Education on Patient's Self-Assessed Knowledge About Obesity and Pelvic Floor Disorders: A Randomized Controlled Trial	Outcome - no health or behavioural outcomes
Damodaran_2015	Effect of Progressive Muscle Relaxation Technique in Terms oi Anxiety and Physiological Parameters of Antenatal Mothers with Pregnancy-Induced Hypertension	Outcome - no health or behavioural outcomes
Dean_2012	A pilot study using interactive SMS support groups to prevent mother-to-child HIV transmission in South Africa	Outcome - no health or behavioural outcomes
Dickerson_2010	Pregnancy and village outreach tibet: a descriptive report of a community- and home-based maternal-newborn outreach program in rural Tibet	Outcome - no health or behavioural outcomes
Elsinga_2008	The effect of preconception counselling on lifestyle and other behaviour before and during pregnancy	Outcome - no health or behavioural outcomes
Evans_2014	Initial outcomes from a 4-week follow-up study of the Text4baby program in the military women's population: randomized controlled trial	Outcome - no health or behavioural outcomes
Grym_2019	Feasibility of smart wristbands for continuous monitoring during pregnancy and one month after birth 11 Medical and Health Sciences 1117 Public Health and Health Services	Outcome - no health or behavioural outcomes
Haakstad_2018	How does a lifestyle intervention during pregnancy influence perceived barriers to leisure-time physical activity? The Norwegian fit for delivery study, a randomized controlled trial	Outcome - no health or behavioural outcomes
Harris_2015	A Pilot Study of Home-Based Smoking Cessation Programs for Rural, Appalachian, Pregnant Smokers	Outcome - no health or behavioural outcomes
Harrison_2020	A consumer co-created infographic improves short-term knowledge about physical activity and self-efficacy to exercise in women with gestational diabetes mellitus: a randomised trial	Outcome - no health or behavioural outcomes
Hasani_2021	The effect of counseling based on health promotion awareness on mental health and self-esteem in women with ectopic pregnancy: a randomized controlled clinical trial	Outcome - no health or behavioural outcomes
Hausenblas_2008	Development and evaluation of a multimedia CD-ROM for exercise during pregnancy and postpartum	Outcome - no health or behavioural outcomes
Hazra_2020	Effects of health behaviour change intervention through women's self-help groups on maternal and newborn health practices and related inequalities in rural india: A quasi-experimental study	Outcome - no health or behavioural outcomes
Howarth_2019	Low-cost, self-paced, educational programmes increase birth satisfaction in first-time mothers	Outcome - no health or behavioural outcomes
Jack_2015	Reducing Preconception Risks Among African American Women with Conversational Agent Technology	Outcome - no health or behavioural outcomes
Ke_2021	Perioperative mobile application for mothers undergoing Cesarean delivery: a prospective cohort study on patient engagement	Outcome - no health or behavioural outcomes
Korukcu_2017	The effect of the mindfulness-based transition to motherhood program in pregnant women with preterm premature rupture of membranes	Outcome - no health or behavioural outcomes
Koury_2019	A 3Dimensional Anatomical Education Model in Postpartum Perineal Laceration Care: A PrePost Intervention Study	Outcome - no health or behavioural outcomes
Kuonza_2010	Non-adherence to the single dose nevirapine regimen for the prevention of mother-to-child transmission of HIV in Bindura town, Zimbabwe: a cross-sectional analytic study	Outcome - no health or behavioural outcomes
Lawrence_2003	Design and evaluation of interventions promoting periconceptional multivitamin use	Outcome - no health or behavioural outcomes
Ledford_2016	Mobile application as a prenatal education and engagement tool: A randomized controlled pilot	Outcome - no health or behavioural outcomes
Lepper_2018	Effectiveness of a Prison-Based Healthy Pregnancy Curriculum Delivered to Pregnant Inmates	Outcome - no health or behavioural outcomes
Liu_2019	Effects of exercise on pregnant women's quality of life: A systematic review	Outcome - no health or behavioural outcomes
Logsdon_2020	Partnering with new mothers to develop a smart phone app to prevent maternal mortality after hospital discharge: A pilot study	Outcome - no health or behavioural outcomes
Mahomed_2000	Home-based mother's record: operational feasibility, understanding and usage in a rural community in Zimbabwe	Outcome - no health or behavioural outcomes
Medley_2018	Interventions during pregnancy to prevent preterm birth: an overview of Cochrane systematic reviews	Outcome - no health or behavioural outcomes
Menard_2012	Self-collected vaginal swabs for the quantitative real-time polymerase chain reaction assay of Atopobium vaginae and Gardnerella vaginalis and the diagnosis of bacterial vaginosis	Outcome - no health or behavioural outcomes
Mendelson_2018	A mindfulness intervention to reduce maternal distress in neonatal intensive care: a mixed methods pilot study	Outcome - no health or behavioural outcomes
Mollart_2016	A feasibility randomised controlled trial of acupressure to assist spontaneous labour for primigravid women experiencing a post-date pregnancy	Outcome - no health or behavioural outcomes
Mozumdar_2018	Increasing knowledge of home based maternal and newborn care using self-help groups: Evidence from rural Uttar Pradesh, India	Outcome - no health or behavioural outcomes

For peer review only - http://bmjopen.bmj.com/site/about/guidelines.xhtml

Author/Year	Title	Reasons for exclusion
Muhammad_2019	Pelvic Floor Muscle Exercise Education and Factors Associated with Implementation among Antenatal Women in Hospital Universiti Sains Malaysia	Outcome - no health or behavioural outcomes
Murphy_2005	Asthma self-management skills and the use of asthma education during pregnancy	Outcome - no health or behavioural outcomes
Nadeau_2021	Beta Testing of a Gestational Diabetes Risk Reduction Intervention for American Indian and Alaska Native TeensPre-eclampsia Educational Tool Impact on Knowledge, Anxiety, and Satisfaction in Pregnant Women: A Randomized Trial	Outcome - no health or behavioural outcomes
Ockhuijsen_2015	Exploring a self-help coping intervention for pregnant women with a miscarriage history	Outcome - no health or behavioural outcomes
Osterman_2011	Feasibility of Using Motivational Interviewing to Decrease Alcohol Consumption During Pregnancy	Outcome - no health or behavioural outcomes
Parsa_2019	Improving the knowledge of pregnant women using a pre-eclampsia app: A controlled before and after study	Outcome - no health or behavioural outcomes
Paterno_2020	Feasibility of a pilot, randomized controlled trial using a personalized health monitoring device with pregnant women for behavioral sleep research	Outcome - no health or behavioural outcomes
PetrovFieril_2016	Hemodynamic responses to single sessions of aerobic exercise and resistance exercise in pregnancy	Outcome - no health or behavioural outcomes
Prado_2016	Effects of maternal and child lipid-based nutrient supplements on infant development: a randomized trial in Malawi	Outcome - no health or behavioural outcomes
Reilly_2021	Attitudes and engagement of pregnant and postnatal women with a web-based emotional health tool (mummatters): Cross-sectional study	Outcome - no health or behavioural outcomes
Rigaud_2017	Assessing a Tool for Self-Monitoring Hydration Using Urine Color in Pregnant and Breastfeeding Women: A Cross-Sectional, Online Survey	Outcome - no health or behavioural outcomes
Robins_2019	Treatment Fidelity in Mind–Body Interventions	Outcome - no health or behavioural outcomes
Ross-McGill_2000	Antenatal home blood pressure monitoring: a pilot randomised controlled trial	Outcome - no health or behavioural outcomes
Sapiano_2012	Attitudes towards preconception care in Maltese women with type 1 diabetes mellitus	Outcome - no health or behavioural outcomes
Sercekus_2010	Effects of antenatal education on maternal prenatal and postpartum adaptation	Outcome - no health or behavioural outcomes
Shahnazi_2016	Effect of educational intervention on perceived susceptibility self-efficacy and DMFT of pregnantwomen	Outcome - no health or behavioural outcomes
Sharps_2013	Engaging and retaining abused women in perinatal home visitation programs	Outcome - no health or behavioural outcomes
Snyder_2019	Mental and physical support (MAPS) for moms: preliminary findings from a prenatal health support program	Outcome - no health or behavioural outcomes
Szymanski_2018	Uterine Artery Doppler Velocimetry During Individually Prescribed Exercise in Pregnancy	Outcome - no health or behavioural outcomes
Thackeray_2017	Effectiveness of message framing on women's intention to perform cytomegalovirus prevention behaviors: A cross-sectional study	Outcome - no health or behavioural outcomes
Troy_1995	Development of a self-care guide for postpartum fatigue	Outcome - no health or behavioural outcomes
Truong_2019	Community pharmacist counseling in early pregnancy—Results from the SafeStart feasibility study	Outcome - no health or behavioural outcomes
Ventura_2012	Cortisol and anxiety response to a relaxing intervention on pregnant women awaiting amniocentesis	Outcome - no health or behavioural outcomes
Weis_2012	Mentors offering maternal support: a support intervention for military mothers	Outcome - no health or behavioural outcomes
Weis_2017	Mentors Offering Maternal Support Reduces Prenatal, Pregnancy-Specific Anxiety in a Sample of Military Women	Outcome - no health or behavioural outcomes
Aveyard-2005	The influence of in-pregnancy smoking cessation programmes on partner quitting and women's social support mobilization: A randomized controlled trial	Outcome - socioeconomic/financial
Ammerman_2011	An Open Trial of In-Home CBT for Depressed Mothers in Home Visitation	Population - beyond 42 days postpartum
Armstrong_2004	The effectiveness of a pram-walking exercise programme in reducing depressive symptomatology for postnatal women	Population - beyond 42 days postpartum
Ayers_2015	Brief Online Self-help Exercises for Postnatal Women to Improve Mood: A Pilot Study	Population - beyond 42 days postpartum
Ayers_2018	Evaluation of expressive writing for postpartum health: a randomised controlled trial	Population - beyond 42 days postpartum
Bennion_2020	Impact of an Internet-Based Lifestyle Intervention on Behavioral and Psychosocial Factors During Postpartum Weight Loss	Population - beyond 42 days postpartum
Berry_2013	Rationale, design, and methodology for the optimizing outcomes in women with gestational diabetes mellitus and their infants study	Population - beyond 42 days postpartum
Bick_2019	Protocol for a two-arm feasibility RCT to support postnatal maternal weight management and positive lifestyle behaviour in women from an ethnically diverse inner city population: The SWAN feasibility trial	Population - beyond 42 days postpartum
Brandon_2012	Self-help booklets for preventing postpartum smoking relapse: a randomized trial	Population - beyond 42 days postpartum

For peer review only - http://bmjopen.bmj.com/site/about/guidelines.xhtml

Author/Year	Title	Reasons for exclusion
Branquinho_2020	A blended cognitive–behavioral intervention for the treatment of postpartum depression: study protocol for a randomized controlled trial	Population - beyond 42 days postpartum
Carson_2013	Evaluation of home testing to improve follow up after gestational diabetes (Fingerstick Assessments of sugar two-months postpartum or FAST)	Population - beyond 42 days postpartum
Chen_2000	Effects of support group intervention in postnatally distressed women A controlled study in Taiwan	Population - beyond 42 days postpartum
Cramp_2006	Moms in motion: a group-mediated cognitive-behavioral physical activity intervention	Population - beyond 42 days postpartum
Cramp_2009	Sustaining self-regulatory efficacy and psychological outcome expectations for postnatal exercise: effects of a group-mediated cognitive behavioural intervention	Population - beyond 42 days postpartum
Crawley_2018	Feasibility and acceptability of expressive writing with postpartum women: A randomised controlled trial	Population - beyond 42 days postpartum
Daley_2008	Feasibility of an exercise intervention for women with postnatal depression: a pilot randomised controlled trial	Population - beyond 42 days postpartum
Daley_2012	The effectiveness of exercise as a treatment for postnatal depression: study protocol	Population - beyond 42 days postpartum
Daley_2015	A pragmatic randomized controlled trial to evaluate the effectiveness of a facilitated exercise intervention as a treatment for postnatal depression: the PAM-PeRS trial	Population - beyond 42 days postpartum
Daley_2020	Feasibility and acceptability of a brief routine weight management intervention for postnatal women embedded within the national child immunisation programme in primary care: randomised controlled cluster feasibility trial	Population - beyond 42 days postpartum
Daley_2021	Practice nurse-supported weight self-management delivered within the national child immunisation programme for postnatal women: a feasibility cluster RCT	Population - beyond 42 days postpartum
Danaher_2012	Web-based intervention for postpartum depression: Formative research and design of the mommoodbooster program	Population - beyond 42 days postpartum
Danaher_2013	MomMoodBooster web-based intervention for postpartum depression: feasibility trial results	Population - beyond 42 days postpartum
Dennis_2003	The effect of peer support on postpartum depression: a pilot randomized controlled trial	Population - beyond 42 days postpartum
deRosset_2013	Mama Sana Usted Sana: Lessons learned from a postpartum weight loss intervention for Hispanic women with infants six months or less	Population - beyond 42 days postpartum
Diebold_2021	Testing the effects of a prenatal depression preventive intervention on parenting and young children's self-regulation and functioning (EPIC): protocol for a longitudinal observational study	Population - beyond 42 days postpartum
Dunning_2013	Wide Awake Parenting: Study protocol for a randomised controlled trial of a parenting program for the management of post-partum fatigue	Population - beyond 42 days postpartum
Fjeldsoe_2010	MobileMums: A<br>randomized controlled trial of an SMS-based physical activity intervention	Population - beyond 42 days postpartum
Fleming_1992	The effects of a social support group on depression, maternal attitudes and behavior in new mothers	Population - beyond 42 days postpartum
Fonseco_2019	Be a Mom, a WebBased Intervention to Prevent Postpartum Depression: The Enhancement of SelfRegulatory Skills and Its Association With Postpartum Depressive Symptoms	Population - beyond 42 days postpartum
Gammer_2020	A randomized controlled trial of an online, compassion-based intervention for maternal psychological well-being in the first year postpartum	Population - beyond 42 days postpartum
Giallo_2015	The efficacy of an intervention for the management of postpartum fatigue	Population - beyond 42 days postpartum
Golmakani_2015	The effect of pelvic floor muscle exercises program on sexual self-efficacy in primiparous women after delivery	Population - beyond 42 days postpartum
Gutke_2010	Specific muscle stabilizing as home exercises for persistent pelvic girdle pain after pregnancy: a randomized, controlled clinical trial	Population - beyond 42 days postpartum
Herring_2014	Using Technology to Promote Postpartum Weight Loss in Urban, Low-Income Mothers: A Pilot Randomized Controlled Trial	Population - beyond 42 days postpartum
Herring_2020	Feasibility of using a peer coach to deliver a behavioral intervention for promoting postpartum weight loss in Black and Latina mothers	Population - beyond 42 days postpartum
Kim_2021	Self-management mobile virtual reality program for women with gestational diabetes	Population - beyond 42 days postpartum
Koniak-Griffin_2003	Nurse visitation for adolescent mothers: two-year infant health and maternal outcomes	Population - beyond 42 days postpartum
Krummel_2010	Lessons Learned from the Mothers' Overweight Management Study in 4 West Virginia WIC Offices	Population - beyond 42 days postpartum
LeCheminant_2014	Effect of resistance training on body composition, self-efficacy, depression, and activity in postpartum women	Population - beyond 42 days postpartum
Mailey_2019	Is a general or specific exercise recommendation more effective for promoting physical activity among postpartum mothers?	Population - beyond 42 days postpartum
Mitchell_2018	Feasibility and acceptability of a brief online self-compassion intervention for mothers of infants	Population - beyond 42 days postpartum
Monteiro_2021	Be a Mom, a WebBased Intervention to Promote Positive Mental Health Among Postpartum Women With Low Risk for Postpartum Depression: Exploring Psychological Mechanisms of Change	Population - beyond 42 days postpartum
Morrell_2009	Clinical effectiveness of health visitor training in psychologically informed approaches for depression in postnatal women: pragmatic cluster randomised trial in primary care	Population - beyond 42 days postpartum

For peer review only - http://bmjopen.bmj.com/site/about/guidelines.xhtml

Author/Year	Title	Reasons for exclusion
Muijsers_2020	Blood pressure after PREeclampsia/HELLP by SELF monitoring (BP-PRESELF): Rationale and design of a multicenter randomized controlled trial	Population - beyond 42 days postpartum
Murphy_2017	Pilot trial of a parenting and self-care intervention for HIV-positive mothers: the IMAGE program	Population - beyond 42 days postpartum
Peacock_2015	A randomised controlled trial to delay or prevent type 2 diabetes after gestational diabetes: walking for exercise and nutrition to prevent diabetes for you	Population - beyond 42 days postpartum
Perez-Blasco_2013	Effects of a mindfulness-based intervention on psychological distress, well-being, and maternal self-efficacy in breast-feeding mothers: results of a pilot study	Population - beyond 42 days postpartum
Phelan_2017	Effect of an Internet-Based Program on Weight Loss for Low-Income Postpartum Women: a Randomized Clinical Trial	Population - beyond 42 days postpartum
Power_2019	Engagement and Weight Loss in a Web and Mobile Program for Low-Income Postpartum Women: fit Moms/Mamás Activas	Population - beyond 42 days postpartum
Rich-Edwards_2019	Randomized Trial to Reduce Cardiovascular Risk in Women with Recent Preeclampsia	Population - beyond 42 days postpartum
Rollo_2020	The feasibility and preliminary efficacy of an ehealth lifestyle program in women with recent gestational diabetes mellitus: a pilot study	Population - beyond 42 days postpartum
Sampson_2020	Reducing depression among mothers in a residential treatment setting: building evidence for the PST4PPD intervention	Population - beyond 42 days postpartum
Swanson_2009	Resolution of depression and grief during the first year after miscarriage: a randomized controlled clinical trial of couples-focused interventions	Population - beyond 42 days postpartum
Teychenne_2021	Mums on the Move: A pilot randomised controlled trial of a home-based physical activity intervention for mothers at risk of postnatal depression	Population - beyond 42 days postpartum
Timlin_2017	A preliminary randomised control trial of the effects of Dru yoga on psychological well-being in Northern Irish first time mothers	Population - beyond 42 days postpartum
Tripette_2014	Home-Based Active Video Games to Promote Weight Loss during the Postpartum Period	Population - beyond 42 days postpartum
Walker_2012	Ethnic-Specific Weight-Loss Interventions for Low-Income Postpartum Women: Findings and Lessons	Population - beyond 42 days postpartum
Watson_2005	The feasibility and effectiveness of pram walking groups for postpartum women in western Sydney	Population - beyond 42 days postpartum
Wilkinson_2015	Trial for Reducing Weight Retention in New Mums: a randomised controlled trial evaluating a low intensity, postpartum weight management programme	Population - beyond 42 days postpartum
Abbasi_2017	Comparing the effect of e-learning and educational booklet on the childbirth self-efficacy: a randomized controlled clinical trial	Population - not targeting pregnant women
Aleman_2017	Brief counseling on secondhand smoke exposure in pregnant women in Argentina and uruguay	Population - not targeting pregnant women
Althabe_2016	A Multifaceted Strategy to Implement Brief Smoking Cessation Counseling During Antenatal Care in Argentina and Uruguay: A Cluster Randomized Trial	Population - not targeting pregnant women
Baradaran-Akbarzadeh_2018	The effect of educational package on sexual function in cold temperament women of reproductive age	Population - not targeting pregnant women
Briggs_2016	A systematic review of interventions to improve knowledge and self-management skills concerning contraception, pregnancy and breastfeeding in people with rheumatoid arthritis	Population - not targeting pregnant women
Chomat_2019	Women's circles as a culturally safe psychosocial intervention in Guatemalan indigenous communities: a community-led pilot randomised trial	Population - not targeting pregnant women
DeNicola_2020	Telehealth Interventions to Improve Obstetric and Gynecologic Health Outcomes: A Systematic Review	Population - not targeting pregnant women
Fallin-Bennett_2018	Pilot Tobacco Treatment Intervention for Women in Residential Treatment for Substance Use Disorder	Population - not targeting pregnant women
Fisher_2018	Addressing multiple modifiable risks through structured community-based Learning Clubs to improve maternal and infant health and infant development in rural Vietnam: protocol for a parallel group cluster randomised controlled trial	Population - not targeting pregnant women
Ghetti_2019	Longitudinal Study of music Therapy's Effectiveness for Premature infants and their caregivers (LongSTEP): protocol for an international randomised trial	Population - not targeting pregnant women
Harvey_2008	Hygiene interventions for prevention of cytomegalovirus infection among childbearing women: Systematic review	Population - not targeting pregnant women
Hillemeier_2008	Improving women's preconceptional health: findings from a randomized trial of the Strong Healthy Women intervention in the Central Pennsylvania women's health study	Population - not targeting pregnant women
Johnson_2018	Protocol for the ROSE sustainment (ROSES) study, a sequential multiple assignment randomized trial to determine the minimum necessary intervention to maintain a postpartum depression prevention program in prenatal clinics serving low-income women	Population - not targeting pregnant women
Karimiankakolaki_2019	Designing an educational intervention on second-hand smoke in smoker men on the exposure of pregnant wives: a protocol for a randomized controlled trial	Population - not targeting pregnant women
Karyotaki_2021	The effects of psychological treatment of perinatal depression: an overview	Population - not targeting pregnant women
Mackintosh_2020	Interventions to increase patient and family involvement in escalation of care for acute life-threatening illness in community health and hospital settings	Population - not targeting pregnant women
Makaruk_2019	The Effectiveness of Regular Exercise Programs in the Prevention of Gestational Diabetes Mellitus-A Systematic Review	Population - not targeting pregnant women

Author/Year	Title	Reasons for exclusion
Matkin_2019	Telephone counselling for smoking cessation	Population - not targeting pregnant women
McRobbie_2016	Tackling obesity in areas of high social deprivation: clinical effectiveness and cost-effectiveness of a task-based weight management group programme - A randomised controlled trial and economic evaluation	Population - not targeting pregnant women
Mutale_2021	Addition of HIV self-test kits to partner notification services to increase HIV testing of male partners of pregnant women in Zambia: two parallel randomised trials	Population - not targeting pregnant women
Nayak_2019	Randomized Trial of an Innovative Electronic Screening and Brief Intervention for Reducing Drinking among Women of Childbearing Age	Population - not targeting pregnant women
Nguyen_2018	Engagement of Husbands in a Maternal Nutrition Program Substantially Contributed to Greater Intake of Micronutrient Supplements and Dietary Diversity during Pregnancy: Results of a Cluster-Randomized Program Evaluation in Bangladesh	Population - not targeting pregnant women
O'Dea_2015	Can the Onset of Type 2 Diabetes Be Delayed by a Group-Based Lifestyle Intervention in Women with Prediabetes following Gestational Diabetes Mellitus (GDM)? Findings from a Randomized Control Mixed Methods Trial	Population - not targeting pregnant women
Okeibunor_2011	Preventing malaria in pregnancy through community-directed interventions: Evidence from Akwa Ibom State, Nigeria	Population - not targeting pregnant women
Ovretveit_2021	Innovations in self care and close care made during COVID 19 pandemic: a narrative review	Population - not targeting pregnant women
Pettifor_2020	HIV self-testing among young women in rural South Africa: A randomized controlled trial comparing clinic-based HIV testing to the choice of either clinic testing or HIV self-testing with secondary distribution to peers and partners	Population - not targeting pregnant women
Pinchoff_2019	Evidence-Based Process for Prioritizing Positive Behaviors for Promotion: Zika Prevention in Latin America and the Caribbean and Applicability to Future Health Emergency Responses	Population - not targeting pregnant women
Pintye_2019	Acceptability and outcomes of distributing HIV selftests for male partner testing in Kenyan maternal and child health and family planning clinics	Population - not targeting pregnant women
Shen_2021	Clinical Evaluation of a Self-Testing Kit for Vaginal Infection Diagnosis	Population - not targeting pregnant women
Taylor_2019	Be Healthe for Your Heart: protocol for a Pilot Randomized Controlled Trial Evaluating a Web-Based Behavioral Intervention to Improve the Cardiovascular Health of Women With a History of Preeclampsia	Population - not targeting pregnant women
Tieu_2017	Preconception care for diabetic women for improving maternal and infant health	Population - not targeting pregnant women
van-der-Zwan_2019	The Effect of Heart Rate Variability Biofeedback Training on Mental Health of Pregnant and Non-Pregnant Women: a Randomized Controlled Trial	Population - not targeting pregnant women
Vézina-Im_2016	Implementation intentions intervention to promote fruit and vegetable intake in childbearing age women at risk for gestational diabetes mellitus: 6-month follow-up of a randomized controlled trial	Population - not targeting pregnant women
Youngkin_2010	Promoting self-care and secondary prevention in women's health: A study to test the accuracy of a home self-test system for bacterial vaginosis	Population - not targeting pregnant women
Adolfsson_2014	A web-based support for pregnant women and new mothers with type 1 diabetes mellitus in Sweden (MODIAB-Web): Study protocol for a randomized controlled trial	Protocol - results already included
Bais_2016	Bright light therapy in pregnant women with major depressive disorder: study protocol for a randomized, double-blind, controlled clinical trial	Protocol - results already included
Band_2019	Intervention planning and modification of the BUMP intervention: A digital intervention for the early detection of raised blood pressure in pregnancy	Protocol - results already included
Clifton_2016	Design of lifestyle intervention trials to prevent excessive gestational weight gain in women with overweight or obesity	Protocol - results already included
Cooper_2015	Pilot study to evaluate a tailored text message intervention for pregnant smokers (MiQuit): Study protocol for a randomised controlled trial	Protocol - results already included
Dodd_2011	Limiting weight gain in overweight and obese women during pregnancy to improve health outcomes: the LIMIT randomised controlled trial	Protocol - results already included
Dol_2019	Impact of mobile health interventions during the perinatal period for mothers in low- and middle-income countries: a systematic review protocol	Protocol - results already included
Evans_2019	Design of a novel digital intervention to promote healthy weight management among postpartum African American women	Protocol - results already included
Garmendia_2018	Effectiveness on maternal and offspring metabolic control of a home-based dietary counseling intervention and DHA supplementation in obese/overweight pregnant women (MIGHT study): A randomized controlled trial-Study protocol	Protocol - results already included
Griffiths_2016	Are digital interventions for smoking cessation in pregnancy effective? A systematic review protocol	Protocol - results already included
Grzeskowiak_2014	A randomized controlled trial to assess the clinical and cost effectiveness of a nurse-led Antenatal Asthma Management Service in South Australia (AAMS study)	Protocol - results already included
Jones_2012	Pain management for women in labour: an overview of systematic reviews	Protocol - results already included
Kinser_2020	Protocol for Pilot Study on Self-Management of Depressive Symptoms in Pregnancy	Protocol - results already included
Lim_2019	Smart Phone APP to Restore Optimal Weight (SPAROW): protocol for a randomised controlled trial for women with recent gestational diabetes	Protocol - results already included
Loukopoulou_2011	Design and study protocol of the maternal smoking cessation during pregnancy study, (M-SCOPE)	Protocol - results already included

For peer review only - http://bmjopen.bmj.com/site/about/guidelines.xhtml

Author/Year	Title	Reasons for exclusion
Lucas_2019	Promoting self-management of breast and nipple pain in breastfeeding women: Protocol of a pilot randomized controlled trial	Protocol - results already included
Miquelutti_2015	Developing strategies to be added to the protocol for antenatal care: an exercise and birth preparation program	Protocol - results already included
Navas_2018	Effectiveness and safety of moderate-intensity aerobic water exercise during pregnancy for reducing use of epidural analgesia during labor: protocol for a randomized clinical trial	Protocol - results already included
Sapkota_2019	Counselling-based psychosocial intervention to improve the mental health of abused pregnant women: a protocol for randomised controlled feasibility trial in a tertiary hospital in eastern Nepal	Protocol - results already included
Ternstrom_2017	A randomized controlled study comparing internet-based cognitive behavioral therapy and counselling by standard care for fear of birth - A study protocol	Protocol - results already included
Trevillion_2015	Depression: an exploratory parallel-group randomised controlled trial of antenatal guided self help for women (dawn trial)	Protocol - results already included
Whitemore_2019	Effectiveness and cost-effectiveness of a tailored text-message programme (MiQuit) for smoking cessation in pregnancy: Study protocol for a randomised controlled trial (RCT) and meta-analysis	Protocol - results already included
Willcox_2015	Testing the feasibility of a mobile technology intervention promoting healthy gestational weight gain in pregnant women (txt4two) - study protocol for a randomised controlled trial	Protocol - results already included
Zairina_2015	Study protocol for a randomised controlled trial evaluating the efficacy of a telehealth program - management of asthma with supportive telehealth of respiratory function in pregnancy (MASTERY©)	Protocol - results already included
Aslani_2014	Impact of Computer-Based Pregnancy-Induced Hypertension and Diabetes Decision Aids on Empowering Pregnant Women	Protocol - results not published
Baird_2016	Southampton PRegnancy Intervention for the Next Generation (SPRING): protocol for a randomised controlled trial	Protocol - results not published
Bei_2019	A scalable cognitive behavioural program to promote healthy sleep during pregnancy and postpartum periods: Protocol of a randomised controlled trial (the SEED project)	Protocol - results not published
Browne_2015	Uterine Tonus Assessment by Midwives versus Patient self-assessment in the active management of the third stage of labor (UTAMP): Study protocol for a randomized controlled trial	Protocol - results not published
Caramlau_2011	Mums 4 Mums: structured telephone peer-support for women experiencing postnatal depression Pilot and exploratory RCT of its clinical and cost effectiveness	Protocol - results not published
Carolan-Olah_2015	Development and initial testing of a GDM information website for multi-ethnic women with GDM	Protocol - results not published
Chang_2015	Electronic personal maternity records: Both web and smartphone services	Protocol - results not published
Dallosso_2018	Baby Steps - A structured group education programme with accompanying mobile web application designed to promote physical activity in women with a history of gestational diabetes: Study protocol for a randomised controlled trial	Protocol - results not published
Davis_2018	Optimizing Gestational Weight Gain With the Eating4Two Smartphone App: Protocol for a Randomized Controlled Trial	Protocol - results not published
Edwards_2019	Can an educational web-intervention, co-created by service users alongside self-efficacy theory, affect nulliparous women's experiences of early labour? A study protocol for a randomised control trial (the L-TEL Trial)	Protocol - results not published
Hawkins_2019	Feasibility of a Sleep Self-Management Intervention in Pregnancy Using a Personalized Health Monitoring Device: Protocol for a Pilot Randomized Controlled Trial	Protocol - results not published
Koushede_2013	Group-based antenatal birth and parent preparation for improving birth outcomes and parenting resources: study protocol for a randomised trial	Protocol - results not published
LeBlanc_2016	Prepare, a randomized trial to promote and evaluate weight loss among overweight and obese women planning pregnancy: Study design and rationale	Protocol - results not published
Lenz_2018	Mindfulness-based Stress Reduction in Pregnancy: an App-Based Programme to Improve the Health of Mothers and Children (MINDFUL/PMI Study)	Protocol - results not published
Martin_2018	A pilot randomized controlled trial of a remotely-delivered behavioral health coaching program to limit weight gain in pregnancy and reduce postpar-tum weight retention	Protocol - results not published
Monteiro_2019	Non-pharmacological interventions for treating sexual dysfunction in postpartum women: A systematic review protocol	Protocol - results not published
Nagle_2013	Primary prevention of gestational diabetes for women who are overweight and obese: a randomised controlled trial	Protocol - results not published
Nwolise_2017	Exploring the acceptability and feasibility of a preconception and diabetes information app for women with pregestational diabetes: A mixed-methods study protocol	Protocol - results not published
Pallitto_2016	Testing a counselling intervention in antenatal care for women experiencing partner violence: a study protocol for a randomized controlled trial in Johannesburg, South Africa	Protocol - results not published
Pascual-Morena_2019	Exercise vs metformin for gestational diabetes mellitus: Protocol for a network meta-analysis	Protocol - results not published
Patten_2019	The Healthy Pregnancies Project: Study protocol and baseline characteristics for a cluster-randomized controlled trial of a community intervention to reduce tobacco use among Alaska Native pregnant women	Protocol - results not published
Pelaez-Crisologo_2009	Different techniques of blood glucose monitoring in women with gestational diabetes for improving maternal and infant health	Protocol - results not published
Prabhu_2020	The effect of a prenatal psychosocial education program on postnatal depression, stress, and parenting self-efficacy in women in South India - A study protocol for a randomised controlled trial	Protocol - results not published

For peer review only

For peer review only - http://bmjopen.bmj.com/site/about/guidelines.xhtml

Author/Year	Title	Reasons for exclusion
Qian_2021	Effectiveness of non-pharmacological interventions for reducing postpartum fatigue: a systematic review protocol	Protocol - results not published
Reynolds_2016	MAHILA: a protocol for evaluating a nurse-delivered mHealth intervention for women with HIV and psychosocial risk factors in India	Protocol - results not published
Roberts_2004	Protocol for a randomised controlled trial of a decision aid for the management of pain in labour and childbirth	Protocol - results not published
Sacristan-Martin_2019	A mindfulness and compassion-based program applied to pregnant women and their partners to decrease depression symptoms during pregnancy and postpartum: study protocol for a randomized controlled trial	Protocol - results not published
Seo_2021	The Development of the Postpartum Depression Self-Management Mobile Application "happy Mother"	Protocol - results not published
Singh_2017	Internet-delivered blood glucose awareness training (BGAT) for women with type 1 diabetes (T1D) contemplating pregnancy: results from the Bump2be trial	Protocol - results not published
Tielsch_2015	Designs of two randomized, community-based trials to assess the impact of alternative cookstove installation on respiratory illness among young children and reproductive outcomes in rural Nepal	Protocol - results not published
Tomfohr-Madsen_2016	Mindfulness-based cognitive therapy for psychological distress in pregnancy: study protocol for a randomized controlled trial	Protocol - results not published
Tomlinson_2011	An effectiveness study of an integrated, community-based package for maternal, newborn, child and HIV care in South Africa: study protocol for a randomized controlled trial	Protocol - results not published
Veringa_2016	I've Changed My Mind', Mindfulness-Based Childbirth and Parenting (MBCP) for pregnant women with a high level of fear of childbirth and their partners: study protocol of the quasi-experimental controlled trial	Protocol - results not published
Volrathongchai_2013	Non-pharmacological interventions for women with postpartum fatigue	Protocol - results not published
Wilcox_2018	A randomized controlled trial to prevent excessive gestational weight gain and promote postpartum weight loss in overweight and obese women: Health In Pregnancy and Postpartum (HIPP)	Protocol - results not published
Yeung_2019	Birth ball for pregnant women in labour research protocol: A multi-centre randomised controlled trial	Protocol - results not published

For peer review only

Supplementary File S7. Reference list of included studies

1. Aaronson NK, Ershoff DH, Danaher BG: Smoking cessation in pregnancy: a self-help approach. *Addict Behav* 1985, 10(1):103-108.
2. Abdel-Aleem H, Singata M, Abdel-Aleem M, Mshweshwe N, Williams X, Hofmeyr GJ: Uterine massage to reduce postpartum hemorrhage after vaginal delivery. *Int J Gynaecol Obstet* 2010, 111(1):32-36.
3. Abedi P, Jahanfar S, Namvar F, Lee J: Breastfeeding or nipple stimulation for reducing postpartum haemorrhage in the third stage of labour. In., vol. 2016; 2016.
4. Adams J, Frawley J, Steel A, Broom A, Sibbritt D: Use of pharmacological and non-pharmacological labour pain management techniques and their relationship to maternal and infant birth outcomes: examination of a nationally representative sample of 1835 pregnant women. *Midwifery* 2015, 31(4):458-463.
5. Adler SP, Finney JW, Manganello AM, Best AM: Prevention of child-to-mother transmission of cytomegalovirus among pregnant women. *J Pediatr* 2004, 145(4):485-491.
6. Afravi S, Abbaspoor Z, Montazeri S, Cheraghian B: The effect of Hugo point pressure on postpartum pain in multiparous women. *Family Medicine & Primary Care Review* 2019, 21(1):7-11.
7. Ahmadpour P, Mosavi S, Mohammad-Alizadeh-Charandabi S, Jahanfar S, Mirghafourvand M: Evaluation of the birth plan implementation: a parallel convergent mixed study. *Reprod Health* 2020, 17(1):138.
8. Ajibaye O, Balogun EO, Olukosi YA, Orok BA, Oyebola KM, Iwalokun BA, Aina OO, Shittu O, Adeneye AK, Ojewunmi OO *et al*: Impact of training of mothers, drug shop attendants and voluntary health workers on effective diagnosis and treatment of malaria in Lagos, Nigeria. *Trop Parasitol* 2019, 9(1):36-44.
9. Ajuzieogu OV, Amucheazi A, Ezike HA, Achi J, Abam DS: The efficacy of chewing gum on postoperative ileus following cesarean section in Enugu, South East Nigeria: A randomized controlled clinical trial. *Niger J Clin Pract* 2014, 17(6):739-742.
10. Akselsson A, Lindgren H, Georgsson S, Pettersson K, Steineck G, Skokic V, Radestad I: Mindfetalness to increase women's awareness of fetal movements and pregnancy outcomes: a cluster-randomised controlled trial including 39 865 women. *BJOG* 2020, 127(7):829-837.
11. Al Hashmi I, Nandy K, Seshan V: Non-medical strategies to improve pregnancy outcomes of women with gestational diabetes mellitus: a literature review. In., vol. 19; 2019.
12. Al Khamis S, Asimakopoulou K, Newton T, Daly B: The effect of dental health education on pregnant women's adherence with toothbrushing and flossing - A randomized control trial. *Community Dent Oral Epidemiol* 2017, 45(5):469-477.
13. Al-Hashmi I, Hodge F, Nandy K, Thomas E, Brecht ML: The Effect of a Self-Efficacy-Enhancing Intervention on Perceived Self-Efficacy and Actual Adherence to Healthy Behaviours Among Women with Gestational Diabetes Mellitus. *Sultan Qaboos Univ Med J* 2018, 18(4):e513-e519.
14. Alekseev NP, Vladimir, II, Nadezhda TE: Pathological postpartum breast engorgement: prediction, prevention, and resolution. *Breastfeed Med* 2015, 10(4):203-208.
15. Alexander DA, Northcross A, Karrison T, Morhasson-Bello O, Wilson N, Atalabi OM, Dutta A, Adu D, Ibigbami T, Olamijulo J *et al*: Pregnancy outcomes and ethanol cook stove intervention: A randomized-controlled trial in Ibadan, Nigeria. *Environ Int* 2018, 111:152-163.
16. Alfadhli E, Osman E, Basri T: Use of a real time continuous glucose monitoring system as an educational tool for patients with gestational diabetes. *Diabetol Metab Syndr* 2016, 8(1):48.
17. Alizadeh S, Riazi H, Majd HA, Ozgoli G: The effect of sexual health education on sexual activity, sexual quality of life, and sexual violence in pregnancy: a prospective randomized controlled trial. *BMC Pregnancy Childbirth* 2021, 21(1):334.
18. Alnuaimi K, Abuidhail J, Abuzaid H: The effects of an educational programme about preeclampsia on women's awareness: a randomised control trial. *Int Nurs Rev* 2020, 67(4):501-511.

For peer review only - http://bmjopen.bmj.com/site/about/guidelines.xhtml

19. Altazan AD, Redman LM, Burton JH, Beyl RA, Cain LE, Sutton EF, Martin CK: Mood and quality of life changes in pregnancy and postpartum and the effect of a behavioral intervention targeting excess gestational weight gain in women with overweight and obesity: a parallel-arm randomized controlled pilot trial. *BMC Pregnancy Childbirth* 2019, 19(1):50.

20. Ambardekar S, Shochet T, Bracken H, Coyaji K, Winikoff B: Calibrated delivery drape versus indirect gravimetric technique for the measurement of blood loss after delivery: a randomized trial. *BMC Pregnancy Childbirth* 2014, 14(1):276.

21. Anderson L, Kynoch K, Kildea S, Lee N: Effectiveness of breast massage for the treatment of women with breastfeeding problems: a systematic review. *JBI Database System Rev Implement Rep* 2019, 17(8):1668-1694.

22. Anderson NH, Gordon A, Li M, Cronin RS, Thompson JMD, Raynes-Greenow CH, Heazell AEP, Stacey T, Culling VM, Wilson J *et al*: Association of Supine Going-to-Sleep Position in Late Pregnancy With Reduced Birth Weight: A Secondary Analysis of an Individual Participant Data Meta-analysis. *JAMA Netw Open* 2019, 2(10):e1912614.

23. Antoszewski B, Sobczak M, Kasielska-Trojan A: Self-assessment of striae gravidarum prophylaxis. *Postepy Dermatol Alergol* 2015, 32(6):459-464.

24. Aprilina HD, Krislinggardini K, Isnaini N, Suratmi S: The Effect of Cabbage Leaves Compress on Breast Engorgement in Postpartum Mother. *Open Access Macedonian Journal of Medical Sciences* 2021, 9(T4):124-128.

25. Araban M, Baharzadeh K, Karimy M: Nutrition modification aimed at enhancing dietary iron and folic acid intake: an application of health belief model in practice. *Eur J Public Health* 2017, 27(2):287-292.

26. Araban M, Tavafian SS, Zarandi SM, Hidarnia AR, Burri A, Montazeri A: A behavioral strategy to minimize air pollution exposure in pregnant women: a randomized controlled trial. *Environ Health Prev Med* 2017, 22(1):26.

27. Ardilouze A, Bouchard P, Hivert MF, Simard C, Allard C, Garant MP, Menard J, Ouellet A, Houde G, Pesant MH *et al*: Self-Monitoring of Blood Glucose: A Complementary Method Beyond the Oral Glucose Tolerance Test to Identify Hyperglycemia During Pregnancy. *Can J Diabetes* 2019, 43(8):627-635.

28. Arimori N: Randomized controlled trial of decision aids for women considering prenatal testing: The effect of the Ottawa Personal Decision Guide on decisional conflict. *Japan Journal of Nursing Science* 2006, 3(2):119-130.

29. Arthur C, Di Corleto E, Ballard E, Kothari A: A randomized controlled trial of daily weighing in pregnancy to control gestational weight gain. *BMC Pregnancy Childbirth* 2020, 20(1):223.

30. Asci O, Rathfisch G: Effect of lifestyle interventions of pregnant women on their dietary habits, lifestyle behaviors, and weight gain: a randomized controlled trial. *J Health Popul Nutr* 2016, 35:7.

31. Ashorn P, Alho L, Ashorn U, Cheung YB, Dewey KG, Harjunmaa U, Lartey A, Nkhoma M, Phiri N, Phuka J *et al*: The impact of lipid-based nutrient supplement provision to pregnant women on newborn size in rural Malawi: a randomized controlled trial. *Am J Clin Nutr* 2015, 101(2):387-397.

32. Ashworth DC, Maule SP, Stewart F, Nathan HL, Shennan AH, Chappell LC: Setting and techniques for monitoring blood pressure during pregnancy. In., vol. 2020; 2020.

33. Avery MD, Leon AS, Kopher RA: Effects of a partially home-based exercise program for women with gestational diabetes. *Obstet Gynecol* 1997, 89(1):10-15.

34. Avsar TS, McLeod H, Jackson L: Health outcomes of smoking during pregnancy and the postpartum period: an umbrella review. *BMC Pregnancy Childbirth* 2021, 21(1):254.

35. Bagharpoosh M, Sangestani G, Goodarzi M: Effect of progressive muscle relaxation technique on pain relief during labor. *Acta Medica Iranica* 2006, 44(3).

36. Bais B, Kamperman AM, Bijma HH, Hoogendijk WJ, Souman JL, Knijff E, Lambregtse-van den Berg MP: Effects of bright light therapy for depression during pregnancy: a randomised, double-blind controlled trial. *BMJ Open* 2020, 10(10):e038030.

For peer review only - http://bmjopen.bmj.com/site/about/guidelines.xhtml

37. Balami AD, Said SM, Zulkefli NAM, Norsa'adah B, Audu B: Improving malaria preventive practices and pregnancy outcomes through a health education intervention: A randomized controlled trial. *Malar J* 2021, 20(1):55.

38. Bao H, Yu P, Song X, Zhou Y, Zhu Y, Xu X: The influence of home-based exercise on gestational diabetes: a meta-analysis of randomized controlled trials. *J Matern Fetal Neonatal Med* 2020, 33(13):2300-2305.

39. Barakat R, Vargas M, Brik M, Fernandez I, Gil J, Coteron J, Santacruz B: Does Exercise During Pregnancy Affect Placental Weight?: A Randomized Clinical Trial. *Eval Health Prof* 2018, 41(3):400-414.

40. Barber V, Calvert A, Vandrevala T, Star C, Khalil A, Griffiths P, Heath PT, Jones CE: Prevention of Acquisition of Cytomegalovirus Infection in Pregnancy through Hygiene-based Behavioral Interventions: A Systematic Review and Gap Analysis. In.; 2020.

41. Barroso CS, Yockey A, Degon E, Gautam Poudel P, Brown SD, Hedderson MM, Moreno-Hunt C, Ehrlich SF: Efficacious lifestyle interventions for appropriate gestational weight gain in women with overweight or obesity set in the health care system: a scoping review. In.; 2021.

42. Bartu A, Sharp J, Ludlow J, Doherty DA: Postnatal home visiting for illicit drug-using mothers and their infants: a randomised controlled trial. *Aust N Z J Obstet Gynaecol* 2006, 46(5):419-426.

43. Baruth M, Schlaff RA, Deere S, Walker JL, Dressler BL, Wagner SF, Boggs A, Simon HA: The Feasibility and Efficacy of a Behavioral Intervention to Promote Appropriate Gestational Weight Gain. *Matern Child Health J* 2019, 23(12):1604-1612.

44. Bastani F, Hidarnia A, Kazemnejad A, Vafaei M, Kashanian M: A randomized controlled trial of the effects of applied relaxation training on reducing anxiety and perceived stress in pregnant women. *J Midwifery Womens Health* 2005, 50(4):e36-40.

45. Bastani FF, Sharami SH, Aski SK, Milani F, Khakifirooz B, Shakiba M, Attari SM, Omidkhah T: The Effect of Simultaneous Application of Transcutaneous Electrical Nerve Stimulation (TENS) on Specific Lumbar and Acupuncture Points on Labour Pain Relief: A Randomised Clinical Trial. *Journal of Clinical and Diagnostic Research* 2020.

46. Bastiaenen CH, de Bie RA, Vlaeyen JW, Goossens ME, Leffers P, Wolters PM, Bastiaanssen JM, Brandt PA, Essed GG: Long-term effectiveness and costs of a brief self-management intervention in women with pregnancy-related low back pain after delivery. *BMC Pregnancy Childbirth* 2008, 8:19.

47. Batra P, Mangione CM, Cheng E, Steers WN, Nguyen TA, Bell D, Kuo AA, Gregory KD: A Cluster Randomized Controlled Trial of the MyFamilyPlan Online Preconception Health Education Tool. *Am J Health Promot* 2018, 32(4):897-905.

48. Baumel A, Tinkelman A, Mathur N, Kane JM: Digital Peer-Support Platform (7Cups) as an Adjunct Treatment for Women With Postpartum Depression: Feasibility, Acceptability, and Preliminary Efficacy Study. *JMIR Mhealth Uhealth* 2018, 6(2):e38.

49. Beckmann MM, Stock OM: Antenatal perineal massage for reducing perineal trauma. *Cochrane Database Syst Rev* 2013(4):CD005123.

50. Beetham KS, Giles C, Noetel M, Clifton V, Jones JC, Naughton G: The effects of vigorous intensity exercise in the third trimester of pregnancy: A systematic review and meta-analysis. In., vol. 19; 2019.

51. Beleza ACS, Ferreira C, Meirelles M, Santos C, Nakanno AM: The effect of cryotherapy in perineal pain after vaginal childbirth with episiotomy. *Physiotherapy (united kingdom)* 2011, 97.

52. Bellussi F, Po G, Livi A, Saccone G, De Vivo V, Oliver EA, Berghella V: Fetal Movement Counting and Perinatal Mortality: A Systematic Review and Meta-analysis. *Obstet Gynecol* 2020, 135(2):453-462.

53. Berghella V, Di Mascio D: Evidence-based labor management: before labor (Part 1). In., vol. 2; 2020.

54. Bergman E, Axelsson O, Petzold M, Sonesson C, Kieler H: Self-administered symphysis-fundus measurements analyzed with a novel statistical method for detection of intrauterine growth restriction: a clinical evaluation. *Acta Obstet Gynecol Scand* 2011, 90(8):890-896.

For peer review only - http://bmjopen.bmj.com/site/about/guidelines.xhtml

55. Bergstrom S: External cephalic version and daily post-versional maternal self-assessment of fetal presentation. A prospective study. *Gynecol Obstet Invest* 1992, 33(1):15-18.

56. Berta M, Lindgren H, Christensson K, Mekonnen S, Adefris M: Effect of maternal birth positions on duration of second stage of labor: systematic review and meta-analysis. *BMC Pregnancy Childbirth* 2019, 19(1):466.

57. Bhutta ZA, Rizvi A, Raza F, Hotwani S, Zaidi S, Moazzam Hossain S, Soofi S, Bhutta S: A comparative evaluation of multiple micronutrient and iron-folic acid supplementation during pregnancy in Pakistan: impact on pregnancy outcomes. *Food Nutr Bull* 2009, 30(4 Suppl):S496-505.

58. Biana CB, Cecagno D, Porto AR, Cecagno S, Marques VA, Soares MC: Non-pharmacological therapies applied in pregnancy and labor: an integrative review. *Rev Esc Enferm USP* 2021, 55:e03681.

59. Bilgin CN, Ak B, Ayhan F, Kocyigit F, Yorgun S, Topcuoglu MA: Effect of childbirth education on the perceptions of childbirth and breastfeeding self-efficacy and the obstetric outcomes of nulliparous women. *Health Care Women Int* 2020, 41(2):188-204.

60. Bitzer EM, Schneider A, Wenzlaff P, Hoyme UB, Siegmund-Schultze E: Self-testing of vaginal pH to prevent preterm delivery: a controlled trial. *Dtsch Arztebl Int* 2011, 108(6):81-86.

61. Bornstein J, Ohel G, Sorokin Y, Reape KZ, Shnaider O, Kessary-Shoham H, Ophir E: Effectiveness of a novel home-based testing device for the detection of rupture of membranes. *Am J Perinatol* 2009, 26(1):45-50.

62. Boryri T, Navidian A, Zehi F: Assessing the effect of self-care education on anxiety and depression among pregnant women with a history of spontaneous abortion. In., vol. 9; 2020.

63. Bowen L, Pealing L, Tucker K, McManus RJ, Chappell LC: Adherence with blood pressure self-monitoring in women with pregnancy hypertension, and comparisons to clinic readings: A secondary analysis of OPTIMUM-BP. *Pregnancy Hypertens* 2021, 25:68-74.

64. Brennan M, Young G, Devane D: Topical preparations for preventing stretch marks in pregnancy. *Cochrane Database Syst Rev* 2012, 11:CD000066.

65. Bright KS, Mughal MK, Wajid A, Lane-Smith M, Murray L, Roy N, Van Zanten SV, McNeil DA, Stuart S, Kingston D: Internet-based interpersonal psychotherapy for stress, anxiety, and depression in prenatal women: study protocol for a pilot randomized controlled trial. *Trials* 2019, 20(1):814.

66. Brik M, Fernandez-Buhigas I, Martin-Arias A, Vargas-Terrones M, Barakat R, Santacruz B: Does exercise during pregnancy impact on maternal weight gain and fetal cardiac function? A randomized controlled trial. *Ultrasound Obstet Gynecol* 2019, 53(5):583-589.

67. Brown J, Alwan NA, West J, Brown S, McKinlay CJD, Farrar D, Crowther CA: Lifestyle interventions for the treatment of women with gestational diabetes. In., vol. 2017; 2017.

68. Brustman LE, Langer O, Anyaegbunam A, Belle C, Merkatz IR: Education does not improve patient perception of preterm uterine contractility. *International Journal of Gynecology & Obstetrics* 1991, 34(3):290-290.

69. Buppasiri P, Lumbiganon P, Thinkhamrop J, Ngamjarus C, Laopaiboon M, Medley N: Calcium supplementation (other than for preventing or treating hypertension) for improving pregnancy and infant outcomes. In., vol. 2015; 2015.

70. Byrne J, Hauck Y, Fisher C, Bayes S, Schutze R: Effectiveness of a Mindfulness-Based Childbirth Education pilot study on maternal self-efficacy and fear of childbirth. *J Midwifery Womens Health* 2014, 59(2):192-197.

71. Cahill AG, Haire-Joshu D, Cade WT, Stein RI, Woolfolk CL, Moley K, Mathur A, Schechtman K, Klein S: Weight Control Program and Gestational Weight Gain in Disadvantaged Women with Overweight or Obesity: A Randomized Clinical Trial. *Obesity (Silver Spring)* 2018, 26(3):485-491.

72. Callaghan S: The role of fetal movement counting and 'kick charts' to reduce stillbirths in pregnancies ≥28 weeks' gestation. In., vol. 26; 2018.

73. Callaway LK, McIntyre HD, Barrett HL, Foxcroft K, Tremellen A, Lingwood BE, Tobin JM, Wilkinson S, Kothari A, Morrison M *et al*: Probiotics for the Prevention of Gestational Diabetes Mellitus in Overweight and Obese Women: Findings From the SPRING Double-Blind Randomized Controlled Trial. *Diabetes Care* 2019, 42(3):364-371.

For peer review only - http://bmjopen.bmj.com/site/about/guidelines.xhtml

74. Can HO, Saruhan A: Evaluation of the effects of ice massage applied to large intestine 4 (hegu) on postpartum pain during the active phase of labor. *Iran J Nurs Midwifery Res* 2015, 20(1):129-138.

75. Cankaya S, Simsek B: Effects of Antenatal Education on Fear of Birth, Depression, Anxiety, Childbirth Self-Efficacy, and Mode of Delivery in Primiparous Pregnant Women: A Prospective Randomized Controlled Study. *Clin Nurs Res* 2021, 30(6):818-829.

76. Carducci B, Keats EC, Bhutta ZA: Zinc supplementation for improving pregnancy and infant outcome. In., vol. 2021; 2021.

77. Carlson LM, Harris S, Hardisty EE, Hocutt G, Vargo D, Campbell E, Davis E, Gilmore K, Vora NL: Use of a novel computerized decision aid for aneuploidy screening: a randomized controlled trial. *Genet Med* 2019, 21(4):923-929.

78. Carrascosa MDC, Navas A, Artigues C, Ortas S, Portells E, Soler A, Bennasar-Veny M, Leiva A, Aquanatal T: Effect of aerobic water exercise during pregnancy on epidural use and pain: A multi-centre, randomised, controlled trial. *Midwifery* 2021, 103:103105.

79. Chamberlain C, O'Mara-Eves A, Porter J, Coleman T, Perlen SM, Thomas J, McKenzie JE: Psychosocial interventions for supporting women to stop smoking in pregnancy. In., vol. 2017; 2017.

80. Chang G, McNamara TK, Orav EJ, Koby D, Lavigne A, Ludman B, Vincitorio NA, Wilkins-Haug L: Brief intervention for prenatal alcohol use: a randomized trial. *Obstet Gynecol* 2005, 105(5 Pt 1):991-998.

81. Chankhunaphas W, Charoenkwan K: Effect of elastic abdominal binder on pain and functional recovery after caesarean delivery: a randomised controlled trial. *J Obstet Gynaecol* 2020, 40(4):473-478.

82. Chao AS, Chao A, Wang TH, Chang YC, Peng HH, Chang SD, Chao A, Chang CJ, Lai CH, Wong AMK: Pain relief by applying transcutaneous electrical nerve stimulation (TENS) on acupuncture points during the first stage of labor: a randomized double-blind placebo-controlled trial. *Pain* 2007, 127(3):214-220.

83. Charkamyani F, Hosseinkhani A, Neisani Samani L, Khedmat L: Reducing the Adverse Maternal and Fetal Outcomes in IVF Women by Exercise Interventions During Pregnancy. *Res Q Exerc Sport* 2019, 90(4):589-599.

84. Chatzakis C, Goulis DG, Mareti E, Eleftheriades M, Zavlanos A, Dinas K, Sotiriadis A: Prevention of gestational diabetes mellitus in overweight or obese pregnant women: A network meta-analysis. In., vol. 158; 2019.

85. Chen I, Opiyo N, Tavender E, Mortazhejri S, Rader T, Petkovic J, Yogasingam S, Taljaard M, Agarwal S, Laopaiboon M *et al*: Non-clinical interventions for reducing unnecessary caesarean section. *Cochrane Database Syst Rev* 2018, 9:CD005528.

86. Chen M, Chang Q, Duan T, He J, Zhang L, Liu X: Uterine massage to reduce blood loss after vaginal delivery: a randomized controlled trial. *Obstet Gynecol* 2013, 122(2 Pt 1):290-295.

87. Chen Y, Yue R, Zhang B, Li Z, Shui J, Huang X: Effects of probiotics on blood glucose, biomarkers of inflammation and oxidative stress in pregnant women with gestational diabetes mellitus: A meta-analysis of randomized controlled trials. *Med Clin (Barc)* 2020, 154(6):199-206.

88. Chi YC, Wu CL, Chen CY, Lyu SY, Lo FE, Morisky DE: Randomized trial of a secondhand smoke exposure reduction intervention among hospital-based pregnant women. *Addict Behav* 2015, 41:117-123.

89. Chirakalwasan N, Amnakkittikul S, Wanitcharoenkul E, Charoensri S, Saetung S, Chanprasertyothin S, Chailurkit LO, Panburana P, Bumrungphuet S, Thakkinstian A *et al*: Continuous Positive Airway Pressure Therapy in Gestational Diabetes With Obstructive Sleep Apnea: A Randomized Controlled Trial. *J Clin Sleep Med* 2018, 14(3):327-336.

90. Choi HS, Lee KE, Shin Y: Effects of an emotional regulation program on maladaptive cognitive emotion regulation, stress, anxiety, and postpartum depression among South Korean mothers of premature infants in the neonatal intensive care unit. *Child Health Nurs Res* 2021, 27(1):24-33.

91. Choi J, Lee JH, Vittinghoff E, Fukuoka Y: mHealth Physical Activity Intervention: A Randomized Pilot Study in Physically Inactive Pregnant Women. *Matern Child Health J* 2016, 20(5):1091-1101.

For peer review only - http://bmjopen.bmj.com/site/about/guidelines.xhtml

92. Christian P, Klemm R, Shamim AA, Ali H, Rashid M, Shaikh S, Wu L, Mehra S, Labrique A, Katz J *et al*: Effects of vitamin A and beta-carotene supplementation on birth size and length of gestation in rural Bangladesh: a cluster-randomized trial. *Am J Clin Nutr* 2013, 97(1):188-194.

93. Chughtai A, Navaee M, Hadi M, Yaghoubinia F: Comparing the Effect of Aromatherapy With Essential Oils of Rosa damascena and Lavender Alone and in Combination on Severity of Pain in the First Phase of Labor in Primiparous Women. 2018.

94. Clasen T, Checkley W, Peel JL, Balakrishnan K, McCracken JP, Rosa G, Thompson LM, Barr DB, Clark ML, Johnson MA *et al*: Design and Rationale of the HAPIN Study: A Multicountry Randomized Controlled Trial to Assess the Effect of Liquefied Petroleum Gas Stove and Continuous Fuel Distribution. *Environ Health Perspect* 2020, 128(4):47008.

95. Cluett ER, Burns E, Cuthbert A: Immersion in water during labour and birth. In., vol. 2018; 2018.

96. Coentro VS, Perrella SL, Lai CT, Rea A, Murray K, Geddes DT: Impact of Nipple Shield Use on Milk Transfer and Maternal Nipple Pain. *Breastfeed Med* 2021, 16(3):222-229.

97. Coleman J, Okere M, Seffah J, Kember A, O'Brien LM, Borazjani A, Butler M, Wells J, MacRitchie S, Isaac A *et al*: The Ghana PrenaBelt trial: a double-blind, sham-controlled, randomised clinical trial to evaluate the effect of maternal positional therapy during third-trimester sleep on birth weight. *BMJ Open* 2019, 9(4):e022981.

98. Coleman T, Clark M, Welch C, Whitemore R, Leonardi-Bee J, Cooper S, Hewitt C, Jones M, Sutton S, Watson J *et al*: Effectiveness of offering tailored text message, self-help smoking cessation support to pregnant women who want information on stopping smoking: MiQuit3 randomised controlled trial and meta-analysis. *Addiction* 2022, 117(4):1079-1094.

99. Crawford TJ, Crowther CA, Alsweiler J, Brown J: Antenatal dietary supplementation with myo-inositol in women during pregnancy for preventing gestational diabetes. In., vol. 2015; 2015.

100. Crepinsek MA, Taylor EA, Michener K, Stewart F: Interventions for preventing mastitis after childbirth. In., vol. 2020; 2020.

101. Crockett K, Zlotnick C, Davis M, Payne N, Washington R: A depression preventive intervention for rural low-income African-American pregnant women at risk for postpartum depression. *Arch Womens Ment Health* 2008, 11(5-6):319-325.

102. Cronin RS, Li M, Thompson JMD, Gordon A, Raynes-Greenow CH, Heazell AEP, Stacey T, Culling VM, Bowring V, Anderson NH *et al*: An Individual Participant Data Meta-analysis of Maternal Going-to-Sleep Position, Interactions with Fetal Vulnerability, and the Risk of Late Stillbirth. *EClinicalMedicine* 2019, 10:49-57.

103. Czech I, Fuchs P, Fuchs A, Lorek M, Tobolska-Lorek D, Drosdzol-Cop A, Sikora J: Pharmacological and Non-Pharmacological Methods of Labour Pain Relief-Establishment of Effectiveness and Comparison. *Int J Environ Res Public Health* 2018, 15(12).

104. D'Almeida A, Carter JP, Anatol A, Prost C: Effects of a combination of evening primrose oil (gamma linolenic acid) and fish oil (eicosapentaenoic + docahexaenoic acid) versus magnesium, and versus placebo in preventing pre-eclampsia. *Women Health* 1992, 19(2-3):117-131.

105. da Silva SG, Hallal PC, Domingues MR, Bertoldi AD, Silveira MFD, Bassani D, da Silva ICM, da Silva BGC, Coll CVN, Evenson K: A randomized controlled trial of exercise during pregnancy on maternal and neonatal outcomes: results from the PAMELA study. *Int J Behav Nutr Phys Act* 2017, 14(1):175.

106. Dabiri F, Shahi A: The Effect of LI4 Acupressure on Labor Pain Intensity and Duration of Labor: A Randomized Controlled Trial. *Oman Med J* 2014, 29(6):425-429.

107. Dahlen HG, Homer CS, Cooke M, Upton AM, Nunn R, Brodrick B: Perineal outcomes and maternal comfort related to the application of perineal warm packs in the second stage of labor: a randomized controlled trial. *Birth* 2007, 34(4):282-290.

108. Dai L, Shen Q, Redding SR, Ouyang YQ: Simulation-based childbirth education for Chinese primiparas: A pilot randomized controlled trial. *Patient Educ Couns* 2021, 104(9):2266-2274.

For peer review only - http://bmjopen.bmj.com/site/about/guidelines.xhtml

109. Daley AJ, Macarthur C, Winter H: The role of exercise in treating postpartum depression: a review of the literature. *J Midwifery Womens Health* 2007, 52(1):56-62.
110. Dashtinejad E, Abedi P, Afshari P: Comparison of the effect of breast pump stimulation and oxytocin administration on the length of the third stage of labor, postpartum hemorrhage, and anemia: a randomized controlled trial. *BMC Pregnancy Childbirth* 2018, 18(1):293.
111. Davidson SJ, Barrett HL, Price SA, Callaway LK, Dekker Nitert M: Probiotics for preventing gestational diabetes. In., vol. 2021; 2021.
112. de Vries H, Bakker M, Mullen PD, van Breukelen G: The effects of smoking cessation counseling by midwives on Dutch pregnant women and their partners. *Patient Educ Couns* 2006, 63(1-2):177-187.
113. Dehcheshmeh FS, Rafiei H: Complementary and alternative therapies to relieve labor pain: A comparative study between music therapy and Hoku point ice massage. *Complement Ther Clin Pract* 2015, 21(4):229-232.
114. Deliktas A, Kukulu K: A meta-analysis of the effect on maternal health of upright positions during the second stage of labour, without routine epidural analgesia. In., vol. 74; 2018.
115. Demirel G, Guler H: The Effect of Uterine and Nipple Stimulation on Induction With Oxytocin and the Labor Process. *Worldviews Evid Based Nurs* 2015, 12(5):273-280.
116. Dennis CL, Dowswell T: Interventions (other than pharmacological, psychosocial or psychological) for treating antenatal depression. In., vol. 2013; 2013.
117. Dennis CL, Jackson K, Watson J: Interventions for treating painful nipples among breastfeeding women. In., vol. 2014; 2014.
118. Dennis CL, Schottle N, Hodnett E, McQueen K: An all-purpose nipple ointment versus lanolin in treating painful damaged nipples in breastfeeding women: a randomized controlled trial. *Breastfeed Med* 2012, 7(6):473-479.
119. Dennis CLE: Treatment of postpartum depression, Part 2: A critical review of nonbiological interventions. In., vol. 65; 2004.
120. Dennis CLE: Preventing postpartum depression part I: A review of biological interventions. In., vol. 49; 2004.
121. Desai V, Kozyrskyj AL, Lau S, Sanni O, Dennett L, Walter J, Ospina MB: Effectiveness of Probiotic, Prebiotic, and Synbiotic Supplementation to Improve Perinatal Mental Health in Mothers: A Systematic Review and Meta-Analysis. In., vol. 12; 2021.
122. Dettinger JC, Kinuthia J, Pintye J, Abuna F, Begnel E, Mugwanya K, Sila J, Lagat H, Baeten JM, John-Stewart G: Perinatal outcomes following maternal pre-exposure prophylaxis (PrEP) use during pregnancy: results from a large PrEP implementation program in Kenya. *J Int AIDS Soc* 2019, 22(9):e25378.
123. Deussen AR, Ashwood P, Martis R, Stewart F, Grzeskowiak LE: Relief of pain due to uterine cramping/involution after birth. In., vol. 2020; 2020.
124. Diaz V, Abalos E, Carroli G: Methods for blood loss estimation after vaginal birth. In., vol. 2018; 2018.
125. DiClemente RJ, Wingood GM, Rose E, Sales JM, Crosby RA: Evaluation of an HIV/STD sexual risk-reduction intervention for pregnant African American adolescents attending a prenatal clinic in an urban public hospital: preliminary evidence of efficacy. *J Pediatr Adolesc Gynecol* 2010, 23(1):32-38.
126. DiMarco MA, Menke EM, McNamara T: Evaluating a support group for perinatal loss. *MCN Am J Matern Child Nurs* 2001, 26(3):135-140.
127. Dineva M, Fishpool H, Rayman MP, Mendis J, Bath SC: Systematic review and meta-analysis of the effects of iodine supplementation on thyroid function and child neurodevelopment in mildly-to-moderately iodine-deficient pregnant women. In., vol. 112; 2020.
128. Dodd JM, Turnbull D, McPhee AJ, Deussen AR, Grivell RM, Yelland LN, Crowther CA, Wittert G, Owens JA, Robinson JS *et al*: Antenatal lifestyle advice for women who are overweight or obese: LIMIT randomised trial. *BMJ* 2014, 348:g1285.
129. Dodd V, Chalmers C: Comparing the use of hydrogel dressings to lanolin ointment with lactating mothers. *J Obstet Gynecol Neonatal Nurs* 2003, 32(4):486-494.

For peer review only - http://bmjopen.bmj.com/site/about/guidelines.xhtml

130. Doering JJ, Dogan S: A Postpartum Sleep and Fatigue Intervention Feasibility Pilot Study. *Behav Sleep Med* 2018, 16(2):185-201.

131. Dokmak F, Michalek IM, Boulvain M, Desseauve D: Squatting position in the second stage of labor: A systematic review and meta-analysis. In., vol. 254; 2020.

132. Dougall G, Franssen M, Tucker KL, Yu LM, Hinton L, Rivero-Arias O, Abel L, Allen J, Band RJ, Chisholm A *et al*: Blood pressure monitoring in high-risk pregnancy to improve the detection and monitoring of hypertension (the BUMP 1 and 2 trials): protocol for two linked randomised controlled trials. *BMJ Open* 2020, 10(1):e034593.

133. Downe S, Finlayson K, Melvin C, Spiby H, Ali S, Diggle P, Gyte G, Hinder S, Miller V, Slade P *et al*: Self-hypnosis for intrapartum pain management in pregnant nulliparous women: a randomised controlled trial of clinical effectiveness. *BJOG* 2015, 122(9):1226-1234.

134. Downs DS, Dinallo JM, Birch LL, Paul IM, Ulbrecht JS: Randomized Face-to-Face vs. Home Exercise Interventions in Pregnant Women with Gestational Diabetes. *Psychol Sport Exerc* 2017, 30:73-81.

135. Downs DS, Savage JS, Rivera DE, Pauley AM, Leonard KS, Hohman EE, Guo P, McNitt KM, Stetter C, Kunselman A: Adaptive, behavioral intervention impact on weight gain, physical activity, energy intake, and motivational determinants: results of a feasibility trial in pregnant women with overweight/obesity. *J Behav Med* 2021, 44(5):605-621.

136. Draffin CR, Alderdice FA, McCance DR, Maresh M, Harper R, Patterson CC, Bernatavicius G, Brennan SF, Gough A, McSorley O *et al*: Impact of an educational DVD on anxiety and glycaemic control in women diagnosed with gestational diabetes mellitus (GDM): A randomised controlled trial. *Diabetes Res Clin Pract* 2017, 126:164-171.

137. Dritsa M, Dupuis G, Lowensteyn I, Da Costa D: Effects of home-based exercise on fatigue in postpartum depressed women: who is more likely to benefit and why? *J Psychosom Res* 2009, 67(2):159-163.

138. Dugas M, Shorten A, Dube E, Wassef M, Bujold E, Chaillet N: Decision aid tools to support women's decision making in pregnancy and birth: a systematic review and meta-analysis. *Soc Sci Med* 2012, 74(12):1968-1978.

139. Duncan LG, Cohn MA, Chao MT, Cook JG, Riccobono J, Bardacke N: Benefits of preparing for childbirth with mindfulness training: a randomized controlled trial with active comparison. *BMC Pregnancy Childbirth* 2017, 17(1):140.

140. Dutra LRDV, Araújo AMPdH, Micussi MTABC: Non-pharmacological therapies for postpartum analgesia: a systematic review. *Brazilian Journal Of Pain* 2019, 2(1).

141. East CE, Dolan WJ, Forster DA: Antenatal breast milk expression by women with diabetes for improving infant outcomes. In., vol. 2014; 2014.

142. East CE, Dorward ED, Whale RE, Liu J: Local cooling for relieving pain from perineal trauma sustained during childbirth. *Cochrane Database Syst Rev* 2020, 10(10):CD006304.

143. Ebrahimian A, Rahmani Bilandi R: Comparisons of the Effects of Watching Virtual Reality Videos and Chewing Gum on the Length of Delivery Stages and Maternal Childbirth Satisfaction: A Randomized Controlled Trial. *Iran J Med Sci* 2021, 46(1):15-22.

144. Edwards MJ, Geiser T, Chafin C, Weatherby NL, Smith CM: S.M.A.R.T. mothers are resisting tobacco: prenatal smoking cessation in WIC mothers. *J Allied Health* 2009, 38(3):170-176.

145. Ehrlich SF, Hedderson MM, Brown SD, Sternfeld B, Chasan-Taber L, Feng J, Adams J, Ching J, Crites Y, Quesenberry CP *et al*: Moderate intensity sports and exercise is associated with glycaemic control in women with gestational diabetes. *Diabetes Metab* 2017, 43(5):416-423.

146. Ejike BU, Ohaeri CC, Amaechi EC: Home management practices and its impact on malaria prevalence amongst pregnant women in South-Eastern Nigeria. *Asian Pacific Journal of Tropical Disease* 2017, 7(2):68-70.

147. Ekhtiari YS, Majlessi F, Foroushani AR, Shakibazadeh E: Effect of a self-care educational program based on the health belief model on reducing low birth weight among pregnant Iranian women. *International Journal of Preventive Medicine* 2014, 5(1).

For peer review only - http://bmjopen.bmj.com/site/about/guidelines.xhtml

148. El-Rafie MM, Khafagy GM, Gamal MG: Effect of aerobic exercise during pregnancy on antenatal depression. *Int J Womens Health* 2016, 8:53-57.

149. Epidural, Position Trial Collaborative G: Upright versus lying down position in second stage of labour in nulliparous women with low dose epidural: BUMPES randomised controlled trial. *BMJ* 2017, 359:j4471.

150. Erchick DJ, Agrawal NK, Khatry SK, Katz J, LeClerq SC, Reynolds MA, Mullany LC: Adherence to and acceptability of three alcohol-free, antiseptic oral rinses: A community-based pilot randomized controlled trial among pregnant women in rural Nepal. *Community Dent Oral Epidemiol* 2020, 48(6):501-512.

151. Ershoff DH, Mullen PD, Quinn VP: A randomized trial of a serialized self-help smoking cessation program for pregnant women in an HMO. *Am J Public Health* 1989, 79(2):182-187.

152. Ershoff DH, Quinn VP, Boyd NR, Stern J, Gregory M, Wirtschafter D: The Kaiser Permanente prenatal smoking-cessation trial11The full text of this article is available via AJPM Online at http://www.elsevier.com/locate/ajpmonline. *American Journal of Preventive Medicine* 1999, 17(3):161-168.

153. Espersen T, Klebe JG: Self-monitoring of blood glucose in pregnant diabetics. A comparative study of the blood glucose level and course of pregnancy in pregnant diabetics on an out-patient regime before and after the introduction of methods for home analysis of blood glucose. *Acta Obstet Gynecol Scand* 1985, 64(1):11-14.

154. Facchinetti F, Pedrielli G, Benoni G, Joppi M, Verlato G, Dante G, Balduzzi S, Cuzzolin L: Herbal supplements in pregnancy: unexpected results from a multicentre study. *Hum Reprod* 2012, 27(11):3161-3167.

155. Fathi-Ashtiani A, Ahmadi A, Ghobari-Bonab B, Azizi MP, Saheb-Alzamani SM: Randomized Trial of Psychological Interventions to Preventing Postpartum Depression among Iranian First-time Mothers. *Int J Prev Med* 2015, 6:109.

156. Feder L, Niolon PH, Campbell J, Whitaker DJ, Brown J, Rostad W, Bacon S: An Intimate Partner Violence Prevention Intervention in a Nurse Home Visitation Program: A Randomized Clinical Trial. *J Womens Health (Larchmt)* 2018, 27(12):1482-1490.

157. Feng G, Simpson JA, Chaluluka E, Molyneux ME, Rogerson SJ: Decreasing burden of malaria in pregnancy in Malawian women and its relationship to use of intermittent preventive therapy or bed nets. *PLoS One* 2010, 5(8):e12012.

158. Fernandez L, Cardenas N, Arroyo R, Manzano S, Jimenez E, Martin V, Rodriguez JM: Prevention of Infectious Mastitis by Oral Administration of Lactobacillus salivarius PS2 During Late Pregnancy. *Clin Infect Dis* 2016, 62(5):568-573.

159. Field T, Diego M, Delgado J, Medina L: Yoga and social support reduce prenatal depression, anxiety and cortisol. *J Bodyw Mov Ther* 2013, 17(4):397-403.

160. Flannery C, Fredrix M, Olander EK, McAuliffe FM, Byrne M, Kearney PM: Effectiveness of physical activity interventions for overweight and obesity during pregnancy: A systematic review of the content of behaviour change interventions. In., vol. 16; 2019.

161. Fogarty S, McInerney C, Hay P: Pregnancy-related Pelvic Girdle Pain and Pregnancy Massage: Findings from a Subgroup Analysis of an Observational Study. *Int J Ther Massage Bodywork* 2020, 13(2):1-8.

162. Fokam EB, Ngimuh L, Anchang-Kimbi JK, Wanji S: Assessment of the usage and effectiveness of intermittent preventive treatment and insecticide-treated nets on the indicators of malaria among pregnant women attending antenatal care in the Buea Health District, Cameroon. *Malar J* 2016, 15(1):172.

163. Foudil-Bey I, Murphy MSQ, Dunn S, Keely EJ, El-Chaar D: Evaluating antenatal breastmilk expression outcomes: a scoping review. *Int Breastfeed J* 2021, 16(1):25.

164. Franciscatto LG, Silva CM, Barcellos RB, Angeli S, Silva MS, Almeida SE, Rossetti ML: Comparison of urine and self-collected vaginal samples for detecting human papillomavirus DNA in pregnant women. *Int J Gynaecol Obstet* 2014, 125(1):69-72.

165. Fritel X, de Tayrac R, Bader G, Savary D, Gueye A, Deffieux X, Fernandez H, Richet C, Guilhot J, Fauconnier A: Preventing Urinary Incontinence With Supervised Prenatal Pelvic Floor Exercises: A Randomized Controlled Trial. *Obstet Gynecol* 2015, 126(2):370-377.

For peer review only - http://bmjopen.bmj.com/site/about/guidelines.xhtml

166. Fujioka N, Kobayashi T, Turale S: Short-term behavioral changes in pregnant women after a quit-smoking program via e-learning: a descriptive study from Japan. *Nurs Health Sci* 2012, 14(3):304-311.

167. Gallo RBS, Santana LS, Marcolin AC, Duarte G, Quintana SM: Sequential application of non-pharmacological interventions reduces the severity of labour pain, delays use of pharmacological analgesia, and improves some obstetric outcomes: a randomised trial. *J Physiother* 2018, 64(1):33-40.

168. Gamble C, Ekwaru JP, ter Kuile FO: Insecticide-treated nets for preventing malaria in pregnancy. *Cochrane Database Syst Rev* 2006(2):CD003755.

169. Garmendia ML, Casanello P, Flores M, Kusanovic JP, Uauy R: The effects of a combined intervention (docosahexaenoic acid supplementation and home-based dietary counseling) on metabolic control in obese and overweight pregnant women: the MIGHT study. *Am J Obstet Gynecol* 2021, 224(5):526 e521-526 e525.

170. Garnaes KK, Morkved S, Salvesen KA, Salvesen O, Moholdt T: Exercise training during pregnancy reduces circulating insulin levels in overweight/obese women postpartum: secondary analysis of a randomised controlled trial (the ETIP trial). *BMC Pregnancy Childbirth* 2018, 18(1):18.

171. Garnweidner-Holme L, Henriksen L, Torheim LE, Lukasse M: Effect of the Pregnant+ Smartphone App on the Dietary Behavior of Women With Gestational Diabetes Mellitus: Secondary Analysis of a Randomized Controlled Trial. *JMIR Mhealth Uhealth* 2020, 8(11):e18614.

172. Gau ML, Chang CY, Tian SH, Lin KC: Effects of birth ball exercise on pain and self-efficacy during childbirth: a randomised controlled trial in Taiwan. *Midwifery* 2011, 27(6):e293-300.

173. Gedde-Dahl M, Fors EA: Impact of self-administered relaxation and guided imagery techniques during final trimester and birth. *Complement Ther Clin Pract* 2012, 18(1):60-65.

174. Geisinger ML, Geurs NC, Bain JL, Kaur M, Vassilopoulos PJ, Cliver SP, Hauth JC, Reddy MS: Oral health education and therapy reduces gingivitis during pregnancy. *J Clin Periodontol* 2014, 41(2):141-148.

175. Germeroth LJ, Benno MT, Kolko Conlon RP, Emery RL, Cheng Y, Grace J, Salk RH, Levine MD: Trial design and methodology for a non-restricted sequential multiple assignment randomized trial to evaluate combinations of perinatal interventions to optimize women's health. *Contemp Clin Trials* 2019, 79:111-121.

176. Ghaffari M, Rakhshanderou S, Safari-Moradabadi A, Torabi S: Oral and dental health care during pregnancy: Evaluating a theory-driven intervention. *Oral Dis* 2018, 24(8):1606-1614.

177. Ghahremani L, Alipoor M, Amoee S, Keshavarzi S: Health Promoting Behaviors and Self-efficacy of Physical Activity During Pregnancy: An Interventional Study. *International Journal of Women's Health and Reproduction Sciences* 2017, 5(3):181-185.

178. Ghiasvand F, Riazi H, Hajian S, Kazemi E, Firoozi A: The effect of a self-care program based on the teach back method on the postpartum quality of life. *Electron Physician* 2017, 9(4):4180-4189.

179. Ghouri F, Hollywood A, Ryan K: A systematic review of non-antibiotic measures for the prevention of urinary tract infections in pregnancy. *BMC Pregnancy Childbirth* 2018, 18(1):99.

180. Gielen AC, Windsor R, Faden RR, O'Campo P, Repke J, Davis M: Evaluation of a smoking cessation intervention for pregnant women in an urban prenatal clinic. *Health Educ Res* 1997, 12(2):247-254.

181. Gilad R, Hochner H, Savitsky B, Porat S, Hochner-Celnikier D: Castor oil for induction of labor in post-date pregnancies: A randomized controlled trial. *Women Birth* 2018, 31(1):e26-e31.

182. Gilbert L, Gross J, Lanzi S, Quansah DY, Puder J, Horsch A: How diet, physical activity and psychosocial well-being interact in women with gestational diabetes mellitus: an integrative review. *BMC Pregnancy Childbirth* 2019, 19(1):60.

183. Gilinsky A, Swanson V, Power K: Interventions delivered during antenatal care to reduce alcohol consumption during pregnancy: A systematic review. In., vol. 19; 2011.

184. Gilinsky AS, Dale H, Robinson C, Hughes AR, McInnes R, Lavallee D: Efficacy of physical activity interventions in post-natal populations: systematic review, meta-analysis and content coding of behaviour change techniques. *Health Psychol Rev* 2015, 9(2):244-263.

For peer review only - http://bmjopen.bmj.com/site/about/guidelines.xhtml

185. Gille C, Boer B, Marschal M, Urschitz MS, Heinecke V, Hund V, Speidel S, Tarnow I, Mylonas I, Franz A *et al*: Effect of probiotics on vaginal health in pregnancy. EFFPRO, a randomized controlled trial. *Am J Obstet Gynecol* 2016, 215(5):608 e601-608 e607.

186. Godfrey KM, Barton SJ, El-Heis S, Kenealy T, Nield H, Baker PN, Chong YS, Cutfield W, Chan SY, Ni PSG: Myo-Inositol, Probiotics, and Micronutrient Supplementation From Preconception for Glycemia in Pregnancy: NiPPeR International Multicenter Double-Blind Randomized Controlled Trial. *Diabetes Care* 2021, 44(5):1091-1099.

187. Goetz M, Schiele C, Muller M, Matthies LM, Deutsch TM, Spano C, Graf J, Zipfel S, Bauer A, Brucker SY *et al*: Effects of a Brief Electronic Mindfulness-Based Intervention on Relieving Prenatal Depression and Anxiety in Hospitalized High-Risk Pregnant Women: Exploratory Pilot Study. *J Med Internet Res* 2020, 22(8):e17593.

188. Goksin I, Ayaz-Alkaya S: The effect of progressive muscle relaxation on the postpartum depression risk and general comfort levels in primiparas. *Stress Health* 2020, 36(3):322-329.

189. Gomes F, King SE, Dallmann D, Golan J, da Silva ACF, Hurley KM, Bergeron G, Bourassa MW, Mehta S: Interventions to increase adherence to micronutrient supplementation during pregnancy: a systematic review. *Ann N Y Acad Sci* 2021, 1493(1):41-58.

190. Goodman JH, Guarino A, Chenausky K, Klein L, Prager J, Petersen R, Forget A, Freeman M: CALM Pregnancy: results of a pilot study of mindfulness-based cognitive therapy for perinatal anxiety. *Arch Womens Ment Health* 2014, 17(5):373-387.

191. Grant SM, Wolever TM, O'Connor DL, Nisenbaum R, Josse RG: Effect of a low glycaemic index diet on blood glucose in women with gestational hyperglycaemia. *Diabetes Res Clin Pract* 2011, 91(1):15-22.

192. Green SM, Donegan E, McCabe RE, Streiner DL, Agako A, Frey BN: Cognitive behavioral therapy for perinatal anxiety: A randomized controlled trial. *Aust N Z J Psychiatry* 2020, 54(4):423-432.

193. Grenvik JM, Rosenthal E, Saccone G, Della Corte L, Quist-Nelson J, Gerkin RD, Gimovsky AC, Kwan M, Mercier R, Berghella V: Peanut ball for decreasing length of labor: A systematic review and meta-analysis of randomized controlled trials. *Eur J Obstet Gynecol Reprod Biol* 2019, 242:159-165.

194. Griffith RJ, Alsweiler J, Moore AE, Brown S, Middleton P, Shepherd E, Crowther CA: Interventions to prevent women from developing gestational diabetes mellitus: an overview of Cochrane Reviews. In., vol. 2020; 2020.

195. Griffiths SE, Parsons J, Naughton F, Fulton EA, Tombor I, Brown KE: Are digital interventions for smoking cessation in pregnancy effective? A systematic review and meta-analysis. In., vol. 12; 2018.

196. Grzeskowiak LE, Smith B, Roy A, Dekker GA, Clifton VL: An observational study of the impact of an antenatal asthma management service on asthma control during pregnancy. *Eur J Obstet Gynecol Reprod Biol* 2016, 197:48-53.

197. Guelfi KJ, Ong MJ, Crisp NA, Fournier PA, Wallman KE, Grove JR, Doherty DA, Newnham JP: Regular Exercise to Prevent the Recurrence of Gestational Diabetes Mellitus: A Randomized Controlled Trial. In*: 2016*.

198. Gunther J, Hoffmann J, Kunath J, Spies M, Meyer D, Stecher L, Rosenfeld E, Kick L, Rauh K, Hauner H: Effects of a Lifestyle Intervention in Routine Care on Prenatal Dietary Behavior-Findings from the Cluster-Randomized GeliS Trial. *J Clin Med* 2019, 8(7).

199. Guo W, Zhang B, Wang X: Lifestyle interventions for gestational diabetes mellitus to control blood glucose: a meta-analysis of randomized studies. *International Journal of Diabetes in Developing Countries* 2017, 38(1):26-35.

200. Gupta JK, Sood A, Hofmeyr GJ, Vogel JP: Position in the second stage of labour for women without epidural anaesthesia. In., vol. 2017; 2017.

201. Gustafsson MK, Romundstad PR, Stafne SN, Helvik AS, Stunes AK, Morkved S, Salvesen KA, Thorsby PM, Mosti MP, Syversen U: The effect of an exercise program in pregnancy on vitamin D status among healthy, pregnant Norwegian women: a randomized controlled trial. *BMC Pregnancy Childbirth* 2019, 19(1):76.

202. Gutke A, Betten C, Degerskär K, Pousette S, Fagevik Olsén M: Treatments for pregnancy-related lumbopelvic pain: A systematic review of physiotherapy modalities. In., vol. 94; 2015.

For peer review only - http://bmjopen.bmj.com/site/about/guidelines.xhtml

203. Hagan R, Evans SF, Pope S: Preventing postnatal depression in mothers of very preterm infants: a randomised controlled trial. *BJOG* 2004, 111(7):641-647.

204. Haghighat M, Mirghafourvand M, Mohammad-Alizadeh-Charandabi S, Malakouti J, Erfani M: The Effect of Spiritual Counseling on Stress and Anxiety in Pregnancy: A Randomized Controlled Clinical Trial. *Iranian Red Crescent Medical Journal* 2018, In Press(In Press).

205. Haire-Joshu D, Cahill AG, Stein RI, Cade WT, Woolfolk CL, Moley K, Mathur A, Schwarz CD, Schechtman KB, Klein S: Randomized Controlled Trial of Home-Based Lifestyle Therapy on Postpartum Weight in Underserved Women with Overweight or Obesity. *Obesity (Silver Spring)* 2019, 27(4):535-541.

206. Hajek P, West R, Lee A, Foulds J, Owen L, Eiser JR, Main N: Randomized controlled trial of a midwife-delivered brief smoking cessation intervention in pregnancy. *Addiction* 2001, 96(3):485-494.

207. Halse RE, Wallman KE, Dimmock JA, Newnham JP, Guelfi KJ: Home-Based Exercise Improves Fitness and Exercise Attitude and Intention in Women with GDM. *Med Sci Sports Exerc* 2015, 47(8):1698-1704.

208. Halvorsen S, Haakstad LA, Edvardsen E, Bo K: Effect of aerobic dance on cardiorespiratory fitness in pregnant women: a randomised controlled trial. *Physiotherapy* 2013, 99(1):42-48.

209. Hannover W, Thyrian JR, Roske K, Grempler J, Rumpf HJ, John U, Hapke U: Smoking cessation and relapse prevention for postpartum women: results from a randomized controlled trial at 6, 12, 18 and 24 months. *Addict Behav* 2009, 34(1):1-8.

210. Harding KB, Peña-Rosas JP, Webster AC, Yap CMY, Payne BA, Ota E, De-Regil LM: Iodine supplementation for women during the preconception, pregnancy and postpartum period. In., vol. 2017; 2017.

211. Hardy I, Lloyd A, Morisset AS, Camirand Lemyre F, Baillargeon JP, Fraser WD: Healthy for My Baby Research Protocol- a Randomized Controlled Trial Assessing a Preconception Intervention to Improve the Lifestyle of Overweight Women and Their Partners. *Front Public Health* 2021, 9:670304.

212. Harrison CL, Lombard CB, Teede HJ: Limiting postpartum weight retention through early antenatal intervention: the HeLP-her randomised controlled trial. *Int J Behav Nutr Phys Act* 2014, 11(1):134.

213. Haruna M, Matsuzaki M, Ota E, Shiraishi M, Hanada N, Mori R: Guided imagery for treating hypertension in pregnancy. *Cochrane Database Syst Rev* 2019, 4(4):CD011337.

214. Haugland KS, Rasmussen S, Daltveit AK: Group intervention for women with pelvic girdle pain in pregnancy. A randomized controlled trial. *Acta Obstet Gynecol Scand* 2006, 85(11):1320-1326.

215. Haver J, Ansari N, Zainullah P, Kim YM, Tappis H: Misoprostol for Prevention of Postpartum Hemorrhage at Home Birth in Afghanistan: Program Expansion Experience. *J Midwifery Womens Health* 2016, 61(2):196-202.

216. Hawkins JS, Casey BM, Lo JY, Moss K, McIntire DD, Leveno KJ: Weekly compared with daily blood glucose monitoring in women with diet-treated gestational diabetes. *Obstet Gynecol* 2009, 113(6):1307-1312.

217. Heffron R, Mugo N, Hong T, Celum C, Marzinke MA, Ngure K, Asiimwe S, Katabira E, Bukusi EA, Odoyo J *et al*: Pregnancy outcomes and infant growth among babies with in-utero exposure to tenofovir-based preexposure prophylaxis for HIV prevention. *AIDS* 2018, 32(12):1707-1713.

218. Heller HM, Hoogendoorn AW, Honig A, Broekman BFP, van Straten A: The Effectiveness of a Guided Internet-Based Tool for the Treatment of Depression and Anxiety in Pregnancy (MamaKits Online): Randomized Controlled Trial. *J Med Internet Res* 2020, 22(3):e15172.

219. Henrique AJ, Gabrielloni MC, Rodney P, Barbieri M: Non-pharmacological interventions during childbirth for pain relief, anxiety, and neuroendocrine stress parameters: A randomized controlled trial. *Int J Nurs Pract* 2018, 24(3):e12642.

220. Herbec A, Brown J, Tombor I, Michie S, West R: Pilot randomized controlled trial of an internet-based smoking cessation intervention for pregnant smokers ('MumsQuit'). *Drug Alcohol Depend* 2014, 140:130-136.

221. Herring SJ, Cruice JF, Bennett GG, Rose MZ, Davey A, Foster GD: Preventing excessive gestational weight gain among African American women: A randomized clinical trial. *Obesity (Silver Spring)* 2016, 24(1):30-36.

For peer review only - http://bmjopen.bmj.com/site/about/guidelines.xhtml

222. Heslehurst N, Hayes L, Jones D, Newham J, Olajide J, McLeman L, McParlin C, de Brun C, Azevedo L: The effectiveness of smoking cessation, alcohol reduction, diet and physical activity interventions in changing behaviours during pregnancy: A systematic review of systematic reviews. *PLoS One* 2020, 15(5):e0232774.
223. Hill B, Skouteris H, Fuller-Tyszkiewicz M: Interventions designed to limit gestational weight gain: a systematic review of theory and meta-analysis of intervention components. *Obes Rev* 2013, 14(6):435-450.
224. Hirshberg A, Downes K, Srinivas S: Comparing standard office-based follow-up with text-based remote monitoring in the management of postpartum hypertension: a randomised clinical trial. *BMJ Qual Saf* 2018, 27(11):871-877.
225. Ho M, Chang YY, Chang WC, Lin HC, Wang MH, Lin WC, Chiu TH: Oral Lactobacillus rhamnosus GR-1 and Lactobacillus reuteri RC-14 to reduce Group B Streptococcus colonization in pregnant women: A randomized controlled trial. *Taiwan J Obstet Gynecol* 2016, 55(4):515-518.
226. Hofmeyr GJ, Abdel-Aleem H, Abdel-Aleem MA: Uterine massage for preventing postpartum haemorrhage. *Cochrane Database Syst Rev* 2013(7):CD006431.
227. Hofmeyr GJ, Lawrie TA, Atallah ÁN, Torloni MR: Calcium supplementation during pregnancy for preventing hypertensive disorders and related problems. In., vol. 2018; 2018.
228. Hofmeyr GJ, Manyame S, Medley N, Williams MJ: Calcium supplementation commencing before or early in pregnancy, for preventing hypertensive disorders of pregnancy. *Cochrane Database Syst Rev* 2019, 9:CD011192.
229. Holden SC, Manor B, Zhou J, Zera C, Davis RB, Yeh GY: Prenatal Yoga for Back Pain, Balance, and Maternal Wellness: A Randomized, Controlled Pilot Study. *Glob Adv Health Med* 2019, 8:2164956119870984.
230. Hollenbach D, Broker R, Herlehy S, Stuber K: Non-pharmacological interventions for sleep quality and insomnia during pregnancy: A systematic review. *J Can Chiropr Assoc* 2013, 57(3):260-270.
231. Homko CJ, Sivan E, Reece EA: The impact of self-monitoring of blood glucose on self-efficacy and pregnancy outcomes in women with diet-controlled gestational diabetes. *The Diabetes educator* 2002, 28(3).
232. Howarth AM, Swain NR: Skills-based childbirth preparation increases childbirth self-efficacy for first time mothers. *Midwifery* 2019, 70:100-105.
233. Howell EA, Balbierz A, Wang J, Parides M, Zlotnick C, Leventhal H: Reducing postpartum depressive symptoms among black and Latina mothers: a randomized controlled trial. *Obstet Gynecol* 2012, 119(5):942-949.
234. Howell EA, Bodnar-Deren S, Balbierz A, Loudon H, Mora PA, Zlotnick C, Wang J, Leventhal H: An intervention to reduce postpartum depressive symptoms: a randomized controlled trial. *Arch Womens Ment Health* 2014, 17(1):57-63.
235. Hsiao WH, Paterno MT, Iradukunda F, Hawkins M: The Preliminary Efficacy of a Sleep Self-management Intervention Using a Personalized Health Monitoring Device during Pregnancy. *Behav Sleep Med* 2021, 19(6):705-716.
236. Hu Y, Lu H, Huang J, Zang Y: Efficacy and safety of non-pharmacological interventions for labour pain management: A systematic review and Bayesian network meta-analysis. In., vol. 30; 2021.
237. Huang CM, Wu HL, Huang SH, Chien LY, Guo JL: Transtheoretical model-based passive smoking prevention programme among pregnant women and mothers of young children. *Eur J Public Health* 2013, 23(5):777-782.
238. Huang TT, Yeh CY, Tsai YC: A diet and physical activity intervention for preventing weight retention among Taiwanese childbearing women: a randomised controlled trial. *Midwifery* 2011, 27(2):257-264.
239. Hughes BL, Gans KM, Raker C, Hipolito ER, Rouse DJ: A brief prenatal intervention of behavioral change to reduce the risk of maternal cytomegalovirus: A randomized controlled trial. In*: 2017*.
240. Huhn EA, Linder T, Eppel D, Weisshaupt K, Klapp C, Schellong K, Henrich W, Yerlikaya-Schatten G, Rosicky I, Husslein P *et al*: Effectiveness of real-time continuous glucose monitoring to improve glycaemic control and pregnancy outcome in patients with gestational diabetes mellitus: a study protocol for a randomised controlled trial. *BMJ Open* 2020, 10(11):e040498.

For peer review only - http://bmjopen.bmj.com/site/about/guidelines.xhtml

241. Hui AL, Ludwig S, Gardiner P, Sevenhuysen G, Murray R, Morris M, Shen GX: Community-based Exercise and Dietary Intervention During Pregnancy:A Pilot Study. *Canadian Journal of Diabetes* 2006, 30(2):1-7.
242. Hussein N, Kai J, Qureshi N: The effects of preconception interventions on improving reproductive health and pregnancy outcomes in primary care: A systematic review. In., vol. 22; 2016.
243. Huvinen E, Koivusalo SB, Meinila J, Valkama A, Tiitinen A, Rono K, Stach-Lempinen B, Eriksson JG: Effects of a Lifestyle Intervention During Pregnancy and First Postpartum Year: Findings From the RADIEL Study. *J Clin Endocrinol Metab* 2018, 103(4):1669-1677.
244. Ibrahim HA-F, Said HII, Elgzar WTI: Effect of upright and ambulant positions versus lying down during the active first stage of labor on birth outcomes among nulliparous women: randomized controlled clinical trial. *Frontiers of Nursing* 2020, 7(3):239-248.
245. Ip WY, Tang CS, Goggins WB: An educational intervention to improve women's ability to cope with childbirth. *J Clin Nurs* 2009, 18(15):2125-2135.
246. Isbir GG, Inci F, Onal H, Yildiz PD: The effects of antenatal education on fear of childbirth, maternal self-efficacy and post-traumatic stress disorder (PTSD) symptoms following childbirth: an experimental study. *Appl Nurs Res* 2016, 32:227-232.
247. Islam S, Mohanty SK: Understanding the association between gradient of cooking fuels and low birth weight in India. *SSM Popul Health* 2021, 13:100732.
248. Jackson KT, Dennis CL: Lanolin for the treatment of nipple pain in breastfeeding women: a randomized controlled trial. *Matern Child Nutr* 2017, 13(3).
249. Jahanfar S, Jaafar SH: Effects of restricted caffeine intake by mother on fetal, neonatal and pregnancy outcomes. In., vol. 2015; 2015.
250. Jalambadani Z, Borji A, Bakaeian M: Examining the effect of mindfulness-based art therapy (MBAT) on stress and lifestyle of Iranian pregnant women. *J Obstet Gynaecol* 2020, 40(6):779-783.
251. Jallo N, Thacker LR, 2nd, Menzies V, Stojanovic P, Svikis DS: A Stress Coping App for Hospitalized Pregnant Women at Risk for Preterm Birth. *MCN Am J Matern Child Nurs* 2017, 42(5):257-262.
252. Jarde A, Lewis-Mikhael AM, Moayyedi P, Stearns JC, Collins SM, Beyene J, McDonald SD: Pregnancy outcomes in women taking probiotics or prebiotics: a systematic review and meta-analysis. *BMC Pregnancy Childbirth* 2018, 18(1):14.
253. Jasemzadeh M, Khafaie MA, Jaafarzadeh N, Araban M: Effectiveness of a theory-based mobile phone text message intervention for improving protective behaviors of pregnant women against air pollution: a randomized controlled trial. *Environ Sci Pollut Res Int* 2018, 25(7):6648-6655.
254. Jesse DE, Gaynes BN, Feldhousen EB, Newton ER, Bunch S, Hollon SD: Performance of a Culturally Tailored Cognitive-Behavioral Intervention Integrated in a Public Health Setting to Reduce Risk of Antepartum Depression: A Randomized Controlled Trial. *J Midwifery Womens Health* 2015, 60(5):578-592.
255. Johnsen M, Klingenberg C, Brand M, Revhaug A, Andreassen G: Antenatal breastmilk expression for women with diabetes in pregnancy - a feasibility study. *Int Breastfeed J* 2021, 16(1):56.
256. Jois RS, Tan JK, Silva D: Do probiotics in pregnancy reduce the risk of group B streptococcal colonisation? *J Paediatr Child Health* 2020, 56(9):1468-1472.
257. Jones DL, Rodriguez VJ, Mandell LN, Lee TK, Weiss SM, Peltzer K: Sexual risk behavior among HIV-infected women in South Africa at 12-months postpartum. *AIDS Care* 2019, 31(9):1114-1123.
258. Jones LV, Ray A, Moy FM, Buckley BS: Techniques of monitoring blood glucose during pregnancy for women with pre-existing diabetes. In., vol. 2019; 2019.
259. Jones MH, Barik S, Mangune HH, Jones P, Gregory SJ, Spring JE: Do birth plans adversely affect the outcome of labour? *British Journal of Midwifery* 1998, 6(1):38-41.
260. Jung ME, Stork MJ, Stapleton J, Bourne JE, Martin Ginis KA: A systematic review of behavioural interventions to increase maternal calcium intake. In., vol. 12; 2016.

For peer review only - http://bmjopen.bmj.com/site/about/guidelines.xhtml

261. Kabanywanyi AM, Macarthur JR, Stolk WA, Habbema JD, Mshinda H, Bloland PB, Abdulla S, Kachur SP: Malaria in pregnant women in an area with sustained high coverage of insecticide-treated bed nets. *Malar J* 2008, 7:133.

262. Kalafat E, Leslie K, Bhide A, Thilaganathan B, Khalil A: Pregnancy outcomes following home blood pressure monitoring in gestational hypertension. *Pregnancy Hypertens* 2019, 18:14-20.

263. Kalafat E, Mir I, Perry H, Thilaganathan B, Khalil A: Is home blood-pressure monitoring in hypertensive disorders of pregnancy consistent with clinic recordings? *Ultrasound Obstet Gynecol* 2018, 52(4):515-521.

264. Kantrowitz-Gordon I, McCurry SM, Landis CA, Lee R, Wi D: Online prenatal trial in mindfulness sleep management (OPTIMISM): protocol for a pilot randomized controlled trial. *Pilot Feasibility Stud* 2020, 6(1):128.

265. Kardel KR, Henriksen T, Iversen PO: No effect of energy supply during childbirth on delivery outcomes in nulliparous women: a randomised, double-blind, placebo-controlled trial. *J Obstet Gynaecol* 2010, 30(3):248-252.

266. Karimi F, Babazadeh R, Roudsari RL, Asgharipour N, Esmaily H: Comparing the Effectiveness of Sexual Counseling Based on PLISSITand BETTER Models on Sexual Selfdisclosure in Women with Sexual Problems after Childbirth: A Randomized Trial. *Iran J Nurs Midwifery Res* 2021, 26(1):68-74.

267. Katz J, Tielsch JM, Khatry SK, Shrestha L, Breysse P, Zeger SL, Kozuki N, Checkley W, LeClerq SC, Mullany LC: Impact of Improved Biomass and Liquid Petroleum Gas Stoves on Birth Outcomes in Rural Nepal: Results of 2 Randomized Trials. *Glob Health Sci Pract* 2020, 8(3):372-382.

268. Kavanagh J, Kelly AJ, Thomas J: Sexual intercourse for cervical ripening and induction of labour. *Cochrane Database Syst Rev* 2001(2):CD003093.

269. Kavanagh J, Kelly AJ, Thomas J: Breast stimulation for cervical ripening and induction of labour. In., vol. 2010; 2005.

270. Kazemi A, Ehsanpour S, Zahraei NSN, Hasanzadeh A, Beigi NMA, Malverdi Z: Impact of health belief modification on intention to make smoke free home among pregnant women. *Journal of Research in Medical Sciences* 2011, 16(6).

271. Keats EC, Akseer N, Thurairajah P, Cousens S, Bhutta ZA, Global Young Women's Nutrition Investigators G: Multiple-micronutrient supplementation in pregnant adolescents in low- and middle-income countries: a systematic review and a meta-analysis of individual participant data. *Nutr Rev* 2022, 80(2):141-156.

272. Keller C, Records K, Ainsworth B, Permana P, Coonrod DV: Interventions for weight management in postpartum women. In., vol. 37; 2008.

273. Kelly AJ, Kavanagh J, Thomas J: Castor oil, bath and/or enema for cervical priming and induction of labour. In., vol. 2013; 2013.

274. Kennelly MA, Ainscough K, Lindsay KL, O'Sullivan E, Gibney ER, McCarthy M, Segurado R, DeVito G, Maguire O, Smith T *et al*: Pregnancy exercise and nutrition with smartphone application support a randomized controlled trial. In*: 2018*.

275. Khatun F, Lee TW, Lee HJ, Park J, Song JE, Kim S: Does a nurse-led postpartum self-care program for first-time mothers in Bangladesh improve postpartum fatigue, depressive mood, and maternal functioning?: a non-synchronized quasi-experimental study. *Korean Journal of Women Health Nursing* 2021, 27(3):196-208.

276. Kim HK, Niederdeppe J, Graham M, Olson C, Gay G: Effects of Online Self-Regulation Activities on Physical Activity Among Pregnant and Early Postpartum Women. *J Health Commun* 2015, 20(10):1115-1124.

277. Kimber L, McNabb M, Mc Court C, Haines A, Brocklehurst P: Massage or music for pain relief in labour: a pilot randomised placebo controlled trial. *Eur J Pain* 2008, 12(8):961-969.

278. King SE, Yeh PT, Rhee DK, Tuncalp O, Rogers LM, Narasimhan M: Self-management of iron and folic acid supplementation during pre-pregnancy, pregnancy and postnatal periods: a systematic review. *BMJ Glob Health* 2021, 6(5).

For peer review only - http://bmjopen.bmj.com/site/about/guidelines.xhtml

279. Kinser P, Jallo N, Huberty J, Jones E, Thacker L, Moyer S, Laird B, Rider A, Lanni S, Drozd F *et al*: Study protocol for a multisite randomized controlled trial of an internet and mobile-based intervention for preventing and reducing perinatal depressive symptoms. *Res Nurs Health* 2021, 44(1):13-23.

280. Kinser PA, Thacker LR, Rider A, Moyer S, Amstadter AB, Mazzeo SE, Bodnar-Deren S, Starkweather A: Feasibility, Acceptability, and Preliminary Effects of "Mindful Moms": A Mindful Physical Activity Intervention for Pregnant Women with Depression. *Nurs Res* 2021, 70(2):95-105.

281. Kirca AS, Kanza Gul D: Effects of self-acupressure on pregnancy-related constipation: A single-blind randomized controlled study. *Explore (NY)* 2021, 17(5):463-468.

282. Kirihara N, Kamitomo M, Tabira T, Hashimoto T, Taniguchi H, Maeda T: Effect of probiotics on perinatal outcome in patients at high risk of preterm birth. *J Obstet Gynaecol Res* 2018, 44(2):241-247.

283. Kitt JA, Fox RL, Cairns AE, Mollison J, Burchert HH, Kenworthy Y, McCourt A, Suriano K, Lewandowski AJ, Mackillop L *et al*: Short-Term Postpartum Blood Pressure Self-Management and Long-Term Blood Pressure Control: A Randomized Controlled Trial. *Hypertension* 2021, 78(2):469-479.

284. Kobayashi M, Ogawa K, Morisaki N, Tani Y, Horikawa R, Fujiwara T: Dietary n-3 Polyunsaturated Fatty Acids in Late Pregnancy and Postpartum Depressive Symptom among Japanese Women. *Front Psychiatry* 2017, 8(NOV):241.

285. Koivusalo SB, Rono K, Klemetti MM, Roine RP, Lindstrom J, Erkkola M, Kaaja RJ, Poyhonen-Alho M, Tiitinen A, Huvinen E *et al*: Gestational Diabetes Mellitus Can Be Prevented by Lifestyle Intervention: The Finnish Gestational Diabetes Prevention Study (RADIEL): A Randomized Controlled Trial. *Diabetes Care* 2016, 39(1):24-30.

286. Kolivand M, Rahimi MA, Keramat A, Shariati M, Emamian MH: Effect of a new self-care guide package on maternal and neonatal outcomes in gestational diabetes: A randomized control trial. *J Diabetes* 2019, 11(2):139-147.

287. Kolivand M, Rahimi MA, Shariati M, Keramat A, Emamian MH: The Effect of Self-care Educational/Training Interventions on the Outcomes of Gestational Diabetes: A Review Article. *Iran J Public Health* 2018, 47(12):1805-1815.

288. Korn L, Koren G, Yaakov A, Madar G, Blau A: Evaluating the Effectiveness of Childbirth Preparation Courses on Women's Self-Efficacy among Ultra-Orthodox Jewish Religious Women in Israel. *Healthcare (Basel)* 2021, 9(7).

289. Korte JE, Kisa R, Vrana-Diaz CJ, Malek AM, Buregyeya E, Matovu JKB, Kagaayi J, Musoke W, Chemusto H, Mukama SC *et al*: HIV Oral Self-Testing for Male Partners of Women Attending Antenatal Care in Central Uganda: Uptake of Testing and Linkage to Care in a Randomized Trial. *J Acquir Immune Defic Syndr* 2020, 84(3):271-279.

290. Koshida S, Tokoro S, Katsura D, Tsuji S, Murakami T, Takahashi K: Fetal movement counting is associated with the reduction of delayed maternal reaction after perceiving decreased fetal movements: a prospective study. *Sci Rep* 2021, 11(1):10818.

291. Koyuncu SB, Bulbul M: The impact of yoga on fear of childbirth and childbirth self-efficacy among third trimester pregnants. *Complement Ther Clin Pract* 2021, 44:101438.

292. Krauss-Silva L, Moreira ME, Alves MB, Braga A, Camacho KG, Batista MR, Almada-Horta A, Rebello MR, Guerra F: A randomised controlled trial of probiotics for the prevention of spontaneous preterm delivery associated with bacterial vaginosis: preliminary results. *Trials* 2011, 12:239.

293. Kumar R, Farzeen M, Hafeez A, Achakzai BK, Vankwani M, Lal M, Iqbal R, Somrongthong R: Effectiveness of a health education intervention on the use of long-lasting insecticidal nets for the prevention of malaria in pregnant women of Pakistan: a quasi-experimental study. *Malar J* 2020, 19(1):232.

294. Kuppermann M, Kaimal AJ, Blat C, Gonzalez J, Thiet MP, Bermingham Y, Altshuler AL, Bryant AS, Bacchetti P, Grobman WA: Effect of a Patient-Centered Decision Support Tool on Rates of Trial of Labor After Previous Cesarean Delivery: The PROCEED Randomized Clinical Trial. *JAMA* 2020, 323(21):2151-2159.

For peer review only - http://bmjopen.bmj.com/site/about/guidelines.xhtml

295. Kwena Z, Kimbo L, Darbes LA, Hatcher AM, Helova A, Owino G, Thirumurthy H, Bukusi EA, Braun T, Kilgore M *et al*: Testing strategies for couple engagement in prevention of mother-to-child transmission of HIV and family health in Kenya: study protocol for a randomized controlled trial. *Trials* 2021, 22(1):19.
296. Labrecque M, Eason E, Marcoux S: Randomized trial of perineal massage during pregnancy: perineal symptoms three months after delivery. *Am J Obstet Gynecol* 2000, 182(1 Pt 1):76-80.
297. Labrecque M, Nouwen A, Bergeron M, Rancourt JF: A randomized controlled trial of nonpharmacologic approaches for relief of low back pain during labor. *J Fam Pract* 1999, 48(4):259-263.
298. Landon MB, Spong CY, Thom E, Carpenter MW, Ramin SM, Casey B, Wapner RJ, Varner MW, Rouse DJ, Thorp JM, Jr. *et al*: A multicenter, randomized trial of treatment for mild gestational diabetes. *N Engl J Med* 2009, 361(14):1339-1348.
299. Lanou H, Huybregts L, Roberfroid D, Nikiema L, Kouanda S, Van Camp J, Kolsteren P: Prenatal nutrient supplementation and postnatal growth in a developing nation: an RCT. *Pediatrics* 2014, 133(4):e1001-1008.
300. Lanssens D, Thijs IM, Gyselaers W, consortium PI: Design of the Pregnancy REmote MOnitoring II study (PREMOM II): a multicenter, randomized controlled trial of remote monitoring for gestational hypertensive disorders. *BMC Pregnancy Childbirth* 2020, 20(1):626.
301. Lauzon L, Hodnett ED, Kellie FJ: Antenatal education for self-diagnosis of the onset of active labour at term. In., vol. 2017; 1998.
302. Lawrence T, Aveyard P, Cheng KK, Griffin C, Johnson C, Croghan E: Does stage-based smoking cessation advice in pregnancy result in long-term quitters? 18-month postpartum follow-up of a randomized controlled trial. *Addiction* 2005, 100(1):107-116.
303. Lee JT, Tsai JL: Transtheoretical model-based postpartum sexual health education program improves women's sexual behaviors and sexual health. *J Sex Med* 2012, 9(4):986-996.
304. Lee M, Miller SM, Wen KY, Hui SK, Roussi P, Hernandez E: Cognitive-behavioral intervention to promote smoking cessation for pregnant and postpartum inner city women. *J Behav Med* 2015, 38(6):932-943.
305. Lertbunnaphong T, Lapthanapat N, Leetheeragul J, Hakularb P, Ownon A: Postpartum blood loss: visual estimation versus objective quantification with a novel birthing drape. *Singapore Med J* 2016, 57(6):325-328.
306. Leung SS, Lam TH: Group antenatal intervention to reduce perinatal stress and depressive symptoms related to intergenerational conflicts: a randomized controlled trial. *Int J Nurs Stud* 2012, 49(11):1391-1402.
307. Levitt C, Shaw E, Wong S, Kaczorowski J: Systematic review of the literature on postpartum care: Effectiveness of interventions for smoking relapse prevention, cessation, and reduction in postpartum women. In., vol. 34; 2007.
308. Lim K, Chan SY, Lim SL, Tai BC, Tsai C, Wong SR, Ang SM, Yew TW, Tai ES, Yong EL: A Smartphone App to Restore Optimal Weight (SPAROW) in Women With Recent Gestational Diabetes Mellitus: Randomized Controlled Trial. *JMIR Mhealth Uhealth* 2021, 9(3):e22147.
309. Lim S, Hill B, Pirotta S, O'Reilly S, Moran L: What Are the Most Effective Behavioural Strategies in Changing Postpartum Women's Physical Activity and Healthy Eating Behaviours? A Systematic Review and Meta-Analysis. *J Clin Med* 2020, 9(1).
310. Lim S, O'Reilly S, Behrens H, Skinner T, Ellis I, Dunbar JA: Effective strategies for weight loss in post-partum women: A systematic review and meta-analysis. In., vol. 16; 2015.
311. Lin YC, Gau ML, Kao GH, Lee HC: Efficacy of an Ergonomic Ankle Support Aid for Squatting Position in Improving Pushing Skills and Birth Outcomes During the Second Stage of Labor: A Randomized Controlled Trial. *J Nurs Res* 2018, 26(6):376-384.
312. Linden K, Berg M, Adolfsson A, Sparud-Lundin C: Person-centred, web-based support in pregnancy and early motherhood for women with Type 1 diabetes mellitus: a randomized controlled trial. *Diabet Med* 2018, 35(2):232-241.
313. Liston RM, Bloom K, Zimmer P: The psychological effects of counting fetal movements. *Birth* 1994, 21(3):135-140.

For peer review only - http://bmjopen.bmj.com/site/about/guidelines.xhtml

314. Liu J, Wilcox S, Wingard E, Turner-McGrievy G, Hutto B, Burgis J: A Behavioral Lifestyle Intervention to Limit Gestational Weight Gain in Pregnant Women with Overweight and Obesity. *Obesity (Silver Spring)* 2021, 29(4):672-680.

315. Liu N, Wang J, Chen Dd, Sun Wj, Li P, Zhang W: Effects of exercise on pregnancy and postpartum fatigue: A systematic review and meta-analysis. In., vol. 253; 2020.

316. Liu P, Wen W, Yu KF, Gao X, Lo ECM, Wong MCM: Effectiveness of a family-centered behavioral and educational counselling approach to improve periodontal health of pregnant women: a randomized controlled trial. *BMC Oral Health* 2020, 20(1):284.

317. Liu YH, Lee CS, Yu CH, Chen CH: Effects of music listening on stress, anxiety, and sleep quality for sleep-disturbed pregnant women. *Women Health* 2016, 56(3):296-311.

318. Llorente AM, Jensen CL, Voigt RG, Fraley JK, Berretta MC, Heird WC: Effect of maternal docosahexaenoic acid supplementation on postpartum depression and information processing. *Am J Obstet Gynecol* 2003, 188(5):1348-1353.

319. Logan S, Browne J, McKenzie H, Templeton A, Bhattacharya S: Evaluation of endocervical, first-void urine and self-administered vulval swabs for the detection of Chlamydia trachomatis in a miscarriage population. *BJOG* 2005, 112(1):103-106.

320. Loughnan SA, Sie A, Hobbs MJ, Joubert AE, Smith J, Haskelberg H, Mahoney AEJ, Kladnitski N, Holt CJ, Milgrom J *et al*: A randomized controlled trial of 'MUMentum Pregnancy': Internet-delivered cognitive behavioral therapy program for antenatal anxiety and depression. *J Affect Disord* 2019, 243:381-390.

321. Loukopoulou AN, Vardavas CI, Farmakides G, Rosolymos C, Chrelias C, Tzatzarakis M, Tsatsakis A, Myridakis A, Lyberi M, Behrakis PK: Counselling for smoking cessation during pregnancy reduces tobacco-specific nitrosamine (NNAL) concentrations: A randomized controlled trial. *Eur J Midwifery* 2018, 2(November):14.

322. Lowndes TA, Egan SJ, McEvoy PM: Efficacy of brief guided self-help cognitive behavioral treatment for perfectionism in reducing perinatal depression and anxiety: a randomized controlled trial. *Cogn Behav Ther* 2019, 48(2):106-120.

323. Lucas R, Zhang Y, Walsh SJ, Evans H, Young E, Starkweather A: Efficacy of a Breastfeeding Pain Self-Management Intervention: A Pilot Randomized Controlled Trial. *Nurs Res* 2019, 68(2):E1-E10.

324. Lucena L, Frange C, Pinto ACA, Andersen ML, Tufik S, Hachul H: Mindfulness interventions during pregnancy: A narrative review. In., vol. 18; 2020.

325. Maddady SM, Charandabi SM-A, Shafaei FS, Mirghafourvand M: Comparing the Effects of Hot Shower and Intravenous Injection of Hyoscine on the Pain Intensity and Duration of Active Phase of Labour in Nulliparous Women. *Journal of Clinical and Diagnostic Research* 2018, 12(8).

326. Madden K, Middleton P, Cyna AM, Matthewson M, Jones L: Hypnosis for pain management during labour and childbirth. In., vol. 2016; 2016.

327. Magwood O, Kpade V, Thavorn K, Oliver S, Mayhew AD, Pottie K: Effectiveness of home-based records on maternal, newborn and child health outcomes: A systematic review and meta-analysis. *PLoS One* 2019, 14(1):e0209278.

328. Mahnaz E, Nasim B, Sonia O: Effect of a structured educational package on women's sexual function during pregnancy. *Int J Gynaecol Obstet* 2020, 148(2):225-230.

329. Malekuti J, Mirghafourvand M, Samadi K, Abbasalizadeh F, Khodaei L: Comparison of the effect of Myrtus communis herbal and anti-hemorrhoid ointments on the hemorrhoid symptoms and quality of life in postpartum women with grade I and II internal hemorrhoid: A triple-blinded randomized controlled clinical trial. *J Complement Integr Med* 2019, 16(4).

330. Maman S, Moodley D, McNaughton-Reyes HL, Groves AK, Kagee A, Moodley P: Efficacy of enhanced HIV counseling for risk reduction during pregnancy and in the postpartum period: a randomized controlled trial. *PLoS One* 2014, 9(5):e97092.

For peer review only - http://bmjopen.bmj.com/site/about/guidelines.xhtml

331. Mangesi L, Hofmeyr GJ, Smith V, Smyth RMD: Fetal movement counting for assessment of fetal wellbeing. In., vol. 2015; 2015.
332. Mao HJ, Li HJ, Chiu H, Chan WC, Chen SL: Effectiveness of antenatal emotional self-management training program in prevention of postnatal depression in Chinese women. *Perspect Psychiatr Care* 2012, 48(4):218-224.
333. Marc I, Toureche N, Ernst E, Hodnett ED, Blanchet C, Dodin S, Njoya MM: Mind-body interventions during pregnancy for preventing or treating women's anxiety. In., vol. 2011; 2011.
334. Marchant T, Schellenberg JA, Edgar T, Nathan R, Abdulla S, Mukasa O, Mponda H, Lengeler C: Socially marketed insecticide-treated nets improve malaria and anaemia in pregnancy in southern Tanzania. *Trop Med Int Health* 2002, 7(2):149-158.
335. Martin J, MacDonald-Wicks L, Hure A, Smith R, Collins CE: Reducing postpartum weight retention and improving breastfeeding outcomes in overweight women: a pilot randomised controlled trial. *Nutrients* 2015, 7(3):1464-1479.
336. Martis R, Crowther CA, Shepherd E, Alsweiler J, Downie MR, Brown J: Treatments for women with gestational diabetes mellitus: An overview of Cochrane systematic reviews. In., vol. 2018; 2018.
337. Marufu TC, Ahankari A, Coleman T, Lewis S: Maternal smoking and the risk of still birth: systematic review and meta-analysis. *BMC Public Health* 2015, 15(1):239.
338. Mary M, Jafarey S, Dabash R, Kamal I, Rabbani A, Abbas D, Durocher J, Tan YL, Winikoff B: The Safety and Feasibility of a Family First Aid Approach for the Management of Postpartum Hemorrhage in Home Births: A Pre-post Intervention Study in Rural Pakistan. *Matern Child Health J* 2021, 25(1):118-126.
339. Masoumi SZ, Kazemi F, Oshvandi K, Jalali M, Esmaeili-Vardanjani A, Rafiei H: Effect of Training Preparation for Childbirth on Fear of Normal Vaginal Delivery and Choosing the Type of Delivery Among Pregnant Women in Hamadan, Iran: A Randomized Controlled Trial. *Journal of family & reproductive health* 2016, 10(3).
340. Masters SH, Agot K, Obonyo B, Napierala Mavedzenge S, Maman S, Thirumurthy H: Promoting Partner Testing and Couples Testing through Secondary Distribution of HIV Self-Tests: A Randomized Clinical Trial. *PLoS Med* 2016, 13(11):e1002166.
341. Masulli M, Vitacolonna E, Fraticelli F, Della Pepa G, Mannucci E, Monami M: Effects of probiotic supplementation during pregnancy on metabolic outcomes: A systematic review and meta-analysis of randomized controlled trials. In., vol. 162; 2020.
342. Masuzawa Y, Kataoka Y, Nakamura S, Yaju Y: Cooling the lower abdomen to reduce postpartum blood loss: A randomized controlled trial. *PLoS One* 2017, 12(10):e0186365.
343. Matei A, Saccone G, Vogel JP, Armson AB: Primary and secondary prevention of preterm birth: a review of systematic reviews and ongoing randomized controlled trials. In., vol. 236; 2019.
344. Matenchuk B, Khurana R, Cai C, Boule NG, Slater L, Davenport MH: Prenatal bed rest in developed and developing regions: a systematic review and meta-analysis. *CMAJ Open* 2019, 7(3):E435-E445.
345. Matthews LT, Jaggernath M, Kriel Y, Smith PM, O'Neil K, Haberer JE, Hendrix C, Baeten JM, Ware NC, Wirth K *et al*: Protocol for a longitudinal study to evaluate the use of tenofovir-based PrEP for safer conception and pregnancy among women in South Africa. *BMJ Open* 2019, 9(7):e027227.
346. Matvienko-Sikar K, Lee L, Murphy G, Murphy L: The effects of mindfulness interventions on prenatal well-being: A systematic review. *Psychol Health* 2016, 31(12):1415-1434.
347. McBride CM, Baucom DH, Peterson BL, Pollak KI, Palmer C, Westman E, Lyna P: Prenatal and postpartum smoking abstinence a partner-assisted approach. *Am J Prev Med* 2004, 27(3):232-238.
348. McBride CM, Curry SJ, Lando HA, Pirie PL, Grothaus LC, Nelson JC: Prevention of relapse in women who quit smoking during pregnancy. *Am J Public Health* 1999, 89(5):706-711.
349. McCarthy EA, Walker SP, Ugoni A, Lappas M, Leong O, Shub A: Self-weighing and simple dietary advice for overweight and obese pregnant women to reduce obstetric complications without impact on quality of life: A randomised controlled trial. In., vol. 123; 2016.

For peer review only - http://bmjopen.bmj.com/site/about/guidelines.xhtml

350. McCloskey RJ, Reno R: Complementary health approaches for postpartum depression: A systematic review. In., vol. 17; 2019.

351. McGrath A, Sharpe L, Lah S, Parratt K: Evaluation of a Decision Aid for Women with Epilepsy Who Are Considering Pregnancy: A Randomized Controlled Trial. *Med Decis Making* 2017, 37(5):589-599.

352. McLeod D, Pullon S, Benn C, Cookson T, Dowell A, Viccars A, White S, Green R, Crooke M: Can support and education for smoking cessation and reduction be provided effectively by midwives within primary maternity care? *Midwifery* 2004, 20(1):37-50.

353. Megregian M, Emeis C, Nieuwenhuijze M: The Impact of Shared Decision-Making in Perinatal Care: A Scoping Review. *J Midwifery Womens Health* 2020, 65(6):777-788.

354. Middleton P, Gomersall JC, Gould JF, Shepherd E, Olsen SF, Makrides M: Omega-3 fatty acid addition during pregnancy. In., vol. 2018; 2018.

355. Midhet F, Becker S: Impact of community-based interventions on maternal and neonatal health indicators: Results from a community randomized trial in rural Balochistan, Pakistan. *Reprod Health* 2010, 7(1):30.

356. Milgrom J, Holt C, Holt CJ, Ross J, Ericksen J, Gemmill AW: Feasibility study and pilot randomised trial of an antenatal depression treatment with infant follow-up. *Arch Womens Ment Health* 2015, 18(5):717-730.

357. Ming WK, Ding W, Zhang CJP, Zhong L, Long Y, Li Z, Sun C, Wu Y, Chen H, Chen H *et al*: The effect of exercise during pregnancy on gestational diabetes mellitus in normal-weight women: a systematic review and meta-analysis. *BMC Pregnancy Childbirth* 2018, 18(1):440.

358. Minschart C, Maes T, De Block C, Van Pottelbergh I, Myngheer N, Abrams P, Vinck W, Leuridan L, Mathieu C, Billen J *et al*: Mobile-Based Lifestyle Intervention in Women with Glucose Intolerance after Gestational Diabetes Mellitus (MELINDA), A Multicenter Randomized Controlled Trial: Methodology and Design. *J Clin Med* 2020, 9(8).

359. Miquelutti MA, Cecatti JG, Makuch MY: Upright position during the first stage of labor: a randomised controlled trial. *Acta Obstet Gynecol Scand* 2007, 86(5):553-558.

360. Miquelutti MA, Cecatti JG, Makuch MY: Evaluation of a birth preparation program on lumbopelvic pain, urinary incontinence, anxiety and exercise: a randomized controlled trial. *BMC Pregnancy Childbirth* 2013, 13:154.

361. Miquelutti MA, Silveira C, Cecatti JG: Kinesiologic tape for labor pain control: Randomized controlled trial. *Physiother Theory Pract* 2019, 35(7):614-621.

362. Mir AM, Wajid A, Gull S: Helping rural women in Pakistan to prevent postpartum hemorrhage: a quasi experimental study. *BMC Pregnancy Childbirth* 2012, 12:120.

363. Mobarakabadi SS, Shahbazzadegan S, Ozgoli G: The effect of P6 acupressure on nausea and vomiting of pregnancy: A randomized, single-blind, placebo-controlled trial. *Advances in Integrative Medicine* 2020, 7(2):67-72.

364. Mofenson LM, Baggaley RC, Mameletzis I: Tenofovir disoproxil fumarate safety for women and their infants during pregnancy and breastfeeding. *AIDS* 2017, 31(2):213-232.

365. Mohammadi F, Malakooti J, Babapoor J, Mohammad-Alizadeh-Charandabi S: The effect of a home-based exercise intervention on postnatal depression and fatigue: A randomized controlled trial. *Int J Nurs Pract* 2015, 21(5):478-485.

366. Monim Alzurfi N, Shadhan Al-Ogaili S: Effectiveness of Breathing as a Non-Pharmacological Method to Reduce Pain Severity among Woman During Labor. *Medico Legal Update* 2021, 21(1):674-677.

367. Moore L, Campbell R, Whelan A, Mills N, Lupton P, Misselbrook E, Frohlich J: Self help smoking cessation in pregnancy: cluster randomised controlled trial. *BMJ* 2002, 325(7377):1383.

368. Moradi M, Niazi A, Heydarian Miri H, Lopez V: The effect of evening primrose oil on labor induction and cervical ripening: A systematic review and meta-analysis. In., vol. 35; 2021.

369. Morison PN, Bacardi-Gascon M, Lopez-Corrales M, Jimenez-Cruz A: Combined dietary-exercise intervention for gestational weight gain and birthweight: a meta-analysis. *Asia Pac J Clin Nutr* 2018, 27(4):860-868.

For peer review only - http://bmjopen.bmj.com/site/about/guidelines.xhtml

370. Motahari-Tabari NS, Nasiri-Amiri F, Faramarzi M, Shirvani MA, Bakhtiari A, Omidvar S: The Effectiveness of Information-Motivation-Behavioral Skills Model on Self-Care Practices in Early Pregnancy to Prevent Gestational Diabetes Mellitus in Iranian Overweight and Obese Women: A Randomized Controlled Trial. *Int Q Community Health Educ* 2021:272684X211020300.

371. Motlagh AE, Babazadeh R, Akhlaghi F, Esmaily H: Effect of an educational intervention program based on bandura's self-efficacy theory on self-care, self-efficacy, and blood sugar levels in mothers with pre-diabetes during pregnancy. In., vol. 9; 2019.

372. Munkhondya BMJ, Munkhondya TE, Chirwa E, Wang H: Efficacy of companion-integrated childbirth preparation for childbirth fear, self-efficacy, and maternal support in primigravid women in Malawi. *BMC Pregnancy Childbirth* 2020, 20(1):48.

373. Munro S, Stacey D, Lewis KB, Bansback N: Choosing treatment and screening options congruent with values: Do decision aids help? Sub-analysis of a systematic review. In., vol. 99; 2016.

374. Murphy HR, Rayman G, Lewis K, Kelly S, Johal B, Duffield K, Fowler D, Campbell PJ, Temple RC: Effectiveness of continuous glucose monitoring in pregnant women with diabetes: randomised clinical trial. *BMJ* 2008, 337(7675):a1680.

375. N.Y VDW, C H, W VD: Preventing prenatal alcohol use via health counseling by midwives and Internet-based computer tailored feedback: A randomized controlled trial. In., vol. 19; 2012.

376. Napolitano MA, Harrington CB, Patchen L, Ellis LP, Ma T, Chang K, Gaminian A, Bailey CP, Evans WD: Feasibility of a Digital Intervention to Promote Healthy Weight Management among Postpartum African American/Black Women. *Int J Environ Res Public Health* 2021, 18(4).

377. Nassar N, Roberts CL, Raynes-Greenow CH, Barratt A, Peat B, Decision Aid for Breech Presentation Trial C: Evaluation of a decision aid for women with breech presentation at term: a randomised controlled trial [ISRCTN14570598]. *BJOG* 2007, 114(3):325-333.

378. Naughton F, Cooper S, Foster K, Emery J, Leonardi-Bee J, Sutton S, Jones M, Ussher M, Whitemore R, Leighton M *et al*: Large multi-centre pilot randomized controlled trial testing a low-cost, tailored, self-help smoking cessation text message intervention for pregnant smokers (MiQuit). *Addiction* 2017, 112(7):1238-1249.

379. Naughton F, Prevost AT, Gilbert H, Sutton S: Randomized controlled trial evaluation of a tailored leaflet and SMS text message self-help intervention for pregnant smokers (MiQuit). *Nicotine Tob Res* 2012, 14(5):569-577.

380. Naughton F, Prevost AT, Sutton S: Self-help smoking cessation interventions in pregnancy: A systematic review and meta-analysis. In., vol. 103; 2008.

381. Nazari M, Basiri F, Kaveh MH, Ghaem H: Investigating the Effect of Fordyce Happiness Cognitive-Behavioral Training on Perceived Stress and Happiness in Pregnant Females: Applying the Self-Efficacy Theory. *Iranian Red Crescent Medical Journal* 2018, In Press(In Press).

382. Ndyomugyenyi R, Clarke SE, Hutchison CL, Hansen KS, Magnussen P: Efficacy of malaria prevention during pregnancy in an area of low and unstable transmission: an individually-randomised placebo-controlled trial using intermittent preventive treatment and insecticide-treated nets in the Kabale Highlands, southwestern Uganda. *Trans R Soc Trop Med Hyg* 2011, 105(11):607-616.

383. Nelson DB, Bellamy S, Gray TS, Nachamkin I: Self-collected versus provider-collected vaginal swabs for the diagnosis of bacterial vaginosis: an assessment of validity and reliability. *J Clin Epidemiol* 2003, 56(9):862-866.

384. Neri I, Dante G, Pignatti L, Salvioli C, Facchinetti F: Castor oil for induction of labour: a retrospective study. *J Matern Fetal Neonatal Med* 2018, 31(16):2105-2108.

385. Ngichabe SK, Gatinu BW, Nyangore MA, Karuga R, Wanyonyi SZ, Kiarie JN: Reminder Systems for Self Uterine Massage in the Prevention of Postpartum Blood Loss. *East Afr Med J* 2012, 89(4):128-133.

386. Nicholson WK, Beckham AJ, Hatley K, Diamond M, Johnson LS, Green SL, Tate D: The Gestational Diabetes Management System (GooDMomS): development, feasibility and lessons learned from a patient-informed, web-based pregnancy and postpartum lifestyle intervention. *BMC Pregnancy Childbirth* 2016, 16(1):277.

For peer review only - http://bmjopen.bmj.com/site/about/guidelines.xhtml

387. Nielsen JN, Gittelsohn J, Anliker J, O'Brien K: Interventions to improve diet and weight gain among pregnant adolescents and recommendations for future research. *J Am Diet Assoc* 2006, 106(11):1825-1840.

388. Nieminen K, Andersson G, Wijma B, Ryding EL, Wijma K: Treatment of nulliparous women with severe fear of childbirth via the Internet: a feasibility study. *J Psychosom Obstet Gynaecol* 2016, 37(2):37-43.

389. Nilsson C, Lundgren I, Smith V, Vehvilainen-Julkunen K, Nicoletti J, Devane D, Bernloehr A, van Limbeek E, Lalor J, Begley C: Women-centred interventions to increase vaginal birth after caesarean section (VBAC): A systematic review. *Midwifery* 2015, 31(7):657-663.

390. Njagi JK, Magnussen P, Estambale B, Ouma J, Mugo B: Prevention of anaemia in pregnancy using insecticide-treated bednets and sulfadoxine-pyrimethamine in a highly malarious area of Kenya: a randomized controlled trial. *Transactions of the Royal Society of Tropical Medicine and Hygiene* 2003, 97(3):277-282.

391. Nwebube C, Glover V, Stewart L: Prenatal listening to songs composed for pregnancy and symptoms of anxiety and depression: a pilot study. *BMC Complement Altern Med* 2017, 17(1):256.

392. O'Kelly SM, Moore ZE: Antenatal maternal education for improving postnatal perineal healing for women who have birthed in a hospital setting. In., vol. 2017; 2017.

393. Oakeshott P, Hay P, Hay S, Steinke F, Rink E, Thomas B, Oakeley P, Kerry S: Detection of Chlamydia trachomatis infection in early pregnancy using self-administered vaginal swabs and first pass urines: A cross-sectional community-based survey. *British Journal of General Practice* 2002, 52(483).

394. Offianan AT, Penali LK, Coulibaly M, Tiacoh N, Ako A, Adji E, Coulibaly B, Koffi D, Sarr D, Jambou R *et al*: Comparative efficacy of uncontrolled and controlled intermittent preventive treatment during pregnancy (IPTp) with combined use of LLTNs in high resistance area to sulfadoxine-pyrimethamine in Cote d'Ivoire. *Infect Drug Resist* 2012, 5(1):53-63.

395. Oken E, Ning Y, Rifas-Shiman SL, Radesky JS, Rich-Edwards JW, Gillman MW: Associations of physical activity and inactivity before and during pregnancy with glucose tolerance. *Obstet Gynecol* 2006, 108(5):1200-1207.

396. Okesene-Gafa KAM, Li M, McKinlay CJD, Taylor RS, Rush EC, Wall CR, Wilson J, Murphy R, Taylor R, Thompson JMD *et al*: Effect of antenatal dietary interventions in maternal obesity on pregnancy weight-gain and birthweight: Healthy Mums and Babies (HUMBA) randomized trial. *Am J Obstet Gynecol* 2019, 221(2):152 e151-152 e113.

397. Oladapo OT, Blum J, Abalos E, Okusanya BO: Advance misoprostol distribution to pregnant women for preventing and treating postpartum haemorrhage. In., vol. 2020; 2020.

398. Oliveira FS, Vieira F, Guimaraes JV, Aredes ND, Campbell SH: Lanolin and prenatal health education for prevention of nipple pain and trauma: Randomized clinical trial. *Enferm Clin (Engl Ed)* 2021, 31(2):82-90.

399. Olson CM, Groth SW, Graham ML, Reschke JE, Strawderman MS, Fernandez ID: The effectiveness of an online intervention in preventing excessive gestational weight gain: the e-moms roc randomized controlled trial. *BMC Pregnancy Childbirth* 2018, 18(1):148.

400. Olson CM, Strawderman MS, Graham ML: Use of an Online Diet Goal-Setting Tool: Relationships With Gestational Weight Gain. *J Nutr Educ Behav* 2019, 51(4):391-399.

401. Ong MJ, Guelfi KJ, Hunter T, Wallman KE, Fournier PA, Newnham JP: Supervised home-based exercise may attenuate the decline of glucose tolerance in obese pregnant women. *Diabetes Metab* 2009, 35(5):418-421.

402. Ononge S, Campbell OM, Kaharuza F, Lewis JJ, Fielding K, Mirembe F: Effectiveness and safety of misoprostol distributed to antenatal women to prevent postpartum haemorrhage after child-births: a stepped-wedge cluster-randomized trial. *BMC Pregnancy Childbirth* 2015, 15:315.

403. Oostdam N, Van Poppel MNM, Wouters MGAJ, Van Mechelen W: Interventions for preventing gestational diabetes mellitus: A systematic review and meta-analysis. In., vol. 20; 2011.

404. Osaki K, Hattori T, Kosen S: The role of home-based records in the establishment of a continuum of care for mothers, newborns, and children in Indonesia. *Glob Health Action* 2013, 6(1):1-12.

For peer review only - http://bmjopen.bmj.com/site/about/guidelines.xhtml

405. Osório S, Júnior L, Nicolau A: Assessment of the effectiveness of non-pharmacological methods in pain relief during labor. *Revista da Rede de Enfermagem do Nordeste* 2014, 15:174-184.

406. Osterman RL, Carle AC, Ammerman RT, Gates D: Single-session motivational intervention to decrease alcohol use during pregnancy. *J Subst Abuse Treat* 2014, 47(1):10-19.

407. Owais S, Chow CHT, Furtado M, Frey BN, Van Lieshout RJ: Non-pharmacological interventions for improving postpartum maternal sleep: A systematic review and meta-analysis. In., vol. 41; 2018.

408. Palacios C, Kostiuk LK, Pena-Rosas JP: Vitamin D supplementation for women during pregnancy. *Cochrane Database Syst Rev* 2019, 7:CD008873.

409. Palnati M, Marcus BH, Pekow P, Rosal MC, Manson JE, Chasan-Taber L: The Impact of a Lifestyle Intervention on Postpartum Weight Retention Among At-Risk Hispanic Women. *Am J Prev Med* 2021, 61(1):44-54.

410. Pan WL, Gau ML, Lee TY, Jou HJ, Liu CY, Wen TK: Mindfulness-based programme on the psychological health of pregnant women. *Women Birth* 2019, 32(1):e102-e109.

411. Parikh R, Rao SR, Kukde R, O'Connor GT, Patel A, Hibberd PL: Assessing the Respiratory Effects of Air Pollution from Biomass Cookstoves on Pregnant Women in Rural India. *Int J Environ Res Public Health* 2020, 18(1).

412. Park SJ, Lee J: The effects of health care programs for gestational diabetes mellitus in South Korea: a systematic review. *Korean Journal of Women Health Nursing* 2020, 26(4):274-284.

413. Passerini L, Casey GJ, Biggs BA, Cong DT, Phu LB, Phuc TQ, Carone M, Montresor A: Increased birth weight associated with regular pre-pregnancy deworming and weekly iron-folic acid supplementation for Vietnamese women. *PLoS Negl Trop Dis* 2012, 6(4):e1608.

414. Patel A, Goudar SS, Geller SE, Kodkany BS, Edlavitch SA, Wagh K, Patted SS, Naik VA, Moss N, Derman RJ: Drape estimation vs. visual assessment for estimating postpartum hemorrhage. *Int J Gynaecol Obstet* 2006, 93(3):220-224.

415. Peaceman AM, Clifton RG, Phelan S, Gallagher D, Evans M, Redman LM, Knowler WC, Joshipura K, Haire-Joshu D, Yanovski SZ *et al*: Lifestyle Interventions Limit Gestational Weight Gain in Women with Overweight or Obesity: LIFE-Moms Prospective Meta-Analysis. *Obesity (Silver Spring)* 2018, 26(9):1396-1404.

416. Pena-Rosas JP, De-Regil LM, Garcia-Casal MN, Dowswell T: Daily oral iron supplementation during pregnancy. *Cochrane Database Syst Rev* 2015(7):CD004736.

417. Peña-Rosas JP, De-Regil LM, Malave HG, Flores-Urrutia MC, Dowswell T: Intermittent oral iron supplementation during pregnancy. In., vol. 2015; 2015.

418. Pereira Gomes Morais E, Riera R, Porfírio GJM, Macedo CR, Sarmento Vasconcelos V, de Souza Pedrosa A, Torloni MR: Chewing gum for enhancing early recovery of bowel function after caesarean section. In., vol. 2016; 2016.

419. Phelan S, Hagobian T, Brannen A, Stewart A, Schmid B, Cedars L: Promoting Weight Loss Before Pregnancy: Feasible or Futile? *Californian Journal of Health Promotion* 2013, 11(2).

420. Phelan S, Phipps MG, Abrams B, Darroch F, Grantham K, Schaffner A, Wing RR: Does behavioral intervention in pregnancy reduce postpartum weight retention? Twelve-month outcomes of the Fit for Delivery randomized trial. *Am J Clin Nutr* 2014, 99(2):302-311.

421. Phipps MG, Raker CA, Ware CF, Zlotnick C: Randomized controlled trial to prevent postpartum depression in adolescent mothers. *Am J Obstet Gynecol* 2013, 208(3):192 e191-196.

422. Pilarczyk R, Strozik M, Hirnle L: Diagnostic equivalency of mobile CTG devices and remote analysis to conventional on-site nonstress test. *Adv Clin Exp Med* 2020, 29(1):33-44.

423. Pineles BL, Hsu S, Park E, Samet JM: Systematic review and meta-analyses of perinatal death and maternal exposure to tobacco smoke during pregnancy. In., vol. 184; 2016.

424. Polsky S, Garcetti R, Pyle L, Joshee P, Demmitt JK, Snell-Bergeon JK: Continuous glucose monitor use with and without remote monitoring in pregnant women with type 1 diabetes: A pilot study. *PLoS One* 2020, 15(4):e0230476.

For peer review only - http://bmjopen.bmj.com/site/about/guidelines.xhtml

425. Ragnar I, Altman D, Tyden T, Olsson SE: Comparison of the maternal experience and duration of labour in two upright delivery positions--a randomised controlled trial. *BJOG* 2006, 113(2):165-170.

426. Rajbhandari S, Hodgins S, Sanghvi H, McPherson R, Pradhan YV, Baqui AH, Misoprostol Study G: Expanding uterotonic protection following childbirth through community-based distribution of misoprostol: operations research study in Nepal. *Int J Gynaecol Obstet* 2010, 108(3):282-288.

427. Ramakrishnan U, Grant FK, Goldenberg T, Bui V, Imdad A, Bhutta ZA: Effect of multiple micronutrient supplementation on pregnancy and infant outcomes: a systematic review. *Paediatr Perinat Epidemiol* 2012, 26 Suppl 1(SUPPL. 1):153-167.

428. Raman P, Shepherd E, Dowswell T, Middleton P, Crowther CA: Different methods and settings for glucose monitoring for gestational diabetes during pregnancy. In., vol. 2017; 2017.

429. Ramanathan K, Sirala Jagadeesh N, Vishwanath U, Dayal C, Chandrababu R, Hayter M: Efficacy of supplementation of probiotics on maternal glycaemic control – A systematic review and meta-analysis of randomized controlled trials. In., vol. 10; 2021.

430. Rashtchi V, Maryami N, Molaei B: Comparison of entonox and transcutaneous electrical nerve stimulation (TENS) in labor pain: a randomized clinical trial study. *J Matern Fetal Neonatal Med* 2022, 35(16):3124-3128.

431. Rasmussen L, Christensen ML, Poulsen CW, Rud C, Christensen AS, Andersen JR, Kampmann U, Ovesen PG: Effect of High Versus Low Carbohydrate Intake in the Morning on Glycemic Variability and Glycemic Control Measured by Continuous Blood Glucose Monitoring in Women with Gestational Diabetes Mellitus-A Randomized Crossover Study. *Nutrients* 2020, 12(2).

432. Rasouli M, Pourheidari M, Hamzeh Gardesh Z: Effect of Self-care Before and During Pregnancy to Prevention and Control Preeclampsia in High-risk Women. *Int J Prev Med* 2019, 10:21.

433. Redman LM, Drews KL, Klein S, Horn LV, Wing RR, Pi-Sunyer X, Evans M, Joshipura K, Arteaga SS, Cahill AG *et al*: Attenuated early pregnancy weight gain by prenatal lifestyle interventions does not prevent gestational diabetes in the LIFE-Moms consortium. *Diabetes Res Clin Pract* 2021, 171:108549.

434. Revello MG, Tibaldi C, Masuelli G, Frisina V, Sacchi A, Furione M, Arossa A, Spinillo A, Klersy C, Ceccarelli M *et al*: Prevention of Primary Cytomegalovirus Infection in Pregnancy. *EBioMedicine* 2015, 2(9):1205-1210.

435. Rezaeean SM, Abedian Z, Latifnejad-Roudsari R, Mazloum SR, Abbasi Z: The Effect of Prenatal Self-Care Based on Orem's Theory on Preterm Birth Occurrence in Women at Risk for Preterm Birth. *Iran J Nurs Midwifery Res* 2020, 25(3):242-248.

436. Rigla M, Martinez-Sarriegui I, Garcia-Saez G, Pons B, Hernando ME: Gestational Diabetes Management Using Smart Mobile Telemedicine. *J Diabetes Sci Technol* 2018, 12(2):260-264.

437. Robertson N, Okano S, Kumar S: Sleep in the Supine Position during Pregnancy Is Associated with Fetal Cerebral Redistribution. *J Clin Med* 2020, 9(6).

438. Rodriguez-Blanque R, Sanchez-Garcia JC, Sanchez-Lopez AM, Mur-Villar N, Aguilar-Cordero MJ: The influence of physical activity in water on sleep quality in pregnant women: A randomised trial. *Women Birth* 2018, 31(1):e51-e58.

439. Rondung E, Ternstrom E, Hildingsson I, Haines HM, Sundin O, Ekdahl J, Karlstrom A, Larsson B, Segeblad B, Baylis R *et al*: Comparing Internet-Based Cognitive Behavioral Therapy With Standard Care for Women With Fear of Birth: Randomized Controlled Trial. *JMIR Ment Health* 2018, 5(3):e10420.

440. Rong L, Wang R, Ouyang YQ, Redding SR: Efficacy of yoga on physiological and psychological discomforts and delivery outcomes in Chinese primiparas. *Complement Ther Clin Pract* 2021, 44:101434.

441. Rotheram-Borus MJ, Tomlinson M, le Roux IM, Harwood JM, Comulada S, O'Connor MJ, Weiss RE, Worthman CM: A cluster randomised controlled effectiveness trial evaluating perinatal home visiting among South African mothers/infants. *PLoS One* 2014, 9(10):e105934.

442. Rumbold A, Ota E, Hori H, Miyazaki C, Crowther CA: Vitamin E supplementation in pregnancy. In., vol. 2016; 2015.

For peer review only - http://bmjopen.bmj.com/site/about/guidelines.xhtml

443. Rungsiprakarn P, Laopaiboon M, Sangkomkamhang US, Lumbiganon P, Pratt JJ: Interventions for treating constipation in pregnancy. In., vol. 2015; 2015.

444. Saastad E, Israel P, Ahlborg T, Gunnes N, Froen JF: Fetal movement counting--effects on maternal-fetal attachment: a multicenter randomized controlled trial. *Birth* 2011, 38(4):282-293.

445. Saccone G, Berghella V, Venturella R, D'Alessandro P, Arduino B, Raffone A, Giudicepietro A, Visentin S, Vitagliano A, Martinelli P *et al*: Effects of exercise during pregnancy in women with short cervix: Secondary analysis from the Italian Pessary Trial in singletons. *Eur J Obstet Gynecol Reprod Biol* 2018, 229:132-136.

446. Saccone G, Caissutti C, Ciardulli A, Berghella V: Uterine massage for preventing postpartum hemorrhage at cesarean delivery: Which evidence? In., vol. 223; 2018.

447. Sagedal LR, Overby NC, Bere E, Torstveit MK, Lohne-Seiler H, Smastuen M, Hillesund ER, Henriksen T, Vistad I: Lifestyle intervention to limit gestational weight gain: the Norwegian Fit for Delivery randomised controlled trial. *BJOG* 2017, 124(1):97-109.

448. Sajitha AT, Philip AT, Sarika TK: Effectiveness of Prenatal Education Regarding Practice on Antenatal Exercises and Minor Ailments among Pregnant Mothers. *Journal of Clinical and Diagnostic Research* 2018, 12(9).

449. Salam RA, Zuberi NF, Bhutta ZA: Pyridoxine (vitamin B6) supplementation during pregnancy or labour for maternal and neonatal outcomes. In., vol. 2016; 2015.

450. Samawi L, Williams PP, Myers B, Fuhr DC: Effectiveness of psychological interventions to reduce alcohol consumption among pregnant and postpartum women: a systematic review. In., vol. 24; 2021.

451. Sampselle CM, Miller JM, Mims BL, Delancey JO, Ashton-Miller JA, Antonakos CL: Effect of pelvic muscle exercise on transient incontinence during pregnancy and after birth. *Obstet Gynecol* 1998, 91(3):406-412.

452. Sandborg J, Soderstrom E, Henriksson P, Bendtsen M, Henstrom M, Leppanen MH, Maddison R, Migueles JH, Blomberg M, Lof M: Effectiveness of a Smartphone App to Promote Healthy Weight Gain, Diet, and Physical Activity During Pregnancy (HealthyMoms): Randomized Controlled Trial. *JMIR Mhealth Uhealth* 2021, 9(3):e26091.

453. Sanghvi H, Ansari N, Prata NJ, Gibson H, Ehsan AT, Smith JM: Prevention of postpartum hemorrhage at home birth in Afghanistan. *Int J Gynaecol Obstet* 2010, 108(3):276-281.

454. Sapkota D, Baird K, Saito A, Anderson D: Interventions for reducing and/or controlling domestic violence among pregnant women in low- and middle-income countries: A systematic review. In., vol. 8; 2019.

455. Sapkota D, Baird K, Saito A, Rijal P, Anderson D: Antenatal-Based Pilot Psychosocial Intervention to Enhance Mental Health of Pregnant Women Experiencing Domestic and Family Violence in Nepal. *J Interpers Violence* 2022, 37(5-6):NP3605-NP3627.

456. Sarkar A, Mburu G, Shivkumar PV, Sharma P, Campbell F, Behera J, Dargan R, Mishra SK, Mehra S: Feasibility of supervised self-testing using an oral fluid-based HIV rapid testing method: a cross-sectional, mixed method study among pregnant women in rural India. *J Int AIDS Soc* 2016, 19(1):20993.

457. Scott EM, Feig DS, Murphy HR, Law GR: Continuous glucose monitoring in pregnancy: Importance of analyzing temporal profiles to understand clinical outcomes. In*: 2020*.

458. Secher AL, Ringholm L, Andersen HU, Damm P, Mathiesen ER: The effect of real-time continuous glucose monitoring in pregnant women with diabetes: a randomized controlled trial. *Diabetes Care* 2013, 36(7):1877-1883.

459. Sehhatie F, Mirgafourvand M, Vosoughi Niri Z: The Effect of Non-Pharmaceutical Methods of Labor Pain Relief on Mothers' Postpartum Depression: A Randomized Controlled Trial. *International Journal of Women's Health and Reproduction Sciences* 2015, 3(1):48-55.

460. Seneviratne SN, Jiang Y, Derraik J, McCowan L, Parry GK, Biggs JB, Craigie S, Gusso S, Peres G, Rodrigues RO *et al*: Effects of antenatal exercise in overweight and obese pregnant women on maternal and perinatal outcomes: a randomised controlled trial. *BJOG* 2016, 123(4):588-597.

For peer review only

461. Senol DK, Aslan E: The Effects of Cold Application to the Perineum on Pain Relief After Vaginal Birth. *Asian Nurs Res (Korean Soc Nurs Sci)* 2017, 11(4):276-282.

462. Sercekus P, Baskale H: Effects of antenatal education on fear of childbirth, maternal self-efficacy and parental attachment. *Midwifery* 2016, 34:166-172.

463. Serra R, Peñailillo R, Monteiro LJ, Monckeberg M, Peña M, Moyano L, Brunner C, Vega G, Choolani M, Illanes SE: Supplementation of omega 3 during pregnancy and the risk of preterm birth: A systematic review and meta-analysis. In., vol. 13; 2021.

464. Sethi V, Bhanot A, Bhattacharjee S, Gope R, Sarangi D, Nath V, Nair N, Singh U, Daniel A, Parhi RN *et al*: Integrated multisectoral strategy to improve girls' and women's nutrition before conception, during pregnancy and after birth in India (Swabhimaan): protocol for a prospective, non-randomised controlled evaluation. *BMJ Open* 2019, 9(11):e031632.

465. Shahsavan F, Akbari N, Gharraee B, Abolghasemi J, Khedmat L: The effect of internet-based guided self-help cognitive-behavioral therapies on Iranian women's psychological symptoms and preferred method of childbirth. *Perspect Psychiatr Care* 2021, 57(1):138-147.

466. Sharps PW, Bullock LF, Campbell JC, Alhusen JL, Ghazarian SR, Bhandari SS, Schminkey DL: Domestic Violence Enhanced Perinatal Home Visits: The DOVE Randomized Clinical Trial. *J Womens Health (Larchmt)* 2016, 25(11):1129-1138.

467. Shedmake PV, Wakode SR: A Hospital-Based Randomized Controlled Trial-Comparing the Outcome of Normal Delivery Between Squatting and Lying Down Positions During Labour. *J Obstet Gynaecol India* 2021, 71(4):393-398.

468. Shirah BH, Shirah HA, Fallata AH, Alobidy SN, Hawsawi MMA: Hemorrhoids during pregnancy: Sitz bath vs. ano-rectal cream: A comparative prospective study of two conservative treatment protocols. *Women Birth* 2018, 31(4):e272-e277.

469. Shivalli S, Srivastava RK, Singh GP: Trials of Improved Practices (TIPs) to Enhance the Dietary and Iron-Folate Intake during Pregnancy- A Quasi Experimental Study among Rural Pregnant Women of Varanasi, India. *PLoS One* 2015, 10(9):e0137735.

470. Shorey S, Chee CYI, Ng ED, Lau Y, Dennis CL, Chan YH: Evaluation of a Technology-Based Peer-Support Intervention Program for Preventing Postnatal Depression (Part 1): Randomized Controlled Trial. *J Med Internet Res* 2019, 21(8):e12410.

471. Shorten A, Shorten B, Keogh J, West S, Morris J: Making choices for childbirth: a randomized controlled trial of a decision-aid for informed birth after cesarean. *Birth* 2005, 32(4):252-261.

472. Sibley L, Buffington ST, Haileyesus D: The American College of Nurse-Midwives' home-based lifesaving skills program: a review of the Ethiopia field test. *J Midwifery Womens Health* 2004, 49(4):320-328.

473. Siddika N, Balogun HA, Amegah AK, Jaakkola JJK: Prenatal ambient air pollution exposure and the risk of stillbirth: Systematic review and meta-analysis of the empirical evidence. In., vol. 73; 2016.

474. Sidiki NNA, Payne VK, Cedric Y, Nadia NAC: Effect of Impregnated Mosquito Bed Nets on the Prevalence of Malaria among Pregnant Women in Foumban Subdivision, West Region of Cameroon. *J Parasitol Res* 2020, 2020:7438317.

475. Silver RM, Hunter S, Reddy UM, Facco F, Gibbins KJ, Grobman WA, Mercer BM, Haas DM, Simhan HN, Parry S *et al*: Prospective Evaluation of Maternal Sleep Position Through 30 Weeks of Gestation and Adverse Pregnancy Outcomes. *Obstet Gynecol* 2019, 134(4):667-676.

476. Simpson SA, Coulman E, Gallagher D, Jewell K, Cohen D, Newcombe RG, Huang C, Robles-Zurita JA, Busse M, Owen-Jones E *et al*: Healthy eating and lifestyle in pregnancy (HELP): a cluster randomised trial to evaluate the effectiveness of a weight management intervention for pregnant women with obesity on weight at 12 months postpartum. *Int J Obes (Lond)* 2021, 45(8):1728-1739.

477. Skouteris H, Hartley-Clark L, McCabe M, Milgrom J, Kent B, Herring SJ, Gale J: Preventing excessive gestational weight gain: a systematic review of interventions. *Obes Rev* 2010, 11(11):757-768.

For peer review only - http://bmjopen.bmj.com/site/about/guidelines.xhtml

478. Skouteris H, Morris H, Nagle C, Nankervis A: Behavior modification techniques used to prevent gestational diabetes: A systematic review of the literature topical collection on obesity. In., vol. 14; 2014.
479. Skovbjerg S, Birk D, Bruggisser S, Wolf ALA, Fjorback L: Mindfulness-based stress reduction adapted to pregnant women with psychosocial vulnerabilities-a protocol for a randomized feasibility study in a Danish hospital-based outpatient setting. *Pilot Feasibility Stud* 2021, 7(1):118.
480. Slade P, West H, Thomson G, Lane S, Spiby H, Edwards RT, Charles JM, Garrett C, Flanagan B, Treadwell M *et al*: STRAWB2 (Stress and Wellbeing After Childbirth): a randomised controlled trial of targeted self-help materials to prevent post-traumatic stress disorder following childbirth. *BJOG* 2020, 127(7):886-896.
481. Smith CA, Collins CT, Crowther CA: Aromatherapy for pain management in labour. *Cochrane Database Syst Rev* 2011, 14(7):CD009215.
482. Smith CA, Collins CT, Levett KM, Armour M, Dahlen HG, Tan AL, Mesgarpour B: Acupuncture or acupressure for pain management during labour. In., vol. 2020; 2020.
483. Smith CA, Hill E, Denejkina A, Thornton C, Dahlen HG: The effectiveness and safety of complementary health approaches to managing postpartum pain: A systematic review and meta-analysis. In., vol. 11; 2022.
484. Smith CA, Levett KM, Collins CT, Armour M, Dahlen HG, Suganuma M: Relaxation techniques for pain management in labour. In., vol. 2018; 2018.
485. Smith CA, Levett KM, Collins CT, Dahlen HG, Ee CC, Suganuma M: Massage, reflexology and other manual methods for pain management in labour. In., vol. 2018; 2018.
486. Smith ER, Shankar AH, Wu LS, Aboud S, Adu-Afarwuah S, Ali H, Agustina R, Arifeen S, Ashorn P, Bhutta ZA *et al*: Modifiers of the effect of maternal multiple micronutrient supplementation on stillbirth, birth outcomes, and infant mortality: a meta-analysis of individual patient data from 17 randomised trials in low-income and middle-income countries. *Lancet Glob Health* 2017, 5(11):e1090-e1100.
487. Smith JM, Gubin R, Holston MM, Fullerton J, Prata N: Misoprostol for postpartum hemorrhage prevention at home birth: an integrative review of global implementation experience to date. *BMC Pregnancy Childbirth* 2013, 13:44.
488. Smith SA, Michel Y: A pilot study on the effects of aquatic exercises on discomforts of pregnancy. *J Obstet Gynecol Neonatal Nurs* 2006, 35(3):315-323.
489. Smyth RMD, Aflaifel N, Bamigboye AA: Interventions for varicose veins and leg oedema in pregnancy. In., vol. 2015; 2015.
490. Sobhgol SS, Priddis H, Smith CA, Dahlen HG: Evaluation of the effect of an antenatal pelvic floor muscle exercise programme on female sexual function during pregnancy and the first 3 months following birth: study protocol for a pragmatic randomised controlled trial. *Trials* 2019, 20(1):144.
491. Soltani H, Scott AM: Antenatal breast expression in women with diabetes: outcomes from a retrospective cohort study. *Int Breastfeed J* 2012, 7(1):18.
492. Srisopa P, Cong X, Russell B, Lucas R: The Role of Emotion Regulation in Pain Management Among Women From Labor to Three Months Postpartum: An Integrative Review. *Pain Manag Nurs* 2021, 22(6):783-790.
493. Stade BC, Bailey C, Dzendoletas D, Sgro M, Dowswell T, Bennett D: Psychological and/or educational interventions for reducing alcohol consumption in pregnant women and women planning pregnancy. In.; 2009.
494. Steen M, Briggs M, King D: Alleviating postnatal perineal trauma: To cool or not to cool? In., vol. 14; 2006.
495. Steen M, Cooper K, Marchant P, Griffiths-Jones M, Walker J: A randomised controlled trial to compare the effectiveness of ice-packs and Epifoam with cooling maternity gel pads at alleviating postnatal perineal trauma. *Midwifery* 2000, 16(1):48-55.
496. Steen M, Robinson M, Robertson S, Raine G: Pre and post survey findings from the Mind 'Building resilience programme for better mental health: Pregnant women and new mothers'. In., vol. 13; 2015.
497. Stith BJ, Buls SM, Keim SA, Thung SF, Klebanoff MA, Landon MB, Gabbe SG, Gandhi KK, Oza-Frank R: Moms in motion: weight loss intervention for postpartum mothers after gestational diabetes: a randomized controlled trial. *BMC Pregnancy Childbirth* 2021, 21(1):461.

For peer review only - http://bmjopen.bmj.com/site/about/guidelines.xhtml

498. Strauss RA, Eucker B, Savitz DA, Thorp JM, Jr.: Diagnosis of bacterial vaginosis from self-obtained vaginal swabs. *Infect Dis Obstet Gynecol* 2005, 13(1):31-35.

499. Sun Y, Li Y, Wang J, Chen Q, Bazzano AN, Cao F: Effectiveness of Smartphone-Based Mindfulness Training on Maternal Perinatal Depression: Randomized Controlled Trial. *J Med Internet Res* 2021, 23(1):e23410.

500. Sun YC, Hung YC, Chang Y, Kuo SC: Effects of a prenatal yoga programme on the discomforts of pregnancy and maternal childbirth self-efficacy in Taiwan. *Midwifery* 2010, 26(6):e31-36.

501. Sungkar A, Purwosunu Y, Aziz MF, Pratomo H, Sutrisna B, Sekizawa A: Influence of early self-diagnosis and treatment of bacterial vaginosis on preterm birth rate. *Int J Gynaecol Obstet* 2012, 117(3):264-267.

502. Suntarattiwong P, Mott JA, Mohanty S, Sinthuwattanawibool C, Srisantiroj N, Patamasingh Na Ayudhaya O, Klungthong C, Fernandez S, Kim L, Hunt D *et al*: Feasibility and Performance of Self-Collected Nasal Swabs for Detection of Influenza Virus, Respiratory Syncytial Virus, and Human Metapneumovirus. *J Infect Dis* 2021, 224(5):831-838.

503. Surkan PJ, Hamdani SU, Huma ZE, Nazir H, Atif N, Rowther AA, Chaudhri R, Zafar S, Mullany LC, Malik A *et al*: Cognitive-behavioral therapy-based intervention to treat symptoms of anxiety in pregnancy in a prenatal clinic using non-specialist providers in Pakistan: design of a randomised trial. *BMJ Open* 2020, 10(4):e037590.

504. Sushko K, Menezes HT, Strachan P, Butt M, Sherifali D: Self-management education among women with pre-existing diabetes in pregnancy: A scoping review. In., vol. 117; 2021.

505. Syngelaki A, Sequeira Campos M, Roberge S, Andrade W, Nicolaides KH: Diet and exercise for preeclampsia prevention in overweight and obese pregnant women: systematic review and meta-analysis. In., vol. 32; 2019.

506. Szkwara JM, Milne N, Rathbone E: A prospective quasi-experimental controlled study evaluating the use of dynamic elastomeric fabric orthoses to manage common postpartum ailments during postnatal care. *Womens Health (Lond)* 2020, 16:1745506520927196.

507. Szumilewicz A, Dornowski M, Piernicka M, Worska A, Kuchta A, Kortas J, Bludnicka M, Radziminski L, Jastrzebski Z: High-Low Impact Exercise Program Including Pelvic Floor Muscle Exercises Improves Pelvic Floor Muscle Function in Healthy Pregnant Women - A Randomized Control Trial. *Front Physiol* 2018, 9:1867.

508. Taavoni S, Sheikhan F, Abdolahian S, Ghavi F: Birth ball or heat therapy? A randomized controlled trial to compare the effectiveness of birth ball usage with sacrum-perineal heat therapy in labor pain management. *Complement Ther Clin Pract* 2016, 24:99-102.

509. Taheri Z, Mazaheri MA, Khorsandi M, Hassanzadeh A, Amiri M: Effect of educational intervention on self-efficacy for choosing delivery method among pregnant women in 2013. *International Journal of Preventive Medicine* 2014, 5(10).

510. Tan PC, Andi A, Azmi N, Noraihan MN: Effect of coitus at term on length of gestation, induction of labor, and mode of delivery. *Obstet Gynecol* 2006, 108(1):134-140.

511. Tan XYJ, Choong SYX, Cheng LJ, Lau Y: Relaxation interventions for improving sleep outcomes in perinatal women: A systematic review and meta-analysis of randomized controlled trials. In., vol. 103; 2021.

512. Tanvig MH, Jensen DM, Andersen MS, Ovesen PG, Jorgensen JS, Vinter CA: Vitamin D levels were significantly higher during and after lifestyle intervention in pregnancy: A randomized controlled trial. *Acta Obstet Gynecol Scand* 2020, 99(3):350-356.

513. Tatala SR, Ash D, Makola D, Latham M, Ndosi G, Grohn Y: Effect of micronutrient fortified beverage on nutritional anaemia during pregnancy. *East Afr Med J* 2002, 79(11):598-603.

514. ter Kuile FO, Terlouw DJ, Phillips-Howard PA, Hawley WA, Friedman JF, Kariuki SK, Shi YP, Kolczak MS, Lal AA, Vulule JM *et al*: Reduction of malaria during pregnancy by permethrin-treated bed nets in an area of intense perennial malaria transmission in western Kenya. *Am J Trop Med Hyg* 2003, 68(4 Suppl):50-60.

515. Thompson MG, Ferber JR, Odouli R, David D, Shifflett P, Meece JK, Naleway AL, Bozeman S, Spencer SM, Fry AM *et al*: Results of a pilot study using self-collected mid-turbinate nasal swabs for detection of influenza virus infection among pregnant women. *Influenza Other Respir Viruses* 2015, 9(3):155-160.

For peer review only - http://bmjopen.bmj.com/site/about/guidelines.xhtml

516. Timur Tashan S, Kafkasli A: The effect of bitter almond oil and massaging on striae gravidarum in primiparaous women. *J Clin Nurs* 2012, 21(11-12):1570-1576.

517. Tinius R, Edens K, Link K, Jones MS, Lyons S, Rebelle T, Pearson KJ, Maples J: Effect of Evidence-Based Materials and Access to Local Resources on Physical Activity Levels, Beliefs, and Motivation During Pregnancy in a Rural Setting. *J Phys Act Health* 2020, 17(10):947-957.

518. Tixier H, Boucard C, Ferdynus C, Douvier S, Sagot P: Interest of using an underbuttocks drape with collection pouch for early diagnosis of postpartum hemorrhage. *Arch Gynecol Obstet* 2011, 283(1):25-29.

519. Todd CS, Chowdhury Z, Mahmud Z, Islam N, Shabnam S, Parvin M, Bernholc A, Martinez A, Aktar B, Afsana K *et al*: Maternal nutrition intervention and maternal complications in 4 districts of Bangladesh: A nested cross-sectional study. *PLoS Med* 2019, 16(10):e1002927.

520. Tragea C, Chrousos GP, Alexopoulos EC, Darviri C: A randomized controlled trial of the effects of a stress management programme during pregnancy. *Complement Ther Med* 2014, 22(2):203-211.

521. Tran K, Padwal R, Khan N, Wright MD, Chan WS: Home blood pressure monitoring in the diagnosis and treatment of hypertension in pregnancy: a systematic review and meta-analysis. *CMAJ Open* 2021, 9(2):E642-E650.

522. Tsai YJ, Hsu YY, Hou TW, Chang CH: Effects of a Web-Based Antenatal Care System on Maternal Stress and Self-Efficacy During Pregnancy: A Study in Taiwan. *J Midwifery Womens Health* 2018, 63(2):205-213.

523. Tseng YF, Chen CH, Lee CS: Effects of listening to music on postpartum stress and anxiety levels. *J Clin Nurs* 2010, 19(7-8):1049-1055.

524. Tucker KL, Bowen L, Crawford C, Mallon P, Hinton L, Lee MM, Oke J, Taylor KS, Heneghan C, Bankhead C *et al*: The feasibility and acceptability of self-testing for proteinuria during pregnancy: A mixed methods approach. *Pregnancy Hypertens* 2018, 12:161-168.

525. Tucker KL, Taylor KS, Crawford C, Hodgkinson JA, Bankhead C, Carver T, Ewers E, Glogowska M, Greenfield SM, Ingram L *et al*: Blood pressure self-monitoring in pregnancy: examining feasibility in a prospective cohort study. *BMC Pregnancy Childbirth* 2017, 17(1):442.

526. Tumminia A, Milluzzo A, Festa C, Fresa R, Pintaudi B, Scavini M, Vitacolonna E, Napoli A, Sciacca L: Efficacy of flash glucose monitoring in pregnant women with poorly controlled pregestational diabetes (FlashMom): A randomized pilot study. *Nutr Metab Cardiovasc Dis* 2021, 31(6):1851-1859.

527. Uğurlu M, Yavan T, Karaşahin KE: The effect of an education and counseling program on maternal/neonatal outcomes in pregnant women at risk of preeclampsia. *Puerto Rico Health Sciences Journal* 2021, 40(3).

528. Vakilian K, Atarha M, Bekhradi R, Chaman R: Healing advantages of lavender essential oil during episiotomy recovery: a clinical trial. *Complement Ther Clin Pract* 2011, 17(1):50-53.

529. Vamos CA, Thompson EL, Avendano M, Daley EM, Quinonez RB, Boggess K: Oral health promotion interventions during pregnancy: A systematic review. In., vol. 43; 2015.

530. Van der Pligt P, Willcox J, Hesketh KD, Ball K, Wilkinson S, Crawford D, Campbell K: Systematic review of lifestyle interventions to limit postpartum weight retention: Implications for future opportunities to prevent maternal overweight and obesity following childbirth. In., vol. 14; 2013.

531. Van Der Windt M, Van Der Kleij RMJJ, Snoek KM, Dykgraaf RHM, Laven JSE, Schoenmakers S, Steegers-Theunissen RPM: Impact of a blended periconception lifestyle care approach combining face-to-face counseling with ehealth. *Reproductive Sciences* 2020, 27(1).

532. Van Horn L, Peaceman A, Kwasny M, Vincent E, Fought A, Josefson J, Spring B, Neff LM, Gernhofer N: Dietary Approaches to Stop Hypertension Diet and Activity to Limit Gestational Weight: Maternal Offspring Metabolics Family Intervention Trial, a Technology Enhanced Randomized Trial. *Am J Prev Med* 2018, 55(5):603-614.

533. Van Parys AS, Verhamme A, Temmerman M, Verstraelen H: Intimate partner violence and pregnancy: A systematic review of interventions. In., vol. 9; 2014.

For peer review only - http://bmjopen.bmj.com/site/about/guidelines.xhtml

534. van Poppel MN, Jelsma JGM, Simmons D, Devlieger R, Jans G, Galjaard S, Corcoy R, Adelantado JM, Dunne F, Harreiter J *et al*: Mediators of Lifestyle Behaviour Changes in Obese Pregnant Women. Secondary Analyses from the DALI Lifestyle Randomised Controlled Trial. *Nutrients* 2019, 11(2).

535. Varisoglu Y, Gungor Satilmis I: The Effects of Listening to Music on Breast Milk Production by Mothers of Premature Newborns in the Neonatal Intensive Care Unit: A Randomized Controlled Study. *Breastfeed Med* 2020, 15(7):465-470.

536. Vasegh Rahimparvar SF, Hamzehkhani M, Geranmayeh M, Rahimi R: Effect of educational software on self-efficacy of pregnant women to cope with labor: a randomized controlled trial. *Arch Gynecol Obstet* 2012, 286(1):63-70.

537. Vauloup-Fellous C, Picone O, Cordier AG, Parent-du-Chatelet I, Senat MV, Frydman R, Grangeot-Keros L: Does hygiene counseling have an impact on the rate of CMV primary infection during pregnancy? Results of a 3-year prospective study in a French hospital. *J Clin Virol* 2009, 46 Suppl 4(SUPPL. 4):S49-53.

538. Vazquez JC: Constipation, haemorrhoids, and heartburn in pregnancy. *BMJ Clin Evid* 2010, 2010.

539. Veisani Y, Jenabi E, Delpisheh A, Khazaei S: Effect of prenatal smoking cessation interventions on birth weight: meta-analysis. In., vol. 32; 2019.

540. Vesco KK, Karanja N, King JC, Gillman MW, Leo MC, Perrin N, McEvoy CT, Eckhardt CL, Smith KS, Stevens VJ: Efficacy of a group-based dietary intervention for limiting gestational weight gain among obese women: a randomized trial. *Obesity (Silver Spring)* 2014, 22(9):1989-1996.

541. Vodopivec-Jamsek V, de Jongh T, Gurol-Urganci I, Atun R, Car J: Mobile phone messaging for preventive health care. In., vol. 2017; 2012.

542. Vrana-Diaz CJ, Korte JE, Gebregziabher M, Richey L, Selassie A, Sweat M, Gichangi A: Relationship Gender Equality and Couples' Uptake of Oral Human Immunodeficiency Virus Self-Testing Kits Delivered by Pregnant Women in Kenya. *Sex Transm Dis* 2019, 46(9):588-593.

543. Wan CS, Nankervis A, Teede H, Aroni R: Dietary intervention strategies for ethnic Chinese women with gestational diabetes mellitus: A systematic review and meta-analysis. In., vol. 76; 2019.

544. Wang X, Xu X, Luo J, Chen Z, Feng S: Effect of app-based audio guidance pelvic floor muscle training on treatment of stress urinary incontinence in primiparas: A randomized controlled trial. *Int J Nurs Stud* 2020, 104:103527.

545. Wang Z, Liu Q, Min L, Mao X: The effectiveness of the laid-back position on lactation-related nipple problems and comfort: a meta-analysis. *BMC Pregnancy Childbirth* 2021, 21(1):248.

546. Wannakosit S, Phupong V: Sexual behavior in pregnancy: comparing between sexual education group and nonsexual education group. *J Sex Med* 2010, 7(10):3434-3438.

547. Warland J, Dorrian J, Morrison JL, O'Brien LM: Maternal sleep during pregnancy and poor fetal outcomes: A scoping review of the literature with meta-analysis. In., vol. 41; 2018.

548. Webber GC, Chirangi B: Women's health in women's hands: a pilot study assessing the feasibility of providing women with medications to reduce postpartum hemorrhage and sepsis in rural Tanzania. *Health Care Women Int* 2014, 35(7-9):758-770.

549. Weeks AD, Ditai J, Ononge S, Faragher B, Frye LJ, Durocher J, Mirembe FM, Byamugisha J, Winikoff B, Alfirevic Z: The MamaMiso study of self-administered misoprostol to prevent bleeding after childbirth in rural Uganda: a community-based, placebo-controlled randomised trial. *BMC Pregnancy Childbirth* 2015, 15:219.

550. Werner A, Uldbjerg N, Zachariae R, Rosen G, Nohr EA: Self-hypnosis for coping with labour pain: a randomised controlled trial. *BJOG* 2013, 120(3):346-353.

551. Whitford HM, Alder B, Jones M: A longitudinal follow up of women in their practice of perinatal pelvic floor exercises and stress urinary incontinence in North-East Scotland. *Midwifery* 2007, 23(3):298-308.

552. Willcox JC, Wilkinson SA, Lappas M, Ball K, Crawford D, McCarthy EA, Fjeldsoe B, Whittaker R, Maddison R, Campbell KJ: A mobile health intervention promoting healthy gestational weight gain for women entering

For peer review only - http://bmjopen.bmj.com/site/about/guidelines.xhtml

pregnancy at a high body mass index: the txt4two pilot randomised controlled trial. *BJOG* 2017, 124(11):1718-1728.

553. Windsor R, Woodby L, Miller T, Hardin M: Effectiveness of Smoking Cessation and Reduction in Pregnancy Treatment (SCRIPT) methods in Medicaid-supported prenatal care: Trial III. *Health Educ Behav* 2011, 38(4):412-422.

554. Windsor RA, Cutter G, Morris J, Reese Y, Manzella B, Bartlett EE, Samuelson C, Spanos D: The effectiveness of smoking cessation methods for smokers in public health maternity clinics: a randomized trial. *Am J Public Health* 1985, 75(12):1389-1392.

555. Wing DA, Rumney PJ, Preslicka CW, Chung JH: Daily cranberry juice for the prevention of asymptomatic bacteriuria in pregnancy: a randomized, controlled pilot study. *J Urol* 2008, 180(4):1367-1372.

556. Witt AM, Bolman M, Kredit S: Mothers Value and Utilize Early Outpatient Education on Breast Massage and Hand Expression in Their Self-Management of Engorgement. *Breastfeed Med* 2016, 11(9):433-439.

557. Woodley SJ, Lawrenson P, Boyle R, Cody JD, Mørkved S, Kernohan A, Hay-Smith EJC: Pelvic floor muscle training for preventing and treating urinary and faecal incontinence in antenatal and postnatal women. In., vol. 2020; 2020.

558. Woolhouse H, Mercuri K, Judd F, Brown SJ: Antenatal mindfulness intervention to reduce depression, anxiety and stress: a pilot randomised controlled trial of the MindBabyBody program in an Australian tertiary maternity hospital. *BMC Pregnancy Childbirth* 2014, 14(1):369.

559. Wulff V, Hepp P, Wolf OT, Balan P, Hagenbeck C, Fehm T, Schaal NK: The effects of a music and singing intervention during pregnancy on maternal well-being and mother-infant bonding: a randomised, controlled study. *Arch Gynecol Obstet* 2021, 303(1):69-83.

560. Yakoob MY, Qadir M, Hany OE: Vitamin A Supplementation for Prevention and Treatment of Malaria during Pregnancy and Childhood: A Systematic Review and Meta-analysis. *J Epidemiol Glob Health* 2018, 8(1-2):20-28.

561. Yan CF, Hung YC, Gau ML, Lin KC: Effects of a stability ball exercise programme on low back pain and daily life interference during pregnancy. *Midwifery* 2014, 30(4):412-419.

562. Yang X, Tian H, Zhang F, Zhang C, Li Y, Leng J, Wang L, Liu G, Dong L, Yu Z *et al*: A randomised translational trial of lifestyle intervention using a 3-tier shared care approach on pregnancy outcomes in Chinese women with gestational diabetes mellitus but without diabetes. *J Transl Med* 2014, 12(1):290.

563. Yaping X, Huifen Z, Meijing Z, Huibin H, Chunhong L, Fengfeng H, Jingjing W: Effects of Moderate-Intensity Aerobic Exercise on Blood Glucose Levels and Pregnancy Outcomes in Patients With Gestational Diabetes Mellitus: A Randomized Controlled Trial. *Diabetes Ther* 2021, 12(9):2585-2598.

564. Yerlikaya-Schatten G, Trimmal L, Rosicky I, Husslein P, Schatten C, Eppel D, Eppel W, Tura A, Gobl CS: Effects of gum chewing on glycaemic control in women with gestational diabetes mellitus: A randomized controlled trial. Impact of chewing on hyperglycaemia in women with GDM. *Eur J Obstet Gynecol Reprod Biol* 2020, 247:61-65.

565. Yew TW, Chi C, Chan SY, van Dam RM, Whitton C, Lim CS, Foong PS, Fransisca W, Teoh CL, Chen J *et al*: A Randomized Controlled Trial to Evaluate the Effects of a Smartphone Application-Based Lifestyle Coaching Program on Gestational Weight Gain, Glycemic Control, and Maternal and Neonatal Outcomes in Women With Gestational Diabetes Mellitus: The SMART-GDM Study. *Diabetes Care* 2021, 44(2):456-463.

566. Yuksel H, Cayir Y, Kosan Z, Tastan K: Effectiveness of breathing exercises during the second stage of labor on labor pain and duration: a randomized controlled trial. *J Integr Med* 2017, 15(6):456-461.

567. Zairina E, Abramson MJ, McDonald CF, Li J, Dharmasiri T, Stewart K, Walker SP, Paul E, George J: Telehealth to improve asthma control in pregnancy: A randomized controlled trial. *Respirology* 2016, 21(5):867-874.

568. Zairina E, Stewart K, Abramson MJ, George J: The effectiveness of non-pharmacological healthcare interventions for asthma management during pregnancy: a systematic review. *BMC Pulm Med* 2014, 14(1):46.

For peer review only - http://bmjopen.bmj.com/site/about/guidelines.xhtml

569. Zakarija-Grkovic I, Stewart F: Treatments for breast engorgement during lactation. *Cochrane Database Syst Rev* 2020, 9:CD006946.

570. Zang Y, Lu H, Zhang H, Huang J, Ren L, Li C: Effects of upright positions during the second stage of labour for women without epidural analgesia: A meta-analysis. In., vol. 76; 2020.

571. Zang Y, Lu H, Zhang H, Huang J, Zhao Y, Ren L: Benefits and risks of upright positions during the second stage of labour: An overview of systematic reviews. In., vol. 114; 2021.

572. Zarei MR, Khachian A, Mohammadi N, Haghani H, Rostami F: The effect of self-care education program on wound healing in women undergoing cesarean section. *Annals of Tropical Medicine and Public Health* 2018, 11(Special Issue).

573. Zarenejad M, Yazdkhasti M, Rahimzadeh M, Mehdizadeh Tourzani Z, Esmaelzadeh-Saeieh S: The effect of mindfulness-based stress reduction on maternal anxiety and self-efficacy: A randomized controlled trial. *Brain Behav* 2020, 10(4):e01561.

574. Zelkowitz P, Feeley N, Shrier I, Stremler R, Westreich R, Dunkley D, Steele R, Rosberger Z, Lefebvre F, Papageorgiou A: The cues and care randomized controlled trial of a neonatal intensive care unit intervention: effects on maternal psychological distress and mother-infant interaction. *J Dev Behav Pediatr* 2011, 32(8):591-599.

575. Zemestani M, Fazeli Nikoo Z: Effectiveness of mindfulness-based cognitive therapy for comorbid depression and anxiety in pregnancy: a randomized controlled trial. *Arch Womens Ment Health* 2020, 23(2):207-214.

576. Zhang MM, Zou Y, Li SM, Wang L, Sun YH, Shi L, Lu L, Bao YP, Li SX: The efficacy and safety of omega-3 fatty acids on depressive symptoms in perinatal women: a meta-analysis of randomized placebo-controlled trials. *Transl Psychiatry* 2020, 10(1):193.

577. Zhang X, Lin P, Sun J, Sun Y, Shao D, Cao D, Cao F: Prenatal stress self-help mindfulness intervention via social media: a randomized controlled trial. *J Ment Health* 2021:1-10.

578. Zhao Y, Lin Q, Zhu X, Wang J: Randomized Clinical Trial of a Prenatal Breastfeeding and Mental Health Mixed Management Intervention. *J Hum Lact* 2021, 37(4):761-774.

579. Zhu YP, Wang WJ, Zhang SL, Dai B, Ye DW: Effects of gum chewing on postoperative bowel motility after caesarean section: A meta-analysis of randomised controlled trials. In., vol. 121; 2014.

580. Zlotnick C, Tzilos G, Miller I, Seifer R, Stout R: Randomized controlled trial to prevent postpartum depression in mothers on public assistance. *J Affect Disord* 2016, 189:263-268.

For peer review only

For peer review only - http://bmjopen.bmj.com/site/about/guidelines.xhtml

Supplementary File S8. Characteristics of studies by category

A. Distribution by study design

Category	Total no. of studies	Observational & diagnostic studies	Non-randomised interventional studies	Randomised controlled trials	Evidence synthesis studies
Diet & nutrition	143	3 (2%)	4 (3%)	66 (46%)	70 (49%)
Physical activities	132	4 (3%)	11 (8%)	76 (58%)	41 (31%)
Lifestyle adjustments	90	11 (12%)	7 (8%)	48 (53%)	24 (27%)
Complementary & alternative therapies	64	3 (5%)	3 (5%)	32 (50%)	26 (41%)
Psycho-social strategies	97	2 (2%)	15 (15%)	58 (60%)	22 (23%)
Breast & nipple care	22	1 (5%)	4 (18%)	8 (36%)	9 (41%)
Sexual health & family planning	12	0 (0%)	0 (0%)	10 (83%)	2 (17%)
Self-monitoring	69	8 (12%)	8 (12%)	36 (52%)	17 (25%)
Self-management of medication	17	1 (6%)	7 (41%)	6 (35%)	3 (18%)
Self-testing or sampling	18	10 (56%)	2 (11%)	6 (33%)	0 (0%)
Other self-management skills	54	8 (15%)	3 (6%)	23 (43%)	20 (37%)

B. Distribution by time of intervention

Category	Total no. of studies	Postpartum	Intrapartum	Antenatal	Preconception
Diet & nutrition	143	27 (19%)	4 (3%)	123 (86%)	7 (5%)
Physical activities	132	25 (19%)	7 (5%)	110 (83%)	3 (2%)
Lifestyle adjustments	90	10 (11%)	0 (0%)	84 (93%)	3 (3%)
Complementary & alternative therapies	64	22 (34%)	23 (36%)	19 (30%)	0 (0%)
Psycho-social strategies	97	21 (22%)	10 (10%)	72 (74%)	1 (1%)
Breast & nipple care	22	14 (64%)	1 (5%)	7 (32%)	0 (0%)
Sexual health & family planning	12	3 (25%)	0 (0%)	10 (83%)	0 (0%)
Self-monitoring	69	9 (13%)	0 (0%)	63 (91%)	4 (6%)
Self-management of medication	17	3 (18%)	3 (18%)	14 (82%)	1 (6%)
Self-testing or sampling	18	1 (6%)	0 (0%)	18 (100%)	0 (0%)
Other self-management skills	54	11 (20%)	28 (52%)	20 (37%)	1 (2%)

**Some studies feature self-care that can be carried out in more than one pregnancy periods; hence the percentages do not total 100%.*

For peer review only - http://bmjopen.bmj.com/site/about/guidelines.xhtml

C. Distribution by venue of access to intervention

Category	Total no. of studies	Peer/partner support	Home-based care	Community-based healthcare	Digital technologies / platforms	OTC availability	Health facilities
Diet & nutrition	143	3 (2%)	22 (15%)	12 (8%)	25 (17%)	21 (15%)	37 (26%)
Physical activities	132	6 (5%)	8 (6%)	37 (28%)	26 (20%)	6 (5%)	50 (38%)
Lifestyle adjustments	90	4 (4%)	18 (20%)	6 (7%)	17 (19%)	8 (9%)	32 (36%)
Complementary & alternative therapies	64	2 (3%)	11 (17%)	1 (2%)	0 (0%)	25 (39%)	27 (42%)
Psycho-social strategies	97	8 (8%)	10 (10%)	14 (14%)	18 (19%)	1 (1%)	40 (41%)
Breast & nipple care	22	0 (0%)	5 (23%)	0 (0%)	1 (5%)	6 (27%)	7 (32%)
Sexual health & family planning	12	2 (17%)	0 (0%)	2 (17%)	0 (0%)	0 (0%)	8 (67%)
Self-monitoring	69	1 (1%)	5 (7%)	4 (6%)	35 (51%)	0 (0%)	40 (58%)
Self-management of medication	17	3 (18%)	0 (0%)	7 (41%)	1 (6%)	0 (0%)	7 (41%)
Self-testing or sampling	18	1 (6%)	1 (6%)	2 (11%)	0 (0%)	0 (0%)	17 (94%)
Other self-management skills	54	6 (11%)	4 (7%)	2 (4%)	5 (9%)	3 (6%)	24 (44%)

**Some studies feature more than one venues of access to intervention; hence the percentages do not total 100%. Evidence synthesis studies were not assessed as the included studies might have different venues of access or there might be insufficient information to determine venues of access for all included studies.*

D. Distribution by persons providing external support

Category	Total no. of studies	Family & other community members	Community health workers	Healthcare workers	No external support
Diet & nutrition	143	4 (3%)	9 (6%)	38 (27%)	27 (19%)
Physical activities	132	15 (11%)	17 (13%)	58 (44%)	19 (14%)
Lifestyle adjustments	90	1 (1%)	2 (2%)	37 (41%)	26 (29%)
Complementary & alternative therapies	64	3 (5%)	0 (0%)	17 (27%)	19 (30%)
Psycho-social strategies	97	10 (10%)	2 (2%)	44 (45%)	23 (24%)
Breast & nipple care	22	0 (0%)	0 (0%)	3 (14%)	9 (41%)
Sexual health & family planning	12	0 (0%)	2 (17%)	8 (67%)	0 (0%)
Self-monitoring	69	2 (3%)	3 (4%)	30 (43%)	18 (26%)
Self-management of medication	17	0 (0%)	7 (41%)	5 (29%)	2 (12%)
Self-testing or sampling	18	0 (0%)	2 (11%)	7 (39%)	10 (56%)
Other self-management skills	54	1 (2%)	2 (4%)	12 (22%)	20 (37%)

**Some studies feature more than one sources of external support; hence the percentages do not total 100%. Evidence synthesis studies were not assessed as the included studies might have different sources of external support or there might be insufficient information to determine sources of external support for all included studies.*

For peer review only - http://bmjopen.bmj.com/site/about/guidelines.xhtml

Supplementary File S9. Complex interventions and their characteristics (N=35)

A. Self-care activities featured as a component of complex interventions

Self-care activity	No. of studies (%)
**Diet & nutrition**	
Dietary adjustment	19 (54%)
Dietary intake - Iodine	1 (3%)
Supplement intake - Calcium	2 (6%)
Supplement intake - Iron and/or folic acid (IFA)	5 (14%)
Supplement intake - not otherwise specified	2 (6%)
Caffeine intake reduction	1 (3%)
**Physical activity**	
Birthing/exercise balls	3 (9%)
Pelvic floor exercises	1 (3%)
Yoga	2 (6%)
Physical activity (not otherwise specified)	14 (40%)
**Lifestyle adjustments**	
Air pollution avoidance	1 (3%)
Alcohol use reduction	6 (17%)
Avoiding strenuous activities	3 (9%)
Deworming	1 (3%)
Drug use reduction	2 (6%)
Oral hygiene	2 (6%)
Insecticide-treated nets	1 (3%)
Sleep hygiene	4 (11%)
Smoking cessation	5 (14%)
TORCH prevention	1 (3%)
Urogenital hygiene	3 (9%)
Other risk reduction strategies (traffic accident, X-ray exposure)	1 (3%)
General self-care to relieve physiological discomforts (not otherwise specified)	6 (17%)
**Complementary & alternative therapies**	
Acupressure	1 (3%)
Aromatherapy	2 (6%)
General massage	9 (26%)
Shower/bath	2 (6%)
**Psycho-social strategies**	
Interpersonal relationship & support network	10 (29%)
Mind-body relaxation	16 (46%)
Music therapy	4 (11%)
Other emotion management strategies	11 (31%)
**Breast & nipple care**	
Breast/nipple care (not otherwise specified)	3 (9%)
Breastfeeding techniques	12 (34%)
**Sexual health & postpartum family planning**	
Condom use	2 (6%)
HIV disclosure	2 (6%)

For peer review only - http://bmjopen.bmj.com/site/about/guidelines.xhtml

Self-care activity	No. of studies (%)
Intimate partner violence (IPV) prevention	3 (9%)
Safe and healthy sexual life	2 (6%)
Sexual negotiation	1 (3%)
STI prevention (not otherwise specified)	1 (3%)
**Self-monitoring**	
Foetal movement monitoring	2 (6%)
Home-based records	2 (6%)
Recognising signs of labour	6 (17%)
Recognising signs of complications	6 (17%)
Self-monitoring of uterine contraction	1 (3%)
Self-monitoring weight	5 (14%)
Self-monitoring blood glucose	2 (6%)
Self-monitoring blood pressure	1 (3%)
**Self-management of medication**	
Self-medication	2 (6%)
Adherence to PMTCT & ART	1 (3%)
**Other self-management skills**	
Birth plans	7 (20%)
Birth position	5 (14%)
Techniques during childbirth (not otherwise specified)	3 (9%)
Postoperative wound care	1 (3%)

Note: Each of these self-care activities is often a component of a larger intervention, which feature more than 3 different self-care activities.

B. Health issues targeted by complex interventions

Target outcomes	No. of studies (%)
Care-seeking behaviours	1 (3%)
Diabetes mellitus	2 (6%)
Fatigue	1 (3%)
HIV/STDs	2 (6%)
Hypertensive disorders of pregnancy	1 (3%)
Low birth weight	1 (3%)
Nutritional deficiencies	2 (6%)
Physiological discomforts	1 (3%)
Positive childbirth experience	9 (26%)
Postpartum health	1 (3%)
Preconception health	3 (9%)
Preterm birth	1 (3%)
Psychological wellbeing	11 (31%)
Various maternal & neonatal outcomes	3 (9%)
Weight management	3 (9%)

For peer review only - http://bmjopen.bmj.com/site/about/guidelines.xhtml

Supplementary File S10. List of all self-care interventions identified from included studies

Self-care intervention	Description	No. of studies (%, N=545)	Health issues / outcomes targeted by intervention	Periods of intervention*
**Diet & nutrition**		**143 (26%)<br>P: 73<br>E: 70**		
Dietary adjustment	Modifying daily intakes of vegetables, fruit, water, salt, fat, sugar, etc. or total calorie consumption	75 (14%)<br>P: 49 †<br>E: 26	Birth weight, diabetes mellitus, hypertensive disorders of pregnancy, nutritional deficiencies, physiological discomforts, preconception health, preterm birth, psychological wellbeing, weight management	Preconception<br>Antenatal<br>Postpartum
Dietary intake - Calcium	From diet (dairy products and green leafy vegetables)	1 (0.2%)<br>P: 0<br>E: 1	Various maternal & neonatal outcomes	Antenatal<br>Postpartum
Dietary intake - Omega-3 fatty acids	From diet (fish, nuts and seeds, plant oils)	1 (0.2%)<br>P: 1<br>E: 0	Psychological wellbeing	Antenatal
Supplement intake - Calcium		5 (1%)<br>P: 0<br>E: 5	Hypertensive disorders of pregnancy, nutritional deficiencies, preterm birth	Preconception<br>Antenatal<br>Postpartum
Supplement intake - Iodine		2 (0.4%)<br>P: 0<br>E: 2	Various maternal & neonatal outcomes	Preconception<br>Antenatal<br>Postpartum
Supplement intake - Iron and/or folic acid (IFA)	Potential interaction with some medications e.g. malaria chemoprophylaxis<br>Including self-management of weekly IFA supplements (n=2)	12 (2%)<br>P: 5<br>E: 7	Diabetes mellitus, hypertensive disorders of pregnancy, low birth weight, nutritional deficiencies, preconception health, preterm birth	Preconception<br>Antenatal<br>Postpartum
Supplement intake - Lipid-based nutrients	Fortified products which provide energy mainly in the form of lipids, and additionally other nutrients (protein, essential fatty acids, micronutrients)	2 (0.4%)<br>P: 2<br>E: 0	Various maternal & neonatal outcomes	Antenatal
Supplement intake - Multiple-micronutrients	Supplements containing 13-15 micronutrients (vitamins and minerals) in one tablet, including UNIMMAP formulation	9 (2%)<br>P: 4<br>E: 5	Diabetes mellitus, nutritional deficiencies, preterm birth, other maternal & neonatal outcomes	Preconception<br>Antenatal
Supplement intake - Omega-3 fatty acids	Including DHA, EHA, fish oils etc.	11 (2%)<br>P: 3<br>E: 8	Diabetes mellitus, preeclampsia, preterm birth, postpartum depression	Preconception<br>Antenatal<br>Postpartum
Supplement intake - Vitamin A	Only recommended in areas with endemic vitamin A deficiency or night blindness	2 (0.4%)<br>P: 1<br>E: 1	Bacterial vaginosis, malaria, nutritional deficiencies, preterm birth	Antenatal<br>Postpartum
Supplement intake - Vitamin B		1 (0.2%)<br>P: 0<br>E: 1	Various maternal & neonatal outcomes	Antenatal<br>Intrapartum
Supplement intake - Vitamin C		2 (0.4%)<br>P: 0<br>E: 2	Urinary tract infections, other maternal & neonatal outcomes	Antenatal
Supplement intake - Vitamin D		2 (0.4%)<br>P: 0<br>E: 2	Diabetes mellitus, other maternal & neonatal outcomes	Preconception<br>Antenatal
Supplement intake - Vitamin E		1 (0.2%)<br>P: 0<br>E: 1	Various maternal & neonatal outcomes	Antenatal
Supplement intake - Zinc		2 (0.4%)<br>P: 0<br>E: 2	Preterm birth, other maternal & neonatal outcomes	Antenatal
Supplement intake - not otherwise specified		1 (0.2%)<br>P: 0<br>E: 1	Hypertensive disorders of pregnancy	Antenatal
Caffeine intake reduction		1 (0.2%)<br>P: 0<br>E: 1	Various maternal & neonatal outcomes	Antenatal
Energy drinks during childbirth	Drinks containing various nutrients and electrolytes	1 (0.2%)<br>P: 1<br>E: 0	Positive labour outcomes	Intrapartum
Fibre supplement	Psyllium, methycellulose, calcium polycarbophil etc. that function as bulk-forming laxatives	3 (1%)<br>P: 1<br>E: 2	Constipation, haemorrhoids, physiological discomforts	Antenatal

Self-care intervention	Description	No. of studies (%, N=545)	Health issues / outcomes targeted by intervention	Periods of intervention*
Gum chewing	Sugar-free gum for chewing to induce satiation or bowel movement	5 (1%)<br>P: 3<br>E: 2	Diabetes mellitus, positive childbirth experience, postoperative bowel function	Antenatal<br>Intrapartum<br>Postpartum
Probiotics	Products containing Lactobacillus, Bifidobacterium and/or Enterococcus sp.	19 (3%)<br>P: 8<br>E: 11	Bacterial vaginosis, birth weight, diabetes mellitus, group B streptococcus infection, mastitis, preterm birth, psychological wellbeing, weight management	Preconception<br>Antenatal<br>Postpartum
Other dietary/nutritional products	Fruit juices, herbal products, any other product containing natural ingredients that were not regulated as medicine	13 (2%)<br>P: 3<br>E: 10	Diabetes mellitus, induction of labour, mastitis, physiological discomforts, postpartum healing & pain relief, psychological wellbeing, sleep quality, urinary tract infections	Preconception<br>Antenatal<br>Intrapartum<br>Postpartum
**Physical activity**		**132 (24%)<br>P: 91<br>E: 41**		
Ankle weights	Worn during daily activities as a resistance exercise	1 (0.2%)<br>P: 1<br>E: 0	Weight management	Postpartum
Aquatic exercises	Exercises conduct under water (e.g. in a swimming pool)	4 (1%)<br>P: 3<br>E: 1	Labour pain, lumbopelvic pain, physiological discomforts, psychological wellbeing, sleep quality	Antenatal
Birthing/exercise balls	Exercises using Swiss balls, exercise balls or peanut balls, etc.	7 (1%)<br>P: 5<br>E: 2	Childbirth self-efficiency (CBSE), labour pain, lumbopelvic pain, positive childbirth experience, positive labour outcomes	Antenatal<br>Intrapartum
Pelvic floor exercises	Exercises aimed at strengthening pelvic floor muscles, e.g. Kegel exercises	17 (3%)<br>P: 13<br>E: 4	Diabetes mellitus, lumbopelvic pain, pelvic floor disorders, physiological discomforts, positive childbirth experience, sexual functioning, weight management	Antenatal<br>Intrapartum<br>Postpartum
Yoga	Physical component (yoga poses) only; meditation component was classified as mind-body relaxation	15 (3%)<br>P: 8<br>E: 7	Diabetes mellitus, labour pain, lumbopelvic pain, physiological discomforts, positive childbirth experience, psychological wellbeing, sleep quality	Antenatal<br>Intrapartum<br>Postpartum
Physical activity (not otherwise specified)		99 (18%)<br>P: 67<br>E: 32	Birth weight, cardiometabolic fitness, diabetes mellitus, fatigue, foetal health, hypertensive disorders of pregnancy, lumbopelvic pain, pelvic floor disorders, physiological discomforts, preconception health, preeclampsia, preterm birth, psychological wellbeing, sleep quality, weight management	Preconception<br>Antenatal<br>Postpartum
**Lifestyle adjustments**		**90 (17%)<br>P: 66<br>E: 24**		
Air pollution avoidance	Avoiding going outdoor when air pollution levels are high	3 (1%)<br>P: 2<br>E: 1	Air pollution exposure, stillbirth	Antenatal
Alcohol use reduction	Self-initiated abstinence or relapse prevention without pharmacological treatment	7 (1%)<br>P: 3<br>E: 4	Alcohol consumption	Preconception<br>Antenatal<br>Postpartum
Avoiding strenuous activities	Sleeping with pillows, ergonomic postures in daily activities (lifting, bending), etc.	2 (0.4%)<br>P: 2<br>E: 0	Lumbopelvic pain	Antenatal
Bed rest during pregnancy	Ranging from selected activity restriction to strict bed rest	1 (0.2%)<br>P: 0<br>E: 1	Various maternal & neonatal outcomes	Antenatal
Clean cookstoves	Cookstoves using cleaner energy sources (electricity, ethanol or biogas) instead of open fire, kerosene or biomass	5 (1%)<br>P: 5<br>E: 0	Hypertensive disorders of pregnancy, low birth weight, respiratory irritation	Antenatal
General self-care to relieve physiological discomforts (not otherwise specified)		3 (1%)<br>P: 3<br>E: 0	Psychological wellbeing	Antenatal<br>Postpartum
Hand hygiene	Washing hands, avoiding exposure to saliva from young children (sharing food and utensils, kissing)	5 (1%)<br>P: 4<br>E: 1	Cytomegalovirus infection	Preconception<br>Antenatal
Oral hygiene	Tooth brushing, flossing, using oral rinses, etc.	6 (1%)<br>P: 5<br>E: 1	Periodontal diseases	Antenatal

For peer review only - http://bmjopen.bmj.com/site/about/guidelines.xhtml

Self-care intervention	Description	No. of studies (%, N=545)	Health issues / outcomes targeted by intervention	Periods of intervention*
Mosquito-repellent strategies - Insecticide-treated nets	Bed nets impregnated with insecticides (e.g. pyrroles, pyrethroids)	12 (2%)<br>P: 11<br>E: 1	Low birth weight, malaria, preterm birth	Antenatal
Mosquito-repellent strategies - Other	Use of insecticides, mosquito coils, fans, insect-repellent cream, wearing long sleeves, burning local plants, etc.	1 (0.2%)<br>P: 1<br>E: 0	Malaria	Antenatal
Second-hand smoke exposure prevention	Establishing a smoke-free home/office, communicating with smokers	3 (1%)<br>P: 3<br>E: 0	Second-hand smoke exposure	Antenatal
Sleep hygiene	Sleeping schedules, good sleep habits, improving sleeping environment	4 (1%)<br>P: 3<br>E: 1	Fatigue, sleep quality	Antenatal<br>Postpartum
Sleeping position	Choosing to left lateral recumbent sleeping position / avoiding supine position, raising the head to relieve heartburn	6 (1%)<br>P: 2<br>E: 4	Hypertensive disorders of pregnancy, low birth weight, physiological discomforts, stillbirth	Antenatal
Smoking cessation	Self-initiated cessation or relapse prevention without pharmacological treatment	32 (6%)<br>P: 22<br>E: 10	Low birth weight, neonatal death, smoking status, stillbirth, other maternal & neonatal outcomes	Antenatal<br>Postpartum
Urogenital hygiene	Hygiene behaviours (daily cleaning, using clean water, using pads)	2 (0.4%)<br>P: 1<br>E: 1	Sexual functioning, urinary tract infections	Antenatal
**Complementary & alternative therapies**		**64 (12%)<br>P: 38<br>E: 26**		
Acupressure	Applying pressure on specific acupoints. Only manoeuvres that can be administered by the woman herself or her partner are included; reflexology by HCWs is excluded.	13 (2%)<br>P: 6<br>E: 7	Breast engorgement, labour pain, mastitis, perineal healing & pain relief, physiological discomforts, positive labour outcomes, postpartum healing & pain relief	Antenatal<br>Intrapartum<br>Postpartum
Aromatherapy	Including the use of essential oils	7 (1%)<br>P: 2<br>E: 5	Labour pain, perineal healing & pain relief, psychological wellbeing, sleep quality	Intrapartum<br>Postpartum
Bright light therapy	Exposure to natural light (getting outdoor) or artificial light (using a light therapy lamp)	3 (1%)<br>P: 1<br>E: 2	Psychological wellbeing	Antenatal<br>Postpartum
Compress - cold	Ice packs, cryotherapy	9 (2%)<br>P: 5<br>E: 4	Labour pain, perineal healing & pain relief	Intrapartum<br>Postpartum
Compress - hot	Hot water packs, heat therapy	3 (1%)<br>P: 2<br>E: 1	Labour pain, perineal healing & pain relief	Intrapartum<br>Postpartum
Compression stockings	Stockings designed to promote circulation in the legs via gentle compression	1 (0.2%)<br>P: 0<br>E: 1	Physiological discomforts	Antenatal
Continuous positive airway pressure (CPAP)	Delivering positive airway pressure to keep airway open during sleep. CPAP machines are available over-the-counter and do not require specialised skills to operate.	1 (0.2%)<br>P: 1<br>E: 0	Diabetes mellitus, obstructive sleep apnoea	Antenatal
General massage	Any form of body massage	17 (3%)<br>P: 7<br>E: 10	Labour pain, lumbopelvic pain, physiological discomforts, positive childbirth experience, postpartum healing & pain relief, psychological wellbeing, sleep quality, striae gravidarum	Antenatal<br>Intrapartum<br>Postpartum
Kinesiologic taping	Adhesive tape applied to the specific region of the back to provide support	1 (0.2%)<br>P: 1<br>E: 0	Labour pain	Intrapartum
Pelvic support devices	Any device worn at the lower abdominal level to support the pelvic or lumbosacral region e.g. pelvic belts/girdles, abdominal binders, elastomeric fabric orthoses etc.	5 (1%)<br>P: 4<br>E: 1	C-section wound healing, lumbopelvic pain, postpartum healing & pain relief	Antenatal<br>Postpartum
Perineal massage	Can be administered by either the woman herself or the partner in the antenatal or postpartum period	4 (1%)<br>P: 1<br>E: 3	Positive childbirth experience, postpartum healing & pain relief, prevention of perineal injury	Antenatal<br>Intrapartum
Shower/bath	Including birthing pools, shower before labour, foot baths and Sitz baths	15 (3%)<br>P: 7<br>E: 8	Duration of labour, haemorrhoids, labour pain, lumbopelvic pain, perineal healing & pain relief, physiological discomforts, positive	Antenatal<br>Intrapartum<br>Postpartum

For peer review only - http://bmjopen.bmj.com/site/about/guidelines.xhtml

Self-care intervention	Description	No. of studies (%, N=545)	Health issues / outcomes targeted by intervention	Periods of intervention*
			childbirth experience, positive labour outcomes	
Topical products	Ointments, creams, etc. that do not contain medicinal ingredients and are available over-the-counter	5 (1%)<br>P: 3<br>E: 2	Haemorrhoids, postpartum healing & pain relief, striae gravidarum	Antenatal<br>Postpartum
Transcutaneous electrical nerve stimulation (TENS)	Delivering small electrical impulses through electrodes placed on the skin. TENS machines are available over-the-counter and do not require specialised skills to operate.	9 (2%)<br>P: 4<br>E: 5	Labour pain, lumbopelvic pain, postpartum healing & pain relief	Antenatal<br>Intrapartum<br>Postpartum
**Psycho-social strategies**		**97 (18%)<br>P: 75<br>E: 22**		
Interpersonal relationship & support network	Establishing social support, relationship management, communication with others	23 (4%)<br>P: 20<br>E: 3	Diabetes mellitus, intimate partner violence (IPV), psychological wellbeing	Preconception<br>Antenatal<br>Postpartum
Mind-body relaxation	Breathing exercises, guided imagery, progressive muscle relaxation, self-hypnosis, mindfulness (in meditation exercises or daily practices)	63 (12%)<br>P: 45<br>E: 18	Breast & nipple pain, diabetes mellitus, hypertensive disorders of pregnancy, labour pain, lumbopelvic pain, physiological discomforts, positive childbirth experience, positive labour outcomes, postpartum healing & pain relief, psychological wellbeing, sleep quality	Antenatal<br>Intrapartum<br>Postpartum
Music therapy	Listening to music (lullabies, classical music, nature round), either standalone or combined with other techniques (meditation, breathing)	11 (2%)<br>P: 7<br>E: 4	Labour pain, positive childbirth experience, postpartum healing & pain relief, psychological wellbeing, sleep quality	Antenatal<br>Intrapartum<br>Postpartum
Other emotion management strategies	Cognitive restructuring, problem-solving, mood-tracking, etc.	32 (6%)<br>P: 30<br>E: 2	Diabetes mellitus, hypertensive disorders of pregnancy, positive childbirth experience, psychological wellbeing	Antenatal<br>Postpartum
**Breast & nipple care**		**22 (4%)<br>P: 13<br>E: 9**		
Breast compress (hot & cold)	Warm compress (warm towels, heat packs) to promote milk flow before breastfeeding; cold compress (cold gel packs, cold cabbage leaf) to stop/reduce milk flow in case of engorgement	2 (0.4%)<br>P: 1<br>E: 1	Breast engorgement	Postpartum
Breast milk expression	Can be conducted prenatally or postnatally (for colostrum extraction, a few hours after birth), using manual extraction, breast pumps or via breastfeeding	5 (1%)<br>P: 3<br>E: 2	Breast engorgement, various neonatal outcomes	Antenatal<br>Postpartum
Breast massage	Massaging techniques to promote blood circulation and lymphatic drainage	5 (1%)<br>P: 1<br>E: 4	Breast & nipple pain, breast engorgement, mastitis	Postpartum
Breast/nipple shells	Plastic parts worn over the breast to protect the nipples	2 (0.4%)<br>P: 1<br>E: 1	Breast & nipple pain	Postpartum
Hydrogel dressings	Adhesive hydrogel discs or pads applied over breast or nipple to form a protective cushion, support healing and provide cooling effect	2 (0.4%)<br>P: 1<br>E: 1	Breast & nipple pain	Postpartum
Lanolin ointment	Applied over nipple to form a protective layer and promote healing	5 (1%)<br>P: 4<br>E: 1	Breast & nipple pain	Postpartum
Breast/nipple care (not otherwise specified)		1 (0.2%)<br>P: 1<br>E: 0	Breast & nipple pain	Postpartum
Antenatal education on breastfeeding techniques	Any technique for effective breastfeeding (e.g. timing, holding the newborn, latching) taught in the antenatal period	6 (1%)<br>P: 4<br>E: 2	Breast & nipple pain, breast engorgement, psychological wellbeing	Antenatal<br>Postpartum
**Sexual health & postpartum family planning**		**12 (2%)<br>P: 10<br>E: 2**		
Condom use	For STI prevention during pregnancy	2 (0.4%)<br>P: 2<br>E: 0	HIV/STI	Antenatal

For peer review only - http://bmjopen.bmj.com/site/about/guidelines.xhtml

Self-care intervention	Description	No. of studies (%, N=545)	Health issues / outcomes targeted by intervention	Periods of intervention*
HIV disclosure	Deciding when and how to disclose HIV status to family and partner	1 (0.2%)<br>P: 1<br>E: 0	HIV	Antenatal
Intimate partner violence (IPV) prevention	Safety planning, managing conflicts with partner	6 (1%)<br>P: 4<br>E: 2	IPV, psychological wellbeing, sexual functioning	Antenatal<br>Postpartum
Safe and healthy sexual life	Safe sex positions, sexual restrictions during pregnancy, restarting sex after pregnancy, coping with changing sexuality	5 (1%)<br>P: 5<br>E: 0	Sexual functioning	Antenatal<br>Postpartum
Sexual negotiation	Sexual disclosure to partners, refusing risky/unprotected sex	2 (0.4%)<br>P: 1<br>E: 1	HIV/STI, IPV	Antenatal
STI prevention (not otherwise specified)		3 (1%)<br>P: 3<br>E: 0	HIV/STI, sexual functioning	Antenatal
**Self-monitoring**		**69 (13%)<br>P: 52<br>E: 17**		
Foetal heartbeat self-monitoring	Using a home cardiotocography device	1 (0.2%)<br>P: 1<br>E: 0	Detection of abnormal foetal heart rate	Antenatal
Foetal movement monitoring	Counting or observing foetal movements	7 (1%)<br>P: 4<br>E: 3	Perinatal mortality, psychological wellbeing	Antenatal
Home-based records	Home-based records to keep track of multiple health indicators, such as weight, mood, diet, physical activity, medication use, vaccination records, etc.	4 (1%)<br>P: 2<br>E: 2	Diabetes mellitus, health service utilisation, psychological wellbeing, other maternal & neonatal outcomes	Preconception<br>Antenatal<br>Postpartum
Recognising signs of labour	Differentiate false vs true labour, recognising when to notify health providers	2 (0.4%)<br>P: 0<br>E: 2	Positive childbirth experience	Antenatal
Self-assessment of foetal presentation	Self-palpation to detect spontaneous reversion to breech presentation	1 (0.2%)<br>P: 1<br>E: 0	Breech presentation	Antenatal
Self-measurement of symphysis-fundus height	Measuring distance from the symphysis pubis to the uterine fundus with a tape measure	1 (0.2%)<br>P: 1<br>E: 0	Intrauterine growth restriction	Antenatal
Self-monitoring lung function	Using handheld respiratory devices	1 (0.2%)<br>P: 1<br>E: 0	Asthma control	Antenatal
Self-monitoring of ketonuria	Using urine dipsticks	1 (0.2%)<br>P: 1<br>E: 0	Diabetes mellitus	Antenatal
Self-monitoring of uterine contraction	Self-palpation and counting contractions	1 (0.2%)<br>P: 1<br>E: 0	Uterine contraction awareness	Antenatal
Self-monitoring weight	Often accompanying lifestyle interventions for weight loss	17 (3%)<br>P: 16<br>E: 1	Preconception health, weight management, other maternal & neonatal outcomes	Preconception<br>Antenatal<br>Postpartum
Self-monitoring blood glucose (Continuous glucose monitoring or CGM)	Using a CGM device that measures interstitial blood glucose levels. Only interventions where CGM data is accessible for pregnant women for lifestyle adjustments or insulin dose self-titration were included.	8 (1%)<br>P: 6<br>E: 2	Diabetes mellitus	Preconception<br>Antenatal
Self-monitoring blood glucose (Flash glucose monitoring or FGM)	Similar to CGM but only displaying glucose results when activated via phone or device	1 (0.2%)<br>P: 1<br>E: 0	Diabetes mellitus	Antenatal
Self-monitoring blood glucose (not otherwise specified)	Any interventions involving SMBG using glucometers, CGM or FGM devices	16 (3%)<br>P: 9<br>E: 7	Diabetes mellitus, other maternal & neonatal outcomes	Preconception<br>Antenatal<br>Postpartum
Self-monitoring blood pressure	Blood pressure monitors are available over-the-counter	12 (2%)<br>P: 10<br>E: 2	Diabetes mellitus, hypertensive disorders of pregnancy, postpartum hypertension	Antenatal<br>Postpartum

For peer review only - http://bmjopen.bmj.com/site/about/guidelines.xhtml

Self-care intervention	Description	No. of studies (%, N=545)	Health issues / outcomes targeted by intervention	Periods of intervention*
**Self-management of medication**		**17 (3%)<br>P: 14<br>E: 3**		
Self-adjustment of antihypertensive medication	Guided by a blood pressure-based algorithm	1 (0.2%)<br>P: 1<br>E: 0	Postpartum hypertension	Postpartum
Self-administration of antibiotics	Distributed during antenatal visits to be administered by the woman after birth	1 (0.2%)<br>P: 1<br>E: 0	Postpartum infection	Antenatal<br>Intrapartum
Self-administration of misoprostol	Distributed during antenatal visits to be administered by the woman after birth	10 (2%)<br>P: 8<br>E: 2	Postpartum haemorrhage	Antenatal<br>Intrapartum
Self-administration of malaria intermittent preventive treatment for pregnancy (IPTp)	All doses distributed to be administered at home, without direct observation	2 (0.4%)<br>P: 2<br>E: 0	Low birth weight, malaria	Antenatal
Self-administration of HIV oral pre-exposure prophylaxis (PrEP)	Daily oral tenofovir disoproxil fumarate/emtricitabine (TDF/FTC) for women with high risk of HIV acquisition	4 (1%)<br>P: 3<br>E: 1	HIV prevention	Preconception<br>Antenatal<br>Postpartum
**Self-testing or sampling**		**18 (3%)<br>P: 18<br>E: 0**		
pH-sensitive panty liner	To identify vaginal discharge of abnormal pH	1 (0.2%)<br>P: 1<br>E: 0	Amniotic fluid leakage	Antenatal
Self-collection of nasal samples	Self-collecting samples to be sent to a central laboratory for testing, as opposed to clinic-based collection by health providers	2 (0.4%)<br>P: 2<br>E: 0	Viral upper respiratory tract infections	Antenatal
Self-collection of urine/vaginal samples	Self-collecting samples at clinic visits, as opposed to collection by health providers	5 (1%)<br>P: 5<br>E: 0	Bacterial vaginosis, chlamydia, human papillovirus	Antenatal
Self-testing for HIV	For couple self-testing, studies only providing testing rates by the partner were excluded.	5 (1%)<br>P: 5<br>E: 0	HIV	Antenatal<br>Postpartum
Self-testing for malaria	Using rapid diagnostic test kits	1 (0.2%)<br>P: 1<br>E: 0	Malaria	Antenatal
Self-testing for proteinuria	Using urinalysis dipstick-based test kits	2 (0.4%)<br>P: 2<br>E: 0	Hypertensive disorders of pregnancy, proteinuria	Antenatal
Self-testing of vaginal pH	Using pH-sensitive dipsticks	2 (0.4%)<br>P: 2<br>E: 0	Bacterial vaginosis, preterm birth	Antenatal
**Other self-management skills**		**54 (10%)<br>P: 34<br>E: 20**		
Birth plans	Written plans to communicate preferences and goals during labour and childbirth	2 (0.4%)<br>P: 2<br>E: 0	Positive labour outcomes, psychological wellbeing	Antenatal<br>Intrapartum
Birth position	Choosing upright versus recumbent position during childbirth	12 (2%)<br>P: 6<br>E: 6	Duration of labour, positive labour outcomes	Intrapartum
Techniques during childbirth (not otherwise specified)	Correct pushing techniques and timing, walking and changing positions frequently, emptying bladder	3 (1%)<br>P: 2<br>E: 1	Lumbopelvic pain, positive childbirth experience	Antenatal<br>Intrapartum
Estimation of blood loss during birth by family members	Either by visual estimation, gravimetric methods or using a calibrated drape/collecting pouch. Can be administered by a family member if birth attendants are not available	6 (1%)<br>P: 5<br>E: 1	Postpartum haemorrhage	Intrapartum<br>Postpartum
First-aid for complications by family members - Naturally-induced oxytocin release	To increase uterine tone in postpartum haemorrhage. Achieved via breast, nipple or uterine stimulation. Can be self-administered or administered by partner if birth attendants are not available	6 (1%)<br>P: 3<br>E: 3	Postpartum haemorrhage	Postpartum

Self-care intervention	Description	No. of studies (%, N=545)	Health issues / outcomes targeted by intervention	Periods of intervention*
First-aid for complications by family members (not otherwise specified)	Basic first aid training for major postpartum complications (postpartum haemorrhage, postpartum infection, seizures, etc.)	1 (0.2%)<br>P: 1<br>E: 0	Various maternal & neonatal outcomes	Antenatal
Labour induction - Castor oil	Stimulates cervical ripening via PGE2 pathway	3 (1%)<br>P: 2<br>E: 1	Duration of labour	Intrapartum
Labour induction - Naturally-induced oxytocin release	Via breast or nipple stimulation, uterine stimulation or coitus	4 (1%)<br>P: 3<br>E: 1	Duration of labour	Antenatal<br>Intrapartum
Making medical decisions	Making decisions for oneself or the foetus/newborn e.g. prenatal screening, mode of delivery, etc.	12 (2%)<br>P: 7<br>E: 5	Decisional conflict, positive childbirth experience	Preconception<br>Antenatal
Postoperative wound care	Cleaning the wound area, identifying signs of infection. Applicable to C-section/episiotomy wounds and perineal injuries	2 (0.4%)<br>P: 1<br>E: 1	C-section wound healing, postpartum healing & pain relief	Antenatal<br>Postpartum
Self-management of asthma	Self-managing symptoms and medications, personalised action plan for attack episodes	3 (1%)<br>P: 2<br>E: 1	Asthma control	Antenatal

* If an intervention involves providing training or educating pregnant women in the antenatal period for self-care skills to be used in the postpartum period, the period of intervention will be classified as both "Antenatal" and "Postpartum".

† P: number of primary studies (interventional, observational and diagnostic accuracy studies); E: number of evidence synthesis studies (systematic reviews, meta-analyses and scoping reviews). Some primary studies (n=45, 8%) are also included in evidence synthesis studies featured in this review.

For peer review only

Supplementary File S11. Categorising self-care interventions based on WHO's classification framework

Category	N	%
**1.1 Individual agency**		
Interventions to promote awareness about self-care	0	0%
Interventions to promote autonomy and/or confidence to engage in self-care *<br>Interventions for improving self-care capacities and capabilities *	325	56%
Interventions for improving health and digital literacy	0	0%
Interventions to promote sustained adoption of self-care practices and behaviours	0	0%
**1.2 Health information seeking**		
Acquiring health education for informed health decision-making *<br>Improving health literacy to support health decision-making *	22	4%
Individuals seeking health information via on-demand information services	0	0%
Individuals health decision-making support via online or mobile tools	46	8%
**1.3 Social and community support**		
Peer mentorship and counselling	1	0.2%
Peer-to-peer action to support access to and the uptake and use of self-care interventions	42	7%
Individuals sharing health data with peers	3	1%
**1.4 Personal health tracking**		
Home-based record for health and diagnostic data	39	7%
Self-monitoring of health	70	12%
Active data capture/documentation by self-care user	32	6%
Passive data capture/documentation by device	9	2%
Use of diagnostic device at community location for self-measuring health indicator	0	0%
Tracking rational and responsible self-use of medicines and therapeutics	8	1%
**1.5 Self-diagnosis of health conditions**		
Self-testing, including to inform prevention	11	2%
Self-examining for health conditions	1	0%
Self-collection of samples to send for external testing	7	1%
Using online symptom checkers and health information	0	0%
**1.6 Self-management of health**		
Self-care for long-term health conditions	86	15%
Self-care for short-term health conditions	189	33%
Self-medication/treatment without medical prescription including for side effects	16	3%
Self-regulation of health conditions	148	26%
Self-care prevention including risk avoidance and support for physical and mental health and well-being	296	51%
Health management performed by caregivers	16	3%
Individuals procurement of safe over-the-counter medicines and products for self-care	106	18%

For peer review only - http://bmjopen.bmj.com/site/about/guidelines.xhtml

Category	N	%
**1.7 Individuals linkage to health system**		
Individuals-initiated communication with health system	5	1%
Individuals sharing health data with health workers	38	7%
Individuals identifying location of health facilities/structures	0	0%
Individuals receiving feedback from health workers	60	10%
**1.8 Individuals financial transactions for health** ***-not within the scope of inclusion of this review-***		
Expenses for over-the-counter health medicines and products that do not require prescription	0	0%
Availability or management of vouchers to individuals(s) for health services	0	0%
Expenses for prescription medicines	0	0%
Access to health insurance and/or adequate health insurance coverage	0	0%
Expenses for indirect health costs	0	0%

* These categories were considered to be equivalent during classifying, as there was insufficient information on how to differentiate between the two categories.

Discussion

The categories was based on WHO's guideline on classification of self-care interventions[1], which was published in 2021 as an initiative to standardise the language used in researching and reporting about self-care interventions. The framework describes four broad types of self-care interventions (interventions by self-carers and caregivers, by health workers, by health programme managers and by health policymakers/ legislators/regulators). For our review, we used the categories under self-care interventions for self-carers and caregivers. In classifying each study, we examined the description of its intervention in both the article and the associated protocol (if available).

As no standardised definition was provided in WHO's framework, liberty was taken in interpreting the meaning of these self-care categories when classifying our studies. For example, for interventions under category "Individual agency", we assume that these refer to interventions to raise public awareness or health/digital literacy about self-care, such as a public campaign or education programme. Therefore, digital platforms or education workshops targeting specific health issues (e.g. diabetes management or weight maintenance) is not classified under these categories.

Percentages were calculated based on the total number of studies (n=580). The percentages in the table above do not add up to 100% in some instances because some interventions can match to multiple WHO categories. For example, an intervention to increase physical activities involving a support group and a mobile app will be classified under "Self-regulation of health conditions" and "Peer-to-peer action to support access to and the uptake and use of self-care interventions".

There are several likely reasons for an absence of studies (0%) under some categories.

(a) For category 1.8 - Individuals financial transactions for health, the focus is on economic interventions, which is determined *a priori* as outside the scope of our review.

For peer review only - http://bmjopen.bmj.com/site/about/guidelines.xhtml

(b) For other categories, it can be interpreted as our review did not find any corresponding evidence of effectiveness or safety in the literature. Nonetheless, it is possible that qualitative or economic evidence still exists for these categories.

Reference

1 World Health Organization. Classification of self-care interventions for health: a shared language to describe the uses of self-care interventions. Geneva, 2021 https://www.who.int/publications/i/item/9789240039469 (accessed April 6, 2022).

For peer review only

Preferred Reporting Items for Systematic reviews and Meta-Analyses extension for Scoping Reviews (PRISMA-ScR) Checklist

SECTION	ITEM	PRISMA-ScR CHECKLIST ITEM	REPORTED ON PAGE #
**TITLE**			
Title	1	Identify the report as a scoping review.	1
**ABSTRACT**			
Structured summary	2	Provide a structured summary that includes (as applicable): background, objectives, eligibility criteria, sources of evidence, charting methods, results, and conclusions that relate to the review questions and objectives.	2-3
**INTRODUCTION**			
Rationale	3	Describe the rationale for the review in the context of what is already known. Explain why the review questions/objectives lend themselves to a scoping review approach.	4
Objectives	4	Provide an explicit statement of the questions and objectives being addressed with reference to their key elements (e.g., population or participants, concepts, and context) or other relevant key elements used to conceptualize the review questions and/or objectives.	4
**METHODS**			
Protocol and registration	5	Indicate whether a review protocol exists; state if and where it can be accessed (e.g., a Web address); and if available, provide registration information, including the registration number.	5<br>Supp File S1
Eligibility criteria	6	Specify characteristics of the sources of evidence used as eligibility criteria (e.g., years considered, language, and publication status), and provide a rationale.	5-7<br>Supp File S3
Information sources*	7	Describe all information sources in the search (e.g., databases with dates of coverage and contact with authors to identify additional sources), as well as the date the most recent search was executed.	5<br>Supp File S2
Search	8	Present the full electronic search strategy for at least 1 database, including any limits used, such that it could be repeated.	Supp File S2
Selection of sources of evidence†	9	State the process for selecting sources of evidence (i.e., screening and eligibility) included in the scoping review.	6-7
Data charting process‡	10	Describe the methods of charting data from the included sources of evidence (e.g., calibrated forms or forms that have been tested by the team before their use, and whether data charting was done independently or in duplicate) and any processes for obtaining and confirming data from investigators.	6-7<br>Supp File S4
Data items	11	List and define all variables for which data were sought and any assumptions and simplifications made.	7<br>Supp File S4
Critical appraisal of individual sources of evidence§	12	If done, provide a rationale for conducting a critical appraisal of included sources of evidence; describe the methods used and how this information was used in any data synthesis (if appropriate).	N/A
Synthesis of results	13	Describe the methods of handling and summarizing the data that were charted.	7
**RESULTS**			
Selection of sources of evidence	14	Give numbers of sources of evidence screened, assessed for eligibility, and included in the review, with reasons for exclusions at each stage, ideally using a flow diagram.	8<br>Fig 1
Characteristics of sources of evidence	15	For each source of evidence, present characteristics for which data were charted and provide the citations.	N/A
Critical appraisal within sources of evidence	16	If done, present data on critical appraisal of included sources of evidence (see item 12).	N/A
Results of individual sources of evidence	17	For each included source of evidence, present the relevant data that were charted that relate to the review questions and objectives.	N/A

For peer review only

SECTION	ITEM	PRISMA-ScR CHECKLIST ITEM	REPORTED ON PAGE #
Synthesis of results	18	Summarize and/or present the charting results as they relate to the review questions and objectives.	8-12
**DISCUSSION**			
Summary of evidence	19	Summarize the main results (including an overview of concepts, themes, and types of evidence available), link to the review questions and objectives, and consider the relevance to key groups.	13-4
Limitations	20	Discuss the limitations of the scoping review process.	14-15
Conclusions	21	Provide a general interpretation of the results with respect to the review questions and objectives, as well as potential implications and/or next steps.	15
**FUNDING**			
Funding	22	Describe sources of funding for the included sources of evidence, as well as sources of funding for the scoping review. Describe the role of the funders of the scoping review.	16

From: Tricco AC, Lillie E, Zarin W, O'Brien KK, Colquhoun H, Levac D, et al. PRISMA Extension for Scoping Reviews (PRISMAScR): Checklist and Explanation. Ann Intern Med. 2018;169:467–473. doi: 10.7326/M18-0850.

For peer review only
